# Human-specific protein isoforms produced by novel splice sites in the human genome after the human-chimpanzee divergence

**DOI:** 10.1186/1471-2105-13-299

**Published:** 2012-11-13

**Authors:** Dong Seon Kim, Yoonsoo Hahn

**Affiliations:** 1 Department of Life Science, Research Center for Biomolecules and Biosystems, Chung-Ang University, Seoul 156-756, Korea

**Keywords:** Splice site, Human genome, Protein variant, Molecular evolution

## Abstract

**Background:**

Evolution of splice sites is a well-known phenomenon that results in transcript diversity during human evolution. Many novel splice sites are derived from repetitive elements and may not contribute to protein products. Here, we analyzed annotated human protein-coding exons and identified human-specific splice sites that arose after the human-chimpanzee divergence.

**Results:**

We analyzed multiple alignments of the annotated human protein-coding exons and their respective orthologous mammalian genome sequences to identify 85 novel splice sites (50 splice acceptors and 35 donors) in the human genome. The novel protein-coding exons, which are expressed either constitutively or alternatively, produce novel protein isoforms by insertion, deletion, or frameshift. We found three cases in which the human-specific isoform conferred novel molecular function in the human cells: the human-specific IMUP protein isoform induces apoptosis of the trophoblast and is implicated in pre-eclampsia; the intronization of a part of *SMOX* gene exon produces inactive spermine oxidase; the human-specific NUB1 isoform shows reduced interaction with ubiquitin-like proteins, possibly affecting ubiquitin pathways.

**Conclusions:**

Although the generation of novel protein isoforms does not equate to adaptive evolution, we propose that these cases are useful candidates for a molecular functional study to identify proteomic changes that might bring about novel phenotypes during human evolution.

## Background

Humans have many unique traits compared with those of other primates that must be derived from genetic changes acquired during human evolution [1]. These genetic modifications include accelerated amino acid substitutions [2,3], *de novo* origin of protein-coding genes from non-coding sequences [4], formation of novel transcript variants by DNA insertion [5], and inactivation of long-established genes [6,7].

The generation of novel alternative splice sites plays a role in the evolution of gene structure [8,9]. Alternative usage of splice sites often results in insertion or deletion of amino acids and/or frameshift in proteins. Generation of novel splice sites or activation of cryptic splice sites in transposable elements such as Alu repeats is rather common in human genes [10-13]. These sites are often alternatively spliced, as the novel Alu splice sites are generally weak donors or acceptors and/or the insertion of Alu-derived fragments into the coding region of genes generally results in disruption of the host proteins [14,15]. There are reports on novel exons originating from non-coding intronic sequences in some organisms such as rodents and humans [16,17]. However, these are not species-specific but have been originated during rodent and primate evolution, respectively; the novel “human” exons reported by Zhang and Chasin [17] originated before the human-chimpanzee divergence.

Although many reports are available on novel exons originating during human evolution, most of them have no evidence of protein-coding capability or are not human-specific. In this study, we hypothesized that nucleotide changes in the human genome after the human-chimpanzee divergence may have generated novel splice sites and produced novel protein-coding exons. To find such cases, we analyzed annotated human protein coding exons and their orthologous genomic sequences of other primates including chimpanzee and some non-primate mammals. We examined possible changes in the proteins caused by the formation of novel splice sites.

## Results and discussion

### Human-specific splice sites

We analyzed multiple alignments of human protein-coding exons and their respective orthologous sequences from various primate and mammalian genomes. We identified 50 canonical splice acceptors (AG) and 35 canonical splice donors (GT) that had newly arisen in the human genome after the human-chimpanzee divergence. Lists of the novel splice acceptors and donors reported in this study are presented in Additional file 1: Table S1 and in Additional file 2: Table S2, respectively.

In the present study, we aimed to collect highly plausible cases for generation of novel protein-coding exons induced by human-specific splice sites. To achieve this goal, we used annotated protein-coding exons as the initial data set, excluding exons without annotated coding regions such as alternative exons that were obtained from expressed sequence tag (EST) data analysis. We also employed highly stringent filtering conditions for subsequent analyses. For example, we discarded exons that were supported by only a single transcript record, because these exons are likely to be derived from noisy splicing events [18]. We also excluded exons that were derived from repetitive elements, although many novel exons were reported to be derived from repetitive elements such as Alu or L1 [5,10-13]. Repetitive elements are frequently exapted as coding exons during evolution [19]; however, the vast majority of repeats that appeared within the coding region of mRNAs cause a frameshift or a premature termination codon [15]. Therefore, we only focused on novel coding exons of which their origin was from a non-repetitive genomic region.

In this study, we only collected canonical splice sites (GT-AG) although there are many exons flanked by non-canonical minor splice sites such as GC-AG or AT-AC [18]. In the early phase of this study, we collected all the human splice junction sequences that were different from the chimpanzee sequences as candidates for human-specific splice sites. We found that the most of non-canonical human splice sites were not one of known minor splice sites but random sequences. We assumed this was due to intrinsic errors in the input data such as incomplete genome sequence, incorrect alignment, and non-orthologous alignment. Because we aimed to collect highly accurate cases, we only considered canonical splice sites.

**Figure 1:**
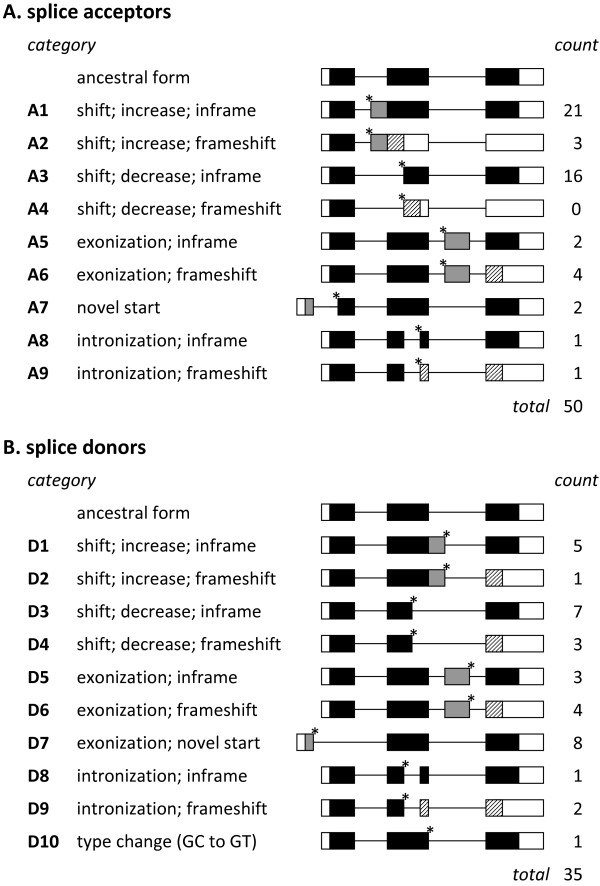
**Summary of splice site generation and protein modification.** This figure summarizes the categories of transcript structures modified by the generation of novel splice acceptors (**A**) and donors (**B**) in human proteins. The left column shows a summary of the modification with the category codes A1–D10. The center column shows a schematic of the transcript and coding region structures. Novel splice sites are marked by asterisks. The ancestral coding regions (black), derived coding regions (grey), alternative coding regions (hatched), and untranslated regions (white) are represented by boxes with different patterns. The last column “count” shows the number of cases reported in this study.

**Figure 2:**
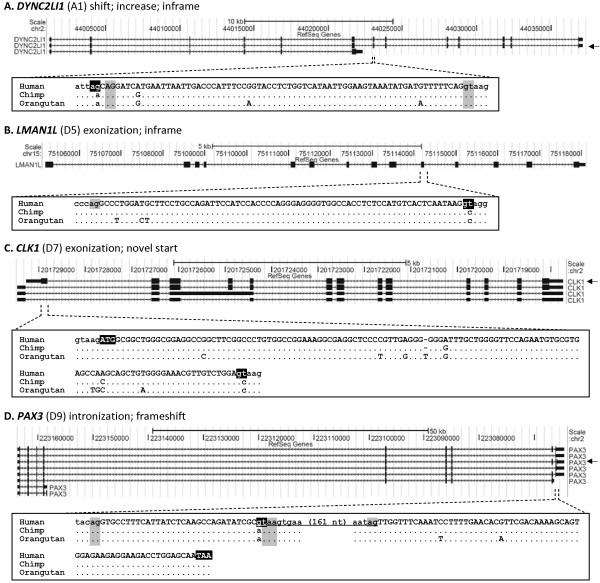
**Examples of human-specific protein-coding exons with novel splice sites.** In each part, the top panel shows the exonic structures of the human *DYNC2LI1* (**A**), *LMAN1L* (**B**), *CLK1* (**C**), and *PAX3* (**D**) gene transcripts. The transcript isoform with a novel protein-coding exon is marked by an arrow at the right. A multiple sequence alignment of the orthologous segments from the human, chimpanzee, and orangutan genomes is shown below each panel (see Additional file 2: Tables S2A and S2B for the alignment of all species examined). The coding regions of the human-specific exon is in uppercase. The novel splice sites (gt and ag), start codon (ATG), and stop codon (TAA) in humans are highlighted in black. The conserved ancestral splice sites, stop codon (taa in D), and human cryptic splice sites are highlighted in grey. The underlined sequence in the human *PAX3* (**D**) gene indicates the intronized region. Dots indicate that the sequences are the same as the human sequence.

In all the cases except one, the human-specific splice sites reported in this study were associated with genes conserved in mammals. Only one gene (*C14orf182*) appeared to be a *de novo* protein-coding gene derived from a non-coding sequence. Out of 85 derived human-specific exons, 42 were annotated as a part of a RefSeq transcript. Utilization of novel splice sites results in various modifications such as splice site shift of ancestral exons, generation of novel exons (exonization) or intronization of exonic segments. We classified the protein modification types into 19 categories, which are presented in Figure 1. The detailed information on representative cases is shown in Figure 2 and Additional file 3: Figures S1–S9. Multiple sequence alignments of all the novel splice sites and associated exons are presented in Additional file 4: Table S3 (splice acceptor sites) and in Additional file 5: Table S4 (splice donor sites).

**Figure 3:**
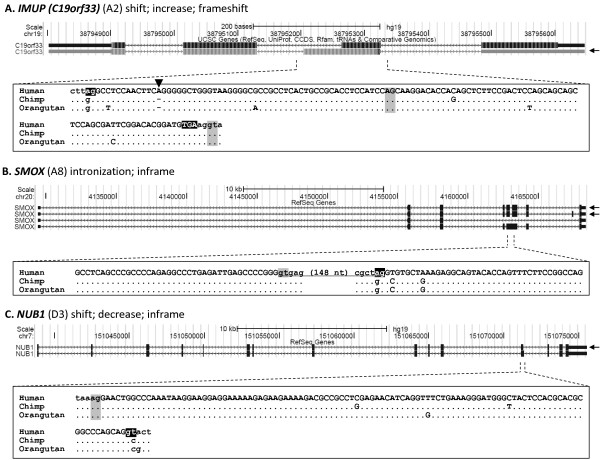
**Human-specific protein isoforms with reported molecular function.** The human-specific coding exons for the IMUP isoform 2 (IMUP-2) (**A**), SMOX isoforms 2 and 4 (**B**), and the NUB1 isoform 2 (**C**) are presented. The transcript isoform with a novel protein-coding exon is marked by an arrow at the right. The downward arrowhead in the IMUP-2 exon indicates the human-specific insertion of an adenine nucleotide. The coding region of the human-specific exon is in uppercase. The novel splice sites (gt and ag) and the stop codon (TGA in IMUP-2) in humans are highlighted in black. The conserved ancestral and human cryptic splice sites are highlighted in grey. The underlined sequence in the human *SMOX* (**B**) gene indicates the intronized region. Dots indicate that the sequences are the same as the human sequence

In 18 cases, the human-specific splice sites caused a frameshift compared with the ancestral open reading frame. A frameshift mutation often results in premature termination codon and induces nonsense-mediated mRNA decay (NMD) [20]. Generally, a stop codon which is situated at 50–55 nucleotides upstream of the last exon-exon junction can induce NMD. We looked at the stop codon position of the 18 human-specific transcripts and found that in all but one case the stop codon was in the last exon. In one case, the *IMUP* gene transcript variant, the stop codon is in an internal exon. However, it is very close to the last exon-exon junction (2 nucleotides upstream) and would not trigger NMD (see Figure 3A). Therefore, none of the human-specific transcripts in this study is susceptible to NMD, suggesting the human-specific transcripts are stable in the human cells.

In nine cases, we found a splice site-associated polymorphism in the human population, indicating that these human-specific transcripts are differentially expressed in human individuals. The frequencies of the derived allele that generated a human-specific splice site were: *PLP1* (dbSNP accession number rs2233697, AG vs GG), 98.804%; *TXNDC16* (rs28759013, AG vs AA), 96.350%; *KHK* (rs74537742, AG vs GG), 95.256%; *IL12RB1* (rs393548, AG vs GG), 80.378%; *HOXD1* (rs13390932, GT vs GC), 75.443%; *XRCC4* (rs1805377, AG vs AA), 65.843%; *NIPAL2* (rs3735887, GT vs AT), 46.445%; *MRE11A* (rs496797, GT vs AT), 46.374%; and *STXBP4* (rs11658717, AG vs AA), 20.947%.

### Splice site shift and type change

The generation of a novel splice site close to the ancestral site of an exon causes a splice site shift that either lengthens or shortens the affected exon (category codes A1–A4 and D1–D4; see Figure 1 for details). Fifty-six of 85 novel splice sites resulted in shifting of splice sites (see Figure 2A and Additional file 3: Figures S1–S4 for representative examples). In 50 cases, the derived exon increased or decreased in size without changing the downstream reading frame.

In 20 cases, the splice site was shifted by three bases, producing tandem splice sites known as NAGNAG acceptors and GTNGTN donors [21-23]. Alternative usage of these splice sites generated single amino acid insertion and deletion isoforms. We found 16 NAGNAG acceptors and four GTNGTN donors (see Figure 2A and Additional file 3: Figures S1 and S2). Usually, the addition or deletion of a single codon does not affect protein function. However, when a functionally important residue is removed or a nonsense codon is destroyed or added, the result can be substantial and cause diseases in humans [21,24]. There was no premature stop codon in the cases reported in this study. The TassDB2 (http://www.tassdb.info) is a comprehensive database of tandem splice sites in human and mouse [25]. Out of 20 tandem splice sites, 18 were recorded in the TassDB2. However, it is not previously reported that these splice sites are human-specific.

We found a GC-to-GT change in the splice donor of exon 45 of the human *DOCK1* gene (see Additional file 3: Figure S9). However, the orthologous mammalian exon used GC as the splice donor so that the exon boundaries are conserved in human and mammals. Thus, the ancestral GC-AG intron had changed to a canonical GT-AG intron in humans. Although both the GC-AG and GT-AG splice sites are processed by the standard U2-type spliceosome, the strength of the donor site could be affected [26].

### Exonization and intronization

In 21 cases, the novel splice sites (six acceptors and 15 donors) arose in the non-coding region resulting in exonization of intronic segments (categories A5–A9 and D5–D9; see Figure 1 for details). A cryptic intronic splice donor for a given novel acceptor or a cryptic acceptor for a novel donor was activated in the human genome. The 13 cases generated novel internal exons that added some additional amino acids (five cases) or produced alternative C-termini by frameshift (eight cases) (see Figure 2B and Additional file 3: Figures S5 and S6). In the remaining eight cases, the exons were associated with cryptic or authentic promoters and produced alternative N-termini of the proteins (see Figure 2C and Additional file 3: Figure S7).

Interestingly, in five cases, novel splice acceptors or donors appeared in the exons, and corresponding cryptic exonic donors or acceptors were accordingly activated. As a result, part of the exonic sequence was spliced out as an intron (see Figure 2D and Additional file 3: Figure S8).

### Novel molecular function of human-specific protein isoforms

We performed a literature review to find reported molecular functions of human-specific protein isoforms produced by human-specific splice sites. We found three cases (*IMUP*, *SMOX*, and *NUB1*) of which the molecular function of the human-specific protein isoform has been described.

The human *IMUP* (also known as *C19orf33*) gene encoding immortalization-up-regulated protein produces two protein isoforms reported as IMUP-1 and IMUP-2, respectively (http://www.uniprot.org/uniprot/Q9GZP8). Both isoforms are highly expressed in cancer cells and localized in the nucleus [27,28]. These two protein variants share 46 N-terminal amino acids but have different C-termini. The IMUP-2 protein (isoform 2 of IMUP) is a human-specific isoform that is produced by using a human-specific splice donor site (Figure 3A). IMUP-2 expression is specifically elevated during preterm pre-eclampsia and under hypoxic conditions, and the IMUP-2 protein induces apoptosis of the trophoblast [29]. Therefore, production of the IMUP-2 human-specific isoform may be functionally involved in placental development and gynecological diseases such as pre-eclampsia. This case clearly shows that a human-specific protein isoform induced by the generation of a novel splice site sequence plays a role in the human cells and is associated with a phenotype.

The human *SMOX* gene encoding spermine oxidase is implicated in various tumors and diseases in humans [30-32]. Alternative splicing of the human *SMOX* gene transcripts results in the production of six protein isoforms (http://www.uniprot.org/uniprot/Q9NWM0). A part of exon 5 can act as an intron and is spliced out to produce SMOX protein isoforms 2 and 4 [33]. We found that this intronization of an internal part of exon 5 was induced by the generation of a human-specific splice donor sequence (Figure 3B). This intronization removed 53 amino acids from the ancestral form. Many of these residues are highly conserved components of the FAD-binding domain and are essential for catalysis. Therefore, the deletion caused by the intronization seemed to result in the production of catalytically inactive spermine oxidase protein [33,34]. The human-specific SMOX protein isoform 4 has been localized in the nucleus [34]. Although the human-specific SMOX isoforms are inactive as oxidases, it is possible that they could confer other molecular functions in the human cell in general, or that production of non-functional isoform could decrease the cellular concentration of active spermine oxidase and affect spermine catabolism.

The human *NUB1* gene encodes two isoforms of a negative regulator of ubiquitin-like proteins 1 (originally known as NEDD8 ultimate buster-1) [35]. Isoforms 1 (also known as NUB1L for larger variant) and 2 are 615-aa and 601-aa long, respectively. We identified that the 601-aa-long isoform 2 was produced using a human-specific splice donor site (Figure 3C). The NUB1 protein interacts with ubiquitin-like proteins such as NEDD8 and FAT10 and accelerates their degradation [36-38]. Downregulation of NEDD8 by NUB1 leads to decreased p53 modification, resulting in cytoplasmic localization and inhibition of p53 transcriptional activity [39]. The generation of the novel splice donor site within exon 12 resulted in shortening of the exon and removal of 14 amino acids from the ancestral form. Human-specific isoform 2 has been detected almost equally in all examined tissues and shows a higher expression level than that of ancestral isoform 1, which is not equally detected in tissues examined [35]. The 14-aa deletion removes one of the ubiquitin associated domains, probably resulting in weakening of the interaction between NUB1 and ubiquitin-like proteins. Actually, isoform 2 is less efficient in promoting degradation of NEDD8 [40]. Therefore, production of the human-specific NUB1 isoform 2 may affect the ubiquitin pathway in human cells.

## Conclusions

We identified 85 cases of annotated human protein-coding exons with evidence of substantial expression that had been generated by acquisition of a novel splice site in the human genome. The novel protein-coding exons were expressed either constitutively or alternatively. We found three cases in which the human-specific isoform conferred novel molecular function. We propose that the generation of novel splice sites contributes to the evolution of the human genome and the variety of protein isoforms that may furnish novel functional proteins and novel phenotypes.

## Methods

### Collection of human-specific splice site candidates

**Figure 4:**
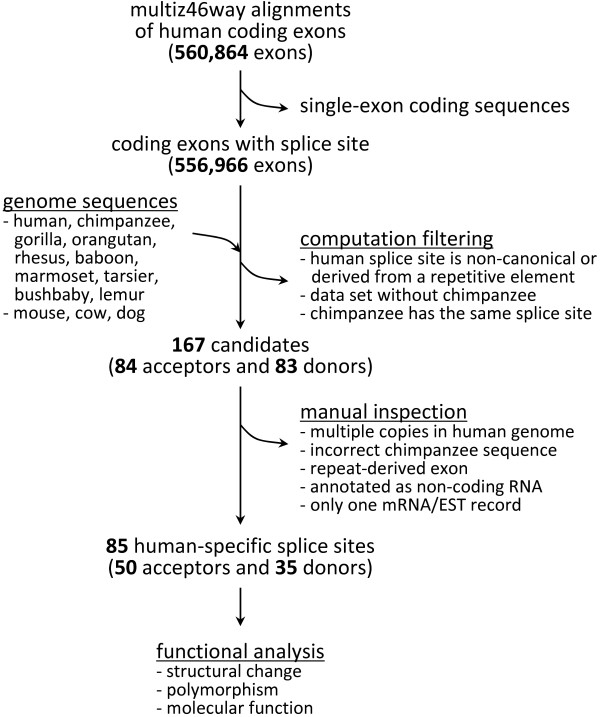
Procedure for identifying human-specific splice sites of annotated human proteins.

The overall procedure we employed in this study is presented in Figure 4. We downloaded the multiple alignment data of the human protein-coding exon sequences and their mammalian orthologous genome sequences from the University of California Santa Cruz (UCSC) Genome Brower Database (http://genome.ucsc.edu). The human coding regions were defined in the “knownGene” track, and the multiple sequence alignments of the coding regions were derived from the “multiz46way” alignment data. We extracted the protein-coding exon sequences, padded by 2 bp at both ends, from the respective genome assemblies. The genome assemblies analyzed in the present study included 10 primates and three other representative non-primate mammals: human (hg19), chimpanzee (panTro2), gorilla (gorGor1), orangutan (ponAbe2), rhesus macaque (rheMac2), baboon (papHam1), marmoset (calJac1), tarsier (tarSyr1), bushbaby, also known as galago (otoGar1), mouse lemur (micMur1), mouse (mm9), dog (canFam2), and cow (bosTau4).

We discarded splice sites derived from repetitive elements which were marked in lowercase in the repeat-masked genome sequences. When a human protein-coding exon has a canonical splice sequence (GT as the donor and AG as the acceptor), but the chimpanzee and other primates have a different sequence (other than GT or AG), we considered it to be a case of human-specific acquisition of a novel splice site after the human-chimpanzee divergence. We collected 167 human-specific splice site candidates (84 acceptors and 83 donors) by filtering the multiple alignment data.

### Manual inspection of the candidates

We scrutinized each case by visual inspection mainly based on data available in the UCSC Genome Browser. We discarded cases showing one of the following conditions: the novel human exon was derived from a repeat sequence (the exon overlaps a repetitive element in the “RepeatMasker” track); the orthologous chimpanzee genomic segment had a sequencing error (the “Quality Score” track of the chimpanzee genome was reviewed); the orthology relationship of the gene was ambiguous due to the presence of highly similar paralogous copies in the human genome (BLAT was used to map the sequence to the human genome); the corresponding human transcript was annotated as a non-coding RNA (RefSeq record was reviewed); or the human transcript was not supported by two or more transcript records in the database (human mRNA and EST tracks were analyzed). As a result, we identified 50 splice acceptors (AG) and 35 splice donors (GT) that were specific to humans.

We compared the structures of the derived human exons and corresponding ancestral mammalian genome sequences to classify the protein modification types. We examined human mRNA and EST tracks of the UCSC Genome Browser to determine whether a novel splice site is used alternatively or constitutively. For the human simple nucleotide polymorphism (SNP), we analyzed the “Common SNPs(135)” or “Common SNPs(132)” track of the UCSC Genome Browser. We referred to UniProt database (http://www.uniprot.org) for characterized molecular function of the human-specific protein isoforms.

## Competing interests

The authors declare that they have no competing interests.

## Authors’ contributions

YH conceived of the study, conducted the programming work, and prepared the manuscript. DSK participated in the sequence analysis and the literature survey. Both authors read and approved the final manuscript.

## Supplementary Material

Additional file 1 List of the human-specific splice acceptors reported in this study. Click here for file

Additional file 2 List of the human-specific splice donors reported in this study. Click here for file

Additional file 3 Examples of the human-specific splice sites. Click here for file

Additional file 4 Alignments of the exon and splice acceptor sequences. Click here for file

Additional file 5 Alignments of the exon and splice donor sequences. Click here for file
