# Supplementary material for: Human-specific protein isoforms produced by novel splice sites in the human genome after the human-chimpanzee divergence
Source: BMC Bioinformatics. 2012 Nov 13;13:299. doi: 10.1186/1471-2105-13-299 (PMC3538075; doi:10.1186/1471-2105-13-299)
Supplement: Additional file 1 — List of the human-specific splice acceptors reported in this study. [file 1471-2105-13-299-S1.html]

 
 Table S1. List of the human-specific splice acceptors reported in this study 

 Table S1. List of the human-specific splice acceptors reported in this study 

 
  No Exon ID Position (hg19) Dir Human acceptor Chimp acceptor Category Usage Gene symbol Protein accession mRNA accession Gene title Note
  1 uc001aoe.1_4_5  chr1:6692430  + AG TG (A1) shift; increase; inframe alternative THAP3 NP_612359.2  NM_138350.3 THAP domain-containing protein 3  
  2 uc001bfa.2_3_15  chr1:22047656  - AG AA (A3) shift; decrease; inframe alternative USP48 Q86UV5-5  AK021830.1 ubiquitin specific protease 48  
  3 uc001cax.1_15_15  chr1:36932509  - AG AA (A1) shift; increase; inframe alternative CSF3R NP_724781.1 NM_156039.3 granulocyte colony-stimulating factor receptor  
  4 uc001ctt.2_5_16  chr1:52859475  - AG GG (A1) shift; increase; inframe constitutive ORC1 NP_004144.2 NM_004153.3 origin recognition complex subunit 1  
  5 uc010ork.1_15_16  chr1:78338686  + AG AA (A1) shift; increase; inframe alternative FAM73A B7ZLZ8 BC144167.1 family with sequence similarity 73, member A NAGNAG
  6 uc001flb.2_9_11  chr1:155583216  + AG TG (A2) shift; increase; frameshift alternative MSTO1 Q9BUK6-7 AK056128.1 misato homolog 1  
  7 uc001gli.1_6_25  chr1:177930074  - AG GG (A3) shift; decrease; inframe alternative SEC16B NP_149118.2 NM_033127.2 protein transport protein Sec16B NAGNAG
  8 uc001how.2_33_83  chr1:225347031  + AG AA (A3) shift; decrease; inframe alternative DNAH14 NP_001364.1 NM_001373.1 dynein heavy chain 14, axonemal  
  9 uc002rin.2_3_8  chr2:27317342  + AG GG (A1) shift; increase; inframe alternative KHK P50053-2 BX427548.2 ketohexokinase NAGNAG, dbSNP:rs74537742
  10 uc002rtl.2_7_13  chr2:44023026  + AG AA (A1) shift; increase; inframe alternative DYNC2LI1 NP_001180393.1 NM_001193464.1 cytoplasmic dynein 2 light intermediate chain 1 NAGNAG
  11 uc002smk.1_5_7  chr2:74758694  + AG GG (A1) shift; increase; inframe alternative HTRA2 O43464-3 AF141306.1 HtrA serine peptidase 2  
  12 uc002tzo.2_3_9  chr2:158958552  + AG GA (A7) novel start alternative UPP2 NP_001128570.1 NM_001135098.1 uridine phosphorylase 2  
  13 uc002vnu.2_6_8  chr2:224845117  - AG AA (A1) shift; increase; inframe alternative SERPINE2 NP_006207.1 NM_006216.3 serpin peptidase inhibitor, clade E (nexin, plasminogen activator inhibitor type 1), member 2 NAGNAG
  14 uc003epq.2_3_5  chr3:133302846  + AG AA (A1) shift; increase; inframe alternative CDV3 NP_001127894.1 NM_001134422.1 protein CDV3 homolog NAGNAG
  15 uc003gay.2_6_10  chr4:678391  - AG CG (A1) shift; increase; inframe alternative MFSD7 Q6UXD7 AY203936.1 major facilitator superfamily domain containing 7 NAGNAG
  16 uc003htb.3_18_23  chr4:95200074  + AG GG (A1) shift; increase; inframe alternative SMARCAD1 NP_001121902.1 NM_001128430.1 SWI/SNF-related matrix-associated actin-dependent regulator of chromatin subfamily A containing DEAD/H box 1  
  17 uc011chk.1_3_6  chr4:141484669  - AG AA (A3) shift; decrease; inframe alternative UCP1 Q4KMT7 BC096736.1 uncoupling protein 1 NAGNAG
  18 uc003kib.2_7_7  chr5:82648944  + AG GA (A1) shift; increase; inframe alternative XRCC4 NP_071801.1 NM_022406.2 X-ray repair complementing defective repair in Chinese hamster cells 4 dbSNP:rs1805377
  19 uc010kiv.2_6_61  chr6:152629763  - AG GG (A3) shift; decrease; inframe alternative SYNE1 NP_149062.1 NM_033071.3 spectrin repeat containing, nuclear envelope 1  
  20 uc003svh.2_2_4  chr7:22533398  - AG AC (A3) shift; decrease; inframe alternative STEAP1B NP_997225.1 NM_207342.2 STEAP family member 1B  
  21 uc003wia.1_3_4  chr7:150557532  + AG GG (A1) shift; increase; inframe alternative ABP1 P19801-2 U11863.1 amiloride-sensitive amine oxidase [copper-containing]  
  22 uc004amn.2_3_4  chr9:84605332  + AG TG (A5) exonization; inframe alternative FAM75D1 NP_001001670.1 NM_001001670.2 family with sequence similarity 75, member D1  
  23 uc001nyl.2_9_14  chr11:63987351  + AG CG (A1) shift; increase; inframe alternative FERMT3 NP_848537.1 NM_178443.2 fermitin family homolog 3 long form  
  24 uc001nzq.1_4_6  chr11:64082213  + AG CG (A1) shift; increase; inframe alternative ESRRA NP_004442.3 NM_004451.3 steroid hormone receptor ERR1 NAGNAG
  25 uc001pqi.1_14_27  chr11:117059445  + AG GG (A3) shift; decrease; inframe alternative SIDT2 Q24JR2 BC114522.1 SID1 transmembrane family, member 2  
  26 uc009zhm.1_7_7  chr12:10594899  - AG AC (A6) exonization; frameshift alternative KLRC1 A8MYW4 AK310992.1 killer cell lectin-like receptor subfamily C, member 1  
  27 uc001rqw.2_16_17  chr12:48440231  - AG AA (A3) shift; decrease; inframe alternative SENP1 NP_055369.1 NM_014554.2 sentrin-specific protease 1 NAGNAG
  28 uc010sxw.1_9_15  chr12:110465513  + AG GG (A1) shift; increase; inframe alternative ANKRD13A NP_149112.1 AF155103.1  ankyrin repeat domain-containing protein 13A NAGNAG
  29 uc010agc.2_7_7  chr13:103491901  + AG AC (A1) shift; increase; inframe alternative BIVM NP_001153068.1 NM_001159596.1 basic immunoglobulin-like variable motif-containing protein  
  30 uc010tqu.1_5_19  chr14:52985996  - AG AA (A3) shift; decrease; inframe alternative TXNDC16 NP_001153519.1 NM_001160047.1 thioredoxin domain-containing protein 16 dbSNP:rs28759013
  31 uc001xpi.2_13_13  chr14:74426256  - AG AA (A6) exonization; frameshift alternative ENTPD5 O75356 BC020966.2 ectonucleoside triphosphate diphosphohydrolase 5  
  32 uc001xxw.1_6_6  chr14:90391021  - AG GG (A6) exonization; frameshift alternative C14orf143 Q9BUY7-3 AK298930.1 hypothetical protein LOC90141 (EF-hand calcium-binding domain-containing protein 11)  
  33 uc002elm.1_2_8  chr16:57473207  - AG GG (A3) shift; decrease; inframe alternative CIAPIN1 Q6FI81 AL136613.1 cytokine induced apoptosis inhibitor 1  
  34 uc010wbi.1_2_8  chr17:28323324  + AG AT (A1) shift; increase; inframe alternative EFCAB5 B5MEA3 AK302745.1 EF-hand calcium binding domain 5  
  35 uc010whf.1_3_5  chr17:41059617  + AG GG (A3) shift; decrease; inframe alternative G6PC B4E1C3 AK303771.1 glucose-6-phosphatase  
  36 uc010dcc.1_2_8  chr17:53076987  + AG AA (A1) shift; increase; inframe alternative STXBP4 AAH41485.1 BC041485.2 syntaxin-binding protein 4 dbSNP:rs11658717
  37 uc002luw.1_9_10  chr19:2098975  + AG AA (A5) exonization; inframe alternative IZUMO4 NP_001026905.2 NM_001039846.1 izumo sperm-egg fusion protein 4  
  38 uc002nhx.1_2_18  chr19:18197742  - AG GG (A7) novel start alternative IL12RB1 P42701 BX647221.1  interleukin 12 receptor, beta 1 dbSNP:rs393548
  39 uc002ohv.1_3_3  chr19:38795205  + AG GG (A2) shift; increase; frameshift alternative IMUP Q9GZP8-2 AB038318.1 Immortalization up-regulated protein  
  40 uc002qfm.1_3_10  chr19:54872813  - AG GG (A3) shift; decrease; inframe alternative LAIR1 Q6GTX8-3 AF251509.2 Leukocyte-associated immunoglobulin-like receptor 1 NAGNAG
  41 uc002qiy.2_10_22  chr19:55606968  - AG TG (A3) shift; decrease; inframe alternative PPP1R12C Q9BZL4-3 AB209452.1 protein phosphatase 1, regulatory subunit 12C NAGNAG
  42 uc002wkk.1_5_8  chr20:4163130  + AG GG (A8) intronization; inframe alternative SMOX NP_787034.1 NM_175840.1 spermine oxidase  
  43 uc010zta.1_13_14  chr20:25205798  + AG GG (A2) shift; increase; frameshift alternative ENTPD6 B4DDM7 AK293256.1 ectonucleoside triphosphate diphosphohydrolase 6  
  44 uc002xvp.1_4_4  chr20:49457074  + AG AA (A6) exonization; frameshift alternative BCAS4 Q8TDM0-2 BC056883.1 breast carcinoma amplified sequence 4  
  45 uc010gpg.1_8_11  chr21:44274733  - AG GG (A3) shift; decrease; inframe alternative WDR4 P57081-2 AK292923.1 WD repeat domain 4 NAGNAG
  46 uc010guy.1_14_40  chr22:26231260  + AG AC (A1) shift; increase; inframe alternative MYO18B B0QYF5 AY077700.1 myosin XVIIIB NAGNAG
  47 uc010gwc.1_6_14  chr22:31285164  + AG TG (A1) shift; increase; inframe alternative OSBP2 NP_110385.1 NM_030758.3 oxysterol-binding protein 2 NAGNAG
  48 uc011aqc.1_2_7  chr22:44285375  - AG GG (A3) shift; decrease; inframe alternative PNPLA5 NP_001171146.1 NM_001177675.1 patatin-like phospholipase domain-containing protein 5  
  49 uc003bjv.2_10_10  chr22:50637545  + AG AA (A9) intronization; frameshift alternative TRABD Q9H4I3 BI909982.1 TraB domain containing  
  50 uc011msf.1_2_7  chrX:103040676  + AG GG (A3) shift; decrease; inframe alternative PLP1 B4DI30 AK295388.1 proteolipid protein 1 dbSNP:rs2233697
 
