# Supplementary material for: Human-specific protein isoforms produced by novel splice sites in the human genome after the human-chimpanzee divergence
Source: BMC Bioinformatics. 2012 Nov 13;13:299. doi: 10.1186/1471-2105-13-299 (PMC3538075; doi:10.1186/1471-2105-13-299)
Supplement: Additional file 2 — List of the human-specific splice donors reported in this study. [file 1471-2105-13-299-S2.html]

 
 Table S2. List of the human-specific splice donors reported in this study 

 Table S2. List of the human-specific splice donors reported in this study 

 
  No Exon ID Position (hg19) Dir Human donor Chimp donor Category Usage Gene symbol Protein accession mRNA accession Gene title Note
  1 uc001cjm.2_6_7  chr1:43917099  - GT GC (D4) shift; decrease; frameshift alternative HYI NP_112484.3 NM_031207.5 putative hydroxypyruvate isomerase  
  2 uc001gdg.2_1_6  chr1:165601511  + GT AT (D7) exonization; novel start alternative MGST3 Q5VV89 BG709310.1 microsomal glutathione S-transferase 3  
  3 uc010pkz.1_1_11  chr1:165667645  - GT GC (D3) shift; decrease; inframe alternative ALDH9A1 B4DX14 AK293520.1 aldehyde dehydrogenase 9 family, member A1  
  4 uc010pxj.1_9_10  chr1:232144729  + GT GC (D3) shift; decrease; inframe alternative DISC1 NP_001012975.1 NM_001012957.1 disrupted in schizophrenia 1 protein  
  5 uc010pyf.1_1_9  chr1:240408865  + GT AT (D7) exonization; novel start alternative FMN2 B7Z4S3 AK297755.1 formin 2  
  6 uc002tif.2_1_7  chr2:113514470  - GT CT (D8) intronization; inframe alternative CKAP2L Q8IYA6-2 AK097948.1 cytoskeleton associated protein 2-like  
  7 uc010fqy.2_2_3  chr2:177054409  + GT GC (D6) exonization; frameshift alternative HOXD1 B2RAB4 BC028190.1 homeobox D1 dbSNP:rs13390932
  8 uc010zhi.1_1_13  chr2:201728824  - GT GC (D7) exonization; novel start alternative CLK1 NP_001155879.1 NM_001162407.1 CDC-like kinase 1  
  9 uc002vmt.1_9_10  chr2:223066131  - GT AT (D9) intronization; frameshift alternative PAX3 NP_852124.1 NM_181459.3 paired box 3  
  10 uc011axk.1_2_17  chr3:33118586  - GT AT (D5) exonization; inframe alternative GLB1 B7Z6Q5 AK300753.1 galactosidase, beta 1  
  11 uc011bql.1_1_17  chr3:180633316  + GT GG (D7) exonization; novel start alternative FXR1 B4DXZ6 AK302197.1 fragile X mental retardation, autosomal homolog 1  
  12 uc011cfo.1_2_11  chr4:109683977  - GT GA (D2) shift; increase; frameshift alternative AGXT2L1 NP_001140099.1 NM_001146627.1 alanine-glyoxylate aminotransferase 2-like 1  
  13 uc003ldj.2_17_18  chr5:138268647  + GT GG (D6) exonization; frameshift alternative CTNNA1 P35221-2 U03100.1 catenin (cadherin-associated protein), alpha 1, 102kDa  
  14 uc011dbw.1_12_13  chr5:146763517  + GT AT (D4) shift; decrease; frameshift alternative STK32A B7Z9H7 AK315942.1 serine/threonine-protein kinase 32A  
  15 uc003pne.3_14_15  chr6:90338942  + GT GC (D3) shift; decrease; inframe alternative ANKRD6 NP_055757.3 NM_014942.4 ankyrin repeat domain-containing protein 6  
  16 uc011kma.1_7_8  chr7:107254177  + GT AT (D6) exonization; frameshift alternative BCAP29 NP_001008405.1 NM_001008405.2 B-cell receptor-associated protein 29  
  17 uc003wii.2_2_10  chr7:150725998  + GT TT (D5) exonization; inframe alternative ABCB8 Q6ZRM3 AK128129.1 ATP-binding cassette, sub-family B (MDR/TAP), member 8  
  18 uc003wjw.2_11_14  chr7:151071287  + GT GC (D3) shift; decrease; inframe alternative NUB1 NP_057202.3 NM_016118.4 negative regulator of ubiquitin-like proteins 1  
  19 uc003ybd.2_5_9  chr8:79601608  + GT AT (D1) shift; increase; inframe constitutive FAM164A NP_057094.2 NM_016010.2 family with sequence similarity 164, member A GTNGTN
  20 uc003yil.1_11_12  chr8:99205613  - GT AT (D6) exonization; frameshift alternative NIPAL2 NP_079035.1 NM_024759.1 NIPA-like domain containing 2 dbSNP:rs3735887
  21 uc001ljt.2_45_52  chr10:129216805  + GT GC (D10) type change (GC to GT) constitutive DOCK1 NP_001371.1 NM_001380.3 dedicator of cytokinesis 1 GC to GT
  22 uc001lvx.1_6_7  chr11:2334938  + GT GC (D4) shift; decrease; frameshift alternative TSPAN32 Q96QS1-3 AF176070.1 tetraspanin-32  
  23 uc009ywj.2_1_19  chr11:94225808  - GT AT (D1) shift; increase; inframe alternative MRE11A B3KTC7 AK095388.1 meiotic recombination 11 homolog A (S. cerevisiae) dbSNP:rs496797
  24 uc001qip.3_20_26  chr12:999676  + GT AT (D3) shift; decrease; inframe alternative WNK1 NP_055638.2 NM_014823.2 serine/threonine-protein kinase WNK1 GTNGTN
  25 uc001szm.1_1_16  chr12:81472207  + GT TT (D3) shift; decrease; inframe alternative ACSS3 Q9H6R3 DA156034.1 acyl-CoA synthetase short-chain family member 3 GTNGTN
  26 uc001wxi.1_1_3  chr14:50472312  - GT CT (D7) exonization; novel start constitutive C14orf182 NP_001012724.1 NM_001012706.1 chromosome 14 open reading frame 182 de novo protein
  27 uc010tyr.1_1_8  chr14:105939849  + GT GC (D7) exonization; novel start alternative CRIP2 B7Z6C0 AK300092.1 cysteine-rich protein 2  
  28 uc010ujw.1_1_8  chr15:67841545  + GT TT (D7) exonization; novel start alternative MAP2K5 NP_001193733.1 NM_001206804.1 mitogen-activated protein kinase kinase 5  
  29 uc002ayt.1_10_14  chr15:75114241  + GT GC (D5) exonization; inframe constitutive LMAN1L NP_068591.2 NM_021819.2 lectin, mannose-binding, 1 like  
  30 uc002fck.2_4_9  chr16:72863678  - GT GC (D3) shift; decrease; inframe constitutive ZFHX3 NP_001158238.1 NM_001164766.1 zinc finger homeobox protein 3  
  31 uc010vqa.1_2_3  chr17:650785  - GT AT (D9) intronization; frameshift alternative GEMIN4 B4DZ29 AK302722.1 gem (nuclear organelle) associated protein 4  
  32 uc002htp.2_2_5  chr17:38172107  + GT GC (D1) shift; increase; inframe alternative CSF3 NP_000750.1 NM_000759.3 colony stimulating factor 3 (granulocyte)  
  33 uc002kms.1_1_6  chr18:5406764  - GT GC (D1) shift; increase; inframe alternative EPB41L3 B3KT50 AK094952.1 erythrocyte membrane protein band 4.1-like 3  
  34 uc010dpa.2_1_7  chr18:48346267  - GT GG (D7) exonization; novel start alternative MRO NP_001120648.1 NM_001127176.1 maestro  
  35 uc010zuc.1_1_15  chr20:31619550  + GT GC (D1) shift; increase; inframe alternative BPIFB6 NP_777557.1 NM_174897.2 BPI fold containing family B, member 6 GTNGTN
 
