# Supplementary material for: Human-specific protein isoforms produced by novel splice sites in the human genome after the human-chimpanzee divergence
Source: BMC Bioinformatics. 2012 Nov 13;13:299. doi: 10.1186/1471-2105-13-299 (PMC3538075; doi:10.1186/1471-2105-13-299)
Supplement: Additional file 3 — Examples of the human-specific splice sites. [file 1471-2105-13-299-S3.pdf]

DYNC2LI1 (A1) shift; increase; inframe

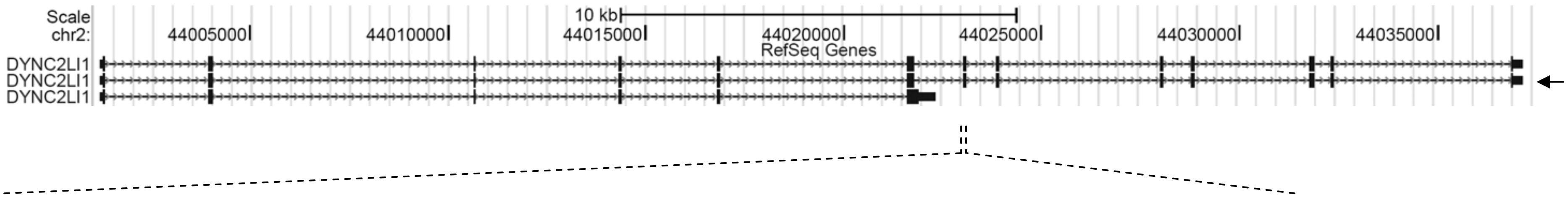


```
Human       attagCAGGATCATGAATTAATTGACCCATTTCCGGTACCTCTGGTCATAATTGGAAGTAAATATGATGTTTTTCAGgtaag
Chimp       ....a.......G.....................................................................
Gorilla     ....a.......G.....................A...............................................
Orangutan   ....a.......G.....................A.................................A.............
Rhesus      t.a.a.......G.....................A.................................A.............
Baboon      t.a.a.......G.....................A.................................A.............
Marmoset    ....a.......G....A................A..............T..C...............A.............
Tarsier     ....a.......G............T........AA................................A.............
Galago      ....a.......G.....................AA................................A.............
Mouse       ....a.....C.GG....................AA.............T..................A....C......g.
Cow         g...a.......G.......C.............AA.T..............C...........C...A..........g..
Dog         g...a.....C.G.......G............TAA............................C...A.............
```

Figure S1. The human-specific novel alternative splice acceptor of exon 7 of the *DYNC2LI1* gene. The top panel shows the exonic structures of the human *DYNC2LI1* transcripts. The novel human isoform is marked by an arrow at the right. The bottom panel shows a multiple sequence alignment of the orthologous genomic segments from the selected mammalian species. The coding region of the novel human isoform is in uppercase. The novel human splice acceptor (ag) is highlighted in black. The ancestral splice acceptors (AG) and donors (gt) are highlighted in grey. Note the consecutive acceptor sequence TAGCAG, which is known as the NAGNAG acceptor. Dots indicate that the sequences are the same as the human sequence.

ESRRA (A1) shift; increase; inframe

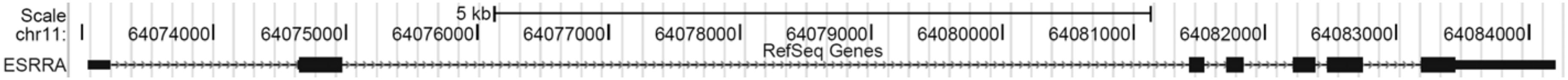


```
Human      cacagCAGCCCCAGTGAATGCACTGGTGTCTCATCTGCTGGTGGTTGAGCCTGAGAAGCTCTATGCCATGCCTGACCCCGCAGGCCCTGATGGGCACCTC
Chimp      ...c................................................................................................
Gorilla    ...c................................................................................................
Orangutan  ...c...........A..............................................................T.....................
Rhesus     ...c....................................................................C........G..................
Baboon     ...c....................................................................C........G..................
Marmoset   tg.ca.....................................C..G...........A....................T..G..................
Lemur      ...t........G....................C.....A........A.....A.....A...........C.....A..G...........A......
Mouse      ...t.T............C..T........G.................A...........G..C..............A...A....C.....A......
Cow        ...ta.......G.....C.....C.....C..C..............A...........G...........C.....A..G...........A......
Dog        ...t.....T..T..A.................C...........A.....C..........................A..G........C..A......

Human      CCAGCCGTGGCTACCCTCTGTGACCTCTTTGACCGAGAGATTGTGGTCACCATCAGCTGGGCCAAGAGCATCCCAGgtaaa
Chimp      .................................................................................
Gorilla    .........................................C.......................................
Orangutan  .........................................C.......................................
Rhesus     .................................................................................
Baboon     .................................................................................
Marmoset   .................................................................................
Lemur      .....T...................................C.......................................
Mouse      ..C..T.....C..T...........T.....T........A.......................................
Cow        .....T.....C.............................C.....................................g.
Dog        .....T..............................................................T............
```

Figure S2. The human-specific novel alternative splice acceptor of the last exon of the *ESRRA* gene. The top panel shows the exonic structure of the human *ESRRA* transcript. The bottom panel shows a multiple sequence alignment of the orthologous genomic segments from the selected mammalian species. The coding region of the novel human isoform is in uppercase. The novel human splice acceptor (ag) is highlighted in black. The ancestral splice acceptors (AG) and donors (gt) are highlighted in grey. Note the consecutive acceptor sequence CAGCAG, which is known as the NAGNAG acceptor. Dots indicate that the sequences are the same as the human sequence.

SMARCAD1 (A1) shift; increase; inframe

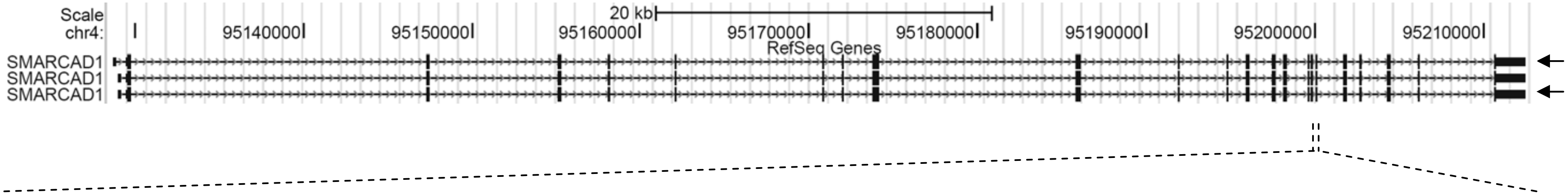


```
Human      ttgagTCACAGAAAAAAACACAGAAATGTGCAATGTCATGATGCAGTTGAGGAAAATGGCCAATCATCCTTTATTACATCGCCAATATTACACAGCTGAA
Chimp      ...g................................................................................................
Orangutan  ...g........G.......................................................................................
Rhesus     ...g........G.......................................................................................
Baboon     ...g........G.......................................................................................
Marmoset   ...g........G.......................................................................................
Tarsier    ...ta.............T................................A..........................C.....................
Lemur      ...ga.........................T...............C....A................................................
Mouse      ...ga...................G....................A.....A..............C......C....C.....G.........C....G
Cow        ...ga.............T..........................A.....A................................................
Dog        .g..a............G.................................A................................................

Human      AAACTCAAGGAAATGTCTCAGCTTATGCTAAAGgtaag
Chimp      ......................................
Orangutan  ......................................
Rhesus     ......................................
Baboon     ......................................
Marmoset   ......................................
Tarsier    ..G..................................t
Lemur      ......................................
Mouse      .....G.....G...........A.....G........
Cow        ......................................
Dog        ..G...................................
```

Figure S3. The human-specific novel alternative splice acceptor of exon 19 of the *SMARCAD1* gene. The top panel shows the exonic structures of the human *SMARCAD1* transcripts. The isoforms spliced using the novel acceptor are marked by arrows at the right. The bottom panel shows a multiple sequence alignment of the orthologous genomic segments from the selected mammalian species. The coding region of the novel human isoform is in uppercase. The derived exon is 6-bp longer than the ancestral form. The novel human splice acceptor (ag) is highlighted in black. The ancestral splice acceptors (AG) and donors (gt) are highlighted in grey. Dots indicate that the sequences are the same as the human sequence.

PNPLA5 (A3) shift; decrease; inframe

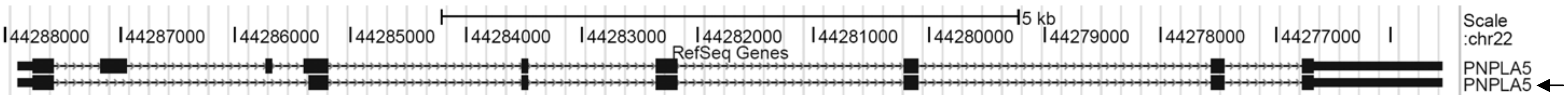


```
Human      tgcagcgctacatcgatggggctctgagcaacaacttgccctttgcagACTGCCCCTCCACCATCACGGTGTCGCCCTTCCATGGGACAGTGGACATCTG
Chimp      ..............................................g..........................A..........................
Gorilla    ..............................................g.....T............................G..................
Orangutan  ................c...t.........................g..............................C................T.....
Rhesus     ..............................................g..........................C..........................
Baboon     ..............................................g..........................C..........................
Marmoset   .a.....a......................................g..........TT........C.....A........C..............T..
Galago     c...............c.......................g.....g...................T.....A..T..............A........
Mouse      .c.......t...t..c..................c.......ct.c..T......A.......T..C..C..T..T...A.C.................
Dog        ................................c..c.t......t.g....CG........A.....C.....C..T...........G...........

Human      CCCCCAGAGCACCTCCCCCAACCTGCATGAGCTGAACGTCTTCAACTTCAGCTTCCAAATCTCCACTGAGAACTTCTTCCTGGGGCTCATATGTCTCATA
Chimp      ...............................................C....................................................
Gorilla    ...............................................C....................................................
Orangutan  ......................................C........C.....................................T....G.........
Rhesus     ....................................T.C.......GC..................CA.................T..G..A.C......
Baboon     ....................................T.C.......GC..................CA.................T.....A.C......
Marmoset   ...............TG..............T....T.C......TGC..................C.................AT.......C..TG.G
Galago     ...............A....G...............T.C......TGC..................CAT................T....T.CC......
Mouse      T.......A..TA..A.AT.GT..TTT......A.CT.C......TGC...T.....G.....T..CAG.........TAG........A..C.G.GT.C
Dog        ...............GG.....A.A...........T.C......TGC...TC....G........CA.................T...GT..C......

Human      CCCCCCAGCCTCGAGGTAGgtctg
Chimp      ........................
Gorilla    ........................
Orangutan  ........G...............
Rhesus     ........................
Baboon     ........................
Marmoset   .......A..CT......C...ca
Galago     ........T.CT............
Mouse      .......AG.CT..........ac
Dog        T......A.TCT........c...
```

Figure S4. The human-specific novel alternative exon of the *PNPLA5* gene. The top panel shows the exonic structures of the human *PNPLA5* transcripts. The human-specific isoform is indicated by an arrow at the right. The bottom panel shows a multiple sequence alignment of the orthologous genomic segments from the selected mammalian species. The coding region is in uppercase. The human-specific splice acceptor (ag) is highlighted in black. The ancestral splice acceptors (ag) and donors (gt) are highlighted in grey. The derived exon is 48-bp shorter than the ancestral form. Dots indicate that the sequences are the same as the human sequence.

IZUMO4 (A5) exonization; inframe

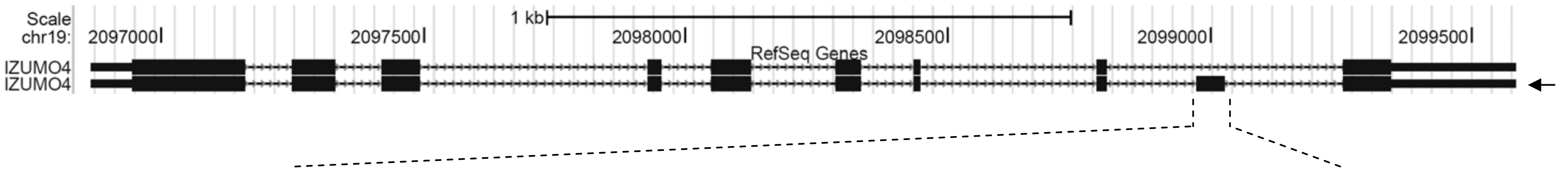


```
Human      aacagACCACGCTCCTCTGCCTTCTCCTGGCCTGGGACACACAGAGCCACCCCGGCCTTgtgag
Chimp      ....a....................T.................C.........A..........
Gorilla    ....a................................................A..........
Orangutan  ....a............CT..................................A..........
Rhesus     ....a..T.........CT..................................A..........
Baboon     ....a..T.........CT..................................A..........
```

Figure S5. The human-specific novel alternative exon of the *IZUMO4* gene. The top panel shows the exonic structures of the human *IZUMO4* transcripts. The human-specific isoform is indicated by an arrow at the right. The bottom panel shows a multiple sequence alignment of the orthologous genomic segments from the selected mammalian species. The coding region is in uppercase. The human-specific splice acceptor (ag) is highlighted in black. The cryptic splice donor (gt) is highlighted in grey. Dots indicate that the sequences are the same as the human sequence.

LMAN1L (D5) exonization; inframe

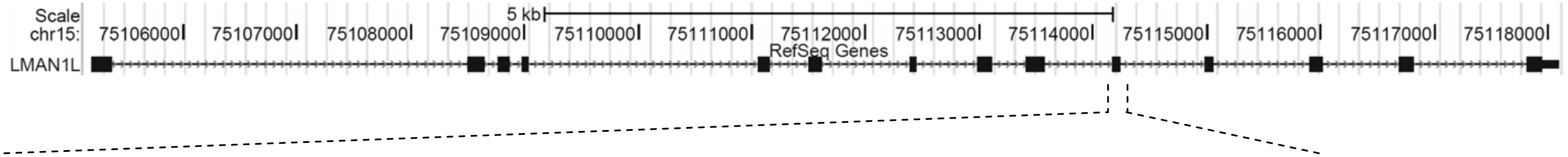


```
Human       cccagGCCCTGGATGCTTCCTGCCAGATTCCATCCACCCCAGGGAGGGGTGGCCACCTCTCCATGTCACTCAATAAGgtagg
Chimp       ..............................................................................c...
Gorilla     ..............................................................................c...
Orangutan   ........T....CT...............................................................c...
Lemur       tt.........C.CC.C.........G.......AG......A..AAC...............G........GCG...c...
Mouse       tt...........CC.C..T...TT....G..CT.....TCA.A.AT.TCA..T.........G...CT..TGCC..actt.
Cow         g........A...CT.CC........GC....C.......T.A...A......TC........G......GGGC...a.gcc
Dog         t........A.A.CT.C.........G...T.C.......G.A......C..T...T......G.C.T....GCG..ac..a
```

Figure S6. The human-specific novel constitutive exon 10 of the *LMAN1L* gene. The top panel shows the exonic structure of the human *LMAN1L* transcript. The bottom panel shows a multiple sequence alignment of the orthologous genomic segments from the selected mammalian species. The coding region is in uppercase. The human-specific splice donor (gt) is highlighted in black. The cryptic splice acceptor is highlighted in grey. Dots indicate that the sequences are the same as the human sequence.

CLK1 (D7) exonization; novel start

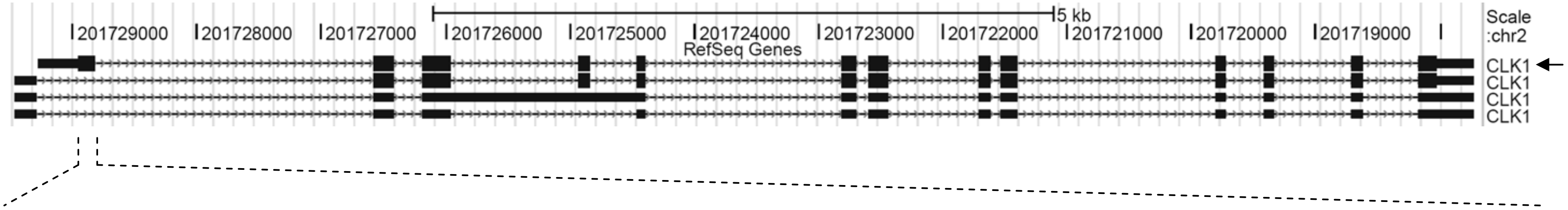


```
Human      gtaagATGGCGGCTGGGCGGAGGCCGGCTTCGGCCCTGTGGCCGGAAAGGCGAGGCTCCCCGTTGAGGG-GGGATTTGCTGGGGTTCCAGAATGTGCGTG
Chimp      .....................................................................-...G..........................
Gorilla    .....................................................................-..TG.......................A..
Orangutan  .........................C..................................T....G...T...G..........................
Rhesus     ............................C.T...A.........................T....G...T...G..............G........T..
Baboon     ............................C.T...A.........................T....G...T...G..............G........T..
Marmoset   ............................C.T..T.AC-...G..................T....G...T...G....T....A........AA.-----

Human      AGCCAAGCAGCTGTGGGGAAACGTTGTCTGGAgtaag
Chimp      .....C...........................c...
Gorilla    .....C...........................c...
Orangutan  ...TGC.......A...................c...
Rhesus     .AG..C...............T..C........c...
Baboon     .AG..C...............T..C........c...
Marmoset   -----C..C............---......CG.c...
```

Figure S7. The novel first exon of the human *CLK1* gene. The top panel shows the exonic structures of the human *CLK1* transcripts. The human-specific isoform with a novel first exon encoding an alternative N-terminus is marked by an arrow at the right. The bottom panel shows a multiple sequence alignment of the human-specific exon and its orthologous regions of primate species. The coding region is in uppercase. The human-specific splice donor and start codon are highlighted in black. Dots indicate that the sequences are the same as the human sequence.

PAX3 (D9) intronization; frameshift

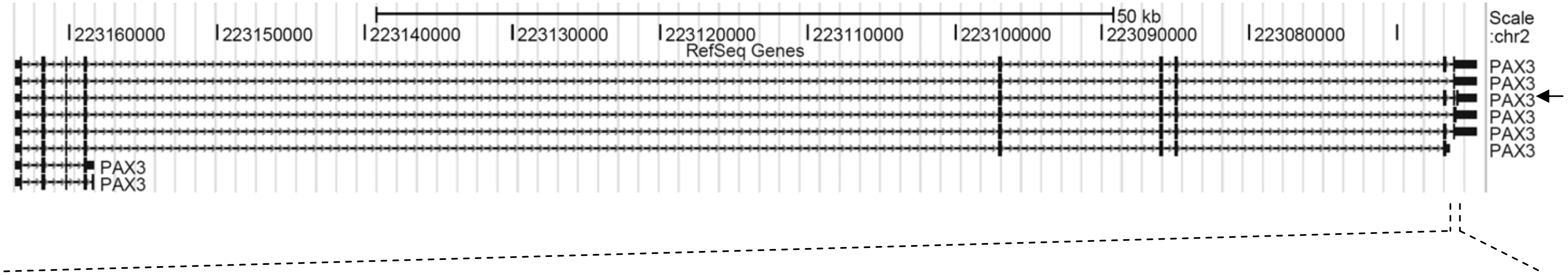


```
Human        tacagGTGCCTTTCATTATCTCAAGCCAGATATCGCgtaagtgaa (161 nt) aatagTTGGTTTCAAATCCTTTTGAACACGTTCGACAAAAGCAGT
Chimp        ....................................a........          .............................................
Gorilla      ....................................a........          .............................................
Orangutan    ....................................a........          .................T...........A...............
Rhesus       ....................................a........          .................T-.........T...T........TG..
Baboon       ....................................a........          .................T-.........T...T........TG..
Marmoset     ....................................a........          .g...............T..........A...G.C.......C..
Tarsier      ....................................a........          ....a..T.....C...T..........T...G............
Lemur        .c..................................a....a...          .g...........C...T.A.......TT...G..........A.
Galago       ....................................a....a...          .g..............GTTA.......TT...G..........A.
Mouse        .................................T..a........          ------------------------------------....A..--
Cow          ....................................a........          .g...............T.........GT..CGA...........

Human        GGAGAAGAGGAAGACCTGGAGCAATAA
Chimp        ...........................
Gorilla      ...........................
Orangutan    ...........................
Rhesus       ........A..................
Baboon       ........A..................
Marmoset     ...........................
Tarsier      .A..---.C.....T......GG....
Lemur        .A..............C..........
Galago       .A..C.........G.....A...G.C
Mouse        ----------------------..C..
Cow          ......A.......T.....A......
```

Figure S8. Human-specific intronization in the last exon of the *PAX3* gene. The top panel shows the exonic structures of the human *PAX3* transcripts with the human-specific isoform being marked by an arrow at the right. The bottom panel shows a multiple sequence alignment of the orthologous genomic segments from the selected mammalian species. The coding region of the human-specific isoform is in uppercase. The underlined part of the last exon is intronized owing to the novel splice donor (gt), which is highlighted in black. The ancestral stop codon (taa) and the novel stop codon (TAA) are highlighted in grey and black, respectively. The conserved mammalian splice acceptors (ag) of the last exon and the human cryptic acceptor (ag) are highlighted in grey. Dots indicate that the sequences are the same as the human sequence.

DOCK1 **(D10) type change (GC to GT)**

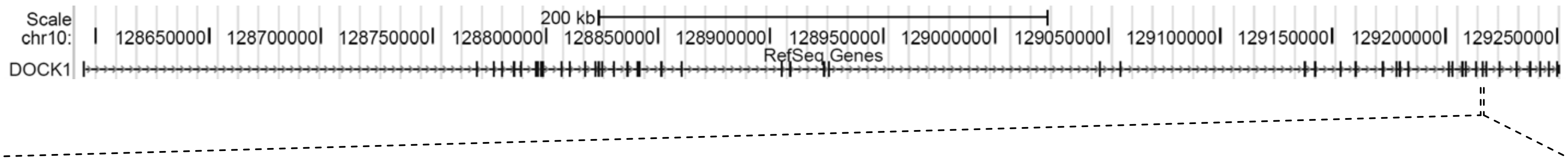


```
Human      tgcagGTGGAAATCAGCCCCCTGGAGAATGCCATTGAGACCATGCAGCTGACGAACGACAAGATCAACAGCATGGTGCAGCAGCACCTGGATGACCCCAG
Chimp      ..................................C.................................................................
Orangutan  ...............................T..C.........................................A.......................
Rhesus     ............................C.....C.................................................................
Baboon     ............................C.....C.................................................................
Marmoset   .............T..............C.....C.................A..T...................................C........
Lemur      ..........G.................C.....C.................A............G.........................C........
Galago     ..t.......GG................C.....A...........A.....C..T.........G..................................
Mouse      .......A..G........A..............C.....A......T....C..T..........G.........C....................GG.
Cow        c.........G.................C.....C.................C.......................C...........A..C........
Dog        ..........G.......................C.................C...........G..........................C........

Human      CCTGCCCATCAACCCGCTCTCCATGCTCCTGAACGGCATCGTGGACCCAGCTGTCATGGGGGGCTTCGCAAACTACGAAAAGgtacg
Chimp      .......................................T...........................A...............c...
Orangutan  .................................T.................................A...............c...
Rhesus     ...............A...................................................A...............c...
Baboon     ...................................................................A...............c...
Marmoset   ...................................................................A...............c.t.
Lemur      ...C..TG.......C...........G..T.....A...........T..C..T...........T................ct..
Galago     ...............C..G...........C.................C...........A..A...........T.......ct..
Mouse      ......T........C..G..............T.....T.....T..T...........T..T..T..C..T..T..G....c...
Cow        ...C..........................A.................C..C........A..T..T..C..T.....G....c...
Dog        ...C...........C..............C..T..............C..C........A.....T.....T..T...........
```

Figure S9. The splice donor type change (GC to GT) of the human *DOCK1* gene exon 45. The top panel shows the exonic structure of the human *DOCK1* transcript. The bottom panel shows a multiple sequence alignment of the orthologous genomic segments from the selected mammalian species. The coding region is in uppercase. The human splice donor (gt) is highlighted in black. The ancestral splice acceptors (ag) and donors (gc) are highlighted in grey. Dots indicate that the sequences are the same as the human sequence.
