# Supplementary material for: Human-specific protein isoforms produced by novel splice sites in the human genome after the human-chimpanzee divergence
Source: BMC Bioinformatics. 2012 Nov 13;13:299. doi: 10.1186/1471-2105-13-299 (PMC3538075; doi:10.1186/1471-2105-13-299)
Supplement: Additional file 4 — Alignments of the exon and splice acceptor sequences. [file 1471-2105-13-299-S4.html]

Table S3. Alignments of the exon and splice acceptor sequences

# Table S3. Alignments of the exon and splice acceptor sequences

---

## 1. uc001aoe.1\_4\_5

**Summary**  

|  |  |  |  |  |  |  |  |  |  |  |  |  |  |  |  |  |  |  |  |  |  |  |  |  |  |
| --- | --- | --- | --- | --- | --- | --- | --- | --- | --- | --- | --- | --- | --- | --- | --- | --- | --- | --- | --- | --- | --- | --- | --- | --- | --- |
| No Exon ID Position (hg19) Dir Human acceptor Chimp acceptor Category Usage Gene symbol Protein accession mRNA accession Gene title Note|  |  |  |  |  |  |  |  |  |  |  |  |  | | --- | --- | --- | --- | --- | --- | --- | --- | --- | --- | --- | --- | --- | | 1 uc001aoe.1\_4\_5 chr1:6692430 + AG TG (A1) shift; increase; inframe alternative THAP3 NP\_612359.2 NM\_138350.3 THAP domain-containing protein 3  | | | | | | | | | | | | | | | | | | | | | | | | | |

**Orthologs**  

|  |  |  |  |  |  |  |  |  |  |  |  |  |  |  |  |  |  |  |  |  |  |  |  |  |  |  |  |  |  |  |  |  |  |  |  |  |  |  |  |  |  |  |  |  |  |  |  |  |  |  |  |  |  |  |  |  |  |  |  |  |  |  |  |  |  |  |  |  |  |  |  |  |  |  |  |  |  |  |  |  |  |  |  |  |  |  |  |
| --- | --- | --- | --- | --- | --- | --- | --- | --- | --- | --- | --- | --- | --- | --- | --- | --- | --- | --- | --- | --- | --- | --- | --- | --- | --- | --- | --- | --- | --- | --- | --- | --- | --- | --- | --- | --- | --- | --- | --- | --- | --- | --- | --- | --- | --- | --- | --- | --- | --- | --- | --- | --- | --- | --- | --- | --- | --- | --- | --- | --- | --- | --- | --- | --- | --- | --- | --- | --- | --- | --- | --- | --- | --- | --- | --- | --- | --- | --- | --- | --- | --- | --- | --- | --- | --- | --- | --- |
| Species Assembly Chromosome Exon start Exon end Dir Acceptor Exon sequence|  |  |  |  |  |  |  |  |  |  |  |  |  |  |  |  |  |  |  |  |  |  |  |  |  |  |  |  |  |  |  |  |  |  |  |  |  |  |  |  |  |  |  |  |  |  |  |  |  |  |  |  |  |  |  |  |  |  |  |  |  |  |  |  |  |  |  |  |  |  |  |  |  |  |  |  |  |  |  |  | | --- | --- | --- | --- | --- | --- | --- | --- | --- | --- | --- | --- | --- | --- | --- | --- | --- | --- | --- | --- | --- | --- | --- | --- | --- | --- | --- | --- | --- | --- | --- | --- | --- | --- | --- | --- | --- | --- | --- | --- | --- | --- | --- | --- | --- | --- | --- | --- | --- | --- | --- | --- | --- | --- | --- | --- | --- | --- | --- | --- | --- | --- | --- | --- | --- | --- | --- | --- | --- | --- | --- | --- | --- | --- | --- | --- | --- | --- | --- | --- | | Human hg19 chr1 6692430 6692555 + **AG** | ACTTCACCCTGCCGTTCCCAGGTCCTCCCTGAGGCGGGGGCCGGAGAGGACAGTCCTGGGAGAAACATGGACACTGCACTTGAAGAGCTTCAGTTGCCCCCAAATGCCGAAGGCCACGTAAAACAG|  |  |  |  |  |  |  |  |  |  |  |  |  |  |  |  |  |  |  |  |  |  |  |  |  |  |  |  |  |  |  |  |  |  |  |  |  |  |  |  |  |  |  |  |  |  |  |  |  |  |  |  |  |  |  |  |  |  |  |  |  |  |  |  |  |  |  |  |  |  |  |  | | --- | --- | --- | --- | --- | --- | --- | --- | --- | --- | --- | --- | --- | --- | --- | --- | --- | --- | --- | --- | --- | --- | --- | --- | --- | --- | --- | --- | --- | --- | --- | --- | --- | --- | --- | --- | --- | --- | --- | --- | --- | --- | --- | --- | --- | --- | --- | --- | --- | --- | --- | --- | --- | --- | --- | --- | --- | --- | --- | --- | --- | --- | --- | --- | --- | --- | --- | --- | --- | --- | --- | --- | | Chimp panTro2 chr1 6691722 6691847 + **TG** | ACTTCACCCTGCCGTTCCCAGGTCCTCCCTGAGGCGGGGGCCGGAGAGGACAGTCCTGGGAGAAACATGGACACTGCACTTGAAGAGCTTCAGTTGCCCCCAAATGCCGAAGGCCACGTAAAACAG|  |  |  |  |  |  |  |  |  |  |  |  |  |  |  |  |  |  |  |  |  |  |  |  |  |  |  |  |  |  |  |  |  |  |  |  |  |  |  |  |  |  |  |  |  |  |  |  |  |  |  |  |  |  |  |  |  |  |  |  |  |  |  |  | | --- | --- | --- | --- | --- | --- | --- | --- | --- | --- | --- | --- | --- | --- | --- | --- | --- | --- | --- | --- | --- | --- | --- | --- | --- | --- | --- | --- | --- | --- | --- | --- | --- | --- | --- | --- | --- | --- | --- | --- | --- | --- | --- | --- | --- | --- | --- | --- | --- | --- | --- | --- | --- | --- | --- | --- | --- | --- | --- | --- | --- | --- | --- | --- | | Gorilla gorGor1 Supercontig\_0039685 7380 7505 - **TG** | ACTTCACCCTGCCGTTCCCAGGTCCTCCCTGAGGCGGGGGCTGGAGAGGACAGTCCTGGGAGAAACATGGACACTGCACTTGAAGAGCTTCAGTTGCCCCCAAATGCCGAAGGCCACGTAAAACAG|  |  |  |  |  |  |  |  |  |  |  |  |  |  |  |  |  |  |  |  |  |  |  |  |  |  |  |  |  |  |  |  |  |  |  |  |  |  |  |  |  |  |  |  |  |  |  |  |  |  |  |  |  |  |  |  | | --- | --- | --- | --- | --- | --- | --- | --- | --- | --- | --- | --- | --- | --- | --- | --- | --- | --- | --- | --- | --- | --- | --- | --- | --- | --- | --- | --- | --- | --- | --- | --- | --- | --- | --- | --- | --- | --- | --- | --- | --- | --- | --- | --- | --- | --- | --- | --- | --- | --- | --- | --- | --- | --- | --- | --- | | Orangutan ponAbe2 chr1 223877427 223877552 - **TG** | ACTCCACCCTGCCGTTCCCAGGTCCTCCCTGAGGGGGGGGCCGGAGAGGACAGTCCTGGGAGAAACATGGACACTGCACTTGAAGAGCTTCAGTTGCCCCCAAATGCCGAAGACCCCGTAAAACAG|  |  |  |  |  |  |  |  |  |  |  |  |  |  |  |  |  |  |  |  |  |  |  |  |  |  |  |  |  |  |  |  |  |  |  |  |  |  |  |  |  |  |  |  |  |  |  |  | | --- | --- | --- | --- | --- | --- | --- | --- | --- | --- | --- | --- | --- | --- | --- | --- | --- | --- | --- | --- | --- | --- | --- | --- | --- | --- | --- | --- | --- | --- | --- | --- | --- | --- | --- | --- | --- | --- | --- | --- | --- | --- | --- | --- | --- | --- | --- | --- | | Rhesus rheMac2 chr1 9652285 9652410 + **TG** | ACTCCACGCTGCCGTTCCCAGGGCCTCCCTGAGGGGGGGGCCGGAGAGGACAGCCCTGGGAGAAAGATGGACACTGCGCTTGAAGAGCTTCAGTTGCCCCCAAATGCCGAAGGCCCCGTAAAACAG|  |  |  |  |  |  |  |  |  |  |  |  |  |  |  |  |  |  |  |  |  |  |  |  |  |  |  |  |  |  |  |  |  |  |  |  |  |  |  |  | | --- | --- | --- | --- | --- | --- | --- | --- | --- | --- | --- | --- | --- | --- | --- | --- | --- | --- | --- | --- | --- | --- | --- | --- | --- | --- | --- | --- | --- | --- | --- | --- | --- | --- | --- | --- | --- | --- | --- | --- | | Baboon papHam1 scaffold9211 13801 13926 - **TG** | ACTCCACGCTGCCGTTCCCAGGTCCTCCCTGAGGCGGGGGCCGGAGAGGACAGCCCTGGGAGAAAGATGGACACTGCGCTTGAAGAGCTTCAGTTGCCCCCAAATGCCGAAGGCCCCGTAAAACAG|  |  |  |  |  |  |  |  |  |  |  |  |  |  |  |  |  |  |  |  |  |  |  |  |  |  |  |  |  |  |  |  | | --- | --- | --- | --- | --- | --- | --- | --- | --- | --- | --- | --- | --- | --- | --- | --- | --- | --- | --- | --- | --- | --- | --- | --- | --- | --- | --- | --- | --- | --- | --- | --- | | Marmoset calJac1 Contig1199 553569 553694 + **TG** | ACTTCACCCTGCTGTTCCCAGGTCCTCCCTGAGGCGGGGGCCGGAGAGAATGGCCCTGGGAGAAACATGAACACTGCGCATGAGGAGCTTCAGTTGCCCCCAGATGCTGCAGGACCCGTAAAACAG|  |  |  |  |  |  |  |  |  |  |  |  |  |  |  |  |  |  |  |  |  |  |  |  | | --- | --- | --- | --- | --- | --- | --- | --- | --- | --- | --- | --- | --- | --- | --- | --- | --- | --- | --- | --- | --- | --- | --- | --- | | Lemur micMur1 scaffold\_4729 49593 49718 + **TG** | ATTGCACCCTGCTGTCCCCAGGTCCTCCCTGAGGCAGGGGCTGAGGAGCACAGCCCCGGGAGAAACATGGACACTGCACTCGAAGAGCTTCAGCTGCCCCCGAATGCTGAAGGCCCCACCAAACAG|  |  |  |  |  |  |  |  |  |  |  |  |  |  |  |  | | --- | --- | --- | --- | --- | --- | --- | --- | --- | --- | --- | --- | --- | --- | --- | --- | | Mouse mm9 chr4 151357744 151357865 - **TG** | ACTCCGCCTGCTGTTCCCAGGTCTGCCCTGAGGTGGGGGCTGGTGGGGACAGCTCAGGGAGGAACATGGACACCACACTGGAAGAACTTCAGCCTCCAACCCCGGAAGGCCCCGTGCAGCAG|  |  |  |  |  |  |  |  | | --- | --- | --- | --- | --- | --- | --- | --- | | Cow bosTau4 chr16 43984955 43985079 - **TG** | GCTCCGCCCTGTCTTTACAGGTCCTCCCTGAGACGGGCTCCGGGGAGTGTGGCCTGGGGAGGAAGATGGATACGACAGTTGAAGTGCTGCAGCTGCCCCCCGAGGTTGGAGGCCTAGGAGCACAG | | | | | | | | | | | | | | | | | | | | | | | | | | | | | | | | | | | | | | | | | | | | | | | | | | | | | | | | | | | | | | | | | | | | | | | | | | | | | |

**Alignment** (splice site sequences are in lowercase)  

```
Human      agACTTCACCCTGCCGTTCCCAGGTCCTCCCTGAGGCGGGGGCCGGAGAGGACAGTCCTGGGAGAAACATGGACACTGCA
Chimp      t...............................................................................
Gorilla    t..........................................T....................................
Orangutan  t....C..............................G...........................................
Rhesus     t....C...G..............G...........G..................C...........G...........G
Baboon     t....C...G.............................................C...........G...........G
Marmoset   t.............T...................................A.TG.C...............A.......G
Lemur      t..T.G........T..C...................A.....T.AG...C....C..C.....................
Mouse      t....C.G.-....T...........TG........T......T..T.G......CT.A.....G...........CA..
Cow        t.G..C.G.....T.-..TA...............A....CT....G...TGTG.C.TG.....G..G.....T..GA..

Human      CTTGAAGAGCTTCAGTTGCCCCCAAATGCCGAAGGCCACGTAAAACAGgt
Chimp      ..................................................
Gorilla    ..................................................
Orangutan  ..................................A..C............
Rhesus     .....................................C............
Baboon     .....................................C............
Marmoset   .A...G..................G....T.C...A.C............
Lemur      ..C............C.......G.....T.......C.ACC........
Mouse      ..G.....A.....---...T....CCC.G.......C...GC.G.....
Cow        G......T...G...C.......CG.G.TT.G.....TA.G.GC......
```

---

## 2. uc001bfa.2\_3\_15

**Summary**  

|  |  |  |  |  |  |  |  |  |  |  |  |  |  |  |  |  |  |  |  |  |  |  |  |  |  |
| --- | --- | --- | --- | --- | --- | --- | --- | --- | --- | --- | --- | --- | --- | --- | --- | --- | --- | --- | --- | --- | --- | --- | --- | --- | --- |
| No Exon ID Position (hg19) Dir Human acceptor Chimp acceptor Category Usage Gene symbol Protein accession mRNA accession Gene title Note|  |  |  |  |  |  |  |  |  |  |  |  |  | | --- | --- | --- | --- | --- | --- | --- | --- | --- | --- | --- | --- | --- | | 2 uc001bfa.2\_3\_15 chr1:22047656 - AG AA (A3) shift; decrease; inframe alternative USP48 Q86UV5-5 AK021830.1 ubiquitin specific protease 48  | | | | | | | | | | | | | | | | | | | | | | | | | |

**Orthologs**  

|  |  |  |  |  |  |  |  |  |  |  |  |  |  |  |  |  |  |  |  |  |  |  |  |  |  |  |  |  |  |  |  |  |  |  |  |  |  |  |  |  |  |  |  |  |  |  |  |  |  |  |  |  |  |  |  |  |  |  |  |  |  |  |  |  |  |  |  |  |  |  |  |  |  |  |  |  |  |  |  |  |  |  |  |  |  |  |  |  |  |  |  |  |  |  |  |
| --- | --- | --- | --- | --- | --- | --- | --- | --- | --- | --- | --- | --- | --- | --- | --- | --- | --- | --- | --- | --- | --- | --- | --- | --- | --- | --- | --- | --- | --- | --- | --- | --- | --- | --- | --- | --- | --- | --- | --- | --- | --- | --- | --- | --- | --- | --- | --- | --- | --- | --- | --- | --- | --- | --- | --- | --- | --- | --- | --- | --- | --- | --- | --- | --- | --- | --- | --- | --- | --- | --- | --- | --- | --- | --- | --- | --- | --- | --- | --- | --- | --- | --- | --- | --- | --- | --- | --- | --- | --- | --- | --- | --- | --- | --- | --- |
| Species Assembly Chromosome Exon start Exon end Dir Acceptor Exon sequence|  |  |  |  |  |  |  |  |  |  |  |  |  |  |  |  |  |  |  |  |  |  |  |  |  |  |  |  |  |  |  |  |  |  |  |  |  |  |  |  |  |  |  |  |  |  |  |  |  |  |  |  |  |  |  |  |  |  |  |  |  |  |  |  |  |  |  |  |  |  |  |  |  |  |  |  |  |  |  |  |  |  |  |  |  |  |  |  | | --- | --- | --- | --- | --- | --- | --- | --- | --- | --- | --- | --- | --- | --- | --- | --- | --- | --- | --- | --- | --- | --- | --- | --- | --- | --- | --- | --- | --- | --- | --- | --- | --- | --- | --- | --- | --- | --- | --- | --- | --- | --- | --- | --- | --- | --- | --- | --- | --- | --- | --- | --- | --- | --- | --- | --- | --- | --- | --- | --- | --- | --- | --- | --- | --- | --- | --- | --- | --- | --- | --- | --- | --- | --- | --- | --- | --- | --- | --- | --- | --- | --- | --- | --- | --- | --- | --- | --- | | Human hg19 chr1 22047529 22047656 - **AG** | CGATGGATTTTGGGTGGGGAAGTCCTCCTTGCGGAGTTGGCGCCAGCTAGCTCTTGAACAGCTGGATGAGCAAGATGGTGATGCAGAACAAAGCAACGGAAAGATGAACGGTAGCACCTTAAATAAAG|  |  |  |  |  |  |  |  |  |  |  |  |  |  |  |  |  |  |  |  |  |  |  |  |  |  |  |  |  |  |  |  |  |  |  |  |  |  |  |  |  |  |  |  |  |  |  |  |  |  |  |  |  |  |  |  |  |  |  |  |  |  |  |  |  |  |  |  |  |  |  |  |  |  |  |  |  |  |  |  | | --- | --- | --- | --- | --- | --- | --- | --- | --- | --- | --- | --- | --- | --- | --- | --- | --- | --- | --- | --- | --- | --- | --- | --- | --- | --- | --- | --- | --- | --- | --- | --- | --- | --- | --- | --- | --- | --- | --- | --- | --- | --- | --- | --- | --- | --- | --- | --- | --- | --- | --- | --- | --- | --- | --- | --- | --- | --- | --- | --- | --- | --- | --- | --- | --- | --- | --- | --- | --- | --- | --- | --- | --- | --- | --- | --- | --- | --- | --- | --- | | Chimp panTro2 chr1 21861000 21861127 - **AA** | CGATGGATTTTGGGTGGGGAAGTCTTCCTTGCGGAGTTGGCGCCAGCTAGCTCTTGAACAGCTGGATGAGCAAGATGGTGATGCAGAACAAAGCAACGGAAAGATGAACGGTAGCACCTTAAATAAAG|  |  |  |  |  |  |  |  |  |  |  |  |  |  |  |  |  |  |  |  |  |  |  |  |  |  |  |  |  |  |  |  |  |  |  |  |  |  |  |  |  |  |  |  |  |  |  |  |  |  |  |  |  |  |  |  |  |  |  |  |  |  |  |  |  |  |  |  |  |  |  |  | | --- | --- | --- | --- | --- | --- | --- | --- | --- | --- | --- | --- | --- | --- | --- | --- | --- | --- | --- | --- | --- | --- | --- | --- | --- | --- | --- | --- | --- | --- | --- | --- | --- | --- | --- | --- | --- | --- | --- | --- | --- | --- | --- | --- | --- | --- | --- | --- | --- | --- | --- | --- | --- | --- | --- | --- | --- | --- | --- | --- | --- | --- | --- | --- | --- | --- | --- | --- | --- | --- | --- | --- | | Gorilla gorGor1 Supercontig\_0390476 8198 8325 - **AA** | CGATGGATTTTGGGTGGGGAAGTCCTCCTTGCGGAGTTGGCGCCAGCTAGCTCTTGAACAGCTGGATGAGCAAGATGGTGATGCAGAACAAAGCAACGGAAAGATGAACGGTAGCACCTTAAATAAAG|  |  |  |  |  |  |  |  |  |  |  |  |  |  |  |  |  |  |  |  |  |  |  |  |  |  |  |  |  |  |  |  |  |  |  |  |  |  |  |  |  |  |  |  |  |  |  |  |  |  |  |  |  |  |  |  |  |  |  |  |  |  |  |  | | --- | --- | --- | --- | --- | --- | --- | --- | --- | --- | --- | --- | --- | --- | --- | --- | --- | --- | --- | --- | --- | --- | --- | --- | --- | --- | --- | --- | --- | --- | --- | --- | --- | --- | --- | --- | --- | --- | --- | --- | --- | --- | --- | --- | --- | --- | --- | --- | --- | --- | --- | --- | --- | --- | --- | --- | --- | --- | --- | --- | --- | --- | --- | --- | | Orangutan ponAbe2 chr1 208731124 208731251 + **AA** | CGATGGATTTTGGGTGGGGAAGTCCTCCTTGCGGAGTTGGCGCCAGCTAGCTCTTGAACAGCTGGATGAGCAAGATGGTGATGCAGAACAAAGCAACGGAAAGATGAACGGTAGCACCTTAAATAAAG|  |  |  |  |  |  |  |  |  |  |  |  |  |  |  |  |  |  |  |  |  |  |  |  |  |  |  |  |  |  |  |  |  |  |  |  |  |  |  |  |  |  |  |  |  |  |  |  |  |  |  |  |  |  |  |  | | --- | --- | --- | --- | --- | --- | --- | --- | --- | --- | --- | --- | --- | --- | --- | --- | --- | --- | --- | --- | --- | --- | --- | --- | --- | --- | --- | --- | --- | --- | --- | --- | --- | --- | --- | --- | --- | --- | --- | --- | --- | --- | --- | --- | --- | --- | --- | --- | --- | --- | --- | --- | --- | --- | --- | --- | | Baboon papHam1 scaffold641 49150 49277 + **AA** | TGATGGATTTTGGGTGGGGAAGTCCTCCTTGAGGAGCTGGCGCCAGCTAGCTCTTGAACAGCTAGATGAGCAAGATGGTGATGCAGAACAAAGCAACGGAAAGATGAATGGTAGCACCTTAAATAAAG|  |  |  |  |  |  |  |  |  |  |  |  |  |  |  |  |  |  |  |  |  |  |  |  |  |  |  |  |  |  |  |  |  |  |  |  |  |  |  |  |  |  |  |  |  |  |  |  | | --- | --- | --- | --- | --- | --- | --- | --- | --- | --- | --- | --- | --- | --- | --- | --- | --- | --- | --- | --- | --- | --- | --- | --- | --- | --- | --- | --- | --- | --- | --- | --- | --- | --- | --- | --- | --- | --- | --- | --- | --- | --- | --- | --- | --- | --- | --- | --- | | Marmoset calJac1 Contig1452 351274 351401 - **AA** | CGATGGATTTTGGGTGGGGAAGTCTTCTTTGCGGAGTTGGCGCCAGCTAGCTCTTGAACAGCTAGATGAGCAAGACGGTGATGCAGAACAAAGCAACGGAAAGATGAACGGCAGCACCTTAAATAAAG|  |  |  |  |  |  |  |  |  |  |  |  |  |  |  |  |  |  |  |  |  |  |  |  |  |  |  |  |  |  |  |  |  |  |  |  |  |  |  |  | | --- | --- | --- | --- | --- | --- | --- | --- | --- | --- | --- | --- | --- | --- | --- | --- | --- | --- | --- | --- | --- | --- | --- | --- | --- | --- | --- | --- | --- | --- | --- | --- | --- | --- | --- | --- | --- | --- | --- | --- | | Tarsier tarSyr1 scaffold\_3926 16289 16416 + **AA** | TGATGGATTTTGGGTGGGGAAGTCCTCCTTGCGGAGCTGGCGCCAGCTGGCTCTTGAGCAACTAGATGAGCAAGACGGTGATGGAGACCAAAGCAATGGAAAAATGAACGGCAACACCTTGAACAAAG|  |  |  |  |  |  |  |  |  |  |  |  |  |  |  |  |  |  |  |  |  |  |  |  |  |  |  |  |  |  |  |  | | --- | --- | --- | --- | --- | --- | --- | --- | --- | --- | --- | --- | --- | --- | --- | --- | --- | --- | --- | --- | --- | --- | --- | --- | --- | --- | --- | --- | --- | --- | --- | --- | | Galago otoGar1 scaffold\_96755.1-30206 20280 20407 + **AC** | GGATGGATTTTGGGTGGGAAAATCCTCCTTGCGGAGTTGGCGCCAGCTGGCTCTCGAACAGCTGGACGAGCAAGATGGCGACACAGACCACAGCAATGGAAAAATGAATGGCAGCACCTTGAATAAAG|  |  |  |  |  |  |  |  |  |  |  |  |  |  |  |  |  |  |  |  |  |  |  |  | | --- | --- | --- | --- | --- | --- | --- | --- | --- | --- | --- | --- | --- | --- | --- | --- | --- | --- | --- | --- | --- | --- | --- | --- | | Mouse mm9 chr4 137181075 137181202 + **AG** | TGATGGATTTTGGGTAGGAAAGTCTTCCTTGCGTAGCTGGCGCCAGCTGGCTCTTGAACAACTAGATGAGCAAGATGGTGAGGCGGAGCAAAGCAACGGGAAGATAAACGGGAGCACCTTCAATAAAG|  |  |  |  |  |  |  |  |  |  |  |  |  |  |  |  | | --- | --- | --- | --- | --- | --- | --- | --- | --- | --- | --- | --- | --- | --- | --- | --- | | Cow bosTau4 chr2 135543495 135543622 + **AG** | TGATGGATTTTGGGTGGGGAAGTCCTCCTTACGCAGTTGGCGCCAGCTGGCTCTTGAACAGCTTGATGAGCAAGATGGCGATGCAGACCAAAGCAACGGGAAAATGAATGGCAACACCTTGAATAAAG|  |  |  |  |  |  |  |  | | --- | --- | --- | --- | --- | --- | --- | --- | | Dog canFam2 chr2 80345184 80345311 + **AA** | TGATGGATTTTGGGTGGGGAAATCCTCCCTGCGCAGTTGGCGCCAGCTCGCTCTTGAACAGTTAGATGAACAAGATGGTGATGTAGACCAAAGCAACGGGAAAATGAACGGCAACACATTGAGTAAAG | | | | | | | | | | | | | | | | | | | | | | | | | | | | | | | | | | | | | | | | | | | | | | | | | | | | | | | | | | | | | | | | | | | | | | | | | | | | | | | | | | | | |

**Alignment** (splice site sequences are in lowercase)  

```
Human      agCGATGGATTTTGGGTGGGGAAGTCCTCCTTGCGGAGTTGGCGCCAGCTAGCTCTTGAACAGCTGGATGAGCAAGATGG
Chimp      .a........................T.....................................................
Gorilla    .a..............................................................................
Orangutan  .a..............................................................................
Baboon     .aT..............................A....C..........................A..............
Marmoset   .a........................T..T...................................A...........C..
Tarsier    .aT...................................C...........G........G..A..A...........C..
Galago     .cG.................A..A..........................G.....C...........C...........
Mouse      ..T..............A..A.....T........T..C...........G...........A..A..............
Cow        ..T.............................A..C..............G..............T..............
Dog        .aT....................A......C....C..............C............T.A.....A........

Human      TGATGCAGAACAAAGCAACGGAAAGATGAACGGTAGCACCTTAAATAAAGgt
Chimp      ....................................................
Gorilla    ....................................................
Orangutan  ....................................................
Baboon     ..............................T.....................
Marmoset   .................................C..................
Tarsier    .....G...C........T.....A........C.A......G..C......
Galago     C..CA....C..C.....T.....A.....T..C........G.........
Mouse      ...G..G..G...........G.....A.....G........C.........
Cow        C........C...........G..A.....T..C.A......G.........
Dog        .....T...C...........G..A........C.A...A..G.G.......
```

---

## 3. uc001cax.1\_15\_15

**Summary**  

|  |  |  |  |  |  |  |  |  |  |  |  |  |  |  |  |  |  |  |  |  |  |  |  |  |  |
| --- | --- | --- | --- | --- | --- | --- | --- | --- | --- | --- | --- | --- | --- | --- | --- | --- | --- | --- | --- | --- | --- | --- | --- | --- | --- |
| No Exon ID Position (hg19) Dir Human acceptor Chimp acceptor Category Usage Gene symbol Protein accession mRNA accession Gene title Note|  |  |  |  |  |  |  |  |  |  |  |  |  | | --- | --- | --- | --- | --- | --- | --- | --- | --- | --- | --- | --- | --- | | 3 uc001cax.1\_15\_15 chr1:36932509 - AG AA (A1) shift; increase; inframe alternative CSF3R NP\_724781.1 NM\_156039.3 granulocyte colony-stimulating factor receptor  | | | | | | | | | | | | | | | | | | | | | | | | | |

**Orthologs**  

|  |  |  |  |  |  |  |  |  |  |  |  |  |  |  |  |  |  |  |  |  |  |  |  |  |  |  |  |  |  |  |  |  |  |  |  |  |  |  |  |  |  |  |  |  |  |  |  |  |  |  |  |  |  |  |  |  |  |  |  |  |  |  |  |  |  |  |  |  |  |  |  |  |  |  |  |  |  |  |  |  |  |  |  |  |  |  |  |
| --- | --- | --- | --- | --- | --- | --- | --- | --- | --- | --- | --- | --- | --- | --- | --- | --- | --- | --- | --- | --- | --- | --- | --- | --- | --- | --- | --- | --- | --- | --- | --- | --- | --- | --- | --- | --- | --- | --- | --- | --- | --- | --- | --- | --- | --- | --- | --- | --- | --- | --- | --- | --- | --- | --- | --- | --- | --- | --- | --- | --- | --- | --- | --- | --- | --- | --- | --- | --- | --- | --- | --- | --- | --- | --- | --- | --- | --- | --- | --- | --- | --- | --- | --- | --- | --- | --- | --- |
| Species Assembly Chromosome Exon start Exon end Dir Acceptor Exon sequence|  |  |  |  |  |  |  |  |  |  |  |  |  |  |  |  |  |  |  |  |  |  |  |  |  |  |  |  |  |  |  |  |  |  |  |  |  |  |  |  |  |  |  |  |  |  |  |  |  |  |  |  |  |  |  |  |  |  |  |  |  |  |  |  |  |  |  |  |  |  |  |  |  |  |  |  |  |  |  |  | | --- | --- | --- | --- | --- | --- | --- | --- | --- | --- | --- | --- | --- | --- | --- | --- | --- | --- | --- | --- | --- | --- | --- | --- | --- | --- | --- | --- | --- | --- | --- | --- | --- | --- | --- | --- | --- | --- | --- | --- | --- | --- | --- | --- | --- | --- | --- | --- | --- | --- | --- | --- | --- | --- | --- | --- | --- | --- | --- | --- | --- | --- | --- | --- | --- | --- | --- | --- | --- | --- | --- | --- | --- | --- | --- | --- | --- | --- | --- | --- | | Human hg19 chr1 36931958 36932509 - **AG** | CTGCCCGGACCCAGACAGGGACAGTGGCTGGGGCAGACATCTGAAATGAGCCGTGCTCTCACCCCACATCCTTGTGTGCAGGATGCCTTCCAGCTGCCCGGCCTTGGCACGCCACCCATCACCAAGCTCACAGTGCTGGAGGAGGATGAAAAGAAGCCGGTGCCCTGGGAGTCCCATAACAGCTCAGAGACCTGTGGCCTCCCCACTCTGGTCCAGACCTATGTGCTCCAGGGGGACCCAAGAGCAGTTTCCACCCAGCCCCAATCCCAGTCTGGCACCAGCGATCAGGTCCTTTATGGGCAGCTGCTGGGCAGCCCCACAAGCCCAGGGCCAGGGCACTATCTCCGCTGTGACTCCACTCAGCCCCTCTTGGCGGGCCTCACCCCCAGCCCCAAGTCCTATGAGAACCTCTGGTTCCAGGCCAGCCCCTTGGGGACCCTGGTAACCCCAGCCCCAAGCCAGGAGGACGACTGTGTCTTTGGGCCACTGCTCAACTTCCCCCTCCTGCAGGGGATCCGGGTCCATGGGATGGAGGCGCTGGGGAGCTTCTAG|  |  |  |  |  |  |  |  |  |  |  |  |  |  |  |  |  |  |  |  |  |  |  |  |  |  |  |  |  |  |  |  |  |  |  |  |  |  |  |  |  |  |  |  |  |  |  |  |  |  |  |  |  |  |  |  |  |  |  |  |  |  |  |  |  |  |  |  |  |  |  |  | | --- | --- | --- | --- | --- | --- | --- | --- | --- | --- | --- | --- | --- | --- | --- | --- | --- | --- | --- | --- | --- | --- | --- | --- | --- | --- | --- | --- | --- | --- | --- | --- | --- | --- | --- | --- | --- | --- | --- | --- | --- | --- | --- | --- | --- | --- | --- | --- | --- | --- | --- | --- | --- | --- | --- | --- | --- | --- | --- | --- | --- | --- | --- | --- | --- | --- | --- | --- | --- | --- | --- | --- | | Chimp panTro2 chr1 37006241 37006792 - **AA** | CTGCCCGGACCCAGACAGGGACGGTGGCTGGGGCACACATCTGAAATGAGCCGTGCTCTCACCCCACATCCTTGTGTGCAGGATGCCTTCCAGCTGCCCGGCCTTGGCACGCCACCCATCACCAAGCTCACAGTGCTGGAGGAGGATGAGAAGAAGCCGGTGCCCTGGGAGTCCCATAACAGCTCAGAGACCTGTGGCCTCCCTACTCTGGTCCAGACCTATGTGCTCCAGGGGGACCCAAGAGCAGCTTCCACCCAGCCCCAATCCCAGTCTGGCACCAGCGATCAGGTCCTTTATGGGCAGCTGCTGGGCAGCCCCACAAGCCCAGGGCCAGGGCACTATCTCCGCTGTGACTCCACTCAGCCTCTCTTGGCGGGCCTCACCCCCAGCCCCAAGTCCTATGAGAACCTCTGGTTCCAGGCCAGCCCCTTGGGGACCCTGGTAACCCCAGCCCCAAGCCAGGAGGACGACTGTGTCTTTGGGCCACTGCTCAACTTCCCCCTCCTGCAGGGGATCCGGGTCCATGGGATGGAGGCGCTGGGGAGCTTCTAG|  |  |  |  |  |  |  |  |  |  |  |  |  |  |  |  |  |  |  |  |  |  |  |  |  |  |  |  |  |  |  |  |  |  |  |  |  |  |  |  |  |  |  |  |  |  |  |  |  |  |  |  |  |  |  |  |  |  |  |  |  |  |  |  | | --- | --- | --- | --- | --- | --- | --- | --- | --- | --- | --- | --- | --- | --- | --- | --- | --- | --- | --- | --- | --- | --- | --- | --- | --- | --- | --- | --- | --- | --- | --- | --- | --- | --- | --- | --- | --- | --- | --- | --- | --- | --- | --- | --- | --- | --- | --- | --- | --- | --- | --- | --- | --- | --- | --- | --- | --- | --- | --- | --- | --- | --- | --- | --- | | Gorilla gorGor1 Supercontig\_0053709 14103 14654 - **AA** | CTGCCCGGACCCAGACAGGGACGGTGACTGGGGCAGACATCTGAAATAAGCCGTGCTCTCACCCCACATCCTTGTGTGCAGGATGCCTTCCAGCTGCCCGGCCTTGGCACGCCACCCATCACCAAGCTCACAGTGCTGGAGGAGGACGAGAAGAAGCCAGTGCCCTGGGAGTCCCATAACAGCTCAGAGACCTGTGGCCTCCCCACTCTGGTCCAGACCTATGTGCTCCAGGGGGACCCAAGAGCAGCTTCCACCCAGCCCCAATCCCAGTCTGGCACCAGCGATCAGGTCCTTTATGGGCAGCTGCTGGGCAGCCCCACAAGCCCAGGGCCAGGGCACTATCTCCGCTGTGACTCCACTCAGCCCCTCTTGGCGGGCCTCACCCCCAGCCCCAAGTCCTATGAGAACCTCTGGTTCCAGGCCAGCCCCTTGGGGACCCTGGTAACCCCAGCCCCAAGCCAGGAGGACGACTGTGTCTTTGGGCCACTGCTCAACTTCCCCCTCCTGCAGGGGATCCGGGTCCATGGGATGGAGGCGTTGGGGAGCTTCTAG|  |  |  |  |  |  |  |  |  |  |  |  |  |  |  |  |  |  |  |  |  |  |  |  |  |  |  |  |  |  |  |  |  |  |  |  |  |  |  |  |  |  |  |  |  |  |  |  |  |  |  |  |  |  |  |  | | --- | --- | --- | --- | --- | --- | --- | --- | --- | --- | --- | --- | --- | --- | --- | --- | --- | --- | --- | --- | --- | --- | --- | --- | --- | --- | --- | --- | --- | --- | --- | --- | --- | --- | --- | --- | --- | --- | --- | --- | --- | --- | --- | --- | --- | --- | --- | --- | --- | --- | --- | --- | --- | --- | --- | --- | | Orangutan ponAbe2 chr1 193720293 193720844 + **AA** | CTGCCTGGACCCAGACAGGGATGGTGGCTGGGGCAGACATCTGAAAAGAGCCGTGCTCTCACCCCACATCCTTGTGTGCAGGATGCCTTCCAGCTGCCCGGCCTTGGCACGCCACCCATCACCAAGCTCACAGTGCTGGAGGAGGACAAGAAGAAGCCGGTGCCCTGGGAATCCCATAACAGCTCAGAGACCTGTGGCCTCCCCACTCTGGTCCAAACCTATGTGCTCCAGGGGGACCCAAGAGCAGCTTCCACCCAGCCCCAATCCCAGTCTGGCACCAGCGATCAGGTCCTTTATGGGCAGCTGCTGGGCAGCCCCACAAGCCCAGGGCCAGGGCACTATCTCCGCTGTGACTCCACTCAGCCCCTCTTGGCGGGCCTCACCCCCAGCCCCAAGTCCTATGAGAACCTCTGGTTCCAGGCCAGCCCCCTGGGGACCCTGGTAACCCCAGCCCCAAGCCAGGAGGACGACTGTGTCTTTGGGCCACTGCTCGACTTCCCCCTCCTGCAGGGGATCCGGGTGCATGGGATGGAGGGGCTGGGGAGCTTCTAG|  |  |  |  |  |  |  |  |  |  |  |  |  |  |  |  |  |  |  |  |  |  |  |  |  |  |  |  |  |  |  |  |  |  |  |  |  |  |  |  |  |  |  |  |  |  |  |  | | --- | --- | --- | --- | --- | --- | --- | --- | --- | --- | --- | --- | --- | --- | --- | --- | --- | --- | --- | --- | --- | --- | --- | --- | --- | --- | --- | --- | --- | --- | --- | --- | --- | --- | --- | --- | --- | --- | --- | --- | --- | --- | --- | --- | --- | --- | --- | --- | | Rhesus rheMac2 chr1 39331835 39332386 - **AA** | CTGCCTGGACCTAGACAGGGACGGTGGCTGGGGCAGACATCTGAAAGGAGCCGTGCCCTCACCCCACATCCTTGTGTGCAGGAGGCCTTCCAGCTGCCTGGCCTGGGCATGCCACCCATCACCAAGCTCACAGTGCTGGAGGAGGACGAGAAGAAGCCACTGCCCTGGGAGTCCCATAACAGCTCAGAGACCTGTGGCCTCCCCACTCTGGTCCAGACCTATGTGCTCCAGGGGGACCTAAGAGCAGCTTCCGCCCAGCCCCAATCCCAGTCTGGCACCAGCAATCAGGTCCTCTACGGGCAGCTGCTGGGCAGCCCCACAAGCCCAGGGCCAGGGCACTATCTCCGCTGCGACTCCACTCAGCCCCTCTTGGCGGGCCTCACCCCCAGCCCCAAGTCCTATGAGAACCTCTGGTTCCAGGCCAGCCCCCTGGGGACCCTGGTAACCCCAGCCCCAAGCCAGGAGGACGACTGTGTCTTTGGGCCACTGCTCGACTTCCCCCTCCTGCAGGGGATCCGAGTCCATGGGGTGGAGGGGCTGGAAAGCTTCTAG|  |  |  |  |  |  |  |  |  |  |  |  |  |  |  |  |  |  |  |  |  |  |  |  |  |  |  |  |  |  |  |  |  |  |  |  |  |  |  |  | | --- | --- | --- | --- | --- | --- | --- | --- | --- | --- | --- | --- | --- | --- | --- | --- | --- | --- | --- | --- | --- | --- | --- | --- | --- | --- | --- | --- | --- | --- | --- | --- | --- | --- | --- | --- | --- | --- | --- | --- | | Baboon papHam1 scaffold7406 46194 46745 - **AA** | CTGCCTGGACCTAGACAGGGACGGTGGCTGGGGCAGACATCTGAAAGGAGCCGTGCCCTCACCCCACATCCTTGTGTGCAGGAGGCCTTCCAGCTGCCCGGCCTGGGCATGCCACCCATCACCAAGCTCACAGTGCTGGAGGAGGACGAGAAGAAGCCACTGCCCTGGGAGTCCCATGACAGCTCAGAGACCTGTGGCCTCCCCACTCTGGTCCAGACCTACGTGCTCCAGGGGGACCTAAGAGCAGCTTCCGCCCAGCCCCAATCCCAGTCCGGCACCAGCAATCAGGTCCTCTACGGGCAGCTGCTGGGCAGCCCCACAAGCCCAGGGCCAGGGCACTATCTCCGCTGCGACTCCACTCAGCCCCTCTTGGCGGGCCTCACCCCCAGCCCCAAGTCCTATGAGAACCTCTGGTTCCAGGCCAGCCCCCTGGGGACCCTGGTAACCCCAGCCCCAAGCCAGGAGGACGACTGTGTCTTTGGGCCACTGCTCGACTTCCCCCTCCTGCAGGGGATCCGGGTCCATGGGGTGGAGGGGCTGGAAAGCTTCTAG|  |  |  |  |  |  |  |  |  |  |  |  |  |  |  |  |  |  |  |  |  |  |  |  |  |  |  |  |  |  |  |  | | --- | --- | --- | --- | --- | --- | --- | --- | --- | --- | --- | --- | --- | --- | --- | --- | --- | --- | --- | --- | --- | --- | --- | --- | --- | --- | --- | --- | --- | --- | --- | --- | | Marmoset calJac1 Contig2067 341153 341693 + **CA** | CTGCCAGAACCCAGACAGGGACTGTGGCTGGGGCAGACATATGAAATGAGCCCCACATCCTTGTGTGCAGGAGGCCTTCCAGCTGCCCAGCCTCGGCACGCCACCCATCACCAAGCTCACAGTGCTGGAGGAGGAGAAGAAGAAGCCGGTGCCCTGGGAGTCTCTTAACAGCTCCGAGACTTGTGGCCTCCCCACTCTGGTCCAGCCCTATGTGCTCCAGAGGGACCCAAGAACAGCTTCCACCCAGCCCCAATCCCAGTCTGGCACCAGCAATCAGGTCCTTTATGGGCAGCTGGTGGGCAGTCCCACAAGCCCAGGGCCAGGGCACTACCTCCGCTGTGACTCCACTCAGCCCCTCTTGGGGGGCGTCAGCCCCAGCACTAAGTCCTATGAGAACCTCTGGTTCCAGGCCAGCCCCCTGGGGACCCTGGTAGCCCCAGCCCCAAGCCAGGAGGACGACTGTGTCTTTGGGCCACTGCTCGACTTCCCCCTCCTGCAGGGGATCCAGGTCCATGGGGTGGAGGGGTTGGGGAGCTTCTAG|  |  |  |  |  |  |  |  |  |  |  |  |  |  |  |  |  |  |  |  |  |  |  |  | | --- | --- | --- | --- | --- | --- | --- | --- | --- | --- | --- | --- | --- | --- | --- | --- | --- | --- | --- | --- | --- | --- | --- | --- | | Lemur micMur1 scaffold\_1740 123257 123807 + **TA** | CTGCCCCAACTCAGGAAGGGACAGTGGCTGGGGTAGATATCTGAAATGAACCATGCCCTCCCCCAATGTCCTGTGTCCAGGAGACCTTCCAGCTGCCCAGCCTTCGGGACCCTGGCATGCCACCCATCACCAAGATCACAGTGCTAGAGGAGGAAGAGAAGAAGCTGGGGCCCTGGGAGTCCAGTGACAGCTCAGAGACCTGCCTCCCCACCCTGGTCCAGACCTATGTGCTCCAAGGGGACCCAAAAGCAGCTTCTACCCAGCCACAGGCCGAATCTAGCACCAGCGACCCGGTCCTTTACGGGCAGGTGCTGGGCAGCCCCACAGGCCCAGGGCACTACCTCCGCTATGACTCTACTCAGCCCCTTTTGGGGGGCCTCACTCCCAGCCCCAAGTGTTATGAGAACCTCTGGTTCCAGACCAGCCCCTTGAGGTCCCCTGTACCCTCAGACCCAAGCCAGGAGGATGACTGTGTCTTTGGGCCACTGCTAGACTTCCCCCTCCTGCAGGGGCTCCAGGTCCATGGGGTGGAGGGACTAGGGGGCTTCTAG|  |  |  |  |  |  |  |  |  |  |  |  |  |  |  |  | | --- | --- | --- | --- | --- | --- | --- | --- | --- | --- | --- | --- | --- | --- | --- | --- | | Mouse mm9 chr4 125720527 125721082 + **AA** | CTACCTCATCTCTGGAAGGAACGATGTGTGGAGCAGACATGTGAGACAAACCATGCCGTATTGTGTATATCCCTGTGTTCAGGAAACCTTCCAGTTACCCAGCTTCTGGGACTCCAGCGTGCCATCAATCACCAAGATCACTGAACTGGAGGAAGACAAGAAACCGACCCACTGGGATTCCGAAAGCTCTGGGAATGGTAGCCTTCCAGCCCTGGTTCAGGCCTATGTGCTCCAAGGAGATCCAAGAGAAATTTCCAACCAGTCCCAGCCTCCCTCTCGCACTGGTGACCAGGTCCTCTATGGTCAGGTGCTTGAGAGCCCCACCAGCCCAGGAGTAATGCAGTACATTCGCTCTGACTCCACTCAGCCCCTCTTGGGGGGCCCCACCCCTAGCCCTAAATCTTATGAAAACATCTGGTTCCATTCAAGACCCCAGGAGACCTTTGTGCCCCAACCTCCAAACCAGGAAGATGACTGTGTCTTTGGGCCTCCATTTGATTTTCCCCTCTTTCAGGGGCTCCAGGTCCATGGAGTTGAAGAACAAGGGGGTTTCTAG|  |  |  |  |  |  |  |  | | --- | --- | --- | --- | --- | --- | --- | --- | | Cow bosTau4 chr3 116643351 116643910 + **CA** | CCACCCCAGTGCAGGAAGGGACAGTGGCTGGAACAGACATCTGAAATGAACCAGGCCCTCACCACACATCTTTATGTCAGGATATCTTACAGCTGCCCAGCCTTCGGGACCCCGGCATGCCACCCATCACCAAGATCACGGTGCTGGAGGAGGAAGAGAAGAAGCCAGGGCCCTGGGAGTCCAATGCCAGCTCAGGGCCCGGTAGCCTCTCCACCCTTGTCCAGGCCTATGTGCTCCAGGGGGACCCAAGAGTGCCCTCCGCTCAGCCTCAGCCCCAGCCTGGCAACAGCGACCAGGTGCTTTACGTGCAGGTGCTGGGCAGCCCCACGGGCCCAGGGCCTGGGCACTACCTCCGCTGCGACTCAACTCAGCCCCTCTTGGAGGGCCTCTCCCCCAGTCCCAAGTCCTACGAGAACCTCTGGTTCCAGACCAGCTCTCCGGGGACCCCAGAGCCCCTAGTCCCACATCCGGAGGACGACAGTATCTTTGAGCCTCTGCTTGACTTCCCTCTACTACAAGGACTCCGGGTCAGTGGGGCGGAGGGTCTTGGGGGCTTCTAG | | | | | | | | | | | | | | | | | | | | | | | | | | | | | | | | | | | | | | | | | | | | | | | | | | | | | | | | | | | | | | | | | | | | | | | | | | | | | |

**Alignment** (splice site sequences are in lowercase)  

```
Human      agCTGCCCGGACCCAGACAGGGACAGTGGCTGGGGCAGACATCTGAAATGAGCCGTGCTCTCAC-CCCACATCCTTGTGT
Chimp      .a......................G............C..........................-...............
Gorilla    .a......................G...A....................A..............-...............
Orangutan  .a.....T...............TG.......................A...............-...............
Rhesus     .a.....T.....T..........G.......................G.........C.....-...............
Baboon     .a.....T.....T..........G.......................G.........C.....-...............
Marmoset   ca.....A.A..............T.................A..........------------...............
Lemur      ta......CA..T...GA.................T...T...........A..A...C...C.-..A.TG...-.....
Mouse      .a..A..TCAT.T.T.GA...A..GA..TG...A........G...G.CA.A..A...CG.ATTGTGT.T....C.....
Cow        ca.CA...CAGTG...GA...............AA................A..AG..C.....-.A......T..A...

Human      GCAGGATGCCTTCCAGCTGCCCGGCCTT---------GGCACGCCACCCATCACCAAGCTCACAGTGCTGGAGGAGGATG
Chimp      ............................---------...........................................
Gorilla    ............................---------.........................................C.
Orangutan  ............................---------.........................................CA
Rhesus     ......G..............T.....G---------....T....................................C.
Baboon     ......G....................G---------....T....................................C.
Marmoset   ......G...............A....C---------.........................................GA
Lemur      C.....GA..............A.....CGGGACCCT....T................A..........A........A.
Mouse      T.....AA........T.A...A..T.CTGGGACTCCA..GT....T.A.........A....T.AA........A..C-
Cow        -......AT...A.........A.....CGGGACCCC....T................A....G..............A.

Human      AAAAGAAGCCGGTGCCCTGGGAGTCCCATAACAGCTCAGAGACCTGTGGCCTCCCCACTCTGGTCCAGACCTATGTGCTC
Chimp      .G.....................................................T........................
Gorilla    .G........A.....................................................................
Orangutan  .G....................A............................................A............
Rhesus     .G........AC....................................................................
Baboon     .G........AC.................G...........................................C......
Marmoset   .G.......................T.T.........C.....T........................C...........
Lemur      .G.......T..G.............AG.G...............---..........C.....................
Mouse      --.....A...ACC.A......T...---G.A.....T.G..ATG..A....T..AG.C.....T...G...........
Cow        .G........A.G.............A..GC........G.C..G..A.....T....C..T......G...........

Human      CAGGGGGACCCAAGAGCAGTTTCCACCCAGCCCCAATCCCAGTCTGGCACCAGCGATCAGGTCCTTTATGGGCAGCTGCT
Chimp      ...................C............................................................
Gorilla    ...................C............................................................
Orangutan  ...................C............................................................
Rhesus     ..........T........C....G.............................A..........C..C...........
Baboon     ..........T........C....G...................C.........A..........C..C...........
Marmoset   ...A...........A...C..................................A.......................G.
Lemur      ..A..........A.....C...T........A..GG..G.A...A..........C.C.........C......G....
Mouse      ..A..A..T.......A.A......A....T....GC.T.CC...C....TG.T..C........C.....T...G....
Cow        ................TGCCC...G.T.....T..GC.....C......A......C.....G.....C.T....G....

Human      GGGCAGCCCCACAAGCCCAGGGCCAGGGCACTATCTCCGCTGTGACTCCACTCAGCCCCTCTTGGCGGGCCTCACCCCCA
Chimp      .........................................................T......................
Gorilla    ................................................................................
Orangutan  ................................................................................
Rhesus     ..........................................C.....................................
Baboon     ..........................................C.....................................
Marmoset   ......T..........................C...............................G....G...G.....
Lemur      .............G.......------......C.......A......T...........T....G.........T....
Mouse      T.AG........C........AGT.AT...G..CA.T....C.......................G.....C......T.
Cow        ............GG..........T........C........C.....A................A.......T......

Human      GCCCCAAGTCCTATGAGAACCTCTGGTTCCAGGCCAGCCCCTTGGGGACCCTGGTAACCCCAGCCCCAAGCCAGGAGGAC
Chimp      ................................................................................
Gorilla    ................................................................................
Orangutan  .........................................C......................................
Rhesus     .........................................C......................................
Baboon     .........................................C......................................
Marmoset   ..A.T....................................C..............G.......................
Lemur      .........GT.....................A...........A..T...CT...C..T...A...............T
Mouse      ....T..A..T.....A...A..........TT.A..A...CA..A....T.T..GC...A.C.T....A......A..T
Cow        .T...........C..................A.....T.TCC........CA.AGC...T..T....CAT.C.......

Human      GACTGTGTCTTTGGGCCACTGCTCAACTTCCCCCTCCTGCAGGGGATCCGGGTCCATGGGATGGAGGCGCTGGGGAGCTT
Chimp      ................................................................................
Gorilla    .....................................................................T..........
Orangutan  ........................G............................G.............G............
Rhesus     ........................G.........................A.........G......G.....AA.....
Baboon     ........................G...................................G......G.....AA.....
Marmoset   ........................G........................A..........G......G.T..........
Lemur      .......................AG....................C...A..........G......GA..A...G....
Mouse      .................T.CAT.TG.T..T......T.T......C...A.........AG.T..A.AA.AA...G.T..
Cow        ...A..A......A...T.....TG.......T..A..A..A..AC........AG....GC.....GT..T...G....

Human      CTAG
Chimp      ....
Gorilla    ....
Orangutan  ....
Rhesus     ....
Baboon     ....
Marmoset   ....
Lemur      ....
Mouse      ....
Cow        ....
```

---

## 4. uc001ctt.2\_5\_16

**Summary**  

|  |  |  |  |  |  |  |  |  |  |  |  |  |  |  |  |  |  |  |  |  |  |  |  |  |  |
| --- | --- | --- | --- | --- | --- | --- | --- | --- | --- | --- | --- | --- | --- | --- | --- | --- | --- | --- | --- | --- | --- | --- | --- | --- | --- |
| No Exon ID Position (hg19) Dir Human acceptor Chimp acceptor Category Usage Gene symbol Protein accession mRNA accession Gene title Note|  |  |  |  |  |  |  |  |  |  |  |  |  | | --- | --- | --- | --- | --- | --- | --- | --- | --- | --- | --- | --- | --- | | 4 uc001ctt.2\_5\_16 chr1:52859475 - AG GG (A1) shift; increase; inframe constitutive ORC1 NP\_004144.2 NM\_004153.3 origin recognition complex subunit 1  | | | | | | | | | | | | | | | | | | | | | | | | | |

**Orthologs**  

|  |  |  |  |  |  |  |  |  |  |  |  |  |  |  |  |  |  |  |  |  |  |  |  |  |  |  |  |  |  |  |  |  |  |  |  |  |  |  |  |  |  |  |  |  |  |  |  |  |  |  |  |  |  |  |  |  |  |  |  |  |  |  |  |  |  |  |  |  |  |  |  |  |  |  |  |  |  |  |  |  |  |  |  |  |  |  |  |
| --- | --- | --- | --- | --- | --- | --- | --- | --- | --- | --- | --- | --- | --- | --- | --- | --- | --- | --- | --- | --- | --- | --- | --- | --- | --- | --- | --- | --- | --- | --- | --- | --- | --- | --- | --- | --- | --- | --- | --- | --- | --- | --- | --- | --- | --- | --- | --- | --- | --- | --- | --- | --- | --- | --- | --- | --- | --- | --- | --- | --- | --- | --- | --- | --- | --- | --- | --- | --- | --- | --- | --- | --- | --- | --- | --- | --- | --- | --- | --- | --- | --- | --- | --- | --- | --- | --- | --- |
| Species Assembly Chromosome Exon start Exon end Dir Acceptor Exon sequence|  |  |  |  |  |  |  |  |  |  |  |  |  |  |  |  |  |  |  |  |  |  |  |  |  |  |  |  |  |  |  |  |  |  |  |  |  |  |  |  |  |  |  |  |  |  |  |  |  |  |  |  |  |  |  |  |  |  |  |  |  |  |  |  |  |  |  |  |  |  |  |  |  |  |  |  |  |  |  |  | | --- | --- | --- | --- | --- | --- | --- | --- | --- | --- | --- | --- | --- | --- | --- | --- | --- | --- | --- | --- | --- | --- | --- | --- | --- | --- | --- | --- | --- | --- | --- | --- | --- | --- | --- | --- | --- | --- | --- | --- | --- | --- | --- | --- | --- | --- | --- | --- | --- | --- | --- | --- | --- | --- | --- | --- | --- | --- | --- | --- | --- | --- | --- | --- | --- | --- | --- | --- | --- | --- | --- | --- | --- | --- | --- | --- | --- | --- | --- | --- | | Human hg19 chr1 52859115 52859475 - **AG** | ACTTAGGTAACCCTCAGATGTCCCAGCAGACTTCATGTGCCTCCTTGGATTCTCCAGGAAGAATAAAACGGAAAGTGGCCTTCTCGGAGATCACCTCACCTTCTAAGAGATCTCAGCCTGATAAACTTCAAACCTTGTCTCCAGCTCTGAAAGCCCCAGAGAAAACCAGAGAGACTGGACTCTCTTATACTGAGGATGACAAGAAGGCTTCACCTGAACATCGCATAATCCTGAGAACCCGAATTGCAGCTTCGAAAACCATAGACATTAGAGAGGAGAGAACACTTACCCCTATCAGTGGGGGACAGAGATCTTCAGTGGTGCCATCCGTGATTCTGAAACCAGAAAACATCAAAAAGAG|  |  |  |  |  |  |  |  |  |  |  |  |  |  |  |  |  |  |  |  |  |  |  |  |  |  |  |  |  |  |  |  |  |  |  |  |  |  |  |  |  |  |  |  |  |  |  |  |  |  |  |  |  |  |  |  |  |  |  |  |  |  |  |  |  |  |  |  |  |  |  |  | | --- | --- | --- | --- | --- | --- | --- | --- | --- | --- | --- | --- | --- | --- | --- | --- | --- | --- | --- | --- | --- | --- | --- | --- | --- | --- | --- | --- | --- | --- | --- | --- | --- | --- | --- | --- | --- | --- | --- | --- | --- | --- | --- | --- | --- | --- | --- | --- | --- | --- | --- | --- | --- | --- | --- | --- | --- | --- | --- | --- | --- | --- | --- | --- | --- | --- | --- | --- | --- | --- | --- | --- | | Chimp panTro2 chr1 53261382 53261742 - **GG** | ACTTAGGTAACCCTCAGATGTCCCAGCAGACTTCATGTGCCTCCTTGGATTCTCCAGGAAGAATAAAACGGAAAGTGGCCTTCTCGGAGATCACCTCACCTTCTAAGAGATCTCAGCCTGATAAACTTCAAACCTTGTCTCTAGCTCTGAAAGCCCCAGAGAAAACCAGAGAGACTGGACTCTCTTATACTGAGGATGACAAGAAGGCTTCATCTGAACATCGCATAATCCTGAGAACCCGAATTCCAGCTTCGAAAACCACAGACATTAGAGAGGAGAGAACACTTACCCCTATCAGTGGGGGACAGAGATCTTCAGTGGTGCCATCCGTGATTCTGAAACCAGAAAACATCAAAAAGAG|  |  |  |  |  |  |  |  |  |  |  |  |  |  |  |  |  |  |  |  |  |  |  |  |  |  |  |  |  |  |  |  |  |  |  |  |  |  |  |  |  |  |  |  |  |  |  |  |  |  |  |  |  |  |  |  |  |  |  |  |  |  |  |  | | --- | --- | --- | --- | --- | --- | --- | --- | --- | --- | --- | --- | --- | --- | --- | --- | --- | --- | --- | --- | --- | --- | --- | --- | --- | --- | --- | --- | --- | --- | --- | --- | --- | --- | --- | --- | --- | --- | --- | --- | --- | --- | --- | --- | --- | --- | --- | --- | --- | --- | --- | --- | --- | --- | --- | --- | --- | --- | --- | --- | --- | --- | --- | --- | | Orangutan ponAbe2 chr1 177353853 177354213 + **GG** | GCTTAGGTAACCCTCAGATGTCCCAGCAGACTTCATGTGCCTCCTTGGATTCTCCAGGAAGAATAAAACGGAGAGTGGCCTTCTCGGAGATCACCTCACCTTCTAAGAGATCTCAGCCTGATAAACTTCAAACCTTGTCTCCAGCTCTGAAAGCCCCAGAGAAAACCAGAGAGACTGGACTCTCTTATACTGAGGATGACAAGAAGGCTTCACCTGAATGTTGCGTAATCCTGAGAACCCGAATTCCAGCTTTGAAAACCATAGACATTAGAGAGGAGAGAACACTTACCCCTATCAGTGGGGGACAGAGATCTTCAGTGGTGCCATCCGTGATTCTGAAACCAGAAAACATCAAAAAGAG|  |  |  |  |  |  |  |  |  |  |  |  |  |  |  |  |  |  |  |  |  |  |  |  |  |  |  |  |  |  |  |  |  |  |  |  |  |  |  |  |  |  |  |  |  |  |  |  |  |  |  |  |  |  |  |  | | --- | --- | --- | --- | --- | --- | --- | --- | --- | --- | --- | --- | --- | --- | --- | --- | --- | --- | --- | --- | --- | --- | --- | --- | --- | --- | --- | --- | --- | --- | --- | --- | --- | --- | --- | --- | --- | --- | --- | --- | --- | --- | --- | --- | --- | --- | --- | --- | --- | --- | --- | --- | --- | --- | --- | --- | | Rhesus rheMac2 chr1 55228661 55229018 - **GG** | GCTTAGGTAACCCTCAGATGTCCCAGCAGACTTCACGTGCCTCCTTGGATTCTCCAGGAAGAATGAAACGGAAAGTGGCCTTCTCGGAGATCACCTCACCTTCTAAGAGATCTCAGCCTGATAAACTTCAGACCTTGTCTCCAGCTCTGAAAGCCCCAGAGAAAACCAGAGAGACTGGACTCTGTTATACTGAGGATGATGAGGCTTCACCCAAACGTTGCATAATCCTGAGAACCCGAATTCCAGCTTCGAAAACCATAGACATTAAAGAGGAGAGAATACTTACCCCTATCAGAGGGGGACAGAAATCTTCAGTGATGCCATCTGTGATTCTGAAACCAGAAAACATCAAAAAGAG|  |  |  |  |  |  |  |  |  |  |  |  |  |  |  |  |  |  |  |  |  |  |  |  |  |  |  |  |  |  |  |  |  |  |  |  |  |  |  |  |  |  |  |  |  |  |  |  | | --- | --- | --- | --- | --- | --- | --- | --- | --- | --- | --- | --- | --- | --- | --- | --- | --- | --- | --- | --- | --- | --- | --- | --- | --- | --- | --- | --- | --- | --- | --- | --- | --- | --- | --- | --- | --- | --- | --- | --- | --- | --- | --- | --- | --- | --- | --- | --- | | Baboon papHam1 scaffold6699 47359 47716 - **GG** | GCTTAGGTAACCCTCAGATGTCCCAGCAGACTTCACGTGCCTCCTTGGATTCTCCAGGAAGAATGAAACGGAAAGTGGCCTTCTCGGAGATCACCTCACCTTCTAAGAGATCTCAGCCTGATAAACTTCAGACCTTGTCTCCAGCTCTGAAAGCCCCAGAGAAAACCAGAGAGACTGGACTCTGTTATACTGAGGATGATGAGGCTTCACCCAAACGTTGCATAATCCTGAGAACCCGAATTCCAGCTTCGAAAACCATAGACATTAAAGAGGAGAGAATACTTACCCCTATCAGAGGGGGACAGAAATCTTCAGTGATGCCATCTGTGATTCTGAAACCAGAAAACATCAAAAAGAG|  |  |  |  |  |  |  |  |  |  |  |  |  |  |  |  |  |  |  |  |  |  |  |  |  |  |  |  |  |  |  |  |  |  |  |  |  |  |  |  | | --- | --- | --- | --- | --- | --- | --- | --- | --- | --- | --- | --- | --- | --- | --- | --- | --- | --- | --- | --- | --- | --- | --- | --- | --- | --- | --- | --- | --- | --- | --- | --- | --- | --- | --- | --- | --- | --- | --- | --- | | Lemur micMur1 scaffold\_5022 61445 61820 - **GG** | GCTTAGGTTTACCAAGGACACCTAACACTAGGATGTGCCAGCAAACTTCATCTGCCTCCTTGAGTTCTCCAGAAGGAAATAAACGGAAAGTGGCTTTCTCCGAGATCACCTCACCTTCTAAGAGGTGTCAGCCTGATAAACTTCAGACCCTGTCTCCAGTTCTGAAAGCCCCAGAGAAAACTGGAAAGATGCCTCTCTCTTGTACTGGTGCTGACAAGAAGGCTTCACCTGAATGTCACATGATCCTGAGAGCCCGAATCACTGCTTTGGAAGCCACAGAAACTAGCAAGGAAAGAACACTTTCCCCTATCAGTGGGGGTCAGAGATCCTCAGTGATGGCTTCAGTGATTCTGAAACCAGAAAGCATCAAAAAGAG|  |  |  |  |  |  |  |  |  |  |  |  |  |  |  |  |  |  |  |  |  |  |  |  |  |  |  |  |  |  |  |  | | --- | --- | --- | --- | --- | --- | --- | --- | --- | --- | --- | --- | --- | --- | --- | --- | --- | --- | --- | --- | --- | --- | --- | --- | --- | --- | --- | --- | --- | --- | --- | --- | | Galago otoGar1 scaffold\_83145.1-54844 20349 20721 - **GG** | GCTTAGGTTTCCCTAGGACACCTAACACGAGGATGTCGCAGCAGACTTCATGTGCCTCCTCGGGTTCTCTGGGAGTGAGTAAACGGAAAATGGCCCTCTCTGAGATCACTTCACCTTCTAAGAGGTCCCAGCCTGATAAACTTCAAACCTTATCTCCAGTTCGGAAAGCCCCAGAGAAAAGTGGAAAGATTCGACTCTTTGGTACTAAAGATGACAAGAAGGCCTTACCCGAACATCACATGATCCTGAGAACCCGAATCTCAGCTTTGGAAACCACAGAAGTTAGTGGGGACACAACACTTAACCCTGTCAGCGGGGGATGGAGATCCTCAGAGGTGCCTTCCATCATCCTGAAGCCAGTAATCAAAAAGAG|  |  |  |  |  |  |  |  |  |  |  |  |  |  |  |  |  |  |  |  |  |  |  |  | | --- | --- | --- | --- | --- | --- | --- | --- | --- | --- | --- | --- | --- | --- | --- | --- | --- | --- | --- | --- | --- | --- | --- | --- | | Mouse mm9 chr4 108269735 108270083 + **AG** | GCTTAGGTTTTACCAGGAAGCCTAACACGAGGTGGTCAAAGAAGAGCTCGTGTGACTCCTTGGATTATCAAAAAACATCTAAAAGAAGAGCAGCCTTCTCTGAGACCACCTCGCCTCCTAAAAAGCCTAATAAACCTCGTGAAATCAAGCCCTCTTCAGCTTTGGAAACTCGAGTTAAAAATGGACAAACTCAACCTTTTTGTGCCAAAAGTAGTGTGGTTCTGAGAGCCCGGAACCCAGCTATGACGACCACAAAGCTTGGTGTGGACAACACACTTAGCCCTATCAGGAATGGGTTGAGATCTTCAGTGGTGCCCTCTGGGGGTCTGACACCAGTATACATTAGGAG|  |  |  |  |  |  |  |  |  |  |  |  |  |  |  |  | | --- | --- | --- | --- | --- | --- | --- | --- | --- | --- | --- | --- | --- | --- | --- | --- | | Cow bosTau4 chr3 100723002 100723377 + **GA** | ACTTAGGTTTCCCTAGGACTCCTAACACAAGGATTTCCCCGGCAGCTTCGTGTGCCTCATTGGATTCTCCTGGAAGAATGAAACGGAAAGTGGCCTTCTCTGAGGTCATGTCACCTTCAAAGAGGTCTCTGCCTGATGGTTTTCAGACCTCATCTCCAGCTCTGAAAGCCCCAGAGAAAACTGGAGAGATTCAACACTCCTGTACCAAAGATGCCAAGAAGACCTCACCTGATCATGGCATGATCCTGAGAGCTCGAGCCCCAGCTTTGAAAATAACAGAGACTAGTGAAGAAAGAACACTTACTCCTATCGGCGGGGGACGGAAATCCTCAGTGGTGCCTTCTGTGATTCTGAAACCAGAATACATCAAAAGAAG|  |  |  |  |  |  |  |  | | --- | --- | --- | --- | --- | --- | --- | --- | | Dog canFam2 chr15 11988227 11988602 + **GG** | GATTAGGTTTCCCTAGGACACCTAGCACTAGGATTTCCCAGCAGAATTCAGGGGCCTTGTTGGATTCTCCCGGAAGAAGTAAAAGAAAAGTGGCCTTATCTGAGATCACCTCACCTACTAAGAGGTCTCAGCCTGATGAACTTCATACCTTGTCTCCAGCTCTGAAAACTCCACAGAAAACTGGAGAGATTCACAGCTCATGTGCCAGGAATGACAAGAAGGCATCACCTGACTGTCAGAGAATCCTGAGAACCCGAGTCCCAGCTTTGAATACCATGGAGATTATTGAGGAAAGAACACTTAAGCCTATCTACAGGGGCTGGCCATCCTCGGTGGTGCCTTCCGTGATTCTGACACCAAAAAACATCAAAAAGAG | | | | | | | | | | | | | | | | | | | | | | | | | | | | | | | | | | | | | | | | | | | | | | | | | | | | | | | | | | | | | | | | | | | | | | | | | | | | | |

**Alignment** (splice site sequences are in lowercase)  

```
Human      agACTTAGG---------------TAACCCTCAGATGTCCCAGCAGACTTCATGTGCCTCCTTGGATTCTCCAGGAAGAA
Chimp      g........---------------........................................................
Orangutan  g.G......---------------........................................................
Rhesus     g.G......---------------............................C...........................
Baboon     g.G......---------------............................C...........................
Lemur      g.G......TTTACCAAGGACACC....A..AG.....G......A.......C..........AG........A.G...
Galago     g.G......TTTCCCTAGGACACC....A.GAG......G......................C..G.....TG...GTG.
Mouse      ..G......TTTTACCAGGAAGCC....A.GAG.TG...AA..A...GC..G....A...........A..A.AA..C.T
Cow        ga.......TTTCCCTAGGACTCC....A.AAG...T....C.GCAG....G........A...........T.......
Dog        g.GA.....TTTCCCTAGGACACC..G.A..AG...T..........A....G.G....TG...........C.......

Human      TAAAACGGAAAGTGGCCTTCTCGGAGATCACCTCACCTTCTAAGAGATCTCAGCCTGATAAACT--------TCAAACCT
Chimp      ................................................................--------........
Orangutan  .........G......................................................--------........
Rhesus     .G..............................................................--------...G....
Baboon     .G..............................................................--------...G....
Lemur      AT..............T.....C.......................G.G...............--------...G...C
Galago     GT.........A.....C....T........T..............G..C..............--------........
Mouse      CT...A.A.G..CA........T....C......G...C....A.------.....A......CTCGTGAAA....G..C
Cow        .G....................T...G...TG........A.....G....T.......GGTT.--------...G....
Dog        GT...A.A...........A..T...............A.......G............G....--------...T....

Human      TGTCTCCAGCTCTGAAAGCCCCAGAGAAAACCAGAGAGACTGGACTCTCTTATACTGAGGATGACAAGAAGGCTTCACCT
Chimp      ......T......................................................................T..
Orangutan  ................................................................................
Rhesus     ................................................G...............T---G..........C
Baboon     ................................................G...............T---G..........C
Lemur      .........T.....................TG..A...TGCCT.......G.....GT.C...................
Galago     .A.......T..G.................GTG..A...T.C......T.GG....A.A..............C.T...C
Mouse      --...T.....T..G..A.T.G..TT....ATG..C.A...CA..CT.T..G.G.CA.AA--------------------
Cow        CA.............................TG......T.CA..A...C.G...CA.A....C.......A.C......
Dog        .................A.T...C.......TG......T.CACAG...A.G.G.CAG.A.............A......

Human      GAACATCGCATAATCCTGAGAACCCGAATTGCAGCTTCGAAAACCATAGACATTAGAGAGGAGAGAACACTTACCCCTAT
Chimp      ..............................C...............C.................................
Orangutan  ...TG.T..G....................C......T..........................................
Rhesus     A...G.T.......................C........................A...........T............
Baboon     A...G.T.......................C........................A...........T............
Lemur      ...TG..A...G.........G.......CA.T....T.G..G...C...A.C...CA....A.........T.......
Galago     .......A...G.................CT......T.G......C...AG....T.G...C.C........A....G.
Mouse      ----G.A.TG.GG.T......G....G.ACC.....AT..CG....C.A.GC..G.T.T...C.AC.......G......
Cow        ..T...G....G.........G.T...GCCC......T.....TA.C...G.C...T..A..A...........T.....
Dog        ..CTG..AG.G................G.CC......T...T.....G..G....TT.....A..........AG.....

Human      CAGTGGGGGACAGAGATCTTCAGTGGTGCCATCCGTGATTCTGAAACCAGAAAACATCAAAAAGAGgt
Chimp      ....................................................................
Orangutan  ....................................................................
Rhesus     ...A..........A..........A.......T..................................
Baboon     ...A..........A..........A.......T..................................
Lemur      .........T........C......A..G.T..A...................G..............
Galago     ...C......TG......C....A......T...A.C..C.....G....T.---.............
Mouse      ...GAAT..GTT..................C..T.G.GG.....C.....T.T....T---.G.....
Cow        .G.C.......G..A...C...........T..T..................T.........GA....
Dog        .TACA....CTG.CC...C..G........T.............C....A..................
```

---

## 5. uc010ork.1\_15\_16

**Summary**  

|  |  |  |  |  |  |  |  |  |  |  |  |  |  |  |  |  |  |  |  |  |  |  |  |  |  |
| --- | --- | --- | --- | --- | --- | --- | --- | --- | --- | --- | --- | --- | --- | --- | --- | --- | --- | --- | --- | --- | --- | --- | --- | --- | --- |
| No Exon ID Position (hg19) Dir Human acceptor Chimp acceptor Category Usage Gene symbol Protein accession mRNA accession Gene title Note|  |  |  |  |  |  |  |  |  |  |  |  |  | | --- | --- | --- | --- | --- | --- | --- | --- | --- | --- | --- | --- | --- | | 5 uc010ork.1\_15\_16 chr1:78338686 + AG AA (A1) shift; increase; inframe alternative FAM73A B7ZLZ8 BC144167.1 family with sequence similarity 73, member A NAGNAG | | | | | | | | | | | | | | | | | | | | | | | | | |

**Orthologs**  

|  |  |  |  |  |  |  |  |  |  |  |  |  |  |  |  |  |  |  |  |  |  |  |  |  |  |  |  |  |  |  |  |  |  |  |  |  |  |  |  |  |  |  |  |  |  |  |  |  |  |  |  |  |  |  |  |  |  |  |  |  |  |  |  |  |  |  |  |  |  |  |  |  |  |  |  |  |  |  |  |  |  |  |  |  |  |  |  |
| --- | --- | --- | --- | --- | --- | --- | --- | --- | --- | --- | --- | --- | --- | --- | --- | --- | --- | --- | --- | --- | --- | --- | --- | --- | --- | --- | --- | --- | --- | --- | --- | --- | --- | --- | --- | --- | --- | --- | --- | --- | --- | --- | --- | --- | --- | --- | --- | --- | --- | --- | --- | --- | --- | --- | --- | --- | --- | --- | --- | --- | --- | --- | --- | --- | --- | --- | --- | --- | --- | --- | --- | --- | --- | --- | --- | --- | --- | --- | --- | --- | --- | --- | --- | --- | --- | --- | --- |
| Species Assembly Chromosome Exon start Exon end Dir Acceptor Exon sequence|  |  |  |  |  |  |  |  |  |  |  |  |  |  |  |  |  |  |  |  |  |  |  |  |  |  |  |  |  |  |  |  |  |  |  |  |  |  |  |  |  |  |  |  |  |  |  |  |  |  |  |  |  |  |  |  |  |  |  |  |  |  |  |  |  |  |  |  |  |  |  |  |  |  |  |  |  |  |  |  | | --- | --- | --- | --- | --- | --- | --- | --- | --- | --- | --- | --- | --- | --- | --- | --- | --- | --- | --- | --- | --- | --- | --- | --- | --- | --- | --- | --- | --- | --- | --- | --- | --- | --- | --- | --- | --- | --- | --- | --- | --- | --- | --- | --- | --- | --- | --- | --- | --- | --- | --- | --- | --- | --- | --- | --- | --- | --- | --- | --- | --- | --- | --- | --- | --- | --- | --- | --- | --- | --- | --- | --- | --- | --- | --- | --- | --- | --- | --- | --- | | Human hg19 chr1 78338686 78338805 + **AG** | CAGATCCCAGATGGATTTTTTGCCCATTTTTATGCCATTTGTGAACACATCAGTCCTGTCCTAGCCTGGGGCTTTTTGGGTCCTAGAAATTCTCTGTATGATTTATGTTGCTTTTTTAAG|  |  |  |  |  |  |  |  |  |  |  |  |  |  |  |  |  |  |  |  |  |  |  |  |  |  |  |  |  |  |  |  |  |  |  |  |  |  |  |  |  |  |  |  |  |  |  |  |  |  |  |  |  |  |  |  |  |  |  |  |  |  |  |  |  |  |  |  |  |  |  |  | | --- | --- | --- | --- | --- | --- | --- | --- | --- | --- | --- | --- | --- | --- | --- | --- | --- | --- | --- | --- | --- | --- | --- | --- | --- | --- | --- | --- | --- | --- | --- | --- | --- | --- | --- | --- | --- | --- | --- | --- | --- | --- | --- | --- | --- | --- | --- | --- | --- | --- | --- | --- | --- | --- | --- | --- | --- | --- | --- | --- | --- | --- | --- | --- | --- | --- | --- | --- | --- | --- | --- | --- | | Chimp panTro2 chr1 79201210 79201329 + **AA** | CAGATCCCAGATGGATTTTTTGCCCATTTTTATGCCATTTGTGAACACATCAGTCCTGTCCTAGCCTGGGGCTTTTTGGGTCCTAGAAATTCTCTGTATGATTTATGTTGCTTTTTTAAG|  |  |  |  |  |  |  |  |  |  |  |  |  |  |  |  |  |  |  |  |  |  |  |  |  |  |  |  |  |  |  |  |  |  |  |  |  |  |  |  |  |  |  |  |  |  |  |  |  |  |  |  |  |  |  |  |  |  |  |  |  |  |  |  | | --- | --- | --- | --- | --- | --- | --- | --- | --- | --- | --- | --- | --- | --- | --- | --- | --- | --- | --- | --- | --- | --- | --- | --- | --- | --- | --- | --- | --- | --- | --- | --- | --- | --- | --- | --- | --- | --- | --- | --- | --- | --- | --- | --- | --- | --- | --- | --- | --- | --- | --- | --- | --- | --- | --- | --- | --- | --- | --- | --- | --- | --- | --- | --- | | Gorilla gorGor1 Supercontig\_0542555 675 794 + **AA** | CAGATCCCAGATGGATTTTTTGCCCATTTTTATGCCATTTGTGAACACATCAGTCCTGTCCTAGCCTGGGGCTTTTTGGGTCCTAGAAATTCTCTGTATGATTTATGTTGCTTTTTTAAG|  |  |  |  |  |  |  |  |  |  |  |  |  |  |  |  |  |  |  |  |  |  |  |  |  |  |  |  |  |  |  |  |  |  |  |  |  |  |  |  |  |  |  |  |  |  |  |  |  |  |  |  |  |  |  |  | | --- | --- | --- | --- | --- | --- | --- | --- | --- | --- | --- | --- | --- | --- | --- | --- | --- | --- | --- | --- | --- | --- | --- | --- | --- | --- | --- | --- | --- | --- | --- | --- | --- | --- | --- | --- | --- | --- | --- | --- | --- | --- | --- | --- | --- | --- | --- | --- | --- | --- | --- | --- | --- | --- | --- | --- | | Orangutan ponAbe2 chr1 151258906 151259025 - **AA** | CAGATCCCAGATGGATTTTTTGCCCATTTTTATGCCATTTGTGAACACATCAGTCCTGTCCTAGCCTGGGGCTTTTTGGGTCCTAGAAATTCTCTCTATGATTTATGTTGCTTTTTTAAG|  |  |  |  |  |  |  |  |  |  |  |  |  |  |  |  |  |  |  |  |  |  |  |  |  |  |  |  |  |  |  |  |  |  |  |  |  |  |  |  |  |  |  |  |  |  |  |  | | --- | --- | --- | --- | --- | --- | --- | --- | --- | --- | --- | --- | --- | --- | --- | --- | --- | --- | --- | --- | --- | --- | --- | --- | --- | --- | --- | --- | --- | --- | --- | --- | --- | --- | --- | --- | --- | --- | --- | --- | --- | --- | --- | --- | --- | --- | --- | --- | | Rhesus rheMac2 chr1 80709557 80709676 + **AA** | CAGATCCCAGATGGATTTTTTGCCCATTTTTATGCCATTTGTGAACACATCAGTCCTGTCCTAGCCTGGGGCTTTTTGGGTCCTAGAAATTCTCTCTATGATTTATGTTGCTTTTTTAAG|  |  |  |  |  |  |  |  |  |  |  |  |  |  |  |  |  |  |  |  |  |  |  |  |  |  |  |  |  |  |  |  |  |  |  |  |  |  |  |  | | --- | --- | --- | --- | --- | --- | --- | --- | --- | --- | --- | --- | --- | --- | --- | --- | --- | --- | --- | --- | --- | --- | --- | --- | --- | --- | --- | --- | --- | --- | --- | --- | --- | --- | --- | --- | --- | --- | --- | --- | | Baboon papHam1 scaffold11774 55224 55343 - **AA** | CAGATCCCAGATGGATTTTTTGCCCATTTTTATGCCATTTGTGAACACATCAGTCCTGTCCTAGCCTGGGGCTTTTTGGGTCCTAGAAATTCTCTCTATGATTTATGTTGCTTTTTTAAG|  |  |  |  |  |  |  |  |  |  |  |  |  |  |  |  |  |  |  |  |  |  |  |  |  |  |  |  |  |  |  |  | | --- | --- | --- | --- | --- | --- | --- | --- | --- | --- | --- | --- | --- | --- | --- | --- | --- | --- | --- | --- | --- | --- | --- | --- | --- | --- | --- | --- | --- | --- | --- | --- | | Marmoset calJac1 Contig317 462421 462540 + **AA** | CAGATCCCAGATGGATTTTTTGCCCATTTTTATGCCATTTGTGAACACATCAGCCCTGTCCTAGCCTGGGGCTTTTTGGGTCCTAGAAATTCTCTCTATGATTTATGTTGCTTTTTTAAG|  |  |  |  |  |  |  |  |  |  |  |  |  |  |  |  |  |  |  |  |  |  |  |  | | --- | --- | --- | --- | --- | --- | --- | --- | --- | --- | --- | --- | --- | --- | --- | --- | --- | --- | --- | --- | --- | --- | --- | --- | | Lemur micMur1 scaffold\_1980 4086 4205 + **AA** | CAGATCCCAGATGGATTTTTTGCCCATTTTTATGCCATTTGTGAACACATCAGCCCAGTCCTAGCCTGGGGCTTTCTGGGTCCTAGAAATTCTCTCTATGATTTATGTTGCTTCTTTAAG|  |  |  |  |  |  |  |  |  |  |  |  |  |  |  |  | | --- | --- | --- | --- | --- | --- | --- | --- | --- | --- | --- | --- | --- | --- | --- | --- | | Mouse mm9 chr3 151941857 151941976 - **AA** | CAGATCTCAGATGGATTTTTCGCTCATTTTTATGCCATTTGTGAGCATGTCAGCCCTGTTTTAGCCTGGGGCTTCTTGGGTCCTAGAAATTCTCTCTATGACTTATGTTGCTTCTTTAAG|  |  |  |  |  |  |  |  | | --- | --- | --- | --- | --- | --- | --- | --- | | Dog canFam2 chr6 72089743 72089862 - **AA** | CAGATCCCAGATGGATTTTTTGCCCATTTTTATGCCATTTGTGAGCAAATCAGCCCTGTCCTTGCCTGGGGCTTTTTGGGTCCTAGAAATTCTCTCTATGACTTATGTTGCTTCTTTAAG | | | | | | | | | | | | | | | | | | | | | | | | | | | | | | | | | | | | | | | | | | | | | | | | | | | | | | | | | | | | | | | | | | | | | | | | | | | | | |

**Alignment** (splice site sequences are in lowercase)  

```
Human      agCAGATCCCAGATGGATTTTTTGCCCATTTTTATGCCATTTGTGAACACATCAGTCCTGTCCTAGCCTGGGGCTTTTTG
Chimp      .a..............................................................................
Gorilla    .a..............................................................................
Orangutan  .a..............................................................................
Rhesus     .a..............................................................................
Baboon     .a..............................................................................
Marmoset   .a.....................................................C........................
Lemur      .a.....................................................C..A..................C..
Mouse      .a......T.............C..T....................G..TG....C.....TT.............C...
Dog        .a............................................G..A.....C........T...............

Human      GGTCCTAGAAATTCTCTGTATGATTTATGTTGCTTTTTTAAGgt
Chimp      ............................................
Gorilla    ............................................
Orangutan  .................C..........................
Rhesus     .................C..........................
Baboon     .................C..........................
Marmoset   .................C..........................
Lemur      .................C.................C........
Mouse      .................C.....C...........C........
Dog        .................C.....C...........C........
```

---

## 6. uc001flb.2\_9\_11

**Summary**  

|  |  |  |  |  |  |  |  |  |  |  |  |  |  |  |  |  |  |  |  |  |  |  |  |  |  |
| --- | --- | --- | --- | --- | --- | --- | --- | --- | --- | --- | --- | --- | --- | --- | --- | --- | --- | --- | --- | --- | --- | --- | --- | --- | --- |
| No Exon ID Position (hg19) Dir Human acceptor Chimp acceptor Category Usage Gene symbol Protein accession mRNA accession Gene title Note|  |  |  |  |  |  |  |  |  |  |  |  |  | | --- | --- | --- | --- | --- | --- | --- | --- | --- | --- | --- | --- | --- | | 6 uc001flb.2\_9\_11 chr1:155583216 + AG TG (A2) shift; increase; frameshift alternative MSTO1 Q9BUK6-7 AK056128.1 misato homolog 1  | | | | | | | | | | | | | | | | | | | | | | | | | |

**Orthologs**  

|  |  |  |  |  |  |  |  |  |  |  |  |  |  |  |  |  |  |  |  |  |  |  |  |  |  |  |  |  |  |  |  |  |  |  |  |  |  |  |  |  |  |  |  |  |  |  |  |  |  |  |  |  |  |  |  |  |  |  |  |  |  |  |  |
| --- | --- | --- | --- | --- | --- | --- | --- | --- | --- | --- | --- | --- | --- | --- | --- | --- | --- | --- | --- | --- | --- | --- | --- | --- | --- | --- | --- | --- | --- | --- | --- | --- | --- | --- | --- | --- | --- | --- | --- | --- | --- | --- | --- | --- | --- | --- | --- | --- | --- | --- | --- | --- | --- | --- | --- | --- | --- | --- | --- | --- | --- | --- | --- |
| Species Assembly Chromosome Exon start Exon end Dir Acceptor Exon sequence|  |  |  |  |  |  |  |  |  |  |  |  |  |  |  |  |  |  |  |  |  |  |  |  |  |  |  |  |  |  |  |  |  |  |  |  |  |  |  |  |  |  |  |  |  |  |  |  |  |  |  |  |  |  |  |  | | --- | --- | --- | --- | --- | --- | --- | --- | --- | --- | --- | --- | --- | --- | --- | --- | --- | --- | --- | --- | --- | --- | --- | --- | --- | --- | --- | --- | --- | --- | --- | --- | --- | --- | --- | --- | --- | --- | --- | --- | --- | --- | --- | --- | --- | --- | --- | --- | --- | --- | --- | --- | --- | --- | --- | --- | | Human hg19 chr1 155583216 155583357 + **AG** | CCACAGACTAATGGTGGTTTTGGCTTTGTTCTGGCAGCCAGCTCACCCCAGGGACACCTCCACCCTCTGCCCTTCATGCATGTACCACTGGGGAAGAAATCTTGGCTCAGTATTTACAACAGCAGCAGCCTGGAGTCATGAG|  |  |  |  |  |  |  |  |  |  |  |  |  |  |  |  |  |  |  |  |  |  |  |  |  |  |  |  |  |  |  |  |  |  |  |  |  |  |  |  |  |  |  |  |  |  |  |  | | --- | --- | --- | --- | --- | --- | --- | --- | --- | --- | --- | --- | --- | --- | --- | --- | --- | --- | --- | --- | --- | --- | --- | --- | --- | --- | --- | --- | --- | --- | --- | --- | --- | --- | --- | --- | --- | --- | --- | --- | --- | --- | --- | --- | --- | --- | --- | --- | | Chimp panTro2 chr1 134788762 134788903 + **TG** | CCACAGACTAATGGTGGTTTTGGCTTTGTTCTGGCAGTCAGCTCACCCCAGGGACACCTCCACCCTCTGCCCTTCATGCATGTACCACTGGGGAAGAAATCTTGGCTCAGTATTTACAACAGCAGCAGCCTGGAGTCATGAG|  |  |  |  |  |  |  |  |  |  |  |  |  |  |  |  |  |  |  |  |  |  |  |  |  |  |  |  |  |  |  |  |  |  |  |  |  |  |  |  | | --- | --- | --- | --- | --- | --- | --- | --- | --- | --- | --- | --- | --- | --- | --- | --- | --- | --- | --- | --- | --- | --- | --- | --- | --- | --- | --- | --- | --- | --- | --- | --- | --- | --- | --- | --- | --- | --- | --- | --- | | Orangutan ponAbe2 chr1 95768097 95768238 - **TG** | CCACAGACTAATGGTGGTTTTGGCTTTGTTCTGGCAGCCAGCTCACCCCAGGGACACCTCCACCCTCCGCCCTTCATGCATGTACCACTGGGGAAGAAGTCTTGGCTCAGTATTTACAACAGCAGCAGCCTGGAGTGATGAG|  |  |  |  |  |  |  |  |  |  |  |  |  |  |  |  |  |  |  |  |  |  |  |  |  |  |  |  |  |  |  |  | | --- | --- | --- | --- | --- | --- | --- | --- | --- | --- | --- | --- | --- | --- | --- | --- | --- | --- | --- | --- | --- | --- | --- | --- | --- | --- | --- | --- | --- | --- | --- | --- | | Rhesus rheMac2 chr1 134191728 134191869 + **TG** | CCACAGCCTAATGGTGGTTTTGGCTTTGTTCTGGCAGCCAGCTCACCCCAGGGACACCTCCACCCTCCTCCCTTCACGCATGTACCACTGGGGAAGAAGTCTTGGCTCAGTATTTACAACAGCAGCAGCCTAGAGTCATGAG|  |  |  |  |  |  |  |  |  |  |  |  |  |  |  |  |  |  |  |  |  |  |  |  | | --- | --- | --- | --- | --- | --- | --- | --- | --- | --- | --- | --- | --- | --- | --- | --- | --- | --- | --- | --- | --- | --- | --- | --- | | Mouse mm9 chr3 88714236 88714370 - **AG** | CCAAAGGTAAATGCGGTTTTTTCCTTGCAGTAAGCTCAACCCAGGGACACCTCTGCCCTCTGCTCTCCACGCATGCGCCTCTGGAGAAGAAGTCTTGGCCCAGTACTTACAGCAGCAGCATCCTAGAGTCTTGAG|  |  |  |  |  |  |  |  |  |  |  |  |  |  |  |  | | --- | --- | --- | --- | --- | --- | --- | --- | --- | --- | --- | --- | --- | --- | --- | --- | | Cow bosTau4 chr3 16379677 16379818 - **TG** | CCACTGATTAAAGGTGGTTTTGGCTTTGTTCTGGCAGTCAGCTCGCCCCCGGGACACCTCTGCCCTCCCCGCTCCATGCGTGCGCCACTGGGGAAGAAGTCTTGGCCCAGTATTTGCAGCAGCAGCAGCCTAGAGTCAGGAG|  |  |  |  |  |  |  |  | | --- | --- | --- | --- | --- | --- | --- | --- | | Dog canFam2 chr7 44974039 44974180 - **TG** | CCATGGACTTAAAGGCAGTTTTGGCTTTGTTCTGCAGTCAGCTCGTCCCGGGGACACCTCTGCCTTCGCCACTCCATGCCTGCACCGCCGGGGAGGATGTCCTGGCTCAGTATTTACAGCAGCAGCAGCCTCGGGTCAGGAG | | | | | | | | | | | | | | | | | | | | | | | | | | | | | | | | | | | | | | | | | | | | | | | | | | | | | | | | |

**Alignment** (splice site sequences are in lowercase)  

```
Human      agCCACAGAC-TAATGGTGGTTTTGGCTTTGTTCTGGCAGCCAGCTCACCCCAGGGACACCTCCACCCTCTGCCCTTCAT
Chimp      t.........-.............................T.......................................
Orangutan  t.........-...........................................................C.........
Rhesus     t.......C.-...........................................................CT.......C
Mouse      ...--------C..A...AAA.GC..T...T.C..T....TA......A..............TG........T..C..C
Cow        t.....T..T-...A.........................T......G....C..........TG.....CC.G..C...
Dog        t....TG...T...A..CA................-....T......GT...G..........TG..T..GC.A..C...

Human      GCATGTACCACTGGGGAAGAAATCTTGGCTCAGTATTTACAACAGCAGCAGCCTGGAGTCATGAGgt
Chimp      ...................................................................
Orangutan  .....................G.....................................G.......
Rhesus     .....................G................................A............
Mouse      .....CG..T....A......G.......C.....C.....G........T...A.....T......
Cow        ..G..CG..............G.......C........G..G............A......G.....
Dog        ..C..C...G.C.....G..TG..C................G............C.G....G.....
```

---

## 7. uc001gli.1\_6\_25

**Summary**  

|  |  |  |  |  |  |  |  |  |  |  |  |  |  |  |  |  |  |  |  |  |  |  |  |  |  |
| --- | --- | --- | --- | --- | --- | --- | --- | --- | --- | --- | --- | --- | --- | --- | --- | --- | --- | --- | --- | --- | --- | --- | --- | --- | --- |
| No Exon ID Position (hg19) Dir Human acceptor Chimp acceptor Category Usage Gene symbol Protein accession mRNA accession Gene title Note|  |  |  |  |  |  |  |  |  |  |  |  |  | | --- | --- | --- | --- | --- | --- | --- | --- | --- | --- | --- | --- | --- | | 7 uc001gli.1\_6\_25 chr1:177930074 - AG GG (A3) shift; decrease; inframe alternative SEC16B NP\_149118.2 NM\_033127.2 protein transport protein Sec16B NAGNAG | | | | | | | | | | | | | | | | | | | | | | | | | |

**Orthologs**  

|  |  |  |  |  |  |  |  |  |  |  |  |  |  |  |  |  |  |  |  |  |  |  |  |  |  |  |  |  |  |  |  |  |  |  |  |  |  |  |  |  |  |  |  |  |  |  |  |  |  |  |  |  |  |  |  |  |  |  |  |  |  |  |  |  |  |  |  |  |  |  |  |  |  |  |  |  |  |  |  |  |  |  |  |  |  |  |  |  |  |  |  |  |  |  |  |  |  |  |  |  |  |  |  |  |  |  |  |  |  |  |  |
| --- | --- | --- | --- | --- | --- | --- | --- | --- | --- | --- | --- | --- | --- | --- | --- | --- | --- | --- | --- | --- | --- | --- | --- | --- | --- | --- | --- | --- | --- | --- | --- | --- | --- | --- | --- | --- | --- | --- | --- | --- | --- | --- | --- | --- | --- | --- | --- | --- | --- | --- | --- | --- | --- | --- | --- | --- | --- | --- | --- | --- | --- | --- | --- | --- | --- | --- | --- | --- | --- | --- | --- | --- | --- | --- | --- | --- | --- | --- | --- | --- | --- | --- | --- | --- | --- | --- | --- | --- | --- | --- | --- | --- | --- | --- | --- | --- | --- | --- | --- | --- | --- | --- | --- | --- | --- | --- | --- | --- | --- | --- | --- |
| Species Assembly Chromosome Exon start Exon end Dir Acceptor Exon sequence|  |  |  |  |  |  |  |  |  |  |  |  |  |  |  |  |  |  |  |  |  |  |  |  |  |  |  |  |  |  |  |  |  |  |  |  |  |  |  |  |  |  |  |  |  |  |  |  |  |  |  |  |  |  |  |  |  |  |  |  |  |  |  |  |  |  |  |  |  |  |  |  |  |  |  |  |  |  |  |  |  |  |  |  |  |  |  |  |  |  |  |  |  |  |  |  |  |  |  |  |  |  |  |  | | --- | --- | --- | --- | --- | --- | --- | --- | --- | --- | --- | --- | --- | --- | --- | --- | --- | --- | --- | --- | --- | --- | --- | --- | --- | --- | --- | --- | --- | --- | --- | --- | --- | --- | --- | --- | --- | --- | --- | --- | --- | --- | --- | --- | --- | --- | --- | --- | --- | --- | --- | --- | --- | --- | --- | --- | --- | --- | --- | --- | --- | --- | --- | --- | --- | --- | --- | --- | --- | --- | --- | --- | --- | --- | --- | --- | --- | --- | --- | --- | --- | --- | --- | --- | --- | --- | --- | --- | --- | --- | --- | --- | --- | --- | --- | --- | --- | --- | --- | --- | --- | --- | --- | --- | | Human hg19 chr1 177929926 177930074 - **AG** | ATGTCTCCTCAGCTGGTCCCAAAGCACCCATGAAGTTCTACATCCCTCATGTTCCTGTGAGTTTCGGGCCAGGAGGTCAGCTGGTGCATGTAGGTCCCAGCTCTCCCACTGACGGGCAAGCAGCCCTTGTTGAACTGCACAGCATGGAG|  |  |  |  |  |  |  |  |  |  |  |  |  |  |  |  |  |  |  |  |  |  |  |  |  |  |  |  |  |  |  |  |  |  |  |  |  |  |  |  |  |  |  |  |  |  |  |  |  |  |  |  |  |  |  |  |  |  |  |  |  |  |  |  |  |  |  |  |  |  |  |  |  |  |  |  |  |  |  |  |  |  |  |  |  |  |  |  |  |  |  |  |  |  |  |  | | --- | --- | --- | --- | --- | --- | --- | --- | --- | --- | --- | --- | --- | --- | --- | --- | --- | --- | --- | --- | --- | --- | --- | --- | --- | --- | --- | --- | --- | --- | --- | --- | --- | --- | --- | --- | --- | --- | --- | --- | --- | --- | --- | --- | --- | --- | --- | --- | --- | --- | --- | --- | --- | --- | --- | --- | --- | --- | --- | --- | --- | --- | --- | --- | --- | --- | --- | --- | --- | --- | --- | --- | --- | --- | --- | --- | --- | --- | --- | --- | --- | --- | --- | --- | --- | --- | --- | --- | --- | --- | --- | --- | --- | --- | --- | --- | | Chimp panTro2 chr1 157548953 157549101 - **GG** | ATGTCTCCTCAGCTGGTCCCAAAGCACCCATGAAGTTCTACATCCCTCATGTTCCTGTGAGTTTCGGGCCAGGAGGTCAGCTGGTGCGTGTAGGTCCCAGCTCTCCCACTGACGGGCAAGCAGCCCTTGTTGAACTGCACAGCATGGAG|  |  |  |  |  |  |  |  |  |  |  |  |  |  |  |  |  |  |  |  |  |  |  |  |  |  |  |  |  |  |  |  |  |  |  |  |  |  |  |  |  |  |  |  |  |  |  |  |  |  |  |  |  |  |  |  |  |  |  |  |  |  |  |  |  |  |  |  |  |  |  |  |  |  |  |  |  |  |  |  |  |  |  |  |  |  |  |  | | --- | --- | --- | --- | --- | --- | --- | --- | --- | --- | --- | --- | --- | --- | --- | --- | --- | --- | --- | --- | --- | --- | --- | --- | --- | --- | --- | --- | --- | --- | --- | --- | --- | --- | --- | --- | --- | --- | --- | --- | --- | --- | --- | --- | --- | --- | --- | --- | --- | --- | --- | --- | --- | --- | --- | --- | --- | --- | --- | --- | --- | --- | --- | --- | --- | --- | --- | --- | --- | --- | --- | --- | --- | --- | --- | --- | --- | --- | --- | --- | --- | --- | --- | --- | --- | --- | --- | --- | | Gorilla gorGor1 Supercontig\_0597956 664 812 + **GG** | ATGTCTCCTCAGCTGGTCCCAAAGCACCCGTGAAGTTCTACATCCCTCATGTTCCTGTGAGTTTCGGGCCAGGAGGTCAGCTGGTGCGTGTGGGTCCNAGCTCTCCCACTGACGGGCAAGCAGCCCTTGTTGAACTGCACAGCATGGAG|  |  |  |  |  |  |  |  |  |  |  |  |  |  |  |  |  |  |  |  |  |  |  |  |  |  |  |  |  |  |  |  |  |  |  |  |  |  |  |  |  |  |  |  |  |  |  |  |  |  |  |  |  |  |  |  |  |  |  |  |  |  |  |  |  |  |  |  |  |  |  |  |  |  |  |  |  |  |  |  | | --- | --- | --- | --- | --- | --- | --- | --- | --- | --- | --- | --- | --- | --- | --- | --- | --- | --- | --- | --- | --- | --- | --- | --- | --- | --- | --- | --- | --- | --- | --- | --- | --- | --- | --- | --- | --- | --- | --- | --- | --- | --- | --- | --- | --- | --- | --- | --- | --- | --- | --- | --- | --- | --- | --- | --- | --- | --- | --- | --- | --- | --- | --- | --- | --- | --- | --- | --- | --- | --- | --- | --- | --- | --- | --- | --- | --- | --- | --- | --- | | Orangutan ponAbe2 chr1 72885805 72885953 + **GG** | ATGTCTCATCAGCTGGTCCCAAAGCACCCATGAAGTTCTACATCCCTCATGTTCCTGTGAGTTTCGGGCCAGGAGGTCAGCTGGTGCGTGTAGGTCCCAGCTTTCCCACTGACGGGCAAGCAGCCCTTGTTGAACTGCACAGCATGGAG|  |  |  |  |  |  |  |  |  |  |  |  |  |  |  |  |  |  |  |  |  |  |  |  |  |  |  |  |  |  |  |  |  |  |  |  |  |  |  |  |  |  |  |  |  |  |  |  |  |  |  |  |  |  |  |  |  |  |  |  |  |  |  |  |  |  |  |  |  |  |  |  | | --- | --- | --- | --- | --- | --- | --- | --- | --- | --- | --- | --- | --- | --- | --- | --- | --- | --- | --- | --- | --- | --- | --- | --- | --- | --- | --- | --- | --- | --- | --- | --- | --- | --- | --- | --- | --- | --- | --- | --- | --- | --- | --- | --- | --- | --- | --- | --- | --- | --- | --- | --- | --- | --- | --- | --- | --- | --- | --- | --- | --- | --- | --- | --- | --- | --- | --- | --- | --- | --- | --- | --- | | Rhesus rheMac2 chr1 207547781 207547929 - **GG** | ATGTCTCCTCAGCTGGTCCCAAAGCGCCCATGAAGTTCTACGTTCCTCATGCTCCTGTGAGTTTCGGGCCAGGAGGTCAGCTGGTGTGTGTAGGTCCCAGCTCTCCCACTGACGGGCAAGCAGCCCTTGTTGAACTGCACAGCATGGAG|  |  |  |  |  |  |  |  |  |  |  |  |  |  |  |  |  |  |  |  |  |  |  |  |  |  |  |  |  |  |  |  |  |  |  |  |  |  |  |  |  |  |  |  |  |  |  |  |  |  |  |  |  |  |  |  |  |  |  |  |  |  |  |  | | --- | --- | --- | --- | --- | --- | --- | --- | --- | --- | --- | --- | --- | --- | --- | --- | --- | --- | --- | --- | --- | --- | --- | --- | --- | --- | --- | --- | --- | --- | --- | --- | --- | --- | --- | --- | --- | --- | --- | --- | --- | --- | --- | --- | --- | --- | --- | --- | --- | --- | --- | --- | --- | --- | --- | --- | --- | --- | --- | --- | --- | --- | --- | --- | | Baboon papHam1 scaffold16654 27993 28141 + **GG** | ATGTCTCCTCAGCTGGTCCCAAAGCGCCCATGAAGTTCTACGTTCCTCATGTTCCTGTGAGTTTCGGGCCAGGAGGTCAGCTGGTGTGTGTAGGTCCCAGCTCTCCCACTGACGGGCAAGCAGCCCTTGTTGAACTGCACAGCATGGAG|  |  |  |  |  |  |  |  |  |  |  |  |  |  |  |  |  |  |  |  |  |  |  |  |  |  |  |  |  |  |  |  |  |  |  |  |  |  |  |  |  |  |  |  |  |  |  |  |  |  |  |  |  |  |  |  | | --- | --- | --- | --- | --- | --- | --- | --- | --- | --- | --- | --- | --- | --- | --- | --- | --- | --- | --- | --- | --- | --- | --- | --- | --- | --- | --- | --- | --- | --- | --- | --- | --- | --- | --- | --- | --- | --- | --- | --- | --- | --- | --- | --- | --- | --- | --- | --- | --- | --- | --- | --- | --- | --- | --- | --- | | Marmoset calJac1 Contig2688 176345 176493 + **GT** | ATGTCTCCTCAGCTGGTCCCAAAGCACCCATGAAGTTCTACGTCCCTCATGTTCCTGTGAATTTTGGGCCAGGAGGTCAGTTGGTGTGTGTGGGTCCCAGCTCTCTCACTGACGGGCAAGCAGCCCTCGTTGAAGTGCACAGCATGGAG|  |  |  |  |  |  |  |  |  |  |  |  |  |  |  |  |  |  |  |  |  |  |  |  |  |  |  |  |  |  |  |  |  |  |  |  |  |  |  |  |  |  |  |  |  |  |  |  | | --- | --- | --- | --- | --- | --- | --- | --- | --- | --- | --- | --- | --- | --- | --- | --- | --- | --- | --- | --- | --- | --- | --- | --- | --- | --- | --- | --- | --- | --- | --- | --- | --- | --- | --- | --- | --- | --- | --- | --- | --- | --- | --- | --- | --- | --- | --- | --- | | Tarsier tarSyr1 scaffold\_18420 16461 16609 + **GG** | ATGTCTCCACAGCTGGTTCCAAAGCACTTGTGAAGTTCTACAACCCTCACGTACCTGTGAGTTTTGGGCCAGGAGGTCAGCTGGTGTGTGTGGTGCCCAGTTCCCCCATGGATGGGCAGACAGCTCTTGTGGAACTGCACAGCATGGAA|  |  |  |  |  |  |  |  |  |  |  |  |  |  |  |  |  |  |  |  |  |  |  |  |  |  |  |  |  |  |  |  |  |  |  |  |  |  |  |  | | --- | --- | --- | --- | --- | --- | --- | --- | --- | --- | --- | --- | --- | --- | --- | --- | --- | --- | --- | --- | --- | --- | --- | --- | --- | --- | --- | --- | --- | --- | --- | --- | --- | --- | --- | --- | --- | --- | --- | --- | | Lemur micMur1 scaffold\_1609 147389 147537 + **GG** | ATGTCTCTGCAGCTGATCCCAAAGCACCCATGAAGTTCTATGGCCCTCATGTACCTGTGAGTTTTGGACCAGGAGGTCAGCTGGTGTGTGTGGCGCCCAGCTCGCCCACTGGTGGGCGAACAGCCCTTGTTGACCTGCACAGCGTGGAG|  |  |  |  |  |  |  |  |  |  |  |  |  |  |  |  |  |  |  |  |  |  |  |  |  |  |  |  |  |  |  |  | | --- | --- | --- | --- | --- | --- | --- | --- | --- | --- | --- | --- | --- | --- | --- | --- | --- | --- | --- | --- | --- | --- | --- | --- | --- | --- | --- | --- | --- | --- | --- | --- | | Galago otoGar1 scaffold\_15320.1-212087 194900 195048 + **GA** | ATATCTCCACAGCTGGTCCCAAAACACCCATGAAGTTCTATATCCCTCATGTACCTGTGAGTTTTGGACCTGGAGGTCAGCTGGTGTGTGTGGCTCCCAGCTCTTTTATTGATGAGCAATCTGCCCTTGTTGAACTGCACAGCATGGAG|  |  |  |  |  |  |  |  |  |  |  |  |  |  |  |  |  |  |  |  |  |  |  |  | | --- | --- | --- | --- | --- | --- | --- | --- | --- | --- | --- | --- | --- | --- | --- | --- | --- | --- | --- | --- | --- | --- | --- | --- | | Mouse mm9 chr1 159465495 159465643 + **TG** | ACACCTCAGCGACAGTTCCAAAGGCACCTATGAGGTTCTATGTTCCCCATGTGTCTGTGAGCTTTGGGCCAGGAGGCCAGCTAGTGTGTGTCCCCCCCAACTCTCCTGCTGATGGACAAACGGCCCTCGTTGAAGTGCACAGCATGGAG|  |  |  |  |  |  |  |  |  |  |  |  |  |  |  |  | | --- | --- | --- | --- | --- | --- | --- | --- | --- | --- | --- | --- | --- | --- | --- | --- | | Cow bosTau4 chr16 57155708 57155856 - **GG** | ACATCTCAGCAGCTGGTCCCAAGGAACCCATGAAGTTCTATGTCCCTCACATGCCTGTGAGTTTTGGGCCAGGAGGTCAGCTGGTGTGTGTGAGTCCCAGCTCTCCCAGTGATGGACAGACAGCCCTTGTTGAACTGCACAGCATGGAG|  |  |  |  |  |  |  |  | | --- | --- | --- | --- | --- | --- | --- | --- | | Dog canFam2 chr7 24676283 24676431 + **GA** | ATGTCTCGGCAGCAGGTCCTAAGGAGCCCATGAAATTCTACATCCCTCATGTGCCTGTGAGTTTTGGGCCAGGAGGTCAGCTGGTGTATGTGAGTCCCAGCTCCCCCAGAGATGGACAGACGGCCCTAGTTGAACTGCACAGCATGGAG | | | | | | | | | | | | | | | | | | | | | | | | | | | | | | | | | | | | | | | | | | | | | | | | | | | | | | | | | | | | | | | | | | | | | | | | | | | | | | | | | | | | | | | | | | | | | | | | | | |

**Alignment** (splice site sequences are in lowercase)  

```
Human      agATGTCTCCTCAGCTGGTCCCAAAGCACCCATGAAGTTCTACATCCCTCATGTTCCTGTGAGTTTCGGGCCAGGAGGTC
Chimp      g...............................................................................
Gorilla    g..............................G................................................
Orangutan  g........A......................................................................
Rhesus     g..........................G...............G.T.......C..........................
Baboon     g..........................G...............G.T..................................
Marmoset   gt.........................................G..................A...T.............
Tarsier    g.........A........T.........TTG............A......C..A...........T.............
Lemur      g........TG......A........................TGG.........A...........T..A..........
Galago     ga..A.....A..............A................T...........A...........T..A..T.......
Mouse      t..CAC...AG.GA.A.T...A..G.....T....G......TG.T..C.....GT.......C..T...........C.
Cow        g..CA....AG.............G.A...............TG.......CA.G...........T.............
Dog        ga.......GG....A.....T..G.AG........A.................G...........T.............

Human      AGCTGGTGCATGTAGGTCCCAGCTCTCCCACTGACGGGCAAGCAGCCCTTGTTGAACTGCACAGCATGGAGgt
Chimp      .........G...............................................................
Gorilla    .........G...G.....N.....................................................
Orangutan  .........G..............T................................................
Rhesus     ........TG...............................................................
Baboon     ........TG...............................................................
Marmoset   ..T.....TG...G.............T.....................C......G................
Tarsier    ........TG...G.TG.....T..C....TG..T.....GA....T.....G.................A..
Lemur      ........TG...G.CG........G.......GT....G.A.............C.........G.......
Galago     ........TG...G.C..........TTT.T...T.A....T.T.............................
Mouse      ....A...TG...CCCC....A......TG....T..A...A.G.....C......G................
Cow        ........TG...GA...............G...T..A..GA...............................
Dog        ........T....GA..........C....GA..T..A..GA.G.....A.......................
```

---

## 8. uc001how.2\_33\_83

**Summary**  

|  |  |  |  |  |  |  |  |  |  |  |  |  |  |  |  |  |  |  |  |  |  |  |  |  |  |
| --- | --- | --- | --- | --- | --- | --- | --- | --- | --- | --- | --- | --- | --- | --- | --- | --- | --- | --- | --- | --- | --- | --- | --- | --- | --- |
| No Exon ID Position (hg19) Dir Human acceptor Chimp acceptor Category Usage Gene symbol Protein accession mRNA accession Gene title Note|  |  |  |  |  |  |  |  |  |  |  |  |  | | --- | --- | --- | --- | --- | --- | --- | --- | --- | --- | --- | --- | --- | | 8 uc001how.2\_33\_83 chr1:225347031 + AG AA (A3) shift; decrease; inframe alternative DNAH14 NP\_001364.1 NM\_001373.1 dynein heavy chain 14, axonemal  | | | | | | | | | | | | | | | | | | | | | | | | | |

**Orthologs**  

|  |  |  |  |  |  |  |  |  |  |  |  |  |  |  |  |  |  |  |  |  |  |  |  |  |  |  |  |  |  |  |  |  |  |  |  |  |  |  |  |  |  |  |  |  |  |  |  |  |  |  |  |  |  |  |  |  |  |  |  |  |  |  |  |  |  |  |  |  |  |  |  |  |  |  |  |  |  |  |  |
| --- | --- | --- | --- | --- | --- | --- | --- | --- | --- | --- | --- | --- | --- | --- | --- | --- | --- | --- | --- | --- | --- | --- | --- | --- | --- | --- | --- | --- | --- | --- | --- | --- | --- | --- | --- | --- | --- | --- | --- | --- | --- | --- | --- | --- | --- | --- | --- | --- | --- | --- | --- | --- | --- | --- | --- | --- | --- | --- | --- | --- | --- | --- | --- | --- | --- | --- | --- | --- | --- | --- | --- | --- | --- | --- | --- | --- | --- | --- | --- |
| Species Assembly Chromosome Exon start Exon end Dir Acceptor Exon sequence|  |  |  |  |  |  |  |  |  |  |  |  |  |  |  |  |  |  |  |  |  |  |  |  |  |  |  |  |  |  |  |  |  |  |  |  |  |  |  |  |  |  |  |  |  |  |  |  |  |  |  |  |  |  |  |  |  |  |  |  |  |  |  |  |  |  |  |  |  |  |  |  | | --- | --- | --- | --- | --- | --- | --- | --- | --- | --- | --- | --- | --- | --- | --- | --- | --- | --- | --- | --- | --- | --- | --- | --- | --- | --- | --- | --- | --- | --- | --- | --- | --- | --- | --- | --- | --- | --- | --- | --- | --- | --- | --- | --- | --- | --- | --- | --- | --- | --- | --- | --- | --- | --- | --- | --- | --- | --- | --- | --- | --- | --- | --- | --- | --- | --- | --- | --- | --- | --- | --- | --- | | Human hg19 chr1 225347031 225347187 + **AG** | TCTTTCTGAAGCAGATGAAACCTTGATTGTTATCGAGGCTATAAGAGAAGCTAGTTTGCCAAAATGTCCTCCTGAAGATGTCCCACTTTTTGAAAATATTATAGGAGATATTTTTCCAGAAGTGACAGTTTTGAAAGTAAATCAACTTGCCTTGGAG|  |  |  |  |  |  |  |  |  |  |  |  |  |  |  |  |  |  |  |  |  |  |  |  |  |  |  |  |  |  |  |  |  |  |  |  |  |  |  |  |  |  |  |  |  |  |  |  |  |  |  |  |  |  |  |  |  |  |  |  |  |  |  |  | | --- | --- | --- | --- | --- | --- | --- | --- | --- | --- | --- | --- | --- | --- | --- | --- | --- | --- | --- | --- | --- | --- | --- | --- | --- | --- | --- | --- | --- | --- | --- | --- | --- | --- | --- | --- | --- | --- | --- | --- | --- | --- | --- | --- | --- | --- | --- | --- | --- | --- | --- | --- | --- | --- | --- | --- | --- | --- | --- | --- | --- | --- | --- | --- | | Chimp panTro2 chr1 205772224 205772380 + **AA** | TCTTTCTGAAGCAGATGAAACCTTGATTGTTGTCGAGGCTATAAGAGAAGCTAGTTTGCCAAAATGTCCTCCTGAAGATGTCCCGCTTTTTGAAAATATTATAGGAGATATTTTTCCAGAAGTGACAGTTTTGAAAGTAAATCAACTTGCCTTGGAG|  |  |  |  |  |  |  |  |  |  |  |  |  |  |  |  |  |  |  |  |  |  |  |  |  |  |  |  |  |  |  |  |  |  |  |  |  |  |  |  |  |  |  |  |  |  |  |  |  |  |  |  |  |  |  |  | | --- | --- | --- | --- | --- | --- | --- | --- | --- | --- | --- | --- | --- | --- | --- | --- | --- | --- | --- | --- | --- | --- | --- | --- | --- | --- | --- | --- | --- | --- | --- | --- | --- | --- | --- | --- | --- | --- | --- | --- | --- | --- | --- | --- | --- | --- | --- | --- | --- | --- | --- | --- | --- | --- | --- | --- | | Orangutan ponAbe2 chr1 24572514 24572670 - **AA** | TCTTTCTGAAGCAAATGAAACCTTGATTGTTATCGAGGCTATAAGAGAAGCTAGTTTGCCAAAATGTCCTCCTGAAGATGTCCCACTTTTTGAAAATATTATAGGAGATATTTTTCCAGAAGTGACAGTTTTAAAAGTAAATCAACTTGCCTTGGAG|  |  |  |  |  |  |  |  |  |  |  |  |  |  |  |  |  |  |  |  |  |  |  |  |  |  |  |  |  |  |  |  |  |  |  |  |  |  |  |  |  |  |  |  |  |  |  |  | | --- | --- | --- | --- | --- | --- | --- | --- | --- | --- | --- | --- | --- | --- | --- | --- | --- | --- | --- | --- | --- | --- | --- | --- | --- | --- | --- | --- | --- | --- | --- | --- | --- | --- | --- | --- | --- | --- | --- | --- | --- | --- | --- | --- | --- | --- | --- | --- | | Rhesus rheMac2 chr1 145291465 145291621 - **AA** | TCTTTCTGAAGCAGATGAAACCTTGATTGTTATCGAGGCTATAAGAGAAGCTAGTTTGCCAAAATGTCCTCCTGAAGATGTCCCACTTTTTGAAAATATTATAGGAGATATTTTTCCAGAAGTGACAGTTTTAAAAGTAAATCAACTTGCCTTGGAG|  |  |  |  |  |  |  |  |  |  |  |  |  |  |  |  |  |  |  |  |  |  |  |  |  |  |  |  |  |  |  |  |  |  |  |  |  |  |  |  | | --- | --- | --- | --- | --- | --- | --- | --- | --- | --- | --- | --- | --- | --- | --- | --- | --- | --- | --- | --- | --- | --- | --- | --- | --- | --- | --- | --- | --- | --- | --- | --- | --- | --- | --- | --- | --- | --- | --- | --- | | Baboon papHam1 scaffold38439 3649 3805 - **AA** | TCTTTCTGAAGCAGATGAAACCTTGATTGTTATCGAGGCTATAAGAGAAGCTAGTTTGCCAAAATGTCCTCCTGAAGATGTTCCACTTTTTGAAAATATTATAGGAGATATTTTTCCAGAAGTGACAGTTTTAAAAGTAAATCAACTTGCCTTGGAG|  |  |  |  |  |  |  |  |  |  |  |  |  |  |  |  |  |  |  |  |  |  |  |  |  |  |  |  |  |  |  |  | | --- | --- | --- | --- | --- | --- | --- | --- | --- | --- | --- | --- | --- | --- | --- | --- | --- | --- | --- | --- | --- | --- | --- | --- | --- | --- | --- | --- | --- | --- | --- | --- | | Tarsier tarSyr1 scaffold\_224456 1768 1926 + **AA** | TCTTGCTGAAGCAGATGAAACTCTGATCATTATTGAGGCTATAAGAGAAGCTAATTTACCAAAATGTCCTCCTGAAGATGTCCCGCTTTTTGAAAATATTATAGGAGGTATATTTCCAGGAGTGTCAGTTTTAAAAGTAAATCAACTTGCCCTTGGAAG|  |  |  |  |  |  |  |  |  |  |  |  |  |  |  |  |  |  |  |  |  |  |  |  | | --- | --- | --- | --- | --- | --- | --- | --- | --- | --- | --- | --- | --- | --- | --- | --- | --- | --- | --- | --- | --- | --- | --- | --- | | Galago otoGar1 scaffold\_15462.1-40686 5626 5782 - **AA** | TCTTTCTGAAGCAGAGGAAACGCTGATCATTATTGAGGCTGTAAGAGAAGCTAGTTTGCCAAAATGTCCTCCTGAAGATGTTCCACTTTTTGAAAGGATTATAGGAGATATTTTTCCTGGAGTGACAGTTTTAAAAGCAAATCAACTTGCATTGGAG|  |  |  |  |  |  |  |  |  |  |  |  |  |  |  |  | | --- | --- | --- | --- | --- | --- | --- | --- | --- | --- | --- | --- | --- | --- | --- | --- | | Mouse mm9 chr1 183596490 183596646 + **CA** | CCTCTCTGAGTTAGACGAGTCCCTCATCCTCATCGAGGCCATCCGAGAAGCCAGTTTGTCCAAGCTCCTTCCGGAAGATGTCCTACCTTTTGAAAAGATCATAGAAGATGTTTTTCCTAGAATAACGGTTTCAAAAATAAATCACCTTACCTTGGAG|  |  |  |  |  |  |  |  | | --- | --- | --- | --- | --- | --- | --- | --- | | Dog canFam2 chr7 42456886 42457042 - **CA** | TCTTTCTGAAACAGATGAGACTCTGATCATTATTGAGGCTATAAGAGAAGCTAGTCTGCCAAAATTTCTCCCTAAAGATGCCCCACATTTTGAAAAGATTATAAGAGATTTTTTTCCTGGAGCAACATTTTCAACAGCAAATCAAATTGCCTTGGAG | | | | | | | | | | | | | | | | | | | | | | | | | | | | | | | | | | | | | | | | | | | | | | | | | | | | | | | | | | | | | | | | | | | | | | |

**Alignment** (splice site sequences are in lowercase)  

```
Human      agTCTTTCTGAAGCAGATGAAACCTTGATTGTTATCGAGGCTATAAGAGAAGCTAGTTTGCCAAAATGTCCTCCTGAAGA
Chimp      .a...............................G..............................................
Orangutan  .a.............A................................................................
Rhesus     .a..............................................................................
Baboon     .a..............................................................................
Tarsier    .a....G................TC....CA....T...................A...A....................
Galago     .a...............G.....GC....CA....T......G.....................................
Mouse      caC..C.....GTT...C..GT..C.C..CC.C........C..CC.......C......T.C..GCTC.T...G.....
Dog        ca..........A.......G..TC....CA....T.....................C.........T..TC...A....

Human      TGTCCCACTTTTTGAAAATATTATAGGAGATATTTTTCCAGAAGTGACAGTTTTGAAAGTAAATCAACTTGCCTT--GGA
Chimp      ......G....................................................................--...
Orangutan  ......................................................A....................--...
Rhesus     ......................................................A....................--...
Baboon     ...T..................................................A....................--...
Tarsier    ......G......................G...A.......G....T.......A..................C.TG.A.
Galago     ...T.............GG....................T.G............A....C............A..--...
Mouse      .....T..C.........G..C....A....G.......TAG.A.A..G....CA...A.......C...A....--...
Dog        ..C.....A.........G......A.....T.......T.G..CA...T...CA.C..C.......A.......--...

Human      Ggt
Chimp      ...
Orangutan  ...
Rhesus     ...
Baboon     ...
Tarsier    ...
Galago     ...
Mouse      ...
Dog        ...
```

---

## 9. uc002rin.2\_3\_8

**Summary**  

|  |  |  |  |  |  |  |  |  |  |  |  |  |  |  |  |  |  |  |  |  |  |  |  |  |  |
| --- | --- | --- | --- | --- | --- | --- | --- | --- | --- | --- | --- | --- | --- | --- | --- | --- | --- | --- | --- | --- | --- | --- | --- | --- | --- |
| No Exon ID Position (hg19) Dir Human acceptor Chimp acceptor Category Usage Gene symbol Protein accession mRNA accession Gene title Note|  |  |  |  |  |  |  |  |  |  |  |  |  | | --- | --- | --- | --- | --- | --- | --- | --- | --- | --- | --- | --- | --- | | 9 uc002rin.2\_3\_8 chr2:27317342 + AG GG (A1) shift; increase; inframe alternative KHK P50053-2 BX427548.2 ketohexokinase NAGNAG, dbSNP:rs74537742 | | | | | | | | | | | | | | | | | | | | | | | | | |

**Orthologs**  

|  |  |  |  |  |  |  |  |  |  |  |  |  |  |  |  |  |  |  |  |  |  |  |  |  |  |  |  |  |  |  |  |  |  |  |  |  |  |  |  |  |  |  |  |  |  |  |  |  |  |  |  |  |  |  |  |  |  |  |  |  |  |  |  |  |  |  |  |  |  |  |  |  |  |  |  |  |  |  |  |  |  |  |  |  |  |  |  |  |  |  |  |  |  |  |  |  |  |  |  |  |  |  |  |
| --- | --- | --- | --- | --- | --- | --- | --- | --- | --- | --- | --- | --- | --- | --- | --- | --- | --- | --- | --- | --- | --- | --- | --- | --- | --- | --- | --- | --- | --- | --- | --- | --- | --- | --- | --- | --- | --- | --- | --- | --- | --- | --- | --- | --- | --- | --- | --- | --- | --- | --- | --- | --- | --- | --- | --- | --- | --- | --- | --- | --- | --- | --- | --- | --- | --- | --- | --- | --- | --- | --- | --- | --- | --- | --- | --- | --- | --- | --- | --- | --- | --- | --- | --- | --- | --- | --- | --- | --- | --- | --- | --- | --- | --- | --- | --- | --- | --- | --- | --- | --- | --- | --- | --- |
| Species Assembly Chromosome Exon start Exon end Dir Acceptor Exon sequence|  |  |  |  |  |  |  |  |  |  |  |  |  |  |  |  |  |  |  |  |  |  |  |  |  |  |  |  |  |  |  |  |  |  |  |  |  |  |  |  |  |  |  |  |  |  |  |  |  |  |  |  |  |  |  |  |  |  |  |  |  |  |  |  |  |  |  |  |  |  |  |  |  |  |  |  |  |  |  |  |  |  |  |  |  |  |  |  |  |  |  |  |  |  |  |  | | --- | --- | --- | --- | --- | --- | --- | --- | --- | --- | --- | --- | --- | --- | --- | --- | --- | --- | --- | --- | --- | --- | --- | --- | --- | --- | --- | --- | --- | --- | --- | --- | --- | --- | --- | --- | --- | --- | --- | --- | --- | --- | --- | --- | --- | --- | --- | --- | --- | --- | --- | --- | --- | --- | --- | --- | --- | --- | --- | --- | --- | --- | --- | --- | --- | --- | --- | --- | --- | --- | --- | --- | --- | --- | --- | --- | --- | --- | --- | --- | --- | --- | --- | --- | --- | --- | --- | --- | --- | --- | --- | --- | --- | --- | --- | --- | | Human hg19 chr2 27317342 27317479 + **AG** | CAGTTTTGTCCTGGATGACCTCCGCCGCTATTCTGTGGACCTACGCTACACAGTCTTTCAGACCACAGGCTCCGTCCCCATCGCCACGGTCATCATCAACGAGGCCAGTGGTAGCCGCACCATCCTATACTATGACAG|  |  |  |  |  |  |  |  |  |  |  |  |  |  |  |  |  |  |  |  |  |  |  |  |  |  |  |  |  |  |  |  |  |  |  |  |  |  |  |  |  |  |  |  |  |  |  |  |  |  |  |  |  |  |  |  |  |  |  |  |  |  |  |  |  |  |  |  |  |  |  |  |  |  |  |  |  |  |  |  |  |  |  |  |  |  |  |  | | --- | --- | --- | --- | --- | --- | --- | --- | --- | --- | --- | --- | --- | --- | --- | --- | --- | --- | --- | --- | --- | --- | --- | --- | --- | --- | --- | --- | --- | --- | --- | --- | --- | --- | --- | --- | --- | --- | --- | --- | --- | --- | --- | --- | --- | --- | --- | --- | --- | --- | --- | --- | --- | --- | --- | --- | --- | --- | --- | --- | --- | --- | --- | --- | --- | --- | --- | --- | --- | --- | --- | --- | --- | --- | --- | --- | --- | --- | --- | --- | --- | --- | --- | --- | --- | --- | --- | --- | | Chimp panTro2 chr2a 27685036 27685173 + **GG** | CAGTTTTGTCCTGGATGACCTCCGCCGCTATTCTGTGGACCTACGCTACACAGTCTTTCAGACCACAGGCTCCGTCCCCATCGCCACGGTCATCATCAACGAGGCCAGTGGTAGCCGCACCATCCTATACTATGACAG|  |  |  |  |  |  |  |  |  |  |  |  |  |  |  |  |  |  |  |  |  |  |  |  |  |  |  |  |  |  |  |  |  |  |  |  |  |  |  |  |  |  |  |  |  |  |  |  |  |  |  |  |  |  |  |  |  |  |  |  |  |  |  |  |  |  |  |  |  |  |  |  |  |  |  |  |  |  |  |  | | --- | --- | --- | --- | --- | --- | --- | --- | --- | --- | --- | --- | --- | --- | --- | --- | --- | --- | --- | --- | --- | --- | --- | --- | --- | --- | --- | --- | --- | --- | --- | --- | --- | --- | --- | --- | --- | --- | --- | --- | --- | --- | --- | --- | --- | --- | --- | --- | --- | --- | --- | --- | --- | --- | --- | --- | --- | --- | --- | --- | --- | --- | --- | --- | --- | --- | --- | --- | --- | --- | --- | --- | --- | --- | --- | --- | --- | --- | --- | --- | | Gorilla gorGor1 Supercontig\_0000008 34173 34310 + **GG** | CAGTTTTGTCCTGGATGACCTCCGCCGCTATTCTGTGGACCTACGCTACATGGTCTTTCAGACCACAGGCTCCGTCCCCATCGCCACAGTCATCATCAACGAGGCCAGTGGTAGCCGCACCATCCTATACTATGACAG|  |  |  |  |  |  |  |  |  |  |  |  |  |  |  |  |  |  |  |  |  |  |  |  |  |  |  |  |  |  |  |  |  |  |  |  |  |  |  |  |  |  |  |  |  |  |  |  |  |  |  |  |  |  |  |  |  |  |  |  |  |  |  |  |  |  |  |  |  |  |  |  | | --- | --- | --- | --- | --- | --- | --- | --- | --- | --- | --- | --- | --- | --- | --- | --- | --- | --- | --- | --- | --- | --- | --- | --- | --- | --- | --- | --- | --- | --- | --- | --- | --- | --- | --- | --- | --- | --- | --- | --- | --- | --- | --- | --- | --- | --- | --- | --- | --- | --- | --- | --- | --- | --- | --- | --- | --- | --- | --- | --- | --- | --- | --- | --- | --- | --- | --- | --- | --- | --- | --- | --- | | Orangutan ponAbe2 chr2a 85042113 85042250 - **GG** | CAGTTTTGTCCTGGATGACCTCCGCCGCTATTCTGTGGACCTACGCTACACGGTCTTTCAGACCACAGGCTCCGTCCCCATCGCCACGGTCATCATCAACGAGGCCAGTGGTAGCCGCACCATCCTATACTATGACAG|  |  |  |  |  |  |  |  |  |  |  |  |  |  |  |  |  |  |  |  |  |  |  |  |  |  |  |  |  |  |  |  |  |  |  |  |  |  |  |  |  |  |  |  |  |  |  |  |  |  |  |  |  |  |  |  |  |  |  |  |  |  |  |  | | --- | --- | --- | --- | --- | --- | --- | --- | --- | --- | --- | --- | --- | --- | --- | --- | --- | --- | --- | --- | --- | --- | --- | --- | --- | --- | --- | --- | --- | --- | --- | --- | --- | --- | --- | --- | --- | --- | --- | --- | --- | --- | --- | --- | --- | --- | --- | --- | --- | --- | --- | --- | --- | --- | --- | --- | --- | --- | --- | --- | --- | --- | --- | --- | | Rhesus rheMac2 chr13 27047891 27048028 + **GG** | CAGTTTTGTCCTGGATGACCTCCGCCGCTATTCTGTGGACCTACGCTACACGGTCTTTCAGACCACGGGCTCCGTCCCCATCGCCACGGTCATCATCAACGAGGCCAGTGGTAGCCGCACCATCCTATACTACGACAG|  |  |  |  |  |  |  |  |  |  |  |  |  |  |  |  |  |  |  |  |  |  |  |  |  |  |  |  |  |  |  |  |  |  |  |  |  |  |  |  |  |  |  |  |  |  |  |  |  |  |  |  |  |  |  |  | | --- | --- | --- | --- | --- | --- | --- | --- | --- | --- | --- | --- | --- | --- | --- | --- | --- | --- | --- | --- | --- | --- | --- | --- | --- | --- | --- | --- | --- | --- | --- | --- | --- | --- | --- | --- | --- | --- | --- | --- | --- | --- | --- | --- | --- | --- | --- | --- | --- | --- | --- | --- | --- | --- | --- | --- | | Baboon papHam1 scaffold2792 115698 115835 + **GG** | CAGTTTTGTCCTGGATGACCTCCGCCGCTATTCTGTGGACCTACGCTACACGGTCTTTCAGACCACGGGCTCCGTCCCCATCGCCACGGTCATCATCAACGAGGCCAGTGGTAGCCGCACCATCCTATACTACGACAG|  |  |  |  |  |  |  |  |  |  |  |  |  |  |  |  |  |  |  |  |  |  |  |  |  |  |  |  |  |  |  |  |  |  |  |  |  |  |  |  |  |  |  |  |  |  |  |  | | --- | --- | --- | --- | --- | --- | --- | --- | --- | --- | --- | --- | --- | --- | --- | --- | --- | --- | --- | --- | --- | --- | --- | --- | --- | --- | --- | --- | --- | --- | --- | --- | --- | --- | --- | --- | --- | --- | --- | --- | --- | --- | --- | --- | --- | --- | --- | --- | | Marmoset calJac1 Contig652 515297 515434 + **GG** | CAGTTTTGTCCTGGATGACCTCCGCCGCTATTCTGTGGACCTCCGCTACATGGTCTTTCAGACCACAGGCTCCGTCCCCATCGCCACAGTGATCATCAACGAGGCCAGTGGTAGCCGCACCATCCTATATTATGACAG|  |  |  |  |  |  |  |  |  |  |  |  |  |  |  |  |  |  |  |  |  |  |  |  |  |  |  |  |  |  |  |  |  |  |  |  |  |  |  |  | | --- | --- | --- | --- | --- | --- | --- | --- | --- | --- | --- | --- | --- | --- | --- | --- | --- | --- | --- | --- | --- | --- | --- | --- | --- | --- | --- | --- | --- | --- | --- | --- | --- | --- | --- | --- | --- | --- | --- | --- | | Lemur micMur1 scaffold\_1117 236307 236444 + **GG** | CAGTTTTGTCCTGGATGACCTCCGTCGCTATTCTGTGGACCTACGCTACACAGTCTTTCAGACCACAGGCTCCGTCCCCATTGCCACGGTCATCATCAACGAGGCCAGTGGTAGCCGCACCATCCTATACTCCAACAG|  |  |  |  |  |  |  |  |  |  |  |  |  |  |  |  |  |  |  |  |  |  |  |  |  |  |  |  |  |  |  |  | | --- | --- | --- | --- | --- | --- | --- | --- | --- | --- | --- | --- | --- | --- | --- | --- | --- | --- | --- | --- | --- | --- | --- | --- | --- | --- | --- | --- | --- | --- | --- | --- | | Galago otoGar1 scaffold\_93321.1-65426 3319 3456 + **GG** | CAGTTTTGTCCTGGATGACCTCCGTCGCTATTCTGTGGACCTACGCTACACAGTCTTTCAGACCATAGGCTCCGTTCACATCGCCACAGTCATCATCAACGAGGCCAGTGGTAGTCGCACCATCCTATACTCTGACAG|  |  |  |  |  |  |  |  |  |  |  |  |  |  |  |  |  |  |  |  |  |  |  |  | | --- | --- | --- | --- | --- | --- | --- | --- | --- | --- | --- | --- | --- | --- | --- | --- | --- | --- | --- | --- | --- | --- | --- | --- | | Mouse mm9 chr5 31229058 31229195 + **GG** | CAGTTTTGTCCTGGATGACCTCCGCCAACATTCTGTGGACTTACGATATGTGGTCCTTCAGACCGAGGGCTCCATCCCCACTTCTACAGTCATCATCAACGAGGCCAGCGGCAGCCGCACCATTCTGCACGCCTACAG|  |  |  |  |  |  |  |  |  |  |  |  |  |  |  |  | | --- | --- | --- | --- | --- | --- | --- | --- | --- | --- | --- | --- | --- | --- | --- | --- | | Cow bosTau4 chr11 74632728 74632865 - **GG** | CAGTTTTGTCCTGGACGACCTTCGCCGCTATTCTGTGGACCTCCGCTACATGGTCTTTCAGACCACGGGCTCCGTCCCCATCTCCACAGTCATCATCAGCGAGGCCAGCGGTAGCCGCACCATCCTACATGCCTACAG|  |  |  |  |  |  |  |  | | --- | --- | --- | --- | --- | --- | --- | --- | | Dog canFam2 chr17 24067520 24067657 + **GG** | CAGTTTTGTCCTGGATGACTTCCGCCGCTATTCCGTGGACCTCCACTACACAGTCTTTCAGACCACGGGCTCTGTCCCCATCTCCACGGTCATCATCAACAAGGCCAGTGGTAGCCGCACCATCCTACATGCCTACAG | | | | | | | | | | | | | | | | | | | | | | | | | | | | | | | | | | | | | | | | | | | | | | | | | | | | | | | | | | | | | | | | | | | | | | | | | | | | | | | | | | | | | | | | | | | |

**Alignment** (splice site sequences are in lowercase)  

```
Human      agCAGTTTTGTCCTGGATGACCTCCGCCGCTATTCTGTGGACCTACGCTACACAGTCTTTCAGACCACAGGCTCCGTCCC
Chimp      g...............................................................................
Gorilla    g...................................................TG..........................
Orangutan  g....................................................G..........................
Rhesus     g....................................................G..............G...........
Baboon     g....................................................G..............G...........
Marmoset   g...........................................C.......TG..........................
Lemur      g.........................T.....................................................
Galago     g.........................T........................................T.........T.A
Mouse      g...........................AAC...........T....A..TGTG...C........GAG......A....
Cow        g................C.....T....................C.......TG..............G...........
Dog        g....................T.............C........C.A.....................G.....T.....

Human      CATCGCCACGGTCATCATCAACGAGGCCAGTGGTAGCCGCACCATCCTATACTATGACAGgt
Chimp      ..............................................................
Gorilla    .........A....................................................
Orangutan  ..............................................................
Rhesus     ......................................................C.......
Baboon     ......................................................C.......
Marmoset   .........A..G......................................T..........
Lemur      ...T.................................................CCA......
Galago     .........A..........................T................C........
Mouse      ..CTT.T..A....................C..C...........T..GC..GCCT......
Cow        ....T....A..........G.........C..................C.TGCCT......
Dog        ....T.................A..........................C.TGCCT......
```

---

## 10. uc002rtl.2\_7\_13

**Summary**  

|  |  |  |  |  |  |  |  |  |  |  |  |  |  |  |  |  |  |  |  |  |  |  |  |  |  |
| --- | --- | --- | --- | --- | --- | --- | --- | --- | --- | --- | --- | --- | --- | --- | --- | --- | --- | --- | --- | --- | --- | --- | --- | --- | --- |
| No Exon ID Position (hg19) Dir Human acceptor Chimp acceptor Category Usage Gene symbol Protein accession mRNA accession Gene title Note|  |  |  |  |  |  |  |  |  |  |  |  |  | | --- | --- | --- | --- | --- | --- | --- | --- | --- | --- | --- | --- | --- | | 10 uc002rtl.2\_7\_13 chr2:44023026 + AG AA (A1) shift; increase; inframe alternative DYNC2LI1 NP\_001180393.1 NM\_001193464.1 cytoplasmic dynein 2 light intermediate chain 1 NAGNAG | | | | | | | | | | | | | | | | | | | | | | | | | |

**Orthologs**  

|  |  |  |  |  |  |  |  |  |  |  |  |  |  |  |  |  |  |  |  |  |  |  |  |  |  |  |  |  |  |  |  |  |  |  |  |  |  |  |  |  |  |  |  |  |  |  |  |  |  |  |  |  |  |  |  |  |  |  |  |  |  |  |  |  |  |  |  |  |  |  |  |  |  |  |  |  |  |  |  |  |  |  |  |  |  |  |  |  |  |  |  |  |  |  |  |  |  |  |  |  |  |  |  |
| --- | --- | --- | --- | --- | --- | --- | --- | --- | --- | --- | --- | --- | --- | --- | --- | --- | --- | --- | --- | --- | --- | --- | --- | --- | --- | --- | --- | --- | --- | --- | --- | --- | --- | --- | --- | --- | --- | --- | --- | --- | --- | --- | --- | --- | --- | --- | --- | --- | --- | --- | --- | --- | --- | --- | --- | --- | --- | --- | --- | --- | --- | --- | --- | --- | --- | --- | --- | --- | --- | --- | --- | --- | --- | --- | --- | --- | --- | --- | --- | --- | --- | --- | --- | --- | --- | --- | --- | --- | --- | --- | --- | --- | --- | --- | --- | --- | --- | --- | --- | --- | --- | --- | --- |
| Species Assembly Chromosome Exon start Exon end Dir Acceptor Exon sequence|  |  |  |  |  |  |  |  |  |  |  |  |  |  |  |  |  |  |  |  |  |  |  |  |  |  |  |  |  |  |  |  |  |  |  |  |  |  |  |  |  |  |  |  |  |  |  |  |  |  |  |  |  |  |  |  |  |  |  |  |  |  |  |  |  |  |  |  |  |  |  |  |  |  |  |  |  |  |  |  |  |  |  |  |  |  |  |  |  |  |  |  |  |  |  |  | | --- | --- | --- | --- | --- | --- | --- | --- | --- | --- | --- | --- | --- | --- | --- | --- | --- | --- | --- | --- | --- | --- | --- | --- | --- | --- | --- | --- | --- | --- | --- | --- | --- | --- | --- | --- | --- | --- | --- | --- | --- | --- | --- | --- | --- | --- | --- | --- | --- | --- | --- | --- | --- | --- | --- | --- | --- | --- | --- | --- | --- | --- | --- | --- | --- | --- | --- | --- | --- | --- | --- | --- | --- | --- | --- | --- | --- | --- | --- | --- | --- | --- | --- | --- | --- | --- | --- | --- | --- | --- | --- | --- | --- | --- | --- | --- | | Human hg19 chr2 44023026 44023097 + **AG** | CAGGATCATGAATTAATTGACCCATTTCCGGTACCTCTGGTCATAATTGGAAGTAAATATGATGTTTTTCAG|  |  |  |  |  |  |  |  |  |  |  |  |  |  |  |  |  |  |  |  |  |  |  |  |  |  |  |  |  |  |  |  |  |  |  |  |  |  |  |  |  |  |  |  |  |  |  |  |  |  |  |  |  |  |  |  |  |  |  |  |  |  |  |  |  |  |  |  |  |  |  |  |  |  |  |  |  |  |  |  |  |  |  |  |  |  |  |  | | --- | --- | --- | --- | --- | --- | --- | --- | --- | --- | --- | --- | --- | --- | --- | --- | --- | --- | --- | --- | --- | --- | --- | --- | --- | --- | --- | --- | --- | --- | --- | --- | --- | --- | --- | --- | --- | --- | --- | --- | --- | --- | --- | --- | --- | --- | --- | --- | --- | --- | --- | --- | --- | --- | --- | --- | --- | --- | --- | --- | --- | --- | --- | --- | --- | --- | --- | --- | --- | --- | --- | --- | --- | --- | --- | --- | --- | --- | --- | --- | --- | --- | --- | --- | --- | --- | --- | --- | | Chimp panTro2 chr2a 44818737 44818808 + **AA** | CAGGATCGTGAATTAATTGACCCATTTCCGGTACCTCTGGTCATAATTGGAAGTAAATATGATGTTTTTCAG|  |  |  |  |  |  |  |  |  |  |  |  |  |  |  |  |  |  |  |  |  |  |  |  |  |  |  |  |  |  |  |  |  |  |  |  |  |  |  |  |  |  |  |  |  |  |  |  |  |  |  |  |  |  |  |  |  |  |  |  |  |  |  |  |  |  |  |  |  |  |  |  |  |  |  |  |  |  |  |  | | --- | --- | --- | --- | --- | --- | --- | --- | --- | --- | --- | --- | --- | --- | --- | --- | --- | --- | --- | --- | --- | --- | --- | --- | --- | --- | --- | --- | --- | --- | --- | --- | --- | --- | --- | --- | --- | --- | --- | --- | --- | --- | --- | --- | --- | --- | --- | --- | --- | --- | --- | --- | --- | --- | --- | --- | --- | --- | --- | --- | --- | --- | --- | --- | --- | --- | --- | --- | --- | --- | --- | --- | --- | --- | --- | --- | --- | --- | --- | --- | | Gorilla gorGor1 Supercontig\_0254163 538 609 - **AA** | CAGGATCGTGAATTAATTGACCCATTTCCAGTACCTCTGGTCATAATTGGAAGTAAATATGATGTTTTTCAG|  |  |  |  |  |  |  |  |  |  |  |  |  |  |  |  |  |  |  |  |  |  |  |  |  |  |  |  |  |  |  |  |  |  |  |  |  |  |  |  |  |  |  |  |  |  |  |  |  |  |  |  |  |  |  |  |  |  |  |  |  |  |  |  |  |  |  |  |  |  |  |  | | --- | --- | --- | --- | --- | --- | --- | --- | --- | --- | --- | --- | --- | --- | --- | --- | --- | --- | --- | --- | --- | --- | --- | --- | --- | --- | --- | --- | --- | --- | --- | --- | --- | --- | --- | --- | --- | --- | --- | --- | --- | --- | --- | --- | --- | --- | --- | --- | --- | --- | --- | --- | --- | --- | --- | --- | --- | --- | --- | --- | --- | --- | --- | --- | --- | --- | --- | --- | --- | --- | --- | --- | | Orangutan ponAbe2 chr2a 67647682 67647753 - **AA** | CAGGATCGTGAATTAATTGACCCATTTCCAGTACCTCTGGTCATAATTGGAAGTAAATATGATATTTTTCAG|  |  |  |  |  |  |  |  |  |  |  |  |  |  |  |  |  |  |  |  |  |  |  |  |  |  |  |  |  |  |  |  |  |  |  |  |  |  |  |  |  |  |  |  |  |  |  |  |  |  |  |  |  |  |  |  |  |  |  |  |  |  |  |  | | --- | --- | --- | --- | --- | --- | --- | --- | --- | --- | --- | --- | --- | --- | --- | --- | --- | --- | --- | --- | --- | --- | --- | --- | --- | --- | --- | --- | --- | --- | --- | --- | --- | --- | --- | --- | --- | --- | --- | --- | --- | --- | --- | --- | --- | --- | --- | --- | --- | --- | --- | --- | --- | --- | --- | --- | --- | --- | --- | --- | --- | --- | --- | --- | | Rhesus rheMac2 chr13 43915623 43915694 + **AA** | CAGGATCGTGAATTAATTGACCCATTTCCAGTACCTCTGGTCATAATTGGAAGTAAATATGATATTTTTCAG|  |  |  |  |  |  |  |  |  |  |  |  |  |  |  |  |  |  |  |  |  |  |  |  |  |  |  |  |  |  |  |  |  |  |  |  |  |  |  |  |  |  |  |  |  |  |  |  |  |  |  |  |  |  |  |  | | --- | --- | --- | --- | --- | --- | --- | --- | --- | --- | --- | --- | --- | --- | --- | --- | --- | --- | --- | --- | --- | --- | --- | --- | --- | --- | --- | --- | --- | --- | --- | --- | --- | --- | --- | --- | --- | --- | --- | --- | --- | --- | --- | --- | --- | --- | --- | --- | --- | --- | --- | --- | --- | --- | --- | --- | | Baboon papHam1 scaffold268 185519 185590 - **AA** | CAGGATCGTGAATTAATTGACCCATTTCCAGTACCTCTGGTCATAATTGGAAGTAAATATGATATTTTTCAG|  |  |  |  |  |  |  |  |  |  |  |  |  |  |  |  |  |  |  |  |  |  |  |  |  |  |  |  |  |  |  |  |  |  |  |  |  |  |  |  |  |  |  |  |  |  |  |  | | --- | --- | --- | --- | --- | --- | --- | --- | --- | --- | --- | --- | --- | --- | --- | --- | --- | --- | --- | --- | --- | --- | --- | --- | --- | --- | --- | --- | --- | --- | --- | --- | --- | --- | --- | --- | --- | --- | --- | --- | --- | --- | --- | --- | --- | --- | --- | --- | | Marmoset calJac1 Contig1007 494477 494548 - **AA** | CAGGATCGTGAAATAATTGACCCATTTCCAGTACCTCTGGTCATTATCGGAAGTAAATATGATATTTTTCAG|  |  |  |  |  |  |  |  |  |  |  |  |  |  |  |  |  |  |  |  |  |  |  |  |  |  |  |  |  |  |  |  |  |  |  |  |  |  |  |  | | --- | --- | --- | --- | --- | --- | --- | --- | --- | --- | --- | --- | --- | --- | --- | --- | --- | --- | --- | --- | --- | --- | --- | --- | --- | --- | --- | --- | --- | --- | --- | --- | --- | --- | --- | --- | --- | --- | --- | --- | | Tarsier tarSyr1 scaffold\_9653 15471 15542 + **AA** | CAGGATCGTGAATTAATTGATCCATTTCCAATACCTCTGGTCATAATTGGAAGTAAATATGATATTTTTCAG|  |  |  |  |  |  |  |  |  |  |  |  |  |  |  |  |  |  |  |  |  |  |  |  |  |  |  |  |  |  |  |  | | --- | --- | --- | --- | --- | --- | --- | --- | --- | --- | --- | --- | --- | --- | --- | --- | --- | --- | --- | --- | --- | --- | --- | --- | --- | --- | --- | --- | --- | --- | --- | --- | | Galago otoGar1 scaffold\_87102.1-63967 48737 48808 + **AA** | CAGGATCGTGAATTAATTGACCCATTTCCAATACCTCTGGTCATAATTGGAAGTAAATATGATATTTTTCAG|  |  |  |  |  |  |  |  |  |  |  |  |  |  |  |  |  |  |  |  |  |  |  |  | | --- | --- | --- | --- | --- | --- | --- | --- | --- | --- | --- | --- | --- | --- | --- | --- | --- | --- | --- | --- | --- | --- | --- | --- | | Mouse mm9 chr17 85043442 85043513 + **AA** | CAGGACCGGGAATTAATTGACCCATTTCCAATACCTCTGGTCATTATTGGAAGTAAATATGATATTTTCCAG|  |  |  |  |  |  |  |  |  |  |  |  |  |  |  |  | | --- | --- | --- | --- | --- | --- | --- | --- | --- | --- | --- | --- | --- | --- | --- | --- | | Cow bosTau4 chr11 27368866 27368937 + **AA** | CAGGATCGTGAATTACTTGACCCATTTCCAATTCCTCTGGTCATAATCGGAAGTAAATACGATATTTTTCAG|  |  |  |  |  |  |  |  | | --- | --- | --- | --- | --- | --- | --- | --- | | Dog canFam2 chr10 49381939 49382010 + **AA** | CAGGACCGTGAATTAGTTGACCCATTTCTAATACCTCTGGTCATAATTGGAAGTAAATACGATATTTTTCAG | | | | | | | | | | | | | | | | | | | | | | | | | | | | | | | | | | | | | | | | | | | | | | | | | | | | | | | | | | | | | | | | | | | | | | | | | | | | | | | | | | | | | | | | | | | |

**Alignment** (splice site sequences are in lowercase)  

```
Human      agCAGGATCATGAATTAATTGACCCATTTCCGGTACCTCTGGTCATAATTGGAAGTAAATATGATGTTTTTCAGgt
Chimp      .a.......G..................................................................
Gorilla    .a.......G.....................A............................................
Orangutan  .a.......G.....................A.................................A..........
Rhesus     .a.......G.....................A.................................A..........
Baboon     .a.......G.....................A.................................A..........
Marmoset   .a.......G....A................A..............T..C...............A..........
Tarsier    .a.......G............T........AA................................A..........
Galago     .a.......G.....................AA................................A..........
Mouse      .a.....C.GG....................AA.............T..................A....C.....
Cow        .a.......G.......C.............AA.T..............C...........C...A..........
Dog        .a.....C.G.......G............TAA............................C...A..........
```

---

## 11. uc002smk.1\_5\_7

**Summary**  

|  |  |  |  |  |  |  |  |  |  |  |  |  |  |  |  |  |  |  |  |  |  |  |  |  |  |
| --- | --- | --- | --- | --- | --- | --- | --- | --- | --- | --- | --- | --- | --- | --- | --- | --- | --- | --- | --- | --- | --- | --- | --- | --- | --- |
| No Exon ID Position (hg19) Dir Human acceptor Chimp acceptor Category Usage Gene symbol Protein accession mRNA accession Gene title Note|  |  |  |  |  |  |  |  |  |  |  |  |  | | --- | --- | --- | --- | --- | --- | --- | --- | --- | --- | --- | --- | --- | | 11 uc002smk.1\_5\_7 chr2:74758694 + AG GG (A1) shift; increase; inframe alternative HTRA2 O43464-3 AF141306.1 HtrA serine peptidase 2  | | | | | | | | | | | | | | | | | | | | | | | | | |

**Orthologs**  

|  |  |  |  |  |  |  |  |  |  |  |  |  |  |  |  |  |  |  |  |  |  |  |  |  |  |  |  |  |  |  |  |  |  |  |  |  |  |  |  |  |  |  |  |  |  |  |  |  |  |  |  |  |  |  |  |  |  |  |  |  |  |  |  |  |  |  |  |  |  |  |  |  |  |  |  |  |  |  |  |  |  |  |  |  |  |  |  |
| --- | --- | --- | --- | --- | --- | --- | --- | --- | --- | --- | --- | --- | --- | --- | --- | --- | --- | --- | --- | --- | --- | --- | --- | --- | --- | --- | --- | --- | --- | --- | --- | --- | --- | --- | --- | --- | --- | --- | --- | --- | --- | --- | --- | --- | --- | --- | --- | --- | --- | --- | --- | --- | --- | --- | --- | --- | --- | --- | --- | --- | --- | --- | --- | --- | --- | --- | --- | --- | --- | --- | --- | --- | --- | --- | --- | --- | --- | --- | --- | --- | --- | --- | --- | --- | --- | --- | --- |
| Species Assembly Chromosome Exon start Exon end Dir Acceptor Exon sequence|  |  |  |  |  |  |  |  |  |  |  |  |  |  |  |  |  |  |  |  |  |  |  |  |  |  |  |  |  |  |  |  |  |  |  |  |  |  |  |  |  |  |  |  |  |  |  |  |  |  |  |  |  |  |  |  |  |  |  |  |  |  |  |  |  |  |  |  |  |  |  |  |  |  |  |  |  |  |  |  | | --- | --- | --- | --- | --- | --- | --- | --- | --- | --- | --- | --- | --- | --- | --- | --- | --- | --- | --- | --- | --- | --- | --- | --- | --- | --- | --- | --- | --- | --- | --- | --- | --- | --- | --- | --- | --- | --- | --- | --- | --- | --- | --- | --- | --- | --- | --- | --- | --- | --- | --- | --- | --- | --- | --- | --- | --- | --- | --- | --- | --- | --- | --- | --- | --- | --- | --- | --- | --- | --- | --- | --- | --- | --- | --- | --- | --- | --- | --- | --- | | Human hg19 chr2 74758694 74758829 + **AG** | GCTAGGGAACTGGGGGCTGTATCCCTGCAGGATGGGGAGGTGATTGGAGTGAACACCATGAAGGTCACAGCTGGAATCTCCTTTGCCATCCCTTCTGATCGTCTTCGAGAGTTTCTGCATCGTGGGGAAAAGAAGA|  |  |  |  |  |  |  |  |  |  |  |  |  |  |  |  |  |  |  |  |  |  |  |  |  |  |  |  |  |  |  |  |  |  |  |  |  |  |  |  |  |  |  |  |  |  |  |  |  |  |  |  |  |  |  |  |  |  |  |  |  |  |  |  |  |  |  |  |  |  |  |  | | --- | --- | --- | --- | --- | --- | --- | --- | --- | --- | --- | --- | --- | --- | --- | --- | --- | --- | --- | --- | --- | --- | --- | --- | --- | --- | --- | --- | --- | --- | --- | --- | --- | --- | --- | --- | --- | --- | --- | --- | --- | --- | --- | --- | --- | --- | --- | --- | --- | --- | --- | --- | --- | --- | --- | --- | --- | --- | --- | --- | --- | --- | --- | --- | --- | --- | --- | --- | --- | --- | --- | --- | | Chimp panTro2 chr2a 76131026 76131161 + **GG** | GCTAGGGAACTGGGGGCTGTATCCCTGCAGGATGGGGAGGTGATTGGAGTGAACACCATGAAGGTCACAGCTGGAATCTCCTTTGCCATCCCTTCTGATCGTCTTCGAGAGTTTCTGCATCGTGGGGAAAAGAAGA|  |  |  |  |  |  |  |  |  |  |  |  |  |  |  |  |  |  |  |  |  |  |  |  |  |  |  |  |  |  |  |  |  |  |  |  |  |  |  |  |  |  |  |  |  |  |  |  |  |  |  |  |  |  |  |  |  |  |  |  |  |  |  |  | | --- | --- | --- | --- | --- | --- | --- | --- | --- | --- | --- | --- | --- | --- | --- | --- | --- | --- | --- | --- | --- | --- | --- | --- | --- | --- | --- | --- | --- | --- | --- | --- | --- | --- | --- | --- | --- | --- | --- | --- | --- | --- | --- | --- | --- | --- | --- | --- | --- | --- | --- | --- | --- | --- | --- | --- | --- | --- | --- | --- | --- | --- | --- | --- | | Orangutan ponAbe2 chr2a 36168757 36168892 - **GG** | GCTAGGGAACTGGGGGCTGTATCCCTGCAGGATGGGGAGGTGATTGGAGTGAACACCATGAAGGTCACAGCTGGAATCTCCTTTGCCATCCCTTCTGATCGTCTTCGAGAGTTTCTGCATCGTGGGGAAAAGAAGA|  |  |  |  |  |  |  |  |  |  |  |  |  |  |  |  |  |  |  |  |  |  |  |  |  |  |  |  |  |  |  |  |  |  |  |  |  |  |  |  |  |  |  |  |  |  |  |  |  |  |  |  |  |  |  |  | | --- | --- | --- | --- | --- | --- | --- | --- | --- | --- | --- | --- | --- | --- | --- | --- | --- | --- | --- | --- | --- | --- | --- | --- | --- | --- | --- | --- | --- | --- | --- | --- | --- | --- | --- | --- | --- | --- | --- | --- | --- | --- | --- | --- | --- | --- | --- | --- | --- | --- | --- | --- | --- | --- | --- | --- | | Rhesus rheMac2 chr13 74761901 74762036 + **GG** | GCTAGGGAACTGGGGGATGTATCCCTGCAGGATGGAGAGGTGATTGGAGTGAACACCATGAAGGTCACAGCTGGAATCTCCTTTGCCATCCCTTCTGATCGCCTTCGAGAGTTTCTGCATCGTGGGGAAAAGAAGA|  |  |  |  |  |  |  |  |  |  |  |  |  |  |  |  |  |  |  |  |  |  |  |  |  |  |  |  |  |  |  |  |  |  |  |  |  |  |  |  |  |  |  |  |  |  |  |  | | --- | --- | --- | --- | --- | --- | --- | --- | --- | --- | --- | --- | --- | --- | --- | --- | --- | --- | --- | --- | --- | --- | --- | --- | --- | --- | --- | --- | --- | --- | --- | --- | --- | --- | --- | --- | --- | --- | --- | --- | --- | --- | --- | --- | --- | --- | --- | --- | | Baboon papHam1 Contig78359 121 256 - **GG** | GCTAGGGAACTGGGGGATGTATCCCTGCAGGATGGAGAGGTGATTGGAGTGAACACCATGAAGGTCACAGCTGGAATCTCCTTTGCCATCCCTTCTGATCGCCTTCGAGAGTTTCTGCATCGTGGGGAAAAGAAGA|  |  |  |  |  |  |  |  |  |  |  |  |  |  |  |  |  |  |  |  |  |  |  |  |  |  |  |  |  |  |  |  |  |  |  |  |  |  |  |  | | --- | --- | --- | --- | --- | --- | --- | --- | --- | --- | --- | --- | --- | --- | --- | --- | --- | --- | --- | --- | --- | --- | --- | --- | --- | --- | --- | --- | --- | --- | --- | --- | --- | --- | --- | --- | --- | --- | --- | --- | | Marmoset calJac1 Contig2284 188503 188638 - **GG** | GCTAGGGAACAGGGGGCTGTGTCCCTGCAGGATGGAGAAGTGATTGGAGTGAACACCATGAAGGTCACAGCTGGAATCTCCTTTGCTATCCCTTCTGATCGCCTTCGAGAATTTCTGCGTCGTGGGGAACAGGAAA|  |  |  |  |  |  |  |  |  |  |  |  |  |  |  |  |  |  |  |  |  |  |  |  |  |  |  |  |  |  |  |  | | --- | --- | --- | --- | --- | --- | --- | --- | --- | --- | --- | --- | --- | --- | --- | --- | --- | --- | --- | --- | --- | --- | --- | --- | --- | --- | --- | --- | --- | --- | --- | --- | | Lemur micMur1 scaffold\_1959 222426 222561 + **GG** | GCTAGGGAACAGGGGGCTGTGTCCTTGCAGGATGGGGAGGTGATCGGAGTGAACACTATGAAGGTCACAGCCGGAATCTCCTTTGCCATCCCTTCTGACCGCCTTCGAGAGTTTCTTCGTCGTGGGGAAAAGAAGA|  |  |  |  |  |  |  |  |  |  |  |  |  |  |  |  |  |  |  |  |  |  |  |  | | --- | --- | --- | --- | --- | --- | --- | --- | --- | --- | --- | --- | --- | --- | --- | --- | --- | --- | --- | --- | --- | --- | --- | --- | | Mouse mm9 chr6 83002692 83002827 - **GG** | GCTAGGGGACAGGGGGCTGTGTCCCTGCAGGATGGGGAGGTGATTGGAGTGAACACCATGAAGGTGACAGCTGGAATCTCCTTTGCCATCCCTTCTGATCGCCTTAGGGAGTTTCTGCATCGCGGGGAAAAGAAAA|  |  |  |  |  |  |  |  |  |  |  |  |  |  |  |  | | --- | --- | --- | --- | --- | --- | --- | --- | --- | --- | --- | --- | --- | --- | --- | --- | | Cow bosTau4 chr11 10420816 10420951 - **GG** | GTTAGAGAATAGTGGGCTGTGTCCCAACAGGATGGGGAGGTGATTGGGGTGAATACCATGAAGGTCACATCTGGAATCTCCTTTGCCATCCCTTCTGATCGCCTTCGAGAGTTTCTGCATCGTGGAGAAAAGAAGA|  |  |  |  |  |  |  |  | | --- | --- | --- | --- | --- | --- | --- | --- | | Dog canFam2 chr17 51680864 51680999 - **GG** | GCTAGGGATTAGTGGGCTCTGTTCCTGCAGGATGGGGAGGTGATTGGAGTGAATACCATGAAGGTCACAGCTGGAATCTCCTTTGCCATCCCTTCTGATCGCCTTCGAGAGTTTCTGCATCGTGGGGAAAAGAAGA | | | | | | | | | | | | | | | | | | | | | | | | | | | | | | | | | | | | | | | | | | | | | | | | | | | | | | | | | | | | | | | | | | | | | | | | | | | | | |

**Alignment** (splice site sequences are in lowercase)  

```
Human      agGCTAGGGAACTGGGGGCTGTATCCCTGCAGGATGGGGAGGTGATTGGAGTGAACACCATGAAGGTCACAGCTGGAATC
Chimp      g...............................................................................
Orangutan  g...............................................................................
Rhesus     g.................A..................A..........................................
Baboon     g.................A..................A..........................................
Marmoset   g...........A.........G..............A..A.......................................
Lemur      g...........A.........G...T...................C...........T..............C......
Mouse      g........G..A.........G............................................G............
Cow        g..T...A...TA.T.......G....AA....................G.....T...............T........
Dog        g.........TTA.T.....C.G.T..............................T........................

Human      TCCTTTGCCATCCCTTCTGATCGTCTTCGAGAGTTTCTGCATCGTGGGGAAAAGAAGAgt
Chimp      ............................................................
Orangutan  ............................................................
Rhesus     .......................C....................................
Baboon     .......................C....................................
Marmoset   ........T..............C........A.......G..........C..G.A...
Lemur      ....................C..C..............T.G...................
Mouse      .......................C...A.G..............C...........A...
Cow        .......................C.......................A............
Dog        .......................C....................................
```

---

## 12. uc002tzo.2\_3\_9

**Summary**  

|  |  |  |  |  |  |  |  |  |  |  |  |  |  |  |  |  |  |  |  |  |  |  |  |  |  |
| --- | --- | --- | --- | --- | --- | --- | --- | --- | --- | --- | --- | --- | --- | --- | --- | --- | --- | --- | --- | --- | --- | --- | --- | --- | --- |
| No Exon ID Position (hg19) Dir Human acceptor Chimp acceptor Category Usage Gene symbol Protein accession mRNA accession Gene title Note|  |  |  |  |  |  |  |  |  |  |  |  |  | | --- | --- | --- | --- | --- | --- | --- | --- | --- | --- | --- | --- | --- | | 12 uc002tzo.2\_3\_9 chr2:158958552 + AG GA (A7) novel start alternative UPP2 NP\_001128570.1 NM\_001135098.1 uridine phosphorylase 2  | | | | | | | | | | | | | | | | | | | | | | | | | |

**Orthologs**  

|  |  |  |  |  |  |  |  |  |  |  |  |  |  |  |  |  |  |  |  |  |  |  |  |  |  |  |  |  |  |  |  |  |  |  |  |  |  |  |  |  |  |  |  |  |  |  |  |  |  |  |  |  |  |  |  |  |  |  |  |  |  |  |  |  |  |  |  |  |  |  |  |  |  |  |  |  |  |  |  |  |  |  |  |  |  |  |  |  |  |  |  |  |  |  |  |
| --- | --- | --- | --- | --- | --- | --- | --- | --- | --- | --- | --- | --- | --- | --- | --- | --- | --- | --- | --- | --- | --- | --- | --- | --- | --- | --- | --- | --- | --- | --- | --- | --- | --- | --- | --- | --- | --- | --- | --- | --- | --- | --- | --- | --- | --- | --- | --- | --- | --- | --- | --- | --- | --- | --- | --- | --- | --- | --- | --- | --- | --- | --- | --- | --- | --- | --- | --- | --- | --- | --- | --- | --- | --- | --- | --- | --- | --- | --- | --- | --- | --- | --- | --- | --- | --- | --- | --- | --- | --- | --- | --- | --- | --- | --- | --- |
| Species Assembly Chromosome Exon start Exon end Dir Acceptor Exon sequence|  |  |  |  |  |  |  |  |  |  |  |  |  |  |  |  |  |  |  |  |  |  |  |  |  |  |  |  |  |  |  |  |  |  |  |  |  |  |  |  |  |  |  |  |  |  |  |  |  |  |  |  |  |  |  |  |  |  |  |  |  |  |  |  |  |  |  |  |  |  |  |  |  |  |  |  |  |  |  |  |  |  |  |  |  |  |  |  | | --- | --- | --- | --- | --- | --- | --- | --- | --- | --- | --- | --- | --- | --- | --- | --- | --- | --- | --- | --- | --- | --- | --- | --- | --- | --- | --- | --- | --- | --- | --- | --- | --- | --- | --- | --- | --- | --- | --- | --- | --- | --- | --- | --- | --- | --- | --- | --- | --- | --- | --- | --- | --- | --- | --- | --- | --- | --- | --- | --- | --- | --- | --- | --- | --- | --- | --- | --- | --- | --- | --- | --- | --- | --- | --- | --- | --- | --- | --- | --- | --- | --- | --- | --- | --- | --- | --- | --- | | Human hg19 chr2 158958552 158958637 + **AG** | GTGACTTTTCACATAGTAGAGAGAATGGCTTCAGTTATACCTGCCTCCAATAGGTCCATGAGATCTGACAGGAATACATATGTTGG|  |  |  |  |  |  |  |  |  |  |  |  |  |  |  |  |  |  |  |  |  |  |  |  |  |  |  |  |  |  |  |  |  |  |  |  |  |  |  |  |  |  |  |  |  |  |  |  |  |  |  |  |  |  |  |  |  |  |  |  |  |  |  |  |  |  |  |  |  |  |  |  |  |  |  |  |  |  |  |  | | --- | --- | --- | --- | --- | --- | --- | --- | --- | --- | --- | --- | --- | --- | --- | --- | --- | --- | --- | --- | --- | --- | --- | --- | --- | --- | --- | --- | --- | --- | --- | --- | --- | --- | --- | --- | --- | --- | --- | --- | --- | --- | --- | --- | --- | --- | --- | --- | --- | --- | --- | --- | --- | --- | --- | --- | --- | --- | --- | --- | --- | --- | --- | --- | --- | --- | --- | --- | --- | --- | --- | --- | --- | --- | --- | --- | --- | --- | --- | --- | | Chimp panTro2 chr2b 162731779 162731864 + **GA** | GTGACTTTTCACATAGTAGAGAGAATGGCTTCAGTTATACCTGCCTCCAATAGTTCCATGAGATCTGACAGGAATACATATGTTGG|  |  |  |  |  |  |  |  |  |  |  |  |  |  |  |  |  |  |  |  |  |  |  |  |  |  |  |  |  |  |  |  |  |  |  |  |  |  |  |  |  |  |  |  |  |  |  |  |  |  |  |  |  |  |  |  |  |  |  |  |  |  |  |  |  |  |  |  |  |  |  |  | | --- | --- | --- | --- | --- | --- | --- | --- | --- | --- | --- | --- | --- | --- | --- | --- | --- | --- | --- | --- | --- | --- | --- | --- | --- | --- | --- | --- | --- | --- | --- | --- | --- | --- | --- | --- | --- | --- | --- | --- | --- | --- | --- | --- | --- | --- | --- | --- | --- | --- | --- | --- | --- | --- | --- | --- | --- | --- | --- | --- | --- | --- | --- | --- | --- | --- | --- | --- | --- | --- | --- | --- | | Gorilla gorGor1 Supercontig\_0070212 2645 2730 - **GA** | GTGACTTTTCACATANTAGAGAGAATGGCTTCAGTTATACCTGCCTCCAATAGTTCCATGAGATCTGACAGGAATACATATGTTGG|  |  |  |  |  |  |  |  |  |  |  |  |  |  |  |  |  |  |  |  |  |  |  |  |  |  |  |  |  |  |  |  |  |  |  |  |  |  |  |  |  |  |  |  |  |  |  |  |  |  |  |  |  |  |  |  |  |  |  |  |  |  |  |  | | --- | --- | --- | --- | --- | --- | --- | --- | --- | --- | --- | --- | --- | --- | --- | --- | --- | --- | --- | --- | --- | --- | --- | --- | --- | --- | --- | --- | --- | --- | --- | --- | --- | --- | --- | --- | --- | --- | --- | --- | --- | --- | --- | --- | --- | --- | --- | --- | --- | --- | --- | --- | --- | --- | --- | --- | --- | --- | --- | --- | --- | --- | --- | --- | | Orangutan ponAbe2 chr2b 47781659 47781744 + **GA** | GTGACTATTCACATAGTAGAGAGAATGGCTTCGGTTATACCTGTCTCCAATAGTTCCATGAAATCTGACAGGAATACATACATTGG|  |  |  |  |  |  |  |  |  |  |  |  |  |  |  |  |  |  |  |  |  |  |  |  |  |  |  |  |  |  |  |  |  |  |  |  |  |  |  |  |  |  |  |  |  |  |  |  |  |  |  |  |  |  |  |  | | --- | --- | --- | --- | --- | --- | --- | --- | --- | --- | --- | --- | --- | --- | --- | --- | --- | --- | --- | --- | --- | --- | --- | --- | --- | --- | --- | --- | --- | --- | --- | --- | --- | --- | --- | --- | --- | --- | --- | --- | --- | --- | --- | --- | --- | --- | --- | --- | --- | --- | --- | --- | --- | --- | --- | --- | | Rhesus rheMac2 chr12 21689320 21689405 + **GA** | GTGACTATTCACATAGTAGAGAGAATGGCTTCGGTTATACCTGCCTCCAATAGTTCCATGAGATCTGATAGGAATACATATGTGGG|  |  |  |  |  |  |  |  |  |  |  |  |  |  |  |  |  |  |  |  |  |  |  |  |  |  |  |  |  |  |  |  |  |  |  |  |  |  |  |  |  |  |  |  |  |  |  |  | | --- | --- | --- | --- | --- | --- | --- | --- | --- | --- | --- | --- | --- | --- | --- | --- | --- | --- | --- | --- | --- | --- | --- | --- | --- | --- | --- | --- | --- | --- | --- | --- | --- | --- | --- | --- | --- | --- | --- | --- | --- | --- | --- | --- | --- | --- | --- | --- | | Baboon papHam1 scaffold11107 33443 33528 + **GA** | GTGACTATTCACATAGTAGAGAGAATGGCTTCGGTTATACCTGCCTCCAATAGTTCCATGAGATCTGATAGGAATACATATGTTGG|  |  |  |  |  |  |  |  |  |  |  |  |  |  |  |  |  |  |  |  |  |  |  |  |  |  |  |  |  |  |  |  |  |  |  |  |  |  |  |  | | --- | --- | --- | --- | --- | --- | --- | --- | --- | --- | --- | --- | --- | --- | --- | --- | --- | --- | --- | --- | --- | --- | --- | --- | --- | --- | --- | --- | --- | --- | --- | --- | --- | --- | --- | --- | --- | --- | --- | --- | | Marmoset calJac1 Contig8926 13368 13453 + **GA** | GTGACTGTTCACACAGTAGAGAGAATGGCTTCGGTTTTACCTGCCTCCAAAAGATCCATGAGATCTGACGGGAATACATATGTTGG|  |  |  |  |  |  |  |  |  |  |  |  |  |  |  |  |  |  |  |  |  |  |  |  |  |  |  |  |  |  |  |  | | --- | --- | --- | --- | --- | --- | --- | --- | --- | --- | --- | --- | --- | --- | --- | --- | --- | --- | --- | --- | --- | --- | --- | --- | --- | --- | --- | --- | --- | --- | --- | --- | | Tarsier tarSyr1 scaffold\_506693 68 153 + **GA** | GTACCTATCCACATAGTGGAAAATATAGCTTTGGTTTTATCTGCCACCAATAGATCCATGAGATGTGGTAGGAATACATATATTGG|  |  |  |  |  |  |  |  |  |  |  |  |  |  |  |  |  |  |  |  |  |  |  |  | | --- | --- | --- | --- | --- | --- | --- | --- | --- | --- | --- | --- | --- | --- | --- | --- | --- | --- | --- | --- | --- | --- | --- | --- | | Lemur micMur1 scaffold\_1196 190701 190786 + **CA** | GTGACTATCCACACAGGACAAAGGATGGCTTCGGCTTTACCTGCCTCCAATACATCCATGAGATCTGACAGGAATACATATGTTGG|  |  |  |  |  |  |  |  |  |  |  |  |  |  |  |  | | --- | --- | --- | --- | --- | --- | --- | --- | --- | --- | --- | --- | --- | --- | --- | --- | | Galago otoGar1 scaffold\_108496.1-16178 10052 10137 + **AA** | GCGACTGTCCACATAGTGCAAAGTATGGCCTCCGTCCTACCTGCTTCCAATAGCTGCATGAGAGCTGACAGGAAGGCATACGTTGG|  |  |  |  |  |  |  |  | | --- | --- | --- | --- | --- | --- | --- | --- | | Cow bosTau4 chr2 39649904 39649989 - **GA** | GTGACTATCTTCATATTACAAAGTATGGCTTCAGTTTTACCTGCTTCCAATACATCCATGAGATCTGACAGGAATACATATGTTGG | | | | | | | | | | | | | | | | | | | | | | | | | | | | | | | | | | | | | | | | | | | | | | | | | | | | | | | | | | | | | | | | | | | | | | | | | | | | | | | | | | | | |

**Alignment** (splice site sequences are in lowercase)  

```
Human      agGTGACTTTTCACATAGTAGAGAGAATGGCTTCAGTTATACCTGCCTCCAATAGGTCCATGAGATCTGACAGGAATACA
Chimp      ga.....................................................T........................
Gorilla    ga...............N.....................................T........................
Orangutan  ga......A.........................G..........T.........T.......A................
Rhesus     ga......A.........................G....................T..............T.........
Baboon     ga......A.........................G....................T..............T.........
Marmoset   ga......G......C..................G...T.............A..A...............G........
Tarsier    ga..AC..A.C........G..A.AT..A....TG...T..T.....A.......A..........G..GT.........
Lemur      ca......A.C....C..G.C.A..G........G.C.T...............CA........................
Galago     .a.C....G.C........GC.A..T.....C..C..CC.......T........C.G.......G..........GG..
Cow        ga......A.CTT....T..C.A..T............T.......T.......CA........................

Human      TATGTTGGgt
Chimp      ..........
Gorilla    ..........
Orangutan  ..CA......
Rhesus     .....G....
Baboon     ..........
Marmoset   ..........
Tarsier    ...A......
Lemur      ..........
Galago     ..C.......
Cow        ..........
```

---

## 13. uc002vnu.2\_6\_8

**Summary**  

|  |  |  |  |  |  |  |  |  |  |  |  |  |  |  |  |  |  |  |  |  |  |  |  |  |  |
| --- | --- | --- | --- | --- | --- | --- | --- | --- | --- | --- | --- | --- | --- | --- | --- | --- | --- | --- | --- | --- | --- | --- | --- | --- | --- |
| No Exon ID Position (hg19) Dir Human acceptor Chimp acceptor Category Usage Gene symbol Protein accession mRNA accession Gene title Note|  |  |  |  |  |  |  |  |  |  |  |  |  | | --- | --- | --- | --- | --- | --- | --- | --- | --- | --- | --- | --- | --- | | 13 uc002vnu.2\_6\_8 chr2:224845117 - AG AA (A1) shift; increase; inframe alternative SERPINE2 NP\_006207.1 NM\_006216.3 serpin peptidase inhibitor, clade E (nexin, plasminogen activator inhibitor type 1), member 2 NAGNAG | | | | | | | | | | | | | | | | | | | | | | | | | |

**Orthologs**  

|  |  |  |  |  |  |  |  |  |  |  |  |  |  |  |  |  |  |  |  |  |  |  |  |  |  |  |  |  |  |  |  |  |  |  |  |  |  |  |  |  |  |  |  |  |  |  |  |  |  |  |  |  |  |  |  |  |  |  |  |  |  |  |  |  |  |  |  |  |  |  |  |  |  |  |  |  |  |  |  |  |  |  |  |  |  |  |  |  |  |  |  |  |  |  |  |  |  |  |  |  |  |  |  |  |  |  |  |  |  |  |  |
| --- | --- | --- | --- | --- | --- | --- | --- | --- | --- | --- | --- | --- | --- | --- | --- | --- | --- | --- | --- | --- | --- | --- | --- | --- | --- | --- | --- | --- | --- | --- | --- | --- | --- | --- | --- | --- | --- | --- | --- | --- | --- | --- | --- | --- | --- | --- | --- | --- | --- | --- | --- | --- | --- | --- | --- | --- | --- | --- | --- | --- | --- | --- | --- | --- | --- | --- | --- | --- | --- | --- | --- | --- | --- | --- | --- | --- | --- | --- | --- | --- | --- | --- | --- | --- | --- | --- | --- | --- | --- | --- | --- | --- | --- | --- | --- | --- | --- | --- | --- | --- | --- | --- | --- | --- | --- | --- | --- | --- | --- | --- | --- |
| Species Assembly Chromosome Exon start Exon end Dir Acceptor Exon sequence|  |  |  |  |  |  |  |  |  |  |  |  |  |  |  |  |  |  |  |  |  |  |  |  |  |  |  |  |  |  |  |  |  |  |  |  |  |  |  |  |  |  |  |  |  |  |  |  |  |  |  |  |  |  |  |  |  |  |  |  |  |  |  |  |  |  |  |  |  |  |  |  |  |  |  |  |  |  |  |  |  |  |  |  |  |  |  |  |  |  |  |  |  |  |  |  |  |  |  |  |  |  |  |  | | --- | --- | --- | --- | --- | --- | --- | --- | --- | --- | --- | --- | --- | --- | --- | --- | --- | --- | --- | --- | --- | --- | --- | --- | --- | --- | --- | --- | --- | --- | --- | --- | --- | --- | --- | --- | --- | --- | --- | --- | --- | --- | --- | --- | --- | --- | --- | --- | --- | --- | --- | --- | --- | --- | --- | --- | --- | --- | --- | --- | --- | --- | --- | --- | --- | --- | --- | --- | --- | --- | --- | --- | --- | --- | --- | --- | --- | --- | --- | --- | --- | --- | --- | --- | --- | --- | --- | --- | --- | --- | --- | --- | --- | --- | --- | --- | --- | --- | --- | --- | --- | --- | --- | --- | | Human hg19 chr2 224845028 224845117 - **AG** | CAGGGTCAGAAAACCTCCATGTTTCTCATATCTTGCAAAAAGCAAAAATTGAAGTCAGTGAAGATGGAACCAAAGCTTCAGCAGCAACAA|  |  |  |  |  |  |  |  |  |  |  |  |  |  |  |  |  |  |  |  |  |  |  |  |  |  |  |  |  |  |  |  |  |  |  |  |  |  |  |  |  |  |  |  |  |  |  |  |  |  |  |  |  |  |  |  |  |  |  |  |  |  |  |  |  |  |  |  |  |  |  |  |  |  |  |  |  |  |  |  |  |  |  |  |  |  |  |  |  |  |  |  |  |  |  |  | | --- | --- | --- | --- | --- | --- | --- | --- | --- | --- | --- | --- | --- | --- | --- | --- | --- | --- | --- | --- | --- | --- | --- | --- | --- | --- | --- | --- | --- | --- | --- | --- | --- | --- | --- | --- | --- | --- | --- | --- | --- | --- | --- | --- | --- | --- | --- | --- | --- | --- | --- | --- | --- | --- | --- | --- | --- | --- | --- | --- | --- | --- | --- | --- | --- | --- | --- | --- | --- | --- | --- | --- | --- | --- | --- | --- | --- | --- | --- | --- | --- | --- | --- | --- | --- | --- | --- | --- | --- | --- | --- | --- | --- | --- | --- | --- | | Chimp panTro2 chr2b 229997294 229997383 - **AA** | CAGGGTCAGAAAACCTCCATGTTTCTCATATCTTGCAAAAAGCAAAAATTGAAGTCAGTGAAGATGGAACCAAAGCTTCAGCAGCAACAA|  |  |  |  |  |  |  |  |  |  |  |  |  |  |  |  |  |  |  |  |  |  |  |  |  |  |  |  |  |  |  |  |  |  |  |  |  |  |  |  |  |  |  |  |  |  |  |  |  |  |  |  |  |  |  |  |  |  |  |  |  |  |  |  |  |  |  |  |  |  |  |  |  |  |  |  |  |  |  |  |  |  |  |  |  |  |  |  | | --- | --- | --- | --- | --- | --- | --- | --- | --- | --- | --- | --- | --- | --- | --- | --- | --- | --- | --- | --- | --- | --- | --- | --- | --- | --- | --- | --- | --- | --- | --- | --- | --- | --- | --- | --- | --- | --- | --- | --- | --- | --- | --- | --- | --- | --- | --- | --- | --- | --- | --- | --- | --- | --- | --- | --- | --- | --- | --- | --- | --- | --- | --- | --- | --- | --- | --- | --- | --- | --- | --- | --- | --- | --- | --- | --- | --- | --- | --- | --- | --- | --- | --- | --- | --- | --- | --- | --- | | Gorilla gorGor1 Supercontig\_0006475 6178 6267 - **AA** | CAGGGTCAGAAAACCTCCATGTTTCTCATATCTTGCAAAAAGCAAAAATTGAAGTCAGTGAAGATGGAACCAAAGCTTCAGCAGCAACAA|  |  |  |  |  |  |  |  |  |  |  |  |  |  |  |  |  |  |  |  |  |  |  |  |  |  |  |  |  |  |  |  |  |  |  |  |  |  |  |  |  |  |  |  |  |  |  |  |  |  |  |  |  |  |  |  |  |  |  |  |  |  |  |  |  |  |  |  |  |  |  |  |  |  |  |  |  |  |  |  | | --- | --- | --- | --- | --- | --- | --- | --- | --- | --- | --- | --- | --- | --- | --- | --- | --- | --- | --- | --- | --- | --- | --- | --- | --- | --- | --- | --- | --- | --- | --- | --- | --- | --- | --- | --- | --- | --- | --- | --- | --- | --- | --- | --- | --- | --- | --- | --- | --- | --- | --- | --- | --- | --- | --- | --- | --- | --- | --- | --- | --- | --- | --- | --- | --- | --- | --- | --- | --- | --- | --- | --- | --- | --- | --- | --- | --- | --- | --- | --- | | Orangutan ponAbe2 chr2b 116096812 116096901 - **AA** | CAGGGTCAGAAAACCTCCATGTTTCTCATATCTTGCAAAAAGCAAAAATTGAAGTCAGTGAAGATGGAACCAAAGCTTCAGCAGCAACAA|  |  |  |  |  |  |  |  |  |  |  |  |  |  |  |  |  |  |  |  |  |  |  |  |  |  |  |  |  |  |  |  |  |  |  |  |  |  |  |  |  |  |  |  |  |  |  |  |  |  |  |  |  |  |  |  |  |  |  |  |  |  |  |  |  |  |  |  |  |  |  |  | | --- | --- | --- | --- | --- | --- | --- | --- | --- | --- | --- | --- | --- | --- | --- | --- | --- | --- | --- | --- | --- | --- | --- | --- | --- | --- | --- | --- | --- | --- | --- | --- | --- | --- | --- | --- | --- | --- | --- | --- | --- | --- | --- | --- | --- | --- | --- | --- | --- | --- | --- | --- | --- | --- | --- | --- | --- | --- | --- | --- | --- | --- | --- | --- | --- | --- | --- | --- | --- | --- | --- | --- | | Rhesus rheMac2 chr12 87860837 87860926 - **AA** | CAGGGTCAGAAAACCTCCATGTTTCTCATATCTTGCAAAAAGCAAAAATTGAAGTCAGTGAAGATGGAACCAAAGCTTCAGCAGCAACAA|  |  |  |  |  |  |  |  |  |  |  |  |  |  |  |  |  |  |  |  |  |  |  |  |  |  |  |  |  |  |  |  |  |  |  |  |  |  |  |  |  |  |  |  |  |  |  |  |  |  |  |  |  |  |  |  |  |  |  |  |  |  |  |  | | --- | --- | --- | --- | --- | --- | --- | --- | --- | --- | --- | --- | --- | --- | --- | --- | --- | --- | --- | --- | --- | --- | --- | --- | --- | --- | --- | --- | --- | --- | --- | --- | --- | --- | --- | --- | --- | --- | --- | --- | --- | --- | --- | --- | --- | --- | --- | --- | --- | --- | --- | --- | --- | --- | --- | --- | --- | --- | --- | --- | --- | --- | --- | --- | | Baboon papHam1 scaffold1966 103286 103375 - **AA** | CAGGGTCAGAAAACCTCCATGTTTCTCATATCTTGCAAAAAGCAAAAATTGAAGTCAGTGAAGATGGAACCAAAGCTTCAGCAGCAACAA|  |  |  |  |  |  |  |  |  |  |  |  |  |  |  |  |  |  |  |  |  |  |  |  |  |  |  |  |  |  |  |  |  |  |  |  |  |  |  |  |  |  |  |  |  |  |  |  |  |  |  |  |  |  |  |  | | --- | --- | --- | --- | --- | --- | --- | --- | --- | --- | --- | --- | --- | --- | --- | --- | --- | --- | --- | --- | --- | --- | --- | --- | --- | --- | --- | --- | --- | --- | --- | --- | --- | --- | --- | --- | --- | --- | --- | --- | --- | --- | --- | --- | --- | --- | --- | --- | --- | --- | --- | --- | --- | --- | --- | --- | | Marmoset calJac1 Contig846 201927 202016 - **AA** | CAGGGTCAGAAAACCTCCATGTTTCTCAAATTTTGCAAAAAGCAAAAATTGAAGTCAGTGAAGATGGAACCAAAGCTTCAGCAGCAACAA|  |  |  |  |  |  |  |  |  |  |  |  |  |  |  |  |  |  |  |  |  |  |  |  |  |  |  |  |  |  |  |  |  |  |  |  |  |  |  |  |  |  |  |  |  |  |  |  | | --- | --- | --- | --- | --- | --- | --- | --- | --- | --- | --- | --- | --- | --- | --- | --- | --- | --- | --- | --- | --- | --- | --- | --- | --- | --- | --- | --- | --- | --- | --- | --- | --- | --- | --- | --- | --- | --- | --- | --- | --- | --- | --- | --- | --- | --- | --- | --- | | Tarsier tarSyr1 scaffold\_19321 30401 30490 - **AA** | CAGAGTCAGAAAACCTGCACGTTTCTCACATCTTGCAAAAAGCAAAAATCGAAGTCAGTGAAGATGGAACCAAAGCTTCAGCAGCAACAA|  |  |  |  |  |  |  |  |  |  |  |  |  |  |  |  |  |  |  |  |  |  |  |  |  |  |  |  |  |  |  |  |  |  |  |  |  |  |  |  | | --- | --- | --- | --- | --- | --- | --- | --- | --- | --- | --- | --- | --- | --- | --- | --- | --- | --- | --- | --- | --- | --- | --- | --- | --- | --- | --- | --- | --- | --- | --- | --- | --- | --- | --- | --- | --- | --- | --- | --- | | Lemur micMur1 scaffold\_742 214941 215030 - **AA** | CAGGGTCAGAGAACCTCCATGTTTCTCACATCTTGCAAAAAGCAAAAATTGAAGTCAGTGAAGATGGAACCAAAGCTTCAGCAGCTACAA|  |  |  |  |  |  |  |  |  |  |  |  |  |  |  |  |  |  |  |  |  |  |  |  |  |  |  |  |  |  |  |  | | --- | --- | --- | --- | --- | --- | --- | --- | --- | --- | --- | --- | --- | --- | --- | --- | --- | --- | --- | --- | --- | --- | --- | --- | --- | --- | --- | --- | --- | --- | --- | --- | | Galago otoGar1 scaffold\_109401.1-310823 232172 232261 - **AA** | CAGGGTCCGAAAACCTTCATGTTTCTCACATCTTGCAAAAAGCAAAAATTGAAGTCAGTGAAGATGGAACCAAAGCTTCAGCAGCTACAA|  |  |  |  |  |  |  |  |  |  |  |  |  |  |  |  |  |  |  |  |  |  |  |  | | --- | --- | --- | --- | --- | --- | --- | --- | --- | --- | --- | --- | --- | --- | --- | --- | --- | --- | --- | --- | --- | --- | --- | --- | | Mouse mm9 chr1 79796043 79796132 - **AA** | CAGGGTCAGAGAGCCTTCATGTCTCTCACATCTTGCAAAAAGCAAAAATTGAAGTCAGTGAAGATGGAACCAAAGCTTCGGCAGCAACAA|  |  |  |  |  |  |  |  |  |  |  |  |  |  |  |  | | --- | --- | --- | --- | --- | --- | --- | --- | --- | --- | --- | --- | --- | --- | --- | --- | | Cow bosTau4 chr2 116633129 116633218 - **AA** | CAGGGTCAGAAAACCTTCATGTTTCTCACATCTTGCAAAAAGCAAAAATCGAAGTCAGTGAAGATGGAACCAAAGCTTCAGCAGCAACAA|  |  |  |  |  |  |  |  | | --- | --- | --- | --- | --- | --- | --- | --- | | Dog canFam2 chr37 32705071 32705160 - **AA** | CAGGGTCAGAAAACCTGCACGTGTCTCACATCTTGCAGAAAGCAAAAATCGAAGTCAGCGAAGACGGAACCAAAGCTTCAGCAGCAACAA | | | | | | | | | | | | | | | | | | | | | | | | | | | | | | | | | | | | | | | | | | | | | | | | | | | | | | | | | | | | | | | | | | | | | | | | | | | | | | | | | | | | | | | | | | | | | | | | | | |

**Alignment** (splice site sequences are in lowercase)  

```
Human      agCAGGGTCAGAAAACCTCCATGTTTCTCATATCTTGCAAAAAGCAAAAATTGAAGTCAGTGAAGATGGAACCAAAGCTT
Chimp      .a..............................................................................
Gorilla    .a..............................................................................
Orangutan  .a..............................................................................
Rhesus     .a..............................................................................
Baboon     .a..............................................................................
Marmoset   .a............................A..T..............................................
Tarsier    .a...A............G..C........C....................C............................
Lemur      .a..........G.................C.................................................
Galago     .a.......C........T...........C.................................................
Mouse      .a..........G.G...T.....C.....C.................................................
Cow        .a................T...........C....................C............................
Dog        .a................G..C..G.....C........G...........C........C.....C.............

Human      CAGCAGCAACAAgt
Chimp      ..............
Gorilla    ..............
Orangutan  ..............
Rhesus     ..............
Baboon     ..............
Marmoset   ..............
Tarsier    ..............
Lemur      .......T......
Galago     .......T......
Mouse      .G............
Cow        ..............
Dog        ..............
```

---

## 14. uc003epq.2\_3\_5

**Summary**  

|  |  |  |  |  |  |  |  |  |  |  |  |  |  |  |  |  |  |  |  |  |  |  |  |  |  |
| --- | --- | --- | --- | --- | --- | --- | --- | --- | --- | --- | --- | --- | --- | --- | --- | --- | --- | --- | --- | --- | --- | --- | --- | --- | --- |
| No Exon ID Position (hg19) Dir Human acceptor Chimp acceptor Category Usage Gene symbol Protein accession mRNA accession Gene title Note|  |  |  |  |  |  |  |  |  |  |  |  |  | | --- | --- | --- | --- | --- | --- | --- | --- | --- | --- | --- | --- | --- | | 14 uc003epq.2\_3\_5 chr3:133302846 + AG AA (A1) shift; increase; inframe alternative CDV3 NP\_001127894.1 NM\_001134422.1 protein CDV3 homolog NAGNAG | | | | | | | | | | | | | | | | | | | | | | | | | |

**Orthologs**  

|  |  |  |  |  |  |  |  |  |  |  |  |  |  |  |  |  |  |  |  |  |  |  |  |  |  |  |  |  |  |  |  |  |  |  |  |  |  |  |  |  |  |  |  |  |  |  |  |  |  |  |  |  |  |  |  |  |  |  |  |  |  |  |  |  |  |  |  |  |  |  |  |  |  |  |  |  |  |  |  |  |  |  |  |  |  |  |  |  |  |  |  |  |  |  |  |
| --- | --- | --- | --- | --- | --- | --- | --- | --- | --- | --- | --- | --- | --- | --- | --- | --- | --- | --- | --- | --- | --- | --- | --- | --- | --- | --- | --- | --- | --- | --- | --- | --- | --- | --- | --- | --- | --- | --- | --- | --- | --- | --- | --- | --- | --- | --- | --- | --- | --- | --- | --- | --- | --- | --- | --- | --- | --- | --- | --- | --- | --- | --- | --- | --- | --- | --- | --- | --- | --- | --- | --- | --- | --- | --- | --- | --- | --- | --- | --- | --- | --- | --- | --- | --- | --- | --- | --- | --- | --- | --- | --- | --- | --- | --- | --- |
| Species Assembly Chromosome Exon start Exon end Dir Acceptor Exon sequence|  |  |  |  |  |  |  |  |  |  |  |  |  |  |  |  |  |  |  |  |  |  |  |  |  |  |  |  |  |  |  |  |  |  |  |  |  |  |  |  |  |  |  |  |  |  |  |  |  |  |  |  |  |  |  |  |  |  |  |  |  |  |  |  |  |  |  |  |  |  |  |  |  |  |  |  |  |  |  |  |  |  |  |  |  |  |  |  | | --- | --- | --- | --- | --- | --- | --- | --- | --- | --- | --- | --- | --- | --- | --- | --- | --- | --- | --- | --- | --- | --- | --- | --- | --- | --- | --- | --- | --- | --- | --- | --- | --- | --- | --- | --- | --- | --- | --- | --- | --- | --- | --- | --- | --- | --- | --- | --- | --- | --- | --- | --- | --- | --- | --- | --- | --- | --- | --- | --- | --- | --- | --- | --- | --- | --- | --- | --- | --- | --- | --- | --- | --- | --- | --- | --- | --- | --- | --- | --- | --- | --- | --- | --- | --- | --- | --- | --- | | Human hg19 chr3 133302846 133302994 + **AG** | CAGTGAAAAGGAAGAAGACGATAATGAAAAGAGACAAGATCCAGGTGATAACTGGGAAGAAGGTGGAGGTGGTGGTGGAGGTATGGAAAAATCTTCAGGTCCCTGGAATAAAACAGCTCCAGTACAAGCACCTCCTGCTCCAGTAATTG|  |  |  |  |  |  |  |  |  |  |  |  |  |  |  |  |  |  |  |  |  |  |  |  |  |  |  |  |  |  |  |  |  |  |  |  |  |  |  |  |  |  |  |  |  |  |  |  |  |  |  |  |  |  |  |  |  |  |  |  |  |  |  |  |  |  |  |  |  |  |  |  |  |  |  |  |  |  |  |  | | --- | --- | --- | --- | --- | --- | --- | --- | --- | --- | --- | --- | --- | --- | --- | --- | --- | --- | --- | --- | --- | --- | --- | --- | --- | --- | --- | --- | --- | --- | --- | --- | --- | --- | --- | --- | --- | --- | --- | --- | --- | --- | --- | --- | --- | --- | --- | --- | --- | --- | --- | --- | --- | --- | --- | --- | --- | --- | --- | --- | --- | --- | --- | --- | --- | --- | --- | --- | --- | --- | --- | --- | --- | --- | --- | --- | --- | --- | --- | --- | | Chimp panTro2 chr3 138155556 138155704 + **AA** | CAGTGAAAAGGAAGAAGACGATAATGAAAAGAGACAAGATCCAGGTGATAACTGGGAAGAAGGTGGAGGTGGTGGTGGAGGTATGGAAAAATCTTCAGGTCCCTGGAATAAAACAGCTCCAGTACAAGCACCTCCTGCTCCAGTAATTG|  |  |  |  |  |  |  |  |  |  |  |  |  |  |  |  |  |  |  |  |  |  |  |  |  |  |  |  |  |  |  |  |  |  |  |  |  |  |  |  |  |  |  |  |  |  |  |  |  |  |  |  |  |  |  |  |  |  |  |  |  |  |  |  |  |  |  |  |  |  |  |  | | --- | --- | --- | --- | --- | --- | --- | --- | --- | --- | --- | --- | --- | --- | --- | --- | --- | --- | --- | --- | --- | --- | --- | --- | --- | --- | --- | --- | --- | --- | --- | --- | --- | --- | --- | --- | --- | --- | --- | --- | --- | --- | --- | --- | --- | --- | --- | --- | --- | --- | --- | --- | --- | --- | --- | --- | --- | --- | --- | --- | --- | --- | --- | --- | --- | --- | --- | --- | --- | --- | --- | --- | | Rhesus rheMac2 chr2 154153700 154153848 - **AA** | CAGTGAAAAGGAAGAAGATGATAATGAAAAGAGACAAGATCCAGGTGATAACTGGGAAGAAGGTGGAGGTGGTGGTGGAGGTATGGAAAAATCTTCAGGTCCCTGGAATAAAACAGCTCTGGTCCAAGCACCTCCTGCTCCAGTAATCG|  |  |  |  |  |  |  |  |  |  |  |  |  |  |  |  |  |  |  |  |  |  |  |  |  |  |  |  |  |  |  |  |  |  |  |  |  |  |  |  |  |  |  |  |  |  |  |  |  |  |  |  |  |  |  |  |  |  |  |  |  |  |  |  | | --- | --- | --- | --- | --- | --- | --- | --- | --- | --- | --- | --- | --- | --- | --- | --- | --- | --- | --- | --- | --- | --- | --- | --- | --- | --- | --- | --- | --- | --- | --- | --- | --- | --- | --- | --- | --- | --- | --- | --- | --- | --- | --- | --- | --- | --- | --- | --- | --- | --- | --- | --- | --- | --- | --- | --- | --- | --- | --- | --- | --- | --- | --- | --- | | Baboon papHam1 scaffold8414 151 299 + **AA** | CAGTGAAAAGGAAGAAGATGATACTGAAAAGAGACAAGATCCAGGTGATAACTGGGAAGAAGGTGGAGGTGGTGGTGGAGGTATGGAAAAATCTTCAGGTCCCTGGAATAAAACAGCTCTGGTCCAAGCACCTCCTGCTCCAGTAATCG|  |  |  |  |  |  |  |  |  |  |  |  |  |  |  |  |  |  |  |  |  |  |  |  |  |  |  |  |  |  |  |  |  |  |  |  |  |  |  |  |  |  |  |  |  |  |  |  |  |  |  |  |  |  |  |  | | --- | --- | --- | --- | --- | --- | --- | --- | --- | --- | --- | --- | --- | --- | --- | --- | --- | --- | --- | --- | --- | --- | --- | --- | --- | --- | --- | --- | --- | --- | --- | --- | --- | --- | --- | --- | --- | --- | --- | --- | --- | --- | --- | --- | --- | --- | --- | --- | --- | --- | --- | --- | --- | --- | --- | --- | | Marmoset calJac1 Contig13381 12786 12934 + **AA** | CAGTGAAAAGGAAGAAGATGATAATGAAAAGAGACAAGATCCAGGTGATAACTGGGAAGAAGGTGGAGGTGGTGGTGGTGGTATGGAAAAATCTTCAGGTCCCTGGAACAAAACAGCTCCGGTACAAGCACCTCCTGCTCCAGTAATTG|  |  |  |  |  |  |  |  |  |  |  |  |  |  |  |  |  |  |  |  |  |  |  |  |  |  |  |  |  |  |  |  |  |  |  |  |  |  |  |  |  |  |  |  |  |  |  |  | | --- | --- | --- | --- | --- | --- | --- | --- | --- | --- | --- | --- | --- | --- | --- | --- | --- | --- | --- | --- | --- | --- | --- | --- | --- | --- | --- | --- | --- | --- | --- | --- | --- | --- | --- | --- | --- | --- | --- | --- | --- | --- | --- | --- | --- | --- | --- | --- | | Tarsier tarSyr1 scaffold\_162908 3216 3364 + **AA** | TAGTGAAAAGGAAGAAGACGATAATGAAAAGAGAGAAGATCCAGGCGACAACTGGGAAGAAGGTGGAGGAGGTGGTGGTGGTGTAGAAAAGTCTTCAGGTCCCTGGAATAAAGCAGCTCCGGCCCAAGCACCTCCTGCTCCTGTAATTG|  |  |  |  |  |  |  |  |  |  |  |  |  |  |  |  |  |  |  |  |  |  |  |  |  |  |  |  |  |  |  |  |  |  |  |  |  |  |  |  | | --- | --- | --- | --- | --- | --- | --- | --- | --- | --- | --- | --- | --- | --- | --- | --- | --- | --- | --- | --- | --- | --- | --- | --- | --- | --- | --- | --- | --- | --- | --- | --- | --- | --- | --- | --- | --- | --- | --- | --- | | Lemur micMur1 scaffold\_646 39654 39802 + **TA** | TAGCGAAAAGGAAGAAGAAGATAATGAAAAGAGAGAAGATCCAGGTGATAACTGGGAAGAAGGTGGAGGAGGAGGTGGTGGTATAGAAAAGTCTTCAGGTCCCTGGAATAAAACAGCCCCGGTACAAGCACCTCCTGCTCCAGTAATTG|  |  |  |  |  |  |  |  |  |  |  |  |  |  |  |  |  |  |  |  |  |  |  |  |  |  |  |  |  |  |  |  | | --- | --- | --- | --- | --- | --- | --- | --- | --- | --- | --- | --- | --- | --- | --- | --- | --- | --- | --- | --- | --- | --- | --- | --- | --- | --- | --- | --- | --- | --- | --- | --- | | Galago otoGar1 scaffold\_99972.1-148850 106341 106489 + **AA** | AAGTGAAAAGGAAGAAGATGATAATGAAAAGAGAGAAGATCCAGGTGATAACTGGGAAGAAGGTGGAGGAGGTGGTGGTGGTATAGAAAAATCTTCAGGTCCCTGGAATAAAACAGCTCCGGTTCAGGCACCTCCTGCTCCAGTAATTG|  |  |  |  |  |  |  |  |  |  |  |  |  |  |  |  |  |  |  |  |  |  |  |  | | --- | --- | --- | --- | --- | --- | --- | --- | --- | --- | --- | --- | --- | --- | --- | --- | --- | --- | --- | --- | --- | --- | --- | --- | | Mouse mm9 chr9 103262206 103262351 - **AA** | TAGTGAAAAGGAAGATGATGATAATGAGAAGAGAGAAGATCCAGGAGATAATTGGGAAGAAGGTGGAGGTGGCAGTGGAGCAGAAAAATCTTCAGGTCCCTGGAATAAAACCGCTCCGGTACAAGCGCCTCCTGCTCCAGTAACAG|  |  |  |  |  |  |  |  |  |  |  |  |  |  |  |  | | --- | --- | --- | --- | --- | --- | --- | --- | --- | --- | --- | --- | --- | --- | --- | --- | | Cow bosTau4 chr1 138025864 138026012 - **GA** | TAGTGAAAAGGAAGAAGATGAAGTTGAAAAGAGAGAAGATCCAAGTGATAATTGGGAAGAAGGTGGAGGTGGTGGTGGTGGTGTAGAAAAGTCTTCAGGCCCTTGGAATAAAACTGCTCCGGTACAAGCACCTCCTGCTCCAGTAGTTG|  |  |  |  |  |  |  |  | | --- | --- | --- | --- | --- | --- | --- | --- | | Dog canFam2 chr23 33530133 33530281 + **AA** | TAGTGAAAAGGAAGAAGATGATAATGAGAAAAGAGAAGATCCCGGTGATAACTGGGAAGAAGGTGGAGGTGGCGGTGGTGGTGTAGAAAAGTCTTCAGGTCCCTGGAATAAAACAGCTCCAGTACAAGCACCTCCTGCTGCAGTAATTG | | | | | | | | | | | | | | | | | | | | | | | | | | | | | | | | | | | | | | | | | | | | | | | | | | | | | | | | | | | | | | | | | | | | | | | | | | | | | | | | | | | | |

**Alignment** (splice site sequences are in lowercase)  

```
Human      agCAGTGAAAAGGAAGAAGACGATAATGAAAAGAGACAAGATCCAGGTGATAACTGGGAAGAAGGTGGAGGTGGTGGTGG
Chimp      .a..............................................................................
Rhesus     .a..................T...........................................................
Baboon     .a..................T....C......................................................
Marmoset   .a..................T...........................................................
Tarsier    .aT.................................G..........C..C....................A........
Lemur      taT..C..............A...............G..................................A..A.....
Galago     .aA.................T...............G..................................A........
Mouse      .aT..............T..T........G......G..........A.....T....................CA....
Cow        gaT.................T..AGT..........G........A.......T..........................
Dog        .aT.................T........G..A...G.......C.............................C.....

Human      AGGTATGGAAAAATCTTCAGGTCCCTGGAATAAAACAGCTCCAGTACAAGCACCTCCTGCTCCAGTAATTGgt
Chimp      .........................................................................
Rhesus     .........................................TG..C.......................C...
Baboon     .........................................TG..C.......................C...
Marmoset   T.............................C...........G..............................
Tarsier    T...G.A.....G.....................G.......G.CC.................T.........
Lemur      T.....A.....G..........................C..G..............................
Galago     T.....A...................................G..T..G........................
Mouse      ..---CA.............................C.....G........G................CA...
Cow        T...G.A.....G........C..T...........T.....G........................G.....
Dog        T...G.A.....G................................................G...........
```

---

## 15. uc003gay.2\_6\_10

**Summary**  

|  |  |  |  |  |  |  |  |  |  |  |  |  |  |  |  |  |  |  |  |  |  |  |  |  |  |
| --- | --- | --- | --- | --- | --- | --- | --- | --- | --- | --- | --- | --- | --- | --- | --- | --- | --- | --- | --- | --- | --- | --- | --- | --- | --- |
| No Exon ID Position (hg19) Dir Human acceptor Chimp acceptor Category Usage Gene symbol Protein accession mRNA accession Gene title Note|  |  |  |  |  |  |  |  |  |  |  |  |  | | --- | --- | --- | --- | --- | --- | --- | --- | --- | --- | --- | --- | --- | | 15 uc003gay.2\_6\_10 chr4:678391 - AG CG (A1) shift; increase; inframe alternative MFSD7 Q6UXD7 AY203936.1 major facilitator superfamily domain containing 7 NAGNAG | | | | | | | | | | | | | | | | | | | | | | | | | |

**Orthologs**  

|  |  |  |  |  |  |  |  |  |  |  |  |  |  |  |  |  |  |  |  |  |  |  |  |  |  |  |  |  |  |  |  |  |  |  |  |  |  |  |  |  |  |  |  |  |  |  |  |  |  |  |  |  |  |  |  |  |  |  |  |  |  |  |  |  |  |  |  |  |  |  |  |  |  |  |  |  |  |  |  |
| --- | --- | --- | --- | --- | --- | --- | --- | --- | --- | --- | --- | --- | --- | --- | --- | --- | --- | --- | --- | --- | --- | --- | --- | --- | --- | --- | --- | --- | --- | --- | --- | --- | --- | --- | --- | --- | --- | --- | --- | --- | --- | --- | --- | --- | --- | --- | --- | --- | --- | --- | --- | --- | --- | --- | --- | --- | --- | --- | --- | --- | --- | --- | --- | --- | --- | --- | --- | --- | --- | --- | --- | --- | --- | --- | --- | --- | --- | --- | --- |
| Species Assembly Chromosome Exon start Exon end Dir Acceptor Exon sequence|  |  |  |  |  |  |  |  |  |  |  |  |  |  |  |  |  |  |  |  |  |  |  |  |  |  |  |  |  |  |  |  |  |  |  |  |  |  |  |  |  |  |  |  |  |  |  |  |  |  |  |  |  |  |  |  |  |  |  |  |  |  |  |  |  |  |  |  |  |  |  |  | | --- | --- | --- | --- | --- | --- | --- | --- | --- | --- | --- | --- | --- | --- | --- | --- | --- | --- | --- | --- | --- | --- | --- | --- | --- | --- | --- | --- | --- | --- | --- | --- | --- | --- | --- | --- | --- | --- | --- | --- | --- | --- | --- | --- | --- | --- | --- | --- | --- | --- | --- | --- | --- | --- | --- | --- | --- | --- | --- | --- | --- | --- | --- | --- | --- | --- | --- | --- | --- | --- | --- | --- | | Human hg19 chr4 678272 678391 - **AG** | CAGCTCATGTGGAACAAGGCCTATGTCATCCTGGCTGTGTGCTTGGGGGGAATGATCGGGATCTCTGCCAGCTTCTCAGCCCTCCTGGAGCAGATCCTCTGTGCAAGCGGCCACTCCAGT|  |  |  |  |  |  |  |  |  |  |  |  |  |  |  |  |  |  |  |  |  |  |  |  |  |  |  |  |  |  |  |  |  |  |  |  |  |  |  |  |  |  |  |  |  |  |  |  |  |  |  |  |  |  |  |  |  |  |  |  |  |  |  |  | | --- | --- | --- | --- | --- | --- | --- | --- | --- | --- | --- | --- | --- | --- | --- | --- | --- | --- | --- | --- | --- | --- | --- | --- | --- | --- | --- | --- | --- | --- | --- | --- | --- | --- | --- | --- | --- | --- | --- | --- | --- | --- | --- | --- | --- | --- | --- | --- | --- | --- | --- | --- | --- | --- | --- | --- | --- | --- | --- | --- | --- | --- | --- | --- | | Chimp panTro2 chr4 706909 707021 - **CG** | AAGCTGGTGTGGAACAAGGCCTATGTCATCCTGGCTGTGTGCTTGGGGGGAATGATCGGGATCTCTGCCAGCTTCTCAGCCCTCCTGGAGCAGATCCTCTGTGCAAGCGGCCACTCCAGT|  |  |  |  |  |  |  |  |  |  |  |  |  |  |  |  |  |  |  |  |  |  |  |  |  |  |  |  |  |  |  |  |  |  |  |  |  |  |  |  |  |  |  |  |  |  |  |  |  |  |  |  |  |  |  |  | | --- | --- | --- | --- | --- | --- | --- | --- | --- | --- | --- | --- | --- | --- | --- | --- | --- | --- | --- | --- | --- | --- | --- | --- | --- | --- | --- | --- | --- | --- | --- | --- | --- | --- | --- | --- | --- | --- | --- | --- | --- | --- | --- | --- | --- | --- | --- | --- | --- | --- | --- | --- | --- | --- | --- | --- | | Gorilla gorGor1 Supercontig\_0019078 14270 14389 + **TG** | CAGCTCGTGTGGAACAAGGCCTATGTCATCCTGGCTGTGTGTGGGGGGGGAATGATCGGGATCTCTGCCAGCTTCTCAGCCCTCCTGGAGCAGATCCTCTGTGCAAGCGGCCACTCCAGT|  |  |  |  |  |  |  |  |  |  |  |  |  |  |  |  |  |  |  |  |  |  |  |  |  |  |  |  |  |  |  |  |  |  |  |  |  |  |  |  |  |  |  |  |  |  |  |  | | --- | --- | --- | --- | --- | --- | --- | --- | --- | --- | --- | --- | --- | --- | --- | --- | --- | --- | --- | --- | --- | --- | --- | --- | --- | --- | --- | --- | --- | --- | --- | --- | --- | --- | --- | --- | --- | --- | --- | --- | --- | --- | --- | --- | --- | --- | --- | --- | | Orangutan ponAbe2 chr4 667010 667129 - **CG** | CAGCTCGTGAGGAACAAGGCCTATGTCATCCTGGCTGTGTGCTTGGGGGGAGTCATCGGGATCTTCTCCAGCTTCTCAGCCCTCCTGGAGCAGATCCTCTGCGCAAGCAGCTACTCCAGT|  |  |  |  |  |  |  |  |  |  |  |  |  |  |  |  |  |  |  |  |  |  |  |  |  |  |  |  |  |  |  |  |  |  |  |  |  |  |  |  | | --- | --- | --- | --- | --- | --- | --- | --- | --- | --- | --- | --- | --- | --- | --- | --- | --- | --- | --- | --- | --- | --- | --- | --- | --- | --- | --- | --- | --- | --- | --- | --- | --- | --- | --- | --- | --- | --- | --- | --- | | Baboon papHam1 scaffold3334 88428 88547 + **CG** | AAGCTGGTGCGGAATAAGGCCTATGTCATCCTGGCTGTGTGCTTCGGGGGATGCATCGGGATCTCCTACAGCTTCTCAACCCTCCTGCAGCAGATCCTCTGTGCAAGCGGCTACTCCAGT|  |  |  |  |  |  |  |  |  |  |  |  |  |  |  |  |  |  |  |  |  |  |  |  |  |  |  |  |  |  |  |  | | --- | --- | --- | --- | --- | --- | --- | --- | --- | --- | --- | --- | --- | --- | --- | --- | --- | --- | --- | --- | --- | --- | --- | --- | --- | --- | --- | --- | --- | --- | --- | --- | | Marmoset calJac1 Contig9194 3677 3796 + **TG** | CAGCTCCTGAGGAACAAGGCCTATGTCATCCTGGCTGTGTGTTTTGGGGGAGGCATCGGGATCTTCTCCAGCTTCTCAGCCCTCCTGGAGCAGATCCTCTGTGCGAGCGGCTACTCCAGC|  |  |  |  |  |  |  |  |  |  |  |  |  |  |  |  |  |  |  |  |  |  |  |  | | --- | --- | --- | --- | --- | --- | --- | --- | --- | --- | --- | --- | --- | --- | --- | --- | --- | --- | --- | --- | --- | --- | --- | --- | | Mouse mm9 chr5 108873490 108873609 - **TC** | TAGCTCATACAGAATAAGGCCTACGTCCTCCTGGCCGTATGCTTCGGTGGTGGCATTGGTGTCTTCTCTAGCTTCTCAGCCCTCCTGGAACAGATCCTTTGTGCCAGTGGCTATTCTAAT|  |  |  |  |  |  |  |  |  |  |  |  |  |  |  |  | | --- | --- | --- | --- | --- | --- | --- | --- | --- | --- | --- | --- | --- | --- | --- | --- | | Cow bosTau4 chr6 117908586 117908705 - **TT** | TAGCTCACGAGGAACAAGGCCTACATGGTCCTGGCTGTGTGCTTTGGGGGCGGCATTGGCATCTTCTCCAACTTCTCAGCCCTCCTGGAGCAGGTCCTCTGCGTGAACGGCTACTCCAGC|  |  |  |  |  |  |  |  | | --- | --- | --- | --- | --- | --- | --- | --- | | Dog canFam2 chr3 94562730 94562849 + **CC** | CAGCTCCTGCACAACAGGGCCTACGTCACCCTGGCCGTGTGCTTCGGGGGCGGCATCGGCATCTTCTCCGGCTTTTTGGCCCTCCTGGAGCAGGTCCTCTGCGTGAAGGGCTACTCGAAG | | | | | | | | | | | | | | | | | | | | | | | | | | | | | | | | | | | | | | | | | | | | | | | | | | | | | | | | | | | | | | | | | | | | | | |

**Alignment** (splice site sequences are in lowercase)  

```
Human      agCAGCTCATGTGGAACAAGGCCTATGTCATCCTGGCTGTGTGCTTGGGGGGAATGATCGGGATCTCTGCCAGCTTCTCA
Chimp      c.A....GG.......................................................................
Gorilla    t.......G..................................TGG..................................
Orangutan  c.......G..A.........................................G.C..........TCT...........
Baboon     c.A....GG..C....T.............................C......TGC...........CTA..........
Marmoset   t.......C..A...............................T..T......GGC..........TCT...........
Mouse      tcT.......ACA...T........C...C.......C..A.....C..T..TGGC..T..TG...TCT.T.........
Cow        ttT......C.A.............CA.GG................T.....CGGC..T..C....TCT...A.......
Dog        cc......C..CAC....G......C....C......C........C.....CGGC.....C....TCT..G....T.TG

Human      GCCCTCCTGGAGCAGATCCTCTGTGCAAGCGGCCACTCCAGTgt
Chimp      ............................................
Gorilla    ............................................
Orangutan  .......................C......A..T..........
Baboon     A........C.......................T..........
Marmoset   ..........................G......T.......C..
Mouse      ...........A........T.....C..T...T.T..T.A...
Cow        ...............G.......C.TG.A....T.......C..
Dog        ...............G.......C.TG.AG...T....G.AG..
```

---

## 16. uc003htb.3\_18\_23

**Summary**  

|  |  |  |  |  |  |  |  |  |  |  |  |  |  |  |  |  |  |  |  |  |  |  |  |  |  |
| --- | --- | --- | --- | --- | --- | --- | --- | --- | --- | --- | --- | --- | --- | --- | --- | --- | --- | --- | --- | --- | --- | --- | --- | --- | --- |
| No Exon ID Position (hg19) Dir Human acceptor Chimp acceptor Category Usage Gene symbol Protein accession mRNA accession Gene title Note|  |  |  |  |  |  |  |  |  |  |  |  |  | | --- | --- | --- | --- | --- | --- | --- | --- | --- | --- | --- | --- | --- | | 16 uc003htb.3\_18\_23 chr4:95200074 + AG GG (A1) shift; increase; inframe alternative SMARCAD1 NP\_001121902.1 NM\_001128430.1 SWI/SNF-related matrix-associated actin-dependent regulator of chromatin subfamily A containing DEAD/H box 1  | | | | | | | | | | | | | | | | | | | | | | | | | |

**Orthologs**  

|  |  |  |  |  |  |  |  |  |  |  |  |  |  |  |  |  |  |  |  |  |  |  |  |  |  |  |  |  |  |  |  |  |  |  |  |  |  |  |  |  |  |  |  |  |  |  |  |  |  |  |  |  |  |  |  |  |  |  |  |  |  |  |  |  |  |  |  |  |  |  |  |  |  |  |  |  |  |  |  |  |  |  |  |  |  |  |  |  |  |  |  |  |  |  |  |
| --- | --- | --- | --- | --- | --- | --- | --- | --- | --- | --- | --- | --- | --- | --- | --- | --- | --- | --- | --- | --- | --- | --- | --- | --- | --- | --- | --- | --- | --- | --- | --- | --- | --- | --- | --- | --- | --- | --- | --- | --- | --- | --- | --- | --- | --- | --- | --- | --- | --- | --- | --- | --- | --- | --- | --- | --- | --- | --- | --- | --- | --- | --- | --- | --- | --- | --- | --- | --- | --- | --- | --- | --- | --- | --- | --- | --- | --- | --- | --- | --- | --- | --- | --- | --- | --- | --- | --- | --- | --- | --- | --- | --- | --- | --- | --- |
| Species Assembly Chromosome Exon start Exon end Dir Acceptor Exon sequence|  |  |  |  |  |  |  |  |  |  |  |  |  |  |  |  |  |  |  |  |  |  |  |  |  |  |  |  |  |  |  |  |  |  |  |  |  |  |  |  |  |  |  |  |  |  |  |  |  |  |  |  |  |  |  |  |  |  |  |  |  |  |  |  |  |  |  |  |  |  |  |  |  |  |  |  |  |  |  |  |  |  |  |  |  |  |  |  | | --- | --- | --- | --- | --- | --- | --- | --- | --- | --- | --- | --- | --- | --- | --- | --- | --- | --- | --- | --- | --- | --- | --- | --- | --- | --- | --- | --- | --- | --- | --- | --- | --- | --- | --- | --- | --- | --- | --- | --- | --- | --- | --- | --- | --- | --- | --- | --- | --- | --- | --- | --- | --- | --- | --- | --- | --- | --- | --- | --- | --- | --- | --- | --- | --- | --- | --- | --- | --- | --- | --- | --- | --- | --- | --- | --- | --- | --- | --- | --- | --- | --- | --- | --- | --- | --- | --- | --- | | Human hg19 chr4 95200074 95200201 + **AG** | TCACAGAAAAAAACACAGAAATGTGCAATGTCATGATGCAGTTGAGGAAAATGGCCAATCATCCTTTATTACATCGCCAATATTACACAGCTGAAAAACTCAAGGAAATGTCTCAGCTTATGCTAAAG|  |  |  |  |  |  |  |  |  |  |  |  |  |  |  |  |  |  |  |  |  |  |  |  |  |  |  |  |  |  |  |  |  |  |  |  |  |  |  |  |  |  |  |  |  |  |  |  |  |  |  |  |  |  |  |  |  |  |  |  |  |  |  |  |  |  |  |  |  |  |  |  |  |  |  |  |  |  |  |  | | --- | --- | --- | --- | --- | --- | --- | --- | --- | --- | --- | --- | --- | --- | --- | --- | --- | --- | --- | --- | --- | --- | --- | --- | --- | --- | --- | --- | --- | --- | --- | --- | --- | --- | --- | --- | --- | --- | --- | --- | --- | --- | --- | --- | --- | --- | --- | --- | --- | --- | --- | --- | --- | --- | --- | --- | --- | --- | --- | --- | --- | --- | --- | --- | --- | --- | --- | --- | --- | --- | --- | --- | --- | --- | --- | --- | --- | --- | --- | --- | | Chimp panTro2 chr4 97337187 97337314 + **GG** | TCACAGAAAAAAACACAGAAATGTGCAATGTCATGATGCAGTTGAGGAAAATGGCCAATCATCCTTTATTACATCGCCAATATTACACAGCTGAAAAACTCAAGGAAATGTCTCAGCTTATGCTAAAG|  |  |  |  |  |  |  |  |  |  |  |  |  |  |  |  |  |  |  |  |  |  |  |  |  |  |  |  |  |  |  |  |  |  |  |  |  |  |  |  |  |  |  |  |  |  |  |  |  |  |  |  |  |  |  |  |  |  |  |  |  |  |  |  |  |  |  |  |  |  |  |  | | --- | --- | --- | --- | --- | --- | --- | --- | --- | --- | --- | --- | --- | --- | --- | --- | --- | --- | --- | --- | --- | --- | --- | --- | --- | --- | --- | --- | --- | --- | --- | --- | --- | --- | --- | --- | --- | --- | --- | --- | --- | --- | --- | --- | --- | --- | --- | --- | --- | --- | --- | --- | --- | --- | --- | --- | --- | --- | --- | --- | --- | --- | --- | --- | --- | --- | --- | --- | --- | --- | --- | --- | | Orangutan ponAbe2 chr4 98325097 98325225 + **GG** | TCACAGAGAAAAACACAGAAATGTGCAATGTCATGATGCAGTTGAGGAAAATGGCCAATCATCCTTTATTACATCGCCAATATTACACAGCTGAAANAACTCAAGGAAATGTCTCAGCTTATGCTAAAG|  |  |  |  |  |  |  |  |  |  |  |  |  |  |  |  |  |  |  |  |  |  |  |  |  |  |  |  |  |  |  |  |  |  |  |  |  |  |  |  |  |  |  |  |  |  |  |  |  |  |  |  |  |  |  |  |  |  |  |  |  |  |  |  | | --- | --- | --- | --- | --- | --- | --- | --- | --- | --- | --- | --- | --- | --- | --- | --- | --- | --- | --- | --- | --- | --- | --- | --- | --- | --- | --- | --- | --- | --- | --- | --- | --- | --- | --- | --- | --- | --- | --- | --- | --- | --- | --- | --- | --- | --- | --- | --- | --- | --- | --- | --- | --- | --- | --- | --- | --- | --- | --- | --- | --- | --- | --- | --- | | Rhesus rheMac2 chr5 87261286 87261413 + **GG** | TCACAGAGAAAAACACAGAAATGTGCAATGTCATGATGCAGTTGAGGAAAATGGCCAATCATCCTTTATTACATCGCCAATATTACACAGCTGAAAAACTCAAGGAAATGTCTCAGCTTATGCTAAAG|  |  |  |  |  |  |  |  |  |  |  |  |  |  |  |  |  |  |  |  |  |  |  |  |  |  |  |  |  |  |  |  |  |  |  |  |  |  |  |  |  |  |  |  |  |  |  |  |  |  |  |  |  |  |  |  | | --- | --- | --- | --- | --- | --- | --- | --- | --- | --- | --- | --- | --- | --- | --- | --- | --- | --- | --- | --- | --- | --- | --- | --- | --- | --- | --- | --- | --- | --- | --- | --- | --- | --- | --- | --- | --- | --- | --- | --- | --- | --- | --- | --- | --- | --- | --- | --- | --- | --- | --- | --- | --- | --- | --- | --- | | Baboon papHam1 scaffold30603 17315 17442 - **GG** | TCACAGAGAAAAACACAGAAATGTGCAATGTCATGATGCAGTTGAGGAAAATGGCCAATCATCCTTTATTACATCGCCAATATTACACAGCTGAAAAACTCAAGGAAATGTCTCAGCTTATGCTAAAG|  |  |  |  |  |  |  |  |  |  |  |  |  |  |  |  |  |  |  |  |  |  |  |  |  |  |  |  |  |  |  |  |  |  |  |  |  |  |  |  |  |  |  |  |  |  |  |  | | --- | --- | --- | --- | --- | --- | --- | --- | --- | --- | --- | --- | --- | --- | --- | --- | --- | --- | --- | --- | --- | --- | --- | --- | --- | --- | --- | --- | --- | --- | --- | --- | --- | --- | --- | --- | --- | --- | --- | --- | --- | --- | --- | --- | --- | --- | --- | --- | | Marmoset calJac1 Contig3767 132126 132253 - **GG** | TCACAGAGAAAAACACAGAAATGTGCAATGTCATGATGCAGTTGAGGAAAATGGCCAATCATCCTTTATTACATCGCCAATATTACACAGCTGAAAAACTCAAGGAAATGTCTCAGCTTATGCTAAAG|  |  |  |  |  |  |  |  |  |  |  |  |  |  |  |  |  |  |  |  |  |  |  |  |  |  |  |  |  |  |  |  |  |  |  |  |  |  |  |  | | --- | --- | --- | --- | --- | --- | --- | --- | --- | --- | --- | --- | --- | --- | --- | --- | --- | --- | --- | --- | --- | --- | --- | --- | --- | --- | --- | --- | --- | --- | --- | --- | --- | --- | --- | --- | --- | --- | --- | --- | | Tarsier tarSyr1 scaffold\_21280 1419 1546 + **TA** | TCACAGAAAAAAATACAGAAATGTGCAATGTCATGATGCAGTTGAGAAAAATGGCCAATCATCCTTTATTACACCGCCAATATTACACAGCTGAAAAGCTCAAGGAAATGTCTCAGCTTATGCTAAAG|  |  |  |  |  |  |  |  |  |  |  |  |  |  |  |  |  |  |  |  |  |  |  |  |  |  |  |  |  |  |  |  | | --- | --- | --- | --- | --- | --- | --- | --- | --- | --- | --- | --- | --- | --- | --- | --- | --- | --- | --- | --- | --- | --- | --- | --- | --- | --- | --- | --- | --- | --- | --- | --- | | Lemur micMur1 scaffold\_3875 29473 29600 + **GA** | TCACAGAAAAAAACACAGAAATGTGTAATGTCATGATGCAGCTGAGAAAAATGGCCAATCATCCTTTATTACATCGCCAATATTACACAGCTGAAAAACTCAAGGAAATGTCTCAGCTTATGCTAAAG|  |  |  |  |  |  |  |  |  |  |  |  |  |  |  |  |  |  |  |  |  |  |  |  | | --- | --- | --- | --- | --- | --- | --- | --- | --- | --- | --- | --- | --- | --- | --- | --- | --- | --- | --- | --- | --- | --- | --- | --- | | Mouse mm9 chr6 65049254 65049381 + **GA** | TCACAGAAAAAAACACAGAGATGTGCAATGTCATGATGCAATTGAGAAAAATGGCCAATCACCCTTTACTACACCGCCAGTATTACACACCTGAGAAACTGAAGGAGATGTCTCAGCTAATGCTGAAG|  |  |  |  |  |  |  |  |  |  |  |  |  |  |  |  | | --- | --- | --- | --- | --- | --- | --- | --- | --- | --- | --- | --- | --- | --- | --- | --- | | Cow bosTau4 chr6 32226072 32226199 - **GA** | TCACAGAAAAAAATACAGAAATGTGCAATGTCATGATGCAATTGAGAAAAATGGCCAATCATCCTTTATTACATCGCCAATATTACACAGCTGAAAAACTCAAGGAAATGTCTCAGCTTATGCTAAAG|  |  |  |  |  |  |  |  | | --- | --- | --- | --- | --- | --- | --- | --- | | Dog canFam2 chr32 20122694 20122821 + **AA** | TCACAGAAAAAAGCACAGAAATGTGCAATGTCATGATGCAGTTGAGAAAAATGGCCAATCATCCTTTATTACATCGCCAATATTACACAGCTGAAAAGCTCAAGGAAATGTCTCAGCTTATGCTAAAG | | | | | | | | | | | | | | | | | | | | | | | | | | | | | | | | | | | | | | | | | | | | | | | | | | | | | | | | | | | | | | | | | | | | | | | | | | | | | | | | | | | | |

**Alignment** (splice site sequences are in lowercase)  

```
Human      agTCACAGAAAAAAACACAGAAATGTGCAATGTCATGATGCAGTTGAGGAAAATGGCCAATCATCCTTTATTACATCGCC
Chimp      g...............................................................................
Orangutan  g........G......................................................................
Rhesus     g........G......................................................................
Baboon     g........G......................................................................
Marmoset   g........G......................................................................
Tarsier    ta.............T................................A..........................C....
Lemur      ga.........................T...............C....A...............................
Mouse      ga...................G....................A.....A..............C......C....C....
Cow        ga.............T..........................A.....A...............................
Dog        .a............G.................................A...............................

Human      AATATTACACAGCTGAAAAACTCAAGGAAATGTCTCAGCTTATGCTAAAGgt
Chimp      ....................................................
Orangutan  ....................................................
Rhesus     ....................................................
Baboon     ....................................................
Marmoset   ....................................................
Tarsier    ...................G................................
Lemur      ....................................................
Mouse      .G.........C....G.....G.....G...........A.....G.....
Cow        ....................................................
Dog        ...................G................................
```

---

## 17. uc011chk.1\_3\_6

**Summary**  

|  |  |  |  |  |  |  |  |  |  |  |  |  |  |  |  |  |  |  |  |  |  |  |  |  |  |
| --- | --- | --- | --- | --- | --- | --- | --- | --- | --- | --- | --- | --- | --- | --- | --- | --- | --- | --- | --- | --- | --- | --- | --- | --- | --- |
| No Exon ID Position (hg19) Dir Human acceptor Chimp acceptor Category Usage Gene symbol Protein accession mRNA accession Gene title Note|  |  |  |  |  |  |  |  |  |  |  |  |  | | --- | --- | --- | --- | --- | --- | --- | --- | --- | --- | --- | --- | --- | | 17 uc011chk.1\_3\_6 chr4:141484669 - AG AA (A3) shift; decrease; inframe alternative UCP1 Q4KMT7 BC096736.1 uncoupling protein 1 NAGNAG | | | | | | | | | | | | | | | | | | | | | | | | | |

**Orthologs**  

|  |  |  |  |  |  |  |  |  |  |  |  |  |  |  |  |  |  |  |  |  |  |  |  |  |  |  |  |  |  |  |  |  |  |  |  |  |  |  |  |  |  |  |  |  |  |  |  |  |  |  |  |  |  |  |  |  |  |  |  |  |  |  |  |  |  |  |  |  |  |  |  |  |  |  |  |  |  |  |  |  |  |  |  |  |  |  |  |  |  |  |  |  |  |  |  |  |  |  |  |  |  |  |  |
| --- | --- | --- | --- | --- | --- | --- | --- | --- | --- | --- | --- | --- | --- | --- | --- | --- | --- | --- | --- | --- | --- | --- | --- | --- | --- | --- | --- | --- | --- | --- | --- | --- | --- | --- | --- | --- | --- | --- | --- | --- | --- | --- | --- | --- | --- | --- | --- | --- | --- | --- | --- | --- | --- | --- | --- | --- | --- | --- | --- | --- | --- | --- | --- | --- | --- | --- | --- | --- | --- | --- | --- | --- | --- | --- | --- | --- | --- | --- | --- | --- | --- | --- | --- | --- | --- | --- | --- | --- | --- | --- | --- | --- | --- | --- | --- | --- | --- | --- | --- | --- | --- | --- | --- |
| Species Assembly Chromosome Exon start Exon end Dir Acceptor Exon sequence|  |  |  |  |  |  |  |  |  |  |  |  |  |  |  |  |  |  |  |  |  |  |  |  |  |  |  |  |  |  |  |  |  |  |  |  |  |  |  |  |  |  |  |  |  |  |  |  |  |  |  |  |  |  |  |  |  |  |  |  |  |  |  |  |  |  |  |  |  |  |  |  |  |  |  |  |  |  |  |  |  |  |  |  |  |  |  |  |  |  |  |  |  |  |  |  | | --- | --- | --- | --- | --- | --- | --- | --- | --- | --- | --- | --- | --- | --- | --- | --- | --- | --- | --- | --- | --- | --- | --- | --- | --- | --- | --- | --- | --- | --- | --- | --- | --- | --- | --- | --- | --- | --- | --- | --- | --- | --- | --- | --- | --- | --- | --- | --- | --- | --- | --- | --- | --- | --- | --- | --- | --- | --- | --- | --- | --- | --- | --- | --- | --- | --- | --- | --- | --- | --- | --- | --- | --- | --- | --- | --- | --- | --- | --- | --- | --- | --- | --- | --- | --- | --- | --- | --- | --- | --- | --- | --- | --- | --- | --- | --- | | Human hg19 chr4 141484472 141484669 - **AG** | CACCTAGTTTAGGAAGCAAGATTTTAGCTGGTCTAACGACTGGAGGAGTGGCAGTATTCATTGGGCAACCCACAGAGGTCGTGAAAGTCAGACTTCAAGCACAGAGCCATCTCCACGGAATCAAACCTCGCTACACGGGGACTTATAATGCGTACAGAATAATAGCAACAACCGAAGGCTTGACGGGTCTTTGGAAAG|  |  |  |  |  |  |  |  |  |  |  |  |  |  |  |  |  |  |  |  |  |  |  |  |  |  |  |  |  |  |  |  |  |  |  |  |  |  |  |  |  |  |  |  |  |  |  |  |  |  |  |  |  |  |  |  |  |  |  |  |  |  |  |  |  |  |  |  |  |  |  |  |  |  |  |  |  |  |  |  |  |  |  |  |  |  |  |  | | --- | --- | --- | --- | --- | --- | --- | --- | --- | --- | --- | --- | --- | --- | --- | --- | --- | --- | --- | --- | --- | --- | --- | --- | --- | --- | --- | --- | --- | --- | --- | --- | --- | --- | --- | --- | --- | --- | --- | --- | --- | --- | --- | --- | --- | --- | --- | --- | --- | --- | --- | --- | --- | --- | --- | --- | --- | --- | --- | --- | --- | --- | --- | --- | --- | --- | --- | --- | --- | --- | --- | --- | --- | --- | --- | --- | --- | --- | --- | --- | --- | --- | --- | --- | --- | --- | --- | --- | | Chimp panTro2 chr4 144325998 144326195 - **AA** | CACCTAGTTTAGGAAGCAAGATTTTAGCTGGTCTAACGACTGGAGGAGTGGCAGTATTCATTGGGCAACCCACAGAGGTCGTGAAAGTCAGACTTCAAGCACAGAGCCATCTCCACGGAATCAAACCTCGCTACACGGGGACTTATAACGCGTACAGAATAATAGCAACAACCGAAGGCTTGACGGGTCTTTGGAAAG|  |  |  |  |  |  |  |  |  |  |  |  |  |  |  |  |  |  |  |  |  |  |  |  |  |  |  |  |  |  |  |  |  |  |  |  |  |  |  |  |  |  |  |  |  |  |  |  |  |  |  |  |  |  |  |  |  |  |  |  |  |  |  |  |  |  |  |  |  |  |  |  |  |  |  |  |  |  |  |  | | --- | --- | --- | --- | --- | --- | --- | --- | --- | --- | --- | --- | --- | --- | --- | --- | --- | --- | --- | --- | --- | --- | --- | --- | --- | --- | --- | --- | --- | --- | --- | --- | --- | --- | --- | --- | --- | --- | --- | --- | --- | --- | --- | --- | --- | --- | --- | --- | --- | --- | --- | --- | --- | --- | --- | --- | --- | --- | --- | --- | --- | --- | --- | --- | --- | --- | --- | --- | --- | --- | --- | --- | --- | --- | --- | --- | --- | --- | --- | --- | | Orangutan ponAbe2 chr4 145993480 145993677 - **AA** | CACCTAGTTTAGGAAGCAAGATCTTAGCTGGTCTAACGACTGGAGGAGTGGCAGTATTCATTGGGCAACCCACAGAGGTCGTGAAAGTCAGACTTCAAGCACAGAGCCATCTCCACGGTATCAAACCTCGCTACACGGGGACTTATAACGCGTACAGAATAATAGCAACAACCGAAGGCTTGACGGGTCTTTGGAAAG|  |  |  |  |  |  |  |  |  |  |  |  |  |  |  |  |  |  |  |  |  |  |  |  |  |  |  |  |  |  |  |  |  |  |  |  |  |  |  |  |  |  |  |  |  |  |  |  |  |  |  |  |  |  |  |  |  |  |  |  |  |  |  |  |  |  |  |  |  |  |  |  | | --- | --- | --- | --- | --- | --- | --- | --- | --- | --- | --- | --- | --- | --- | --- | --- | --- | --- | --- | --- | --- | --- | --- | --- | --- | --- | --- | --- | --- | --- | --- | --- | --- | --- | --- | --- | --- | --- | --- | --- | --- | --- | --- | --- | --- | --- | --- | --- | --- | --- | --- | --- | --- | --- | --- | --- | --- | --- | --- | --- | --- | --- | --- | --- | --- | --- | --- | --- | --- | --- | --- | --- | | Rhesus rheMac2 chr5 133016226 133016423 - **AA** | CACCTAGTTTAGGAAGCAAGATCTTAGCTGGTCTAATGACTGGAGGAGTGGCAGTATTCATCGGACAACCCACAGAGGTTGTGAAAGTCAGACTTCAAGCGCAGAGCCATCTCCACGGTATCAAACCTCGCTACACGGGGACTTATAACGCGTACAGAATAGTAGCAACAACGGAAGGCTTGACGGGTCTTTGGAAAG|  |  |  |  |  |  |  |  |  |  |  |  |  |  |  |  |  |  |  |  |  |  |  |  |  |  |  |  |  |  |  |  |  |  |  |  |  |  |  |  |  |  |  |  |  |  |  |  |  |  |  |  |  |  |  |  |  |  |  |  |  |  |  |  | | --- | --- | --- | --- | --- | --- | --- | --- | --- | --- | --- | --- | --- | --- | --- | --- | --- | --- | --- | --- | --- | --- | --- | --- | --- | --- | --- | --- | --- | --- | --- | --- | --- | --- | --- | --- | --- | --- | --- | --- | --- | --- | --- | --- | --- | --- | --- | --- | --- | --- | --- | --- | --- | --- | --- | --- | --- | --- | --- | --- | --- | --- | --- | --- | | Baboon papHam1 scaffold563 44577 44774 - **AA** | CACCTAGTTTAGGAAGCAAGATCTTAGCTGGTCTAATGACTGGAGGAGTGGCAGTATTCATCGGACAACCCACAGAGGTTGTGAAAGTCAGACTTCAAGCGCAGAGCCATCTCCACGGTATCAAACCTCGCTACACGGGGACTTATAACGCGTACAGAATAGTAGCAACAACGGAAGGCTTGACGGGTCTTTGGAAAG|  |  |  |  |  |  |  |  |  |  |  |  |  |  |  |  |  |  |  |  |  |  |  |  |  |  |  |  |  |  |  |  |  |  |  |  |  |  |  |  |  |  |  |  |  |  |  |  |  |  |  |  |  |  |  |  | | --- | --- | --- | --- | --- | --- | --- | --- | --- | --- | --- | --- | --- | --- | --- | --- | --- | --- | --- | --- | --- | --- | --- | --- | --- | --- | --- | --- | --- | --- | --- | --- | --- | --- | --- | --- | --- | --- | --- | --- | --- | --- | --- | --- | --- | --- | --- | --- | --- | --- | --- | --- | --- | --- | --- | --- | | Marmoset calJac1 Contig4071 8957 9154 + **AA** | CACCTAGTTTAGGAAGCAAGATCTTAGCTGGTCTAGCGACTGGAGGAGTGGCAGTATTCATTGGGCAACCCACAGAGGTCGTGAAAGTCAGACTTCAAGCACAGAGCCATCTCCACGGTATCAAACCTCGCTACACGGGGACTTACAACGCCTACAGAATAATAGCAACAACCGAAGGCTTGATGGGTCTTTGGAAAG|  |  |  |  |  |  |  |  |  |  |  |  |  |  |  |  |  |  |  |  |  |  |  |  |  |  |  |  |  |  |  |  |  |  |  |  |  |  |  |  |  |  |  |  |  |  |  |  | | --- | --- | --- | --- | --- | --- | --- | --- | --- | --- | --- | --- | --- | --- | --- | --- | --- | --- | --- | --- | --- | --- | --- | --- | --- | --- | --- | --- | --- | --- | --- | --- | --- | --- | --- | --- | --- | --- | --- | --- | --- | --- | --- | --- | --- | --- | --- | --- | | Tarsier tarSyr1 scaffold\_17027 21900 22097 + **AA** | CTGCTACTTTAGGAAACAAGATTTCGGCGGGTCTGACAACGGGAGGAGTGGCAGTATTCATCGGGCAACCCACAGAGGTGGTGAAAGTCAGACTCCAAGCGCAGAGTCACCTGCACGGTCCCAAACCTCGCTACACGGGGACTTACAATGCTTACAGAATCATAGCAACGACAGAAGGCTTGACGGGGCTTTGGAAAG|  |  |  |  |  |  |  |  |  |  |  |  |  |  |  |  |  |  |  |  |  |  |  |  |  |  |  |  |  |  |  |  |  |  |  |  |  |  |  |  | | --- | --- | --- | --- | --- | --- | --- | --- | --- | --- | --- | --- | --- | --- | --- | --- | --- | --- | --- | --- | --- | --- | --- | --- | --- | --- | --- | --- | --- | --- | --- | --- | --- | --- | --- | --- | --- | --- | --- | --- | | Lemur micMur1 scaffold\_586 6656 6850 - **AA** | CAAGCCTGGGAAGCAAGATCTCAGCTGGCTTGACAACTGGAGGAGTGGCAGTGTTCATTGGGCAGCCCACCGAGGTCGTGAAAGTCAGACTGCAAGCCCAGAGCCATCTACATGGCCTCAAACCTCGCTACACTGGGACTTACAATGCTTACAGAATCATAGCAACAACGGAAGGCTTGACGGGTCTTTGGAAAG|  |  |  |  |  |  |  |  |  |  |  |  |  |  |  |  |  |  |  |  |  |  |  |  |  |  |  |  |  |  |  |  | | --- | --- | --- | --- | --- | --- | --- | --- | --- | --- | --- | --- | --- | --- | --- | --- | --- | --- | --- | --- | --- | --- | --- | --- | --- | --- | --- | --- | --- | --- | --- | --- | | Galago otoGar1 scaffold\_84212.1-414233 372321 372518 + **AA** | CAGGTAGTCTGGGAAGCAAGATCTCAGCTGGCTTAACAACAGGAGGAGTGGCAGTGTTCATTGGGCAGCCCACAGAGGTTGTAAAAGTCAGACTTCAGGCACAGAGCCATCTACATGGCCTCCAACCTCGCTATACCGGGACTTACAATGCTTACAGAATCATAGCAACAACAGAAGGCTTGACAGGTCTTTGGAAAG|  |  |  |  |  |  |  |  |  |  |  |  |  |  |  |  |  |  |  |  |  |  |  |  | | --- | --- | --- | --- | --- | --- | --- | --- | --- | --- | --- | --- | --- | --- | --- | --- | --- | --- | --- | --- | --- | --- | --- | --- | | Mouse mm9 chr8 85817790 85817987 + **AC** | CTGCCTCTCTCGGAAACAAGATCTCAGCCGGCTTAATGACTGGAGGTGTGGCAGTGTTCATTGGGCAGCCTACAGAGGTCGTGAAGGTCAGAATGCAAGCCCAGAGCCATCTGCATGGGATCAAACCCCGCTACACGGGGACCTACAATGCTTACAGAGTTATAGCCACCACAGAAAGCTTGTCAACACTTTGGAAAG|  |  |  |  |  |  |  |  |  |  |  |  |  |  |  |  | | --- | --- | --- | --- | --- | --- | --- | --- | --- | --- | --- | --- | --- | --- | --- | --- | | Cow bosTau4 chr17 18325087 18325284 + **AA** | CAGCTAGTTTAGGAAGCAAGATCTCAGCGGGCCTAATGACTGGAGGCGTGGCCGTGTTCATTGGGCAACCCACAGAGGTGGTCAAGGTCAGACTGCAAGCTCAGAGCCATCTCCACGGTCCCAAACCTCGATACACTGGGACTTACAATGCTTACAGAATTATAGCAACAACAGAAGGCTTGACGGGGCTTTGGAAAG|  |  |  |  |  |  |  |  | | --- | --- | --- | --- | --- | --- | --- | --- | | Dog canFam2 chr19 5287096 5287293 + **AG** | CAGCTAGTTTAGGAAGCAGGATCTCCGCTGGTGTAATGACGGGAGGAGCAGCAGTGTTCATAGGGCAACCCACTGAGGTCGTGAAGGTGAGACTTCAAGCACAGAGCCATCTGCATGGCCGCAAACCTCGGTACACCGGCACTTACAATGCCTACAGAATCATAGCCACCACGGAGGGCCTGACGGGCCTTTGGAAAG | | | | | | | | | | | | | | | | | | | | | | | | | | | | | | | | | | | | | | | | | | | | | | | | | | | | | | | | | | | | | | | | | | | | | | | | | | | | | | | | | | | | | | | | | | | |

**Alignment** (splice site sequences are in lowercase)  

```
Human      agCACCTAGTTTAGGAAGCAAGATTTTAGCTGGTCTAACGACTGGAGGAGTGGCAGTATTCATTGGGCAACCCACAGAGG
Chimp      .a..............................................................................
Orangutan  .a......................C.......................................................
Rhesus     .a......................C.............T........................C..A.............
Baboon     .a......................C.............T........................C..A.............
Marmoset   .a......................C............G..........................................
Tarsier    .a.TG...C........A........CG..G.....G..A..G....................C................
Lemur      .a..---..CC.G...........C.C......CT.G..A.................G...........G.....C....
Galago     .a..GG....C.G...........C.C......CT....A..A..............G...........G..........
Mouse      .c.TG.CTC.C.C....A......C.C...C..CT...T.........T........G...........G..T.......
Cow        .a..G...................C.C...G..C....T.........C.....C..G......................
Dog        ....G...............G...C.CC......G...T...G.......CA.....G.....A...........T....

Human      TCGTGAAAGTCAGACTTCAAGCACAGAGCCATCTCCACGGAATCAAACCTCGCTACACGGGGACTTATAATGCGTACAGA
Chimp      ......................................................................C.........
Orangutan  ........................................T.............................C.........
Rhesus     .T....................G.................T.............................C.........
Baboon     .T....................G.................T.............................C.........
Marmoset   ........................................T..........................C..C..C......
Tarsier    .G..............C.....G.....T..C..G.....TCC........................C.....T......
Lemur      ................G.....C...........A..T..CC................T........C.....T......
Galago     .T..A..............G..............A..T..CC..C..........T..C........C.....T......
Mouse      .......G......A.G.....C...........G..T..G........C..............C..C.....T......
Cow        .G..C..G........G.....T.................TCC.........A.....T........C.....T......
Dog        .......G..G.......................G..T..CCG.........G.....C..C.....C.....C......

Human      ATAATAGCAACAACCGAAGGCTTGACGGGTCTTTGGAAAGgt
Chimp      ..........................................
Orangutan  ..........................................
Rhesus     ...G..........G...........................
Baboon     ...G..........G...........................
Marmoset   .........................T................
Tarsier    ..C........G..A..............G............
Lemur      ..C...........G...........................
Galago     ..C...........A...........A...............
Mouse      G.T.....C..C..A...A.....T.AACA............
Cow        ..T...........A..............G............
Dog        ..C.....C..C..G..G...C.......C............
```

---

## 18. uc003kib.2\_7\_7

**Summary**  

|  |  |  |  |  |  |  |  |  |  |  |  |  |  |  |  |  |  |  |  |  |  |  |  |  |  |
| --- | --- | --- | --- | --- | --- | --- | --- | --- | --- | --- | --- | --- | --- | --- | --- | --- | --- | --- | --- | --- | --- | --- | --- | --- | --- |
| No Exon ID Position (hg19) Dir Human acceptor Chimp acceptor Category Usage Gene symbol Protein accession mRNA accession Gene title Note|  |  |  |  |  |  |  |  |  |  |  |  |  | | --- | --- | --- | --- | --- | --- | --- | --- | --- | --- | --- | --- | --- | | 18 uc003kib.2\_7\_7 chr5:82648944 + AG GA (A1) shift; increase; inframe alternative XRCC4 NP\_071801.1 NM\_022406.2 X-ray repair complementing defective repair in Chinese hamster cells 4 dbSNP:rs1805377 | | | | | | | | | | | | | | | | | | | | | | | | | |

**Orthologs**  

|  |  |  |  |  |  |  |  |  |  |  |  |  |  |  |  |  |  |  |  |  |  |  |  |  |  |  |  |  |  |  |  |  |  |  |  |  |  |  |  |  |  |  |  |  |  |  |  |  |  |  |  |  |  |  |  |  |  |  |  |  |  |  |  |  |  |  |  |  |  |  |  |  |  |  |  |  |  |  |  |  |  |  |  |  |  |  |  |  |  |  |  |  |  |  |  |  |  |  |  |  |  |  |  |  |  |  |  |  |  |  |  |
| --- | --- | --- | --- | --- | --- | --- | --- | --- | --- | --- | --- | --- | --- | --- | --- | --- | --- | --- | --- | --- | --- | --- | --- | --- | --- | --- | --- | --- | --- | --- | --- | --- | --- | --- | --- | --- | --- | --- | --- | --- | --- | --- | --- | --- | --- | --- | --- | --- | --- | --- | --- | --- | --- | --- | --- | --- | --- | --- | --- | --- | --- | --- | --- | --- | --- | --- | --- | --- | --- | --- | --- | --- | --- | --- | --- | --- | --- | --- | --- | --- | --- | --- | --- | --- | --- | --- | --- | --- | --- | --- | --- | --- | --- | --- | --- | --- | --- | --- | --- | --- | --- | --- | --- | --- | --- | --- | --- | --- | --- | --- | --- |
| Species Assembly Chromosome Exon start Exon end Dir Acceptor Exon sequence|  |  |  |  |  |  |  |  |  |  |  |  |  |  |  |  |  |  |  |  |  |  |  |  |  |  |  |  |  |  |  |  |  |  |  |  |  |  |  |  |  |  |  |  |  |  |  |  |  |  |  |  |  |  |  |  |  |  |  |  |  |  |  |  |  |  |  |  |  |  |  |  |  |  |  |  |  |  |  |  |  |  |  |  |  |  |  |  |  |  |  |  |  |  |  |  |  |  |  |  |  |  |  |  | | --- | --- | --- | --- | --- | --- | --- | --- | --- | --- | --- | --- | --- | --- | --- | --- | --- | --- | --- | --- | --- | --- | --- | --- | --- | --- | --- | --- | --- | --- | --- | --- | --- | --- | --- | --- | --- | --- | --- | --- | --- | --- | --- | --- | --- | --- | --- | --- | --- | --- | --- | --- | --- | --- | --- | --- | --- | --- | --- | --- | --- | --- | --- | --- | --- | --- | --- | --- | --- | --- | --- | --- | --- | --- | --- | --- | --- | --- | --- | --- | --- | --- | --- | --- | --- | --- | --- | --- | --- | --- | --- | --- | --- | --- | --- | --- | --- | --- | --- | --- | --- | --- | --- | --- | | Human hg19 chr5 82648944 82649061 + **AG** | TTCTAGGCCTGATTCTTCACTACCTGAGACGTCTAAAAAGGAGCACATCTCAGCTGAAAACATGTCTTTAGAAACTCTGAGAAACAGCAGCCCAGAAGACCTCTTTGATGAGATTTAA|  |  |  |  |  |  |  |  |  |  |  |  |  |  |  |  |  |  |  |  |  |  |  |  |  |  |  |  |  |  |  |  |  |  |  |  |  |  |  |  |  |  |  |  |  |  |  |  |  |  |  |  |  |  |  |  |  |  |  |  |  |  |  |  |  |  |  |  |  |  |  |  |  |  |  |  |  |  |  |  |  |  |  |  |  |  |  |  |  |  |  |  |  |  |  |  | | --- | --- | --- | --- | --- | --- | --- | --- | --- | --- | --- | --- | --- | --- | --- | --- | --- | --- | --- | --- | --- | --- | --- | --- | --- | --- | --- | --- | --- | --- | --- | --- | --- | --- | --- | --- | --- | --- | --- | --- | --- | --- | --- | --- | --- | --- | --- | --- | --- | --- | --- | --- | --- | --- | --- | --- | --- | --- | --- | --- | --- | --- | --- | --- | --- | --- | --- | --- | --- | --- | --- | --- | --- | --- | --- | --- | --- | --- | --- | --- | --- | --- | --- | --- | --- | --- | --- | --- | --- | --- | --- | --- | --- | --- | --- | --- | | Chimp panTro2 chr5 32294709 32294826 - **GA** | TTCTAGGCCTGATTCTTCACTACCTGAGACGTCGAAAAAGGAGCACATCTCAGCTGAAAACATGTCTTTAGAAACTCTGAGAAACAGCAGCCCAGAAGACCTCTTTGATGAGATTTAA|  |  |  |  |  |  |  |  |  |  |  |  |  |  |  |  |  |  |  |  |  |  |  |  |  |  |  |  |  |  |  |  |  |  |  |  |  |  |  |  |  |  |  |  |  |  |  |  |  |  |  |  |  |  |  |  |  |  |  |  |  |  |  |  |  |  |  |  |  |  |  |  |  |  |  |  |  |  |  |  |  |  |  |  |  |  |  |  | | --- | --- | --- | --- | --- | --- | --- | --- | --- | --- | --- | --- | --- | --- | --- | --- | --- | --- | --- | --- | --- | --- | --- | --- | --- | --- | --- | --- | --- | --- | --- | --- | --- | --- | --- | --- | --- | --- | --- | --- | --- | --- | --- | --- | --- | --- | --- | --- | --- | --- | --- | --- | --- | --- | --- | --- | --- | --- | --- | --- | --- | --- | --- | --- | --- | --- | --- | --- | --- | --- | --- | --- | --- | --- | --- | --- | --- | --- | --- | --- | --- | --- | --- | --- | --- | --- | --- | --- | | Gorilla gorGor1 Supercontig\_0008430 18836 18953 + **AA** | TTCTAGGCCTGATTCTTCACTACCTGAGACGTCGAAAAAGGAGCACATCTCAGCTGAAAACATGTCTTTAGAAACTCTGAGAAACAGCAGCCCAGAAGACCTCTTTGATGAGATTTAA|  |  |  |  |  |  |  |  |  |  |  |  |  |  |  |  |  |  |  |  |  |  |  |  |  |  |  |  |  |  |  |  |  |  |  |  |  |  |  |  |  |  |  |  |  |  |  |  |  |  |  |  |  |  |  |  |  |  |  |  |  |  |  |  |  |  |  |  |  |  |  |  |  |  |  |  |  |  |  |  | | --- | --- | --- | --- | --- | --- | --- | --- | --- | --- | --- | --- | --- | --- | --- | --- | --- | --- | --- | --- | --- | --- | --- | --- | --- | --- | --- | --- | --- | --- | --- | --- | --- | --- | --- | --- | --- | --- | --- | --- | --- | --- | --- | --- | --- | --- | --- | --- | --- | --- | --- | --- | --- | --- | --- | --- | --- | --- | --- | --- | --- | --- | --- | --- | --- | --- | --- | --- | --- | --- | --- | --- | --- | --- | --- | --- | --- | --- | --- | --- | | Orangutan ponAbe2 chr5 83570317 83570434 + **AT** | CTCTAGGCCTGATTCTTCACTACCTGAGACGTCGAAAAAGGAGCACATCTCAGCTGAAAACATGTCTTTAGAAACTCTGAGAAACAGCAGCCCAGAAGACCTCTTTGATGAGATTTAA|  |  |  |  |  |  |  |  |  |  |  |  |  |  |  |  |  |  |  |  |  |  |  |  |  |  |  |  |  |  |  |  |  |  |  |  |  |  |  |  |  |  |  |  |  |  |  |  |  |  |  |  |  |  |  |  |  |  |  |  |  |  |  |  |  |  |  |  |  |  |  |  | | --- | --- | --- | --- | --- | --- | --- | --- | --- | --- | --- | --- | --- | --- | --- | --- | --- | --- | --- | --- | --- | --- | --- | --- | --- | --- | --- | --- | --- | --- | --- | --- | --- | --- | --- | --- | --- | --- | --- | --- | --- | --- | --- | --- | --- | --- | --- | --- | --- | --- | --- | --- | --- | --- | --- | --- | --- | --- | --- | --- | --- | --- | --- | --- | --- | --- | --- | --- | --- | --- | --- | --- | | Rhesus rheMac2 chr6 79540522 79540639 + **AA** | CTCTAGGCCTGATTCTTCACTACCTGAGACATCGAAAAAGGAGCACATCTCAGCTGAAAACATGTCTTTAGAAACTCTGAGAAACAGCAGCCCAGAAGACCTCTTTGATGAGATTTAA|  |  |  |  |  |  |  |  |  |  |  |  |  |  |  |  |  |  |  |  |  |  |  |  |  |  |  |  |  |  |  |  |  |  |  |  |  |  |  |  |  |  |  |  |  |  |  |  |  |  |  |  |  |  |  |  |  |  |  |  |  |  |  |  | | --- | --- | --- | --- | --- | --- | --- | --- | --- | --- | --- | --- | --- | --- | --- | --- | --- | --- | --- | --- | --- | --- | --- | --- | --- | --- | --- | --- | --- | --- | --- | --- | --- | --- | --- | --- | --- | --- | --- | --- | --- | --- | --- | --- | --- | --- | --- | --- | --- | --- | --- | --- | --- | --- | --- | --- | --- | --- | --- | --- | --- | --- | --- | --- | | Baboon papHam1 scaffold19342 28160 28277 - **AA** | CTCTAGGCCTGATTCTTCACTACCTGAGACATCGAAAAAGGAGCACATCTCAGCTGAAAACATGTCTTTAGAAACTCTGAGAAACAGCAGCCCAGAAGACCTCTTTGATGAGATTTAA|  |  |  |  |  |  |  |  |  |  |  |  |  |  |  |  |  |  |  |  |  |  |  |  |  |  |  |  |  |  |  |  |  |  |  |  |  |  |  |  |  |  |  |  |  |  |  |  |  |  |  |  |  |  |  |  | | --- | --- | --- | --- | --- | --- | --- | --- | --- | --- | --- | --- | --- | --- | --- | --- | --- | --- | --- | --- | --- | --- | --- | --- | --- | --- | --- | --- | --- | --- | --- | --- | --- | --- | --- | --- | --- | --- | --- | --- | --- | --- | --- | --- | --- | --- | --- | --- | --- | --- | --- | --- | --- | --- | --- | --- | | Marmoset calJac1 Contig2687 88315 88432 + **aa** | CTCTAGGCCTGATTCTTCACTACCTGAGACGTTGAAAAAGGAGCACATCTCAGCTGAAAACATGTCCTTAGAAACTCTGAGAAACAGCAGCCCAGAAGACCTCTTTGATGAGATTTAA|  |  |  |  |  |  |  |  |  |  |  |  |  |  |  |  |  |  |  |  |  |  |  |  |  |  |  |  |  |  |  |  |  |  |  |  |  |  |  |  |  |  |  |  |  |  |  |  | | --- | --- | --- | --- | --- | --- | --- | --- | --- | --- | --- | --- | --- | --- | --- | --- | --- | --- | --- | --- | --- | --- | --- | --- | --- | --- | --- | --- | --- | --- | --- | --- | --- | --- | --- | --- | --- | --- | --- | --- | --- | --- | --- | --- | --- | --- | --- | --- | | Tarsier tarSyr1 scaffold\_30956 15744 15861 - **AA** | CTCTAGGCTTGACTCTTCACCACCTGAGACATTGAAAGAGGAGCGCATCTCGGCTGAAGACATGTCTTTAGAAATGCTAAGAAACAGCAACCCAGAAGACCTCTTTGATGAGATTTAA|  |  |  |  |  |  |  |  |  |  |  |  |  |  |  |  |  |  |  |  |  |  |  |  |  |  |  |  |  |  |  |  |  |  |  |  |  |  |  |  | | --- | --- | --- | --- | --- | --- | --- | --- | --- | --- | --- | --- | --- | --- | --- | --- | --- | --- | --- | --- | --- | --- | --- | --- | --- | --- | --- | --- | --- | --- | --- | --- | --- | --- | --- | --- | --- | --- | --- | --- | | Lemur micMur1 scaffold\_1322 107980 108097 + **AA** | CTCTAGACTTGATTCTTCACTACCTGAAACGTTGAAAAAGGGGCACATCTCAGCTGAAAACATGTCTTTGGAAACTCTGAAAAACAGCAGCCCAGAAGACCTCTTTGATGAGATTTAA|  |  |  |  |  |  |  |  |  |  |  |  |  |  |  |  |  |  |  |  |  |  |  |  |  |  |  |  |  |  |  |  | | --- | --- | --- | --- | --- | --- | --- | --- | --- | --- | --- | --- | --- | --- | --- | --- | --- | --- | --- | --- | --- | --- | --- | --- | --- | --- | --- | --- | --- | --- | --- | --- | | Galago otoGar1 scaffold\_10305.1-641162 189385 189502 - **TG** | CTCTAGACTTGATCCTTCTCTACCTGCGACATTGAAAAAGGAGCCCATCTCAGCTGAAAGCGTGTCTTTGGAAACTCTGAGAAACAGCAGCCCAGAAGACCTCTTTGATGAGATTTAA|  |  |  |  |  |  |  |  |  |  |  |  |  |  |  |  |  |  |  |  |  |  |  |  | | --- | --- | --- | --- | --- | --- | --- | --- | --- | --- | --- | --- | --- | --- | --- | --- | --- | --- | --- | --- | --- | --- | --- | --- | | Mouse mm9 chr13 89988958 89989074 - **TA** | CTGTAGGCTTGCCTCTTCACTACCCCAGACCTTGAAAGAGGAGAGCACCTCTGCTGAAAACATGTCTTTAGAAACCCTGAGAAACAGCAGCCCAGAAGATCTCTTTGATTAGATATA|  |  |  |  |  |  |  |  |  |  |  |  |  |  |  |  | | --- | --- | --- | --- | --- | --- | --- | --- | --- | --- | --- | --- | --- | --- | --- | --- | | Cow bosTau4 chr7 84252830 84252947 + **TA** | CTCTAGGCTTGATCCTCCACTACCTGAGATGCCGAAAAAGGATCATGGCCCAGCTGAAAACATGTCCTTAGAAACTCTAAGAAATAGTAGTCCAGGAGACCTCTTTGATGAGATTTAA|  |  |  |  |  |  |  |  | | --- | --- | --- | --- | --- | --- | --- | --- | | Dog canFam2 chr3 27213538 27213655 - **TA** | CTCTAGGCTTGATCCTCCACTACCTGAGACGTCAAAAAAGGAGCACAGCTCAGCTGAAAACATGTCTTTAGAAACTCTAAGAAACAGTAGTTCAGAAGACCTCTTTGATGAGATTTAA | | | | | | | | | | | | | | | | | | | | | | | | | | | | | | | | | | | | | | | | | | | | | | | | | | | | | | | | | | | | | | | | | | | | | | | | | | | | | | | | | | | | | | | | | | | | | | | | | | |

**Alignment** (splice site sequences are in lowercase)  

```
Human      agTTCTAGGCCTGATTCTTCACTACCTGAGACGTCTAAAAAGGAGCACATCTCAGCTGAAAACATGTCTTTAGAAACTCT
Chimp      ga.................................G............................................
Gorilla    .a.................................G............................................
Orangutan  .tC................................G............................................
Rhesus     .aC.............................A..G............................................
Baboon     .aC.............................A..G............................................
Marmoset   .aC...............................TG................................C...........
Tarsier    .aC.......T...C.......C.........A.TG...G......G......G......G...............TG..
Lemur      .aC.....A.T..................A....TG.......G...........................G........
Galago     t.C.....A.T....C....T.......C...A.TG..........C..............G.G.......G........
Mouse      taC.G.....T..CC...........CC....C.TG...G.....AG..C...T.......................C..
Cow        taC.......T....C..C............T.C.G........T..TGG.C................C...........
Dog        taC.......T....C..C................A.............G..............................

Human      GAGAAACAGCAGCCCAGAAGACCTCTTTGATGAGATTTAA
Chimp      ........................................
Gorilla    ........................................
Orangutan  ........................................
Rhesus     ........................................
Baboon     ........................................
Marmoset   ........................................
Tarsier    A..........A............................
Lemur      ..A.....................................
Galago     ........................................
Mouse      .....................T.........T....A...
Cow        A.....T..T..T....G......................
Dog        A........T..TT..........................
```

---

## 19. uc010kiv.2\_6\_61

**Summary**  

|  |  |  |  |  |  |  |  |  |  |  |  |  |  |  |  |  |  |  |  |  |  |  |  |  |  |
| --- | --- | --- | --- | --- | --- | --- | --- | --- | --- | --- | --- | --- | --- | --- | --- | --- | --- | --- | --- | --- | --- | --- | --- | --- | --- |
| No Exon ID Position (hg19) Dir Human acceptor Chimp acceptor Category Usage Gene symbol Protein accession mRNA accession Gene title Note|  |  |  |  |  |  |  |  |  |  |  |  |  | | --- | --- | --- | --- | --- | --- | --- | --- | --- | --- | --- | --- | --- | | 19 uc010kiv.2\_6\_61 chr6:152629763 - AG GG (A3) shift; decrease; inframe alternative SYNE1 NP\_149062.1 NM\_033071.3 spectrin repeat containing, nuclear envelope 1  | | | | | | | | | | | | | | | | | | | | | | | | | |

**Orthologs**  

|  |  |  |  |  |  |  |  |  |  |  |  |  |  |  |  |  |  |  |  |  |  |  |  |  |  |  |  |  |  |  |  |  |  |  |  |  |  |  |  |  |  |  |  |  |  |  |  |  |  |  |  |  |  |  |  |  |  |  |  |  |  |  |  |  |  |  |  |  |  |  |  |  |  |  |  |  |  |  |  |  |  |  |  |  |  |  |  |  |  |  |  |  |  |  |  |  |  |  |  |  |  |  |  |  |  |  |  |  |  |  |  |
| --- | --- | --- | --- | --- | --- | --- | --- | --- | --- | --- | --- | --- | --- | --- | --- | --- | --- | --- | --- | --- | --- | --- | --- | --- | --- | --- | --- | --- | --- | --- | --- | --- | --- | --- | --- | --- | --- | --- | --- | --- | --- | --- | --- | --- | --- | --- | --- | --- | --- | --- | --- | --- | --- | --- | --- | --- | --- | --- | --- | --- | --- | --- | --- | --- | --- | --- | --- | --- | --- | --- | --- | --- | --- | --- | --- | --- | --- | --- | --- | --- | --- | --- | --- | --- | --- | --- | --- | --- | --- | --- | --- | --- | --- | --- | --- | --- | --- | --- | --- | --- | --- | --- | --- | --- | --- | --- | --- | --- | --- | --- | --- |
| Species Assembly Chromosome Exon start Exon end Dir Acceptor Exon sequence|  |  |  |  |  |  |  |  |  |  |  |  |  |  |  |  |  |  |  |  |  |  |  |  |  |  |  |  |  |  |  |  |  |  |  |  |  |  |  |  |  |  |  |  |  |  |  |  |  |  |  |  |  |  |  |  |  |  |  |  |  |  |  |  |  |  |  |  |  |  |  |  |  |  |  |  |  |  |  |  |  |  |  |  |  |  |  |  |  |  |  |  |  |  |  |  |  |  |  |  |  |  |  |  | | --- | --- | --- | --- | --- | --- | --- | --- | --- | --- | --- | --- | --- | --- | --- | --- | --- | --- | --- | --- | --- | --- | --- | --- | --- | --- | --- | --- | --- | --- | --- | --- | --- | --- | --- | --- | --- | --- | --- | --- | --- | --- | --- | --- | --- | --- | --- | --- | --- | --- | --- | --- | --- | --- | --- | --- | --- | --- | --- | --- | --- | --- | --- | --- | --- | --- | --- | --- | --- | --- | --- | --- | --- | --- | --- | --- | --- | --- | --- | --- | --- | --- | --- | --- | --- | --- | --- | --- | --- | --- | --- | --- | --- | --- | --- | --- | --- | --- | --- | --- | --- | --- | --- | --- | | Human hg19 chr6 152629624 152629763 - **AG** | CTGTGGTACAATATGAACAATATGAGCAAGAAATGAAACATCTCCAGCAACTGATAGAAGGAGCTCACAGAGAGATTGAGGATAAACCTGTTGCCACCAGTAACATACAGGAGCTGCAGGCTCAGATTTCTCGGCATGAG|  |  |  |  |  |  |  |  |  |  |  |  |  |  |  |  |  |  |  |  |  |  |  |  |  |  |  |  |  |  |  |  |  |  |  |  |  |  |  |  |  |  |  |  |  |  |  |  |  |  |  |  |  |  |  |  |  |  |  |  |  |  |  |  |  |  |  |  |  |  |  |  |  |  |  |  |  |  |  |  |  |  |  |  |  |  |  |  |  |  |  |  |  |  |  |  | | --- | --- | --- | --- | --- | --- | --- | --- | --- | --- | --- | --- | --- | --- | --- | --- | --- | --- | --- | --- | --- | --- | --- | --- | --- | --- | --- | --- | --- | --- | --- | --- | --- | --- | --- | --- | --- | --- | --- | --- | --- | --- | --- | --- | --- | --- | --- | --- | --- | --- | --- | --- | --- | --- | --- | --- | --- | --- | --- | --- | --- | --- | --- | --- | --- | --- | --- | --- | --- | --- | --- | --- | --- | --- | --- | --- | --- | --- | --- | --- | --- | --- | --- | --- | --- | --- | --- | --- | --- | --- | --- | --- | --- | --- | --- | --- | | Chimp panTro2 chr6 155091091 155091230 - **GG** | CTGTGGTGCAGTATGAACAATATGAGCAAGAAATGAAACATCTCCAGCAACTGATAGAAGGAGCTCACAGAGAGATTGAGGATAAACCTGTTGCCACCAGTAACATACAGGAGCTGCAGGCTCAGATTTCTCGGCATGAG|  |  |  |  |  |  |  |  |  |  |  |  |  |  |  |  |  |  |  |  |  |  |  |  |  |  |  |  |  |  |  |  |  |  |  |  |  |  |  |  |  |  |  |  |  |  |  |  |  |  |  |  |  |  |  |  |  |  |  |  |  |  |  |  |  |  |  |  |  |  |  |  |  |  |  |  |  |  |  |  |  |  |  |  |  |  |  |  | | --- | --- | --- | --- | --- | --- | --- | --- | --- | --- | --- | --- | --- | --- | --- | --- | --- | --- | --- | --- | --- | --- | --- | --- | --- | --- | --- | --- | --- | --- | --- | --- | --- | --- | --- | --- | --- | --- | --- | --- | --- | --- | --- | --- | --- | --- | --- | --- | --- | --- | --- | --- | --- | --- | --- | --- | --- | --- | --- | --- | --- | --- | --- | --- | --- | --- | --- | --- | --- | --- | --- | --- | --- | --- | --- | --- | --- | --- | --- | --- | --- | --- | --- | --- | --- | --- | --- | --- | | Gorilla gorGor1 Supercontig\_0001868 36145 36284 + **GG** | CTGTGGTGCAGTATGAACAATATGAGCAAGAAATGAAACATCTCCAGCAACTGATAGAAGGAGCTCACAGAGAGATTGAGGATAAACCTGTTGCCACCAGTAACATACAGGAGCTGCAGGCTCAGATTTCTCGGCATGAG|  |  |  |  |  |  |  |  |  |  |  |  |  |  |  |  |  |  |  |  |  |  |  |  |  |  |  |  |  |  |  |  |  |  |  |  |  |  |  |  |  |  |  |  |  |  |  |  |  |  |  |  |  |  |  |  |  |  |  |  |  |  |  |  |  |  |  |  |  |  |  |  |  |  |  |  |  |  |  |  | | --- | --- | --- | --- | --- | --- | --- | --- | --- | --- | --- | --- | --- | --- | --- | --- | --- | --- | --- | --- | --- | --- | --- | --- | --- | --- | --- | --- | --- | --- | --- | --- | --- | --- | --- | --- | --- | --- | --- | --- | --- | --- | --- | --- | --- | --- | --- | --- | --- | --- | --- | --- | --- | --- | --- | --- | --- | --- | --- | --- | --- | --- | --- | --- | --- | --- | --- | --- | --- | --- | --- | --- | --- | --- | --- | --- | --- | --- | --- | --- | | Orangutan ponAbe2 chr6 155578435 155578574 - **GG** | CTGTGGTGCAGTATGAACAATATGAGCAAGAAATGAAACATCTCCAGCAACTGATAGAAGGAGCTCACAGAGAGATTGAGGATAAACCTGTTGCCACCAGTAACATACAGGAGCTGCAGGCTCAGATTTCTCGGCATGAG|  |  |  |  |  |  |  |  |  |  |  |  |  |  |  |  |  |  |  |  |  |  |  |  |  |  |  |  |  |  |  |  |  |  |  |  |  |  |  |  |  |  |  |  |  |  |  |  |  |  |  |  |  |  |  |  |  |  |  |  |  |  |  |  |  |  |  |  |  |  |  |  | | --- | --- | --- | --- | --- | --- | --- | --- | --- | --- | --- | --- | --- | --- | --- | --- | --- | --- | --- | --- | --- | --- | --- | --- | --- | --- | --- | --- | --- | --- | --- | --- | --- | --- | --- | --- | --- | --- | --- | --- | --- | --- | --- | --- | --- | --- | --- | --- | --- | --- | --- | --- | --- | --- | --- | --- | --- | --- | --- | --- | --- | --- | --- | --- | --- | --- | --- | --- | --- | --- | --- | --- | | Rhesus rheMac2 chr4 110773550 110773689 + **GG** | CTGTGGTGCAGTATGAACAATATGAGCAAGAAATGAAACATCTCCAGCAACTGATAGAAGGAGCCCACAGAGAGATTGAGGATAAACCTGTTGCCACCAGTAACATACAGGAGCTGCAGGCTCAGATTTCTCGGCATGAG|  |  |  |  |  |  |  |  |  |  |  |  |  |  |  |  |  |  |  |  |  |  |  |  |  |  |  |  |  |  |  |  |  |  |  |  |  |  |  |  |  |  |  |  |  |  |  |  |  |  |  |  |  |  |  |  |  |  |  |  |  |  |  |  | | --- | --- | --- | --- | --- | --- | --- | --- | --- | --- | --- | --- | --- | --- | --- | --- | --- | --- | --- | --- | --- | --- | --- | --- | --- | --- | --- | --- | --- | --- | --- | --- | --- | --- | --- | --- | --- | --- | --- | --- | --- | --- | --- | --- | --- | --- | --- | --- | --- | --- | --- | --- | --- | --- | --- | --- | --- | --- | --- | --- | --- | --- | --- | --- | | Baboon papHam1 scaffold943 207471 207610 - **GG** | CTGTGGTGCAGTATGAACAATATGAGCAAGAAATGAAACATCTCCAGCAACTGATAGAAGGAGCCCACAGAGAGATTGAGGATAAACCTGTTGCCACCAGTAACATACAGGAGCTGCAGGCTCAGATTTCTCGGCATGAG|  |  |  |  |  |  |  |  |  |  |  |  |  |  |  |  |  |  |  |  |  |  |  |  |  |  |  |  |  |  |  |  |  |  |  |  |  |  |  |  |  |  |  |  |  |  |  |  |  |  |  |  |  |  |  |  | | --- | --- | --- | --- | --- | --- | --- | --- | --- | --- | --- | --- | --- | --- | --- | --- | --- | --- | --- | --- | --- | --- | --- | --- | --- | --- | --- | --- | --- | --- | --- | --- | --- | --- | --- | --- | --- | --- | --- | --- | --- | --- | --- | --- | --- | --- | --- | --- | --- | --- | --- | --- | --- | --- | --- | --- | | Marmoset calJac1 Contig1075 252005 252144 - **GG** | CTGTGGTGCAGTATGAACAATATGAGCAAGAAATGAAGCATCTCCAGCAACTGATAGAAGGAGCTCACAGAGAGATTGAGGATAAACCTGTCGCCACCAGTAACATACAGGAGCTGCAAGCTCAGATTTCTCGGCATGAG|  |  |  |  |  |  |  |  |  |  |  |  |  |  |  |  |  |  |  |  |  |  |  |  |  |  |  |  |  |  |  |  |  |  |  |  |  |  |  |  |  |  |  |  |  |  |  |  | | --- | --- | --- | --- | --- | --- | --- | --- | --- | --- | --- | --- | --- | --- | --- | --- | --- | --- | --- | --- | --- | --- | --- | --- | --- | --- | --- | --- | --- | --- | --- | --- | --- | --- | --- | --- | --- | --- | --- | --- | --- | --- | --- | --- | --- | --- | --- | --- | | Tarsier tarSyr1 scaffold\_154 93285 93424 - **GG** | CTGTGGTGCAGTATGAACAATATGAGCAAGAAATGAAGCATCTTCAGCAGCTGATAGAAGGGGCTCACAGAGAGATTGAGGATAAGCCCGTGGCCACCAGTAATATCCAGGAGCTGCAAGCGCAGATCTCTCGACATGAG|  |  |  |  |  |  |  |  |  |  |  |  |  |  |  |  |  |  |  |  |  |  |  |  |  |  |  |  |  |  |  |  |  |  |  |  |  |  |  |  | | --- | --- | --- | --- | --- | --- | --- | --- | --- | --- | --- | --- | --- | --- | --- | --- | --- | --- | --- | --- | --- | --- | --- | --- | --- | --- | --- | --- | --- | --- | --- | --- | --- | --- | --- | --- | --- | --- | --- | --- | | Lemur micMur1 scaffold\_307 471491 471630 - **GG** | CAGTGGTGCAGTATGAACAATATGAACAAGAAATGAAACATCTCCAGCAACTGATAGAAGGAGCTCACAGGGAAATTGAGGATAAACCCGTGGCCACCAGTAACATCCAGGAGCTGCAAGCCCAGATTTCTCGGCATGAG|  |  |  |  |  |  |  |  |  |  |  |  |  |  |  |  |  |  |  |  |  |  |  |  |  |  |  |  |  |  |  |  | | --- | --- | --- | --- | --- | --- | --- | --- | --- | --- | --- | --- | --- | --- | --- | --- | --- | --- | --- | --- | --- | --- | --- | --- | --- | --- | --- | --- | --- | --- | --- | --- | | Galago otoGar1 scaffold\_85226.1-288156 46792 46931 - **GG** | CTGTGGTGCAGTATGAACAATATGAGCAAGAAATGAAGCATCTCCAGCAACTGATAGAAGGAGCCCACAGGGAAATTGAGGATAAACCCGTGGCCACCAGTAACATCCAGGAGCTGCAAGCACAGATTTCTCGGCACGAG|  |  |  |  |  |  |  |  |  |  |  |  |  |  |  |  |  |  |  |  |  |  |  |  | | --- | --- | --- | --- | --- | --- | --- | --- | --- | --- | --- | --- | --- | --- | --- | --- | --- | --- | --- | --- | --- | --- | --- | --- | | Mouse mm9 chr10 5144406 5144545 + **GG** | CTGTGGTGCAGTATGAACAGTACAAGCAGGAAATGAAGCATCTCCAGCAGCTGATAGAAGAGGCACACAGAGAGATCGAGGACAAACCCGTGGCCACCAGTAACATCCAGGAGCTACAGGCACAGATCTCTCTGCACGAG|  |  |  |  |  |  |  |  |  |  |  |  |  |  |  |  | | --- | --- | --- | --- | --- | --- | --- | --- | --- | --- | --- | --- | --- | --- | --- | --- | | Cow bosTau4 chr9 92876001 92876140 - **GG** | CTGTGGTGCAATATGAACAATATGAGCAAGAAATGCAGCACCTCCAGGAACTCATAGAAGGAGCTCACAGAGAGATTGAGGATAAGCCTGTGGCCACCAGCAACATCCAGGAACTGCAAGCCCAGATTTCTCGGCATGAG|  |  |  |  |  |  |  |  | | --- | --- | --- | --- | --- | --- | --- | --- | | Dog canFam2 chr1 45595291 45595430 - **GG** | CTGTGGTGCAATATGAACAGTATGAGCAAGAAATGCAGCACCTCCGGGAGCTTATAGAAGGAGCGCACAGAGAAATCGAGGATACACCTGTGGCCACCAGTAACATCCAGGAGCTGCAAGCCCAGATTTCTCGGCATGAG | | | | | | | | | | | | | | | | | | | | | | | | | | | | | | | | | | | | | | | | | | | | | | | | | | | | | | | | | | | | | | | | | | | | | | | | | | | | | | | | | | | | | | | | | | | | | | | | | | |

**Alignment** (splice site sequences are in lowercase)  

```
Human      agCTGTGGTACAATATGAACAATATGAGCAAGAAATGAAACATCTCCAGCAACTGATAGAAGGAGCTCACAGAGAGATTG
Chimp      g........G..G...................................................................
Gorilla    g........G..G...................................................................
Orangutan  g........G..G...................................................................
Rhesus     g........G..G.....................................................C.............
Baboon     g........G..G.....................................................C.............
Marmoset   g........G..G..........................G........................................
Tarsier    g........G..G..........................G.....T.....G...........G................
Lemur      g..A.....G..G..............A............................................G..A....
Galago     g........G..G..........................G..........................C.....G..A....
Mouse      g........G..G........G..CA....G........G...........G..........AG..A...........C.
Cow        g........G...........................C.G..C......G....C.........................
Dog        g........G...........G...............C.G..C....G.G.G..T...........G........A..C.

Human      AGGATAAACCTGTTGCCACCAGTAACATACAGGAGCTGCAGGCTCAGATTTCTCGGCATGAGgt
Chimp      ................................................................
Gorilla    ................................................................
Orangutan  ................................................................
Rhesus     ................................................................
Baboon     ................................................................
Marmoset   .............C..........................A.......................
Tarsier    .......G..C..G...........T..C...........A..G.....C.....A........
Lemur      ..........C..G..............C...........A..C....................
Galago     ..........C..G..............C...........A..A..............C.....
Mouse      ....C.....C..G..............C........A.....A.....C....T...C.....
Cow        .......G.....G........C.....C.....A.....A..C....................
Dog        ......C......G..............C...........A..C....................
```

---

## 20. uc003svh.2\_2\_4

**Summary**  

|  |  |  |  |  |  |  |  |  |  |  |  |  |  |  |  |  |  |  |  |  |  |  |  |  |  |
| --- | --- | --- | --- | --- | --- | --- | --- | --- | --- | --- | --- | --- | --- | --- | --- | --- | --- | --- | --- | --- | --- | --- | --- | --- | --- |
| No Exon ID Position (hg19) Dir Human acceptor Chimp acceptor Category Usage Gene symbol Protein accession mRNA accession Gene title Note|  |  |  |  |  |  |  |  |  |  |  |  |  | | --- | --- | --- | --- | --- | --- | --- | --- | --- | --- | --- | --- | --- | | 20 uc003svh.2\_2\_4 chr7:22533398 - AG AC (A3) shift; decrease; inframe alternative STEAP1B NP\_997225.1 NM\_207342.2 STEAP family member 1B  | | | | | | | | | | | | | | | | | | | | | | | | | |

**Orthologs**  

|  |  |  |  |  |  |  |  |  |  |  |  |  |  |  |  |  |  |  |  |  |  |  |  |  |  |  |  |  |  |  |  |  |  |  |  |  |  |  |  |
| --- | --- | --- | --- | --- | --- | --- | --- | --- | --- | --- | --- | --- | --- | --- | --- | --- | --- | --- | --- | --- | --- | --- | --- | --- | --- | --- | --- | --- | --- | --- | --- | --- | --- | --- | --- | --- | --- | --- | --- |
| Species Assembly Chromosome Exon start Exon end Dir Acceptor Exon sequence|  |  |  |  |  |  |  |  |  |  |  |  |  |  |  |  |  |  |  |  |  |  |  |  |  |  |  |  |  |  |  |  | | --- | --- | --- | --- | --- | --- | --- | --- | --- | --- | --- | --- | --- | --- | --- | --- | --- | --- | --- | --- | --- | --- | --- | --- | --- | --- | --- | --- | --- | --- | --- | --- | | Human hg19 chr7 22532943 22533398 - **AG** | CAAACAGCCCATGCTGATGAATTTGACTGCCCTTCAGAACTTCAGCACGCACAGGAACTCTTTCCACAGTGGCACTTGCCAATTAAAATAGCTGCTGTTATGGCATCTCTGACTTTTCTTTACACTCTTCTGAGGGAAGTAATTCACCCTTTAGCAACTTCCCATCAACAATATTTTTATAAAATTCCAATCCTGGTCATCAACAAAGTCTTGCCAATGGTTTCCATCACTCTCTTGGCATTGGTTTACCTACCAGGTGTGATAGCAGCAATTGTCCAAGTTCATAATGGAACCAAGTATAAGAAGTTTCCACATTGGTTGGATAAGTGGATGTTAACAAGAAAGCAGTTTGGGCTTCTCAGTTTGTTTTTTGCTGTACTGCATGCAATTTATACTCTGTCTTACGCAATGAGGCGATCCTACAGATACAAGTTGCTAAACTGGGCATATCAACAG|  |  |  |  |  |  |  |  |  |  |  |  |  |  |  |  |  |  |  |  |  |  |  |  | | --- | --- | --- | --- | --- | --- | --- | --- | --- | --- | --- | --- | --- | --- | --- | --- | --- | --- | --- | --- | --- | --- | --- | --- | | Chimp panTro2 chr7 22796436 22796891 - **AC** | CAAACAGCCCATGCTGATGAATTTGACTGCCCTTCAGAACTTCAGCACACACAGGAACTCTTTCCACAGTGGCACTTGCCAATTAAAATAGCTGCTATTTTAGCATCTCTGACTTTTCTTTACACTCTTCTGAGGGGAGTAATTCACCCTTTAGCAACTTCCCATCAACAATATTTTTATAAAATTCCAATCCTGGTCATCAACAAAGTCTTGCCAATGGTTTCCATCACTCTCTTGGCATTGGTTTACCTGCCAGGTGTGATAGCAGCAATTGTCCAACTTCATAATGGAACCAAGTATAAGAAGTTTCCACATTGGTTGGATAAATGGATGTTAACAAGAAAGCAGTTTGGGCTTCTCAGTTTCTTTTTTGCTGTACTGCATGCAATTTATAGTCTGTCTTACCCAATGAGGCGATCCTACAGATACAAGTTGCTAAACTGGGCATATCAACAG|  |  |  |  |  |  |  |  |  |  |  |  |  |  |  |  | | --- | --- | --- | --- | --- | --- | --- | --- | --- | --- | --- | --- | --- | --- | --- | --- | | Orangutan ponAbe2 chr7 86044111 86044566 - **AC** | CAGACAGCCCATGCTGATGAATTTGACTGTCCTTCAGAACTTCAGCAAACACGGGAACTCTTTCCACAGTGGCACTTGCCAATTAAAATAGCTGCTATTATAGCATCTCTGACTTTTCTTTACACTCTTCTGAGGGAAGTAATTCACCCTTTAGCAACTTCCCATCAACAATATTTTTATAAAATTCCAATCCTGGTCATCAACAAAGTCTTGCCAATGGTTTCCATCACTCTCTTGGCATTGGTTTACCTGCCAGGTGTGATAGCAGCAATTGTCCAGCTTCATAATGGAACCAAGTATAAGAAGTTTCCACATTGGTTAGATAAGTGGATGTTAACAAGAAAGCAGTTTGGGCTTCTCAGTTTCTTTTTTGCTGTACTGCATGCAATTTATAGTCTGTCTTACCCAATGAGGCGATCCTACAGATACAAGTTGCTAAACTGGGCATATCAACAG|  |  |  |  |  |  |  |  | | --- | --- | --- | --- | --- | --- | --- | --- | | Dog canFam2 chr14 19029283 19029738 + **AT** | CAAACAACCCACTTTGATGAATTTGATTGTCCTGCCGAGCTTAAGCACAAACAAGAACTCTTTCCAATGTGGCGCTGGCCAGTTAAAATTGCTGCTGTCATCTCATCTCTGACTTTTCTTTACACTCTTCTGAGGGAAATAATTCACCCTTTTGTAACTTCCCACCAACAGTATTTTTACAAAATTCCAATCCTGGTCATCAACAAAGTCTTGCCAATGGTTTCCATCACCCTCTTGGCACTAGTTTATTTGCCAGGCGTGATAGCAGCTGTTGTACAGCTTCATAATGGAACCAAATATAAGAAATTTCCACATTGGTTGGATAGATGGATGTTAACAAGAAAACAATTTGGGCTTCTCAGTTTCTTTTTTGCTGTCCTGCATGCAATTTACAGTTTATCCTATCCAATGAGGCGATCCTACAGATACAAGTTGCTAAACTGGGCATATCAACAG | | | | | | | | | | | | | | | | | | | | | | | | | | | | | | | | | | | |

**Alignment** (splice site sequences are in lowercase)  

```
Human      agCAAACAGCCCATGCTGATGAATTTGACTGCCCTTCAGAACTTCAGCACGCACAGGAACTCTTTCCACAGTGGCACTTG
Chimp      .c................................................A.............................
Orangutan  .c..G..........................T.................AA...G.........................
Dog        .t......A....CTT............T..T...G.C..G...A.....AA...A............AT.....G..G.

Human      CCAATTAAAATAGCTGCTGTTATGGCATCTCTGACTTTTCTTTACACTCTTCTGAGGGAAGTAATTCACCCTTTAGCAAC
Chimp      ..................A..T.A..................................G.....................
Orangutan  ..................A....A........................................................
Dog        ...G.......T........C..CT...................................A.............T.T...

Human      TTCCCATCAACAATATTTTTATAAAATTCCAATCCTGGTCATCAACAAAGTCTTGCCAATGGTTTCCATCACTCTCTTGG
Chimp      ................................................................................
Orangutan  ................................................................................
Dog        ......C.....G........C..................................................C.......

Human      CATTGGTTTACCTACCAGGTGTGATAGCAGCAATTGTCCAAGTTCATAATGGAACCAAGTATAAGAAGTTTCCACATTGG
Chimp      .............G...........................C......................................
Orangutan  .............G..........................GC......................................
Dog        ..C.A.....TT.G.....C...........TG....A..GC................A........A............

Human      TTGGATAAGTGGATGTTAACAAGAAAGCAGTTTGGGCTTCTCAGTTTGTTTTTTGCTGTACTGCATGCAATTTATACTCT
Chimp      ........A......................................C............................G...
Orangutan  ..A............................................C............................G...
Dog        .......GA.................A..A.................C...........C..............C.G.T.

Human      GTCTTACGCAATGAGGCGATCCTACAGATACAAGTTGCTAAACTGGGCATATCAACAGgt
Chimp      .......C....................................................
Orangutan  .......C....................................................
Dog        A..C..TC....................................................
```

---

## 21. uc003wia.1\_3\_4

**Summary**  

|  |  |  |  |  |  |  |  |  |  |  |  |  |  |  |  |  |  |  |  |  |  |  |  |  |  |
| --- | --- | --- | --- | --- | --- | --- | --- | --- | --- | --- | --- | --- | --- | --- | --- | --- | --- | --- | --- | --- | --- | --- | --- | --- | --- |
| No Exon ID Position (hg19) Dir Human acceptor Chimp acceptor Category Usage Gene symbol Protein accession mRNA accession Gene title Note|  |  |  |  |  |  |  |  |  |  |  |  |  | | --- | --- | --- | --- | --- | --- | --- | --- | --- | --- | --- | --- | --- | | 21 uc003wia.1\_3\_4 chr7:150557532 + AG GG (A1) shift; increase; inframe alternative ABP1 P19801-2 U11863.1 amiloride-sensitive amine oxidase [copper-containing]  | | | | | | | | | | | | | | | | | | | | | | | | | |

**Orthologs**  

|  |  |  |  |  |  |  |  |  |  |  |  |  |  |  |  |  |  |  |  |  |  |  |  |  |  |  |  |  |  |  |  |  |  |  |  |  |  |  |  |  |  |  |  |  |  |  |  |  |  |  |  |  |  |  |  |  |  |  |  |  |  |  |  |
| --- | --- | --- | --- | --- | --- | --- | --- | --- | --- | --- | --- | --- | --- | --- | --- | --- | --- | --- | --- | --- | --- | --- | --- | --- | --- | --- | --- | --- | --- | --- | --- | --- | --- | --- | --- | --- | --- | --- | --- | --- | --- | --- | --- | --- | --- | --- | --- | --- | --- | --- | --- | --- | --- | --- | --- | --- | --- | --- | --- | --- | --- | --- | --- |
| Species Assembly Chromosome Exon start Exon end Dir Acceptor Exon sequence|  |  |  |  |  |  |  |  |  |  |  |  |  |  |  |  |  |  |  |  |  |  |  |  |  |  |  |  |  |  |  |  |  |  |  |  |  |  |  |  |  |  |  |  |  |  |  |  |  |  |  |  |  |  |  |  | | --- | --- | --- | --- | --- | --- | --- | --- | --- | --- | --- | --- | --- | --- | --- | --- | --- | --- | --- | --- | --- | --- | --- | --- | --- | --- | --- | --- | --- | --- | --- | --- | --- | --- | --- | --- | --- | --- | --- | --- | --- | --- | --- | --- | --- | --- | --- | --- | --- | --- | --- | --- | --- | --- | --- | --- | | Human hg19 chr7 150557532 150557721 + **AG** | GACTGAGGGGGGCCAGCCCAGGGCCCTGAGCCAAGCTGCTTCGCCTGTGCCTGGCAGGTACCCCCTGGCAGTGACCAAGTACCGGGAGTCGGAGCTGTGCAGCAGCAGCATCTACCACCAGAACGACCCCTGGCACCCGCCCGTGGTCTTTGAGCAGTTTCTTCACAACAACGAGAACATTGAAAATGAG|  |  |  |  |  |  |  |  |  |  |  |  |  |  |  |  |  |  |  |  |  |  |  |  |  |  |  |  |  |  |  |  |  |  |  |  |  |  |  |  |  |  |  |  |  |  |  |  | | --- | --- | --- | --- | --- | --- | --- | --- | --- | --- | --- | --- | --- | --- | --- | --- | --- | --- | --- | --- | --- | --- | --- | --- | --- | --- | --- | --- | --- | --- | --- | --- | --- | --- | --- | --- | --- | --- | --- | --- | --- | --- | --- | --- | --- | --- | --- | --- | | Chimp panTro2 chr7 151382781 151382970 + **GG** | GACTGAGGGGGGCCAGCCCAGGGCCCTGAGCCAAGCTGCTTCACCTCTGCCTGGCAGGTACCCCCTGGCAGTGACCAAGTACCGGGAGTCGGAGCTGTGCAGCAGCAGCATCTACCACCAGAACGACCCCTGGGACCCGCCCGTGGTCTTCGAGAAGTTTCTTCACAACAACGAGAACATTGAAAATGAG|  |  |  |  |  |  |  |  |  |  |  |  |  |  |  |  |  |  |  |  |  |  |  |  |  |  |  |  |  |  |  |  |  |  |  |  |  |  |  |  | | --- | --- | --- | --- | --- | --- | --- | --- | --- | --- | --- | --- | --- | --- | --- | --- | --- | --- | --- | --- | --- | --- | --- | --- | --- | --- | --- | --- | --- | --- | --- | --- | --- | --- | --- | --- | --- | --- | --- | --- | | Gorilla gorGor1 Supercontig\_0006103 1775 1964 - **GG** | GACTGAGGGGGGCCAGCCCAGGGCCCTGAGCCAAGCTGCTTCGCCTCTGCCTGGCAGGTACCCCCTGGCAGTGACCAAGTACCGGGAGTCGGAGCTGTGCAGCAGCAGCATCTACCACCAGAACGACCCCTGGGACCCGCCCGTGGTCTTCGAGAAGTTTCTTCACAACAACGAGAACATTGAAAATGAG|  |  |  |  |  |  |  |  |  |  |  |  |  |  |  |  |  |  |  |  |  |  |  |  |  |  |  |  |  |  |  |  | | --- | --- | --- | --- | --- | --- | --- | --- | --- | --- | --- | --- | --- | --- | --- | --- | --- | --- | --- | --- | --- | --- | --- | --- | --- | --- | --- | --- | --- | --- | --- | --- | | Orangutan ponAbe2 chr7 148538131 148538320 + **GG** | GGCTGAGGGGGGCCAGCCCAGGGCCCTGAGCCAAGCTGCTTCACCTCTGCCTCGCAGGTACCCCCTGGCAGTGACCAAGTACCGGGAATCGGAGCTGTGCAGCAGCAGCATCTACCACCAGAACGACCCCTGGGACCCGCCCGTGGTCTTTGAGAAGTTTCTTCACAACAATGAGAACATTGAAAATGAG|  |  |  |  |  |  |  |  |  |  |  |  |  |  |  |  |  |  |  |  |  |  |  |  | | --- | --- | --- | --- | --- | --- | --- | --- | --- | --- | --- | --- | --- | --- | --- | --- | --- | --- | --- | --- | --- | --- | --- | --- | | Rhesus rheMac2 chr3 187813289 187813478 + **GG** | GGCTAAGGGGGGCCAGCCCAGGCCCCTGAGCCAAGCTGCTTTGCCTCTGCCTGGCAGGTACCCCCTGGCAGTGACCAAGTACCGGGAGTCAGAGCTGTGCAGCAGCAGCATCTACCACCAGAACGACCCCTGGGACCCGCCCGTGGTCTTTGAGAAGTTTCTTCACAACAATGAGAACATTGAAAATGAG|  |  |  |  |  |  |  |  |  |  |  |  |  |  |  |  | | --- | --- | --- | --- | --- | --- | --- | --- | --- | --- | --- | --- | --- | --- | --- | --- | | Baboon papHam1 scaffold1705 160884 161073 - **GG** | GGCTAAGGGGGTCCAGCCCAGGCCCCTGAGCCAAGCTGCTTCGCCTCTGCCTGGCAGGTACCCCCTGGCAGTGACCAAGTACCGGGAGTCAGAGCTGTGCAGCAGCAGCATCTACCACCAGAACGACCCCTGGGACCCGCCCGTGGTCTTTGAGAAGTTTCTTCACAACAATGAGAACATTGAAAATGAG|  |  |  |  |  |  |  |  | | --- | --- | --- | --- | --- | --- | --- | --- | | Marmoset calJac1 Contig11321 36821 37008 + **GG** | GGCTGGGGACCCAGCCCAGGGCCCTGAGCCTGGCTGCTTCGCCTCTGCGTGGCAGGTACCCCCTGGCAGTGACCAGGTACCGGGAGTCTGAGCTGTGCAGCAGCAGCATCTACAACCAGAACGACCCGTGGGACCCACCCGTGGTCTTTGAGGAGTTTCTTCACAACAATGAGAACATTGAAAACCAG | | | | | | | | | | | | | | | | | | | | | | | | | | | | | | | | | | | | | | | | | | | | | | | | | | | | | | | | |

**Alignment** (splice site sequences are in lowercase)  

```
Human      agGACTGAGGGGGGCCAGCCCAGGGCCCTGAGCCAAGCTGCTTCGCCTGTGCCTGGCAGGTACCCCCTGGCAGTGACCAA
Chimp      g...........................................A...C...............................
Gorilla    g...............................................C...............................
Orangutan  g..G........................................A...C.....C.........................
Rhesus     g..G..A.................C..................T....C...............................
Baboon     g..G..A......T..........C.......................C...............................
Marmoset   g..G..--....AC....................TG............C...G..........................G

Human      GTACCGGGAGTCGGAGCTGTGCAGCAGCAGCATCTACCACCAGAACGACCCCTGGCACCCGCCCGTGGTCTTTGAGCAGT
Chimp      .......................................................G................C...A...
Gorilla    .......................................................G................C...A...
Orangutan  .........A.............................................G....................A...
Rhesus     ............A..........................................G....................A...
Baboon     ............A..........................................G....................A...
Marmoset   ............T........................A.............G...G....A...............G...

Human      TTCTTCACAACAACGAGAACATTGAAAATGAGgt
Chimp      ..................................
Gorilla    ..................................
Orangutan  .............T....................
Rhesus     .............T....................
Baboon     .............T....................
Marmoset   .............T..............CC....
```

---

## 22. uc004amn.2\_3\_4

**Summary**  

|  |  |  |  |  |  |  |  |  |  |  |  |  |  |  |  |  |  |  |  |  |  |  |  |  |  |
| --- | --- | --- | --- | --- | --- | --- | --- | --- | --- | --- | --- | --- | --- | --- | --- | --- | --- | --- | --- | --- | --- | --- | --- | --- | --- |
| No Exon ID Position (hg19) Dir Human acceptor Chimp acceptor Category Usage Gene symbol Protein accession mRNA accession Gene title Note|  |  |  |  |  |  |  |  |  |  |  |  |  | | --- | --- | --- | --- | --- | --- | --- | --- | --- | --- | --- | --- | --- | | 22 uc004amn.2\_3\_4 chr9:84605332 + AG TG (A5) exonization; inframe alternative FAM75D1 NP\_001001670.1 NM\_001001670.2 family with sequence similarity 75, member D1  | | | | | | | | | | | | | | | | | | | | | | | | | |

**Orthologs**  

|  |  |  |  |  |  |  |  |  |  |  |  |  |  |  |  |  |  |  |  |  |  |  |  |  |  |  |  |  |  |  |  |  |  |  |  |  |  |  |  |  |  |  |  |  |  |  |  |  |  |  |  |  |  |  |  |  |  |  |  |  |  |  |  |  |  |  |  |  |  |  |  |  |  |  |  |  |  |  |  |
| --- | --- | --- | --- | --- | --- | --- | --- | --- | --- | --- | --- | --- | --- | --- | --- | --- | --- | --- | --- | --- | --- | --- | --- | --- | --- | --- | --- | --- | --- | --- | --- | --- | --- | --- | --- | --- | --- | --- | --- | --- | --- | --- | --- | --- | --- | --- | --- | --- | --- | --- | --- | --- | --- | --- | --- | --- | --- | --- | --- | --- | --- | --- | --- | --- | --- | --- | --- | --- | --- | --- | --- | --- | --- | --- | --- | --- | --- | --- | --- |
| Species Assembly Chromosome Exon start Exon end Dir Acceptor Exon sequence|  |  |  |  |  |  |  |  |  |  |  |  |  |  |  |  |  |  |  |  |  |  |  |  |  |  |  |  |  |  |  |  |  |  |  |  |  |  |  |  |  |  |  |  |  |  |  |  |  |  |  |  |  |  |  |  |  |  |  |  |  |  |  |  |  |  |  |  |  |  |  |  | | --- | --- | --- | --- | --- | --- | --- | --- | --- | --- | --- | --- | --- | --- | --- | --- | --- | --- | --- | --- | --- | --- | --- | --- | --- | --- | --- | --- | --- | --- | --- | --- | --- | --- | --- | --- | --- | --- | --- | --- | --- | --- | --- | --- | --- | --- | --- | --- | --- | --- | --- | --- | --- | --- | --- | --- | --- | --- | --- | --- | --- | --- | --- | --- | --- | --- | --- | --- | --- | --- | --- | --- | | Human hg19 chr9 84605332 84605401 + **AG** | GTTTCCCAGACTGGAAAAGTTTCCAGAGAGAAGAGGAAGAGGAAAGGAAGCTGCTTTCTCTTCTGAAAAG|  |  |  |  |  |  |  |  |  |  |  |  |  |  |  |  |  |  |  |  |  |  |  |  |  |  |  |  |  |  |  |  |  |  |  |  |  |  |  |  |  |  |  |  |  |  |  |  |  |  |  |  |  |  |  |  |  |  |  |  |  |  |  |  | | --- | --- | --- | --- | --- | --- | --- | --- | --- | --- | --- | --- | --- | --- | --- | --- | --- | --- | --- | --- | --- | --- | --- | --- | --- | --- | --- | --- | --- | --- | --- | --- | --- | --- | --- | --- | --- | --- | --- | --- | --- | --- | --- | --- | --- | --- | --- | --- | --- | --- | --- | --- | --- | --- | --- | --- | --- | --- | --- | --- | --- | --- | --- | --- | | Chimp panTro2 chr9 80947928 80947997 + **TG** | GTCTCCCAGACTGGAAAAGTTTCCAGAGAGAAGAGGAAGAGGAAAGGAAGCTGCTTTCTCTTCTGAAAAG|  |  |  |  |  |  |  |  |  |  |  |  |  |  |  |  |  |  |  |  |  |  |  |  |  |  |  |  |  |  |  |  |  |  |  |  |  |  |  |  |  |  |  |  |  |  |  |  |  |  |  |  |  |  |  |  | | --- | --- | --- | --- | --- | --- | --- | --- | --- | --- | --- | --- | --- | --- | --- | --- | --- | --- | --- | --- | --- | --- | --- | --- | --- | --- | --- | --- | --- | --- | --- | --- | --- | --- | --- | --- | --- | --- | --- | --- | --- | --- | --- | --- | --- | --- | --- | --- | --- | --- | --- | --- | --- | --- | --- | --- | | Gorilla gorGor1 Supercontig\_0006951 17458 17527 - **TG** | GTTTCCCAGACTGGAAAAGTTTCCAGAGAGAAGAGGAAGAGGAAAGGAAGCTGCTTTCTCTTCTGAAAAG|  |  |  |  |  |  |  |  |  |  |  |  |  |  |  |  |  |  |  |  |  |  |  |  |  |  |  |  |  |  |  |  |  |  |  |  |  |  |  |  |  |  |  |  |  |  |  |  | | --- | --- | --- | --- | --- | --- | --- | --- | --- | --- | --- | --- | --- | --- | --- | --- | --- | --- | --- | --- | --- | --- | --- | --- | --- | --- | --- | --- | --- | --- | --- | --- | --- | --- | --- | --- | --- | --- | --- | --- | --- | --- | --- | --- | --- | --- | --- | --- | | Rhesus rheMac2 chr15 90746862 90746931 + **TG** | GTTTCCCAGACCGGAAATGTTTCCAGAGAGAAGTGGAAGAGGAAAGGAAGCTGCTTTTTATTCTGAAAAG|  |  |  |  |  |  |  |  |  |  |  |  |  |  |  |  |  |  |  |  |  |  |  |  |  |  |  |  |  |  |  |  |  |  |  |  |  |  |  |  | | --- | --- | --- | --- | --- | --- | --- | --- | --- | --- | --- | --- | --- | --- | --- | --- | --- | --- | --- | --- | --- | --- | --- | --- | --- | --- | --- | --- | --- | --- | --- | --- | --- | --- | --- | --- | --- | --- | --- | --- | | Marmoset calJac1 Contig8907 51928 51997 - **TG** | GTTTCCCAGACCAGATAACCTTCCAGAAAGCAGCAGAAGAGGAAAGGAAGCTGGTTTCTTTTCTGAAAAG|  |  |  |  |  |  |  |  |  |  |  |  |  |  |  |  |  |  |  |  |  |  |  |  |  |  |  |  |  |  |  |  | | --- | --- | --- | --- | --- | --- | --- | --- | --- | --- | --- | --- | --- | --- | --- | --- | --- | --- | --- | --- | --- | --- | --- | --- | --- | --- | --- | --- | --- | --- | --- | --- | | Tarsier tarSyr1 scaffold\_67653 4938 5007 - **TG** | GCTCCGCAGGTAGGACATCTGGCCAGAGTGAAGTGAAGGAGGCGAGGAGGCTGGTTTCTCTTCTGCAGAG|  |  |  |  |  |  |  |  |  |  |  |  |  |  |  |  |  |  |  |  |  |  |  |  | | --- | --- | --- | --- | --- | --- | --- | --- | --- | --- | --- | --- | --- | --- | --- | --- | --- | --- | --- | --- | --- | --- | --- | --- | | Galago otoGar1 scaffold\_84730.1-372351 245735 245804 + **TG** | GTTTCCTAGGTTGGAGAATTTGCCAGAGAGAAGCAGAAGTGGGAAAGAAGCTGCTTTCTGTTCTGGAAAA|  |  |  |  |  |  |  |  |  |  |  |  |  |  |  |  | | --- | --- | --- | --- | --- | --- | --- | --- | --- | --- | --- | --- | --- | --- | --- | --- | | Cow bosTau4 chr8 60397618 60397687 + **TG** | GTTTCGCAGGTTGGAGATTTCACCAGATAGAAACAGAGGAAAAAACGAAGCTGATTTCTATTCTGAAAAG|  |  |  |  |  |  |  |  | | --- | --- | --- | --- | --- | --- | --- | --- | | Dog canFam2 chr1 80396219 80396285 - **TG** | GTTTCCCAGACTACAGATGCTACCAGGGAGAAGTGGAGAAGACACGGAAGCTTCTCTCTGTTTTAAG | | | | | | | | | | | | | | | | | | | | | | | | | | | | | | | | | | | | | | | | | | | | | | | | | | | | | | | | | | | | | | | | | | | | | | |

**Alignment** (splice site sequences are in lowercase)  

```
Human      agGTTTCCCAGACTGGAAAAGTTTCCAGAGAGAAGAGGAAGAGGAAAGGAAGCTGCTTTCTCTTCTGAAAAGgt
Chimp      t...C.....................................................................
Gorilla    t.........................................................................
Rhesus     t............C.....T...............T.......................T.A............
Marmoset   t............CA..T..CC.......A..C..CA..................G.....T............
Tarsier    t..C.C.G...GTA...C.TC.GG......T....T.A.G....CG....G....G...........C.G....
Galago     t.......T..GT....G..T..G...........CA....T..G..A.............G.....G...A..
Cow        t......G...GT....G.TT.CA.....T....ACA..G..AA...C.......A.....A............
Dog        t.............AC.G.T.C.A....G......T...GA..AC.C.......T..C...G..T.---.....
```

---

## 23. uc001nyl.2\_9\_14

**Summary**  

|  |  |  |  |  |  |  |  |  |  |  |  |  |  |  |  |  |  |  |  |  |  |  |  |  |  |
| --- | --- | --- | --- | --- | --- | --- | --- | --- | --- | --- | --- | --- | --- | --- | --- | --- | --- | --- | --- | --- | --- | --- | --- | --- | --- |
| No Exon ID Position (hg19) Dir Human acceptor Chimp acceptor Category Usage Gene symbol Protein accession mRNA accession Gene title Note|  |  |  |  |  |  |  |  |  |  |  |  |  | | --- | --- | --- | --- | --- | --- | --- | --- | --- | --- | --- | --- | --- | | 23 uc001nyl.2\_9\_14 chr11:63987351 + AG CG (A1) shift; increase; inframe alternative FERMT3 NP\_848537.1 NM\_178443.2 fermitin family homolog 3 long form  | | | | | | | | | | | | | | | | | | | | | | | | | |

**Orthologs**  

|  |  |  |  |  |  |  |  |  |  |  |  |  |  |  |  |  |  |  |  |  |  |  |  |  |  |  |  |  |  |  |  |  |  |  |  |  |  |  |  |  |  |  |  |  |  |  |  |  |  |  |  |  |  |  |  |  |  |  |  |  |  |  |  |  |  |  |  |  |  |  |  |
| --- | --- | --- | --- | --- | --- | --- | --- | --- | --- | --- | --- | --- | --- | --- | --- | --- | --- | --- | --- | --- | --- | --- | --- | --- | --- | --- | --- | --- | --- | --- | --- | --- | --- | --- | --- | --- | --- | --- | --- | --- | --- | --- | --- | --- | --- | --- | --- | --- | --- | --- | --- | --- | --- | --- | --- | --- | --- | --- | --- | --- | --- | --- | --- | --- | --- | --- | --- | --- | --- | --- | --- |
| Species Assembly Chromosome Exon start Exon end Dir Acceptor Exon sequence|  |  |  |  |  |  |  |  |  |  |  |  |  |  |  |  |  |  |  |  |  |  |  |  |  |  |  |  |  |  |  |  |  |  |  |  |  |  |  |  |  |  |  |  |  |  |  |  |  |  |  |  |  |  |  |  |  |  |  |  |  |  |  |  | | --- | --- | --- | --- | --- | --- | --- | --- | --- | --- | --- | --- | --- | --- | --- | --- | --- | --- | --- | --- | --- | --- | --- | --- | --- | --- | --- | --- | --- | --- | --- | --- | --- | --- | --- | --- | --- | --- | --- | --- | --- | --- | --- | --- | --- | --- | --- | --- | --- | --- | --- | --- | --- | --- | --- | --- | --- | --- | --- | --- | --- | --- | --- | --- | | Human hg19 chr11 63987351 63987487 + **AG** | CATCCCACGAAGGCCCCGGAAGCTGACCCTGAAGGGCTACCGCCAACACTGGGTGGTGTTCAAGGAGACCACACTGTCCTACTACAAGAGCCAGGACGAGGCCCCTGGGGACCCCATTCAGCAGCTCAACCTCAAGG|  |  |  |  |  |  |  |  |  |  |  |  |  |  |  |  |  |  |  |  |  |  |  |  |  |  |  |  |  |  |  |  |  |  |  |  |  |  |  |  |  |  |  |  |  |  |  |  |  |  |  |  |  |  |  |  | | --- | --- | --- | --- | --- | --- | --- | --- | --- | --- | --- | --- | --- | --- | --- | --- | --- | --- | --- | --- | --- | --- | --- | --- | --- | --- | --- | --- | --- | --- | --- | --- | --- | --- | --- | --- | --- | --- | --- | --- | --- | --- | --- | --- | --- | --- | --- | --- | --- | --- | --- | --- | --- | --- | --- | --- | | Chimp panTro2 chr11 62616928 62617064 + **CG** | CATCCCACGAAGGCCCCGGAACTTTACCCTGAAGGGCTACCGCCAACACTGGGTGGTGTTCAAGGAGACCACACTGTCCTACTACAAGAGCCAGGACGAGGCCCCCGGGGACCCCATTCAGCAGCTCAACCTCAAGG|  |  |  |  |  |  |  |  |  |  |  |  |  |  |  |  |  |  |  |  |  |  |  |  |  |  |  |  |  |  |  |  |  |  |  |  |  |  |  |  |  |  |  |  |  |  |  |  | | --- | --- | --- | --- | --- | --- | --- | --- | --- | --- | --- | --- | --- | --- | --- | --- | --- | --- | --- | --- | --- | --- | --- | --- | --- | --- | --- | --- | --- | --- | --- | --- | --- | --- | --- | --- | --- | --- | --- | --- | --- | --- | --- | --- | --- | --- | --- | --- | | Gorilla gorGor1 Supercontig\_0003388 60994 61130 + **CG** | CATCCCACGAAGGCCCCGGAAGCTGACCCTGAAGGGCTACCGCCAACACTGGGTGGTGTTCAAGGAGACCACACTGTCCTACTACAAGAGCCAGGACGAGGCCCCTGGGGACCCCATTCAGCAGCTCAACCTCAAGG|  |  |  |  |  |  |  |  |  |  |  |  |  |  |  |  |  |  |  |  |  |  |  |  |  |  |  |  |  |  |  |  |  |  |  |  |  |  |  |  | | --- | --- | --- | --- | --- | --- | --- | --- | --- | --- | --- | --- | --- | --- | --- | --- | --- | --- | --- | --- | --- | --- | --- | --- | --- | --- | --- | --- | --- | --- | --- | --- | --- | --- | --- | --- | --- | --- | --- | --- | | Orangutan ponAbe2 chr11 11747146 11747282 - **CG** | CATCCCACGAAGGCCCCGGAAGCTGACCCTGAAGGGCTACCGCCAGCACTGGGTGGTGTTCAAGGAGACCACACTGTCCTACTACAAGAGTCAGGACGAGGCTCCTGGGGACCCCATTCAGCAGCTCAACCTCAAGG|  |  |  |  |  |  |  |  |  |  |  |  |  |  |  |  |  |  |  |  |  |  |  |  |  |  |  |  |  |  |  |  | | --- | --- | --- | --- | --- | --- | --- | --- | --- | --- | --- | --- | --- | --- | --- | --- | --- | --- | --- | --- | --- | --- | --- | --- | --- | --- | --- | --- | --- | --- | --- | --- | | Rhesus rheMac2 chr14 10208875 10209011 - **CA** | CACCCCATGAAGGCCCCGGAAGCTGACCCTGAAGGGCTACCGCCAGCACTGGGTGGTGTTCAAGGAGACTACACTGTCCTACTACAAGAGCCAGGATGAGGCCCCTGGGGACCCCATTCAGCAGCTCAACCTCAAGG|  |  |  |  |  |  |  |  |  |  |  |  |  |  |  |  |  |  |  |  |  |  |  |  | | --- | --- | --- | --- | --- | --- | --- | --- | --- | --- | --- | --- | --- | --- | --- | --- | --- | --- | --- | --- | --- | --- | --- | --- | | Baboon papHam1 scaffold14283 41982 42118 + **CA** | CACCCCATGAAGGCCCCGGAAGCTGACCCTGAAGGGCTACCGCCAGCACTGGGTGGTGTTCAAGGAGACTACACTGTCCTACTACAAGAGCCAGGATGAGGCCCCTGGGGACCCCATTCAGCAGCTCAACCTCAAGG|  |  |  |  |  |  |  |  |  |  |  |  |  |  |  |  | | --- | --- | --- | --- | --- | --- | --- | --- | --- | --- | --- | --- | --- | --- | --- | --- | | Marmoset calJac1 Contig16940 7554 7690 + **CG** | AATTCCACAAAGGCCCCGGAAGCTGACCCTGAAGGGCTACCGCCAGCACTGGGTGGTGTTCAAGGAGACCACACTGTCCTACTACAAGAACCAGGACGAGGCCCCGGGTGACCCCATCCAGCAGCTCAACCTCAAAG|  |  |  |  |  |  |  |  | | --- | --- | --- | --- | --- | --- | --- | --- | | Dog canFam2 chr18 55862358 55862494 - **TG** | CCTCGCCTTCAGGCCCCGGAAGCTGACCCTAAAGGGGTATCGCCAACACTGGGTGGTGTTCAAGGAGACCACCCTGTCCTACTACAAGAGCCAGGATGAGGCCCCAGGGGACCCCATTCAGCAGCTCAACCTCAAGG | | | | | | | | | | | | | | | | | | | | | | | | | | | | | | | | | | | | | | | | | | | | | | | | | | | | | | | | | | | | | | | |

**Alignment** (splice site sequences are in lowercase)  

```
Human      agCATCCCACGAAGGCCCCGGAAGCTGACCCTGAAGGGCTACCGCCAACACTGGGTGGTGTTCAAGGAGACCACACTGTC
Chimp      c......................CT.T.....................................................
Gorilla    c...............................................................................
Orangutan  c..............................................G................................
Rhesus     ca..C....T.....................................G.......................T........
Baboon     ca..C....T.....................................G.......................T........
Marmoset   c.A..T....A....................................G................................
Dog        t..C..G.CTTC....................A.....G..T................................C.....

Human      CTACTACAAGAGCCAGGACGAGGCCCCTGGGGACCCCATTCAGCAGCTCAACCTCAAGGgt
Chimp      ...........................C.................................
Gorilla    .............................................................
Orangutan  ............T...........T....................................
Rhesus     ..................T..........................................
Baboon     ..................T..........................................
Marmoset   ...........A...............G..T........C.................A...
Dog        ..................T........A.................................
```

---

## 24. uc001nzq.1\_4\_6

**Summary**  

|  |  |  |  |  |  |  |  |  |  |  |  |  |  |  |  |  |  |  |  |  |  |  |  |  |  |
| --- | --- | --- | --- | --- | --- | --- | --- | --- | --- | --- | --- | --- | --- | --- | --- | --- | --- | --- | --- | --- | --- | --- | --- | --- | --- |
| No Exon ID Position (hg19) Dir Human acceptor Chimp acceptor Category Usage Gene symbol Protein accession mRNA accession Gene title Note|  |  |  |  |  |  |  |  |  |  |  |  |  | | --- | --- | --- | --- | --- | --- | --- | --- | --- | --- | --- | --- | --- | | 24 uc001nzq.1\_4\_6 chr11:64082213 + AG CG (A1) shift; increase; inframe alternative ESRRA NP\_004442.3 NM\_004451.3 steroid hormone receptor ERR1 NAGNAG | | | | | | | | | | | | | | | | | | | | | | | | | |

**Orthologs**  

|  |  |  |  |  |  |  |  |  |  |  |  |  |  |  |  |  |  |  |  |  |  |  |  |  |  |  |  |  |  |  |  |  |  |  |  |  |  |  |  |  |  |  |  |  |  |  |  |  |  |  |  |  |  |  |  |  |  |  |  |  |  |  |  |  |  |  |  |  |  |  |  |  |  |  |  |  |  |  |  |  |  |  |  |  |  |  |  |  |  |  |  |  |  |  |  |
| --- | --- | --- | --- | --- | --- | --- | --- | --- | --- | --- | --- | --- | --- | --- | --- | --- | --- | --- | --- | --- | --- | --- | --- | --- | --- | --- | --- | --- | --- | --- | --- | --- | --- | --- | --- | --- | --- | --- | --- | --- | --- | --- | --- | --- | --- | --- | --- | --- | --- | --- | --- | --- | --- | --- | --- | --- | --- | --- | --- | --- | --- | --- | --- | --- | --- | --- | --- | --- | --- | --- | --- | --- | --- | --- | --- | --- | --- | --- | --- | --- | --- | --- | --- | --- | --- | --- | --- | --- | --- | --- | --- | --- | --- | --- | --- |
| Species Assembly Chromosome Exon start Exon end Dir Acceptor Exon sequence|  |  |  |  |  |  |  |  |  |  |  |  |  |  |  |  |  |  |  |  |  |  |  |  |  |  |  |  |  |  |  |  |  |  |  |  |  |  |  |  |  |  |  |  |  |  |  |  |  |  |  |  |  |  |  |  |  |  |  |  |  |  |  |  |  |  |  |  |  |  |  |  |  |  |  |  |  |  |  |  |  |  |  |  |  |  |  |  | | --- | --- | --- | --- | --- | --- | --- | --- | --- | --- | --- | --- | --- | --- | --- | --- | --- | --- | --- | --- | --- | --- | --- | --- | --- | --- | --- | --- | --- | --- | --- | --- | --- | --- | --- | --- | --- | --- | --- | --- | --- | --- | --- | --- | --- | --- | --- | --- | --- | --- | --- | --- | --- | --- | --- | --- | --- | --- | --- | --- | --- | --- | --- | --- | --- | --- | --- | --- | --- | --- | --- | --- | --- | --- | --- | --- | --- | --- | --- | --- | --- | --- | --- | --- | --- | --- | --- | --- | | Human hg19 chr11 64082213 64082383 + **AG** | CAGCCCCAGTGAATGCACTGGTGTCTCATCTGCTGGTGGTTGAGCCTGAGAAGCTCTATGCCATGCCTGACCCCGCAGGCCCTGATGGGCACCTCCCAGCCGTGGCTACCCTCTGTGACCTCTTTGACCGAGAGATTGTGGTCACCATCAGCTGGGCCAAGAGCATCCCAG|  |  |  |  |  |  |  |  |  |  |  |  |  |  |  |  |  |  |  |  |  |  |  |  |  |  |  |  |  |  |  |  |  |  |  |  |  |  |  |  |  |  |  |  |  |  |  |  |  |  |  |  |  |  |  |  |  |  |  |  |  |  |  |  |  |  |  |  |  |  |  |  |  |  |  |  |  |  |  |  | | --- | --- | --- | --- | --- | --- | --- | --- | --- | --- | --- | --- | --- | --- | --- | --- | --- | --- | --- | --- | --- | --- | --- | --- | --- | --- | --- | --- | --- | --- | --- | --- | --- | --- | --- | --- | --- | --- | --- | --- | --- | --- | --- | --- | --- | --- | --- | --- | --- | --- | --- | --- | --- | --- | --- | --- | --- | --- | --- | --- | --- | --- | --- | --- | --- | --- | --- | --- | --- | --- | --- | --- | --- | --- | --- | --- | --- | --- | --- | --- | | Chimp panTro2 chr11 62717602 62717772 + **CG** | CAGCCCCAGTGAATGCACTGGTGTCTCATCTGCTGGTGGTTGAGCCTGAGAAGCTCTATGCCATGCCTGACCCCGCAGGCCCTGATGGGCACCTCCCAGCCGTGGCTACCCTCTGTGACCTCTTTGACCGAGAGATTGTGGTCACCATCAGCTGGGCCAAGAGCATCCCAG|  |  |  |  |  |  |  |  |  |  |  |  |  |  |  |  |  |  |  |  |  |  |  |  |  |  |  |  |  |  |  |  |  |  |  |  |  |  |  |  |  |  |  |  |  |  |  |  |  |  |  |  |  |  |  |  |  |  |  |  |  |  |  |  |  |  |  |  |  |  |  |  | | --- | --- | --- | --- | --- | --- | --- | --- | --- | --- | --- | --- | --- | --- | --- | --- | --- | --- | --- | --- | --- | --- | --- | --- | --- | --- | --- | --- | --- | --- | --- | --- | --- | --- | --- | --- | --- | --- | --- | --- | --- | --- | --- | --- | --- | --- | --- | --- | --- | --- | --- | --- | --- | --- | --- | --- | --- | --- | --- | --- | --- | --- | --- | --- | --- | --- | --- | --- | --- | --- | --- | --- | | Gorilla gorGor1 Supercontig\_0041211 3291 3461 + **CG** | CAGCCCCAGTGAATGCACTGGTGTCTCATCTGCTGGTGGTTGAGCCTGAGAAGCTCTATGCCATGCCTGACCCCGCAGGCCCTGATGGGCACCTCCCAGCCGTGGCTACCCTCTGTGACCTCTTTGACCGAGAGATCGTGGTCACCATCAGCTGGGCCAAGAGCATCCCAG|  |  |  |  |  |  |  |  |  |  |  |  |  |  |  |  |  |  |  |  |  |  |  |  |  |  |  |  |  |  |  |  |  |  |  |  |  |  |  |  |  |  |  |  |  |  |  |  |  |  |  |  |  |  |  |  |  |  |  |  |  |  |  |  | | --- | --- | --- | --- | --- | --- | --- | --- | --- | --- | --- | --- | --- | --- | --- | --- | --- | --- | --- | --- | --- | --- | --- | --- | --- | --- | --- | --- | --- | --- | --- | --- | --- | --- | --- | --- | --- | --- | --- | --- | --- | --- | --- | --- | --- | --- | --- | --- | --- | --- | --- | --- | --- | --- | --- | --- | --- | --- | --- | --- | --- | --- | --- | --- | | Orangutan ponAbe2 chr11 11654406 11654576 - **CG** | CAGCCCCAGTAAATGCACTGGTGTCTCATCTGCTGGTGGTTGAGCCTGAGAAGCTCTATGCCATGCCTGACCCTGCAGGCCCTGATGGGCACCTCCCAGCCGTGGCTACCCTCTGTGACCTCTTTGACCGAGAGATCGTGGTCACCATCAGCTGGGCCAAGAGCATCCCAG|  |  |  |  |  |  |  |  |  |  |  |  |  |  |  |  |  |  |  |  |  |  |  |  |  |  |  |  |  |  |  |  |  |  |  |  |  |  |  |  |  |  |  |  |  |  |  |  |  |  |  |  |  |  |  |  | | --- | --- | --- | --- | --- | --- | --- | --- | --- | --- | --- | --- | --- | --- | --- | --- | --- | --- | --- | --- | --- | --- | --- | --- | --- | --- | --- | --- | --- | --- | --- | --- | --- | --- | --- | --- | --- | --- | --- | --- | --- | --- | --- | --- | --- | --- | --- | --- | --- | --- | --- | --- | --- | --- | --- | --- | | Rhesus rheMac2 chr14 10113125 10113295 - **CG** | CAGCCCCAGTGAATGCACTGGTGTCTCATCTGCTGGTGGTTGAGCCTGAGAAGCTCTATGCCATGCCCGACCCCGCGGGCCCTGATGGGCACCTCCCAGCCGTGGCTACCCTCTGTGACCTCTTTGACCGAGAGATTGTGGTCACCATCAGCTGGGCCAAGAGCATCCCAG|  |  |  |  |  |  |  |  |  |  |  |  |  |  |  |  |  |  |  |  |  |  |  |  |  |  |  |  |  |  |  |  |  |  |  |  |  |  |  |  |  |  |  |  |  |  |  |  | | --- | --- | --- | --- | --- | --- | --- | --- | --- | --- | --- | --- | --- | --- | --- | --- | --- | --- | --- | --- | --- | --- | --- | --- | --- | --- | --- | --- | --- | --- | --- | --- | --- | --- | --- | --- | --- | --- | --- | --- | --- | --- | --- | --- | --- | --- | --- | --- | | Baboon papHam1 scaffold6473 73801 73971 + **CG** | CAGCCCCAGTGAATGCACTGGTGTCTCATCTGCTGGTGGTTGAGCCTGAGAAGCTCTATGCCATGCCCGACCCCGCGGGCCCTGATGGGCACCTCCCAGCCGTGGCTACCCTCTGTGACCTCTTTGACCGAGAGATTGTGGTCACCATCAGCTGGGCCAAGAGCATCCCAG|  |  |  |  |  |  |  |  |  |  |  |  |  |  |  |  |  |  |  |  |  |  |  |  |  |  |  |  |  |  |  |  |  |  |  |  |  |  |  |  | | --- | --- | --- | --- | --- | --- | --- | --- | --- | --- | --- | --- | --- | --- | --- | --- | --- | --- | --- | --- | --- | --- | --- | --- | --- | --- | --- | --- | --- | --- | --- | --- | --- | --- | --- | --- | --- | --- | --- | --- | | Marmoset calJac1 Contig3724 85494 85664 + **CA** | CAGCCCCAGTGAATGCACTGGTGTCTCATCTGCTGGTCGTGGAGCCTGAGAAACTCTATGCCATGCCTGACCCTGCGGGCCCTGATGGGCACCTCCCAGCCGTGGCTACCCTCTGTGACCTCTTTGACCGAGAGATTGTGGTCACCATCAGCTGGGCCAAGAGCATCCCAG|  |  |  |  |  |  |  |  |  |  |  |  |  |  |  |  |  |  |  |  |  |  |  |  |  |  |  |  |  |  |  |  | | --- | --- | --- | --- | --- | --- | --- | --- | --- | --- | --- | --- | --- | --- | --- | --- | --- | --- | --- | --- | --- | --- | --- | --- | --- | --- | --- | --- | --- | --- | --- | --- | | Lemur micMur1 scaffold\_498 432633 432803 + **TG** | CAGCCCCGGTGAATGCACTGGTGTCTCACCTGCTAGTGGTTGAACCTGAAAAGCTATATGCCATGCCCGACCCAGCGGGCCCTGATGGACACCTCCCAGCTGTGGCTACCCTCTGTGACCTCTTTGACCGAGAGATCGTGGTCACCATCAGCTGGGCCAAGAGCATCCCAG|  |  |  |  |  |  |  |  |  |  |  |  |  |  |  |  |  |  |  |  |  |  |  |  | | --- | --- | --- | --- | --- | --- | --- | --- | --- | --- | --- | --- | --- | --- | --- | --- | --- | --- | --- | --- | --- | --- | --- | --- | | Mouse mm9 chr19 6987206 6987376 - **TG** | TAGCCCCAGTGAACGCTCTGGTGTCGCATCTGCTGGTGGTTGAACCTGAGAAGCTGTACGCCATGCCTGACCCAGCAAGCCCCGATGGACACCTCCCCGCTGTGGCCACTCTCTGTGACCTTTTTGATCGAGAGATAGTGGTCACCATCAGCTGGGCCAAGAGCATCCCAG|  |  |  |  |  |  |  |  |  |  |  |  |  |  |  |  | | --- | --- | --- | --- | --- | --- | --- | --- | --- | --- | --- | --- | --- | --- | --- | --- | | Cow bosTau4 chr29 44394105 44394275 + **TA** | CAGCCCCGGTGAACGCACTCGTGTCCCACCTGCTGGTGGTTGAACCTGAGAAGCTGTATGCCATGCCCGACCCAGCGGGCCCTGATGGACACCTCCCAGCTGTGGCCACCCTCTGTGACCTCTTTGACCGAGAGATCGTGGTCACCATCAGCTGGGCCAAGAGCATCCCAG|  |  |  |  |  |  |  |  | | --- | --- | --- | --- | --- | --- | --- | --- | | Dog canFam2 chr18 55783389 55783559 - **TG** | CAGCTCCTGTAAATGCACTGGTGTCTCACCTGCTGGTGGTAGAGCCCGAGAAGCTCTATGCCATGCCTGACCCAGCGGGCCCTGACGGACACCTCCCAGCTGTGGCTACCCTCTGTGACCTCTTTGACCGAGAGATTGTGGTCACCATCAGCTGGGCCAAGAGTATCCCAG | | | | | | | | | | | | | | | | | | | | | | | | | | | | | | | | | | | | | | | | | | | | | | | | | | | | | | | | | | | | | | | | | | | | | | | | | | | | | | | | | | | | |

**Alignment** (splice site sequences are in lowercase)  

```
Human      agCAGCCCCAGTGAATGCACTGGTGTCTCATCTGCTGGTGGTTGAGCCTGAGAAGCTCTATGCCATGCCTGACCCCGCAG
Chimp      c...............................................................................
Gorilla    c...............................................................................
Orangutan  c...........A..............................................................T....
Rhesus     c....................................................................C........G.
Baboon     c....................................................................C........G.
Marmoset   ca.....................................C..G...........A....................T..G.
Lemur      t........G....................C.....A........A.....A.....A...........C.....A..G.
Mouse      t.T............C..T........G.................A...........G..C..............A...A
Cow        ta.......G.....C.....C.....C..C..............A...........G...........C.....A..G.
Dog        t.....T..T..A.................C...........A.....C..........................A..G.

Human      GCCCTGATGGGCACCTCCCAGCCGTGGCTACCCTCTGTGACCTCTTTGACCGAGAGATTGTGGTCACCATCAGCTGGGCC
Chimp      ................................................................................
Gorilla    ..........................................................C.....................
Orangutan  ..........................................................C.....................
Rhesus     ................................................................................
Baboon     ................................................................................
Marmoset   ................................................................................
Lemur      ..........A...........T...................................C.....................
Mouse      ....C.....A........C..T.....C..T...........T.....T........A.....................
Cow        ..........A...........T.....C.............................C.....................
Dog        .......C..A...........T.........................................................

Human      AAGAGCATCCCAGgt
Chimp      ...............
Gorilla    ...............
Orangutan  ...............
Rhesus     ...............
Baboon     ...............
Marmoset   ...............
Lemur      ...............
Mouse      ...............
Cow        ...............
Dog        .....T.........
```

---

## 25. uc001pqi.1\_14\_27

**Summary**  

|  |  |  |  |  |  |  |  |  |  |  |  |  |  |  |  |  |  |  |  |  |  |  |  |  |  |
| --- | --- | --- | --- | --- | --- | --- | --- | --- | --- | --- | --- | --- | --- | --- | --- | --- | --- | --- | --- | --- | --- | --- | --- | --- | --- |
| No Exon ID Position (hg19) Dir Human acceptor Chimp acceptor Category Usage Gene symbol Protein accession mRNA accession Gene title Note|  |  |  |  |  |  |  |  |  |  |  |  |  | | --- | --- | --- | --- | --- | --- | --- | --- | --- | --- | --- | --- | --- | | 25 uc001pqi.1\_14\_27 chr11:117059445 + AG GG (A3) shift; decrease; inframe alternative SIDT2 Q24JR2 BC114522.1 SID1 transmembrane family, member 2  | | | | | | | | | | | | | | | | | | | | | | | | | |

**Orthologs**  

|  |  |  |  |  |  |  |  |  |  |  |  |  |  |  |  |  |  |  |  |  |  |  |  |  |  |  |  |  |  |  |  |  |  |  |  |  |  |  |  |  |  |  |  |  |  |  |  |  |  |  |  |  |  |  |  |  |  |  |  |  |  |  |  |  |  |  |  |  |  |  |  |  |  |  |  |  |  |  |  |  |  |  |  |  |  |  |  |  |  |  |  |  |  |  |  |  |  |  |  |  |  |  |  |
| --- | --- | --- | --- | --- | --- | --- | --- | --- | --- | --- | --- | --- | --- | --- | --- | --- | --- | --- | --- | --- | --- | --- | --- | --- | --- | --- | --- | --- | --- | --- | --- | --- | --- | --- | --- | --- | --- | --- | --- | --- | --- | --- | --- | --- | --- | --- | --- | --- | --- | --- | --- | --- | --- | --- | --- | --- | --- | --- | --- | --- | --- | --- | --- | --- | --- | --- | --- | --- | --- | --- | --- | --- | --- | --- | --- | --- | --- | --- | --- | --- | --- | --- | --- | --- | --- | --- | --- | --- | --- | --- | --- | --- | --- | --- | --- | --- | --- | --- | --- | --- | --- | --- | --- |
| Species Assembly Chromosome Exon start Exon end Dir Acceptor Exon sequence|  |  |  |  |  |  |  |  |  |  |  |  |  |  |  |  |  |  |  |  |  |  |  |  |  |  |  |  |  |  |  |  |  |  |  |  |  |  |  |  |  |  |  |  |  |  |  |  |  |  |  |  |  |  |  |  |  |  |  |  |  |  |  |  |  |  |  |  |  |  |  |  |  |  |  |  |  |  |  |  |  |  |  |  |  |  |  |  |  |  |  |  |  |  |  |  | | --- | --- | --- | --- | --- | --- | --- | --- | --- | --- | --- | --- | --- | --- | --- | --- | --- | --- | --- | --- | --- | --- | --- | --- | --- | --- | --- | --- | --- | --- | --- | --- | --- | --- | --- | --- | --- | --- | --- | --- | --- | --- | --- | --- | --- | --- | --- | --- | --- | --- | --- | --- | --- | --- | --- | --- | --- | --- | --- | --- | --- | --- | --- | --- | --- | --- | --- | --- | --- | --- | --- | --- | --- | --- | --- | --- | --- | --- | --- | --- | --- | --- | --- | --- | --- | --- | --- | --- | --- | --- | --- | --- | --- | --- | --- | --- | | Human hg19 chr11 117059445 117059542 + **AG** | GTACTCGGCCCCGAGTGGACTCCATGAGCTCTGTGGAGGAGGATGACTACGACACATTGACCGACATCGATTCCGACAAGAATGTCATTCGCACCAAG|  |  |  |  |  |  |  |  |  |  |  |  |  |  |  |  |  |  |  |  |  |  |  |  |  |  |  |  |  |  |  |  |  |  |  |  |  |  |  |  |  |  |  |  |  |  |  |  |  |  |  |  |  |  |  |  |  |  |  |  |  |  |  |  |  |  |  |  |  |  |  |  |  |  |  |  |  |  |  |  |  |  |  |  |  |  |  |  | | --- | --- | --- | --- | --- | --- | --- | --- | --- | --- | --- | --- | --- | --- | --- | --- | --- | --- | --- | --- | --- | --- | --- | --- | --- | --- | --- | --- | --- | --- | --- | --- | --- | --- | --- | --- | --- | --- | --- | --- | --- | --- | --- | --- | --- | --- | --- | --- | --- | --- | --- | --- | --- | --- | --- | --- | --- | --- | --- | --- | --- | --- | --- | --- | --- | --- | --- | --- | --- | --- | --- | --- | --- | --- | --- | --- | --- | --- | --- | --- | --- | --- | --- | --- | --- | --- | --- | --- | | Chimp panTro2 chr11 116016929 116017026 + **GG** | GTACTCGGCCCCGAGTGGACTCCATGAGCTCTGTGGAGGAGGATGACTATGACACATTGACCGACATCGATTCCGACAAGAATGTCATTCGCACCAAG|  |  |  |  |  |  |  |  |  |  |  |  |  |  |  |  |  |  |  |  |  |  |  |  |  |  |  |  |  |  |  |  |  |  |  |  |  |  |  |  |  |  |  |  |  |  |  |  |  |  |  |  |  |  |  |  |  |  |  |  |  |  |  |  |  |  |  |  |  |  |  |  |  |  |  |  |  |  |  |  | | --- | --- | --- | --- | --- | --- | --- | --- | --- | --- | --- | --- | --- | --- | --- | --- | --- | --- | --- | --- | --- | --- | --- | --- | --- | --- | --- | --- | --- | --- | --- | --- | --- | --- | --- | --- | --- | --- | --- | --- | --- | --- | --- | --- | --- | --- | --- | --- | --- | --- | --- | --- | --- | --- | --- | --- | --- | --- | --- | --- | --- | --- | --- | --- | --- | --- | --- | --- | --- | --- | --- | --- | --- | --- | --- | --- | --- | --- | --- | --- | | Gorilla gorGor1 Supercontig\_0017920 1911 2008 - **GG** | GTACTCGGCCCCGAGTGGACTCCATGAGCTCTGTGGAGGAGGATGACTACGACACATTGACCGACATAGATTCCGACAAGAATGTCATTCGCACCAAG|  |  |  |  |  |  |  |  |  |  |  |  |  |  |  |  |  |  |  |  |  |  |  |  |  |  |  |  |  |  |  |  |  |  |  |  |  |  |  |  |  |  |  |  |  |  |  |  |  |  |  |  |  |  |  |  |  |  |  |  |  |  |  |  |  |  |  |  |  |  |  |  | | --- | --- | --- | --- | --- | --- | --- | --- | --- | --- | --- | --- | --- | --- | --- | --- | --- | --- | --- | --- | --- | --- | --- | --- | --- | --- | --- | --- | --- | --- | --- | --- | --- | --- | --- | --- | --- | --- | --- | --- | --- | --- | --- | --- | --- | --- | --- | --- | --- | --- | --- | --- | --- | --- | --- | --- | --- | --- | --- | --- | --- | --- | --- | --- | --- | --- | --- | --- | --- | --- | --- | --- | | Orangutan ponAbe2 chr11 114097197 114097294 + **GG** | GTACTCGGCCCCGAGTGGACTCTATGAGCTCTGTGGAGGAGGATGACTACGACACATTGACCGACATCGATTCCGACAAGAATGTCATTCGTACCAAG|  |  |  |  |  |  |  |  |  |  |  |  |  |  |  |  |  |  |  |  |  |  |  |  |  |  |  |  |  |  |  |  |  |  |  |  |  |  |  |  |  |  |  |  |  |  |  |  |  |  |  |  |  |  |  |  |  |  |  |  |  |  |  |  | | --- | --- | --- | --- | --- | --- | --- | --- | --- | --- | --- | --- | --- | --- | --- | --- | --- | --- | --- | --- | --- | --- | --- | --- | --- | --- | --- | --- | --- | --- | --- | --- | --- | --- | --- | --- | --- | --- | --- | --- | --- | --- | --- | --- | --- | --- | --- | --- | --- | --- | --- | --- | --- | --- | --- | --- | --- | --- | --- | --- | --- | --- | --- | --- | | Rhesus rheMac2 chr14 115587443 115587540 + **TG** | GTACTCGGCCCCGAGTGGACTCCATGAGCTCTGTGGAGGAGGATGACTATGACACATTGACCGACATCGATTCCGACAAGAATGTCATTCGCACCAAG|  |  |  |  |  |  |  |  |  |  |  |  |  |  |  |  |  |  |  |  |  |  |  |  |  |  |  |  |  |  |  |  |  |  |  |  |  |  |  |  |  |  |  |  |  |  |  |  |  |  |  |  |  |  |  |  | | --- | --- | --- | --- | --- | --- | --- | --- | --- | --- | --- | --- | --- | --- | --- | --- | --- | --- | --- | --- | --- | --- | --- | --- | --- | --- | --- | --- | --- | --- | --- | --- | --- | --- | --- | --- | --- | --- | --- | --- | --- | --- | --- | --- | --- | --- | --- | --- | --- | --- | --- | --- | --- | --- | --- | --- | | Baboon papHam1 scaffold7583 70866 70963 - **GG** | GTACTCGGCCCCGAGTGGACTCCATGAGCTCTGTGGAGGAGGATGACTATGACACATTGACTGACATCGATTCCGATAAGAATGTCATTCGCACCAAG|  |  |  |  |  |  |  |  |  |  |  |  |  |  |  |  |  |  |  |  |  |  |  |  |  |  |  |  |  |  |  |  |  |  |  |  |  |  |  |  |  |  |  |  |  |  |  |  | | --- | --- | --- | --- | --- | --- | --- | --- | --- | --- | --- | --- | --- | --- | --- | --- | --- | --- | --- | --- | --- | --- | --- | --- | --- | --- | --- | --- | --- | --- | --- | --- | --- | --- | --- | --- | --- | --- | --- | --- | --- | --- | --- | --- | --- | --- | --- | --- | | Marmoset calJac1 Contig480 3024 3121 + **GG** | GTACTCGGCCACGGGTGGACTCCATGAGCTCTGTGGAGGAGGACGACTACGACACACTGACCGACATCGACTCAGACAAGAACGTCATTCGCACTAAG|  |  |  |  |  |  |  |  |  |  |  |  |  |  |  |  |  |  |  |  |  |  |  |  |  |  |  |  |  |  |  |  |  |  |  |  |  |  |  |  | | --- | --- | --- | --- | --- | --- | --- | --- | --- | --- | --- | --- | --- | --- | --- | --- | --- | --- | --- | --- | --- | --- | --- | --- | --- | --- | --- | --- | --- | --- | --- | --- | --- | --- | --- | --- | --- | --- | --- | --- | | Lemur micMur1 scaffold\_722 18184 18281 - **GG** | GCACTCGGCCGCGGCTGGACTCCATGAGCTCCGTGGAGGAGGATGACTACGACACATTGACTGACATCGATTCAGACAAGAATGTTATCCGCACCAAG|  |  |  |  |  |  |  |  |  |  |  |  |  |  |  |  |  |  |  |  |  |  |  |  |  |  |  |  |  |  |  |  | | --- | --- | --- | --- | --- | --- | --- | --- | --- | --- | --- | --- | --- | --- | --- | --- | --- | --- | --- | --- | --- | --- | --- | --- | --- | --- | --- | --- | --- | --- | --- | --- | | Galago otoGar1 scaffold\_111272.1-145955 98564 98661 - **GG** | GTACCCGGCCACGACTGGACTCCATGAGCTCTGTGGAGGAGGATGACTATGATACATTGACTGACATCGATTCAGACAAGAATGTTATTCGCACCAAG|  |  |  |  |  |  |  |  |  |  |  |  |  |  |  |  |  |  |  |  |  |  |  |  | | --- | --- | --- | --- | --- | --- | --- | --- | --- | --- | --- | --- | --- | --- | --- | --- | --- | --- | --- | --- | --- | --- | --- | --- | | Mouse mm9 chr9 45753749 45753846 - **GG** | GTCCTCGGCCTCGACTGGACTCCATGAGCTCCGTGGAAGAGGATGACTACGACACACTGACTGACATCGACTCAGACAAAAACGTCATTCGAACCAAG|  |  |  |  |  |  |  |  |  |  |  |  |  |  |  |  | | --- | --- | --- | --- | --- | --- | --- | --- | --- | --- | --- | --- | --- | --- | --- | --- | | Cow bosTau4 chr15 26311608 26311705 + **GG** | GCACTCGACCGCGACTGGACTCCATGAGCTCTGTGGAGGAAGATGACTATGACACTCTGGCCGATGTCGATTCAGACAAGAACGTCATTCGCACCAAG|  |  |  |  |  |  |  |  | | --- | --- | --- | --- | --- | --- | --- | --- | | Dog canFam2 chr5 19410029 19410126 - **GG** | GCACTCGGCCACGCCTGGACTCCATGAGCTCTGTGGAGGAGGACGACTATGACACGTTGACTGACATTGATTCGGACAAGAATGTCATTCGCACCAAG | | | | | | | | | | | | | | | | | | | | | | | | | | | | | | | | | | | | | | | | | | | | | | | | | | | | | | | | | | | | | | | | | | | | | | | | | | | | | | | | | | | | | | | | | | | |

**Alignment** (splice site sequences are in lowercase)  

```
Human      agGTACTCGGCCCCGAGTGGACTCCATGAGCTCTGTGGAGGAGGATGACTACGACACATTGACCGACATCGATTCCGACA
Chimp      g..................................................T............................
Gorilla    g....................................................................A..........
Orangutan  g.......................T.......................................................
Rhesus     t..................................................T............................
Baboon     g..................................................T...........T..............T.
Marmoset   g...........A..G.............................C............C.............C..A....
Lemur      g..C........G..GC................C.............................T...........A....
Galago     g.....C.....A...C..................................T..T........T...........A....
Mouse      g...C.......T...C................C.....A..................C....T........C..A....
Cow        g..C.....A..G...C.........................A........T.....TC..G....TG.......A....
Dog        g..C........A..CC............................C.....T.....G.....T.....T.....G....

Human      AGAATGTCATTCGCACCAAGgt
Chimp      ......................
Gorilla    ......................
Orangutan  .............T........
Rhesus     ......................
Baboon     ......................
Marmoset   ....C...........T.....
Lemur      .......T..C...........
Galago     .......T..............
Mouse      .A..C........A........
Cow        ....C.................
Dog        ......................
```

---

## 26. uc009zhm.1\_7\_7

**Summary**  

|  |  |  |  |  |  |  |  |  |  |  |  |  |  |  |  |  |  |  |  |  |  |  |  |  |  |
| --- | --- | --- | --- | --- | --- | --- | --- | --- | --- | --- | --- | --- | --- | --- | --- | --- | --- | --- | --- | --- | --- | --- | --- | --- | --- |
| No Exon ID Position (hg19) Dir Human acceptor Chimp acceptor Category Usage Gene symbol Protein accession mRNA accession Gene title Note|  |  |  |  |  |  |  |  |  |  |  |  |  | | --- | --- | --- | --- | --- | --- | --- | --- | --- | --- | --- | --- | --- | | 26 uc009zhm.1\_7\_7 chr12:10594899 - AG AC (A6) exonization; frameshift alternative KLRC1 A8MYW4 AK310992.1 killer cell lectin-like receptor subfamily C, member 1  | | | | | | | | | | | | | | | | | | | | | | | | | |

**Orthologs**  

|  |  |  |  |  |  |  |  |  |  |  |  |  |  |  |  |  |  |  |  |  |  |  |  |  |  |  |  |  |  |  |  |  |  |  |  |  |  |  |  |
| --- | --- | --- | --- | --- | --- | --- | --- | --- | --- | --- | --- | --- | --- | --- | --- | --- | --- | --- | --- | --- | --- | --- | --- | --- | --- | --- | --- | --- | --- | --- | --- | --- | --- | --- | --- | --- | --- | --- | --- |
| Species Assembly Chromosome Exon start Exon end Dir Acceptor Exon sequence|  |  |  |  |  |  |  |  |  |  |  |  |  |  |  |  |  |  |  |  |  |  |  |  |  |  |  |  |  |  |  |  | | --- | --- | --- | --- | --- | --- | --- | --- | --- | --- | --- | --- | --- | --- | --- | --- | --- | --- | --- | --- | --- | --- | --- | --- | --- | --- | --- | --- | --- | --- | --- | --- | | Human hg19 chr12 10594898 10594899 - **AG** | AA|  |  |  |  |  |  |  |  |  |  |  |  |  |  |  |  |  |  |  |  |  |  |  |  | | --- | --- | --- | --- | --- | --- | --- | --- | --- | --- | --- | --- | --- | --- | --- | --- | --- | --- | --- | --- | --- | --- | --- | --- | | Chimp panTro2 chr12 10751714 10751715 - **AC** | AA|  |  |  |  |  |  |  |  |  |  |  |  |  |  |  |  | | --- | --- | --- | --- | --- | --- | --- | --- | --- | --- | --- | --- | --- | --- | --- | --- | | Gorilla gorGor1 Supercontig\_0083358 5879 5880 - **AC** | AA|  |  |  |  |  |  |  |  | | --- | --- | --- | --- | --- | --- | --- | --- | | Orangutan ponAbe2 chr12 10732571 10732572 - **AC** | AA | | | | | | | | | | | | | | | | | | | | | | | | | | | | | | | | | | | |

**Alignment** (splice site sequences are in lowercase)  

```
Human      agAA
Chimp      .c..
Gorilla    .c..
Orangutan  .c..
```

---

## 27. uc001rqw.2\_16\_17

**Summary**  

|  |  |  |  |  |  |  |  |  |  |  |  |  |  |  |  |  |  |  |  |  |  |  |  |  |  |
| --- | --- | --- | --- | --- | --- | --- | --- | --- | --- | --- | --- | --- | --- | --- | --- | --- | --- | --- | --- | --- | --- | --- | --- | --- | --- |
| No Exon ID Position (hg19) Dir Human acceptor Chimp acceptor Category Usage Gene symbol Protein accession mRNA accession Gene title Note|  |  |  |  |  |  |  |  |  |  |  |  |  | | --- | --- | --- | --- | --- | --- | --- | --- | --- | --- | --- | --- | --- | | 27 uc001rqw.2\_16\_17 chr12:48440231 - AG AA (A3) shift; decrease; inframe alternative SENP1 NP\_055369.1 NM\_014554.2 sentrin-specific protease 1 NAGNAG | | | | | | | | | | | | | | | | | | | | | | | | | |

**Orthologs**  

|  |  |  |  |  |  |  |  |  |  |  |  |  |  |  |  |  |  |  |  |  |  |  |  |  |  |  |  |  |  |  |  |  |  |  |  |  |  |  |  |  |  |  |  |  |  |  |  |  |  |  |  |  |  |  |  |  |  |  |  |  |  |  |  |  |  |  |  |  |  |  |  |  |  |  |  |  |  |  |  |  |  |  |  |  |  |  |  |  |  |  |  |  |  |  |  |  |  |  |  |  |  |  |  |
| --- | --- | --- | --- | --- | --- | --- | --- | --- | --- | --- | --- | --- | --- | --- | --- | --- | --- | --- | --- | --- | --- | --- | --- | --- | --- | --- | --- | --- | --- | --- | --- | --- | --- | --- | --- | --- | --- | --- | --- | --- | --- | --- | --- | --- | --- | --- | --- | --- | --- | --- | --- | --- | --- | --- | --- | --- | --- | --- | --- | --- | --- | --- | --- | --- | --- | --- | --- | --- | --- | --- | --- | --- | --- | --- | --- | --- | --- | --- | --- | --- | --- | --- | --- | --- | --- | --- | --- | --- | --- | --- | --- | --- | --- | --- | --- | --- | --- | --- | --- | --- | --- | --- | --- |
| Species Assembly Chromosome Exon start Exon end Dir Acceptor Exon sequence|  |  |  |  |  |  |  |  |  |  |  |  |  |  |  |  |  |  |  |  |  |  |  |  |  |  |  |  |  |  |  |  |  |  |  |  |  |  |  |  |  |  |  |  |  |  |  |  |  |  |  |  |  |  |  |  |  |  |  |  |  |  |  |  |  |  |  |  |  |  |  |  |  |  |  |  |  |  |  |  |  |  |  |  |  |  |  |  |  |  |  |  |  |  |  |  | | --- | --- | --- | --- | --- | --- | --- | --- | --- | --- | --- | --- | --- | --- | --- | --- | --- | --- | --- | --- | --- | --- | --- | --- | --- | --- | --- | --- | --- | --- | --- | --- | --- | --- | --- | --- | --- | --- | --- | --- | --- | --- | --- | --- | --- | --- | --- | --- | --- | --- | --- | --- | --- | --- | --- | --- | --- | --- | --- | --- | --- | --- | --- | --- | --- | --- | --- | --- | --- | --- | --- | --- | --- | --- | --- | --- | --- | --- | --- | --- | --- | --- | --- | --- | --- | --- | --- | --- | --- | --- | --- | --- | --- | --- | --- | --- | | Human hg19 chr12 48440139 48440231 - **AG** | ATTCCTCAGCAGATGAATGGAAGTGACTGTGGGATGTTTGCCTGCAAATATGCTGACTGTATTACCAAAGACAGACCAATCAACTTCACACAG|  |  |  |  |  |  |  |  |  |  |  |  |  |  |  |  |  |  |  |  |  |  |  |  |  |  |  |  |  |  |  |  |  |  |  |  |  |  |  |  |  |  |  |  |  |  |  |  |  |  |  |  |  |  |  |  |  |  |  |  |  |  |  |  |  |  |  |  |  |  |  |  |  |  |  |  |  |  |  |  |  |  |  |  |  |  |  |  | | --- | --- | --- | --- | --- | --- | --- | --- | --- | --- | --- | --- | --- | --- | --- | --- | --- | --- | --- | --- | --- | --- | --- | --- | --- | --- | --- | --- | --- | --- | --- | --- | --- | --- | --- | --- | --- | --- | --- | --- | --- | --- | --- | --- | --- | --- | --- | --- | --- | --- | --- | --- | --- | --- | --- | --- | --- | --- | --- | --- | --- | --- | --- | --- | --- | --- | --- | --- | --- | --- | --- | --- | --- | --- | --- | --- | --- | --- | --- | --- | --- | --- | --- | --- | --- | --- | --- | --- | | Chimp panTro2 chr12 41687969 41688061 + **AA** | ATTCCTCAGCAGATGAATGGAAGTGACTGTGGGATGTTTGCCTGCAAATATGCTGACTGTATTACCAAAGACAGACCAATCAACTTCACACAG|  |  |  |  |  |  |  |  |  |  |  |  |  |  |  |  |  |  |  |  |  |  |  |  |  |  |  |  |  |  |  |  |  |  |  |  |  |  |  |  |  |  |  |  |  |  |  |  |  |  |  |  |  |  |  |  |  |  |  |  |  |  |  |  |  |  |  |  |  |  |  |  |  |  |  |  |  |  |  |  | | --- | --- | --- | --- | --- | --- | --- | --- | --- | --- | --- | --- | --- | --- | --- | --- | --- | --- | --- | --- | --- | --- | --- | --- | --- | --- | --- | --- | --- | --- | --- | --- | --- | --- | --- | --- | --- | --- | --- | --- | --- | --- | --- | --- | --- | --- | --- | --- | --- | --- | --- | --- | --- | --- | --- | --- | --- | --- | --- | --- | --- | --- | --- | --- | --- | --- | --- | --- | --- | --- | --- | --- | --- | --- | --- | --- | --- | --- | --- | --- | | Gorilla gorGor1 Supercontig\_0000115 174086 174178 - **AA** | ATTCCTCAGCAGATGAATGGAAGTGACTGTGGGATGTTTGCCTGCAAATATGCTGACTGTATTACCAAAGACAGACCAATCAACTTCACACAG|  |  |  |  |  |  |  |  |  |  |  |  |  |  |  |  |  |  |  |  |  |  |  |  |  |  |  |  |  |  |  |  |  |  |  |  |  |  |  |  |  |  |  |  |  |  |  |  |  |  |  |  |  |  |  |  |  |  |  |  |  |  |  |  |  |  |  |  |  |  |  |  | | --- | --- | --- | --- | --- | --- | --- | --- | --- | --- | --- | --- | --- | --- | --- | --- | --- | --- | --- | --- | --- | --- | --- | --- | --- | --- | --- | --- | --- | --- | --- | --- | --- | --- | --- | --- | --- | --- | --- | --- | --- | --- | --- | --- | --- | --- | --- | --- | --- | --- | --- | --- | --- | --- | --- | --- | --- | --- | --- | --- | --- | --- | --- | --- | --- | --- | --- | --- | --- | --- | --- | --- | | Orangutan ponAbe2 chr12 47694595 47694687 - **AA** | ATTCCTCAGCAGATGAATGGAAGTGACTGTGGGATGTTTGCCTGCAAATATGCTGACTGTATTACCAAAGACAGACCAATCAACTTCACACAG|  |  |  |  |  |  |  |  |  |  |  |  |  |  |  |  |  |  |  |  |  |  |  |  |  |  |  |  |  |  |  |  |  |  |  |  |  |  |  |  |  |  |  |  |  |  |  |  |  |  |  |  |  |  |  |  |  |  |  |  |  |  |  |  | | --- | --- | --- | --- | --- | --- | --- | --- | --- | --- | --- | --- | --- | --- | --- | --- | --- | --- | --- | --- | --- | --- | --- | --- | --- | --- | --- | --- | --- | --- | --- | --- | --- | --- | --- | --- | --- | --- | --- | --- | --- | --- | --- | --- | --- | --- | --- | --- | --- | --- | --- | --- | --- | --- | --- | --- | --- | --- | --- | --- | --- | --- | --- | --- | | Rhesus rheMac2 chr11 45125345 45125437 - **AA** | ATTCCACAGCAGATGAATGGAAGTGACTGTGGGATGTTTGCCTGCAAATATGCTGACTATATTACCAAAGACAGACCAATCAACTTTACACAG|  |  |  |  |  |  |  |  |  |  |  |  |  |  |  |  |  |  |  |  |  |  |  |  |  |  |  |  |  |  |  |  |  |  |  |  |  |  |  |  |  |  |  |  |  |  |  |  |  |  |  |  |  |  |  |  | | --- | --- | --- | --- | --- | --- | --- | --- | --- | --- | --- | --- | --- | --- | --- | --- | --- | --- | --- | --- | --- | --- | --- | --- | --- | --- | --- | --- | --- | --- | --- | --- | --- | --- | --- | --- | --- | --- | --- | --- | --- | --- | --- | --- | --- | --- | --- | --- | --- | --- | --- | --- | --- | --- | --- | --- | | Baboon papHam1 scaffold7340 34708 34800 - **AA** | ATTCCACAGCAGATGAATGGAAGTGACTGTGGGATGTTTGCCTGCAAATATGCTGACTGTATTACCAAAGACAGACCAATCAACTTTACACAG|  |  |  |  |  |  |  |  |  |  |  |  |  |  |  |  |  |  |  |  |  |  |  |  |  |  |  |  |  |  |  |  |  |  |  |  |  |  |  |  |  |  |  |  |  |  |  |  | | --- | --- | --- | --- | --- | --- | --- | --- | --- | --- | --- | --- | --- | --- | --- | --- | --- | --- | --- | --- | --- | --- | --- | --- | --- | --- | --- | --- | --- | --- | --- | --- | --- | --- | --- | --- | --- | --- | --- | --- | --- | --- | --- | --- | --- | --- | --- | --- | | Marmoset calJac1 Contig2801 259394 259486 - **AA** | ATTCCACAGCAGATGAATGGAAGTGACTGTGGAATGTTTGCCTGCAAATACGCTGACTGTATTACCAAAGACAGGCCGATCAGCTTCACACAG|  |  |  |  |  |  |  |  |  |  |  |  |  |  |  |  |  |  |  |  |  |  |  |  |  |  |  |  |  |  |  |  |  |  |  |  |  |  |  |  | | --- | --- | --- | --- | --- | --- | --- | --- | --- | --- | --- | --- | --- | --- | --- | --- | --- | --- | --- | --- | --- | --- | --- | --- | --- | --- | --- | --- | --- | --- | --- | --- | --- | --- | --- | --- | --- | --- | --- | --- | | Tarsier tarSyr1 scaffold\_28896 135 227 + **AA** | ATTCCACAGCAGATGAATGGAAGTGACTGTGGGATGTTTGCCTGCAAATATGCTGACTGTATTACCAAAGACAGACCAATCAACTTCACACAG|  |  |  |  |  |  |  |  |  |  |  |  |  |  |  |  |  |  |  |  |  |  |  |  |  |  |  |  |  |  |  |  | | --- | --- | --- | --- | --- | --- | --- | --- | --- | --- | --- | --- | --- | --- | --- | --- | --- | --- | --- | --- | --- | --- | --- | --- | --- | --- | --- | --- | --- | --- | --- | --- | | Lemur micMur1 scaffold\_10802 25656 25748 - **AA** | ATTCCACAACAGATGAATGGCAGTGACTGTGGGATGTTTGCCTGCAAATATGCTGACTGTATAACCAAAGACAGACCAATCAACTTCACACAG|  |  |  |  |  |  |  |  |  |  |  |  |  |  |  |  |  |  |  |  |  |  |  |  | | --- | --- | --- | --- | --- | --- | --- | --- | --- | --- | --- | --- | --- | --- | --- | --- | --- | --- | --- | --- | --- | --- | --- | --- | | Mouse mm9 chr15 97875734 97875826 - **AA** | ATCCCACAGCAGATGAATGGGAGTGACTGTGGAATGTTTGCTTGCAAATATGCTGACTGCATTACCAAAGACAGACCAATCAACTTCACACAG|  |  |  |  |  |  |  |  |  |  |  |  |  |  |  |  | | --- | --- | --- | --- | --- | --- | --- | --- | --- | --- | --- | --- | --- | --- | --- | --- | | Cow bosTau4 chr5 35424000 35424092 + **AA** | ATTCCACAGCAGATGAATGGAAGCGACTGTGGCATGTTTGCCTGCAAATATGCTGACTGTATTACCAAAGACAGACCAATCAACTTCACACAG|  |  |  |  |  |  |  |  | | --- | --- | --- | --- | --- | --- | --- | --- | | Dog canFam2 chr27 9724711 9724803 + **AA** | ATTCCACAGCAGATGAATGGAAGTGACTGTGGCATGTTTGCTTGCAAATATGCTGACTGCATTACCAAAGACAGACCGATCAACTTCACACAG | | | | | | | | | | | | | | | | | | | | | | | | | | | | | | | | | | | | | | | | | | | | | | | | | | | | | | | | | | | | | | | | | | | | | | | | | | | | | | | | | | | | | | | | | | | |

**Alignment** (splice site sequences are in lowercase)  

```
Human      agATTCCTCAGCAGATGAATGGAAGTGACTGTGGGATGTTTGCCTGCAAATATGCTGACTGTATTACCAAAGACAGACCA
Chimp      .a..............................................................................
Gorilla    .a..............................................................................
Orangutan  .a..............................................................................
Rhesus     .a.....A....................................................A...................
Baboon     .a.....A........................................................................
Marmoset   .a.....A..........................A.................C.......................G..G
Tarsier    .a.....A........................................................................
Lemur      .a.....A..A...........C.........................................A...............
Mouse      .a..C..A..............G...........A........T.................C..................
Cow        .a.....A.................C........C.............................................
Dog        .a.....A..........................C........T.................C.................G

Human      ATCAACTTCACACAGgt
Chimp      .................
Gorilla    .................
Orangutan  .................
Rhesus     ........T........
Baboon     ........T........
Marmoset   ....G............
Tarsier    .................
Lemur      .................
Mouse      .................
Cow        .................
Dog        .................
```

---

## 28. uc010sxw.1\_9\_15

**Summary**  

|  |  |  |  |  |  |  |  |  |  |  |  |  |  |  |  |  |  |  |  |  |  |  |  |  |  |
| --- | --- | --- | --- | --- | --- | --- | --- | --- | --- | --- | --- | --- | --- | --- | --- | --- | --- | --- | --- | --- | --- | --- | --- | --- | --- |
| No Exon ID Position (hg19) Dir Human acceptor Chimp acceptor Category Usage Gene symbol Protein accession mRNA accession Gene title Note|  |  |  |  |  |  |  |  |  |  |  |  |  | | --- | --- | --- | --- | --- | --- | --- | --- | --- | --- | --- | --- | --- | | 28 uc010sxw.1\_9\_15 chr12:110465513 + AG GG (A1) shift; increase; inframe alternative ANKRD13A NP\_149112.1 AF155103.1 ankyrin repeat domain-containing protein 13A NAGNAG | | | | | | | | | | | | | | | | | | | | | | | | | |

**Orthologs**  

|  |  |  |  |  |  |  |  |  |  |  |  |  |  |  |  |  |  |  |  |  |  |  |  |  |  |  |  |  |  |  |  |  |  |  |  |  |  |  |  |  |  |  |  |  |  |  |  |  |  |  |  |  |  |  |  |  |  |  |  |  |  |  |  |  |  |  |  |  |  |  |  |  |  |  |  |  |  |  |  |  |  |  |  |  |  |  |  |  |  |  |  |  |  |  |  |  |  |  |  |  |  |  |  |  |  |  |  |  |  |  |  |
| --- | --- | --- | --- | --- | --- | --- | --- | --- | --- | --- | --- | --- | --- | --- | --- | --- | --- | --- | --- | --- | --- | --- | --- | --- | --- | --- | --- | --- | --- | --- | --- | --- | --- | --- | --- | --- | --- | --- | --- | --- | --- | --- | --- | --- | --- | --- | --- | --- | --- | --- | --- | --- | --- | --- | --- | --- | --- | --- | --- | --- | --- | --- | --- | --- | --- | --- | --- | --- | --- | --- | --- | --- | --- | --- | --- | --- | --- | --- | --- | --- | --- | --- | --- | --- | --- | --- | --- | --- | --- | --- | --- | --- | --- | --- | --- | --- | --- | --- | --- | --- | --- | --- | --- | --- | --- | --- | --- | --- | --- | --- | --- |
| Species Assembly Chromosome Exon start Exon end Dir Acceptor Exon sequence|  |  |  |  |  |  |  |  |  |  |  |  |  |  |  |  |  |  |  |  |  |  |  |  |  |  |  |  |  |  |  |  |  |  |  |  |  |  |  |  |  |  |  |  |  |  |  |  |  |  |  |  |  |  |  |  |  |  |  |  |  |  |  |  |  |  |  |  |  |  |  |  |  |  |  |  |  |  |  |  |  |  |  |  |  |  |  |  |  |  |  |  |  |  |  |  |  |  |  |  |  |  |  |  | | --- | --- | --- | --- | --- | --- | --- | --- | --- | --- | --- | --- | --- | --- | --- | --- | --- | --- | --- | --- | --- | --- | --- | --- | --- | --- | --- | --- | --- | --- | --- | --- | --- | --- | --- | --- | --- | --- | --- | --- | --- | --- | --- | --- | --- | --- | --- | --- | --- | --- | --- | --- | --- | --- | --- | --- | --- | --- | --- | --- | --- | --- | --- | --- | --- | --- | --- | --- | --- | --- | --- | --- | --- | --- | --- | --- | --- | --- | --- | --- | --- | --- | --- | --- | --- | --- | --- | --- | --- | --- | --- | --- | --- | --- | --- | --- | --- | --- | --- | --- | --- | --- | --- | --- | | Human hg19 chr12 110465513 110465571 + **AG** | ACAGGAACCCGCTGGAATCTTTGCTGGGAACTGTGGAACACCAATTTGGTGCACAAGGG|  |  |  |  |  |  |  |  |  |  |  |  |  |  |  |  |  |  |  |  |  |  |  |  |  |  |  |  |  |  |  |  |  |  |  |  |  |  |  |  |  |  |  |  |  |  |  |  |  |  |  |  |  |  |  |  |  |  |  |  |  |  |  |  |  |  |  |  |  |  |  |  |  |  |  |  |  |  |  |  |  |  |  |  |  |  |  |  |  |  |  |  |  |  |  |  | | --- | --- | --- | --- | --- | --- | --- | --- | --- | --- | --- | --- | --- | --- | --- | --- | --- | --- | --- | --- | --- | --- | --- | --- | --- | --- | --- | --- | --- | --- | --- | --- | --- | --- | --- | --- | --- | --- | --- | --- | --- | --- | --- | --- | --- | --- | --- | --- | --- | --- | --- | --- | --- | --- | --- | --- | --- | --- | --- | --- | --- | --- | --- | --- | --- | --- | --- | --- | --- | --- | --- | --- | --- | --- | --- | --- | --- | --- | --- | --- | --- | --- | --- | --- | --- | --- | --- | --- | --- | --- | --- | --- | --- | --- | --- | --- | | Chimp panTro2 chr12 111302479 111302537 + **GG** | ACAGGAACCCGCTGGAATCTTTGCTGGGAACTGTGGAACACCAATTTGGTGCACAAGGG|  |  |  |  |  |  |  |  |  |  |  |  |  |  |  |  |  |  |  |  |  |  |  |  |  |  |  |  |  |  |  |  |  |  |  |  |  |  |  |  |  |  |  |  |  |  |  |  |  |  |  |  |  |  |  |  |  |  |  |  |  |  |  |  |  |  |  |  |  |  |  |  |  |  |  |  |  |  |  |  |  |  |  |  |  |  |  |  | | --- | --- | --- | --- | --- | --- | --- | --- | --- | --- | --- | --- | --- | --- | --- | --- | --- | --- | --- | --- | --- | --- | --- | --- | --- | --- | --- | --- | --- | --- | --- | --- | --- | --- | --- | --- | --- | --- | --- | --- | --- | --- | --- | --- | --- | --- | --- | --- | --- | --- | --- | --- | --- | --- | --- | --- | --- | --- | --- | --- | --- | --- | --- | --- | --- | --- | --- | --- | --- | --- | --- | --- | --- | --- | --- | --- | --- | --- | --- | --- | --- | --- | --- | --- | --- | --- | --- | --- | | Gorilla gorGor1 Supercontig\_0005322 16346 16404 - **GG** | ACAGGAACCCGCTGGAATCTTTGCTGGGAACTGTGGAACACCAATTTGGTGCACAAGGG|  |  |  |  |  |  |  |  |  |  |  |  |  |  |  |  |  |  |  |  |  |  |  |  |  |  |  |  |  |  |  |  |  |  |  |  |  |  |  |  |  |  |  |  |  |  |  |  |  |  |  |  |  |  |  |  |  |  |  |  |  |  |  |  |  |  |  |  |  |  |  |  |  |  |  |  |  |  |  |  | | --- | --- | --- | --- | --- | --- | --- | --- | --- | --- | --- | --- | --- | --- | --- | --- | --- | --- | --- | --- | --- | --- | --- | --- | --- | --- | --- | --- | --- | --- | --- | --- | --- | --- | --- | --- | --- | --- | --- | --- | --- | --- | --- | --- | --- | --- | --- | --- | --- | --- | --- | --- | --- | --- | --- | --- | --- | --- | --- | --- | --- | --- | --- | --- | --- | --- | --- | --- | --- | --- | --- | --- | --- | --- | --- | --- | --- | --- | --- | --- | | Orangutan ponAbe2 chr12 111812297 111812355 + **GG** | ACAGGAACCCGCTGGAATCTTTGCTGGGAACTGTGGAACACCAGTTTGGCGCACAAGGG|  |  |  |  |  |  |  |  |  |  |  |  |  |  |  |  |  |  |  |  |  |  |  |  |  |  |  |  |  |  |  |  |  |  |  |  |  |  |  |  |  |  |  |  |  |  |  |  |  |  |  |  |  |  |  |  |  |  |  |  |  |  |  |  |  |  |  |  |  |  |  |  | | --- | --- | --- | --- | --- | --- | --- | --- | --- | --- | --- | --- | --- | --- | --- | --- | --- | --- | --- | --- | --- | --- | --- | --- | --- | --- | --- | --- | --- | --- | --- | --- | --- | --- | --- | --- | --- | --- | --- | --- | --- | --- | --- | --- | --- | --- | --- | --- | --- | --- | --- | --- | --- | --- | --- | --- | --- | --- | --- | --- | --- | --- | --- | --- | --- | --- | --- | --- | --- | --- | --- | --- | | Rhesus rheMac2 chr11 110945600 110945658 + **GG** | ACAGGAACCCGCTGGAATCTTTGCTGGGAACTGTGGAACACCAGTTTGGTGCACAAGGG|  |  |  |  |  |  |  |  |  |  |  |  |  |  |  |  |  |  |  |  |  |  |  |  |  |  |  |  |  |  |  |  |  |  |  |  |  |  |  |  |  |  |  |  |  |  |  |  |  |  |  |  |  |  |  |  |  |  |  |  |  |  |  |  | | --- | --- | --- | --- | --- | --- | --- | --- | --- | --- | --- | --- | --- | --- | --- | --- | --- | --- | --- | --- | --- | --- | --- | --- | --- | --- | --- | --- | --- | --- | --- | --- | --- | --- | --- | --- | --- | --- | --- | --- | --- | --- | --- | --- | --- | --- | --- | --- | --- | --- | --- | --- | --- | --- | --- | --- | --- | --- | --- | --- | --- | --- | --- | --- | | Baboon papHam1 scaffold7358 55023 55081 + **GG** | ACAGGAACCCGCTGGAATCTTTGCTGGGAACTGTGGAACACCAGTTTGGTGCACAAGGG|  |  |  |  |  |  |  |  |  |  |  |  |  |  |  |  |  |  |  |  |  |  |  |  |  |  |  |  |  |  |  |  |  |  |  |  |  |  |  |  |  |  |  |  |  |  |  |  |  |  |  |  |  |  |  |  | | --- | --- | --- | --- | --- | --- | --- | --- | --- | --- | --- | --- | --- | --- | --- | --- | --- | --- | --- | --- | --- | --- | --- | --- | --- | --- | --- | --- | --- | --- | --- | --- | --- | --- | --- | --- | --- | --- | --- | --- | --- | --- | --- | --- | --- | --- | --- | --- | --- | --- | --- | --- | --- | --- | --- | --- | | Marmoset calJac1 Contig4417 108965 109023 + **TG** | ACAGGAACCCGCTGGAATCTTTGCTGGGAACTGTGGAACACCAGTTTGGTGCTCAAGGG|  |  |  |  |  |  |  |  |  |  |  |  |  |  |  |  |  |  |  |  |  |  |  |  |  |  |  |  |  |  |  |  |  |  |  |  |  |  |  |  |  |  |  |  |  |  |  |  | | --- | --- | --- | --- | --- | --- | --- | --- | --- | --- | --- | --- | --- | --- | --- | --- | --- | --- | --- | --- | --- | --- | --- | --- | --- | --- | --- | --- | --- | --- | --- | --- | --- | --- | --- | --- | --- | --- | --- | --- | --- | --- | --- | --- | --- | --- | --- | --- | | Tarsier tarSyr1 scaffold\_999 20486 20544 - **TG** | ACAGGAACCCACTGGAATCTTTGCTGGGAACTGTGGAACACCAGTTTGGTGCTCAAGGG|  |  |  |  |  |  |  |  |  |  |  |  |  |  |  |  |  |  |  |  |  |  |  |  |  |  |  |  |  |  |  |  |  |  |  |  |  |  |  |  | | --- | --- | --- | --- | --- | --- | --- | --- | --- | --- | --- | --- | --- | --- | --- | --- | --- | --- | --- | --- | --- | --- | --- | --- | --- | --- | --- | --- | --- | --- | --- | --- | --- | --- | --- | --- | --- | --- | --- | --- | | Lemur micMur1 scaffold\_5155 64688 64746 + **GG** | ACAGGAACCCACTGGAGTCTTTGCTGGGGACTGTGGAACACCAGTTTGGTGCTCAAGGG|  |  |  |  |  |  |  |  |  |  |  |  |  |  |  |  |  |  |  |  |  |  |  |  |  |  |  |  |  |  |  |  | | --- | --- | --- | --- | --- | --- | --- | --- | --- | --- | --- | --- | --- | --- | --- | --- | --- | --- | --- | --- | --- | --- | --- | --- | --- | --- | --- | --- | --- | --- | --- | --- | | Galago otoGar1 scaffold\_102006.1-45357 7544 7602 - **GG** | ACAGGAACCCACTGGAATCTTTGCTGGGAACCGTGGAACACCAGTTTGGTGCTCAAGGG|  |  |  |  |  |  |  |  |  |  |  |  |  |  |  |  |  |  |  |  |  |  |  |  | | --- | --- | --- | --- | --- | --- | --- | --- | --- | --- | --- | --- | --- | --- | --- | --- | --- | --- | --- | --- | --- | --- | --- | --- | | Mouse mm9 chr5 115247865 115247923 + **GG** | ACCGGAATCCACTGGAGTCTCTGCTCGGAACCGTGGAACATCAGTTTGGTGCTCAAGGG|  |  |  |  |  |  |  |  |  |  |  |  |  |  |  |  | | --- | --- | --- | --- | --- | --- | --- | --- | --- | --- | --- | --- | --- | --- | --- | --- | | Cow bosTau4 chr17 66566878 66566936 - **CG** | ACAGGAACCCACTGGAATCGCTGCTCGGGACTGTGGAACACCAGTTTGGTGCTCAAGGG|  |  |  |  |  |  |  |  | | --- | --- | --- | --- | --- | --- | --- | --- | | Dog canFam2 chr26 20169742 20169800 - **GG** | ACAGGAACCCACTGGAATCTCTGCTGGGAACTGTGGAACACCAGTTTGGAGCCCAAGGG | | | | | | | | | | | | | | | | | | | | | | | | | | | | | | | | | | | | | | | | | | | | | | | | | | | | | | | | | | | | | | | | | | | | | | | | | | | | | | | | | | | | | | | | | | | | | | | | | | |

**Alignment** (splice site sequences are in lowercase)  

```
Human      agACAGGAACCCGCTGGAATCTTTGCTGGGAACTGTGGAACACCAATTTGGTGCACAAGGGgt
Chimp      g..............................................................
Gorilla    g..............................................................
Orangutan  g............................................G.....C...........
Rhesus     g............................................G.................
Baboon     g............................................G.................
Marmoset   t............................................G........T........
Tarsier    t...........A................................G........T........
Lemur      g...........A.....G...........G..............G........T........
Galago     g...........A....................C...........G........T........
Mouse      g...C....T..A.....G...C....C.....C........T..G........T........
Cow        c...........A........GC....C..G..............G........T........
Dog        g...........A.........C......................G.....A..C........
```

---

## 29. uc010agc.2\_7\_7

**Summary**  

|  |  |  |  |  |  |  |  |  |  |  |  |  |  |  |  |  |  |  |  |  |  |  |  |  |  |
| --- | --- | --- | --- | --- | --- | --- | --- | --- | --- | --- | --- | --- | --- | --- | --- | --- | --- | --- | --- | --- | --- | --- | --- | --- | --- |
| No Exon ID Position (hg19) Dir Human acceptor Chimp acceptor Category Usage Gene symbol Protein accession mRNA accession Gene title Note|  |  |  |  |  |  |  |  |  |  |  |  |  | | --- | --- | --- | --- | --- | --- | --- | --- | --- | --- | --- | --- | --- | | 29 uc010agc.2\_7\_7 chr13:103491901 + AG AC (A1) shift; increase; inframe alternative BIVM NP\_001153068.1 NM\_001159596.1 basic immunoglobulin-like variable motif-containing protein  | | | | | | | | | | | | | | | | | | | | | | | | | |

**Orthologs**  

|  |  |  |  |  |  |  |  |  |  |  |  |  |  |  |  |  |  |  |  |  |  |  |  |  |  |  |  |  |  |  |  |  |  |  |  |  |  |  |  |  |  |  |  |  |  |  |  |  |  |  |  |  |  |  |  |  |  |  |  |  |  |  |  |  |  |  |  |  |  |  |  |  |  |  |  |  |  |  |  |  |  |  |  |  |  |  |  |  |  |  |  |  |  |  |  |
| --- | --- | --- | --- | --- | --- | --- | --- | --- | --- | --- | --- | --- | --- | --- | --- | --- | --- | --- | --- | --- | --- | --- | --- | --- | --- | --- | --- | --- | --- | --- | --- | --- | --- | --- | --- | --- | --- | --- | --- | --- | --- | --- | --- | --- | --- | --- | --- | --- | --- | --- | --- | --- | --- | --- | --- | --- | --- | --- | --- | --- | --- | --- | --- | --- | --- | --- | --- | --- | --- | --- | --- | --- | --- | --- | --- | --- | --- | --- | --- | --- | --- | --- | --- | --- | --- | --- | --- | --- | --- | --- | --- | --- | --- | --- | --- |
| Species Assembly Chromosome Exon start Exon end Dir Acceptor Exon sequence|  |  |  |  |  |  |  |  |  |  |  |  |  |  |  |  |  |  |  |  |  |  |  |  |  |  |  |  |  |  |  |  |  |  |  |  |  |  |  |  |  |  |  |  |  |  |  |  |  |  |  |  |  |  |  |  |  |  |  |  |  |  |  |  |  |  |  |  |  |  |  |  |  |  |  |  |  |  |  |  |  |  |  |  |  |  |  |  | | --- | --- | --- | --- | --- | --- | --- | --- | --- | --- | --- | --- | --- | --- | --- | --- | --- | --- | --- | --- | --- | --- | --- | --- | --- | --- | --- | --- | --- | --- | --- | --- | --- | --- | --- | --- | --- | --- | --- | --- | --- | --- | --- | --- | --- | --- | --- | --- | --- | --- | --- | --- | --- | --- | --- | --- | --- | --- | --- | --- | --- | --- | --- | --- | --- | --- | --- | --- | --- | --- | --- | --- | --- | --- | --- | --- | --- | --- | --- | --- | --- | --- | --- | --- | --- | --- | --- | --- | | Human hg19 chr13 103491901 103492215 + **AG** | CCAACATTTTTTTCTTCTCAGGTTGGGGGAAATTTGCATTGCATCATAGCATTCCAGAGACTTAACTGGCAAAGATTTGGCCTTTGGAACTTTCCATTTGGAACCATTAGACAAGAATCACAACCTCCAACACATGCCCAGGGAATTGCCAAATCTGAGAGTGAAGACAATATTTCCAAGAAGCAGCATGGGCGTCTGGGCCGGTCTTTCAGTGCTAGTTTCCATCAGGACTCGGCATGGAAAAAGATGTCTAGTATCCATGAGAGAAGGAACAGTGGTTACCAGGGTTACAGTGATTACGATGGGAATGATTGA|  |  |  |  |  |  |  |  |  |  |  |  |  |  |  |  |  |  |  |  |  |  |  |  |  |  |  |  |  |  |  |  |  |  |  |  |  |  |  |  |  |  |  |  |  |  |  |  |  |  |  |  |  |  |  |  |  |  |  |  |  |  |  |  |  |  |  |  |  |  |  |  |  |  |  |  |  |  |  |  | | --- | --- | --- | --- | --- | --- | --- | --- | --- | --- | --- | --- | --- | --- | --- | --- | --- | --- | --- | --- | --- | --- | --- | --- | --- | --- | --- | --- | --- | --- | --- | --- | --- | --- | --- | --- | --- | --- | --- | --- | --- | --- | --- | --- | --- | --- | --- | --- | --- | --- | --- | --- | --- | --- | --- | --- | --- | --- | --- | --- | --- | --- | --- | --- | --- | --- | --- | --- | --- | --- | --- | --- | --- | --- | --- | --- | --- | --- | --- | --- | | Chimp panTro2 chr13 103750799 103751113 + **AC** | CCAACTTTTTTTTCTTCTCAGGTTGGGGGAAATTTGCATTGCATCATAGCATTCCAGAGACTTAGTTGGCAAAGATTTGGCCTTTGGAACTTTCCATTTGGAACCATTAGACAAGAATCACAACCTCCAACACATGCCCAGGGAATTGCCAAATCTGAGAGTGAAGACAATATTTCCAAGAAGCAGCATGGGCGTCTGGGCCGGTCTTTCAGTGCTAGTTTCCATCAGGACTCAGCATGGAAAAAGATGTCTAGTATCCATGAGAGAAGGAACAGTGGTTACCAGGGTTACAGTGATTACGATGGGAATGATTGA|  |  |  |  |  |  |  |  |  |  |  |  |  |  |  |  |  |  |  |  |  |  |  |  |  |  |  |  |  |  |  |  |  |  |  |  |  |  |  |  |  |  |  |  |  |  |  |  |  |  |  |  |  |  |  |  |  |  |  |  |  |  |  |  |  |  |  |  |  |  |  |  | | --- | --- | --- | --- | --- | --- | --- | --- | --- | --- | --- | --- | --- | --- | --- | --- | --- | --- | --- | --- | --- | --- | --- | --- | --- | --- | --- | --- | --- | --- | --- | --- | --- | --- | --- | --- | --- | --- | --- | --- | --- | --- | --- | --- | --- | --- | --- | --- | --- | --- | --- | --- | --- | --- | --- | --- | --- | --- | --- | --- | --- | --- | --- | --- | --- | --- | --- | --- | --- | --- | --- | --- | | Gorilla gorGor1 Supercontig\_0051668 14663 14977 - **AC** | CCAACTTTTTTTTCTTCTCAGGTTGGGGGAAATTTGCATTGCATCATAGCATTCCAGAGACTTAATTGGCAAAGATTTGGCCTTTGGAACTTTCCATTTGGAACCATTAGACAAGAATCACAACCTCCAACACATGCCCAGGGAATTGCCAAATCTGAGAGTGAAGACAATATTTCCAAGAAGCAGCATGGGCGTCTGGGCCGGTCTTTCAGTGCTAGTTTCCATCAGGACTCGGCATGGAAAAAGATGTCTAGTATCCATGAGAGAAGGAACAGTGGTTACCAGGGTTACAGTGATTACGATGGGAATGATTGA|  |  |  |  |  |  |  |  |  |  |  |  |  |  |  |  |  |  |  |  |  |  |  |  |  |  |  |  |  |  |  |  |  |  |  |  |  |  |  |  |  |  |  |  |  |  |  |  |  |  |  |  |  |  |  |  |  |  |  |  |  |  |  |  | | --- | --- | --- | --- | --- | --- | --- | --- | --- | --- | --- | --- | --- | --- | --- | --- | --- | --- | --- | --- | --- | --- | --- | --- | --- | --- | --- | --- | --- | --- | --- | --- | --- | --- | --- | --- | --- | --- | --- | --- | --- | --- | --- | --- | --- | --- | --- | --- | --- | --- | --- | --- | --- | --- | --- | --- | --- | --- | --- | --- | --- | --- | --- | --- | | Orangutan ponAbe2 chr13 105213135 105213449 + **AC** | CCAACTTTTTTTTCTTCTCAGGTTGGGGGAAATTTGCATTGCATCATAGCATTCCAGAGACTTAATTGGCAAAGATTTGGCCTTTGGAACTTTCCATTTGGAACCATTAGACAAGAATCACAACCTCCAACACATGCCCAGGGAATTGCCAAATCTGAGAGTGAAGACAATATTTCCAAGAAGCAGCATGGGCGTCTGGGCCGGTCTTTCAGTGCTAGTTTCCATCAGGACTCGGCATGGAAAAAGATGTCTAGTATCCATGAGAGAAGGAACAGTGGTTACCAGGGTTACAGTGATTACGATGGGAATGATTGA|  |  |  |  |  |  |  |  |  |  |  |  |  |  |  |  |  |  |  |  |  |  |  |  |  |  |  |  |  |  |  |  |  |  |  |  |  |  |  |  |  |  |  |  |  |  |  |  |  |  |  |  |  |  |  |  | | --- | --- | --- | --- | --- | --- | --- | --- | --- | --- | --- | --- | --- | --- | --- | --- | --- | --- | --- | --- | --- | --- | --- | --- | --- | --- | --- | --- | --- | --- | --- | --- | --- | --- | --- | --- | --- | --- | --- | --- | --- | --- | --- | --- | --- | --- | --- | --- | --- | --- | --- | --- | --- | --- | --- | --- | | Rhesus rheMac2 chr17 83138054 83138368 + **AC** | CCAACTTTTTTTTCTTCTCAGGTTGGGGGAAATTTGCATTGCATCATAGCATTCCAGAGACTTAACTGGCAAAGATTTGGCCTTTGGAACTTTCCATTTGGAACTATTAGACAAGAATCACAACCTCCCACACATGCCCAGGGAATTTCCAAATCTGAGAGTGAAGACAATATTTCCAAGAAGCAGCATGGGCGTCTGGGCCGGTCTTTCAGTGCTAGTTTCCATCAGGAGTCGGCATGGAAAAAGATGTCTAGTATCCATGAGAGAAGGAACAGTGGTTACCAGGGTTACAGTGATTACGATGGGAATGATTGA|  |  |  |  |  |  |  |  |  |  |  |  |  |  |  |  |  |  |  |  |  |  |  |  |  |  |  |  |  |  |  |  |  |  |  |  |  |  |  |  |  |  |  |  |  |  |  |  | | --- | --- | --- | --- | --- | --- | --- | --- | --- | --- | --- | --- | --- | --- | --- | --- | --- | --- | --- | --- | --- | --- | --- | --- | --- | --- | --- | --- | --- | --- | --- | --- | --- | --- | --- | --- | --- | --- | --- | --- | --- | --- | --- | --- | --- | --- | --- | --- | | Baboon papHam1 scaffold77 112833 113147 - **AC** | CCAACTTTTTTTTCTTCTCAGGTTGGGGGAAATTTGCATTGCATCATAGCATTCCAGAGACTTAACTGGCAAAGATTTGGCCTTTGGAACTTTCCATTTGGAACTATTAGACAAGAATCACAACCTCCAACACATGCCCAGGGAATTTCCAAATCTGAGAGTGAAGACAATATTTCCAAGAAGCAGCATGGGCGTCTGGGCCGGTCTTTCAGTGCTAGTTTCCATCAGGAGTCGGCATGGAAAAAGATGTCTAGTATCCATGAGAGAAGGAACAGTGGTTACCAGGGTTACAGTGATTACGATGGGAATGATTGA|  |  |  |  |  |  |  |  |  |  |  |  |  |  |  |  |  |  |  |  |  |  |  |  |  |  |  |  |  |  |  |  |  |  |  |  |  |  |  |  | | --- | --- | --- | --- | --- | --- | --- | --- | --- | --- | --- | --- | --- | --- | --- | --- | --- | --- | --- | --- | --- | --- | --- | --- | --- | --- | --- | --- | --- | --- | --- | --- | --- | --- | --- | --- | --- | --- | --- | --- | | Marmoset calJac1 Contig1683 253671 253985 - **AC** | CCAACTTTTTTTTCTTCTCAGGTTGGAGGAAATTTGCATTGCATCATAGCATTCCAGAGACTTAATTGGCAAAGATTTGGCATTTGGAACTTTCCATTTGGAACCATTAGACAAGAATCACAACCTCCAACACATGCCCAGGGAATTGCCAAATCTGAGAGTGAAGACAATATTTCCAAGAAGCAGCATGGGCGTCTGGGTCGGTCTTTCAGTGCTAGTTTCCAGCAGGACTCAGCCTGGAAAAAGATGTCTAGTATCCATGAGAGAAGGAACAGTGGTTACCAGGGCTACAGTGATTACGATGGGAATGATTGA|  |  |  |  |  |  |  |  |  |  |  |  |  |  |  |  |  |  |  |  |  |  |  |  |  |  |  |  |  |  |  |  | | --- | --- | --- | --- | --- | --- | --- | --- | --- | --- | --- | --- | --- | --- | --- | --- | --- | --- | --- | --- | --- | --- | --- | --- | --- | --- | --- | --- | --- | --- | --- | --- | | Tarsier tarSyr1 scaffold\_6922 11002 11319 + **AC** | CCAACCTTTTTTTTTTCTTTGCAGGTTGGGGGGAATTTGCATTGCATCATAGCATTTCAGAGACTCAGTTGGCAAAGATTTGGCCTTTGGAACTTTCCATTTGGAACCATCAGACAGGAATCACAACCTCCAACACATTCCCAGGGAATCACCAAATCTGAGAGTGAAGACAATATTTCCAAGAAACAGCGTGGGCGTCTGGGCCGGTCTTTCAGTACTAGTTTCCATCAGGACTCGGCATGGAAAAAGATGACTAGTATCCATGAAAGAAGGAACAGTGGCTACCAGGGTTACAGTGATTATGATGGGAATGACTGA|  |  |  |  |  |  |  |  |  |  |  |  |  |  |  |  |  |  |  |  |  |  |  |  | | --- | --- | --- | --- | --- | --- | --- | --- | --- | --- | --- | --- | --- | --- | --- | --- | --- | --- | --- | --- | --- | --- | --- | --- | | Lemur micMur1 scaffold\_238 189534 189848 + **AC** | CCAACTTTTTTTCTTGTTTAGGTTGGGGGAAATTTGCATTGCATCATAGCATTCCAGAGACTTAGTTGGCAAAGATTTGGCCTTTGGAATTTTCCATTTGGAAACATTAGACAAGAATCACAACCTCCAACACATGCCCAGGGAATTGCCAAATCGGAGAGTGAAGACAATATTTCCAAGAAGCAGCATGGGCGTCTGGGCCGGTCTTTCAGTGCTAGTTTCCATCAGGACTCGGCATGGAAAAAGATGTCTAGTATCCATGAGCGAAGGAACAGTGGCTACCAGGGTTACAGTGATTATGATGGGAATGATTGA|  |  |  |  |  |  |  |  |  |  |  |  |  |  |  |  | | --- | --- | --- | --- | --- | --- | --- | --- | --- | --- | --- | --- | --- | --- | --- | --- | | Cow bosTau4 chr12 76864678 76864995 + **AC** | CCAACATTTTTTTTTTCTTCTTAGGTTGGAGGAAACTTGCATTGCATCATAGCATTCCAGAGACTTAGCTGGCAAAGATTTGGCCTTTGGAACTTTCCGTTTGGAACTATTAGACAGGAATCACAGCCTCCAATGCATGCCCATGGAATTGCCAAATCTGAGAGTGAGGACAATATCTCCAAGAAGCAGCACGGGCGCCTGGGCCGGTCTTTTAGTACTGGTTTCCATCAGGACTCAACATGGAAAAAGATGTCCAGTATCCACGAGAGAAGGAACAGTGGCTACCATGGTTACAGTGATTACGAGGGGAATGACTGA|  |  |  |  |  |  |  |  | | --- | --- | --- | --- | --- | --- | --- | --- | | Dog canFam2 chr22 55280775 55281088 + **AC** | CCAGCTTCTTTTCTTCTCAGGTTGGGGGAAATTTGCATTGCATCATCGCATTCCAGAGACTTAATTGGCAAAGATTTGGCCTTTGGAACTTTCCATTTGGAACCATTAGACGAGAATCACAACCTCCAACACATGCCCAGGGAATTGCTAAATCTGAGAGTGAAGATAATATATCCAAGAAGCAGCATGGGCGTCTGGGCCGGTCTTTTAGTGCTAGTTTCCATCAGGACTCAGCATGGAAAAAGATGTCCAGTATCCATGAGAGAAGGAACAGTGGCTACCAGGGTTACAGTGATTATGATGGGAATGACTGA | | | | | | | | | | | | | | | | | | | | | | | | | | | | | | | | | | | | | | | | | | | | | | | | | | | | | | | | | | | | | | | | | | | | | | | | | | | | | | | | | | | | |

**Alignment** (splice site sequences are in lowercase)  

```
Human      agCCAAC---ATTTTTTTCTTCTCAGGTTGGGGGAAATTTGCATTGCATCATAGCATTCCAGAGACTTAACTGGCAAAGA
Chimp      .c.....---T..........................................................GT.........
Gorilla    .c.....---T...........................................................T.........
Orangutan  .c.....---T...........................................................T.........
Rhesus     .c.....---T.....................................................................
Baboon     .c.....---T.....................................................................
Marmoset   .c.....---T....................A......................................T.........
Tarsier    .c.....CTTT..........TG...........G.......................T........C.GT.........
Lemur      .c.....---T......CT.GT.T.............................................GT.........
Cow        .c.....ATTT............T.......A.....C...............................G..........
Dog        .c...G.----..C......................................C.................T.........

Human      TTTGGCCTTTGGAACTTTCCATTTGGAACCATTAGACAAGAATCACAACCTCCAACACATGCCCAGGGAATTGCCAAATC
Chimp      ................................................................................
Gorilla    ................................................................................
Orangutan  ................................................................................
Rhesus     .............................T.......................C..................T.......
Baboon     .............................T..........................................T.......
Marmoset   ......A.........................................................................
Tarsier    ................................C.....G.....................T..........CA.......
Lemur      ..............T.............A...................................................
Cow        ....................G........T........G........G.......TG........T..............
Dog        .....................................G....................................T.....

Human      TGAGAGTGAAGACAATATTTCCAAGAAGCAGCATGGGCGTCTGGGCCGGTCTTTCAGTGCTAGTTTCCATCAGGACTCGG
Chimp      ..............................................................................A.
Gorilla    ................................................................................
Orangutan  ................................................................................
Rhesus     ...........................................................................G....
Baboon     ...........................................................................G....
Marmoset   .............................................T.......................G........A.
Tarsier    ...........................A....G.........................A.....................
Lemur      G...............................................................................
Cow        .........G........C..............C.....C..............T...A..G................AA
Dog        ............T.....A...................................T.......................A.

Human      CATGGAAAAAGATGTCTAGTATCCATGAGAGAAGGAACAGTGGTTACCAGGGTTACAGTGATTACGATGGGAATGATTGA
Chimp      ................................................................................
Gorilla    ................................................................................
Orangutan  ................................................................................
Rhesus     ................................................................................
Baboon     ................................................................................
Marmoset   .C..................................................C...........................
Tarsier    ..............A.............A..............C....................T...........C...
Lemur      .............................C.............C....................T...............
Cow        ................C........C.................C.....T.................G........C...
Dog        ................C..........................C....................T...........C...
```

---

## 30. uc010tqu.1\_5\_19

**Summary**  

|  |  |  |  |  |  |  |  |  |  |  |  |  |  |  |  |  |  |  |  |  |  |  |  |  |  |
| --- | --- | --- | --- | --- | --- | --- | --- | --- | --- | --- | --- | --- | --- | --- | --- | --- | --- | --- | --- | --- | --- | --- | --- | --- | --- |
| No Exon ID Position (hg19) Dir Human acceptor Chimp acceptor Category Usage Gene symbol Protein accession mRNA accession Gene title Note|  |  |  |  |  |  |  |  |  |  |  |  |  | | --- | --- | --- | --- | --- | --- | --- | --- | --- | --- | --- | --- | --- | | 30 uc010tqu.1\_5\_19 chr14:52985996 - AG AA (A3) shift; decrease; inframe alternative TXNDC16 NP\_001153519.1 NM\_001160047.1 thioredoxin domain-containing protein 16 dbSNP:rs28759013 | | | | | | | | | | | | | | | | | | | | | | | | | |

**Orthologs**  

|  |  |  |  |  |  |  |  |  |  |  |  |  |  |  |  |  |  |  |  |  |  |  |  |  |  |  |  |  |  |  |  |  |  |  |  |  |  |  |  |  |  |  |  |  |  |  |  |  |  |  |  |  |  |  |  |  |  |  |  |  |  |  |  |  |  |  |  |  |  |  |  |  |  |  |  |  |  |  |  |  |  |  |  |  |  |  |  |  |  |  |  |  |  |  |  |  |  |  |  |  |  |  |  |  |  |  |  |  |  |  |  |
| --- | --- | --- | --- | --- | --- | --- | --- | --- | --- | --- | --- | --- | --- | --- | --- | --- | --- | --- | --- | --- | --- | --- | --- | --- | --- | --- | --- | --- | --- | --- | --- | --- | --- | --- | --- | --- | --- | --- | --- | --- | --- | --- | --- | --- | --- | --- | --- | --- | --- | --- | --- | --- | --- | --- | --- | --- | --- | --- | --- | --- | --- | --- | --- | --- | --- | --- | --- | --- | --- | --- | --- | --- | --- | --- | --- | --- | --- | --- | --- | --- | --- | --- | --- | --- | --- | --- | --- | --- | --- | --- | --- | --- | --- | --- | --- | --- | --- | --- | --- | --- | --- | --- | --- | --- | --- | --- | --- | --- | --- | --- | --- |
| Species Assembly Chromosome Exon start Exon end Dir Acceptor Exon sequence|  |  |  |  |  |  |  |  |  |  |  |  |  |  |  |  |  |  |  |  |  |  |  |  |  |  |  |  |  |  |  |  |  |  |  |  |  |  |  |  |  |  |  |  |  |  |  |  |  |  |  |  |  |  |  |  |  |  |  |  |  |  |  |  |  |  |  |  |  |  |  |  |  |  |  |  |  |  |  |  |  |  |  |  |  |  |  |  |  |  |  |  |  |  |  |  |  |  |  |  |  |  |  |  | | --- | --- | --- | --- | --- | --- | --- | --- | --- | --- | --- | --- | --- | --- | --- | --- | --- | --- | --- | --- | --- | --- | --- | --- | --- | --- | --- | --- | --- | --- | --- | --- | --- | --- | --- | --- | --- | --- | --- | --- | --- | --- | --- | --- | --- | --- | --- | --- | --- | --- | --- | --- | --- | --- | --- | --- | --- | --- | --- | --- | --- | --- | --- | --- | --- | --- | --- | --- | --- | --- | --- | --- | --- | --- | --- | --- | --- | --- | --- | --- | --- | --- | --- | --- | --- | --- | --- | --- | --- | --- | --- | --- | --- | --- | --- | --- | --- | --- | --- | --- | --- | --- | --- | --- | | Human hg19 chr14 52985890 52985996 - **AG** | TGAAGTGAAATATATTACCAACCTGGAAGACCTTCAGAACATAGAAAATGCTCTGAAAGGAAAAGCAAATATTATATTCTCATATGTAAGAGCCATTGGAATACCAG|  |  |  |  |  |  |  |  |  |  |  |  |  |  |  |  |  |  |  |  |  |  |  |  |  |  |  |  |  |  |  |  |  |  |  |  |  |  |  |  |  |  |  |  |  |  |  |  |  |  |  |  |  |  |  |  |  |  |  |  |  |  |  |  |  |  |  |  |  |  |  |  |  |  |  |  |  |  |  |  |  |  |  |  |  |  |  |  |  |  |  |  |  |  |  |  | | --- | --- | --- | --- | --- | --- | --- | --- | --- | --- | --- | --- | --- | --- | --- | --- | --- | --- | --- | --- | --- | --- | --- | --- | --- | --- | --- | --- | --- | --- | --- | --- | --- | --- | --- | --- | --- | --- | --- | --- | --- | --- | --- | --- | --- | --- | --- | --- | --- | --- | --- | --- | --- | --- | --- | --- | --- | --- | --- | --- | --- | --- | --- | --- | --- | --- | --- | --- | --- | --- | --- | --- | --- | --- | --- | --- | --- | --- | --- | --- | --- | --- | --- | --- | --- | --- | --- | --- | --- | --- | --- | --- | --- | --- | --- | --- | | Chimp panTro2 chr14 51752640 51752746 - **AA** | TGAAGTGAAATATATTACCAACCTGGAAGACCTTCAGAACATAGAAAATGCTCTGAAAGGAAAAGCAAATATTATATTCTCATATGTAAGAGCCATTGGAATACCAG|  |  |  |  |  |  |  |  |  |  |  |  |  |  |  |  |  |  |  |  |  |  |  |  |  |  |  |  |  |  |  |  |  |  |  |  |  |  |  |  |  |  |  |  |  |  |  |  |  |  |  |  |  |  |  |  |  |  |  |  |  |  |  |  |  |  |  |  |  |  |  |  |  |  |  |  |  |  |  |  |  |  |  |  |  |  |  |  | | --- | --- | --- | --- | --- | --- | --- | --- | --- | --- | --- | --- | --- | --- | --- | --- | --- | --- | --- | --- | --- | --- | --- | --- | --- | --- | --- | --- | --- | --- | --- | --- | --- | --- | --- | --- | --- | --- | --- | --- | --- | --- | --- | --- | --- | --- | --- | --- | --- | --- | --- | --- | --- | --- | --- | --- | --- | --- | --- | --- | --- | --- | --- | --- | --- | --- | --- | --- | --- | --- | --- | --- | --- | --- | --- | --- | --- | --- | --- | --- | --- | --- | --- | --- | --- | --- | --- | --- | | Gorilla gorGor1 Supercontig\_0250740 4444 4550 - **AA** | TGAAGTGAAATATATTACCAACCTGGAAGACCTTCAGAACATAGAAAATGCTCTGAAAGGAAAAGCAAATATTATATTCTCATATGTAAGAGCCATTGGAATACCAG|  |  |  |  |  |  |  |  |  |  |  |  |  |  |  |  |  |  |  |  |  |  |  |  |  |  |  |  |  |  |  |  |  |  |  |  |  |  |  |  |  |  |  |  |  |  |  |  |  |  |  |  |  |  |  |  |  |  |  |  |  |  |  |  |  |  |  |  |  |  |  |  |  |  |  |  |  |  |  |  | | --- | --- | --- | --- | --- | --- | --- | --- | --- | --- | --- | --- | --- | --- | --- | --- | --- | --- | --- | --- | --- | --- | --- | --- | --- | --- | --- | --- | --- | --- | --- | --- | --- | --- | --- | --- | --- | --- | --- | --- | --- | --- | --- | --- | --- | --- | --- | --- | --- | --- | --- | --- | --- | --- | --- | --- | --- | --- | --- | --- | --- | --- | --- | --- | --- | --- | --- | --- | --- | --- | --- | --- | --- | --- | --- | --- | --- | --- | --- | --- | | Orangutan ponAbe2 chr14 52977264 52977370 - **AA** | TGAAGTGAAATATATTACCAACCTGGAAGACCTTCAGAACATAGAAAATGCTCTGAAAGGAAAAGCAAATATTATATTCTCATATGTAAGAGCCATTGGAATACCAG|  |  |  |  |  |  |  |  |  |  |  |  |  |  |  |  |  |  |  |  |  |  |  |  |  |  |  |  |  |  |  |  |  |  |  |  |  |  |  |  |  |  |  |  |  |  |  |  |  |  |  |  |  |  |  |  |  |  |  |  |  |  |  |  |  |  |  |  |  |  |  |  | | --- | --- | --- | --- | --- | --- | --- | --- | --- | --- | --- | --- | --- | --- | --- | --- | --- | --- | --- | --- | --- | --- | --- | --- | --- | --- | --- | --- | --- | --- | --- | --- | --- | --- | --- | --- | --- | --- | --- | --- | --- | --- | --- | --- | --- | --- | --- | --- | --- | --- | --- | --- | --- | --- | --- | --- | --- | --- | --- | --- | --- | --- | --- | --- | --- | --- | --- | --- | --- | --- | --- | --- | | Rhesus rheMac2 chr7 115485721 115485827 - **AA** | TGAAGTGAAATATATTACCAACCTGGAAGACCTTCAGAACATAGAAAATGCTCTGAAAGGAAAAGCAAATATTATATTCTCATATGTAAGAGCCACTGGAATACCAG|  |  |  |  |  |  |  |  |  |  |  |  |  |  |  |  |  |  |  |  |  |  |  |  |  |  |  |  |  |  |  |  |  |  |  |  |  |  |  |  |  |  |  |  |  |  |  |  |  |  |  |  |  |  |  |  |  |  |  |  |  |  |  |  | | --- | --- | --- | --- | --- | --- | --- | --- | --- | --- | --- | --- | --- | --- | --- | --- | --- | --- | --- | --- | --- | --- | --- | --- | --- | --- | --- | --- | --- | --- | --- | --- | --- | --- | --- | --- | --- | --- | --- | --- | --- | --- | --- | --- | --- | --- | --- | --- | --- | --- | --- | --- | --- | --- | --- | --- | --- | --- | --- | --- | --- | --- | --- | --- | | Baboon papHam1 scaffold8127 45105 45211 + **AA** | TGAAGTGAAATATATTACCAACCTGGAAGACCTTCAGAACATAGAAAATGCTCTGAAAGGAAAAGCAAATATTATATTCTCATATGTGAGAGCCATTGGAATACCAG|  |  |  |  |  |  |  |  |  |  |  |  |  |  |  |  |  |  |  |  |  |  |  |  |  |  |  |  |  |  |  |  |  |  |  |  |  |  |  |  |  |  |  |  |  |  |  |  |  |  |  |  |  |  |  |  | | --- | --- | --- | --- | --- | --- | --- | --- | --- | --- | --- | --- | --- | --- | --- | --- | --- | --- | --- | --- | --- | --- | --- | --- | --- | --- | --- | --- | --- | --- | --- | --- | --- | --- | --- | --- | --- | --- | --- | --- | --- | --- | --- | --- | --- | --- | --- | --- | --- | --- | --- | --- | --- | --- | --- | --- | | Marmoset calJac1 Contig4476 179732 179838 - **AA** | TGAAGTGAAATATATTACTAACCTGGAAGACCTTCAAAACATAGAAAATGCTCTGAAAGGGAAAGCAAATATTGTATTCTCATATGTAAAAGCCATTGGGATACCAG|  |  |  |  |  |  |  |  |  |  |  |  |  |  |  |  |  |  |  |  |  |  |  |  |  |  |  |  |  |  |  |  |  |  |  |  |  |  |  |  |  |  |  |  |  |  |  |  | | --- | --- | --- | --- | --- | --- | --- | --- | --- | --- | --- | --- | --- | --- | --- | --- | --- | --- | --- | --- | --- | --- | --- | --- | --- | --- | --- | --- | --- | --- | --- | --- | --- | --- | --- | --- | --- | --- | --- | --- | --- | --- | --- | --- | --- | --- | --- | --- | | Tarsier tarSyr1 scaffold\_50758 463 569 - **AA** | TGAAGTAAAATATATTACTACCCTGGACGGCCTTCAGAATGTAGAAAATGCCATGAAAGGAAAAAAGAATATTGTATTTTCATATGTAAGAGCCATTGGTGTACCAG|  |  |  |  |  |  |  |  |  |  |  |  |  |  |  |  |  |  |  |  |  |  |  |  |  |  |  |  |  |  |  |  |  |  |  |  |  |  |  |  | | --- | --- | --- | --- | --- | --- | --- | --- | --- | --- | --- | --- | --- | --- | --- | --- | --- | --- | --- | --- | --- | --- | --- | --- | --- | --- | --- | --- | --- | --- | --- | --- | --- | --- | --- | --- | --- | --- | --- | --- | | Lemur micMur1 scaffold\_46693 4817 4923 - **AA** | TGAAGTAAAATATATTACCACCTTGGAAGACCTGCAGAACATAGAAAATGCTCTGAAAGGAAAGGAAAATATTGTATTCTCATATGTAAGAGCCATTGGAATACCAG|  |  |  |  |  |  |  |  |  |  |  |  |  |  |  |  |  |  |  |  |  |  |  |  |  |  |  |  |  |  |  |  | | --- | --- | --- | --- | --- | --- | --- | --- | --- | --- | --- | --- | --- | --- | --- | --- | --- | --- | --- | --- | --- | --- | --- | --- | --- | --- | --- | --- | --- | --- | --- | --- | | Galago otoGar1 scaffold\_15997.1-66443 855 961 - **AA** | TGAAGTGAAATATGTTACCACCCAGAAAGACCTGCAGAATGTAGAAAATGCTCTGAAAGGAAAAGAAAATATTATATTCTCATATGTAAGAGCCATCGGAATACCAG|  |  |  |  |  |  |  |  |  |  |  |  |  |  |  |  |  |  |  |  |  |  |  |  | | --- | --- | --- | --- | --- | --- | --- | --- | --- | --- | --- | --- | --- | --- | --- | --- | --- | --- | --- | --- | --- | --- | --- | --- | | Mouse mm9 chr14 45802721 45802827 - **AA** | TGAAGTAAAATATATTACCACCCTGGAAGACCTTCACAGCATAGAAAACTCTCTGAAAGGAAAATCAAATATGATATTCTCATACGTAGAAGCCATCGGAACACCAG|  |  |  |  |  |  |  |  |  |  |  |  |  |  |  |  | | --- | --- | --- | --- | --- | --- | --- | --- | --- | --- | --- | --- | --- | --- | --- | --- | | Cow bosTau4 chr10 11448674 11448780 + **AA** | TGAAGTAAAATATATTACCACCCTGGGAGACCTTCAGAACATAGAAAATGCTCTAAAAGGGAAAAGAAACATTGCATTCTCATATGTCAGAGCCATTGGAACACCAG|  |  |  |  |  |  |  |  | | --- | --- | --- | --- | --- | --- | --- | --- | | Dog canFam2 chr8 31746032 31746138 - **AA** | TGAAGTAAAATATATTACCACCCTGGAAGACCTGCAGAACATAGAAAATGCTCTGAAAGGAAAAGCCAACATTGCATTCGCCTACGTAAGAGCCATTGGAACACCAG | | | | | | | | | | | | | | | | | | | | | | | | | | | | | | | | | | | | | | | | | | | | | | | | | | | | | | | | | | | | | | | | | | | | | | | | | | | | | | | | | | | | | | | | | | | | | | | | | | |

**Alignment** (splice site sequences are in lowercase)  

```
Human      agTGAAGTGAAATATATTACCAACCTGGAAGACCTTCAGAACATAGAAAATGCTCTGAAAGGAAAAGCAAATATTATATT
Chimp      .a..............................................................................
Gorilla    .a..............................................................................
Orangutan  .a..............................................................................
Rhesus     .a..............................................................................
Baboon     .a..............................................................................
Marmoset   .a..................T.................A.......................G............G....
Tarsier    .a......A...........T.C......C.G.........TG..........CA...........AAG......G....
Lemur      .a......A.............C.T..........G.............................G.A.......G....
Galago     .a.............G......C..A.A.......G.....TG........................A............
Mouse      .a......A.............C...............C.G.........CT..............T.......G.....
Cow        .a......A.............C.....G...........................A.....G...AG...C...GC...
Dog        .a......A.............C............G................................C..C...GC...

Human      CTCATATGTAAGAGCCATTGGAATACCAGgt
Chimp      ...............................
Gorilla    ...............................
Orangutan  ...............................
Rhesus     .................C.............
Baboon     .........G.....................
Marmoset   ...........A.........G.........
Tarsier    T....................TG........
Lemur      ...............................
Galago     ..................C............
Mouse      ......C...GA......C....C.......
Cow        .........C.............C.......
Dog        .G.C..C................C.......
```

---

## 31. uc001xpi.2\_13\_13

**Summary**  

|  |  |  |  |  |  |  |  |  |  |  |  |  |  |  |  |  |  |  |  |  |  |  |  |  |  |
| --- | --- | --- | --- | --- | --- | --- | --- | --- | --- | --- | --- | --- | --- | --- | --- | --- | --- | --- | --- | --- | --- | --- | --- | --- | --- |
| No Exon ID Position (hg19) Dir Human acceptor Chimp acceptor Category Usage Gene symbol Protein accession mRNA accession Gene title Note|  |  |  |  |  |  |  |  |  |  |  |  |  | | --- | --- | --- | --- | --- | --- | --- | --- | --- | --- | --- | --- | --- | | 31 uc001xpi.2\_13\_13 chr14:74426256 - AG AA (A6) exonization; frameshift alternative ENTPD5 O75356 BC020966.2 ectonucleoside triphosphate diphosphohydrolase 5  | | | | | | | | | | | | | | | | | | | | | | | | | |

**Orthologs**  

|  |  |  |  |  |  |  |  |  |  |  |  |  |  |  |  |  |  |  |  |  |  |  |  |  |  |  |  |  |  |  |  |  |  |  |  |  |  |  |  |  |  |  |  |  |  |  |  |  |  |  |  |  |  |  |  |  |  |  |  |  |  |  |  |  |  |  |  |  |  |  |  |  |  |  |  |  |  |  |  |  |  |  |  |  |  |  |  |  |  |  |  |  |  |  |  |  |  |  |  |  |  |  |  |  |  |  |  |  |  |  |  |
| --- | --- | --- | --- | --- | --- | --- | --- | --- | --- | --- | --- | --- | --- | --- | --- | --- | --- | --- | --- | --- | --- | --- | --- | --- | --- | --- | --- | --- | --- | --- | --- | --- | --- | --- | --- | --- | --- | --- | --- | --- | --- | --- | --- | --- | --- | --- | --- | --- | --- | --- | --- | --- | --- | --- | --- | --- | --- | --- | --- | --- | --- | --- | --- | --- | --- | --- | --- | --- | --- | --- | --- | --- | --- | --- | --- | --- | --- | --- | --- | --- | --- | --- | --- | --- | --- | --- | --- | --- | --- | --- | --- | --- | --- | --- | --- | --- | --- | --- | --- | --- | --- | --- | --- | --- | --- | --- | --- | --- | --- | --- | --- |
| Species Assembly Chromosome Exon start Exon end Dir Acceptor Exon sequence|  |  |  |  |  |  |  |  |  |  |  |  |  |  |  |  |  |  |  |  |  |  |  |  |  |  |  |  |  |  |  |  |  |  |  |  |  |  |  |  |  |  |  |  |  |  |  |  |  |  |  |  |  |  |  |  |  |  |  |  |  |  |  |  |  |  |  |  |  |  |  |  |  |  |  |  |  |  |  |  |  |  |  |  |  |  |  |  |  |  |  |  |  |  |  |  |  |  |  |  |  |  |  |  | | --- | --- | --- | --- | --- | --- | --- | --- | --- | --- | --- | --- | --- | --- | --- | --- | --- | --- | --- | --- | --- | --- | --- | --- | --- | --- | --- | --- | --- | --- | --- | --- | --- | --- | --- | --- | --- | --- | --- | --- | --- | --- | --- | --- | --- | --- | --- | --- | --- | --- | --- | --- | --- | --- | --- | --- | --- | --- | --- | --- | --- | --- | --- | --- | --- | --- | --- | --- | --- | --- | --- | --- | --- | --- | --- | --- | --- | --- | --- | --- | --- | --- | --- | --- | --- | --- | --- | --- | --- | --- | --- | --- | --- | --- | --- | --- | --- | --- | --- | --- | --- | --- | --- | --- | | Human hg19 chr14 74426233 74426256 - **AG** | CACATCATCAGCTGGGTAAATTGA|  |  |  |  |  |  |  |  |  |  |  |  |  |  |  |  |  |  |  |  |  |  |  |  |  |  |  |  |  |  |  |  |  |  |  |  |  |  |  |  |  |  |  |  |  |  |  |  |  |  |  |  |  |  |  |  |  |  |  |  |  |  |  |  |  |  |  |  |  |  |  |  |  |  |  |  |  |  |  |  |  |  |  |  |  |  |  |  |  |  |  |  |  |  |  |  | | --- | --- | --- | --- | --- | --- | --- | --- | --- | --- | --- | --- | --- | --- | --- | --- | --- | --- | --- | --- | --- | --- | --- | --- | --- | --- | --- | --- | --- | --- | --- | --- | --- | --- | --- | --- | --- | --- | --- | --- | --- | --- | --- | --- | --- | --- | --- | --- | --- | --- | --- | --- | --- | --- | --- | --- | --- | --- | --- | --- | --- | --- | --- | --- | --- | --- | --- | --- | --- | --- | --- | --- | --- | --- | --- | --- | --- | --- | --- | --- | --- | --- | --- | --- | --- | --- | --- | --- | --- | --- | --- | --- | --- | --- | --- | --- | | Chimp panTro2 chr14 73665741 73665764 - **AA** | CACATCATCAGCTGGGTAAATTGA|  |  |  |  |  |  |  |  |  |  |  |  |  |  |  |  |  |  |  |  |  |  |  |  |  |  |  |  |  |  |  |  |  |  |  |  |  |  |  |  |  |  |  |  |  |  |  |  |  |  |  |  |  |  |  |  |  |  |  |  |  |  |  |  |  |  |  |  |  |  |  |  |  |  |  |  |  |  |  |  |  |  |  |  |  |  |  |  | | --- | --- | --- | --- | --- | --- | --- | --- | --- | --- | --- | --- | --- | --- | --- | --- | --- | --- | --- | --- | --- | --- | --- | --- | --- | --- | --- | --- | --- | --- | --- | --- | --- | --- | --- | --- | --- | --- | --- | --- | --- | --- | --- | --- | --- | --- | --- | --- | --- | --- | --- | --- | --- | --- | --- | --- | --- | --- | --- | --- | --- | --- | --- | --- | --- | --- | --- | --- | --- | --- | --- | --- | --- | --- | --- | --- | --- | --- | --- | --- | --- | --- | --- | --- | --- | --- | --- | --- | | Gorilla gorGor1 Supercontig\_0006692 23905 23928 - **AA** | CACATCATCAGCTGGGTAAATTGA|  |  |  |  |  |  |  |  |  |  |  |  |  |  |  |  |  |  |  |  |  |  |  |  |  |  |  |  |  |  |  |  |  |  |  |  |  |  |  |  |  |  |  |  |  |  |  |  |  |  |  |  |  |  |  |  |  |  |  |  |  |  |  |  |  |  |  |  |  |  |  |  |  |  |  |  |  |  |  |  | | --- | --- | --- | --- | --- | --- | --- | --- | --- | --- | --- | --- | --- | --- | --- | --- | --- | --- | --- | --- | --- | --- | --- | --- | --- | --- | --- | --- | --- | --- | --- | --- | --- | --- | --- | --- | --- | --- | --- | --- | --- | --- | --- | --- | --- | --- | --- | --- | --- | --- | --- | --- | --- | --- | --- | --- | --- | --- | --- | --- | --- | --- | --- | --- | --- | --- | --- | --- | --- | --- | --- | --- | --- | --- | --- | --- | --- | --- | --- | --- | | Orangutan ponAbe2 chr14 75126553 75126577 - **AA** | CACATCATCAGGCTGGGTAAATTAA|  |  |  |  |  |  |  |  |  |  |  |  |  |  |  |  |  |  |  |  |  |  |  |  |  |  |  |  |  |  |  |  |  |  |  |  |  |  |  |  |  |  |  |  |  |  |  |  |  |  |  |  |  |  |  |  |  |  |  |  |  |  |  |  |  |  |  |  |  |  |  |  | | --- | --- | --- | --- | --- | --- | --- | --- | --- | --- | --- | --- | --- | --- | --- | --- | --- | --- | --- | --- | --- | --- | --- | --- | --- | --- | --- | --- | --- | --- | --- | --- | --- | --- | --- | --- | --- | --- | --- | --- | --- | --- | --- | --- | --- | --- | --- | --- | --- | --- | --- | --- | --- | --- | --- | --- | --- | --- | --- | --- | --- | --- | --- | --- | --- | --- | --- | --- | --- | --- | --- | --- | | Rhesus rheMac2 chr7 137052757 137052780 - **AA** | CACATCACTAGCTGGGTATATTGA|  |  |  |  |  |  |  |  |  |  |  |  |  |  |  |  |  |  |  |  |  |  |  |  |  |  |  |  |  |  |  |  |  |  |  |  |  |  |  |  |  |  |  |  |  |  |  |  |  |  |  |  |  |  |  |  |  |  |  |  |  |  |  |  | | --- | --- | --- | --- | --- | --- | --- | --- | --- | --- | --- | --- | --- | --- | --- | --- | --- | --- | --- | --- | --- | --- | --- | --- | --- | --- | --- | --- | --- | --- | --- | --- | --- | --- | --- | --- | --- | --- | --- | --- | --- | --- | --- | --- | --- | --- | --- | --- | --- | --- | --- | --- | --- | --- | --- | --- | --- | --- | --- | --- | --- | --- | --- | --- | | Baboon papHam1 scaffold1627 129920 129943 + **AA** | CACATCATTAGCTGGGTATATTGA|  |  |  |  |  |  |  |  |  |  |  |  |  |  |  |  |  |  |  |  |  |  |  |  |  |  |  |  |  |  |  |  |  |  |  |  |  |  |  |  |  |  |  |  |  |  |  |  |  |  |  |  |  |  |  |  | | --- | --- | --- | --- | --- | --- | --- | --- | --- | --- | --- | --- | --- | --- | --- | --- | --- | --- | --- | --- | --- | --- | --- | --- | --- | --- | --- | --- | --- | --- | --- | --- | --- | --- | --- | --- | --- | --- | --- | --- | --- | --- | --- | --- | --- | --- | --- | --- | --- | --- | --- | --- | --- | --- | --- | --- | | Marmoset calJac1 Contig1997 106202 106225 - **AA** | CACATCATCAGCCAGGTAAGTTGA|  |  |  |  |  |  |  |  |  |  |  |  |  |  |  |  |  |  |  |  |  |  |  |  |  |  |  |  |  |  |  |  |  |  |  |  |  |  |  |  |  |  |  |  |  |  |  |  | | --- | --- | --- | --- | --- | --- | --- | --- | --- | --- | --- | --- | --- | --- | --- | --- | --- | --- | --- | --- | --- | --- | --- | --- | --- | --- | --- | --- | --- | --- | --- | --- | --- | --- | --- | --- | --- | --- | --- | --- | --- | --- | --- | --- | --- | --- | --- | --- | | Tarsier tarSyr1 scaffold\_22245 2039 2062 - **AA** | CACATCATCAGCTGGGGACGTTGA|  |  |  |  |  |  |  |  |  |  |  |  |  |  |  |  |  |  |  |  |  |  |  |  |  |  |  |  |  |  |  |  |  |  |  |  |  |  |  |  | | --- | --- | --- | --- | --- | --- | --- | --- | --- | --- | --- | --- | --- | --- | --- | --- | --- | --- | --- | --- | --- | --- | --- | --- | --- | --- | --- | --- | --- | --- | --- | --- | --- | --- | --- | --- | --- | --- | --- | --- | | Lemur micMur1 scaffold\_4911 84368 84391 - **AA** | CACATGATCAGCTGGATAAGTTGA|  |  |  |  |  |  |  |  |  |  |  |  |  |  |  |  |  |  |  |  |  |  |  |  |  |  |  |  |  |  |  |  | | --- | --- | --- | --- | --- | --- | --- | --- | --- | --- | --- | --- | --- | --- | --- | --- | --- | --- | --- | --- | --- | --- | --- | --- | --- | --- | --- | --- | --- | --- | --- | --- | | Galago otoGar1 scaffold\_115816.1-74314 36970 36992 - **AA** | CACATCATTAGCTGGATACATTA|  |  |  |  |  |  |  |  |  |  |  |  |  |  |  |  |  |  |  |  |  |  |  |  | | --- | --- | --- | --- | --- | --- | --- | --- | --- | --- | --- | --- | --- | --- | --- | --- | --- | --- | --- | --- | --- | --- | --- | --- | | Mouse mm9 chr12 85712516 85712538 - **AG** | CACACCAGCAGCTGGACAAGGGG|  |  |  |  |  |  |  |  |  |  |  |  |  |  |  |  | | --- | --- | --- | --- | --- | --- | --- | --- | --- | --- | --- | --- | --- | --- | --- | --- | | Cow bosTau4 chr10 87811771 87811788 - **AA** | CACATCATCGGCAGGTGA|  |  |  |  |  |  |  |  | | --- | --- | --- | --- | --- | --- | --- | --- | | Dog canFam2 chr8 50223183 50223206 - **AA** | CACATAATCAGCTAGACAAGTTCA | | | | | | | | | | | | | | | | | | | | | | | | | | | | | | | | | | | | | | | | | | | | | | | | | | | | | | | | | | | | | | | | | | | | | | | | | | | | | | | | | | | | | | | | | | | | | | | | | | |

**Alignment** (splice site sequences are in lowercase)  

```
Human      agCACATCATCA-GCTGGGTAAATTGA
Chimp      .a..........-..............
Gorilla    .a..........-..............
Orangutan  .a..........G............A.
Rhesus     .a.......CT.-........T.....
Baboon     .a........T.-........T.....
Marmoset   .a..........-..CA.....G....
Tarsier    .a..........-......G.CG....
Lemur      .a.....G....-.....A...G....
Galago     .a........T.-.....A..C...-.
Mouse      ......C..G..-.....AC..G-G.G
Cow        .a.........G-..A..------...
Dog        .a.....A....-...A.AC..G..C.
```

---

## 32. uc001xxw.1\_6\_6

**Summary**  

|  |  |  |  |  |  |  |  |  |  |  |  |  |  |  |  |  |  |  |  |  |  |  |  |  |  |
| --- | --- | --- | --- | --- | --- | --- | --- | --- | --- | --- | --- | --- | --- | --- | --- | --- | --- | --- | --- | --- | --- | --- | --- | --- | --- |
| No Exon ID Position (hg19) Dir Human acceptor Chimp acceptor Category Usage Gene symbol Protein accession mRNA accession Gene title Note|  |  |  |  |  |  |  |  |  |  |  |  |  | | --- | --- | --- | --- | --- | --- | --- | --- | --- | --- | --- | --- | --- | | 32 uc001xxw.1\_6\_6 chr14:90391021 - AG GG (A6) exonization; frameshift alternative C14orf143 Q9BUY7-3 AK298930.1 hypothetical protein LOC90141 (EF-hand calcium-binding domain-containing protein 11)  | | | | | | | | | | | | | | | | | | | | | | | | | |

**Orthologs**  

|  |  |  |  |  |  |  |  |  |  |  |  |  |  |  |  |  |  |  |  |  |  |  |  |  |  |  |  |  |  |  |  |  |  |  |  |  |  |  |  |  |  |  |  |  |  |  |  |  |  |  |  |  |  |  |  |  |  |  |  |  |  |  |  |  |  |  |  |  |  |  |  |  |  |  |  |  |  |  |  |  |  |  |  |  |  |  |  |  |  |  |  |  |  |  |  |
| --- | --- | --- | --- | --- | --- | --- | --- | --- | --- | --- | --- | --- | --- | --- | --- | --- | --- | --- | --- | --- | --- | --- | --- | --- | --- | --- | --- | --- | --- | --- | --- | --- | --- | --- | --- | --- | --- | --- | --- | --- | --- | --- | --- | --- | --- | --- | --- | --- | --- | --- | --- | --- | --- | --- | --- | --- | --- | --- | --- | --- | --- | --- | --- | --- | --- | --- | --- | --- | --- | --- | --- | --- | --- | --- | --- | --- | --- | --- | --- | --- | --- | --- | --- | --- | --- | --- | --- | --- | --- | --- | --- | --- | --- | --- | --- |
| Species Assembly Chromosome Exon start Exon end Dir Acceptor Exon sequence|  |  |  |  |  |  |  |  |  |  |  |  |  |  |  |  |  |  |  |  |  |  |  |  |  |  |  |  |  |  |  |  |  |  |  |  |  |  |  |  |  |  |  |  |  |  |  |  |  |  |  |  |  |  |  |  |  |  |  |  |  |  |  |  |  |  |  |  |  |  |  |  |  |  |  |  |  |  |  |  |  |  |  |  |  |  |  |  | | --- | --- | --- | --- | --- | --- | --- | --- | --- | --- | --- | --- | --- | --- | --- | --- | --- | --- | --- | --- | --- | --- | --- | --- | --- | --- | --- | --- | --- | --- | --- | --- | --- | --- | --- | --- | --- | --- | --- | --- | --- | --- | --- | --- | --- | --- | --- | --- | --- | --- | --- | --- | --- | --- | --- | --- | --- | --- | --- | --- | --- | --- | --- | --- | --- | --- | --- | --- | --- | --- | --- | --- | --- | --- | --- | --- | --- | --- | --- | --- | --- | --- | --- | --- | --- | --- | --- | --- | | Human hg19 chr14 90391003 90391021 - **AG** | AGGCATTTTCAGTGCATGA|  |  |  |  |  |  |  |  |  |  |  |  |  |  |  |  |  |  |  |  |  |  |  |  |  |  |  |  |  |  |  |  |  |  |  |  |  |  |  |  |  |  |  |  |  |  |  |  |  |  |  |  |  |  |  |  |  |  |  |  |  |  |  |  |  |  |  |  |  |  |  |  |  |  |  |  |  |  |  |  | | --- | --- | --- | --- | --- | --- | --- | --- | --- | --- | --- | --- | --- | --- | --- | --- | --- | --- | --- | --- | --- | --- | --- | --- | --- | --- | --- | --- | --- | --- | --- | --- | --- | --- | --- | --- | --- | --- | --- | --- | --- | --- | --- | --- | --- | --- | --- | --- | --- | --- | --- | --- | --- | --- | --- | --- | --- | --- | --- | --- | --- | --- | --- | --- | --- | --- | --- | --- | --- | --- | --- | --- | --- | --- | --- | --- | --- | --- | --- | --- | | Chimp panTro2 chr14 90041692 90041710 - **GG** | AGGCATTTTCAGTGCATGA|  |  |  |  |  |  |  |  |  |  |  |  |  |  |  |  |  |  |  |  |  |  |  |  |  |  |  |  |  |  |  |  |  |  |  |  |  |  |  |  |  |  |  |  |  |  |  |  |  |  |  |  |  |  |  |  |  |  |  |  |  |  |  |  |  |  |  |  |  |  |  |  | | --- | --- | --- | --- | --- | --- | --- | --- | --- | --- | --- | --- | --- | --- | --- | --- | --- | --- | --- | --- | --- | --- | --- | --- | --- | --- | --- | --- | --- | --- | --- | --- | --- | --- | --- | --- | --- | --- | --- | --- | --- | --- | --- | --- | --- | --- | --- | --- | --- | --- | --- | --- | --- | --- | --- | --- | --- | --- | --- | --- | --- | --- | --- | --- | --- | --- | --- | --- | --- | --- | --- | --- | | Gorilla gorGor1 Supercontig\_0214551 22 40 - **GG** | AGGCATTTTCAGTGCATGA|  |  |  |  |  |  |  |  |  |  |  |  |  |  |  |  |  |  |  |  |  |  |  |  |  |  |  |  |  |  |  |  |  |  |  |  |  |  |  |  |  |  |  |  |  |  |  |  |  |  |  |  |  |  |  |  |  |  |  |  |  |  |  |  | | --- | --- | --- | --- | --- | --- | --- | --- | --- | --- | --- | --- | --- | --- | --- | --- | --- | --- | --- | --- | --- | --- | --- | --- | --- | --- | --- | --- | --- | --- | --- | --- | --- | --- | --- | --- | --- | --- | --- | --- | --- | --- | --- | --- | --- | --- | --- | --- | --- | --- | --- | --- | --- | --- | --- | --- | --- | --- | --- | --- | --- | --- | --- | --- | | Orangutan ponAbe2 chr14 91226506 91226524 - **GG** | AGGCGTTTTCGGTGCATGA|  |  |  |  |  |  |  |  |  |  |  |  |  |  |  |  |  |  |  |  |  |  |  |  |  |  |  |  |  |  |  |  |  |  |  |  |  |  |  |  |  |  |  |  |  |  |  |  |  |  |  |  |  |  |  |  | | --- | --- | --- | --- | --- | --- | --- | --- | --- | --- | --- | --- | --- | --- | --- | --- | --- | --- | --- | --- | --- | --- | --- | --- | --- | --- | --- | --- | --- | --- | --- | --- | --- | --- | --- | --- | --- | --- | --- | --- | --- | --- | --- | --- | --- | --- | --- | --- | --- | --- | --- | --- | --- | --- | --- | --- | | Rhesus rheMac2 chr7 153139877 153139895 - **GG** | AGGCATTTTTGGTTCATGA|  |  |  |  |  |  |  |  |  |  |  |  |  |  |  |  |  |  |  |  |  |  |  |  |  |  |  |  |  |  |  |  |  |  |  |  |  |  |  |  |  |  |  |  |  |  |  |  | | --- | --- | --- | --- | --- | --- | --- | --- | --- | --- | --- | --- | --- | --- | --- | --- | --- | --- | --- | --- | --- | --- | --- | --- | --- | --- | --- | --- | --- | --- | --- | --- | --- | --- | --- | --- | --- | --- | --- | --- | --- | --- | --- | --- | --- | --- | --- | --- | | Baboon papHam1 scaffold51 435258 435276 + **GG** | AGGCATTTTTGGTTCATGA|  |  |  |  |  |  |  |  |  |  |  |  |  |  |  |  |  |  |  |  |  |  |  |  |  |  |  |  |  |  |  |  |  |  |  |  |  |  |  |  | | --- | --- | --- | --- | --- | --- | --- | --- | --- | --- | --- | --- | --- | --- | --- | --- | --- | --- | --- | --- | --- | --- | --- | --- | --- | --- | --- | --- | --- | --- | --- | --- | --- | --- | --- | --- | --- | --- | --- | --- | | Marmoset calJac1 Contig7268 8964 8982 + **GG** | AGGCATTTTCAGTTCATGA|  |  |  |  |  |  |  |  |  |  |  |  |  |  |  |  |  |  |  |  |  |  |  |  |  |  |  |  |  |  |  |  | | --- | --- | --- | --- | --- | --- | --- | --- | --- | --- | --- | --- | --- | --- | --- | --- | --- | --- | --- | --- | --- | --- | --- | --- | --- | --- | --- | --- | --- | --- | --- | --- | | Lemur micMur1 scaffold\_33 1005922 1005940 - **GG** | GGACATTTTCAGCTCATGA|  |  |  |  |  |  |  |  |  |  |  |  |  |  |  |  |  |  |  |  |  |  |  |  | | --- | --- | --- | --- | --- | --- | --- | --- | --- | --- | --- | --- | --- | --- | --- | --- | --- | --- | --- | --- | --- | --- | --- | --- | | Galago otoGar1 scaffold\_116030.1-389768 251149 251167 - **GG** | GGACATTTTCAGTTCATGA|  |  |  |  |  |  |  |  |  |  |  |  |  |  |  |  | | --- | --- | --- | --- | --- | --- | --- | --- | --- | --- | --- | --- | --- | --- | --- | --- | | Cow bosTau4 chr10 104643186 104643204 - **AG** | AGGCATTTTCAGTTCCTGA|  |  |  |  |  |  |  |  | | --- | --- | --- | --- | --- | --- | --- | --- | | Dog canFam2 chr8 64035752 64035770 - **GG** | GGTCGCTTGCAATTCACAA | | | | | | | | | | | | | | | | | | | | | | | | | | | | | | | | | | | | | | | | | | | | | | | | | | | | | | | | | | | | | | | | | | | | | | | | | | | | | | | | | | | | |

**Alignment** (splice site sequences are in lowercase)  

```
Human      agAGGCATTTTCAGTGCATGA
Chimp      g....................
Gorilla    g....................
Orangutan  g.....G.....G........
Rhesus     g..........TG..T.....
Baboon     g..........TG..T.....
Marmoset   g..............T.....
Lemur      g.G.A.........CT.....
Galago     g.G.A..........T.....
Cow        ...............T.C...
Dog        g.G.T.GC..G..A.T..CA.
```

---

## 33. uc002elm.1\_2\_8

**Summary**  

|  |  |  |  |  |  |  |  |  |  |  |  |  |  |  |  |  |  |  |  |  |  |  |  |  |  |
| --- | --- | --- | --- | --- | --- | --- | --- | --- | --- | --- | --- | --- | --- | --- | --- | --- | --- | --- | --- | --- | --- | --- | --- | --- | --- |
| No Exon ID Position (hg19) Dir Human acceptor Chimp acceptor Category Usage Gene symbol Protein accession mRNA accession Gene title Note|  |  |  |  |  |  |  |  |  |  |  |  |  | | --- | --- | --- | --- | --- | --- | --- | --- | --- | --- | --- | --- | --- | | 33 uc002elm.1\_2\_8 chr16:57473207 - AG GG (A3) shift; decrease; inframe alternative CIAPIN1 Q6FI81 AL136613.1 cytokine induced apoptosis inhibitor 1  | | | | | | | | | | | | | | | | | | | | | | | | | |

**Orthologs**  

|  |  |  |  |  |  |  |  |  |  |  |  |  |  |  |  |  |  |  |  |  |  |  |  |  |  |  |  |  |  |  |  |  |  |  |  |  |  |  |  |  |  |  |  |  |  |  |  |  |  |  |  |  |  |  |  |  |  |  |  |  |  |  |  |  |  |  |  |  |  |  |  |  |  |  |  |  |  |  |  |  |  |  |  |  |  |  |  |  |  |  |  |  |  |  |  |  |  |  |  |  |  |  |  |
| --- | --- | --- | --- | --- | --- | --- | --- | --- | --- | --- | --- | --- | --- | --- | --- | --- | --- | --- | --- | --- | --- | --- | --- | --- | --- | --- | --- | --- | --- | --- | --- | --- | --- | --- | --- | --- | --- | --- | --- | --- | --- | --- | --- | --- | --- | --- | --- | --- | --- | --- | --- | --- | --- | --- | --- | --- | --- | --- | --- | --- | --- | --- | --- | --- | --- | --- | --- | --- | --- | --- | --- | --- | --- | --- | --- | --- | --- | --- | --- | --- | --- | --- | --- | --- | --- | --- | --- | --- | --- | --- | --- | --- | --- | --- | --- | --- | --- | --- | --- | --- | --- | --- | --- |
| Species Assembly Chromosome Exon start Exon end Dir Acceptor Exon sequence|  |  |  |  |  |  |  |  |  |  |  |  |  |  |  |  |  |  |  |  |  |  |  |  |  |  |  |  |  |  |  |  |  |  |  |  |  |  |  |  |  |  |  |  |  |  |  |  |  |  |  |  |  |  |  |  |  |  |  |  |  |  |  |  |  |  |  |  |  |  |  |  |  |  |  |  |  |  |  |  |  |  |  |  |  |  |  |  |  |  |  |  |  |  |  |  | | --- | --- | --- | --- | --- | --- | --- | --- | --- | --- | --- | --- | --- | --- | --- | --- | --- | --- | --- | --- | --- | --- | --- | --- | --- | --- | --- | --- | --- | --- | --- | --- | --- | --- | --- | --- | --- | --- | --- | --- | --- | --- | --- | --- | --- | --- | --- | --- | --- | --- | --- | --- | --- | --- | --- | --- | --- | --- | --- | --- | --- | --- | --- | --- | --- | --- | --- | --- | --- | --- | --- | --- | --- | --- | --- | --- | --- | --- | --- | --- | --- | --- | --- | --- | --- | --- | --- | --- | --- | --- | --- | --- | --- | --- | --- | --- | | Human hg19 chr16 57473094 57473207 - **AG** | GTTTAGTCCCAGGAAGCACCACTCTGCACAGTGCTGAGATTTTGGCTGAAATCGCCCGGATCCTTCGGCCTGGTGGATGTCTTTTTCTGAAGGAGCCAGTAGAGACAGCTGTAG|  |  |  |  |  |  |  |  |  |  |  |  |  |  |  |  |  |  |  |  |  |  |  |  |  |  |  |  |  |  |  |  |  |  |  |  |  |  |  |  |  |  |  |  |  |  |  |  |  |  |  |  |  |  |  |  |  |  |  |  |  |  |  |  |  |  |  |  |  |  |  |  |  |  |  |  |  |  |  |  |  |  |  |  |  |  |  |  | | --- | --- | --- | --- | --- | --- | --- | --- | --- | --- | --- | --- | --- | --- | --- | --- | --- | --- | --- | --- | --- | --- | --- | --- | --- | --- | --- | --- | --- | --- | --- | --- | --- | --- | --- | --- | --- | --- | --- | --- | --- | --- | --- | --- | --- | --- | --- | --- | --- | --- | --- | --- | --- | --- | --- | --- | --- | --- | --- | --- | --- | --- | --- | --- | --- | --- | --- | --- | --- | --- | --- | --- | --- | --- | --- | --- | --- | --- | --- | --- | --- | --- | --- | --- | --- | --- | --- | --- | | Chimp panTro2 chr16 56885272 56885385 - **GG** | GTTTAGTCCCAGGAAGCACCACTCTGCACAGTGCTGAGATTTTGGCTGAAATCGCCCGGATCCTTCGGCCTGGTGGATGTCTTTTTCTGAAAGAGCCAGTAGAGACAGCTGTAG|  |  |  |  |  |  |  |  |  |  |  |  |  |  |  |  |  |  |  |  |  |  |  |  |  |  |  |  |  |  |  |  |  |  |  |  |  |  |  |  |  |  |  |  |  |  |  |  |  |  |  |  |  |  |  |  |  |  |  |  |  |  |  |  |  |  |  |  |  |  |  |  |  |  |  |  |  |  |  |  | | --- | --- | --- | --- | --- | --- | --- | --- | --- | --- | --- | --- | --- | --- | --- | --- | --- | --- | --- | --- | --- | --- | --- | --- | --- | --- | --- | --- | --- | --- | --- | --- | --- | --- | --- | --- | --- | --- | --- | --- | --- | --- | --- | --- | --- | --- | --- | --- | --- | --- | --- | --- | --- | --- | --- | --- | --- | --- | --- | --- | --- | --- | --- | --- | --- | --- | --- | --- | --- | --- | --- | --- | --- | --- | --- | --- | --- | --- | --- | --- | | Gorilla gorGor1 Supercontig\_0056356 10154 10267 + **GG** | GTTTAGTCCCAGGAAGCACCACTCTGCACAGTGCTGAGATTTTGGCTGAAATCGCCCGGATCCTTCGGCCTGGTGGATGTCTTTTTCTGAAAGAGCCAGTAGAGACAGCTGTAG|  |  |  |  |  |  |  |  |  |  |  |  |  |  |  |  |  |  |  |  |  |  |  |  |  |  |  |  |  |  |  |  |  |  |  |  |  |  |  |  |  |  |  |  |  |  |  |  |  |  |  |  |  |  |  |  |  |  |  |  |  |  |  |  |  |  |  |  |  |  |  |  | | --- | --- | --- | --- | --- | --- | --- | --- | --- | --- | --- | --- | --- | --- | --- | --- | --- | --- | --- | --- | --- | --- | --- | --- | --- | --- | --- | --- | --- | --- | --- | --- | --- | --- | --- | --- | --- | --- | --- | --- | --- | --- | --- | --- | --- | --- | --- | --- | --- | --- | --- | --- | --- | --- | --- | --- | --- | --- | --- | --- | --- | --- | --- | --- | --- | --- | --- | --- | --- | --- | --- | --- | | Orangutan ponAbe2 chr16 44676069 44676182 - **GG** | GTTTAGTCCCGGGAAGCACCACTCTGCACAGTGCTGAGATTTTGGCTGAAATCGCCCGGATCCTTCGGCCTGGTGGATGTCTTTTTCTGAAAGAGCCAGTAGAGACAGCTGTAG|  |  |  |  |  |  |  |  |  |  |  |  |  |  |  |  |  |  |  |  |  |  |  |  |  |  |  |  |  |  |  |  |  |  |  |  |  |  |  |  |  |  |  |  |  |  |  |  |  |  |  |  |  |  |  |  |  |  |  |  |  |  |  |  | | --- | --- | --- | --- | --- | --- | --- | --- | --- | --- | --- | --- | --- | --- | --- | --- | --- | --- | --- | --- | --- | --- | --- | --- | --- | --- | --- | --- | --- | --- | --- | --- | --- | --- | --- | --- | --- | --- | --- | --- | --- | --- | --- | --- | --- | --- | --- | --- | --- | --- | --- | --- | --- | --- | --- | --- | --- | --- | --- | --- | --- | --- | --- | --- | | Rhesus rheMac2 chr20 55787038 55787151 - **GG** | GTTTAGTCCCGCGCAGCACCACTCTGCACAGTGCTGAGATTTTGGCTGAAATCGCCCGGATCCTTCGGCCTGGTGGATGTCTTTTTCTGAAAGAGCCGGTAGAGACAGCTGTAG|  |  |  |  |  |  |  |  |  |  |  |  |  |  |  |  |  |  |  |  |  |  |  |  |  |  |  |  |  |  |  |  |  |  |  |  |  |  |  |  |  |  |  |  |  |  |  |  |  |  |  |  |  |  |  |  | | --- | --- | --- | --- | --- | --- | --- | --- | --- | --- | --- | --- | --- | --- | --- | --- | --- | --- | --- | --- | --- | --- | --- | --- | --- | --- | --- | --- | --- | --- | --- | --- | --- | --- | --- | --- | --- | --- | --- | --- | --- | --- | --- | --- | --- | --- | --- | --- | --- | --- | --- | --- | --- | --- | --- | --- | | Baboon papHam1 scaffold2237 34748 34861 - **GG** | GTTTAGTCCCGGGAAGCACCACTCTGCACAGTGCTGAGATTTTGGCTGAAATCGCCCGGATCCTTCGGCCTGGTGGATGTCTTTTTCTGAAAGAGCCGGTAGAGACAGCTGTAG|  |  |  |  |  |  |  |  |  |  |  |  |  |  |  |  |  |  |  |  |  |  |  |  |  |  |  |  |  |  |  |  |  |  |  |  |  |  |  |  |  |  |  |  |  |  |  |  | | --- | --- | --- | --- | --- | --- | --- | --- | --- | --- | --- | --- | --- | --- | --- | --- | --- | --- | --- | --- | --- | --- | --- | --- | --- | --- | --- | --- | --- | --- | --- | --- | --- | --- | --- | --- | --- | --- | --- | --- | --- | --- | --- | --- | --- | --- | --- | --- | | Marmoset calJac1 Contig485 132855 132968 + **GG** | GTTTAGTCCCGGGAAGCACCACTCTGCACAGTGCTGAGATTTTGGCTGAAATCGCCAGGATCCTTCGACCTGGTGGATGTCTTTTTCTGAAAGAGCCAGTAGAGACAGCTGTAG|  |  |  |  |  |  |  |  |  |  |  |  |  |  |  |  |  |  |  |  |  |  |  |  |  |  |  |  |  |  |  |  |  |  |  |  |  |  |  |  | | --- | --- | --- | --- | --- | --- | --- | --- | --- | --- | --- | --- | --- | --- | --- | --- | --- | --- | --- | --- | --- | --- | --- | --- | --- | --- | --- | --- | --- | --- | --- | --- | --- | --- | --- | --- | --- | --- | --- | --- | | Tarsier tarSyr1 scaffold\_85910 757 870 + **GG** | GTATCATCCCGGGAAGCACCACTCTGCACAGTGCTGAGGTTTTGGCTGAGATGGCCCGGATCCTTCGGCCTGGTGGATGTCTTATTCTGAAAGAGCCAATAGAAACAACTGTAG|  |  |  |  |  |  |  |  |  |  |  |  |  |  |  |  |  |  |  |  |  |  |  |  |  |  |  |  |  |  |  |  | | --- | --- | --- | --- | --- | --- | --- | --- | --- | --- | --- | --- | --- | --- | --- | --- | --- | --- | --- | --- | --- | --- | --- | --- | --- | --- | --- | --- | --- | --- | --- | --- | | Lemur micMur1 scaffold\_5139 40350 40463 - **GG** | GTGTAGTCCCGGGAAGCACCACCCTGCACAGTGCTGAGGTTCTGGCTGAGATGGCCCGTATCCTTCGACCTGGTGGATGTCTATTTCTGAAGGAGCCAGTAGAGACAGCCCTAG|  |  |  |  |  |  |  |  |  |  |  |  |  |  |  |  |  |  |  |  |  |  |  |  | | --- | --- | --- | --- | --- | --- | --- | --- | --- | --- | --- | --- | --- | --- | --- | --- | --- | --- | --- | --- | --- | --- | --- | --- | | Mouse mm9 chr8 97355673 97355786 - **GG** | GTGTAGTCCCAGGAAGCACCTCTCTGCACAGTGCTGAGGTTCTGGCTGAGATGGCCCGGATCCTCCGGCCAGGGGGCTGTCTTTTTCTGAAAGAACCAGTGGAGACAGCTGAAG|  |  |  |  |  |  |  |  |  |  |  |  |  |  |  |  | | --- | --- | --- | --- | --- | --- | --- | --- | --- | --- | --- | --- | --- | --- | --- | --- | | Cow bosTau4 chr18 25206840 25206953 - **GG** | GTATAATTCCTGGAAGCACCACTCTGCACAGTGCTGATATTTTGGCTGAGATGGCCCGGATTCTTCGGCCTGGTGGATGTCTTTTTCTGAAGGAACCGGTAGAGACAGCTGTAG|  |  |  |  |  |  |  |  | | --- | --- | --- | --- | --- | --- | --- | --- | | Dog canFam2 chr2 61863442 61863555 + **GG** | GTGTAATTCCTGGAAGCACCACTCTGCACAGCACTGAGATTTTGGCTGAGATGGCCCGGATCCTGCGGCCTGGTGGATGTCTTTTTCTGAGAGAGCCAGTAGAGACAGCTGTAG | | | | | | | | | | | | | | | | | | | | | | | | | | | | | | | | | | | | | | | | | | | | | | | | | | | | | | | | | | | | | | | | | | | | | | | | | | | | | | | | | | | | | | | | | | | |

**Alignment** (splice site sequences are in lowercase)  

```
Human      agGTTTAGTCCCAGGAAGCACCACTCTGCACAGTGCTGAGATTTTGGCTGAAATCGCCCGGATCCTTCGGCCTGGTGGAT
Chimp      g...............................................................................
Gorilla    g...............................................................................
Orangutan  g...........G...................................................................
Rhesus     g...........GC.C................................................................
Baboon     g...........G...................................................................
Marmoset   g...........G.............................................A..........A..........
Tarsier    g...A.CA....G...........................G..........G..G.........................
Lemur      g...G.......G...........C...............G..C.......G..G.....T........A..........
Mouse      g...G.................T.................G..C.......G..G...........C.....A..G..C.
Cow        g...A..A.T..T..........................T...........G..G........T................
Dog        g...G..A.T..T....................CA................G..G...........G.............

Human      GTCTTTTTCTGAAGGAGCCAGTAGAGACAGCTGTAGgt
Chimp      .............A........................
Gorilla    .............A........................
Orangutan  .............A........................
Rhesus     .............A.....G..................
Baboon     .............A.....G..................
Marmoset   .............A........................
Tarsier    .....A.......A......A....A...A........
Lemur      ....A..........................CC.....
Mouse      .............A..A.....G..........A....
Cow        ................A..G..................
Dog        ............GA........................
```

---

## 34. uc010wbi.1\_2\_8

**Summary**  

|  |  |  |  |  |  |  |  |  |  |  |  |  |  |  |  |  |  |  |  |  |  |  |  |  |  |
| --- | --- | --- | --- | --- | --- | --- | --- | --- | --- | --- | --- | --- | --- | --- | --- | --- | --- | --- | --- | --- | --- | --- | --- | --- | --- |
| No Exon ID Position (hg19) Dir Human acceptor Chimp acceptor Category Usage Gene symbol Protein accession mRNA accession Gene title Note|  |  |  |  |  |  |  |  |  |  |  |  |  | | --- | --- | --- | --- | --- | --- | --- | --- | --- | --- | --- | --- | --- | | 34 uc010wbi.1\_2\_8 chr17:28323324 + AG AT (A1) shift; increase; inframe alternative EFCAB5 B5MEA3 AK302745.1 EF-hand calcium binding domain 5  | | | | | | | | | | | | | | | | | | | | | | | | | |

**Orthologs**  

|  |  |  |  |  |  |  |  |  |  |  |  |  |  |  |  |  |  |  |  |  |  |  |  |  |  |  |  |  |  |  |  |  |  |  |  |  |  |  |  |  |  |  |  |  |  |  |  |  |  |  |  |  |  |  |  |  |  |  |  |  |  |  |  |  |  |  |  |  |  |  |  |
| --- | --- | --- | --- | --- | --- | --- | --- | --- | --- | --- | --- | --- | --- | --- | --- | --- | --- | --- | --- | --- | --- | --- | --- | --- | --- | --- | --- | --- | --- | --- | --- | --- | --- | --- | --- | --- | --- | --- | --- | --- | --- | --- | --- | --- | --- | --- | --- | --- | --- | --- | --- | --- | --- | --- | --- | --- | --- | --- | --- | --- | --- | --- | --- | --- | --- | --- | --- | --- | --- | --- | --- |
| Species Assembly Chromosome Exon start Exon end Dir Acceptor Exon sequence|  |  |  |  |  |  |  |  |  |  |  |  |  |  |  |  |  |  |  |  |  |  |  |  |  |  |  |  |  |  |  |  |  |  |  |  |  |  |  |  |  |  |  |  |  |  |  |  |  |  |  |  |  |  |  |  |  |  |  |  |  |  |  |  | | --- | --- | --- | --- | --- | --- | --- | --- | --- | --- | --- | --- | --- | --- | --- | --- | --- | --- | --- | --- | --- | --- | --- | --- | --- | --- | --- | --- | --- | --- | --- | --- | --- | --- | --- | --- | --- | --- | --- | --- | --- | --- | --- | --- | --- | --- | --- | --- | --- | --- | --- | --- | --- | --- | --- | --- | --- | --- | --- | --- | --- | --- | --- | --- | | Human hg19 chr17 28323324 28323378 + **AG** | TTGCAGATTCAGAATGTTCTTCAAGAATTCTTTCAAAATCCAGATTTCAAGCTTG|  |  |  |  |  |  |  |  |  |  |  |  |  |  |  |  |  |  |  |  |  |  |  |  |  |  |  |  |  |  |  |  |  |  |  |  |  |  |  |  |  |  |  |  |  |  |  |  |  |  |  |  |  |  |  |  | | --- | --- | --- | --- | --- | --- | --- | --- | --- | --- | --- | --- | --- | --- | --- | --- | --- | --- | --- | --- | --- | --- | --- | --- | --- | --- | --- | --- | --- | --- | --- | --- | --- | --- | --- | --- | --- | --- | --- | --- | --- | --- | --- | --- | --- | --- | --- | --- | --- | --- | --- | --- | --- | --- | --- | --- | | Chimp panTro2 chr17 27309929 27309983 - **AT** | TTGCAGATTCAGAATGTTCTTCATGAATTCTTTCAAAATCCAGATTTCAAGCTTG|  |  |  |  |  |  |  |  |  |  |  |  |  |  |  |  |  |  |  |  |  |  |  |  |  |  |  |  |  |  |  |  |  |  |  |  |  |  |  |  |  |  |  |  |  |  |  |  | | --- | --- | --- | --- | --- | --- | --- | --- | --- | --- | --- | --- | --- | --- | --- | --- | --- | --- | --- | --- | --- | --- | --- | --- | --- | --- | --- | --- | --- | --- | --- | --- | --- | --- | --- | --- | --- | --- | --- | --- | --- | --- | --- | --- | --- | --- | --- | --- | | Orangutan ponAbe2 chr17 24767262 24767316 + **AT** | TTGCAGATTCAGAATGTTCTTCATGAATTCTTTCAAAATCCAGATTTCAAGCTTG|  |  |  |  |  |  |  |  |  |  |  |  |  |  |  |  |  |  |  |  |  |  |  |  |  |  |  |  |  |  |  |  |  |  |  |  |  |  |  |  | | --- | --- | --- | --- | --- | --- | --- | --- | --- | --- | --- | --- | --- | --- | --- | --- | --- | --- | --- | --- | --- | --- | --- | --- | --- | --- | --- | --- | --- | --- | --- | --- | --- | --- | --- | --- | --- | --- | --- | --- | | Rhesus rheMac2 chr16 25234029 25234083 + **AT** | TTGCAGATTCAGAATGTTCTTCATGAATTCTTTCAAAATCCAGATTTCAAGCTAG|  |  |  |  |  |  |  |  |  |  |  |  |  |  |  |  |  |  |  |  |  |  |  |  |  |  |  |  |  |  |  |  | | --- | --- | --- | --- | --- | --- | --- | --- | --- | --- | --- | --- | --- | --- | --- | --- | --- | --- | --- | --- | --- | --- | --- | --- | --- | --- | --- | --- | --- | --- | --- | --- | | Galago otoGar1 scaffold\_16576.1-11573 3984 4038 + **AT** | TTGCAGATTCAAAATGTTCTTCATGAATTTTTTCAAAATACAGATTTTGATCTTG|  |  |  |  |  |  |  |  |  |  |  |  |  |  |  |  |  |  |  |  |  |  |  |  | | --- | --- | --- | --- | --- | --- | --- | --- | --- | --- | --- | --- | --- | --- | --- | --- | --- | --- | --- | --- | --- | --- | --- | --- | | Mouse mm9 chr11 76982393 76982447 - **CT** | CTGCAGATTCAGAATGTCCTTTATGATTTTTTTCAGAAGCCAGAGCTGCATCTTG|  |  |  |  |  |  |  |  |  |  |  |  |  |  |  |  | | --- | --- | --- | --- | --- | --- | --- | --- | --- | --- | --- | --- | --- | --- | --- | --- | | Cow bosTau4 chr19 21171133 21171187 + **AT** | TTGCAGATTCAGAACGTTCTTTATGAATTTTTTCAAAATCCAGATTTGCAGCTTG|  |  |  |  |  |  |  |  | | --- | --- | --- | --- | --- | --- | --- | --- | | Dog canFam2 chr9 47367685 47367739 + **AT** | TTGCAGATTCAGAATGTTCTTCATGAATTTTTTCAAAATCCAGATTTACAGCTTG | | | | | | | | | | | | | | | | | | | | | | | | | | | | | | | | | | | | | | | | | | | | | | | | | | | | | | | | | | | | | | | |

**Alignment** (splice site sequences are in lowercase)  

```
Human      agTTGCAGATTCAGAATGTTCTTCAAGAATTCTTTCAAAATCCAGATTTCAAGCTTGgt
Chimp      .t.......................T.................................
Orangutan  .t.......................T.................................
Rhesus     .t.......................T.............................A...
Galago     .t...........A...........T.....T.........A.......TG.T......
Mouse      ctC................C...T.T..T..T.....G..G.....GC.GC.T......
Cow        .t..............C......T.T.....T.................GC........
Dog        .t.......................T.....T.................AC........
```

---

## 35. uc010whf.1\_3\_5

**Summary**  

|  |  |  |  |  |  |  |  |  |  |  |  |  |  |  |  |  |  |  |  |  |  |  |  |  |  |
| --- | --- | --- | --- | --- | --- | --- | --- | --- | --- | --- | --- | --- | --- | --- | --- | --- | --- | --- | --- | --- | --- | --- | --- | --- | --- |
| No Exon ID Position (hg19) Dir Human acceptor Chimp acceptor Category Usage Gene symbol Protein accession mRNA accession Gene title Note|  |  |  |  |  |  |  |  |  |  |  |  |  | | --- | --- | --- | --- | --- | --- | --- | --- | --- | --- | --- | --- | --- | | 35 uc010whf.1\_3\_5 chr17:41059617 + AG GG (A3) shift; decrease; inframe alternative G6PC B4E1C3 AK303771.1 glucose-6-phosphatase  | | | | | | | | | | | | | | | | | | | | | | | | | |

**Orthologs**  

|  |  |  |  |  |  |  |  |  |  |  |  |  |  |  |  |  |  |  |  |  |  |  |  |  |  |  |  |  |  |  |  |  |  |  |  |  |  |  |  |  |  |  |  |  |  |  |  |  |  |  |  |  |  |  |  |  |  |  |  |  |  |  |  |  |  |  |  |  |  |  |  |  |  |  |  |  |  |  |  |  |  |  |  |  |  |  |  |  |  |  |  |  |  |  |  |
| --- | --- | --- | --- | --- | --- | --- | --- | --- | --- | --- | --- | --- | --- | --- | --- | --- | --- | --- | --- | --- | --- | --- | --- | --- | --- | --- | --- | --- | --- | --- | --- | --- | --- | --- | --- | --- | --- | --- | --- | --- | --- | --- | --- | --- | --- | --- | --- | --- | --- | --- | --- | --- | --- | --- | --- | --- | --- | --- | --- | --- | --- | --- | --- | --- | --- | --- | --- | --- | --- | --- | --- | --- | --- | --- | --- | --- | --- | --- | --- | --- | --- | --- | --- | --- | --- | --- | --- | --- | --- | --- | --- | --- | --- | --- | --- |
| Species Assembly Chromosome Exon start Exon end Dir Acceptor Exon sequence|  |  |  |  |  |  |  |  |  |  |  |  |  |  |  |  |  |  |  |  |  |  |  |  |  |  |  |  |  |  |  |  |  |  |  |  |  |  |  |  |  |  |  |  |  |  |  |  |  |  |  |  |  |  |  |  |  |  |  |  |  |  |  |  |  |  |  |  |  |  |  |  |  |  |  |  |  |  |  |  |  |  |  |  |  |  |  |  | | --- | --- | --- | --- | --- | --- | --- | --- | --- | --- | --- | --- | --- | --- | --- | --- | --- | --- | --- | --- | --- | --- | --- | --- | --- | --- | --- | --- | --- | --- | --- | --- | --- | --- | --- | --- | --- | --- | --- | --- | --- | --- | --- | --- | --- | --- | --- | --- | --- | --- | --- | --- | --- | --- | --- | --- | --- | --- | --- | --- | --- | --- | --- | --- | --- | --- | --- | --- | --- | --- | --- | --- | --- | --- | --- | --- | --- | --- | --- | --- | --- | --- | --- | --- | --- | --- | --- | --- | | Human hg19 chr17 41059617 41059645 + **AG** | GGAAAGATAAAGCCGACCTACAGATTTCG|  |  |  |  |  |  |  |  |  |  |  |  |  |  |  |  |  |  |  |  |  |  |  |  |  |  |  |  |  |  |  |  |  |  |  |  |  |  |  |  |  |  |  |  |  |  |  |  |  |  |  |  |  |  |  |  |  |  |  |  |  |  |  |  |  |  |  |  |  |  |  |  |  |  |  |  |  |  |  |  | | --- | --- | --- | --- | --- | --- | --- | --- | --- | --- | --- | --- | --- | --- | --- | --- | --- | --- | --- | --- | --- | --- | --- | --- | --- | --- | --- | --- | --- | --- | --- | --- | --- | --- | --- | --- | --- | --- | --- | --- | --- | --- | --- | --- | --- | --- | --- | --- | --- | --- | --- | --- | --- | --- | --- | --- | --- | --- | --- | --- | --- | --- | --- | --- | --- | --- | --- | --- | --- | --- | --- | --- | --- | --- | --- | --- | --- | --- | --- | --- | | Chimp panTro2 chr17 14614196 14614224 - **GG** | GGAAAGATAAAGCCGACCTACAGATTTCG|  |  |  |  |  |  |  |  |  |  |  |  |  |  |  |  |  |  |  |  |  |  |  |  |  |  |  |  |  |  |  |  |  |  |  |  |  |  |  |  |  |  |  |  |  |  |  |  |  |  |  |  |  |  |  |  |  |  |  |  |  |  |  |  |  |  |  |  |  |  |  |  | | --- | --- | --- | --- | --- | --- | --- | --- | --- | --- | --- | --- | --- | --- | --- | --- | --- | --- | --- | --- | --- | --- | --- | --- | --- | --- | --- | --- | --- | --- | --- | --- | --- | --- | --- | --- | --- | --- | --- | --- | --- | --- | --- | --- | --- | --- | --- | --- | --- | --- | --- | --- | --- | --- | --- | --- | --- | --- | --- | --- | --- | --- | --- | --- | --- | --- | --- | --- | --- | --- | --- | --- | | Orangutan ponAbe2 chr17 46511338 46511366 - **GG** | GGAAAGAAAAAGCCGACCTACAGATTTCG|  |  |  |  |  |  |  |  |  |  |  |  |  |  |  |  |  |  |  |  |  |  |  |  |  |  |  |  |  |  |  |  |  |  |  |  |  |  |  |  |  |  |  |  |  |  |  |  |  |  |  |  |  |  |  |  |  |  |  |  |  |  |  |  | | --- | --- | --- | --- | --- | --- | --- | --- | --- | --- | --- | --- | --- | --- | --- | --- | --- | --- | --- | --- | --- | --- | --- | --- | --- | --- | --- | --- | --- | --- | --- | --- | --- | --- | --- | --- | --- | --- | --- | --- | --- | --- | --- | --- | --- | --- | --- | --- | --- | --- | --- | --- | --- | --- | --- | --- | --- | --- | --- | --- | --- | --- | --- | --- | | Rhesus rheMac2 chr16 53028841 53028869 + **GG** | GGAAAGAAAAAGCCGACCTACAGATTTCG|  |  |  |  |  |  |  |  |  |  |  |  |  |  |  |  |  |  |  |  |  |  |  |  |  |  |  |  |  |  |  |  |  |  |  |  |  |  |  |  |  |  |  |  |  |  |  |  |  |  |  |  |  |  |  |  | | --- | --- | --- | --- | --- | --- | --- | --- | --- | --- | --- | --- | --- | --- | --- | --- | --- | --- | --- | --- | --- | --- | --- | --- | --- | --- | --- | --- | --- | --- | --- | --- | --- | --- | --- | --- | --- | --- | --- | --- | --- | --- | --- | --- | --- | --- | --- | --- | --- | --- | --- | --- | --- | --- | --- | --- | | Baboon papHam1 scaffold7395 101787 101815 - **GG** | GGAAAGAAAAAGCCGACCTACGGATTTCG|  |  |  |  |  |  |  |  |  |  |  |  |  |  |  |  |  |  |  |  |  |  |  |  |  |  |  |  |  |  |  |  |  |  |  |  |  |  |  |  |  |  |  |  |  |  |  |  | | --- | --- | --- | --- | --- | --- | --- | --- | --- | --- | --- | --- | --- | --- | --- | --- | --- | --- | --- | --- | --- | --- | --- | --- | --- | --- | --- | --- | --- | --- | --- | --- | --- | --- | --- | --- | --- | --- | --- | --- | --- | --- | --- | --- | --- | --- | --- | --- | | Marmoset calJac1 Contig1855 280021 280049 - **GG** | GGAAAGAAAAAGCCAACCTACAGATTTCA|  |  |  |  |  |  |  |  |  |  |  |  |  |  |  |  |  |  |  |  |  |  |  |  |  |  |  |  |  |  |  |  |  |  |  |  |  |  |  |  | | --- | --- | --- | --- | --- | --- | --- | --- | --- | --- | --- | --- | --- | --- | --- | --- | --- | --- | --- | --- | --- | --- | --- | --- | --- | --- | --- | --- | --- | --- | --- | --- | --- | --- | --- | --- | --- | --- | --- | --- | | Lemur micMur1 scaffold\_2280 52719 52747 + **GG** | GGAAAGAAAAAGCCGACTTACAGATTTCG|  |  |  |  |  |  |  |  |  |  |  |  |  |  |  |  |  |  |  |  |  |  |  |  |  |  |  |  |  |  |  |  | | --- | --- | --- | --- | --- | --- | --- | --- | --- | --- | --- | --- | --- | --- | --- | --- | --- | --- | --- | --- | --- | --- | --- | --- | --- | --- | --- | --- | --- | --- | --- | --- | | Galago otoGar1 scaffold\_116411.1-118332 55054 55082 - **GG** | GAAAAGAAAAGGACGACCTACAGATTTCG|  |  |  |  |  |  |  |  |  |  |  |  |  |  |  |  |  |  |  |  |  |  |  |  | | --- | --- | --- | --- | --- | --- | --- | --- | --- | --- | --- | --- | --- | --- | --- | --- | --- | --- | --- | --- | --- | --- | --- | --- | | Mouse mm9 chr11 101234093 101234121 + **GA** | GGAAAGAAAAAGCCAACGTATGGATTCCG|  |  |  |  |  |  |  |  |  |  |  |  |  |  |  |  | | --- | --- | --- | --- | --- | --- | --- | --- | --- | --- | --- | --- | --- | --- | --- | --- | | Cow bosTau4 chr19 44364219 44364247 + **GT** | GGAAAGAAAAAGCCAACCTACAGATTTCG|  |  |  |  |  |  |  |  | | --- | --- | --- | --- | --- | --- | --- | --- | | Dog canFam2 chr9 23456543 23456571 - **GG** | GGGAGAAAAAGGCCAACCTACAGATTTCG | | | | | | | | | | | | | | | | | | | | | | | | | | | | | | | | | | | | | | | | | | | | | | | | | | | | | | | | | | | | | | | | | | | | | | | | | | | | | | | | | | | | |

**Alignment** (splice site sequences are in lowercase)  

```
Human      agGGAAAGATAAAGCCGACCTACAGATTTCGgt
Chimp      g................................
Orangutan  g........A.......................
Rhesus     g........A.......................
Baboon     g........A.............G.........
Marmoset   g........A......A.............A..
Lemur      g........A.........T.............
Galago     g..A.....A..G.A..................
Mouse      ga.......A......A..G..TG....C....
Cow        gt.......A......A................
Dog        g...G.GA.A..G...A................
```

---

## 36. uc010dcc.1\_2\_8

**Summary**  

|  |  |  |  |  |  |  |  |  |  |  |  |  |  |  |  |  |  |  |  |  |  |  |  |  |  |
| --- | --- | --- | --- | --- | --- | --- | --- | --- | --- | --- | --- | --- | --- | --- | --- | --- | --- | --- | --- | --- | --- | --- | --- | --- | --- |
| No Exon ID Position (hg19) Dir Human acceptor Chimp acceptor Category Usage Gene symbol Protein accession mRNA accession Gene title Note|  |  |  |  |  |  |  |  |  |  |  |  |  | | --- | --- | --- | --- | --- | --- | --- | --- | --- | --- | --- | --- | --- | | 36 uc010dcc.1\_2\_8 chr17:53076987 + AG AA (A1) shift; increase; inframe alternative STXBP4 AAH41485.1 BC041485.2 syntaxin-binding protein 4 dbSNP:rs11658717 | | | | | | | | | | | | | | | | | | | | | | | | | |

**Orthologs**  

|  |  |  |  |  |  |  |  |  |  |  |  |  |  |  |  |  |  |  |  |  |  |  |  |  |  |  |  |  |  |  |  |  |  |  |  |  |  |  |  |  |  |  |  |  |  |  |  |  |  |  |  |  |  |  |  |  |  |  |  |  |  |  |  |  |  |  |  |  |  |  |  |  |  |  |  |  |  |  |  |  |  |  |  |  |  |  |  |  |  |  |  |  |  |  |  |  |  |  |  |  |  |  |  |  |  |  |  |  |  |  |  |
| --- | --- | --- | --- | --- | --- | --- | --- | --- | --- | --- | --- | --- | --- | --- | --- | --- | --- | --- | --- | --- | --- | --- | --- | --- | --- | --- | --- | --- | --- | --- | --- | --- | --- | --- | --- | --- | --- | --- | --- | --- | --- | --- | --- | --- | --- | --- | --- | --- | --- | --- | --- | --- | --- | --- | --- | --- | --- | --- | --- | --- | --- | --- | --- | --- | --- | --- | --- | --- | --- | --- | --- | --- | --- | --- | --- | --- | --- | --- | --- | --- | --- | --- | --- | --- | --- | --- | --- | --- | --- | --- | --- | --- | --- | --- | --- | --- | --- | --- | --- | --- | --- | --- | --- | --- | --- | --- | --- | --- | --- | --- | --- |
| Species Assembly Chromosome Exon start Exon end Dir Acceptor Exon sequence|  |  |  |  |  |  |  |  |  |  |  |  |  |  |  |  |  |  |  |  |  |  |  |  |  |  |  |  |  |  |  |  |  |  |  |  |  |  |  |  |  |  |  |  |  |  |  |  |  |  |  |  |  |  |  |  |  |  |  |  |  |  |  |  |  |  |  |  |  |  |  |  |  |  |  |  |  |  |  |  |  |  |  |  |  |  |  |  |  |  |  |  |  |  |  |  |  |  |  |  |  |  |  |  | | --- | --- | --- | --- | --- | --- | --- | --- | --- | --- | --- | --- | --- | --- | --- | --- | --- | --- | --- | --- | --- | --- | --- | --- | --- | --- | --- | --- | --- | --- | --- | --- | --- | --- | --- | --- | --- | --- | --- | --- | --- | --- | --- | --- | --- | --- | --- | --- | --- | --- | --- | --- | --- | --- | --- | --- | --- | --- | --- | --- | --- | --- | --- | --- | --- | --- | --- | --- | --- | --- | --- | --- | --- | --- | --- | --- | --- | --- | --- | --- | --- | --- | --- | --- | --- | --- | --- | --- | --- | --- | --- | --- | --- | --- | --- | --- | --- | --- | --- | --- | --- | --- | --- | --- | | Human hg19 chr17 53076987 53077203 + **AG** | TACTAGGTTAGAATCTGCTTGGGAGATAGCATTCATAAGACAAAAATCCGACAACATTCAGCCAGAAAATCTGTCATGTACATCACTTATAGAAGCTTCAGGAGAATATGGACCTCAAGCCTCAACATTAAGTCTTTTTTCTTCTCCTCCTGAAATACTAATCCCAAAGACCTCATCCACTCCCAAAACAAATAATGACATTTTATCTTCTTGTGAG|  |  |  |  |  |  |  |  |  |  |  |  |  |  |  |  |  |  |  |  |  |  |  |  |  |  |  |  |  |  |  |  |  |  |  |  |  |  |  |  |  |  |  |  |  |  |  |  |  |  |  |  |  |  |  |  |  |  |  |  |  |  |  |  |  |  |  |  |  |  |  |  |  |  |  |  |  |  |  |  |  |  |  |  |  |  |  |  |  |  |  |  |  |  |  |  | | --- | --- | --- | --- | --- | --- | --- | --- | --- | --- | --- | --- | --- | --- | --- | --- | --- | --- | --- | --- | --- | --- | --- | --- | --- | --- | --- | --- | --- | --- | --- | --- | --- | --- | --- | --- | --- | --- | --- | --- | --- | --- | --- | --- | --- | --- | --- | --- | --- | --- | --- | --- | --- | --- | --- | --- | --- | --- | --- | --- | --- | --- | --- | --- | --- | --- | --- | --- | --- | --- | --- | --- | --- | --- | --- | --- | --- | --- | --- | --- | --- | --- | --- | --- | --- | --- | --- | --- | --- | --- | --- | --- | --- | --- | --- | --- | | Chimp panTro2 chr17 54105378 54105594 + **AA** | TACTAGGTTAGAATCTGCTTGGGAGATAGCATTCATAAGACAAAAATCCGACAACATTCAGCTAGAAAATCTGTCATGTTCATCACTTATAGAAGCTTCAGGAGAATATGGACCTCAAGCCTCAACATTAAGTCTTTTTTCTTCTCCTCCTGAAATACTAATCCCAAAGACCTCATCCACTCCCAAAACAAATAGTGCCATTTTATCTTCTTGTGAG|  |  |  |  |  |  |  |  |  |  |  |  |  |  |  |  |  |  |  |  |  |  |  |  |  |  |  |  |  |  |  |  |  |  |  |  |  |  |  |  |  |  |  |  |  |  |  |  |  |  |  |  |  |  |  |  |  |  |  |  |  |  |  |  |  |  |  |  |  |  |  |  |  |  |  |  |  |  |  |  |  |  |  |  |  |  |  |  | | --- | --- | --- | --- | --- | --- | --- | --- | --- | --- | --- | --- | --- | --- | --- | --- | --- | --- | --- | --- | --- | --- | --- | --- | --- | --- | --- | --- | --- | --- | --- | --- | --- | --- | --- | --- | --- | --- | --- | --- | --- | --- | --- | --- | --- | --- | --- | --- | --- | --- | --- | --- | --- | --- | --- | --- | --- | --- | --- | --- | --- | --- | --- | --- | --- | --- | --- | --- | --- | --- | --- | --- | --- | --- | --- | --- | --- | --- | --- | --- | --- | --- | --- | --- | --- | --- | --- | --- | | Gorilla gorGor1 Supercontig\_0161187 686 902 + **AA** | TACTAGGTTAGAATCTGCTTGGGAGATAGCATTCATAAGACAAAAATCTGACNACATTCAGCCAGAAAATCTGTCATGTACATCACTTATAGAAGCTTCAGGAGAATATGGACCTCAAGCCTCAACATTAAGTCTTTTTTCTTNTCCTCCTGAANTACTAATCCCAAAGACCTCATCCACTCCCAAAACAAATAANGCCATTTTATCTTCTTGTGAG|  |  |  |  |  |  |  |  |  |  |  |  |  |  |  |  |  |  |  |  |  |  |  |  |  |  |  |  |  |  |  |  |  |  |  |  |  |  |  |  |  |  |  |  |  |  |  |  |  |  |  |  |  |  |  |  |  |  |  |  |  |  |  |  |  |  |  |  |  |  |  |  |  |  |  |  |  |  |  |  | | --- | --- | --- | --- | --- | --- | --- | --- | --- | --- | --- | --- | --- | --- | --- | --- | --- | --- | --- | --- | --- | --- | --- | --- | --- | --- | --- | --- | --- | --- | --- | --- | --- | --- | --- | --- | --- | --- | --- | --- | --- | --- | --- | --- | --- | --- | --- | --- | --- | --- | --- | --- | --- | --- | --- | --- | --- | --- | --- | --- | --- | --- | --- | --- | --- | --- | --- | --- | --- | --- | --- | --- | --- | --- | --- | --- | --- | --- | --- | --- | | Orangutan ponAbe2 chr17 37265144 37265360 - **AA** | CCCTAGGTTAGGATCTGCTTGGGAGATAGCATTCATAAGACAAAAATCCAACAACATTCAGCCAGAAAATCTGTCATGTACATCACTTATAGAAGCTTCAGGAGAATATGGACCTCAAGCCTCAACATTAAGTCTTTTTTCTTCTCCTCCTGAAATACTAATCCCAAAGACCTCATCCACTCCCAAAACAAATAATGCCATTTTACCTTCTTGTGAG|  |  |  |  |  |  |  |  |  |  |  |  |  |  |  |  |  |  |  |  |  |  |  |  |  |  |  |  |  |  |  |  |  |  |  |  |  |  |  |  |  |  |  |  |  |  |  |  |  |  |  |  |  |  |  |  |  |  |  |  |  |  |  |  |  |  |  |  |  |  |  |  | | --- | --- | --- | --- | --- | --- | --- | --- | --- | --- | --- | --- | --- | --- | --- | --- | --- | --- | --- | --- | --- | --- | --- | --- | --- | --- | --- | --- | --- | --- | --- | --- | --- | --- | --- | --- | --- | --- | --- | --- | --- | --- | --- | --- | --- | --- | --- | --- | --- | --- | --- | --- | --- | --- | --- | --- | --- | --- | --- | --- | --- | --- | --- | --- | --- | --- | --- | --- | --- | --- | --- | --- | | Rhesus rheMac2 chr16 39258071 39258287 + **AA** | TACTAGGTCAGAATCTGCTTGGGAGATAGCATTCATCAGACAAAAATCCGACAACATTCAGCCAGAAAATCTGTCATGTACATCACTTGTAGAAGCTTCAGGAGAATATGGACCTCAAGCCTCAACATTTAGTTTTTTTTCTTCTCCTCCTGAAATACTAATCCCAAAGACCTCATCCACTCCCAAAACAAATAATGCCATTTTACCTTCTTGTGAG|  |  |  |  |  |  |  |  |  |  |  |  |  |  |  |  |  |  |  |  |  |  |  |  |  |  |  |  |  |  |  |  |  |  |  |  |  |  |  |  |  |  |  |  |  |  |  |  |  |  |  |  |  |  |  |  |  |  |  |  |  |  |  |  | | --- | --- | --- | --- | --- | --- | --- | --- | --- | --- | --- | --- | --- | --- | --- | --- | --- | --- | --- | --- | --- | --- | --- | --- | --- | --- | --- | --- | --- | --- | --- | --- | --- | --- | --- | --- | --- | --- | --- | --- | --- | --- | --- | --- | --- | --- | --- | --- | --- | --- | --- | --- | --- | --- | --- | --- | --- | --- | --- | --- | --- | --- | --- | --- | | Baboon papHam1 scaffold10534 10888 11104 - **AA** | TACTAGGTCAGAATCTGCTTGGGAGATAGCATTCATCAGACAAAAATCCAACAACATTCAGCCAGAAAATCTGTCATGTACATCACTTGTAGAAGCTTCAGGAGAATATGGACCTCAAGCCTCAACATTTAGTTTTTTTTCTTCTCCTCCTGAAATACTAATCCCAAAGACCTCATCCACTCCCAAAACAAATAATGCCATTTTACCTTCTTGTGAG|  |  |  |  |  |  |  |  |  |  |  |  |  |  |  |  |  |  |  |  |  |  |  |  |  |  |  |  |  |  |  |  |  |  |  |  |  |  |  |  |  |  |  |  |  |  |  |  |  |  |  |  |  |  |  |  | | --- | --- | --- | --- | --- | --- | --- | --- | --- | --- | --- | --- | --- | --- | --- | --- | --- | --- | --- | --- | --- | --- | --- | --- | --- | --- | --- | --- | --- | --- | --- | --- | --- | --- | --- | --- | --- | --- | --- | --- | --- | --- | --- | --- | --- | --- | --- | --- | --- | --- | --- | --- | --- | --- | --- | --- | | Marmoset calJac1 Contig263 177199 177415 + **AA** | TATTAGGTCAGAATCTGCTTGGGAGATAGCATTCATAAGACAAAAATCCGACAACAGTCAACCAGAAAATCTGTCATGTACATCACTTGTAGAAGCTTCAGGAGAATATATACCTCAAGCCTCAGCATTTAGTCTTTTTTCTTCTCCTCCTGAAACACTAATTCCAAAGACCTCATCCACTCCCAAAACTAACAATGCCATTTTACCTTCTTGTGAG|  |  |  |  |  |  |  |  |  |  |  |  |  |  |  |  |  |  |  |  |  |  |  |  |  |  |  |  |  |  |  |  |  |  |  |  |  |  |  |  |  |  |  |  |  |  |  |  | | --- | --- | --- | --- | --- | --- | --- | --- | --- | --- | --- | --- | --- | --- | --- | --- | --- | --- | --- | --- | --- | --- | --- | --- | --- | --- | --- | --- | --- | --- | --- | --- | --- | --- | --- | --- | --- | --- | --- | --- | --- | --- | --- | --- | --- | --- | --- | --- | | Tarsier tarSyr1 scaffold\_50645 5227 5442 + **AA** | TACTAGGTCAGAATCGGCTTGGGAGATAGGATTCATAAGACAAAAATGTGATGGCAGTCATCCAGAAAACCTATCGTGTACATCTCTTGTAGAATTCCACCACAATATGGACCTCAAGCCTCAACACTTAGTCTTGTTTCTTCTCCCTCTGAAATACTAATTCCAAAGACTTCATCCACTCCCAAAACTAACAATGCCATTTTACCTTCTTTTGAG|  |  |  |  |  |  |  |  |  |  |  |  |  |  |  |  |  |  |  |  |  |  |  |  |  |  |  |  |  |  |  |  |  |  |  |  |  |  |  |  | | --- | --- | --- | --- | --- | --- | --- | --- | --- | --- | --- | --- | --- | --- | --- | --- | --- | --- | --- | --- | --- | --- | --- | --- | --- | --- | --- | --- | --- | --- | --- | --- | --- | --- | --- | --- | --- | --- | --- | --- | | Lemur micMur1 scaffold\_2398 23154 23370 + **AA** | TACTAGGTCAGAATCTGCTTGGGAGATAGCATTCAGAAGACAAAAATCTGACAACAATCACCCAGAAAAGCTTTCGTGTACATCCCTTTTAGAAGCTTCAGGAGAATATGGACCTCAAGCCTCAACATTTAGTCTTCTTTCTTCTCCCCCTGAAATACTAATTCCAAAGACCTCATCTACTCCCAAAATTAATGATGCAGCTTTACCTTCTTATAAG|  |  |  |  |  |  |  |  |  |  |  |  |  |  |  |  |  |  |  |  |  |  |  |  |  |  |  |  |  |  |  |  | | --- | --- | --- | --- | --- | --- | --- | --- | --- | --- | --- | --- | --- | --- | --- | --- | --- | --- | --- | --- | --- | --- | --- | --- | --- | --- | --- | --- | --- | --- | --- | --- | | Galago otoGar1 scaffold\_116661.1-395716 36176 36389 + **AA** | TACTAGGTCAGAATCTACTTGGGAGATAGCATTCATAAGACAAAAATCTGATATCCACCATCCAGAAAAGCTATCGTGTGCATCTTTCGAAGCTTCAGAAGAATATGGACCTCAAGCCTCAACATTTAGTCTTCTTCCTTCTCCCCCTGAAATACTAATTCCAAAGACCTCATCCACTCCCAAAATTAATGACGCCATTTTACCTTCTTATGAG|  |  |  |  |  |  |  |  |  |  |  |  |  |  |  |  |  |  |  |  |  |  |  |  | | --- | --- | --- | --- | --- | --- | --- | --- | --- | --- | --- | --- | --- | --- | --- | --- | --- | --- | --- | --- | --- | --- | --- | --- | | Mouse mm9 chr11 90468291 90468504 - **CA** | TCGTAGGTCAGAATCTCCCTGGGAGATAGCATTCATCAGACAAAAGTCTTACTGTGGCCATCCAGGAAATATTTGCTGTCCATCCCCACAAGTGTCAGAAGACTGTGGACCTCAAACCTCAACATTTACTCTTCTTTCCTCTCCCTCTGAAACACTACTTCCAAAGACTTCATCCACTCCCCAGACTCAGGACTCCACTTTCCCTTCTTGTAAA|  |  |  |  |  |  |  |  |  |  |  |  |  |  |  |  | | --- | --- | --- | --- | --- | --- | --- | --- | --- | --- | --- | --- | --- | --- | --- | --- | | Cow bosTau4 chr19 4285765 4285978 + **AA** | TGCTAGGTCAGAATCTTCTTGGGAGATAGCATTCATAAGACAGAAATCTGACAGCAGCCATTTAGAAAATCCATCTTGTTCATCCCTTTTAGAAGCTACAGGAGAATATGGACCTCATGCCTCAAAATTTAGCCTTATTTCTTCTCCTGAAACACTAATTCCAAAGACCTCATCCACTCCCAGATCTACAGACGCCATTTTACCTTCTTTGAGG|  |  |  |  |  |  |  |  | | --- | --- | --- | --- | --- | --- | --- | --- | | Dog canFam2 chr9 33430051 33430267 + **AA** | TGCTAGGTCAGAATCTGCTTGGGAGATAGCATTCATAAGACAAAAATCTGATGGCAGTCATCTAGAAAATCCATCTTGTACATCCCTTTTACAAGCTTCCGGAGAATATGGACCTCAAGCCTCGACATTTAGCCTTCTTCCTTCTCCCCCTGAAATACTAATTCCAAAGACCTCATCCACTCCCCAAACTACAGATACCACTTTATCTTCTTTTAAC | | | | | | | | | | | | | | | | | | | | | | | | | | | | | | | | | | | | | | | | | | | | | | | | | | | | | | | | | | | | | | | | | | | | | | | | | | | | | | | | | | | | | | | | | | | | | | | | | | |

**Alignment** (splice site sequences are in lowercase)  

```
Human      agTACTAGGTTAGAATCTGCTTGGGAGATAGCATTCATAAGACAAAAATCCGACAACATTCAGCCAGAAAATCTGTCATG
Chimp      .a..............................................................T...............
Gorilla    .a................................................T...N.........................
Orangutan  .aCC.........G.....................................A............................
Rhesus     .a........C...........................C.........................................
Baboon     .a........C...........................C............A............................
Marmoset   .a..T.....C...............................................G...A.................
Tarsier    .a........C......G.............G.................GT..TGG..G...T........C..A..G..
Lemur      .a........C..........................G............T.......A...C........G..T..G..
Galago     .a........C.......A...............................T..T.T.CAC..T........G..A..G..
Mouse      ca.CG.....C.......C.C.................C........G..TT..TGTGGC..T....G....A.T.GC..
Cow        .a.G......C.......T.........................G.....T....G..GC..TTT........CA..T..
Dog        .a.G......C.......................................T..TGG..G...T.T........CA..T..

Human      TACATCACTTATAGAAGCTTCAGGAGAATATGGACCTCAAGCCTCAACATTAAGTCTTTTTTCTTCTCCTCCTGAAATAC
Chimp      .T..............................................................................
Gorilla    .................................................................N..........N...
Orangutan  ................................................................................
Rhesus     ..........G........................................T...T........................
Baboon     ..........G........................................T...T........................
Marmoset   ..........G....................AT.............G....T.........................C..
Tarsier    ......T...G.....-T.C..CC.C.......................C.T......G..........CT.........
Lemur      ......C...T........................................T......C..........C..........
Galago     .G....---.T.C..........A...........................T......C..C.......C..........
Mouse      .C....C.---C.C...TG....A...C.G..........A..........T.C....C....C.....CT......C..
Cow        .T....C...T........A...................T.......A...T..C...A.........---......C..
Dog        ......C...T..C.......C.......................G.....T..C...C..C.......C..........

Human      TAATCCCAAAGACCTCATCCACTCCCAAAACAAATAATGACATTTTATCTTCTTGTGAGgt
Chimp      ....................................G..C.....................
Gorilla    .....................................N.C.....................
Orangutan  .......................................C.......C.............
Rhesus     .......................................C.......C.............
Baboon     .......................................C.......C.............
Marmoset   ....T..........................T..C....C.......C.............
Tarsier    ....T........T.................T..C....C.......C......T....a.
Lemur      ....T..............T..........TT...G...CAGC....C......A.A..a.
Galago     ....T.........................TT...G.C.C.......C......A....a.
Mouse      ..C.T........T............C.G..TC.GG.CTC..C...CC........A.A.c
Cow        ....T......................G.T.T.CAG.C.C.......C......TGAG.ag
Dog        ....T.....................C....T.CAG..AC..C...........T.A.Ca.
```

---

## 37. uc002luw.1\_9\_10

**Summary**  

|  |  |  |  |  |  |  |  |  |  |  |  |  |  |  |  |  |  |  |  |  |  |  |  |  |  |
| --- | --- | --- | --- | --- | --- | --- | --- | --- | --- | --- | --- | --- | --- | --- | --- | --- | --- | --- | --- | --- | --- | --- | --- | --- | --- |
| No Exon ID Position (hg19) Dir Human acceptor Chimp acceptor Category Usage Gene symbol Protein accession mRNA accession Gene title Note|  |  |  |  |  |  |  |  |  |  |  |  |  | | --- | --- | --- | --- | --- | --- | --- | --- | --- | --- | --- | --- | --- | | 37 uc002luw.1\_9\_10 chr19:2098975 + AG AA (A5) exonization; inframe alternative IZUMO4 NP\_001026905.2 NM\_001039846.1 izumo sperm-egg fusion protein 4  | | | | | | | | | | | | | | | | | | | | | | | | | |

**Orthologs**  

|  |  |  |  |  |  |  |  |  |  |  |  |  |  |  |  |  |  |  |  |  |  |  |  |  |  |  |  |  |  |  |  |  |  |  |  |  |  |  |  |  |  |  |  |  |  |  |  |  |  |  |  |  |  |  |  |
| --- | --- | --- | --- | --- | --- | --- | --- | --- | --- | --- | --- | --- | --- | --- | --- | --- | --- | --- | --- | --- | --- | --- | --- | --- | --- | --- | --- | --- | --- | --- | --- | --- | --- | --- | --- | --- | --- | --- | --- | --- | --- | --- | --- | --- | --- | --- | --- | --- | --- | --- | --- | --- | --- | --- | --- |
| Species Assembly Chromosome Exon start Exon end Dir Acceptor Exon sequence|  |  |  |  |  |  |  |  |  |  |  |  |  |  |  |  |  |  |  |  |  |  |  |  |  |  |  |  |  |  |  |  |  |  |  |  |  |  |  |  |  |  |  |  |  |  |  |  | | --- | --- | --- | --- | --- | --- | --- | --- | --- | --- | --- | --- | --- | --- | --- | --- | --- | --- | --- | --- | --- | --- | --- | --- | --- | --- | --- | --- | --- | --- | --- | --- | --- | --- | --- | --- | --- | --- | --- | --- | --- | --- | --- | --- | --- | --- | --- | --- | | Human hg19 chr19 2098975 2099028 + **AG** | ACCACGCTCCTCTGCCTTCTCCTGGCCTGGGACACACAGAGCCACCCCGGCCTT|  |  |  |  |  |  |  |  |  |  |  |  |  |  |  |  |  |  |  |  |  |  |  |  |  |  |  |  |  |  |  |  |  |  |  |  |  |  |  |  | | --- | --- | --- | --- | --- | --- | --- | --- | --- | --- | --- | --- | --- | --- | --- | --- | --- | --- | --- | --- | --- | --- | --- | --- | --- | --- | --- | --- | --- | --- | --- | --- | --- | --- | --- | --- | --- | --- | --- | --- | | Chimp panTro2 chr19 2100056 2100109 + **AA** | ACCACGCTCCTCTGCCTTCTTCTGGCCTGGGACACACACAGCCACCCCAGCCTT|  |  |  |  |  |  |  |  |  |  |  |  |  |  |  |  |  |  |  |  |  |  |  |  |  |  |  |  |  |  |  |  | | --- | --- | --- | --- | --- | --- | --- | --- | --- | --- | --- | --- | --- | --- | --- | --- | --- | --- | --- | --- | --- | --- | --- | --- | --- | --- | --- | --- | --- | --- | --- | --- | | Gorilla gorGor1 Supercontig\_0000307 136912 136965 - **AA** | ACCACGCTCCTCTGCCTTCTCCTGGCCTGGGACACACAGAGCCACCCCAGCCTT|  |  |  |  |  |  |  |  |  |  |  |  |  |  |  |  |  |  |  |  |  |  |  |  | | --- | --- | --- | --- | --- | --- | --- | --- | --- | --- | --- | --- | --- | --- | --- | --- | --- | --- | --- | --- | --- | --- | --- | --- | | Orangutan ponAbe2 chr19 2075066 2075119 + **AA** | ACCACGCTCCTCCTCCTTCTCCTGGCCTGGGACACACAGAGCCACCCCAGCCTT|  |  |  |  |  |  |  |  |  |  |  |  |  |  |  |  | | --- | --- | --- | --- | --- | --- | --- | --- | --- | --- | --- | --- | --- | --- | --- | --- | | Rhesus rheMac2 chr19 1885174 1885227 + **AA** | ACTACGCTCCTCCTCCTTCTCCTGGCCTGGGACACACAGAGCCACCCCAGCCTT|  |  |  |  |  |  |  |  | | --- | --- | --- | --- | --- | --- | --- | --- | | Baboon papHam1 scaffold31837 8220 8273 - **AA** | ACTACGCTCCTCCTCCTTCTCCTGGCCTGGGACACACAGAGCCACCCCAGCCTT | | | | | | | | | | | | | | | | | | | | | | | | | | | | | | | | | | | | | | | | | | | | | | | | | |

**Alignment** (splice site sequences are in lowercase)  

```
Human      agACCACGCTCCTCTGCCTTCTCCTGGCCTGGGACACACAGAGCCACCCCGGCCTTgt
Chimp      .a....................T.................C.........A.......
Gorilla    .a................................................A.......
Orangutan  .a............CT..................................A.......
Rhesus     .a..T.........CT..................................A.......
Baboon     .a..T.........CT..................................A.......
```

---

## 38. uc002nhx.1\_2\_18

**Summary**  

|  |  |  |  |  |  |  |  |  |  |  |  |  |  |  |  |  |  |  |  |  |  |  |  |  |  |
| --- | --- | --- | --- | --- | --- | --- | --- | --- | --- | --- | --- | --- | --- | --- | --- | --- | --- | --- | --- | --- | --- | --- | --- | --- | --- |
| No Exon ID Position (hg19) Dir Human acceptor Chimp acceptor Category Usage Gene symbol Protein accession mRNA accession Gene title Note|  |  |  |  |  |  |  |  |  |  |  |  |  | | --- | --- | --- | --- | --- | --- | --- | --- | --- | --- | --- | --- | --- | | 38 uc002nhx.1\_2\_18 chr19:18197742 - AG GG (A7) novel start alternative IL12RB1 P42701 BX647221.1 interleukin 12 receptor, beta 1 dbSNP:rs393548 | | | | | | | | | | | | | | | | | | | | | | | | | |

**Orthologs**  

|  |  |  |  |  |  |  |  |  |  |  |  |  |  |  |  |  |  |  |  |  |  |  |  |  |  |  |  |  |  |  |  |  |  |  |  |  |  |  |  |  |  |  |  |  |  |  |  |  |  |  |  |  |  |  |  |
| --- | --- | --- | --- | --- | --- | --- | --- | --- | --- | --- | --- | --- | --- | --- | --- | --- | --- | --- | --- | --- | --- | --- | --- | --- | --- | --- | --- | --- | --- | --- | --- | --- | --- | --- | --- | --- | --- | --- | --- | --- | --- | --- | --- | --- | --- | --- | --- | --- | --- | --- | --- | --- | --- | --- | --- |
| Species Assembly Chromosome Exon start Exon end Dir Acceptor Exon sequence|  |  |  |  |  |  |  |  |  |  |  |  |  |  |  |  |  |  |  |  |  |  |  |  |  |  |  |  |  |  |  |  |  |  |  |  |  |  |  |  |  |  |  |  |  |  |  |  | | --- | --- | --- | --- | --- | --- | --- | --- | --- | --- | --- | --- | --- | --- | --- | --- | --- | --- | --- | --- | --- | --- | --- | --- | --- | --- | --- | --- | --- | --- | --- | --- | --- | --- | --- | --- | --- | --- | --- | --- | --- | --- | --- | --- | --- | --- | --- | --- | | Human hg19 chr19 18197570 18197742 - **AG** | TCTTTTCTCCTTGCTCAGCTTCAATGTGTTCCGGAGTGGGGACGGGGTGGCTGAACCTCGCAGGTGGCAGAGAGGCTCCCCTGGGGCTGTGGGGCTCTACGTGGATCCGATGGAGCCGCTGGTGACCTGGGTGGTCCCCCTCCTCTTCCTCTTCCTGCTGTCCAGGCAGGGCG|  |  |  |  |  |  |  |  |  |  |  |  |  |  |  |  |  |  |  |  |  |  |  |  |  |  |  |  |  |  |  |  |  |  |  |  |  |  |  |  | | --- | --- | --- | --- | --- | --- | --- | --- | --- | --- | --- | --- | --- | --- | --- | --- | --- | --- | --- | --- | --- | --- | --- | --- | --- | --- | --- | --- | --- | --- | --- | --- | --- | --- | --- | --- | --- | --- | --- | --- | | Chimp panTro2 chr19 18529673 18529845 - **GG** | TCTTTTCTCCTTGCTCAGCTTCAATGTGTTCCGGAGTAGGGACGGGGTGGCTGAACCTCGCAGGTGGCAGAGAGGCTCCCCTGGGGCTGTGGGGCTCTACGTGGATCCGATGGGGCCGCTGGTGACCTGGGTGGTCCCCCTCCTCTTCCTCTTCCTGCTGTCCAGGCAGGGTG|  |  |  |  |  |  |  |  |  |  |  |  |  |  |  |  |  |  |  |  |  |  |  |  |  |  |  |  |  |  |  |  | | --- | --- | --- | --- | --- | --- | --- | --- | --- | --- | --- | --- | --- | --- | --- | --- | --- | --- | --- | --- | --- | --- | --- | --- | --- | --- | --- | --- | --- | --- | --- | --- | | Gorilla gorGor1 Supercontig\_0081814 5788 5960 + **GG** | TCTTTTCTCCTTGCTCAGCTTCAATGTGTTCCGGAGTGGGGACGGGGTGGCTGAACCTCGCAGGTGGCAGAGAGGCTCCCCTGGGGCTGTGGGGCTCTACGTGGATCCGATGGGGCCGCTGGTGACCTGGGTGGTCCCCCTCCTCTTCCTCTTCCTGCTGTCCAGGCAGGGCG|  |  |  |  |  |  |  |  |  |  |  |  |  |  |  |  |  |  |  |  |  |  |  |  | | --- | --- | --- | --- | --- | --- | --- | --- | --- | --- | --- | --- | --- | --- | --- | --- | --- | --- | --- | --- | --- | --- | --- | --- | | Orangutan ponAbe2 chr19 18486094 18486266 - **GG** | CCTTTTTTCCTTGCTCAGCTTCAATGTGTTCCGGAGTGGGGACGGGGTGGCTGAACCTCGCAGGTGGCAGAGAGGCTCCCCTGGGGCTGTGGGGCTCTGCGTGGATCCGATGGGGCCGCTGGTGACCTGGGTGGTCCCCCTCCTCTTCCTCTTCCTGCTGTCCAGGCAGGGTG|  |  |  |  |  |  |  |  |  |  |  |  |  |  |  |  | | --- | --- | --- | --- | --- | --- | --- | --- | --- | --- | --- | --- | --- | --- | --- | --- | | Rhesus rheMac2 chr19 17658284 17658456 - **GG** | TCTTTTTCCTTTGTTCAGCTTCAATGTGTTCCGGAGTGGGGACGGGGTGGCTGAACCTCGCAGGTGGCAGAGAGGCTCCCCTGGGGCTGTGGGGCTCTGCGTGGATCCAATGGGGCCGCTGGTGACCTGGGTGGTCCCCCTCCTCCTCCTCTTCCTGCGATCCAGGCAGGGTG|  |  |  |  |  |  |  |  | | --- | --- | --- | --- | --- | --- | --- | --- | | Baboon papHam1 scaffold15696 8972 9144 - **GG** | TCTTTTTCCCTTGTTCAGCTTCAATGTGTTCCGGAGTGGGGACGGGGTGGCTGAACCTCGCAGGTGGCAGAGAGGCTCCCCTGGGGCTGTGGGGCTCTGCGTGGATCCAATGGGGCCGCTGGTGACCTGGGTGGTCCCCCTCCTCCTCCTCTTCCTGCGATCCAGGCAGGGTG | | | | | | | | | | | | | | | | | | | | | | | | | | | | | | | | | | | | | | | | | | | | | | | | | |

**Alignment** (splice site sequences are in lowercase)  

```
Human      agTCTTTTCTCCTTGCTCAGCTTCAATGTGTTCCGGAGTGGGGACGGGGTGGCTGAACCTCGCAGGTGGCAGAGAGGCTC
Chimp      g......................................A........................................
Gorilla    g...............................................................................
Orangutan  g.C.....T.......................................................................
Rhesus     g.......TC.T...T................................................................
Baboon     g.......TC.....T................................................................

Human      CCCTGGGGCTGTGGGGCTCTACGTGGATCCGATGGAGCCGCTGGTGACCTGGGTGGTCCCCCTCCTCTTCCTCTTCCTGC
Chimp      ...................................G............................................
Gorilla    ...................................G............................................
Orangutan  ....................G..............G............................................
Rhesus     ....................G.........A....G...............................C............
Baboon     ....................G.........A....G...............................C............

Human      TGTCCAGGCAGGGCGgt
Chimp      .............T...
Gorilla    .................
Orangutan  .............T...
Rhesus     GA...........T...
Baboon     GA...........T...
```

---

## 39. uc002ohv.1\_3\_3

**Summary**  

|  |  |  |  |  |  |  |  |  |  |  |  |  |  |  |  |  |  |  |  |  |  |  |  |  |  |
| --- | --- | --- | --- | --- | --- | --- | --- | --- | --- | --- | --- | --- | --- | --- | --- | --- | --- | --- | --- | --- | --- | --- | --- | --- | --- |
| No Exon ID Position (hg19) Dir Human acceptor Chimp acceptor Category Usage Gene symbol Protein accession mRNA accession Gene title Note|  |  |  |  |  |  |  |  |  |  |  |  |  | | --- | --- | --- | --- | --- | --- | --- | --- | --- | --- | --- | --- | --- | | 39 uc002ohv.1\_3\_3 chr19:38795205 + AG GG (A2) shift; increase; frameshift alternative IMUP Q9GZP8-2 AB038318.1 Immortalization up-regulated protein  | | | | | | | | | | | | | | | | | | | | | | | | | |

**Orthologs**  

|  |  |  |  |  |  |  |  |  |  |  |  |  |  |  |  |  |  |  |  |  |  |  |  |  |  |  |  |  |  |  |  |  |  |  |  |  |  |  |  |  |  |  |  |  |  |  |  |  |  |  |  |  |  |  |  |
| --- | --- | --- | --- | --- | --- | --- | --- | --- | --- | --- | --- | --- | --- | --- | --- | --- | --- | --- | --- | --- | --- | --- | --- | --- | --- | --- | --- | --- | --- | --- | --- | --- | --- | --- | --- | --- | --- | --- | --- | --- | --- | --- | --- | --- | --- | --- | --- | --- | --- | --- | --- | --- | --- | --- | --- |
| Species Assembly Chromosome Exon start Exon end Dir Acceptor Exon sequence|  |  |  |  |  |  |  |  |  |  |  |  |  |  |  |  |  |  |  |  |  |  |  |  |  |  |  |  |  |  |  |  |  |  |  |  |  |  |  |  |  |  |  |  |  |  |  |  | | --- | --- | --- | --- | --- | --- | --- | --- | --- | --- | --- | --- | --- | --- | --- | --- | --- | --- | --- | --- | --- | --- | --- | --- | --- | --- | --- | --- | --- | --- | --- | --- | --- | --- | --- | --- | --- | --- | --- | --- | --- | --- | --- | --- | --- | --- | --- | --- | | Human hg19 chr19 38795205 38795324 + **AG** | GCCTCCAACTTCAGGGGGCTGGGTAAGGGGCGCCGCCTCACTGCCGCACCTCCATCCAGCAAGGACACCACAGCTCTTCCGACTCCAGCAGCAGCTCCAGCGATTCGGACACGGATGTGA|  |  |  |  |  |  |  |  |  |  |  |  |  |  |  |  |  |  |  |  |  |  |  |  |  |  |  |  |  |  |  |  |  |  |  |  |  |  |  |  | | --- | --- | --- | --- | --- | --- | --- | --- | --- | --- | --- | --- | --- | --- | --- | --- | --- | --- | --- | --- | --- | --- | --- | --- | --- | --- | --- | --- | --- | --- | --- | --- | --- | --- | --- | --- | --- | --- | --- | --- | | Chimp panTro2 chr19 43858069 43858187 + **GG** | GCCTCCAACTTCGGGGGCTGGGTAAGGGGCGCCGCCTCACTGCCGCACCTCCATCCAGCAAGGACACCAGAGCTCTTCCGACTCCAGCAGCAGCTCCAGCGATTCGGACACGGATGTGA|  |  |  |  |  |  |  |  |  |  |  |  |  |  |  |  |  |  |  |  |  |  |  |  |  |  |  |  |  |  |  |  | | --- | --- | --- | --- | --- | --- | --- | --- | --- | --- | --- | --- | --- | --- | --- | --- | --- | --- | --- | --- | --- | --- | --- | --- | --- | --- | --- | --- | --- | --- | --- | --- | | Orangutan ponAbe2 chr19 39167295 39167413 + **GG** | GCTTCCAACTTCGGGGGCTGGGTAAGGGGCACCGCCTCACTGCCGCACCTCCATCCAGCAAGGACACCACAGCTCTTCCGACTCTAGCAGCAGCTCCAGCGACTCGGACACGGATGTGA|  |  |  |  |  |  |  |  |  |  |  |  |  |  |  |  |  |  |  |  |  |  |  |  | | --- | --- | --- | --- | --- | --- | --- | --- | --- | --- | --- | --- | --- | --- | --- | --- | --- | --- | --- | --- | --- | --- | --- | --- | | Rhesus rheMac2 chr19 44633729 44633847 + **GG** | GCTTCCAACTTCGGGGGCTGGGTAAGGGGCACCGCCTCACTGCCGCACCTCCATCCAGCAAGGACACCACAGCTCTTCCGACTCCAGCAGCAGCTCCAGCGACTCGGACATGGATGTGA|  |  |  |  |  |  |  |  |  |  |  |  |  |  |  |  | | --- | --- | --- | --- | --- | --- | --- | --- | --- | --- | --- | --- | --- | --- | --- | --- | | Baboon papHam1 scaffold23503 5540 5658 - **GG** | GCTTCCAACTTCGGGGGCTGGGTAAGGGGCACCGCCTCACTGCCGCACCTCCATCCAGCAAGGACACCACAGCTCTTCCGACTCCAGCAGCAGCTCCAGCGACTCGGACACGGATGTGA|  |  |  |  |  |  |  |  | | --- | --- | --- | --- | --- | --- | --- | --- | | Marmoset calJac1 Contig12262 23968 24085 + **GG** | GCTTCCAACTTCAGGGGATGCGTAGGGGCCCTGCCTCACTGCCACACCCCCATCCAGCAAGGCCGCCACAGCTCTTCGGACTCCAGCAGCAGCTCCAGCGACTCGGACACGAATGTGA | | | | | | | | | | | | | | | | | | | | | | | | | | | | | | | | | | | | | | | | | | | | | | | | | |

**Alignment** (splice site sequences are in lowercase)  

```
Human      agGCCTCCAACTTCAGGGGGCTGGGTAAGGGGCGCCGCCTCACTGCCGCACCTCCATCCAGCAAGGACACCACAGCTCTT
Chimp      g.............-.........................................................G.......
Orangutan  g...T.........-..................A..............................................
Rhesus     g...T.........-..................A..............................................
Baboon     g...T.........-..................A..............................................
Marmoset   g...T.........-A....A..C..-......C.T...........A....C.............C.G...........

Human      CCGACTCCAGCAGCAGCTCCAGCGATTCGGACACGGATGTGA
Chimp      ..........................................
Orangutan  .......T.................C................
Rhesus     .........................C.......T........
Baboon     .........................C................
Marmoset   .G.......................C.........A......
```

---

## 40. uc002qfm.1\_3\_10

**Summary**  

|  |  |  |  |  |  |  |  |  |  |  |  |  |  |  |  |  |  |  |  |  |  |  |  |  |  |
| --- | --- | --- | --- | --- | --- | --- | --- | --- | --- | --- | --- | --- | --- | --- | --- | --- | --- | --- | --- | --- | --- | --- | --- | --- | --- |
| No Exon ID Position (hg19) Dir Human acceptor Chimp acceptor Category Usage Gene symbol Protein accession mRNA accession Gene title Note|  |  |  |  |  |  |  |  |  |  |  |  |  | | --- | --- | --- | --- | --- | --- | --- | --- | --- | --- | --- | --- | --- | | 40 uc002qfm.1\_3\_10 chr19:54872813 - AG GG (A3) shift; decrease; inframe alternative LAIR1 Q6GTX8-3 AF251509.2 Leukocyte-associated immunoglobulin-like receptor 1 NAGNAG | | | | | | | | | | | | | | | | | | | | | | | | | |

**Orthologs**  

|  |  |  |  |  |  |  |  |  |  |  |  |  |  |  |  |  |  |  |  |  |  |  |  |  |  |  |  |  |  |  |  |  |  |  |  |  |  |  |  |  |  |  |  |  |  |  |  |  |  |  |  |  |  |  |  |  |  |  |  |  |  |  |  |
| --- | --- | --- | --- | --- | --- | --- | --- | --- | --- | --- | --- | --- | --- | --- | --- | --- | --- | --- | --- | --- | --- | --- | --- | --- | --- | --- | --- | --- | --- | --- | --- | --- | --- | --- | --- | --- | --- | --- | --- | --- | --- | --- | --- | --- | --- | --- | --- | --- | --- | --- | --- | --- | --- | --- | --- | --- | --- | --- | --- | --- | --- | --- | --- |
| Species Assembly Chromosome Exon start Exon end Dir Acceptor Exon sequence|  |  |  |  |  |  |  |  |  |  |  |  |  |  |  |  |  |  |  |  |  |  |  |  |  |  |  |  |  |  |  |  |  |  |  |  |  |  |  |  |  |  |  |  |  |  |  |  |  |  |  |  |  |  |  |  | | --- | --- | --- | --- | --- | --- | --- | --- | --- | --- | --- | --- | --- | --- | --- | --- | --- | --- | --- | --- | --- | --- | --- | --- | --- | --- | --- | --- | --- | --- | --- | --- | --- | --- | --- | --- | --- | --- | --- | --- | --- | --- | --- | --- | --- | --- | --- | --- | --- | --- | --- | --- | --- | --- | --- | --- | | Human hg19 chr19 54872523 54872813 - **AG** | ATCTGCCCAGACCCTCCATCTCGGCTGAGCCAGGCACCGTGATCCCCCTGGGGAGCCATGTGACTTTCGTGTGCCGGGGCCCGGTTGGGGTTCAAACATTCCGCCTGGAGAGGGAGAGTAGATCCACATACAATGATACTGAAGATGTGTCTCAAGCTAGTCCATCTGAGTCAGAGGCCAGATTCCGCATTGACTCAGTAAGTGAAGGAAATGCCGGGCCTTATCGCTGCATCTATTATAAGCCCCCTAAATGGTCTGAGCAGAGTGACTACCTGGAGCTGCTGGTGAAAG|  |  |  |  |  |  |  |  |  |  |  |  |  |  |  |  |  |  |  |  |  |  |  |  |  |  |  |  |  |  |  |  |  |  |  |  |  |  |  |  |  |  |  |  |  |  |  |  | | --- | --- | --- | --- | --- | --- | --- | --- | --- | --- | --- | --- | --- | --- | --- | --- | --- | --- | --- | --- | --- | --- | --- | --- | --- | --- | --- | --- | --- | --- | --- | --- | --- | --- | --- | --- | --- | --- | --- | --- | --- | --- | --- | --- | --- | --- | --- | --- | | Chimp panTro2 chr19 60085639 60085929 - **GG** | CCCTGCCCAGACCCTCCATCTCGGCTGAGCCAGGCACTGTGATCCCCCAGGGGAGCCATGTGACTTTCGTGTGCCGGGGCCCGGTTGGGGTTCAAACATTCCGCCTGGAGAGGGAGAGTAGATCCACATACAATGATACTGAAGATGTGTCTCAAGCTAGTCCATCTGAGTCAGAGGCCAGATTCCGCATTGACTCAGTAAGTGAAGGAAATGCCGGGCTTTATCGCTGCATCTATTATAAGCCCCCTAAATGGTCTGAGCAGAGTGACTACCTGGAGCTGCTGGTGAAAG|  |  |  |  |  |  |  |  |  |  |  |  |  |  |  |  |  |  |  |  |  |  |  |  |  |  |  |  |  |  |  |  |  |  |  |  |  |  |  |  | | --- | --- | --- | --- | --- | --- | --- | --- | --- | --- | --- | --- | --- | --- | --- | --- | --- | --- | --- | --- | --- | --- | --- | --- | --- | --- | --- | --- | --- | --- | --- | --- | --- | --- | --- | --- | --- | --- | --- | --- | | Orangutan ponAbe2 chr19 56172321 56172611 - **GG** | CCCTGCCCAGACCCTCCATCTCGGCTGAGCCAGGCACCACGATCCCCCCGGGAAGCCGTGTGACTTTCGTGTGCCGGGGCCCGGCTGGGGTTCAAACATTCCGCCTGGAGAGGGAGAGTAGATCCAAGTACAATGATACTAATGACGTGTCTCAAGCTAGTTCATCTGAGTCAGAGGCCAGATTCCGCATTGACTCAGTAAGTGAAGACAGTGCTGGGTGTTATCGCTGCCTCTATTTTAAGTCCTCCAGATGGTCTGAGCACAGTGACTACCTGGAGCTGCTGGTGAAAG|  |  |  |  |  |  |  |  |  |  |  |  |  |  |  |  |  |  |  |  |  |  |  |  |  |  |  |  |  |  |  |  | | --- | --- | --- | --- | --- | --- | --- | --- | --- | --- | --- | --- | --- | --- | --- | --- | --- | --- | --- | --- | --- | --- | --- | --- | --- | --- | --- | --- | --- | --- | --- | --- | | Rhesus rheMac2 chr19 60508309 60508599 - **GC** | CCCTGCCCAGACCCTCCATCTCAGCTGAGCCAGGCACCGTGATCCCCCCGGGGAGGCCTGTGACTATCGTGTGCCGGGGCCCGGTTGGGGTTGACCAATTCCGCCTGGAGTGGGAGGGTAGATCCAAGTTCGATGATACTAAGAATGTGTCTCAAGCTAGTTCATCTGAGTCCGAGACCAGATTCCGCATTGACTCAGTAAGTGAAGGAAATGCCGGACATTATCGCTGCCTCTATGTGAAGTCCAGCAGATGGTCTCAGCGCAGTGACTACCTGGACCTGGTGGTGAAAG|  |  |  |  |  |  |  |  |  |  |  |  |  |  |  |  |  |  |  |  |  |  |  |  | | --- | --- | --- | --- | --- | --- | --- | --- | --- | --- | --- | --- | --- | --- | --- | --- | --- | --- | --- | --- | --- | --- | --- | --- | | Marmoset calJac1 Contig7093 14251 14541 - **GG** | CCCTGCCCAGACCCTCCATCTCGGCTGAGCCAGGCACCGTGATCCCCCTGGGGAGCCCTGTGACTTTCGTGTGCCGGGGCCCAGTTGGGGTTCACGAATTCCGCCTGGAGAGGGAGAATAGAGCCCAGTACAAAGATAATTATGATGTGTCTCTAGTTAGTCCATTTTTGTCAGAGGCCACATTCCGCATTGACTCAGTGAGTGAGGACAGCGCCGGGCATTATCGCTGCATCTATCATAAGGCCTCCAGATGGTCTCAGCACAGTGAGCAGCTGGAGCTGGTGGTGAAAG|  |  |  |  |  |  |  |  |  |  |  |  |  |  |  |  | | --- | --- | --- | --- | --- | --- | --- | --- | --- | --- | --- | --- | --- | --- | --- | --- | | Tarsier tarSyr1 scaffold\_442100 350 655 + **GG** | TCCTGCCCAGACCCTCCATCTCCACTGAGCCAGATGCTGTGATCCCACTGTTACTGCCTGTGACCCTAGTGTGCCAGGGCCCAGCTAGCATTGAGAAATTCCATCTGGAGAGGGAGGGTAGAACAAATGGGTTCAGAGATGCTAAACTTCAGTCTCAGTTTGACGTTTCTGAAACAGAGGCCAGATTCCCCATCGACTCACTAACTGAAGATAATGCTGGGAATTATCACTGTGTCTATCTGGATGCAGAGTTCTGTATCTGGTCTGAGCCCAGTGACTACCTGAAGCTGGTGTTGTATGTGACAG|  |  |  |  |  |  |  |  | | --- | --- | --- | --- | --- | --- | --- | --- | | Dog canFam2 chr1 105992347 105992625 + **GG** | TCCTGCCCAGCCCCTCCATCTGGGCCCAGCCGAGCTCTGAGATTCCCCGGGGGCAGCCGGTGACCATCGTGTGCCAGGGCCCTGCTGGGGCTGAGACATTCCGCCTGGAGAAGGAGGGAAGTGCTCTACATAAAGATGTGAGGAACCCACAACATGAGACGCAGGCCAGATTCCCCATCCCTGCGGTGGGTGAAGACACTGCCCGGCGCTATCGCTGCCTCTATAATAAAGACGGCACCTGGTCTGACCGCAGCAAGGAACTGCAGCTGGTGGTGACAG | | | | | | | | | | | | | | | | | | | | | | | | | | | | | | | | | | | | | | | | | | | | | | | | | | | | | | | | |

**Alignment** (splice site sequences are in lowercase)  

```
Human      agATCTGCCCAGACCCTCCATCTCGGCTGAGCCAGGCACCGTGATCCCCCTGGGGAGCCATGTGACTTTCGTGTGCCGGG
Chimp      g.CC...................................T..........A.............................
Orangutan  g.CC....................................AC........C...A....G....................
Rhesus     gcCC....................A.........................C......G.C.......A............
Marmoset   g.CC.......................................................C....................
Tarsier    g.TC....................CA.........ATG.T........A...TTACTG.C......CC.A.......A..
Dog        g.TC........C..........G...CC....GA..T.T.A...T....G....CAG.CG.....CA.........A..

Human      GCCCGGTTGGGGTTCAAACATTCCGCCTGGAGAGGGAGAGTAGATCCAC---ATACAATGATACTGAAGATGTGTCTCAA
Chimp      .................................................---............................
Orangutan  ......C.........................................A---G............A.T..C.........
Rhesus     ..............G.CCA.............T.....G.........A---G.T.G........A.GA...........
Marmoset   ....A...........CGA....................A....G..CA---G.....A....A.T.T..........T.
Tarsier    ....A.C.A.CA..G.G.A.....AT............G.....A.A.ATGGG.T..GA...G..A..CT.CA......G
Dog        ....T.C.....C.G.G................A....G.A..TG.TCT---.C.T..A...GTGAGGA.CCCACAA..-

Human      GCTAGTCCATCTGAGTCAGAGGCCAGATTCCGCATTGACTCAGTAAGTGAAGGAAATGCCGGGCCTTATCGCTGCATCTA
Chimp      ................................................................T...............
Orangutan  ......T.............................................AC.G...T...TG..........C....
Rhesus     ......T..........C...A........................................A.A..........C....
Marmoset   .T........T.TT...........C..................G.....G.AC.GC.......A...............
Tarsier    TT.GACGTT.....AA...............C...C......C...C.....AT.....T...AA.....A...TG....
Dog        -----------....A.GC............C...CCCTG.G..GG......AC.C....C...GC.........C....

Human      TTAT------AAGCCCCCTAAATGGTCTGAGCAGAGTGACTACCTGGAGCTGCTG------GTGAAAGgt
Chimp      ....------.............................................------.........
Orangutan  ..T.------...T..T.C.G............C.....................------.........
Rhesus     .GTG------...T..AGC.G.......C...GC..............C...G..------.........
Marmoset   .C..------...G..T.C.G.......C....C.....GC.G.........G..------.........
Tarsier    .CTGGATGCAG..TT.TG..TC..........CC............A.....G..TTGTAT....C....
Dog        .A..------..AGA.GGC.CC........C.GC..CA.GG.A...C.....G..------....C....
```

---

## 41. uc002qiy.2\_10\_22

**Summary**  

|  |  |  |  |  |  |  |  |  |  |  |  |  |  |  |  |  |  |  |  |  |  |  |  |  |  |
| --- | --- | --- | --- | --- | --- | --- | --- | --- | --- | --- | --- | --- | --- | --- | --- | --- | --- | --- | --- | --- | --- | --- | --- | --- | --- |
| No Exon ID Position (hg19) Dir Human acceptor Chimp acceptor Category Usage Gene symbol Protein accession mRNA accession Gene title Note|  |  |  |  |  |  |  |  |  |  |  |  |  | | --- | --- | --- | --- | --- | --- | --- | --- | --- | --- | --- | --- | --- | | 41 uc002qiy.2\_10\_22 chr19:55606968 - AG TG (A3) shift; decrease; inframe alternative PPP1R12C Q9BZL4-3 AB209452.1 protein phosphatase 1, regulatory subunit 12C NAGNAG | | | | | | | | | | | | | | | | | | | | | | | | | |

**Orthologs**  

|  |  |  |  |  |  |  |  |  |  |  |  |  |  |  |  |  |  |  |  |  |  |  |  |  |  |  |  |  |  |  |  |  |  |  |  |  |  |  |  |  |  |  |  |  |  |  |  |  |  |  |  |  |  |  |  |  |  |  |  |  |  |  |  |  |  |  |  |  |  |  |  |  |  |  |  |  |  |  |  |  |  |  |  |  |  |  |  |  |  |  |  |  |  |  |  |
| --- | --- | --- | --- | --- | --- | --- | --- | --- | --- | --- | --- | --- | --- | --- | --- | --- | --- | --- | --- | --- | --- | --- | --- | --- | --- | --- | --- | --- | --- | --- | --- | --- | --- | --- | --- | --- | --- | --- | --- | --- | --- | --- | --- | --- | --- | --- | --- | --- | --- | --- | --- | --- | --- | --- | --- | --- | --- | --- | --- | --- | --- | --- | --- | --- | --- | --- | --- | --- | --- | --- | --- | --- | --- | --- | --- | --- | --- | --- | --- | --- | --- | --- | --- | --- | --- | --- | --- | --- | --- | --- | --- | --- | --- | --- | --- |
[truncated: 92,595 more chars]
