# Supplementary material for: Human-specific protein isoforms produced by novel splice sites in the human genome after the human-chimpanzee divergence
Source: BMC Bioinformatics. 2012 Nov 13;13:299. doi: 10.1186/1471-2105-13-299 (PMC3538075; doi:10.1186/1471-2105-13-299)
Supplement: Additional file 5 — Alignments of the exon and splice donor sequences. [file 1471-2105-13-299-S5.html]

Table S4. Alignments of the exon and splice donor sequences

# Table S4. Alignments of the exon and splice donor sequences

---

## 1. uc001cjm.2\_6\_7

**Summary**  

|  |  |  |  |  |  |  |  |  |  |  |  |  |  |  |  |  |  |  |  |  |  |  |  |  |  |
| --- | --- | --- | --- | --- | --- | --- | --- | --- | --- | --- | --- | --- | --- | --- | --- | --- | --- | --- | --- | --- | --- | --- | --- | --- | --- |
| No Exon ID Position (hg19) Dir Human donor Chimp donor Category Usage Gene symbol Protein accession mRNA accession Gene title Note|  |  |  |  |  |  |  |  |  |  |  |  |  | | --- | --- | --- | --- | --- | --- | --- | --- | --- | --- | --- | --- | --- | | 1 uc001cjm.2\_6\_7 chr1:43917099 - GT GC (D4) shift; decrease; frameshift alternative HYI NP\_112484.3 NM\_031207.5 putative hydroxypyruvate isomerase  | | | | | | | | | | | | | | | | | | | | | | | | | |

**Orthologs**  

|  |  |  |  |  |  |  |  |  |  |  |  |  |  |  |  |  |  |  |  |  |  |  |  |  |  |  |  |  |  |  |  |  |  |  |  |  |  |  |  |  |  |  |  |  |  |  |  |  |  |  |  |  |  |  |  |  |  |  |  |  |  |  |  |  |  |  |  |  |  |  |  |  |  |  |  |  |  |  |  |  |  |  |  |  |  |  |  |  |  |  |  |  |  |  |  |
| --- | --- | --- | --- | --- | --- | --- | --- | --- | --- | --- | --- | --- | --- | --- | --- | --- | --- | --- | --- | --- | --- | --- | --- | --- | --- | --- | --- | --- | --- | --- | --- | --- | --- | --- | --- | --- | --- | --- | --- | --- | --- | --- | --- | --- | --- | --- | --- | --- | --- | --- | --- | --- | --- | --- | --- | --- | --- | --- | --- | --- | --- | --- | --- | --- | --- | --- | --- | --- | --- | --- | --- | --- | --- | --- | --- | --- | --- | --- | --- | --- | --- | --- | --- | --- | --- | --- | --- | --- | --- | --- | --- | --- | --- | --- | --- |
| Species Assembly Chromosome Exon start Exon end Dir Donor Exon sequence|  |  |  |  |  |  |  |  |  |  |  |  |  |  |  |  |  |  |  |  |  |  |  |  |  |  |  |  |  |  |  |  |  |  |  |  |  |  |  |  |  |  |  |  |  |  |  |  |  |  |  |  |  |  |  |  |  |  |  |  |  |  |  |  |  |  |  |  |  |  |  |  |  |  |  |  |  |  |  |  |  |  |  |  |  |  |  |  | | --- | --- | --- | --- | --- | --- | --- | --- | --- | --- | --- | --- | --- | --- | --- | --- | --- | --- | --- | --- | --- | --- | --- | --- | --- | --- | --- | --- | --- | --- | --- | --- | --- | --- | --- | --- | --- | --- | --- | --- | --- | --- | --- | --- | --- | --- | --- | --- | --- | --- | --- | --- | --- | --- | --- | --- | --- | --- | --- | --- | --- | --- | --- | --- | --- | --- | --- | --- | --- | --- | --- | --- | --- | --- | --- | --- | --- | --- | --- | --- | --- | --- | --- | --- | --- | --- | --- | --- | | Human hg19 chr1 43917099 43917215 - **GT** | GGCATGTGCAGGTGGCACAGGTCCCAGGCCGAGGGGAGCCCAGCAGCCCCGGAGAGCTGAATTTCCCCTATCTGTTTCAACTGCTGGAAGATGAAGGCTACAAAGGCTTCGTGGGCT|  |  |  |  |  |  |  |  |  |  |  |  |  |  |  |  |  |  |  |  |  |  |  |  |  |  |  |  |  |  |  |  |  |  |  |  |  |  |  |  |  |  |  |  |  |  |  |  |  |  |  |  |  |  |  |  |  |  |  |  |  |  |  |  |  |  |  |  |  |  |  |  |  |  |  |  |  |  |  |  | | --- | --- | --- | --- | --- | --- | --- | --- | --- | --- | --- | --- | --- | --- | --- | --- | --- | --- | --- | --- | --- | --- | --- | --- | --- | --- | --- | --- | --- | --- | --- | --- | --- | --- | --- | --- | --- | --- | --- | --- | --- | --- | --- | --- | --- | --- | --- | --- | --- | --- | --- | --- | --- | --- | --- | --- | --- | --- | --- | --- | --- | --- | --- | --- | --- | --- | --- | --- | --- | --- | --- | --- | --- | --- | --- | --- | --- | --- | --- | --- | | Chimp panTro2 chr1 44149144 44149260 - **GC** | GGCATGTGCAGGTGGCACAGGTCCCAGGCCGAGGGGAGCCCAGCAGCCCCGGAGAGCTGAATTTCCCCTATCTGTTTCAACTGCTGGAAGATGAAGGCTACAAAGGCTTCGTGGGCT|  |  |  |  |  |  |  |  |  |  |  |  |  |  |  |  |  |  |  |  |  |  |  |  |  |  |  |  |  |  |  |  |  |  |  |  |  |  |  |  |  |  |  |  |  |  |  |  |  |  |  |  |  |  |  |  |  |  |  |  |  |  |  |  |  |  |  |  |  |  |  |  | | --- | --- | --- | --- | --- | --- | --- | --- | --- | --- | --- | --- | --- | --- | --- | --- | --- | --- | --- | --- | --- | --- | --- | --- | --- | --- | --- | --- | --- | --- | --- | --- | --- | --- | --- | --- | --- | --- | --- | --- | --- | --- | --- | --- | --- | --- | --- | --- | --- | --- | --- | --- | --- | --- | --- | --- | --- | --- | --- | --- | --- | --- | --- | --- | --- | --- | --- | --- | --- | --- | --- | --- | | Gorilla gorGor1 Supercontig\_0003107 28916 29032 - **GC** | GGCATGTGCAGGTGGCACAGGTCCCAGGCCGAGGGGAGCCCAGCAGCCCCGGAGAGCTGAATTTCCCCTATCTGTTTCAACTGCTGGAAGATGAAGGCTACAAAGGCTTCGTGGGCT|  |  |  |  |  |  |  |  |  |  |  |  |  |  |  |  |  |  |  |  |  |  |  |  |  |  |  |  |  |  |  |  |  |  |  |  |  |  |  |  |  |  |  |  |  |  |  |  |  |  |  |  |  |  |  |  |  |  |  |  |  |  |  |  | | --- | --- | --- | --- | --- | --- | --- | --- | --- | --- | --- | --- | --- | --- | --- | --- | --- | --- | --- | --- | --- | --- | --- | --- | --- | --- | --- | --- | --- | --- | --- | --- | --- | --- | --- | --- | --- | --- | --- | --- | --- | --- | --- | --- | --- | --- | --- | --- | --- | --- | --- | --- | --- | --- | --- | --- | --- | --- | --- | --- | --- | --- | --- | --- | | Orangutan ponAbe2 chr1 186466683 186466799 + **GC** | GGCATGTGCAGGTGGCACAGGTCCCAGGCCGAGGGGAGCCCAGCAGCCCTGGAGAGCTGAATTTCCCCTATCTGTTTCAACTGCTGGAAGATGAAGGCTACAAAGGCTTTGTGGGCT|  |  |  |  |  |  |  |  |  |  |  |  |  |  |  |  |  |  |  |  |  |  |  |  |  |  |  |  |  |  |  |  |  |  |  |  |  |  |  |  |  |  |  |  |  |  |  |  |  |  |  |  |  |  |  |  | | --- | --- | --- | --- | --- | --- | --- | --- | --- | --- | --- | --- | --- | --- | --- | --- | --- | --- | --- | --- | --- | --- | --- | --- | --- | --- | --- | --- | --- | --- | --- | --- | --- | --- | --- | --- | --- | --- | --- | --- | --- | --- | --- | --- | --- | --- | --- | --- | --- | --- | --- | --- | --- | --- | --- | --- | | Rhesus rheMac2 chr1 46359094 46359210 - **GC** | GGCATGTGCAGGTGGCACAGGTCCCAGGCCGAGGGGAGCCCAGCAGCCCCGGAGAGCTGAATTTCCCCTATCTGTTTCAACTGCTGGAAGATGAAGGCTACAAAGGCTTTGTGGGCT|  |  |  |  |  |  |  |  |  |  |  |  |  |  |  |  |  |  |  |  |  |  |  |  |  |  |  |  |  |  |  |  |  |  |  |  |  |  |  |  |  |  |  |  |  |  |  |  | | --- | --- | --- | --- | --- | --- | --- | --- | --- | --- | --- | --- | --- | --- | --- | --- | --- | --- | --- | --- | --- | --- | --- | --- | --- | --- | --- | --- | --- | --- | --- | --- | --- | --- | --- | --- | --- | --- | --- | --- | --- | --- | --- | --- | --- | --- | --- | --- | | Marmoset calJac1 Contig119 654784 654900 - **GC** | GGCACGTGCAGGTGGCACAGGTCCCAGGCCGAGGGGAGCCCAGCAGCCCCGGAGAGCTGAACTTCCCCTATCTGTTTCAACTGCTGGAAGATGAAGGCTACAAAGGTTTTGTGGGCT|  |  |  |  |  |  |  |  |  |  |  |  |  |  |  |  |  |  |  |  |  |  |  |  |  |  |  |  |  |  |  |  |  |  |  |  |  |  |  |  | | --- | --- | --- | --- | --- | --- | --- | --- | --- | --- | --- | --- | --- | --- | --- | --- | --- | --- | --- | --- | --- | --- | --- | --- | --- | --- | --- | --- | --- | --- | --- | --- | --- | --- | --- | --- | --- | --- | --- | --- | | Tarsier tarSyr1 scaffold\_96244 290 406 + **GC** | GACATGTGCAGGTGGCACAGGTCCCTGGCCGAGGGGAACCCAGCAGCCCTGGAGAGCTGAACTTCCCCTATCTATTTCAACTGCTGGAGGATGAAGGCTACAAAGGCTTTGTGGGTT|  |  |  |  |  |  |  |  |  |  |  |  |  |  |  |  |  |  |  |  |  |  |  |  |  |  |  |  |  |  |  |  | | --- | --- | --- | --- | --- | --- | --- | --- | --- | --- | --- | --- | --- | --- | --- | --- | --- | --- | --- | --- | --- | --- | --- | --- | --- | --- | --- | --- | --- | --- | --- | --- | | Lemur micMur1 scaffold\_754 241036 241152 - **GC** | GGCACGTGCAAGTGGCGCAGGTCCCGGGCCGAGGGGAACCCAGCAGCCCTGGAGAGCTGAACTTCCCCTATCTGTTTCAACTGCTGGAAGACGAAGGCTACAAAGGCTTTGTGGGCT|  |  |  |  |  |  |  |  |  |  |  |  |  |  |  |  |  |  |  |  |  |  |  |  | | --- | --- | --- | --- | --- | --- | --- | --- | --- | --- | --- | --- | --- | --- | --- | --- | --- | --- | --- | --- | --- | --- | --- | --- | | Mouse mm9 chr4 118034968 118035084 + **GC** | GGCATGTGCAAGTGGCCCAGGTCCCAGACCGCGGGGAGCCAGGCAGCTCTGGAGAGCTAGACTTTACTTACCTGTTTCAGCTGCTAGAAGATGAGGGCTACCAAGGATTTGTGGGTT|  |  |  |  |  |  |  |  |  |  |  |  |  |  |  |  | | --- | --- | --- | --- | --- | --- | --- | --- | --- | --- | --- | --- | --- | --- | --- | --- | | Cow bosTau4 chr3 109496466 109496582 + **GC** | GGCATGTGCAGGTGGCACAGGTCCCGGGTCGGGGGGAACCCGATAGCCCCGGAGAGCTCAACTTCTCCTATCTGTTCCAACTGCTGGAAGATGAAGGCTACACAGGCTTCGTGGGCT|  |  |  |  |  |  |  |  | | --- | --- | --- | --- | --- | --- | --- | --- | | Dog canFam2 chr15 19740393 19740509 + **GC** | GGCACGTGCAGGTGGCACAGGTCCCGGGCCGAGGGGAGCCTGACAGCCCTGGAGAGCTGAACTTCCCCTATCTATTCCAACTGCTGGAGGACGAAGGCTACAAGGGCTTTGTGGGCT | | | | | | | | | | | | | | | | | | | | | | | | | | | | | | | | | | | | | | | | | | | | | | | | | | | | | | | | | | | | | | | | | | | | | | | | | | | | | | | | | | | | |

**Alignment** (splice site sequences are in lowercase)  

```
Human      agGGCATGTGCAGGTGGCACAGGTCCCAGGCCGAGGGGAGCCCAGCAGCCCCGGAGAGCTGAATTTCCCCTATCTGTTTC
Chimp      ................................................................................
Gorilla    ................................................................................
Orangutan  ...................................................T............................
Rhesus     ................................................................................
Marmoset   ......C........................................................C................
Tarsier    ...A.......................T...........A...........T...........C...........A....
Lemur      ......C.....A.....G........G...........A...........T...........C................
Mouse      ............A.....C..........A...C........AG.....T.T........AG.C..TA.T..C.......
Cow        ...........................G..T..G.....A...GAT..............C..C...T..........C.
Dog        ......C....................G..............TGA......T...........C...........A..C.

Human      AACTGCTGGAAGATGAAGGCTACAAAGGCTTCGTGGGCTgt
Chimp      ........................................c
Gorilla    ........................................c
Orangutan  ...............................T........c
Rhesus     ...............................T........c
Marmoset   ............................T..T........c
Tarsier    ..........G....................T.....T..c
Lemur      .............C.................T........c
Mouse      .G.....A........G......C....A..T.....T..c
Cow        ........................C...............c
Dog        ..........G..C...........G.....T........c
```

---

## 2. uc001gdg.2\_1\_6

**Summary**  

|  |  |  |  |  |  |  |  |  |  |  |  |  |  |  |  |  |  |  |  |  |  |  |  |  |  |
| --- | --- | --- | --- | --- | --- | --- | --- | --- | --- | --- | --- | --- | --- | --- | --- | --- | --- | --- | --- | --- | --- | --- | --- | --- | --- |
| No Exon ID Position (hg19) Dir Human donor Chimp donor Category Usage Gene symbol Protein accession mRNA accession Gene title Note|  |  |  |  |  |  |  |  |  |  |  |  |  | | --- | --- | --- | --- | --- | --- | --- | --- | --- | --- | --- | --- | --- | | 2 uc001gdg.2\_1\_6 chr1:165601511 + GT AT (D7) exonization; novel start alternative MGST3 Q5VV89 BG709310.1 microsomal glutathione S-transferase 3  | | | | | | | | | | | | | | | | | | | | | | | | | |

**Orthologs**  

|  |  |  |  |  |  |  |  |  |  |  |  |  |  |  |  |  |  |  |  |  |  |  |  |  |  |  |  |  |  |  |  |  |  |  |  |  |  |  |  |  |  |  |  |  |  |  |  |  |  |  |  |  |  |  |  |  |  |  |  |  |  |  |  |  |  |  |  |  |  |  |  |
| --- | --- | --- | --- | --- | --- | --- | --- | --- | --- | --- | --- | --- | --- | --- | --- | --- | --- | --- | --- | --- | --- | --- | --- | --- | --- | --- | --- | --- | --- | --- | --- | --- | --- | --- | --- | --- | --- | --- | --- | --- | --- | --- | --- | --- | --- | --- | --- | --- | --- | --- | --- | --- | --- | --- | --- | --- | --- | --- | --- | --- | --- | --- | --- | --- | --- | --- | --- | --- | --- | --- | --- |
| Species Assembly Chromosome Exon start Exon end Dir Donor Exon sequence|  |  |  |  |  |  |  |  |  |  |  |  |  |  |  |  |  |  |  |  |  |  |  |  |  |  |  |  |  |  |  |  |  |  |  |  |  |  |  |  |  |  |  |  |  |  |  |  |  |  |  |  |  |  |  |  |  |  |  |  |  |  |  |  | | --- | --- | --- | --- | --- | --- | --- | --- | --- | --- | --- | --- | --- | --- | --- | --- | --- | --- | --- | --- | --- | --- | --- | --- | --- | --- | --- | --- | --- | --- | --- | --- | --- | --- | --- | --- | --- | --- | --- | --- | --- | --- | --- | --- | --- | --- | --- | --- | --- | --- | --- | --- | --- | --- | --- | --- | --- | --- | --- | --- | --- | --- | --- | --- | | Human hg19 chr1 165601477 165601511 + **GT** | ATGCCCTGGAGGGGAAGGCGCTTCCGGCTCTGCAG|  |  |  |  |  |  |  |  |  |  |  |  |  |  |  |  |  |  |  |  |  |  |  |  |  |  |  |  |  |  |  |  |  |  |  |  |  |  |  |  |  |  |  |  |  |  |  |  |  |  |  |  |  |  |  |  | | --- | --- | --- | --- | --- | --- | --- | --- | --- | --- | --- | --- | --- | --- | --- | --- | --- | --- | --- | --- | --- | --- | --- | --- | --- | --- | --- | --- | --- | --- | --- | --- | --- | --- | --- | --- | --- | --- | --- | --- | --- | --- | --- | --- | --- | --- | --- | --- | --- | --- | --- | --- | --- | --- | --- | --- | | Chimp panTro2 chr1 144961676 144961710 + **AT** | ATGCCCTGGAGGGGAAGGCGCTTCCGGCTCTGCAG|  |  |  |  |  |  |  |  |  |  |  |  |  |  |  |  |  |  |  |  |  |  |  |  |  |  |  |  |  |  |  |  |  |  |  |  |  |  |  |  |  |  |  |  |  |  |  |  | | --- | --- | --- | --- | --- | --- | --- | --- | --- | --- | --- | --- | --- | --- | --- | --- | --- | --- | --- | --- | --- | --- | --- | --- | --- | --- | --- | --- | --- | --- | --- | --- | --- | --- | --- | --- | --- | --- | --- | --- | --- | --- | --- | --- | --- | --- | --- | --- | | Gorilla gorGor1 Supercontig\_0000869 26021 26055 - **AT** | ATGCCCTGGAGGGGAAGGCGCTTCCGGCTCTGCAG|  |  |  |  |  |  |  |  |  |  |  |  |  |  |  |  |  |  |  |  |  |  |  |  |  |  |  |  |  |  |  |  |  |  |  |  |  |  |  |  | | --- | --- | --- | --- | --- | --- | --- | --- | --- | --- | --- | --- | --- | --- | --- | --- | --- | --- | --- | --- | --- | --- | --- | --- | --- | --- | --- | --- | --- | --- | --- | --- | --- | --- | --- | --- | --- | --- | --- | --- | | Orangutan ponAbe2 chr1 85617990 85618024 - **AT** | GTGCCCTGGAGGGGAAGGCGTTTCCGGCTCTGCAG|  |  |  |  |  |  |  |  |  |  |  |  |  |  |  |  |  |  |  |  |  |  |  |  |  |  |  |  |  |  |  |  | | --- | --- | --- | --- | --- | --- | --- | --- | --- | --- | --- | --- | --- | --- | --- | --- | --- | --- | --- | --- | --- | --- | --- | --- | --- | --- | --- | --- | --- | --- | --- | --- | | Rhesus rheMac2 chr1 195474349 195474383 + **AT** | GTGCCCTGGAGGGGAAGGCGCTTCCGGCTTTGCAG|  |  |  |  |  |  |  |  |  |  |  |  |  |  |  |  |  |  |  |  |  |  |  |  | | --- | --- | --- | --- | --- | --- | --- | --- | --- | --- | --- | --- | --- | --- | --- | --- | --- | --- | --- | --- | --- | --- | --- | --- | | Baboon papHam1 scaffold12435 60180 60214 - **AT** | GTGCCCTGGAGGGAAAGGCGCTTCCGGCTTTGCAG|  |  |  |  |  |  |  |  |  |  |  |  |  |  |  |  | | --- | --- | --- | --- | --- | --- | --- | --- | --- | --- | --- | --- | --- | --- | --- | --- | | Marmoset calJac1 Contig2324 294359 294393 + **AT** | GTGCCCTGAAGAGGAAGGCACTTCCGGCTCTGAAG|  |  |  |  |  |  |  |  | | --- | --- | --- | --- | --- | --- | --- | --- | | Mouse mm9 chr1 169322903 169322937 - **CC** | GTGCCCCAGAGAGCAACCAGCTCTCTGCTCTGTTG | | | | | | | | | | | | | | | | | | | | | | | | | | | | | | | | | | | | | | | | | | | | | | | | | | | | | | | | | | | | | | | |

**Alignment** (splice site sequences are in lowercase)  

```
Human      ATGCCCTGGAGGGGAAGGCGCTTCCGGCTCTGCAGgt
Chimp      ...................................a.
Gorilla    ...................................a.
Orangutan  G...................T..............a.
Rhesus     G............................T.....a.
Baboon     G............A...............T.....a.
Marmoset   G.......A..A.......A............A..a.
Mouse      G.....CA...A.C..CCA...CT.T......TT.cc
```

---

## 3. uc010pkz.1\_1\_11

**Summary**  

|  |  |  |  |  |  |  |  |  |  |  |  |  |  |  |  |  |  |  |  |  |  |  |  |  |  |
| --- | --- | --- | --- | --- | --- | --- | --- | --- | --- | --- | --- | --- | --- | --- | --- | --- | --- | --- | --- | --- | --- | --- | --- | --- | --- |
| No Exon ID Position (hg19) Dir Human donor Chimp donor Category Usage Gene symbol Protein accession mRNA accession Gene title Note|  |  |  |  |  |  |  |  |  |  |  |  |  | | --- | --- | --- | --- | --- | --- | --- | --- | --- | --- | --- | --- | --- | | 3 uc010pkz.1\_1\_11 chr1:165667645 - GT GC (D3) shift; decrease; inframe alternative ALDH9A1 B4DX14 AK293520.1 aldehyde dehydrogenase 9 family, member A1  | | | | | | | | | | | | | | | | | | | | | | | | | |

**Orthologs**  

|  |  |  |  |  |  |  |  |  |  |  |  |  |  |  |  |  |  |  |  |  |  |  |  |  |  |  |  |  |  |  |  |  |  |  |  |  |  |  |  |  |  |  |  |  |  |  |  |  |  |  |  |  |  |  |  |  |  |  |  |  |  |  |  |  |  |  |  |  |  |  |  |
| --- | --- | --- | --- | --- | --- | --- | --- | --- | --- | --- | --- | --- | --- | --- | --- | --- | --- | --- | --- | --- | --- | --- | --- | --- | --- | --- | --- | --- | --- | --- | --- | --- | --- | --- | --- | --- | --- | --- | --- | --- | --- | --- | --- | --- | --- | --- | --- | --- | --- | --- | --- | --- | --- | --- | --- | --- | --- | --- | --- | --- | --- | --- | --- | --- | --- | --- | --- | --- | --- | --- | --- |
| Species Assembly Chromosome Exon start Exon end Dir Donor Exon sequence|  |  |  |  |  |  |  |  |  |  |  |  |  |  |  |  |  |  |  |  |  |  |  |  |  |  |  |  |  |  |  |  |  |  |  |  |  |  |  |  |  |  |  |  |  |  |  |  |  |  |  |  |  |  |  |  |  |  |  |  |  |  |  |  | | --- | --- | --- | --- | --- | --- | --- | --- | --- | --- | --- | --- | --- | --- | --- | --- | --- | --- | --- | --- | --- | --- | --- | --- | --- | --- | --- | --- | --- | --- | --- | --- | --- | --- | --- | --- | --- | --- | --- | --- | --- | --- | --- | --- | --- | --- | --- | --- | --- | --- | --- | --- | --- | --- | --- | --- | --- | --- | --- | --- | --- | --- | --- | --- | | Human hg19 chr1 165667645 165667795 - **GT** | ATGTTTCTCCGAGCAGGCCTGGCCGCGCTCTCCCCGCTTCTTCGCAGTCTTCGGCCCTCTCCTGTCGCCGCCATGAGCACTGGCACCTTCGTCGTGTCGCAGCCGCTCAATTACCGCGGCGGGGCCCGCGTGGAGCCGGCGGACGCCTCCG|  |  |  |  |  |  |  |  |  |  |  |  |  |  |  |  |  |  |  |  |  |  |  |  |  |  |  |  |  |  |  |  |  |  |  |  |  |  |  |  |  |  |  |  |  |  |  |  |  |  |  |  |  |  |  |  | | --- | --- | --- | --- | --- | --- | --- | --- | --- | --- | --- | --- | --- | --- | --- | --- | --- | --- | --- | --- | --- | --- | --- | --- | --- | --- | --- | --- | --- | --- | --- | --- | --- | --- | --- | --- | --- | --- | --- | --- | --- | --- | --- | --- | --- | --- | --- | --- | --- | --- | --- | --- | --- | --- | --- | --- | | Chimp panTro2 chr1 145030074 145030224 - **GC** | ATGTTTCTCCGAGCAGGCCTGGCCGCGCTCTCCCCGCTTCTTCGCAGTCTTCGGCCCTCTCCTGTCGCCGCCATGAGCACTGGCAACTTCGTCGTGTCGCAGCCGCTCAATTACCGCGGCGGGGCCCGCGTGGAGCCGGCGGACGCCTCCG|  |  |  |  |  |  |  |  |  |  |  |  |  |  |  |  |  |  |  |  |  |  |  |  |  |  |  |  |  |  |  |  |  |  |  |  |  |  |  |  |  |  |  |  |  |  |  |  | | --- | --- | --- | --- | --- | --- | --- | --- | --- | --- | --- | --- | --- | --- | --- | --- | --- | --- | --- | --- | --- | --- | --- | --- | --- | --- | --- | --- | --- | --- | --- | --- | --- | --- | --- | --- | --- | --- | --- | --- | --- | --- | --- | --- | --- | --- | --- | --- | | Gorilla gorGor1 Supercontig\_0208017 271 421 - **GC** | ATGTTTCTCGGAGCAGGCCTGGCCGCGCTCTCCCCGCTTCTTCGCAGTCTTCGGCCCTCTTCTGTCGCCGCCATGAGCACTGGCACCTTCGTCGTGTCGCAGCCGCTCAATTACCGCGGCGGGGCCCGCGTGGAGCCGGCGGACGCCTCCG|  |  |  |  |  |  |  |  |  |  |  |  |  |  |  |  |  |  |  |  |  |  |  |  |  |  |  |  |  |  |  |  |  |  |  |  |  |  |  |  | | --- | --- | --- | --- | --- | --- | --- | --- | --- | --- | --- | --- | --- | --- | --- | --- | --- | --- | --- | --- | --- | --- | --- | --- | --- | --- | --- | --- | --- | --- | --- | --- | --- | --- | --- | --- | --- | --- | --- | --- | | Orangutan ponAbe2 chr1 85547429 85547579 + **GC** | ATGTTTCTCCGAGCAGGCCTGGCCGCGCTCTTCCCGCTTCTTCGCAGTCTTCGGCCCTCTTCTGTCGCCGCCATGAGCACTGGCACCTTCGTCGTGTCGCAGCCGCTCAATTACCGCGGCGGGGCCCGCGTGGAGCCGGCGGACGCCTCCG|  |  |  |  |  |  |  |  |  |  |  |  |  |  |  |  |  |  |  |  |  |  |  |  |  |  |  |  |  |  |  |  | | --- | --- | --- | --- | --- | --- | --- | --- | --- | --- | --- | --- | --- | --- | --- | --- | --- | --- | --- | --- | --- | --- | --- | --- | --- | --- | --- | --- | --- | --- | --- | --- | | Rhesus rheMac2 chr1 195544970 195545120 - **GC** | ATGTTTCTCCGAGCAGGCCGGGCCGCGCTCTCTCCGCTTGTTCGCAGTCTTCAGCCCTCTCCTGTCGCCGCCATGAGCACTGGCACCTTCGTCGTGTCGCAGCCGCTCAATTACCGCGGCGGGGCCCGCGTGGAGCCGGCTGACGCCTCCG|  |  |  |  |  |  |  |  |  |  |  |  |  |  |  |  |  |  |  |  |  |  |  |  | | --- | --- | --- | --- | --- | --- | --- | --- | --- | --- | --- | --- | --- | --- | --- | --- | --- | --- | --- | --- | --- | --- | --- | --- | | Baboon papHam1 scaffold44409 316 466 + **GC** | ATGTTTCTCCGAGCAGGCCTGGCCGCGCTTTCTCCGCTTGTTCGCAGTCTTCAGCCCTCTCCTGTCGCCGCCATGAGTACTGGCACGTTCGTCGTGTCGCAGCCGCTCAATTACCGCGGCGGGGCCCGCGTGGAGCCGGCTGACGCCTCCG|  |  |  |  |  |  |  |  |  |  |  |  |  |  |  |  | | --- | --- | --- | --- | --- | --- | --- | --- | --- | --- | --- | --- | --- | --- | --- | --- | | Marmoset calJac1 Contig2324 360785 360935 - **GC** | ATGTTTTTCCGAGCGGGCCGGGCTGCGGTTTCTTCGTTTCTTCGCAGTGTTCTGCAGTCTCCTGTGGCCACCATGAGCACCGGCACCTTCGTTGTGTCGCAGCCGCTTAATTACCGTGGCGGGACCCGCGTGGAGCCGGCGGACGCCTCTG|  |  |  |  |  |  |  |  | | --- | --- | --- | --- | --- | --- | --- | --- | | Mouse mm9 chr1 169280367 169280517 + **GC** | ATGATCCTCGGTGCAGTCGGTTCTGTGCTGACCTCGCTCCTCCGCATTCACCGGGCTGCAGCAGTCGCCGCCATGAGTACTGGCACCTTCGTCGTGTCGCAGCCGCTCAACTACCGCGGCGGGGCCCGTGTGGAGCCGGTGGATGCTTCCG | | | | | | | | | | | | | | | | | | | | | | | | | | | | | | | | | | | | | | | | | | | | | | | | | | | | | | | | | | | | | | | |

**Alignment** (splice site sequences are in lowercase)  

```
Human      ATGTTTCTCCGAGCAGGCCTGGCCGCGCTCTCCCCGCTTCTTCGCAGTCTTCGGCCCTCTCCTGTCGCCGCCATGAGCAC
Chimp      ................................................................................
Gorilla    .........G..................................................T...................
Orangutan  ...............................T............................T...................
Rhesus     ...................G............T......G............A...........................
Baboon     .............................T..T......G............A........................T..
Marmoset   ......T.......G....G...T...G.T..TT..T...........G...T..AG........G...A..........
Mouse      ...A.C...G.T....T.GGTT.T.T...GA..T....C..C....T..AC...G.TG.AG.A..............T..

Human      TGGCACCTTCGTCGTGTCGCAGCCGCTCAATTACCGCGGCGGGGCCCGCGTGGAGCCGGCGGACGCCTCCGgt
Chimp      .....A..................................................................c
Gorilla    ........................................................................c
Orangutan  ........................................................................c
Rhesus     ............................................................T...........c
Baboon     ......G.....................................................T...........c
Marmoset   C...........T..............T........T......A.........................T..c
Mouse      ..............................C.................T..........T...T..T.....c
```

---

## 4. uc010pxj.1\_9\_10

**Summary**  

|  |  |  |  |  |  |  |  |  |  |  |  |  |  |  |  |  |  |  |  |  |  |  |  |  |  |
| --- | --- | --- | --- | --- | --- | --- | --- | --- | --- | --- | --- | --- | --- | --- | --- | --- | --- | --- | --- | --- | --- | --- | --- | --- | --- |
| No Exon ID Position (hg19) Dir Human donor Chimp donor Category Usage Gene symbol Protein accession mRNA accession Gene title Note|  |  |  |  |  |  |  |  |  |  |  |  |  | | --- | --- | --- | --- | --- | --- | --- | --- | --- | --- | --- | --- | --- | | 4 uc010pxj.1\_9\_10 chr1:232144729 + GT GC (D3) shift; decrease; inframe alternative DISC1 NP\_001012975.1 NM\_001012957.1 disrupted in schizophrenia 1 protein  | | | | | | | | | | | | | | | | | | | | | | | | | |

**Orthologs**  

|  |  |  |  |  |  |  |  |  |  |  |  |  |  |  |  |  |  |  |  |  |  |  |  |  |  |  |  |  |  |  |  |  |  |  |  |  |  |  |  |  |  |  |  |  |  |  |  |  |  |  |  |  |  |  |  |  |  |  |  |  |  |  |  |  |  |  |  |  |  |  |  |  |  |  |  |  |  |  |  |  |  |  |  |  |  |  |  |  |  |  |  |  |  |  |  |  |  |  |  |  |  |  |  |
| --- | --- | --- | --- | --- | --- | --- | --- | --- | --- | --- | --- | --- | --- | --- | --- | --- | --- | --- | --- | --- | --- | --- | --- | --- | --- | --- | --- | --- | --- | --- | --- | --- | --- | --- | --- | --- | --- | --- | --- | --- | --- | --- | --- | --- | --- | --- | --- | --- | --- | --- | --- | --- | --- | --- | --- | --- | --- | --- | --- | --- | --- | --- | --- | --- | --- | --- | --- | --- | --- | --- | --- | --- | --- | --- | --- | --- | --- | --- | --- | --- | --- | --- | --- | --- | --- | --- | --- | --- | --- | --- | --- | --- | --- | --- | --- | --- | --- | --- | --- | --- | --- | --- | --- |
| Species Assembly Chromosome Exon start Exon end Dir Donor Exon sequence|  |  |  |  |  |  |  |  |  |  |  |  |  |  |  |  |  |  |  |  |  |  |  |  |  |  |  |  |  |  |  |  |  |  |  |  |  |  |  |  |  |  |  |  |  |  |  |  |  |  |  |  |  |  |  |  |  |  |  |  |  |  |  |  |  |  |  |  |  |  |  |  |  |  |  |  |  |  |  |  |  |  |  |  |  |  |  |  |  |  |  |  |  |  |  |  | | --- | --- | --- | --- | --- | --- | --- | --- | --- | --- | --- | --- | --- | --- | --- | --- | --- | --- | --- | --- | --- | --- | --- | --- | --- | --- | --- | --- | --- | --- | --- | --- | --- | --- | --- | --- | --- | --- | --- | --- | --- | --- | --- | --- | --- | --- | --- | --- | --- | --- | --- | --- | --- | --- | --- | --- | --- | --- | --- | --- | --- | --- | --- | --- | --- | --- | --- | --- | --- | --- | --- | --- | --- | --- | --- | --- | --- | --- | --- | --- | --- | --- | --- | --- | --- | --- | --- | --- | --- | --- | --- | --- | --- | --- | --- | --- | | Human hg19 chr1 232144531 232144729 + **GT** | CTGCAAGTGTCCACTGCTTGGGAAAGTGTGGGAAGCTGACTTGGAAGCTTGTCGATTGCTTATCCAGAGCCTACAGCTCCAGGAAGCCAGGGGAAGCCTGTCTGTAGAAGATGAGAGGCAGATGGATGACTTAGAGGGAGCTGCTCCTCCTATTCCCCCCAGGCTCCACTCCGAGGATAAAAGGAAGACCCCTTTGAAG|  |  |  |  |  |  |  |  |  |  |  |  |  |  |  |  |  |  |  |  |  |  |  |  |  |  |  |  |  |  |  |  |  |  |  |  |  |  |  |  |  |  |  |  |  |  |  |  |  |  |  |  |  |  |  |  |  |  |  |  |  |  |  |  |  |  |  |  |  |  |  |  |  |  |  |  |  |  |  |  |  |  |  |  |  |  |  |  | | --- | --- | --- | --- | --- | --- | --- | --- | --- | --- | --- | --- | --- | --- | --- | --- | --- | --- | --- | --- | --- | --- | --- | --- | --- | --- | --- | --- | --- | --- | --- | --- | --- | --- | --- | --- | --- | --- | --- | --- | --- | --- | --- | --- | --- | --- | --- | --- | --- | --- | --- | --- | --- | --- | --- | --- | --- | --- | --- | --- | --- | --- | --- | --- | --- | --- | --- | --- | --- | --- | --- | --- | --- | --- | --- | --- | --- | --- | --- | --- | --- | --- | --- | --- | --- | --- | --- | --- | | Chimp panTro2 chr1 212644817 212645027 + **GC** | CTGCAAGTGTCCACTGCTCGGGAAAGTGTGGGAAGCTGACTTGGAAGCTTGTCGATTGCTTATCCAGAGCCTCCAACTCCAGGAAGCCAGGGGAAGCCTGTCTGTAGAAGATGAGAGGCAGATGGATGACTTAGAGGGAGCTGTCTGTATAGCTGCTCCTCCTATTCCCCCCAGGCTCCACTCCGAGGATAAAAGGAAGACCCCTTTGCAG|  |  |  |  |  |  |  |  |  |  |  |  |  |  |  |  |  |  |  |  |  |  |  |  |  |  |  |  |  |  |  |  |  |  |  |  |  |  |  |  |  |  |  |  |  |  |  |  |  |  |  |  |  |  |  |  |  |  |  |  |  |  |  |  |  |  |  |  |  |  |  |  |  |  |  |  |  |  |  |  | | --- | --- | --- | --- | --- | --- | --- | --- | --- | --- | --- | --- | --- | --- | --- | --- | --- | --- | --- | --- | --- | --- | --- | --- | --- | --- | --- | --- | --- | --- | --- | --- | --- | --- | --- | --- | --- | --- | --- | --- | --- | --- | --- | --- | --- | --- | --- | --- | --- | --- | --- | --- | --- | --- | --- | --- | --- | --- | --- | --- | --- | --- | --- | --- | --- | --- | --- | --- | --- | --- | --- | --- | --- | --- | --- | --- | --- | --- | --- | --- | | Orangutan ponAbe2 chr1 17691423 17691633 - **GC** | CTGCAAGTGTCCACTGCTTGGGAAAGTGTGGGAAGCTGACTTGGAAGCTTGTCAATTGCTTATCCAGAGCCTACAACTCCAGGAAGCCAGGGGAAGCCTGTCTGTAGAAGATGAGAGGCAGATGGATGACTTAGAGGGAGCTGCCTGTACAGCTGCTCCTCCCTTTCCCCCTAGGCTCCACTCCGAGGATAAAAGGAAGACCCCTTTGCAG|  |  |  |  |  |  |  |  |  |  |  |  |  |  |  |  |  |  |  |  |  |  |  |  |  |  |  |  |  |  |  |  |  |  |  |  |  |  |  |  |  |  |  |  |  |  |  |  |  |  |  |  |  |  |  |  |  |  |  |  |  |  |  |  |  |  |  |  |  |  |  |  | | --- | --- | --- | --- | --- | --- | --- | --- | --- | --- | --- | --- | --- | --- | --- | --- | --- | --- | --- | --- | --- | --- | --- | --- | --- | --- | --- | --- | --- | --- | --- | --- | --- | --- | --- | --- | --- | --- | --- | --- | --- | --- | --- | --- | --- | --- | --- | --- | --- | --- | --- | --- | --- | --- | --- | --- | --- | --- | --- | --- | --- | --- | --- | --- | --- | --- | --- | --- | --- | --- | --- | --- | | Rhesus rheMac2 chr1 224948263 224948473 - **GC** | CTGCAAGTGTCCACTGCTTGGGAAAGTGTGGGAAGCTGACTTGGAAGCTTGTCGATTGCTTATGCAGAGCCTACAACTCCAGGAAGCCAGGGGAAGCCTGTCTGTAGAAGATGAGAGGCAGATGGATGCCTTAGAGGGAGCTGTTTGTATAGCTGCTTCTCCCATTACCCCCAGGCTCCACTCCGAGGATAAAAGGAAGACCCCTTTGCAG|  |  |  |  |  |  |  |  |  |  |  |  |  |  |  |  |  |  |  |  |  |  |  |  |  |  |  |  |  |  |  |  |  |  |  |  |  |  |  |  |  |  |  |  |  |  |  |  |  |  |  |  |  |  |  |  |  |  |  |  |  |  |  |  | | --- | --- | --- | --- | --- | --- | --- | --- | --- | --- | --- | --- | --- | --- | --- | --- | --- | --- | --- | --- | --- | --- | --- | --- | --- | --- | --- | --- | --- | --- | --- | --- | --- | --- | --- | --- | --- | --- | --- | --- | --- | --- | --- | --- | --- | --- | --- | --- | --- | --- | --- | --- | --- | --- | --- | --- | --- | --- | --- | --- | --- | --- | --- | --- | | Baboon papHam1 scaffold3058 89674 89884 + **GC** | CTGCAAGTGTCCACTGCTTGGGAAAGTGTGGGAAGCTGACTTGGAAGCTTGTCGATTGCTTATCCAGAGCCTACAACTCCAGGAAGCCAGGGGAAGCCTGTCTGTAGAAGATGAGAGGCAGATGGATGCCTTAGAGGGAGCTGTTTGTATAGCTGCTCCTCCCATTCCCCCCAGGCTCCACTCCGAGGATAAAAGGAAGACCCCTTTGCAG|  |  |  |  |  |  |  |  |  |  |  |  |  |  |  |  |  |  |  |  |  |  |  |  |  |  |  |  |  |  |  |  |  |  |  |  |  |  |  |  |  |  |  |  |  |  |  |  |  |  |  |  |  |  |  |  | | --- | --- | --- | --- | --- | --- | --- | --- | --- | --- | --- | --- | --- | --- | --- | --- | --- | --- | --- | --- | --- | --- | --- | --- | --- | --- | --- | --- | --- | --- | --- | --- | --- | --- | --- | --- | --- | --- | --- | --- | --- | --- | --- | --- | --- | --- | --- | --- | --- | --- | --- | --- | --- | --- | --- | --- | | Marmoset calJac1 Contig1169 330206 330416 - **GC** | CTGCAAGTGTCCACTGCTTGGAAAAGTGTGGGAAGCTGACTTGGAAGCTTGTCGATTGCTTATCCAGAGGCTACAACTCCATGAAGCCAAGGGCAGCCTGTCTGTAGAAGATGAGGGGCAGATGGATGACCTAGTGGGAGCTGTCTGTTCAGCGGCTCCTCCCATTCCCCCTAGGCTCCACTCTGAGGATGAGAGGAAGACCCCTTTGCAG|  |  |  |  |  |  |  |  |  |  |  |  |  |  |  |  |  |  |  |  |  |  |  |  |  |  |  |  |  |  |  |  |  |  |  |  |  |  |  |  |  |  |  |  |  |  |  |  | | --- | --- | --- | --- | --- | --- | --- | --- | --- | --- | --- | --- | --- | --- | --- | --- | --- | --- | --- | --- | --- | --- | --- | --- | --- | --- | --- | --- | --- | --- | --- | --- | --- | --- | --- | --- | --- | --- | --- | --- | --- | --- | --- | --- | --- | --- | --- | --- | | Tarsier tarSyr1 scaffold\_184886 205 415 - **GC** | CTGCAAGTATCCACTGCTTGGAAAAGTGTGGGAAGCTGACTTGGAAGCTTGCAGGTTGCTTATTCAGAGCCTGCAACTCCGGGAAGCCAGGGGCAGTCTGTCTGGCGAAGACCAGAGGCAGATGAATGACACAGAGGGAGCTGCCTGTACCACCACTCCAGCCATGCCCCTCAGGCTGCAGTCCCATGATGAGAGGAAGACTCATTTGCAG|  |  |  |  |  |  |  |  |  |  |  |  |  |  |  |  |  |  |  |  |  |  |  |  |  |  |  |  |  |  |  |  |  |  |  |  |  |  |  |  | | --- | --- | --- | --- | --- | --- | --- | --- | --- | --- | --- | --- | --- | --- | --- | --- | --- | --- | --- | --- | --- | --- | --- | --- | --- | --- | --- | --- | --- | --- | --- | --- | --- | --- | --- | --- | --- | --- | --- | --- | | Lemur micMur1 scaffold\_387 591459 591669 + **GC** | CTGCACGTGTCCTCTGCTTGGGAAAGTGTGGGAAGCTGACTTGGAAGCTTGTCGGTTGCTTATCCAGAGCCTGCAACTCCAGGAGGCCAGGGGCAGCCTGTCTGTGGAAGATGAGAGGCAGGTGGAGGACATGGAGGGAGCCATCTGTGCAGCCACTGCAGCCGTCCACCCCAGGCCCCACTTCGGAGACGAGAGGAAGACCCCTTTGCAG|  |  |  |  |  |  |  |  |  |  |  |  |  |  |  |  |  |  |  |  |  |  |  |  |  |  |  |  |  |  |  |  | | --- | --- | --- | --- | --- | --- | --- | --- | --- | --- | --- | --- | --- | --- | --- | --- | --- | --- | --- | --- | --- | --- | --- | --- | --- | --- | --- | --- | --- | --- | --- | --- | | Galago otoGar1 scaffold\_109877.1-179029 79331 79541 - **GC** | CTGCAAATGTTTGTTGCTTGGGAAAGTGTGGGAAGCAGACCTGGAAGCTTGTCGGTTGCTTATCCAGAGCCTGAACCTCCAGGAAGCCAGGGGCAGCCTGTCTGCAGAAGATGAGAGGCAGATGGATGACTTAGAGGGAGCCGTCTGTACAGCCTCTCTGGCCCTCCACCCCACACCCCACTCTGAAGATGAGACGAAGACTCCTTTGCAG|  |  |  |  |  |  |  |  |  |  |  |  |  |  |  |  |  |  |  |  |  |  |  |  | | --- | --- | --- | --- | --- | --- | --- | --- | --- | --- | --- | --- | --- | --- | --- | --- | --- | --- | --- | --- | --- | --- | --- | --- | | Mouse mm9 chr8 127756684 127756891 + **GT** | CTGCAGGTGCCCGCTGCTTGGGAGAGTGTGGAAAGCAGACTTGGAGACTTGTCAGTTGCTAATGCAGAGCCTGCAGCTTCAGGAAGCAGGCAGCAGCCCACACGCAGAGGACGAGGAGCAGGTGCATAGCACAGGAGAGGCCGCCCAGACAGCTGCTCTGGCTGTCCCTCGAACACCCCACCCCGAAGAAGAAAAGTCCCCCTTGCAG|  |  |  |  |  |  |  |  |  |  |  |  |  |  |  |  | | --- | --- | --- | --- | --- | --- | --- | --- | --- | --- | --- | --- | --- | --- | --- | --- | | Cow bosTau4 chr28 2843477 2843684 + **GC** | CTGCAAGTGTCCGCTGCTTGGAAAAGTATGGGAAGCTGACTTGGAAGCTTGTCGGTTGCTTATCCAGAGCTTGCAGCTCCAGGAGGCCAGGGGCAGCCTGTTTGCAGATGAGAGACAGACGGATGACTTAGGGGGGGGTACTTATACAATGGCTCTGGCCACCCCCCTGAGGTCCCACCTTGAAGATGGAAGGAAGAACCCTTTGCAA|  |  |  |  |  |  |  |  | | --- | --- | --- | --- | --- | --- | --- | --- | | Dog canFam2 chr4 10553203 10553410 - **GC** | CTGCAAGTGTCCACTGCTTGGAAAAGTGTGGGAAGCTGACTTGGAAGCTTGTCAGTTGCTTATCCAGGGCCTACAGCTTCAGGCCAGGGGCATACTATCTGCAGAAGATGAGAGGCAGACAGATGACTTAGGGGGAGCTGCCTGTACATCCACTCCTGCCATCTCCCCCAGGCTCCACTCTGAAGTTGAGAAGAAGACCCCTTTGCAA | | | | | | | | | | | | | | | | | | | | | | | | | | | | | | | | | | | | | | | | | | | | | | | | | | | | | | | | | | | | | | | | | | | | | | | | | | | | | | | | | | | | | | | | | | | |

**Alignment** (splice site sequences are in lowercase)  

```
Human      agCTGCAAGTGTCCACTGCTTGGGAAAGTGTGGGAAGCTGACTTGGAAGCTTGTCGATTGCTTATCCAGAGCCTACAGCT
Chimp      ....................C.....................................................C..A..
Orangutan  .......................................................A.....................A..
Rhesus     .................................................................G...........A..
Baboon     .............................................................................A..
Marmoset   .......................A...............................................G.....A..
Tarsier    ..........A............A.............................CA.G........T........G..A..
Lemur      .......C......T.........................................G.................G..A..
Galago     ........A...TTGT......................A...C.............G.................GA.C..
Mouse      .......G...C..G..........G.......A....A........GA......AG.....A..G........G.....
Cow        ..............G........A.....A..........................G...............T.G.....
Dog        .......................A...............................AG............G..........

Human      CCAGGAAGCCAGGGGAAGCCTGTCTGTAGAAGATGAGAGGCAGATGGATGACTTAGAGGGAGCTG------------CTC
Chimp      .................................................................TCTGTATAGCTG...
Orangutan  .................................................................CCTGTACAGCTG...
Rhesus     ..................................................C..............TTTGTATAGCTG..T
Baboon     ..................................................C..............TTTGTATAGCTG...
Marmoset   ...T.......A...C.....................G..............C...T........TCTGTTCAGCGG...
Tarsier    ..G............C..T.......GC.....CC...........A.....AC...........CCTGTACCACCA...
Lemur      ......G........C...........G...............G....G...A.G........CATCTGTGCAGCCA..G
Galago     ...............C..........C....................................C.TCTGTACAGCCT...
Mouse      T........AG.CA.C....CACAC.C...G..C...GA....G..C..AG.AC..GA.AG..C.CCCAGACAGCTG...
Cow        ......G........C.......T..C---.........A....C...........G...G.G.ACTTATACAATGG...
Dog        T...---........C.TA..A....C.................CA..........G........CCTGTACATCCA...

Human      CTCCTATTCCCCCCAGGCTCCACTCCGAGGATAAAAGGAAGACCCCTTTGAAGgt
Chimp      ..................................................C...c
Orangutan  ....CT.......T....................................C...c
Rhesus     ....C...A.........................................C...c
Baboon     ....C.............................................C...c
Marmoset   ....C........T...........T......G.G...............C...c
Tarsier    .AG.C..G....T......G..G...C.T...G.G........T.A....C...c
Lemur      .AG.CG.C.A........C.....T..GA..CG.G...............C...c
Galago     TGG.CC.C.A.....CA.C......T..A...G.G.C......T......C...c
Mouse      TGG..G.C..T.GA.CA.C....C....A..AG..---...T....C...C....
Cow        TGG.C.CC....TG...TC....CTT..A...GG........A.......C.A.c
Dog        ..G.C..CT................T..A.T.G.G.A.............C.A.c
```

---

## 5. uc010pyf.1\_1\_9

**Summary**  

|  |  |  |  |  |  |  |  |  |  |  |  |  |  |  |  |  |  |  |  |  |  |  |  |  |  |
| --- | --- | --- | --- | --- | --- | --- | --- | --- | --- | --- | --- | --- | --- | --- | --- | --- | --- | --- | --- | --- | --- | --- | --- | --- | --- |
| No Exon ID Position (hg19) Dir Human donor Chimp donor Category Usage Gene symbol Protein accession mRNA accession Gene title Note|  |  |  |  |  |  |  |  |  |  |  |  |  | | --- | --- | --- | --- | --- | --- | --- | --- | --- | --- | --- | --- | --- | | 5 uc010pyf.1\_1\_9 chr1:240408865 + GT AT (D7) exonization; novel start alternative FMN2 B7Z4S3 AK297755.1 formin 2  | | | | | | | | | | | | | | | | | | | | | | | | | |

**Orthologs**  

|  |  |  |  |  |  |  |  |  |  |  |  |  |  |  |  |  |  |  |  |  |  |  |  |  |  |  |  |  |  |  |  |  |  |  |  |  |  |  |  |  |  |  |  |  |  |  |  |  |  |  |  |  |  |  |  |  |  |  |  |  |  |  |  |  |  |  |  |  |  |  |  |  |  |  |  |  |  |  |  |  |  |  |  |  |  |  |  |  |  |  |  |  |  |  |  |
| --- | --- | --- | --- | --- | --- | --- | --- | --- | --- | --- | --- | --- | --- | --- | --- | --- | --- | --- | --- | --- | --- | --- | --- | --- | --- | --- | --- | --- | --- | --- | --- | --- | --- | --- | --- | --- | --- | --- | --- | --- | --- | --- | --- | --- | --- | --- | --- | --- | --- | --- | --- | --- | --- | --- | --- | --- | --- | --- | --- | --- | --- | --- | --- | --- | --- | --- | --- | --- | --- | --- | --- | --- | --- | --- | --- | --- | --- | --- | --- | --- | --- | --- | --- | --- | --- | --- | --- | --- | --- | --- | --- | --- | --- | --- | --- |
| Species Assembly Chromosome Exon start Exon end Dir Donor Exon sequence|  |  |  |  |  |  |  |  |  |  |  |  |  |  |  |  |  |  |  |  |  |  |  |  |  |  |  |  |  |  |  |  |  |  |  |  |  |  |  |  |  |  |  |  |  |  |  |  |  |  |  |  |  |  |  |  |  |  |  |  |  |  |  |  |  |  |  |  |  |  |  |  |  |  |  |  |  |  |  |  |  |  |  |  |  |  |  |  | | --- | --- | --- | --- | --- | --- | --- | --- | --- | --- | --- | --- | --- | --- | --- | --- | --- | --- | --- | --- | --- | --- | --- | --- | --- | --- | --- | --- | --- | --- | --- | --- | --- | --- | --- | --- | --- | --- | --- | --- | --- | --- | --- | --- | --- | --- | --- | --- | --- | --- | --- | --- | --- | --- | --- | --- | --- | --- | --- | --- | --- | --- | --- | --- | --- | --- | --- | --- | --- | --- | --- | --- | --- | --- | --- | --- | --- | --- | --- | --- | --- | --- | --- | --- | --- | --- | --- | --- | | Human hg19 chr1 240408863 240408865 + **GT** | ATG|  |  |  |  |  |  |  |  |  |  |  |  |  |  |  |  |  |  |  |  |  |  |  |  |  |  |  |  |  |  |  |  |  |  |  |  |  |  |  |  |  |  |  |  |  |  |  |  |  |  |  |  |  |  |  |  |  |  |  |  |  |  |  |  |  |  |  |  |  |  |  |  |  |  |  |  |  |  |  |  | | --- | --- | --- | --- | --- | --- | --- | --- | --- | --- | --- | --- | --- | --- | --- | --- | --- | --- | --- | --- | --- | --- | --- | --- | --- | --- | --- | --- | --- | --- | --- | --- | --- | --- | --- | --- | --- | --- | --- | --- | --- | --- | --- | --- | --- | --- | --- | --- | --- | --- | --- | --- | --- | --- | --- | --- | --- | --- | --- | --- | --- | --- | --- | --- | --- | --- | --- | --- | --- | --- | --- | --- | --- | --- | --- | --- | --- | --- | --- | --- | | Chimp panTro2 chr1 221136708 221136710 + **AT** | ATG|  |  |  |  |  |  |  |  |  |  |  |  |  |  |  |  |  |  |  |  |  |  |  |  |  |  |  |  |  |  |  |  |  |  |  |  |  |  |  |  |  |  |  |  |  |  |  |  |  |  |  |  |  |  |  |  |  |  |  |  |  |  |  |  |  |  |  |  |  |  |  |  | | --- | --- | --- | --- | --- | --- | --- | --- | --- | --- | --- | --- | --- | --- | --- | --- | --- | --- | --- | --- | --- | --- | --- | --- | --- | --- | --- | --- | --- | --- | --- | --- | --- | --- | --- | --- | --- | --- | --- | --- | --- | --- | --- | --- | --- | --- | --- | --- | --- | --- | --- | --- | --- | --- | --- | --- | --- | --- | --- | --- | --- | --- | --- | --- | --- | --- | --- | --- | --- | --- | --- | --- | | Gorilla gorGor1 Supercontig\_0003929 6639 6641 + **AT** | ATG|  |  |  |  |  |  |  |  |  |  |  |  |  |  |  |  |  |  |  |  |  |  |  |  |  |  |  |  |  |  |  |  |  |  |  |  |  |  |  |  |  |  |  |  |  |  |  |  |  |  |  |  |  |  |  |  |  |  |  |  |  |  |  |  | | --- | --- | --- | --- | --- | --- | --- | --- | --- | --- | --- | --- | --- | --- | --- | --- | --- | --- | --- | --- | --- | --- | --- | --- | --- | --- | --- | --- | --- | --- | --- | --- | --- | --- | --- | --- | --- | --- | --- | --- | --- | --- | --- | --- | --- | --- | --- | --- | --- | --- | --- | --- | --- | --- | --- | --- | --- | --- | --- | --- | --- | --- | --- | --- | | Orangutan ponAbe2 chr1 9091502 9091504 - **AT** | ATG|  |  |  |  |  |  |  |  |  |  |  |  |  |  |  |  |  |  |  |  |  |  |  |  |  |  |  |  |  |  |  |  |  |  |  |  |  |  |  |  |  |  |  |  |  |  |  |  |  |  |  |  |  |  |  |  | | --- | --- | --- | --- | --- | --- | --- | --- | --- | --- | --- | --- | --- | --- | --- | --- | --- | --- | --- | --- | --- | --- | --- | --- | --- | --- | --- | --- | --- | --- | --- | --- | --- | --- | --- | --- | --- | --- | --- | --- | --- | --- | --- | --- | --- | --- | --- | --- | --- | --- | --- | --- | --- | --- | --- | --- | | Rhesus rheMac2 chr1 216798381 216798383 - **AT** | ATG|  |  |  |  |  |  |  |  |  |  |  |  |  |  |  |  |  |  |  |  |  |  |  |  |  |  |  |  |  |  |  |  |  |  |  |  |  |  |  |  |  |  |  |  |  |  |  |  | | --- | --- | --- | --- | --- | --- | --- | --- | --- | --- | --- | --- | --- | --- | --- | --- | --- | --- | --- | --- | --- | --- | --- | --- | --- | --- | --- | --- | --- | --- | --- | --- | --- | --- | --- | --- | --- | --- | --- | --- | --- | --- | --- | --- | --- | --- | --- | --- | | Baboon papHam1 scaffold28386 3802 3804 - **AT** | ATG|  |  |  |  |  |  |  |  |  |  |  |  |  |  |  |  |  |  |  |  |  |  |  |  |  |  |  |  |  |  |  |  |  |  |  |  |  |  |  |  | | --- | --- | --- | --- | --- | --- | --- | --- | --- | --- | --- | --- | --- | --- | --- | --- | --- | --- | --- | --- | --- | --- | --- | --- | --- | --- | --- | --- | --- | --- | --- | --- | --- | --- | --- | --- | --- | --- | --- | --- | | Marmoset calJac1 Contig5724 54384 54386 - **AT** | ATG|  |  |  |  |  |  |  |  |  |  |  |  |  |  |  |  |  |  |  |  |  |  |  |  |  |  |  |  |  |  |  |  | | --- | --- | --- | --- | --- | --- | --- | --- | --- | --- | --- | --- | --- | --- | --- | --- | --- | --- | --- | --- | --- | --- | --- | --- | --- | --- | --- | --- | --- | --- | --- | --- | | Lemur micMur1 scaffold\_4942 480 482 + **AG** | ACA|  |  |  |  |  |  |  |  |  |  |  |  |  |  |  |  |  |  |  |  |  |  |  |  | | --- | --- | --- | --- | --- | --- | --- | --- | --- | --- | --- | --- | --- | --- | --- | --- | --- | --- | --- | --- | --- | --- | --- | --- | | Galago otoGar1 scaffold\_114297.1-93085 42664 42666 + **AG** | ATA|  |  |  |  |  |  |  |  |  |  |  |  |  |  |  |  | | --- | --- | --- | --- | --- | --- | --- | --- | --- | --- | --- | --- | --- | --- | --- | --- | | Mouse mm9 chr1 176571332 176571334 + **GT** | ATG|  |  |  |  |  |  |  |  | | --- | --- | --- | --- | --- | --- | --- | --- | | Dog canFam2 chr7 34784602 34784604 + **AT** | AAG | | | | | | | | | | | | | | | | | | | | | | | | | | | | | | | | | | | | | | | | | | | | | | | | | | | | | | | | | | | | | | | | | | | | | | | | | | | | | | | | | | | | |

**Alignment** (splice site sequences are in lowercase)  

```
Human      ATGgt
Chimp      ...a.
Gorilla    ...a.
Orangutan  ...a.
Rhesus     ...a.
Baboon     ...a.
Marmoset   ...a.
Lemur      .CAag
Galago     ..Aag
Mouse      .....
Dog        .A.a.
```

---

## 6. uc002tif.2\_1\_7

**Summary**  

|  |  |  |  |  |  |  |  |  |  |  |  |  |  |  |  |  |  |  |  |  |  |  |  |  |  |
| --- | --- | --- | --- | --- | --- | --- | --- | --- | --- | --- | --- | --- | --- | --- | --- | --- | --- | --- | --- | --- | --- | --- | --- | --- | --- |
| No Exon ID Position (hg19) Dir Human donor Chimp donor Category Usage Gene symbol Protein accession mRNA accession Gene title Note|  |  |  |  |  |  |  |  |  |  |  |  |  | | --- | --- | --- | --- | --- | --- | --- | --- | --- | --- | --- | --- | --- | | 6 uc002tif.2\_1\_7 chr2:113514470 - GT CT (D8) intronization; inframe alternative CKAP2L Q8IYA6-2 AK097948.1 cytoskeleton associated protein 2-like  | | | | | | | | | | | | | | | | | | | | | | | | | |

**Orthologs**  

|  |  |  |  |  |  |  |  |  |  |  |  |  |  |  |  |  |  |  |  |  |  |  |  |  |  |  |  |  |  |  |  |  |  |  |  |  |  |  |  |  |  |  |  |  |  |  |  |  |  |  |  |  |  |  |  |  |  |  |  |  |  |  |  |  |  |  |  |  |  |  |  |  |  |  |  |  |  |  |  |  |  |  |  |  |  |  |  |  |  |  |  |  |  |  |  |
| --- | --- | --- | --- | --- | --- | --- | --- | --- | --- | --- | --- | --- | --- | --- | --- | --- | --- | --- | --- | --- | --- | --- | --- | --- | --- | --- | --- | --- | --- | --- | --- | --- | --- | --- | --- | --- | --- | --- | --- | --- | --- | --- | --- | --- | --- | --- | --- | --- | --- | --- | --- | --- | --- | --- | --- | --- | --- | --- | --- | --- | --- | --- | --- | --- | --- | --- | --- | --- | --- | --- | --- | --- | --- | --- | --- | --- | --- | --- | --- | --- | --- | --- | --- | --- | --- | --- | --- | --- | --- | --- | --- | --- | --- | --- | --- |
| Species Assembly Chromosome Exon start Exon end Dir Donor Exon sequence|  |  |  |  |  |  |  |  |  |  |  |  |  |  |  |  |  |  |  |  |  |  |  |  |  |  |  |  |  |  |  |  |  |  |  |  |  |  |  |  |  |  |  |  |  |  |  |  |  |  |  |  |  |  |  |  |  |  |  |  |  |  |  |  |  |  |  |  |  |  |  |  |  |  |  |  |  |  |  |  |  |  |  |  |  |  |  |  | | --- | --- | --- | --- | --- | --- | --- | --- | --- | --- | --- | --- | --- | --- | --- | --- | --- | --- | --- | --- | --- | --- | --- | --- | --- | --- | --- | --- | --- | --- | --- | --- | --- | --- | --- | --- | --- | --- | --- | --- | --- | --- | --- | --- | --- | --- | --- | --- | --- | --- | --- | --- | --- | --- | --- | --- | --- | --- | --- | --- | --- | --- | --- | --- | --- | --- | --- | --- | --- | --- | --- | --- | --- | --- | --- | --- | --- | --- | --- | --- | --- | --- | --- | --- | --- | --- | --- | --- | | Human hg19 chr2 113514470 113514472 - **GT** | ATG|  |  |  |  |  |  |  |  |  |  |  |  |  |  |  |  |  |  |  |  |  |  |  |  |  |  |  |  |  |  |  |  |  |  |  |  |  |  |  |  |  |  |  |  |  |  |  |  |  |  |  |  |  |  |  |  |  |  |  |  |  |  |  |  |  |  |  |  |  |  |  |  |  |  |  |  |  |  |  |  | | --- | --- | --- | --- | --- | --- | --- | --- | --- | --- | --- | --- | --- | --- | --- | --- | --- | --- | --- | --- | --- | --- | --- | --- | --- | --- | --- | --- | --- | --- | --- | --- | --- | --- | --- | --- | --- | --- | --- | --- | --- | --- | --- | --- | --- | --- | --- | --- | --- | --- | --- | --- | --- | --- | --- | --- | --- | --- | --- | --- | --- | --- | --- | --- | --- | --- | --- | --- | --- | --- | --- | --- | --- | --- | --- | --- | --- | --- | --- | --- | | Chimp panTro2 chr2a 113682725 113682727 - **CT** | ATG|  |  |  |  |  |  |  |  |  |  |  |  |  |  |  |  |  |  |  |  |  |  |  |  |  |  |  |  |  |  |  |  |  |  |  |  |  |  |  |  |  |  |  |  |  |  |  |  |  |  |  |  |  |  |  |  |  |  |  |  |  |  |  |  |  |  |  |  |  |  |  |  | | --- | --- | --- | --- | --- | --- | --- | --- | --- | --- | --- | --- | --- | --- | --- | --- | --- | --- | --- | --- | --- | --- | --- | --- | --- | --- | --- | --- | --- | --- | --- | --- | --- | --- | --- | --- | --- | --- | --- | --- | --- | --- | --- | --- | --- | --- | --- | --- | --- | --- | --- | --- | --- | --- | --- | --- | --- | --- | --- | --- | --- | --- | --- | --- | --- | --- | --- | --- | --- | --- | --- | --- | | Orangutan ponAbe2 chr2a 20743087 20743089 - **CT** | ATG|  |  |  |  |  |  |  |  |  |  |  |  |  |  |  |  |  |  |  |  |  |  |  |  |  |  |  |  |  |  |  |  |  |  |  |  |  |  |  |  |  |  |  |  |  |  |  |  |  |  |  |  |  |  |  |  |  |  |  |  |  |  |  |  | | --- | --- | --- | --- | --- | --- | --- | --- | --- | --- | --- | --- | --- | --- | --- | --- | --- | --- | --- | --- | --- | --- | --- | --- | --- | --- | --- | --- | --- | --- | --- | --- | --- | --- | --- | --- | --- | --- | --- | --- | --- | --- | --- | --- | --- | --- | --- | --- | --- | --- | --- | --- | --- | --- | --- | --- | --- | --- | --- | --- | --- | --- | --- | --- | | Rhesus rheMac2 chr13 112122214 112122216 - **CT** | ATG|  |  |  |  |  |  |  |  |  |  |  |  |  |  |  |  |  |  |  |  |  |  |  |  |  |  |  |  |  |  |  |  |  |  |  |  |  |  |  |  |  |  |  |  |  |  |  |  |  |  |  |  |  |  |  |  | | --- | --- | --- | --- | --- | --- | --- | --- | --- | --- | --- | --- | --- | --- | --- | --- | --- | --- | --- | --- | --- | --- | --- | --- | --- | --- | --- | --- | --- | --- | --- | --- | --- | --- | --- | --- | --- | --- | --- | --- | --- | --- | --- | --- | --- | --- | --- | --- | --- | --- | --- | --- | --- | --- | --- | --- | | Baboon papHam1 scaffold1075 92707 92709 + **CT** | ATG|  |  |  |  |  |  |  |  |  |  |  |  |  |  |  |  |  |  |  |  |  |  |  |  |  |  |  |  |  |  |  |  |  |  |  |  |  |  |  |  |  |  |  |  |  |  |  |  | | --- | --- | --- | --- | --- | --- | --- | --- | --- | --- | --- | --- | --- | --- | --- | --- | --- | --- | --- | --- | --- | --- | --- | --- | --- | --- | --- | --- | --- | --- | --- | --- | --- | --- | --- | --- | --- | --- | --- | --- | --- | --- | --- | --- | --- | --- | --- | --- | | Marmoset calJac1 Contig3611 21797 21799 - **CT** | ATG|  |  |  |  |  |  |  |  |  |  |  |  |  |  |  |  |  |  |  |  |  |  |  |  |  |  |  |  |  |  |  |  |  |  |  |  |  |  |  |  | | --- | --- | --- | --- | --- | --- | --- | --- | --- | --- | --- | --- | --- | --- | --- | --- | --- | --- | --- | --- | --- | --- | --- | --- | --- | --- | --- | --- | --- | --- | --- | --- | --- | --- | --- | --- | --- | --- | --- | --- | | Tarsier tarSyr1 scaffold\_3761 23945 23947 - **CT** | ATG|  |  |  |  |  |  |  |  |  |  |  |  |  |  |  |  |  |  |  |  |  |  |  |  |  |  |  |  |  |  |  |  | | --- | --- | --- | --- | --- | --- | --- | --- | --- | --- | --- | --- | --- | --- | --- | --- | --- | --- | --- | --- | --- | --- | --- | --- | --- | --- | --- | --- | --- | --- | --- | --- | | Galago otoGar1 scaffold\_93571.1-228472 44303 44305 - **CT** | CTG|  |  |  |  |  |  |  |  |  |  |  |  |  |  |  |  |  |  |  |  |  |  |  |  | | --- | --- | --- | --- | --- | --- | --- | --- | --- | --- | --- | --- | --- | --- | --- | --- | --- | --- | --- | --- | --- | --- | --- | --- | | Mouse mm9 chr2 129111521 129111523 - **CT** | ATG|  |  |  |  |  |  |  |  |  |  |  |  |  |  |  |  | | --- | --- | --- | --- | --- | --- | --- | --- | --- | --- | --- | --- | --- | --- | --- | --- | | Cow bosTau4 chr11 48173516 48173518 - **CT** | CTG|  |  |  |  |  |  |  |  | | --- | --- | --- | --- | --- | --- | --- | --- | | Dog canFam2 chr17 40066396 40066398 - **CT** | ATA | | | | | | | | | | | | | | | | | | | | | | | | | | | | | | | | | | | | | | | | | | | | | | | | | | | | | | | | | | | | | | | | | | | | | | | | | | | | | | | | | | | | |

**Alignment** (splice site sequences are in lowercase)  

```
Human      ATGgt
Chimp      ...c.
Orangutan  ...c.
Rhesus     ...c.
Baboon     ...c.
Marmoset   ...c.
Tarsier    ...c.
Galago     C..c.
Mouse      ...c.
Cow        C..c.
Dog        ..Ac.
```

---

## 7. uc010fqy.2\_2\_3

**Summary**  

|  |  |  |  |  |  |  |  |  |  |  |  |  |  |  |  |  |  |  |  |  |  |  |  |  |  |
| --- | --- | --- | --- | --- | --- | --- | --- | --- | --- | --- | --- | --- | --- | --- | --- | --- | --- | --- | --- | --- | --- | --- | --- | --- | --- |
| No Exon ID Position (hg19) Dir Human donor Chimp donor Category Usage Gene symbol Protein accession mRNA accession Gene title Note|  |  |  |  |  |  |  |  |  |  |  |  |  | | --- | --- | --- | --- | --- | --- | --- | --- | --- | --- | --- | --- | --- | | 7 uc010fqy.2\_2\_3 chr2:177054409 + GT GC (D6) exonization; frameshift alternative HOXD1 B2RAB4 BC028190.1 homeobox D1 dbSNP:rs13390932 | | | | | | | | | | | | | | | | | | | | | | | | | |

**Orthologs**  

|  |  |  |  |  |  |  |  |  |  |  |  |  |  |  |  |  |  |  |  |  |  |  |  |  |  |  |  |  |  |  |  |  |  |  |  |  |  |  |  |  |  |  |  |  |  |  |  |  |  |  |  |  |  |  |  |  |  |  |  |  |  |  |  |  |  |  |  |  |  |  |  |  |  |  |  |  |  |  |  |
| --- | --- | --- | --- | --- | --- | --- | --- | --- | --- | --- | --- | --- | --- | --- | --- | --- | --- | --- | --- | --- | --- | --- | --- | --- | --- | --- | --- | --- | --- | --- | --- | --- | --- | --- | --- | --- | --- | --- | --- | --- | --- | --- | --- | --- | --- | --- | --- | --- | --- | --- | --- | --- | --- | --- | --- | --- | --- | --- | --- | --- | --- | --- | --- | --- | --- | --- | --- | --- | --- | --- | --- | --- | --- | --- | --- | --- | --- | --- | --- |
| Species Assembly Chromosome Exon start Exon end Dir Donor Exon sequence|  |  |  |  |  |  |  |  |  |  |  |  |  |  |  |  |  |  |  |  |  |  |  |  |  |  |  |  |  |  |  |  |  |  |  |  |  |  |  |  |  |  |  |  |  |  |  |  |  |  |  |  |  |  |  |  |  |  |  |  |  |  |  |  |  |  |  |  |  |  |  |  | | --- | --- | --- | --- | --- | --- | --- | --- | --- | --- | --- | --- | --- | --- | --- | --- | --- | --- | --- | --- | --- | --- | --- | --- | --- | --- | --- | --- | --- | --- | --- | --- | --- | --- | --- | --- | --- | --- | --- | --- | --- | --- | --- | --- | --- | --- | --- | --- | --- | --- | --- | --- | --- | --- | --- | --- | --- | --- | --- | --- | --- | --- | --- | --- | --- | --- | --- | --- | --- | --- | --- | --- | | Human hg19 chr2 177054277 177054409 + **GT** | ACAGCGGTTTGGGTTTTGTGGAGGAGACGCGTTCCCGGGCGAATTCCATGGAGTCTGCCTCGAGTACAGATTCGGAAAATGTGCCAGGCATTGAGAAAATTTCAGGAAGGAGCTCTCCGTGGAACTTCTCCTG|  |  |  |  |  |  |  |  |  |  |  |  |  |  |  |  |  |  |  |  |  |  |  |  |  |  |  |  |  |  |  |  |  |  |  |  |  |  |  |  |  |  |  |  |  |  |  |  |  |  |  |  |  |  |  |  |  |  |  |  |  |  |  |  | | --- | --- | --- | --- | --- | --- | --- | --- | --- | --- | --- | --- | --- | --- | --- | --- | --- | --- | --- | --- | --- | --- | --- | --- | --- | --- | --- | --- | --- | --- | --- | --- | --- | --- | --- | --- | --- | --- | --- | --- | --- | --- | --- | --- | --- | --- | --- | --- | --- | --- | --- | --- | --- | --- | --- | --- | --- | --- | --- | --- | --- | --- | --- | --- | | Chimp panTro2 chr2b 181195617 181195749 + **GC** | ACAGCGGTTTGGGTTTTGTGGAGGAGACGCGTTCCCGGGCGAATTCCATGGAGTCTGCCTCGAGTACAGATTCGGAAAATGTGCCAGGCATTGAGAAAATTTCAGGAAGGAGCTCTCCGTGGAACTTCTCCTG|  |  |  |  |  |  |  |  |  |  |  |  |  |  |  |  |  |  |  |  |  |  |  |  |  |  |  |  |  |  |  |  |  |  |  |  |  |  |  |  |  |  |  |  |  |  |  |  |  |  |  |  |  |  |  |  | | --- | --- | --- | --- | --- | --- | --- | --- | --- | --- | --- | --- | --- | --- | --- | --- | --- | --- | --- | --- | --- | --- | --- | --- | --- | --- | --- | --- | --- | --- | --- | --- | --- | --- | --- | --- | --- | --- | --- | --- | --- | --- | --- | --- | --- | --- | --- | --- | --- | --- | --- | --- | --- | --- | --- | --- | | Gorilla gorGor1 Supercontig\_0413396 1690 1822 - **GC** | ACAGCGGTTTGGGTTTGTGGAGGAGACGCNTTCCCGGGCGAATTCCACGGAGTCTGCCTCGAGTACAGATTCGGAAAATGTGCCAGGCATTGAGAAAATTTCAGGGAAGGAGCTCTCCGTGGAACTTCTCCTG|  |  |  |  |  |  |  |  |  |  |  |  |  |  |  |  |  |  |  |  |  |  |  |  |  |  |  |  |  |  |  |  |  |  |  |  |  |  |  |  |  |  |  |  |  |  |  |  | | --- | --- | --- | --- | --- | --- | --- | --- | --- | --- | --- | --- | --- | --- | --- | --- | --- | --- | --- | --- | --- | --- | --- | --- | --- | --- | --- | --- | --- | --- | --- | --- | --- | --- | --- | --- | --- | --- | --- | --- | --- | --- | --- | --- | --- | --- | --- | --- | | Orangutan ponAbe2 chr2b 66346877 66347009 + **GC** | ACAGCGGTTTGGGTTTTGTGGAGGAGACGCGTTCCCGGGCGAATTCCATTGAGTCTGCCTCGAGTACAGACTCGGAAAATGTGCCAGGCATTCAGAAAATTGCAGGAAGGAGCTCTCCGTGGAATGTCTCTTG|  |  |  |  |  |  |  |  |  |  |  |  |  |  |  |  |  |  |  |  |  |  |  |  |  |  |  |  |  |  |  |  |  |  |  |  |  |  |  |  | | --- | --- | --- | --- | --- | --- | --- | --- | --- | --- | --- | --- | --- | --- | --- | --- | --- | --- | --- | --- | --- | --- | --- | --- | --- | --- | --- | --- | --- | --- | --- | --- | --- | --- | --- | --- | --- | --- | --- | --- | | Rhesus rheMac2 chr12 39831165 39831297 + **GC** | ACAGCGGTTTGGGTTTTGTGGAGAAGACGCGTTCCTGGGCGAATTCCATTGAGTCTGCCTCGAGTACTGACTCGGAAAATGTGCCAGGCATTGAGAAAATTTCAGGAAGGAGCTCTCCGTGAAATTTCTCCTG|  |  |  |  |  |  |  |  |  |  |  |  |  |  |  |  |  |  |  |  |  |  |  |  |  |  |  |  |  |  |  |  | | --- | --- | --- | --- | --- | --- | --- | --- | --- | --- | --- | --- | --- | --- | --- | --- | --- | --- | --- | --- | --- | --- | --- | --- | --- | --- | --- | --- | --- | --- | --- | --- | | Baboon papHam1 scaffold10146 794 926 - **GC** | ACAGCGGTTTGGGTTTTGCGGAGAAGACACGTTCTTGGGCGAATTCCATTGAGTCTGCCTCGAGTACAGACTCGGAAAATGTGCCAGGCATTGAGAAAATTTCAGGAAGGAGCTCTCCGTGAAATTCCTCCTG|  |  |  |  |  |  |  |  |  |  |  |  |  |  |  |  |  |  |  |  |  |  |  |  | | --- | --- | --- | --- | --- | --- | --- | --- | --- | --- | --- | --- | --- | --- | --- | --- | --- | --- | --- | --- | --- | --- | --- | --- | | Marmoset calJac1 Contig349 558227 558358 + **GC** | ACAGCGGTTGGGGTTTTGTGGAGGAGACGCGCTCCCGGGCGAATTCCATTGAGCGTGCCTCGAGAACAGACTCGGAAAATGTGCCAGCCATTGAGAAAATTTCAGGAAGGAGCTCTCCGTGGAGTTTCTCTG|  |  |  |  |  |  |  |  |  |  |  |  |  |  |  |  | | --- | --- | --- | --- | --- | --- | --- | --- | --- | --- | --- | --- | --- | --- | --- | --- | | Lemur micMur1 scaffold\_2769 58411 58559 + **GC** | ACAGCAGTTTGGTTTTAAGGAGGAAATGCGTTCCTGGGCGTTATTAAAGCAGGAGAATTCCATAGAGCATCCCCTGAGTCCCGGCTCAGAAAGCGTGCTAGGCATTGAGAAAATGACAGAAAGGAGCTCTTCGTGGAGCGCTTCTCCTG|  |  |  |  |  |  |  |  | | --- | --- | --- | --- | --- | --- | --- | --- | | Galago otoGar1 scaffold\_108759.1-101791 93392 93541 + **GC** | ACAACAGTTTGGGTTTTATGCAGGAAATGCGTGCCTGGGCATTATTAAAGCAGGCGAATTTCATCGGGCGTGCCTCGAGTCCAGGCTCGGAAAACTTGCCAGGCACTTAGAAAATTATATAAAAGAACTTTCCACGGAGTGTTTCTCCTG | | | | | | | | | | | | | | | | | | | | | | | | | | | | | | | | | | | | | | | | | | | | | | | | | | | | | | | | | | | | | | | | | | | | | | |

**Alignment** (splice site sequences are in lowercase)  

```
Human      agACAGCGGTTTGGGTTTTGTGGAGGAGACGCGTTCCCGGGC---------------GAATTCCATGGAGTCTGCCTCGA
Chimp      ..........................................---------------.......................
Gorilla    ...............-................N.........---------------........C..............
Orangutan  ..........................................---------------.........T.............
Rhesus     .........................A...........T....---------------.........T.............
Baboon     ....................C....A....A.....TT....---------------.........T.............
Marmoset   ...........G.....................C........---------------.........T...CG........
Lemur      .......A....-......AA......A.T.......T....GTTATTAAAGCAGGA.........A...CA.C..CT..
Galago     .t...A.A...........A..C....A.T....G..T....ATTATTAAAGCAGGC.....T...C.G.CG........

Human      GTACAGATTCGGAAAATGTGCCAGGCATTGAGAAAATTTCA-GGAAGGAGCTCTCCGTGGA--ACTTCTCCTGgt
Chimp      .........................................-...................--...........c
Gorilla    .........................................G...................--...........c
Orangutan  .......C.....................C........G..-...................--.TG....T...c
Rhesus     ....T..C.................................-.................A.--.T.........c
Baboon     .......C.................................-.................A.--.T.C.......c
Marmoset   .A.....C................C................-...................--GT.....-...c
Lemur      ..C.C.GC..A....GC....T...............GA..-.A..........T......GCG..........c
Galago     ..C...GC........CT.........C.T........AT.-TA..A..A..T...AC...GTGT.........c
```

---

## 8. uc010zhi.1\_1\_13

**Summary**  

|  |  |  |  |  |  |  |  |  |  |  |  |  |  |  |  |  |  |  |  |  |  |  |  |  |  |
| --- | --- | --- | --- | --- | --- | --- | --- | --- | --- | --- | --- | --- | --- | --- | --- | --- | --- | --- | --- | --- | --- | --- | --- | --- | --- |
| No Exon ID Position (hg19) Dir Human donor Chimp donor Category Usage Gene symbol Protein accession mRNA accession Gene title Note|  |  |  |  |  |  |  |  |  |  |  |  |  | | --- | --- | --- | --- | --- | --- | --- | --- | --- | --- | --- | --- | --- | | 8 uc010zhi.1\_1\_13 chr2:201728824 - GT GC (D7) exonization; novel start alternative CLK1 NP\_001155879.1 NM\_001162407.1 CDC-like kinase 1  | | | | | | | | | | | | | | | | | | | | | | | | | |

**Orthologs**  

|  |  |  |  |  |  |  |  |  |  |  |  |  |  |  |  |  |  |  |  |  |  |  |  |  |  |  |  |  |  |  |  |  |  |  |  |  |  |  |  |  |  |  |  |  |  |  |  |  |  |  |  |  |  |  |  |  |  |  |  |  |  |  |  |
| --- | --- | --- | --- | --- | --- | --- | --- | --- | --- | --- | --- | --- | --- | --- | --- | --- | --- | --- | --- | --- | --- | --- | --- | --- | --- | --- | --- | --- | --- | --- | --- | --- | --- | --- | --- | --- | --- | --- | --- | --- | --- | --- | --- | --- | --- | --- | --- | --- | --- | --- | --- | --- | --- | --- | --- | --- | --- | --- | --- | --- | --- | --- | --- |
| Species Assembly Chromosome Exon start Exon end Dir Donor Exon sequence|  |  |  |  |  |  |  |  |  |  |  |  |  |  |  |  |  |  |  |  |  |  |  |  |  |  |  |  |  |  |  |  |  |  |  |  |  |  |  |  |  |  |  |  |  |  |  |  |  |  |  |  |  |  |  |  | | --- | --- | --- | --- | --- | --- | --- | --- | --- | --- | --- | --- | --- | --- | --- | --- | --- | --- | --- | --- | --- | --- | --- | --- | --- | --- | --- | --- | --- | --- | --- | --- | --- | --- | --- | --- | --- | --- | --- | --- | --- | --- | --- | --- | --- | --- | --- | --- | --- | --- | --- | --- | --- | --- | --- | --- | | Human hg19 chr2 201728824 201728949 - **GT** | ATGGCGGCTGGGCGGAGGCCGGCTTCGGCCCTGTGGCCGGAAAGGCGAGGCTCCCCGTTGAGGGGGGATTTGCTGGGGTTCCAGAATGTGCGTGAGCCAAGCAGCTGTGGGGAAACGTTGTCTGGA|  |  |  |  |  |  |  |  |  |  |  |  |  |  |  |  |  |  |  |  |  |  |  |  |  |  |  |  |  |  |  |  |  |  |  |  |  |  |  |  |  |  |  |  |  |  |  |  | | --- | --- | --- | --- | --- | --- | --- | --- | --- | --- | --- | --- | --- | --- | --- | --- | --- | --- | --- | --- | --- | --- | --- | --- | --- | --- | --- | --- | --- | --- | --- | --- | --- | --- | --- | --- | --- | --- | --- | --- | --- | --- | --- | --- | --- | --- | --- | --- | | Chimp panTro2 chr2b 206307627 206307752 - **GC** | ATGGCGGCTGGGCGGAGGCCGGCTTCGGCCCTGTGGCCGGAAAGGCGAGGCTCCCCGTTGAGGGGGGGTTTGCTGGGGTTCCAGAATGTGCGTGAGCCACGCAGCTGTGGGGAAACGTTGTCTGGA|  |  |  |  |  |  |  |  |  |  |  |  |  |  |  |  |  |  |  |  |  |  |  |  |  |  |  |  |  |  |  |  |  |  |  |  |  |  |  |  | | --- | --- | --- | --- | --- | --- | --- | --- | --- | --- | --- | --- | --- | --- | --- | --- | --- | --- | --- | --- | --- | --- | --- | --- | --- | --- | --- | --- | --- | --- | --- | --- | --- | --- | --- | --- | --- | --- | --- | --- | | Gorilla gorGor1 Supercontig\_0002069 14986 15111 - **GC** | ATGGCGGCTGGGCGGAGGCCGGCTTCGGCCCTGTGGCCGGAAAGGCGAGGCTCCCCGTTGAGGGGGTGTTTGCTGGGGTTCCAGAATGTGCATGAGCCACGCAGCTGTGGGGAAACGTTGTCTGGA|  |  |  |  |  |  |  |  |  |  |  |  |  |  |  |  |  |  |  |  |  |  |  |  |  |  |  |  |  |  |  |  | | --- | --- | --- | --- | --- | --- | --- | --- | --- | --- | --- | --- | --- | --- | --- | --- | --- | --- | --- | --- | --- | --- | --- | --- | --- | --- | --- | --- | --- | --- | --- | --- | | Orangutan ponAbe2 chr2b 92118000 92118126 - **GC** | ATGGCGGCTGGGCGGAGGCCCGCTTCGGCCCTGTGGCCGGAAAGGCGAGGCTCCCTGTTGGGGGTGGGGTTTGCTGGGGTTCCAGAATGTGCGTGAGCTGCGCAGCTGAGGGGAAACGTTGTCTGGA|  |  |  |  |  |  |  |  |  |  |  |  |  |  |  |  |  |  |  |  |  |  |  |  | | --- | --- | --- | --- | --- | --- | --- | --- | --- | --- | --- | --- | --- | --- | --- | --- | --- | --- | --- | --- | --- | --- | --- | --- | | Rhesus rheMac2 chr12 64616938 64617064 - **GC** | ATGGCGGCTGGGCGGAGGCCGGCCTTGGCACTGTGGCCGGAAAGGCGAGGCTCCCTGTTGGGGGTGGGGTTTGCTGGGGTTCCGGAATGTGCTTGAAGCACGCAGCTGTGGGGAAATGTCGTCTGGA|  |  |  |  |  |  |  |  |  |  |  |  |  |  |  |  | | --- | --- | --- | --- | --- | --- | --- | --- | --- | --- | --- | --- | --- | --- | --- | --- | | Baboon papHam1 scaffold30412 16833 16959 - **GC** | ATGGCGGCTGGGCGGAGGCCGGCCTTGGCACTGTGGCCGGAAAGGCGAGGCTCCCTGTTGGGGGTGGGGTTTGCTGGGGTTCCGGAATGTGCTTGAAGCACGCAGCTGTGGGGAAATGTCGTCTGGA|  |  |  |  |  |  |  |  | | --- | --- | --- | --- | --- | --- | --- | --- | | Marmoset calJac1 Contig1570 16440 16552 - **GC** | ATGGCGGCTGGGCGGAGGCCGGCCTTGGTCACTGGGCGGAAAGGCGAGGCTCCCTGTTGGGGGTGGGGTTTGTTGGGATTCCAGAAAATCGCCGCTGTGGGGAAATGTCTGCG | | | | | | | | | | | | | | | | | | | | | | | | | | | | | | | | | | | | | | | | | | | | | | | | | | | | | | | | |

**Alignment** (splice site sequences are in lowercase)  

```
Human      ATGGCGGCTGGGCGGAGGCCGGCTTCGGCCCTGTGGCCGGAAAGGCGAGGCTCCCCGTTGAGGG-GGGATTTGCTGGGGT
Chimp      ................................................................-...G...........
Gorilla    ................................................................-..TG...........
Orangutan  ....................C..................................T....G...T...G...........
Rhesus     .......................C.T...A.........................T....G...T...G...........
Baboon     .......................C.T...A.........................T....G...T...G...........
Marmoset   .......................C.T..T.AC-...G..................T....G...T...G....T....A.

Human      TCCAGAATGTGCGTGAGCCAAGCAGCTGTGGGGAAACGTTGTCTGGAgt
Chimp      ....................C...........................c
Gorilla    ............A.......C...........................c
Orangutan  ..................TGC.......A...................c
Rhesus     ...G........T...AG..C...............T..C........c
Baboon     ...G........T...AG..C...............T..C........c
Marmoset   .......AA.----------C..C............---......CG.c
```

---

## 9. uc002vmt.1\_9\_10

**Summary**  

|  |  |  |  |  |  |  |  |  |  |  |  |  |  |  |  |  |  |  |  |  |  |  |  |  |  |
| --- | --- | --- | --- | --- | --- | --- | --- | --- | --- | --- | --- | --- | --- | --- | --- | --- | --- | --- | --- | --- | --- | --- | --- | --- | --- |
| No Exon ID Position (hg19) Dir Human donor Chimp donor Category Usage Gene symbol Protein accession mRNA accession Gene title Note|  |  |  |  |  |  |  |  |  |  |  |  |  | | --- | --- | --- | --- | --- | --- | --- | --- | --- | --- | --- | --- | --- | | 9 uc002vmt.1\_9\_10 chr2:223066131 - GT AT (D9) intronization; frameshift alternative PAX3 NP\_852124.1 NM\_181459.3 paired box 3  | | | | | | | | | | | | | | | | | | | | | | | | | |

**Orthologs**  

|  |  |  |  |  |  |  |  |  |  |  |  |  |  |  |  |  |  |  |  |  |  |  |  |  |  |  |  |  |  |  |  |  |  |  |  |  |  |  |  |  |  |  |  |  |  |  |  |  |  |  |  |  |  |  |  |  |  |  |  |  |  |  |  |  |  |  |  |  |  |  |  |  |  |  |  |  |  |  |  |  |  |  |  |  |  |  |  |  |  |  |  |  |  |  |  |  |  |  |  |  |  |  |  |  |  |  |  |  |  |  |  |
| --- | --- | --- | --- | --- | --- | --- | --- | --- | --- | --- | --- | --- | --- | --- | --- | --- | --- | --- | --- | --- | --- | --- | --- | --- | --- | --- | --- | --- | --- | --- | --- | --- | --- | --- | --- | --- | --- | --- | --- | --- | --- | --- | --- | --- | --- | --- | --- | --- | --- | --- | --- | --- | --- | --- | --- | --- | --- | --- | --- | --- | --- | --- | --- | --- | --- | --- | --- | --- | --- | --- | --- | --- | --- | --- | --- | --- | --- | --- | --- | --- | --- | --- | --- | --- | --- | --- | --- | --- | --- | --- | --- | --- | --- | --- | --- | --- | --- | --- | --- | --- | --- | --- | --- | --- | --- | --- | --- | --- | --- | --- | --- |
| Species Assembly Chromosome Exon start Exon end Dir Donor Exon sequence|  |  |  |  |  |  |  |  |  |  |  |  |  |  |  |  |  |  |  |  |  |  |  |  |  |  |  |  |  |  |  |  |  |  |  |  |  |  |  |  |  |  |  |  |  |  |  |  |  |  |  |  |  |  |  |  |  |  |  |  |  |  |  |  |  |  |  |  |  |  |  |  |  |  |  |  |  |  |  |  |  |  |  |  |  |  |  |  |  |  |  |  |  |  |  |  |  |  |  |  |  |  |  |  | | --- | --- | --- | --- | --- | --- | --- | --- | --- | --- | --- | --- | --- | --- | --- | --- | --- | --- | --- | --- | --- | --- | --- | --- | --- | --- | --- | --- | --- | --- | --- | --- | --- | --- | --- | --- | --- | --- | --- | --- | --- | --- | --- | --- | --- | --- | --- | --- | --- | --- | --- | --- | --- | --- | --- | --- | --- | --- | --- | --- | --- | --- | --- | --- | --- | --- | --- | --- | --- | --- | --- | --- | --- | --- | --- | --- | --- | --- | --- | --- | --- | --- | --- | --- | --- | --- | --- | --- | --- | --- | --- | --- | --- | --- | --- | --- | --- | --- | --- | --- | --- | --- | --- | --- | | Human hg19 chr2 223066131 223066161 - **GT** | GTGCCTTTCATTATCTCAAGCCAGATATCGC|  |  |  |  |  |  |  |  |  |  |  |  |  |  |  |  |  |  |  |  |  |  |  |  |  |  |  |  |  |  |  |  |  |  |  |  |  |  |  |  |  |  |  |  |  |  |  |  |  |  |  |  |  |  |  |  |  |  |  |  |  |  |  |  |  |  |  |  |  |  |  |  |  |  |  |  |  |  |  |  |  |  |  |  |  |  |  |  |  |  |  |  |  |  |  |  | | --- | --- | --- | --- | --- | --- | --- | --- | --- | --- | --- | --- | --- | --- | --- | --- | --- | --- | --- | --- | --- | --- | --- | --- | --- | --- | --- | --- | --- | --- | --- | --- | --- | --- | --- | --- | --- | --- | --- | --- | --- | --- | --- | --- | --- | --- | --- | --- | --- | --- | --- | --- | --- | --- | --- | --- | --- | --- | --- | --- | --- | --- | --- | --- | --- | --- | --- | --- | --- | --- | --- | --- | --- | --- | --- | --- | --- | --- | --- | --- | --- | --- | --- | --- | --- | --- | --- | --- | --- | --- | --- | --- | --- | --- | --- | --- | | Chimp panTro2 chr2b 228182873 228182903 - **AT** | GTGCCTTTCATTATCTCAAGCCAGATATCGC|  |  |  |  |  |  |  |  |  |  |  |  |  |  |  |  |  |  |  |  |  |  |  |  |  |  |  |  |  |  |  |  |  |  |  |  |  |  |  |  |  |  |  |  |  |  |  |  |  |  |  |  |  |  |  |  |  |  |  |  |  |  |  |  |  |  |  |  |  |  |  |  |  |  |  |  |  |  |  |  |  |  |  |  |  |  |  |  | | --- | --- | --- | --- | --- | --- | --- | --- | --- | --- | --- | --- | --- | --- | --- | --- | --- | --- | --- | --- | --- | --- | --- | --- | --- | --- | --- | --- | --- | --- | --- | --- | --- | --- | --- | --- | --- | --- | --- | --- | --- | --- | --- | --- | --- | --- | --- | --- | --- | --- | --- | --- | --- | --- | --- | --- | --- | --- | --- | --- | --- | --- | --- | --- | --- | --- | --- | --- | --- | --- | --- | --- | --- | --- | --- | --- | --- | --- | --- | --- | --- | --- | --- | --- | --- | --- | --- | --- | | Gorilla gorGor1 Supercontig\_0549285 766 796 + **AT** | GTGCCTTTCATTATCTCAAGCCAGATATCGC|  |  |  |  |  |  |  |  |  |  |  |  |  |  |  |  |  |  |  |  |  |  |  |  |  |  |  |  |  |  |  |  |  |  |  |  |  |  |  |  |  |  |  |  |  |  |  |  |  |  |  |  |  |  |  |  |  |  |  |  |  |  |  |  |  |  |  |  |  |  |  |  |  |  |  |  |  |  |  |  | | --- | --- | --- | --- | --- | --- | --- | --- | --- | --- | --- | --- | --- | --- | --- | --- | --- | --- | --- | --- | --- | --- | --- | --- | --- | --- | --- | --- | --- | --- | --- | --- | --- | --- | --- | --- | --- | --- | --- | --- | --- | --- | --- | --- | --- | --- | --- | --- | --- | --- | --- | --- | --- | --- | --- | --- | --- | --- | --- | --- | --- | --- | --- | --- | --- | --- | --- | --- | --- | --- | --- | --- | --- | --- | --- | --- | --- | --- | --- | --- | | Orangutan ponAbe2 chr2b 114209032 114209062 - **AT** | GTGCCTTTCATTATCTCAAGCCAGATATCGC|  |  |  |  |  |  |  |  |  |  |  |  |  |  |  |  |  |  |  |  |  |  |  |  |  |  |  |  |  |  |  |  |  |  |  |  |  |  |  |  |  |  |  |  |  |  |  |  |  |  |  |  |  |  |  |  |  |  |  |  |  |  |  |  |  |  |  |  |  |  |  |  | | --- | --- | --- | --- | --- | --- | --- | --- | --- | --- | --- | --- | --- | --- | --- | --- | --- | --- | --- | --- | --- | --- | --- | --- | --- | --- | --- | --- | --- | --- | --- | --- | --- | --- | --- | --- | --- | --- | --- | --- | --- | --- | --- | --- | --- | --- | --- | --- | --- | --- | --- | --- | --- | --- | --- | --- | --- | --- | --- | --- | --- | --- | --- | --- | --- | --- | --- | --- | --- | --- | --- | --- | | Rhesus rheMac2 chr12 86071397 86071427 - **AT** | GTGCCTTTCATTATCTCAAGCCAGATATCGC|  |  |  |  |  |  |  |  |  |  |  |  |  |  |  |  |  |  |  |  |  |  |  |  |  |  |  |  |  |  |  |  |  |  |  |  |  |  |  |  |  |  |  |  |  |  |  |  |  |  |  |  |  |  |  |  |  |  |  |  |  |  |  |  | | --- | --- | --- | --- | --- | --- | --- | --- | --- | --- | --- | --- | --- | --- | --- | --- | --- | --- | --- | --- | --- | --- | --- | --- | --- | --- | --- | --- | --- | --- | --- | --- | --- | --- | --- | --- | --- | --- | --- | --- | --- | --- | --- | --- | --- | --- | --- | --- | --- | --- | --- | --- | --- | --- | --- | --- | --- | --- | --- | --- | --- | --- | --- | --- | | Baboon papHam1 scaffold8480 13779 13809 - **AT** | GTGCCTTTCATTATCTCAAGCCAGATATCGC|  |  |  |  |  |  |  |  |  |  |  |  |  |  |  |  |  |  |  |  |  |  |  |  |  |  |  |  |  |  |  |  |  |  |  |  |  |  |  |  |  |  |  |  |  |  |  |  |  |  |  |  |  |  |  |  | | --- | --- | --- | --- | --- | --- | --- | --- | --- | --- | --- | --- | --- | --- | --- | --- | --- | --- | --- | --- | --- | --- | --- | --- | --- | --- | --- | --- | --- | --- | --- | --- | --- | --- | --- | --- | --- | --- | --- | --- | --- | --- | --- | --- | --- | --- | --- | --- | --- | --- | --- | --- | --- | --- | --- | --- | | Marmoset calJac1 Contig3263 136828 136858 + **AT** | GTGCCTTTCATTATCTCAAGCCAGATATCGC|  |  |  |  |  |  |  |  |  |  |  |  |  |  |  |  |  |  |  |  |  |  |  |  |  |  |  |  |  |  |  |  |  |  |  |  |  |  |  |  |  |  |  |  |  |  |  |  | | --- | --- | --- | --- | --- | --- | --- | --- | --- | --- | --- | --- | --- | --- | --- | --- | --- | --- | --- | --- | --- | --- | --- | --- | --- | --- | --- | --- | --- | --- | --- | --- | --- | --- | --- | --- | --- | --- | --- | --- | --- | --- | --- | --- | --- | --- | --- | --- | | Tarsier tarSyr1 scaffold\_9940 4108 4138 - **AT** | GTGCCTTTCATTATCTCAAGCCAGATATCGC|  |  |  |  |  |  |  |  |  |  |  |  |  |  |  |  |  |  |  |  |  |  |  |  |  |  |  |  |  |  |  |  |  |  |  |  |  |  |  |  | | --- | --- | --- | --- | --- | --- | --- | --- | --- | --- | --- | --- | --- | --- | --- | --- | --- | --- | --- | --- | --- | --- | --- | --- | --- | --- | --- | --- | --- | --- | --- | --- | --- | --- | --- | --- | --- | --- | --- | --- | | Lemur micMur1 scaffold\_863 289901 289931 - **AT** | GTGCCTTTCATTATCTCAAGCCAGATATCGC|  |  |  |  |  |  |  |  |  |  |  |  |  |  |  |  |  |  |  |  |  |  |  |  |  |  |  |  |  |  |  |  | | --- | --- | --- | --- | --- | --- | --- | --- | --- | --- | --- | --- | --- | --- | --- | --- | --- | --- | --- | --- | --- | --- | --- | --- | --- | --- | --- | --- | --- | --- | --- | --- | | Galago otoGar1 scaffold\_109389.1-239627 38001 38031 - **AT** | GTGCCTTTCATTATCTCAAGCCAGATATCGC|  |  |  |  |  |  |  |  |  |  |  |  |  |  |  |  |  |  |  |  |  |  |  |  | | --- | --- | --- | --- | --- | --- | --- | --- | --- | --- | --- | --- | --- | --- | --- | --- | --- | --- | --- | --- | --- | --- | --- | --- | | Mouse mm9 chr1 78099380 78099410 - **AT** | GTGCCTTTCATTATCTCAAGCCAGATATTGC|  |  |  |  |  |  |  |  |  |  |  |  |  |  |  |  | | --- | --- | --- | --- | --- | --- | --- | --- | --- | --- | --- | --- | --- | --- | --- | --- | | Cow bosTau4 chr2 114994349 114994379 - **AT** | GTGCCTTTCATTATCTCAAGCCAGATATCGC|  |  |  |  |  |  |  |  | | --- | --- | --- | --- | --- | --- | --- | --- | | Dog canFam2 chr37 31348626 31348656 - **CT** | GTGCCTTTCATTATCTCAAGCCAGATATCGC | | | | | | | | | | | | | | | | | | | | | | | | | | | | | | | | | | | | | | | | | | | | | | | | | | | | | | | | | | | | | | | | | | | | | | | | | | | | | | | | | | | | | | | | | | | | | | | | | | |

**Alignment** (splice site sequences are in lowercase)  

```
Human      agGTGCCTTTCATTATCTCAAGCCAGATATCGCgt
Chimp      .................................a.
Gorilla    .................................a.
Orangutan  .................................a.
Rhesus     .................................a.
Baboon     .................................a.
Marmoset   .................................a.
Tarsier    .................................a.
Lemur      .................................a.
Galago     .................................a.
Mouse      ..............................T..a.
Cow        .................................a.
Dog        .................................c.
```

---

## 10. uc011axk.1\_2\_17

**Summary**  

|  |  |  |  |  |  |  |  |  |  |  |  |  |  |  |  |  |  |  |  |  |  |  |  |  |  |
| --- | --- | --- | --- | --- | --- | --- | --- | --- | --- | --- | --- | --- | --- | --- | --- | --- | --- | --- | --- | --- | --- | --- | --- | --- | --- |
| No Exon ID Position (hg19) Dir Human donor Chimp donor Category Usage Gene symbol Protein accession mRNA accession Gene title Note|  |  |  |  |  |  |  |  |  |  |  |  |  | | --- | --- | --- | --- | --- | --- | --- | --- | --- | --- | --- | --- | --- | | 10 uc011axk.1\_2\_17 chr3:33118586 - GT AT (D5) exonization; inframe alternative GLB1 B7Z6Q5 AK300753.1 galactosidase, beta 1  | | | | | | | | | | | | | | | | | | | | | | | | | |

**Orthologs**  

|  |  |  |  |  |  |  |  |  |  |  |  |  |  |  |  |  |  |  |  |  |  |  |  |  |  |  |  |  |  |  |  |  |  |  |  |  |  |  |  |  |  |  |  |  |  |  |  |  |  |  |  |  |  |  |  |
| --- | --- | --- | --- | --- | --- | --- | --- | --- | --- | --- | --- | --- | --- | --- | --- | --- | --- | --- | --- | --- | --- | --- | --- | --- | --- | --- | --- | --- | --- | --- | --- | --- | --- | --- | --- | --- | --- | --- | --- | --- | --- | --- | --- | --- | --- | --- | --- | --- | --- | --- | --- | --- | --- | --- | --- |
| Species Assembly Chromosome Exon start Exon end Dir Donor Exon sequence|  |  |  |  |  |  |  |  |  |  |  |  |  |  |  |  |  |  |  |  |  |  |  |  |  |  |  |  |  |  |  |  |  |  |  |  |  |  |  |  |  |  |  |  |  |  |  |  | | --- | --- | --- | --- | --- | --- | --- | --- | --- | --- | --- | --- | --- | --- | --- | --- | --- | --- | --- | --- | --- | --- | --- | --- | --- | --- | --- | --- | --- | --- | --- | --- | --- | --- | --- | --- | --- | --- | --- | --- | --- | --- | --- | --- | --- | --- | --- | --- | | Human hg19 chr3 33118586 33118729 - **GT** | TCTCGATTCCTTCCTTGGGCTTTTCATCTGCCACGGCAAGCGCCTAAGTCTCAGCTGCCGCTTCACAAAAGAGGTACCAAGACTGCACCGAATGAGCACGCAAGCAGCAACAGGAGTGGGAGAAGAAGGAGGCGGCAGCAGTGG|  |  |  |  |  |  |  |  |  |  |  |  |  |  |  |  |  |  |  |  |  |  |  |  |  |  |  |  |  |  |  |  |  |  |  |  |  |  |  |  | | --- | --- | --- | --- | --- | --- | --- | --- | --- | --- | --- | --- | --- | --- | --- | --- | --- | --- | --- | --- | --- | --- | --- | --- | --- | --- | --- | --- | --- | --- | --- | --- | --- | --- | --- | --- | --- | --- | --- | --- | | Chimp panTro2 chr3 33872359 33872502 - **AT** | TCTTGATTCCTTCCTTGGGCTTTTCATCTGCCACGGCAAGCGCCTAAGTCTCAGCTGCCGCTTCACAAAAGAGGTACCAAGACTGCACCGAATGAGCACGCAAGCAGCAACAGGAGTGGGAGAAGGAGGAGGCGGCAGCAGTGG|  |  |  |  |  |  |  |  |  |  |  |  |  |  |  |  |  |  |  |  |  |  |  |  |  |  |  |  |  |  |  |  | | --- | --- | --- | --- | --- | --- | --- | --- | --- | --- | --- | --- | --- | --- | --- | --- | --- | --- | --- | --- | --- | --- | --- | --- | --- | --- | --- | --- | --- | --- | --- | --- | | Gorilla gorGor1 Supercontig\_0005127 23443 23586 - **AT** | TCTTGATTCCTTCCTTGGGCTTTTCATCTGCCACGGCAAGCGCCTAAGTCTCAGCTGCCGCTTCACAAAAGAGGTACCAAGACTGCACCGAATGAGCACGCAAGCAGCAACAGGAGTGGGAGAAGAAGGAGGCGGCAGCAGTGG|  |  |  |  |  |  |  |  |  |  |  |  |  |  |  |  |  |  |  |  |  |  |  |  | | --- | --- | --- | --- | --- | --- | --- | --- | --- | --- | --- | --- | --- | --- | --- | --- | --- | --- | --- | --- | --- | --- | --- | --- | | Orangutan ponAbe2 chr3 113864078 113864221 + **AT** | TCTTGATTCCTTCCTTGGGCTTTTCGTCTGCCACGGCAAGCACCTGAGTCTCAGCTGCCGCTTCACAAAAGAGGTACCAAGACTGCACCAAATGAGCATGCAAGCAGCAACAGGAGTGGGAGAAGAAGGAGGCGGCAGCAGTGG|  |  |  |  |  |  |  |  |  |  |  |  |  |  |  |  | | --- | --- | --- | --- | --- | --- | --- | --- | --- | --- | --- | --- | --- | --- | --- | --- | | Rhesus rheMac2 chr2 175563822 175563965 - **AT** | TCTTGATTCTTTCCTTGGGCTTTTTGTCTGCCATGGCAGGCGCCGGAGTCTCAGCTGCCGCTTCACAAAAGAGGTACCAAGTCTGCACCGAATGAGTACATAAGCAGCACCAGGAGTGGAAGAAGAAGGAGGCGGCAGCGGTGG|  |  |  |  |  |  |  |  | | --- | --- | --- | --- | --- | --- | --- | --- | | Baboon papHam1 scaffold2033 183208 183351 + **AT** | TCTTGATTCTTTCCTTGGGCTTTTTGTCTGCCATGGCAGGCGCCGGAGTCTCAGCTGCCGCTTCACAAAAGAGGTACCAAGTCTGCACCGAATGAGTACATAAGCAGCACCAGGAGAGGAAGAAGAAGGAGGCGGCAGCGGTGG | | | | | | | | | | | | | | | | | | | | | | | | | | | | | | | | | | | | | | | | | | | | | | | | | |

**Alignment** (splice site sequences are in lowercase)  

```
Human      agTCTCGATTCCTTCCTTGGGCTTTTCATCTGCCACGGCAAGCGCCTAAGTCTCAGCTGCCGCTTCACAAAAGAGGTACC
Chimp      .....T..........................................................................
Gorilla    .....T..........................................................................
Orangutan  .....T.....................G...............A...G................................
Rhesus     .....T.....T..............TG.......T....G.....GG................................
Baboon     .....T.....T..............TG.......T....G.....GG................................

Human      AAGACTGCACCGAATGAGCACGCAAGCAGCAACAGGAGTGGGAGAAGAAGGAGGCGGCAGCAGTGGgt
Chimp      ...............................................G..................a.
Gorilla    ..................................................................a.
Orangutan  ...........A........T.............................................a.
Rhesus     ...T..............T..AT........C.........A...................G....a.
Baboon     ...T..............T..AT........C......A..A...................G....a.
```

---

## 11. uc011bql.1\_1\_17

**Summary**  

|  |  |  |  |  |  |  |  |  |  |  |  |  |  |  |  |  |  |  |  |  |  |  |  |  |  |
| --- | --- | --- | --- | --- | --- | --- | --- | --- | --- | --- | --- | --- | --- | --- | --- | --- | --- | --- | --- | --- | --- | --- | --- | --- | --- |
| No Exon ID Position (hg19) Dir Human donor Chimp donor Category Usage Gene symbol Protein accession mRNA accession Gene title Note|  |  |  |  |  |  |  |  |  |  |  |  |  | | --- | --- | --- | --- | --- | --- | --- | --- | --- | --- | --- | --- | --- | | 11 uc011bql.1\_1\_17 chr3:180633316 + GT GG (D7) exonization; novel start alternative FXR1 B4DXZ6 AK302197.1 fragile X mental retardation, autosomal homolog 1  | | | | | | | | | | | | | | | | | | | | | | | | | |

**Orthologs**  

|  |  |  |  |  |  |  |  |  |  |  |  |  |  |  |  |  |  |  |  |  |  |  |  |  |  |  |  |  |  |  |  |  |  |  |  |  |  |  |  |  |  |  |  |  |  |  |  |  |  |  |  |  |  |  |  |  |  |  |  |  |  |  |  |  |  |  |  |  |  |  |  |  |  |  |  |  |  |  |  |
| --- | --- | --- | --- | --- | --- | --- | --- | --- | --- | --- | --- | --- | --- | --- | --- | --- | --- | --- | --- | --- | --- | --- | --- | --- | --- | --- | --- | --- | --- | --- | --- | --- | --- | --- | --- | --- | --- | --- | --- | --- | --- | --- | --- | --- | --- | --- | --- | --- | --- | --- | --- | --- | --- | --- | --- | --- | --- | --- | --- | --- | --- | --- | --- | --- | --- | --- | --- | --- | --- | --- | --- | --- | --- | --- | --- | --- | --- | --- | --- |
| Species Assembly Chromosome Exon start Exon end Dir Donor Exon sequence|  |  |  |  |  |  |  |  |  |  |  |  |  |  |  |  |  |  |  |  |  |  |  |  |  |  |  |  |  |  |  |  |  |  |  |  |  |  |  |  |  |  |  |  |  |  |  |  |  |  |  |  |  |  |  |  |  |  |  |  |  |  |  |  |  |  |  |  |  |  |  |  | | --- | --- | --- | --- | --- | --- | --- | --- | --- | --- | --- | --- | --- | --- | --- | --- | --- | --- | --- | --- | --- | --- | --- | --- | --- | --- | --- | --- | --- | --- | --- | --- | --- | --- | --- | --- | --- | --- | --- | --- | --- | --- | --- | --- | --- | --- | --- | --- | --- | --- | --- | --- | --- | --- | --- | --- | --- | --- | --- | --- | --- | --- | --- | --- | --- | --- | --- | --- | --- | --- | --- | --- | | Human hg19 chr3 180633305 180633316 + **GT** | ATGGTCACTGAG|  |  |  |  |  |  |  |  |  |  |  |  |  |  |  |  |  |  |  |  |  |  |  |  |  |  |  |  |  |  |  |  |  |  |  |  |  |  |  |  |  |  |  |  |  |  |  |  |  |  |  |  |  |  |  |  |  |  |  |  |  |  |  |  | | --- | --- | --- | --- | --- | --- | --- | --- | --- | --- | --- | --- | --- | --- | --- | --- | --- | --- | --- | --- | --- | --- | --- | --- | --- | --- | --- | --- | --- | --- | --- | --- | --- | --- | --- | --- | --- | --- | --- | --- | --- | --- | --- | --- | --- | --- | --- | --- | --- | --- | --- | --- | --- | --- | --- | --- | --- | --- | --- | --- | --- | --- | --- | --- | | Chimp panTro2 chr3 186336886 186336897 + **GG** | ATGGTCACTGAG|  |  |  |  |  |  |  |  |  |  |  |  |  |  |  |  |  |  |  |  |  |  |  |  |  |  |  |  |  |  |  |  |  |  |  |  |  |  |  |  |  |  |  |  |  |  |  |  |  |  |  |  |  |  |  |  | | --- | --- | --- | --- | --- | --- | --- | --- | --- | --- | --- | --- | --- | --- | --- | --- | --- | --- | --- | --- | --- | --- | --- | --- | --- | --- | --- | --- | --- | --- | --- | --- | --- | --- | --- | --- | --- | --- | --- | --- | --- | --- | --- | --- | --- | --- | --- | --- | --- | --- | --- | --- | --- | --- | --- | --- | | Gorilla gorGor1 Supercontig\_0019635 7658 7669 + **GG** | ATGGTCACTGAG|  |  |  |  |  |  |  |  |  |  |  |  |  |  |  |  |  |  |  |  |  |  |  |  |  |  |  |  |  |  |  |  |  |  |  |  |  |  |  |  |  |  |  |  |  |  |  |  | | --- | --- | --- | --- | --- | --- | --- | --- | --- | --- | --- | --- | --- | --- | --- | --- | --- | --- | --- | --- | --- | --- | --- | --- | --- | --- | --- | --- | --- | --- | --- | --- | --- | --- | --- | --- | --- | --- | --- | --- | --- | --- | --- | --- | --- | --- | --- | --- | | Orangutan ponAbe2 chr3 184639389 184639400 + **GG** | ATGGTCACTGAG|  |  |  |  |  |  |  |  |  |  |  |  |  |  |  |  |  |  |  |  |  |  |  |  |  |  |  |  |  |  |  |  |  |  |  |  |  |  |  |  | | --- | --- | --- | --- | --- | --- | --- | --- | --- | --- | --- | --- | --- | --- | --- | --- | --- | --- | --- | --- | --- | --- | --- | --- | --- | --- | --- | --- | --- | --- | --- | --- | --- | --- | --- | --- | --- | --- | --- | --- | | Rhesus rheMac2 chr2 105814365 105814376 - **GG** | ATGGTCACTGAG|  |  |  |  |  |  |  |  |  |  |  |  |  |  |  |  |  |  |  |  |  |  |  |  |  |  |  |  |  |  |  |  | | --- | --- | --- | --- | --- | --- | --- | --- | --- | --- | --- | --- | --- | --- | --- | --- | --- | --- | --- | --- | --- | --- | --- | --- | --- | --- | --- | --- | --- | --- | --- | --- | | Baboon papHam1 scaffold3875 59827 59838 + **GG** | ATGGTCACTGAG|  |  |  |  |  |  |  |  |  |  |  |  |  |  |  |  |  |  |  |  |  |  |  |  | | --- | --- | --- | --- | --- | --- | --- | --- | --- | --- | --- | --- | --- | --- | --- | --- | --- | --- | --- | --- | --- | --- | --- | --- | | Tarsier tarSyr1 scaffold\_15029 616 627 + **GG** | ACGATCAGGGAA|  |  |  |  |  |  |  |  |  |  |  |  |  |  |  |  | | --- | --- | --- | --- | --- | --- | --- | --- | --- | --- | --- | --- | --- | --- | --- | --- | | Lemur micMur1 scaffold\_16499 4965 4976 + **GG** | GTGGTTAGTGAA|  |  |  |  |  |  |  |  | | --- | --- | --- | --- | --- | --- | --- | --- | | Dog canFam2 chr34 17141587 17141598 + **GG** | GTGGTCAGTGAG | | | | | | | | | | | | | | | | | | | | | | | | | | | | | | | | | | | | | | | | | | | | | | | | | | | | | | | | | | | | | | | | | | | | | | |

**Alignment** (splice site sequences are in lowercase)  

```
Human      ATGGTCACTGAGgt
Chimp      .............g
Gorilla    .............g
Orangutan  .............g
Rhesus     .............g
Baboon     .............g
Tarsier    .C.A...GG..A.g
Lemur      G....T.G...A.g
Dog        G......G.....g
```

---

## 12. uc011cfo.1\_2\_11

**Summary**  

|  |  |  |  |  |  |  |  |  |  |  |  |  |  |  |  |  |  |  |  |  |  |  |  |  |  |
| --- | --- | --- | --- | --- | --- | --- | --- | --- | --- | --- | --- | --- | --- | --- | --- | --- | --- | --- | --- | --- | --- | --- | --- | --- | --- |
| No Exon ID Position (hg19) Dir Human donor Chimp donor Category Usage Gene symbol Protein accession mRNA accession Gene title Note|  |  |  |  |  |  |  |  |  |  |  |  |  | | --- | --- | --- | --- | --- | --- | --- | --- | --- | --- | --- | --- | --- | | 12 uc011cfo.1\_2\_11 chr4:109683977 - GT GA (D2) shift; increase; frameshift alternative AGXT2L1 NP\_001140099.1 NM\_001146627.1 alanine-glyoxylate aminotransferase 2-like 1  | | | | | | | | | | | | | | | | | | | | | | | | | |

**Orthologs**  

|  |  |  |  |  |  |  |  |  |  |  |  |  |  |  |  |  |  |  |  |  |  |  |  |  |  |  |  |  |  |  |  |  |  |  |  |  |  |  |  |  |  |  |  |  |  |  |  |  |  |  |  |  |  |  |  |  |  |  |  |  |  |  |  |  |  |  |  |  |  |  |  |
| --- | --- | --- | --- | --- | --- | --- | --- | --- | --- | --- | --- | --- | --- | --- | --- | --- | --- | --- | --- | --- | --- | --- | --- | --- | --- | --- | --- | --- | --- | --- | --- | --- | --- | --- | --- | --- | --- | --- | --- | --- | --- | --- | --- | --- | --- | --- | --- | --- | --- | --- | --- | --- | --- | --- | --- | --- | --- | --- | --- | --- | --- | --- | --- | --- | --- | --- | --- | --- | --- | --- | --- |
| Species Assembly Chromosome Exon start Exon end Dir Donor Exon sequence|  |  |  |  |  |  |  |  |  |  |  |  |  |  |  |  |  |  |  |  |  |  |  |  |  |  |  |  |  |  |  |  |  |  |  |  |  |  |  |  |  |  |  |  |  |  |  |  |  |  |  |  |  |  |  |  |  |  |  |  |  |  |  |  | | --- | --- | --- | --- | --- | --- | --- | --- | --- | --- | --- | --- | --- | --- | --- | --- | --- | --- | --- | --- | --- | --- | --- | --- | --- | --- | --- | --- | --- | --- | --- | --- | --- | --- | --- | --- | --- | --- | --- | --- | --- | --- | --- | --- | --- | --- | --- | --- | --- | --- | --- | --- | --- | --- | --- | --- | --- | --- | --- | --- | --- | --- | --- | --- | | Human hg19 chr4 109683977 109683977 - **GT** | A|  |  |  |  |  |  |  |  |  |  |  |  |  |  |  |  |  |  |  |  |  |  |  |  |  |  |  |  |  |  |  |  |  |  |  |  |  |  |  |  |  |  |  |  |  |  |  |  |  |  |  |  |  |  |  |  | | --- | --- | --- | --- | --- | --- | --- | --- | --- | --- | --- | --- | --- | --- | --- | --- | --- | --- | --- | --- | --- | --- | --- | --- | --- | --- | --- | --- | --- | --- | --- | --- | --- | --- | --- | --- | --- | --- | --- | --- | --- | --- | --- | --- | --- | --- | --- | --- | --- | --- | --- | --- | --- | --- | --- | --- | | Chimp panTro2 chr4 112188172 112188172 - **GA** | A|  |  |  |  |  |  |  |  |  |  |  |  |  |  |  |  |  |  |  |  |  |  |  |  |  |  |  |  |  |  |  |  |  |  |  |  |  |  |  |  |  |  |  |  |  |  |  |  | | --- | --- | --- | --- | --- | --- | --- | --- | --- | --- | --- | --- | --- | --- | --- | --- | --- | --- | --- | --- | --- | --- | --- | --- | --- | --- | --- | --- | --- | --- | --- | --- | --- | --- | --- | --- | --- | --- | --- | --- | --- | --- | --- | --- | --- | --- | --- | --- | | Orangutan ponAbe2 chr4 113396428 113396428 - **GA** | A|  |  |  |  |  |  |  |  |  |  |  |  |  |  |  |  |  |  |  |  |  |  |  |  |  |  |  |  |  |  |  |  |  |  |  |  |  |  |  |  | | --- | --- | --- | --- | --- | --- | --- | --- | --- | --- | --- | --- | --- | --- | --- | --- | --- | --- | --- | --- | --- | --- | --- | --- | --- | --- | --- | --- | --- | --- | --- | --- | --- | --- | --- | --- | --- | --- | --- | --- | | Rhesus rheMac2 chr5 101674608 101674608 - **GA** | A|  |  |  |  |  |  |  |  |  |  |  |  |  |  |  |  |  |  |  |  |  |  |  |  |  |  |  |  |  |  |  |  | | --- | --- | --- | --- | --- | --- | --- | --- | --- | --- | --- | --- | --- | --- | --- | --- | --- | --- | --- | --- | --- | --- | --- | --- | --- | --- | --- | --- | --- | --- | --- | --- | | Baboon papHam1 scaffold22475 31476 31476 - **GA** | A|  |  |  |  |  |  |  |  |  |  |  |  |  |  |  |  |  |  |  |  |  |  |  |  | | --- | --- | --- | --- | --- | --- | --- | --- | --- | --- | --- | --- | --- | --- | --- | --- | --- | --- | --- | --- | --- | --- | --- | --- | | Marmoset calJac1 Contig5520 56017 56017 + **GA** | A|  |  |  |  |  |  |  |  |  |  |  |  |  |  |  |  | | --- | --- | --- | --- | --- | --- | --- | --- | --- | --- | --- | --- | --- | --- | --- | --- | | Tarsier tarSyr1 scaffold\_14592 25640 25640 - **GC** | G|  |  |  |  |  |  |  |  | | --- | --- | --- | --- | --- | --- | --- | --- | | Cow bosTau4 chr6 17968714 17968714 + **GA** | A | | | | | | | | | | | | | | | | | | | | | | | | | | | | | | | | | | | | | | | | | | | | | | | | | | | | | | | | | | | | | | | |

**Alignment** (splice site sequences are in lowercase)  

```
Human      gaAgt
Chimp      ....a
Orangutan  ....a
Rhesus     ....a
Baboon     ....a
Marmoset   ....a
Tarsier    ..G.c
Cow        ....a
```

---

## 13. uc003ldj.2\_17\_18

**Summary**  

|  |  |  |  |  |  |  |  |  |  |  |  |  |  |  |  |  |  |  |  |  |  |  |  |  |  |
| --- | --- | --- | --- | --- | --- | --- | --- | --- | --- | --- | --- | --- | --- | --- | --- | --- | --- | --- | --- | --- | --- | --- | --- | --- | --- |
| No Exon ID Position (hg19) Dir Human donor Chimp donor Category Usage Gene symbol Protein accession mRNA accession Gene title Note|  |  |  |  |  |  |  |  |  |  |  |  |  | | --- | --- | --- | --- | --- | --- | --- | --- | --- | --- | --- | --- | --- | | 13 uc003ldj.2\_17\_18 chr5:138268647 + GT GG (D6) exonization; frameshift alternative CTNNA1 P35221-2 U03100.1 catenin (cadherin-associated protein), alpha 1, 102kDa  | | | | | | | | | | | | | | | | | | | | | | | | | |

**Orthologs**  

|  |  |  |  |  |  |  |  |  |  |  |  |  |  |  |  |  |  |  |  |  |  |  |  |  |  |  |  |  |  |  |  |  |  |  |  |  |  |  |  |  |  |  |  |  |  |  |  |  |  |  |  |  |  |  |  |
| --- | --- | --- | --- | --- | --- | --- | --- | --- | --- | --- | --- | --- | --- | --- | --- | --- | --- | --- | --- | --- | --- | --- | --- | --- | --- | --- | --- | --- | --- | --- | --- | --- | --- | --- | --- | --- | --- | --- | --- | --- | --- | --- | --- | --- | --- | --- | --- | --- | --- | --- | --- | --- | --- | --- | --- |
| Species Assembly Chromosome Exon start Exon end Dir Donor Exon sequence|  |  |  |  |  |  |  |  |  |  |  |  |  |  |  |  |  |  |  |  |  |  |  |  |  |  |  |  |  |  |  |  |  |  |  |  |  |  |  |  |  |  |  |  |  |  |  |  | | --- | --- | --- | --- | --- | --- | --- | --- | --- | --- | --- | --- | --- | --- | --- | --- | --- | --- | --- | --- | --- | --- | --- | --- | --- | --- | --- | --- | --- | --- | --- | --- | --- | --- | --- | --- | --- | --- | --- | --- | --- | --- | --- | --- | --- | --- | --- | --- | | Human hg19 chr5 138268577 138268647 + **GT** | AACTGTGACACCTGCGGGGCACTGCAAGGGCTGAAAGGCTGGCCTCCTCCCCTTTGCTGGCCACTCACTGG|  |  |  |  |  |  |  |  |  |  |  |  |  |  |  |  |  |  |  |  |  |  |  |  |  |  |  |  |  |  |  |  |  |  |  |  |  |  |  |  | | --- | --- | --- | --- | --- | --- | --- | --- | --- | --- | --- | --- | --- | --- | --- | --- | --- | --- | --- | --- | --- | --- | --- | --- | --- | --- | --- | --- | --- | --- | --- | --- | --- | --- | --- | --- | --- | --- | --- | --- | | Chimp panTro2 chr5 140668915 140668985 + **GG** | AACTGTGACACCTGGGGGGCACTGCAAGGGCTGAAAGGCTGGCCTCCTCCCCTTTGCTGGCCGCTCACTGG|  |  |  |  |  |  |  |  |  |  |  |  |  |  |  |  |  |  |  |  |  |  |  |  |  |  |  |  |  |  |  |  | | --- | --- | --- | --- | --- | --- | --- | --- | --- | --- | --- | --- | --- | --- | --- | --- | --- | --- | --- | --- | --- | --- | --- | --- | --- | --- | --- | --- | --- | --- | --- | --- | | Orangutan ponAbe2 chr5 140491878 140491948 + **GA** | AACTGTGACACCTGGGGGGCACTGCAGGGGCTGAAAGGCTGGCCGCCTCCCCTTTGCTGGCCACTCACTGG|  |  |  |  |  |  |  |  |  |  |  |  |  |  |  |  |  |  |  |  |  |  |  |  | | --- | --- | --- | --- | --- | --- | --- | --- | --- | --- | --- | --- | --- | --- | --- | --- | --- | --- | --- | --- | --- | --- | --- | --- | | Rhesus rheMac2 chr6 135350520 135350590 + **GA** | AACTGTGACACGTGTGGGCCACTGCAAGGGCTGAAAGGCTGGCCTCCTCCCCTTTGCTGGCCACTCACTGG|  |  |  |  |  |  |  |  |  |  |  |  |  |  |  |  | | --- | --- | --- | --- | --- | --- | --- | --- | --- | --- | --- | --- | --- | --- | --- | --- | | Baboon papHam1 scaffold1426 129075 129145 + **GA** | AACTGTGACACGTGCGGGCCACTGCAAGGGCTGAAAGGCTGGCCTCCTCCCCTTTGCTGGCCACTCACTGG|  |  |  |  |  |  |  |  | | --- | --- | --- | --- | --- | --- | --- | --- | | Marmoset calJac1 Contig665 128601 128661 + **CA** | AACTGTGATCATTGCAAGGGCTGAAAGGCTGGCTCCCTCCCCTTTGCTGGCCACTCACTGG | | | | | | | | | | | | | | | | | | | | | | | | | | | | | | | | | | | | | | | | | | | | | | | | | |

**Alignment** (splice site sequences are in lowercase)  

```
Human      agAACTGTGACACCTGCGGGGCACTGCAAGGGCTGAAAGGCTGGCCTCCTCCCCTTTGCTGGCCACTCACTGGgt
Chimp      ................G...............................................G.........g
Orangutan  ................G...........G.................G...........................a
Rhesus     .............G..T...C.....................................................a
Baboon     .............G......C.....................................................a
Marmoset   ..........T----------..T.....................TC..........................ca
```

---

## 14. uc011dbw.1\_12\_13

**Summary**  

|  |  |  |  |  |  |  |  |  |  |  |  |  |  |  |  |  |  |  |  |  |  |  |  |  |  |
| --- | --- | --- | --- | --- | --- | --- | --- | --- | --- | --- | --- | --- | --- | --- | --- | --- | --- | --- | --- | --- | --- | --- | --- | --- | --- |
| No Exon ID Position (hg19) Dir Human donor Chimp donor Category Usage Gene symbol Protein accession mRNA accession Gene title Note|  |  |  |  |  |  |  |  |  |  |  |  |  | | --- | --- | --- | --- | --- | --- | --- | --- | --- | --- | --- | --- | --- | | 14 uc011dbw.1\_12\_13 chr5:146763517 + GT AT (D4) shift; decrease; frameshift alternative STK32A B7Z9H7 AK315942.1 serine/threonine-protein kinase 32A  | | | | | | | | | | | | | | | | | | | | | | | | | |

**Orthologs**  

|  |  |  |  |  |  |  |  |  |  |  |  |  |  |  |  |  |  |  |  |  |  |  |  |  |  |  |  |  |  |  |  |  |  |  |  |  |  |  |  |  |  |  |  |  |  |  |  |  |  |  |  |  |  |  |  |  |  |  |  |  |  |  |  |  |  |  |  |  |  |  |  |  |  |  |  |  |  |  |  |  |  |  |  |  |  |  |  |  |  |  |  |  |  |  |  |  |  |  |  |  |  |  |  |
| --- | --- | --- | --- | --- | --- | --- | --- | --- | --- | --- | --- | --- | --- | --- | --- | --- | --- | --- | --- | --- | --- | --- | --- | --- | --- | --- | --- | --- | --- | --- | --- | --- | --- | --- | --- | --- | --- | --- | --- | --- | --- | --- | --- | --- | --- | --- | --- | --- | --- | --- | --- | --- | --- | --- | --- | --- | --- | --- | --- | --- | --- | --- | --- | --- | --- | --- | --- | --- | --- | --- | --- | --- | --- | --- | --- | --- | --- | --- | --- | --- | --- | --- | --- | --- | --- | --- | --- | --- | --- | --- | --- | --- | --- | --- | --- | --- | --- | --- | --- | --- | --- | --- | --- |
| Species Assembly Chromosome Exon start Exon end Dir Donor Exon sequence|  |  |  |  |  |  |  |  |  |  |  |  |  |  |  |  |  |  |  |  |  |  |  |  |  |  |  |  |  |  |  |  |  |  |  |  |  |  |  |  |  |  |  |  |  |  |  |  |  |  |  |  |  |  |  |  |  |  |  |  |  |  |  |  |  |  |  |  |  |  |  |  |  |  |  |  |  |  |  |  |  |  |  |  |  |  |  |  |  |  |  |  |  |  |  |  | | --- | --- | --- | --- | --- | --- | --- | --- | --- | --- | --- | --- | --- | --- | --- | --- | --- | --- | --- | --- | --- | --- | --- | --- | --- | --- | --- | --- | --- | --- | --- | --- | --- | --- | --- | --- | --- | --- | --- | --- | --- | --- | --- | --- | --- | --- | --- | --- | --- | --- | --- | --- | --- | --- | --- | --- | --- | --- | --- | --- | --- | --- | --- | --- | --- | --- | --- | --- | --- | --- | --- | --- | --- | --- | --- | --- | --- | --- | --- | --- | --- | --- | --- | --- | --- | --- | --- | --- | --- | --- | --- | --- | --- | --- | --- | --- | | Human hg19 chr5 146763453 146763517 + **GT** | AGTAAACAGGGACTTTAACAAAAGACAACCAAATCTAGCCTTGGAACAAACCAAAGACCCACAAG|  |  |  |  |  |  |  |  |  |  |  |  |  |  |  |  |  |  |  |  |  |  |  |  |  |  |  |  |  |  |  |  |  |  |  |  |  |  |  |  |  |  |  |  |  |  |  |  |  |  |  |  |  |  |  |  |  |  |  |  |  |  |  |  |  |  |  |  |  |  |  |  |  |  |  |  |  |  |  |  |  |  |  |  |  |  |  |  | | --- | --- | --- | --- | --- | --- | --- | --- | --- | --- | --- | --- | --- | --- | --- | --- | --- | --- | --- | --- | --- | --- | --- | --- | --- | --- | --- | --- | --- | --- | --- | --- | --- | --- | --- | --- | --- | --- | --- | --- | --- | --- | --- | --- | --- | --- | --- | --- | --- | --- | --- | --- | --- | --- | --- | --- | --- | --- | --- | --- | --- | --- | --- | --- | --- | --- | --- | --- | --- | --- | --- | --- | --- | --- | --- | --- | --- | --- | --- | --- | --- | --- | --- | --- | --- | --- | --- | --- | | Chimp panTro2 chr5 149311251 149311315 + **AT** | AGTAAACAGGGACTTTAACAAAAGACAACCAAATCTAGCCTTGGAACAAACCAAAGACCCACAAG|  |  |  |  |  |  |  |  |  |  |  |  |  |  |  |  |  |  |  |  |  |  |  |  |  |  |  |  |  |  |  |  |  |  |  |  |  |  |  |  |  |  |  |  |  |  |  |  |  |  |  |  |  |  |  |  |  |  |  |  |  |  |  |  |  |  |  |  |  |  |  |  |  |  |  |  |  |  |  |  | | --- | --- | --- | --- | --- | --- | --- | --- | --- | --- | --- | --- | --- | --- | --- | --- | --- | --- | --- | --- | --- | --- | --- | --- | --- | --- | --- | --- | --- | --- | --- | --- | --- | --- | --- | --- | --- | --- | --- | --- | --- | --- | --- | --- | --- | --- | --- | --- | --- | --- | --- | --- | --- | --- | --- | --- | --- | --- | --- | --- | --- | --- | --- | --- | --- | --- | --- | --- | --- | --- | --- | --- | --- | --- | --- | --- | --- | --- | --- | --- | | Gorilla gorGor1 Supercontig\_0000921 16959 17023 + **AT** | AGTAAACAGGGACTTTAACAAAAGACAACCAAATCTAGCCTTGGAACAAACCAAAGACCCACAAG|  |  |  |  |  |  |  |  |  |  |  |  |  |  |  |  |  |  |  |  |  |  |  |  |  |  |  |  |  |  |  |  |  |  |  |  |  |  |  |  |  |  |  |  |  |  |  |  |  |  |  |  |  |  |  |  |  |  |  |  |  |  |  |  |  |  |  |  |  |  |  |  | | --- | --- | --- | --- | --- | --- | --- | --- | --- | --- | --- | --- | --- | --- | --- | --- | --- | --- | --- | --- | --- | --- | --- | --- | --- | --- | --- | --- | --- | --- | --- | --- | --- | --- | --- | --- | --- | --- | --- | --- | --- | --- | --- | --- | --- | --- | --- | --- | --- | --- | --- | --- | --- | --- | --- | --- | --- | --- | --- | --- | --- | --- | --- | --- | --- | --- | --- | --- | --- | --- | --- | --- | | Orangutan ponAbe2 chr5 149092632 149092696 + **AT** | AGTAAAAAGGGACTTTAACAAAAGACAACCAAATCTAGCCTTGGAACAAACCAAAGACCCACAAG|  |  |  |  |  |  |  |  |  |  |  |  |  |  |  |  |  |  |  |  |  |  |  |  |  |  |  |  |  |  |  |  |  |  |  |  |  |  |  |  |  |  |  |  |  |  |  |  |  |  |  |  |  |  |  |  |  |  |  |  |  |  |  |  | | --- | --- | --- | --- | --- | --- | --- | --- | --- | --- | --- | --- | --- | --- | --- | --- | --- | --- | --- | --- | --- | --- | --- | --- | --- | --- | --- | --- | --- | --- | --- | --- | --- | --- | --- | --- | --- | --- | --- | --- | --- | --- | --- | --- | --- | --- | --- | --- | --- | --- | --- | --- | --- | --- | --- | --- | --- | --- | --- | --- | --- | --- | --- | --- | | Rhesus rheMac2 chr6 143841020 143841084 + **AT** | AGTAAAAAGGGACTTTAACAAAAGACAACCAAATCTAACCTTGGAACAAACCAAAGACCCACAAA|  |  |  |  |  |  |  |  |  |  |  |  |  |  |  |  |  |  |  |  |  |  |  |  |  |  |  |  |  |  |  |  |  |  |  |  |  |  |  |  |  |  |  |  |  |  |  |  |  |  |  |  |  |  |  |  | | --- | --- | --- | --- | --- | --- | --- | --- | --- | --- | --- | --- | --- | --- | --- | --- | --- | --- | --- | --- | --- | --- | --- | --- | --- | --- | --- | --- | --- | --- | --- | --- | --- | --- | --- | --- | --- | --- | --- | --- | --- | --- | --- | --- | --- | --- | --- | --- | --- | --- | --- | --- | --- | --- | --- | --- | | Baboon papHam1 scaffold3125 125202 125266 - **AT** | AGTAAAAAGGGACTTTAACAAAAGACAACCAAATCTAACCTTGGAACAAACCAAAGACCCACAAA|  |  |  |  |  |  |  |  |  |  |  |  |  |  |  |  |  |  |  |  |  |  |  |  |  |  |  |  |  |  |  |  |  |  |  |  |  |  |  |  |  |  |  |  |  |  |  |  | | --- | --- | --- | --- | --- | --- | --- | --- | --- | --- | --- | --- | --- | --- | --- | --- | --- | --- | --- | --- | --- | --- | --- | --- | --- | --- | --- | --- | --- | --- | --- | --- | --- | --- | --- | --- | --- | --- | --- | --- | --- | --- | --- | --- | --- | --- | --- | --- | | Marmoset calJac1 Contig231 456749 456813 + **AT** | AGTAAAAAGGGACTTTAATAAAAGACAACCAAATCTAGCCTTGGAACAAACCAAAGACCCACAAG|  |  |  |  |  |  |  |  |  |  |  |  |  |  |  |  |  |  |  |  |  |  |  |  |  |  |  |  |  |  |  |  |  |  |  |  |  |  |  |  | | --- | --- | --- | --- | --- | --- | --- | --- | --- | --- | --- | --- | --- | --- | --- | --- | --- | --- | --- | --- | --- | --- | --- | --- | --- | --- | --- | --- | --- | --- | --- | --- | --- | --- | --- | --- | --- | --- | --- | --- | | Tarsier tarSyr1 scaffold\_16673 20845 20909 + **AG** | AGTAAAAAGGGACTTTAATAAAAAGCAAGCAGACCTAGCCTCGGAACAAACCAAAGACCCACAAG|  |  |  |  |  |  |  |  |  |  |  |  |  |  |  |  |  |  |  |  |  |  |  |  |  |  |  |  |  |  |  |  | | --- | --- | --- | --- | --- | --- | --- | --- | --- | --- | --- | --- | --- | --- | --- | --- | --- | --- | --- | --- | --- | --- | --- | --- | --- | --- | --- | --- | --- | --- | --- | --- | | Lemur micMur1 scaffold\_1779 239748 239812 + **AA** | AGTAAAAAGGGACTTTAATAAAAGGCCAGCAAATCTAGCCTTGGAACAAACCAAAGACCCACAAG|  |  |  |  |  |  |  |  |  |  |  |  |  |  |  |  |  |  |  |  |  |  |  |  | | --- | --- | --- | --- | --- | --- | --- | --- | --- | --- | --- | --- | --- | --- | --- | --- | --- | --- | --- | --- | --- | --- | --- | --- | | Galago otoGar1 scaffold\_96106.1-140986 68083 68147 + **GA** | AGTAAAAAAGGACATTACTAAAAAGCAAGCACATCTAGCCTTGGAACAAACCAAAGACCCACAAG|  |  |  |  |  |  |  |  |  |  |  |  |  |  |  |  | | --- | --- | --- | --- | --- | --- | --- | --- | --- | --- | --- | --- | --- | --- | --- | --- | | Cow bosTau4 chrUn.004.7 772996 773060 - **GA** | AGTCAAAAGGGACTTTAATAAAAGACAAGCAAATCAAGCCTTGGAACAAACCAAAGACCCAGAAG|  |  |  |  |  |  |  |  | | --- | --- | --- | --- | --- | --- | --- | --- | | Dog canFam2 chr2 44530855 44530919 + **AA** | AGTAAAAAGGGACTTTACTAAAAGACCAGCTAACCTAGCTCTGGAACAAACCAAAGACCCACAAG | | | | | | | | | | | | | | | | | | | | | | | | | | | | | | | | | | | | | | | | | | | | | | | | | | | | | | | | | | | | | | | | | | | | | | | | | | | | | | | | | | | | | | | | | | | |

**Alignment** (splice site sequences are in lowercase)  

```
Human      agAGTAAACAGGGACTTTAACAAAAGACAACCAAATCTAGCCTTGGAACAAACCAAAGACCCACAAGgt
Chimp      ...................................................................a.
Gorilla    ...................................................................a.
Orangutan  ........A..........................................................a.
Rhesus     ........A..............................A..........................Aa.
Baboon     ........A..............................A..........................Aa.
Marmoset   ........A...........T..............................................a.
Tarsier    ........A...........T....AG...G..G.C.......C.......................ag
Lemur      ........A...........T.....G.C.G....................................aa
Galago     ........A.A....A...CT....AG...G..C..................................a
Cow        .....C..A...........T.........G......A.........................G....a
Dog        ........A..........CT.......C.G.T..C.....TC........................aa
```

---

## 15. uc003pne.3\_14\_15

**Summary**  

|  |  |  |  |  |  |  |  |  |  |  |  |  |  |  |  |  |  |  |  |  |  |  |  |  |  |
| --- | --- | --- | --- | --- | --- | --- | --- | --- | --- | --- | --- | --- | --- | --- | --- | --- | --- | --- | --- | --- | --- | --- | --- | --- | --- |
| No Exon ID Position (hg19) Dir Human donor Chimp donor Category Usage Gene symbol Protein accession mRNA accession Gene title Note|  |  |  |  |  |  |  |  |  |  |  |  |  | | --- | --- | --- | --- | --- | --- | --- | --- | --- | --- | --- | --- | --- | | 15 uc003pne.3\_14\_15 chr6:90338942 + GT GC (D3) shift; decrease; inframe alternative ANKRD6 NP\_055757.3 NM\_014942.4 ankyrin repeat domain-containing protein 6  | | | | | | | | | | | | | | | | | | | | | | | | | |

**Orthologs**  

|  |  |  |  |  |  |  |  |  |  |  |  |  |  |  |  |  |  |  |  |  |  |  |  |  |  |  |  |  |  |  |  |  |  |  |  |  |  |  |  |  |  |  |  |  |  |  |  |  |  |  |  |  |  |  |  |  |  |  |  |  |  |  |  |  |  |  |  |  |  |  |  |  |  |  |  |  |  |  |  |  |  |  |  |  |  |  |  |  |  |  |  |  |  |  |  |
| --- | --- | --- | --- | --- | --- | --- | --- | --- | --- | --- | --- | --- | --- | --- | --- | --- | --- | --- | --- | --- | --- | --- | --- | --- | --- | --- | --- | --- | --- | --- | --- | --- | --- | --- | --- | --- | --- | --- | --- | --- | --- | --- | --- | --- | --- | --- | --- | --- | --- | --- | --- | --- | --- | --- | --- | --- | --- | --- | --- | --- | --- | --- | --- | --- | --- | --- | --- | --- | --- | --- | --- | --- | --- | --- | --- | --- | --- | --- | --- | --- | --- | --- | --- | --- | --- | --- | --- | --- | --- | --- | --- | --- | --- | --- | --- |
| Species Assembly Chromosome Exon start Exon end Dir Donor Exon sequence|  |  |  |  |  |  |  |  |  |  |  |  |  |  |  |  |  |  |  |  |  |  |  |  |  |  |  |  |  |  |  |  |  |  |  |  |  |  |  |  |  |  |  |  |  |  |  |  |  |  |  |  |  |  |  |  |  |  |  |  |  |  |  |  |  |  |  |  |  |  |  |  |  |  |  |  |  |  |  |  |  |  |  |  |  |  |  |  | | --- | --- | --- | --- | --- | --- | --- | --- | --- | --- | --- | --- | --- | --- | --- | --- | --- | --- | --- | --- | --- | --- | --- | --- | --- | --- | --- | --- | --- | --- | --- | --- | --- | --- | --- | --- | --- | --- | --- | --- | --- | --- | --- | --- | --- | --- | --- | --- | --- | --- | --- | --- | --- | --- | --- | --- | --- | --- | --- | --- | --- | --- | --- | --- | --- | --- | --- | --- | --- | --- | --- | --- | --- | --- | --- | --- | --- | --- | --- | --- | --- | --- | --- | --- | --- | --- | --- | --- | | Human hg19 chr6 90338831 90338942 + **GT** | ATATCCTTGGTGGATGAATTAAAAACCTGGTGCATGTTAAAGATTCAGAATCTGGAGCAGAAGCTTTCTGGAGATTCTAGGGCCTGCAGAGCTAAATCCACACCATCTACTT|  |  |  |  |  |  |  |  |  |  |  |  |  |  |  |  |  |  |  |  |  |  |  |  |  |  |  |  |  |  |  |  |  |  |  |  |  |  |  |  |  |  |  |  |  |  |  |  |  |  |  |  |  |  |  |  |  |  |  |  |  |  |  |  |  |  |  |  |  |  |  |  |  |  |  |  |  |  |  |  | | --- | --- | --- | --- | --- | --- | --- | --- | --- | --- | --- | --- | --- | --- | --- | --- | --- | --- | --- | --- | --- | --- | --- | --- | --- | --- | --- | --- | --- | --- | --- | --- | --- | --- | --- | --- | --- | --- | --- | --- | --- | --- | --- | --- | --- | --- | --- | --- | --- | --- | --- | --- | --- | --- | --- | --- | --- | --- | --- | --- | --- | --- | --- | --- | --- | --- | --- | --- | --- | --- | --- | --- | --- | --- | --- | --- | --- | --- | --- | --- | | Chimp panTro2 chr6 90886765 90886876 + **GC** | ATATCCTTGGTGGATGAATTAAAAACCTGGTGCATGTTAAAGATTCAGAATCTGGAGCAGAAGCTTTCTGGAGATTCTAGGGCCTGCAGAGCTAAATCCACACCATCTACTT|  |  |  |  |  |  |  |  |  |  |  |  |  |  |  |  |  |  |  |  |  |  |  |  |  |  |  |  |  |  |  |  |  |  |  |  |  |  |  |  |  |  |  |  |  |  |  |  |  |  |  |  |  |  |  |  |  |  |  |  |  |  |  |  |  |  |  |  |  |  |  |  | | --- | --- | --- | --- | --- | --- | --- | --- | --- | --- | --- | --- | --- | --- | --- | --- | --- | --- | --- | --- | --- | --- | --- | --- | --- | --- | --- | --- | --- | --- | --- | --- | --- | --- | --- | --- | --- | --- | --- | --- | --- | --- | --- | --- | --- | --- | --- | --- | --- | --- | --- | --- | --- | --- | --- | --- | --- | --- | --- | --- | --- | --- | --- | --- | --- | --- | --- | --- | --- | --- | --- | --- | | Gorilla gorGor1 Supercontig\_0048960 13459 13570 + **GC** | ATATCCTTGGTGGATGAATTAAAAACCTGGTGCATGTTAAAGATTCAGAATCTGGAGCAGAAGCTTTCTGGAGATTCTAGGGCCTGCAGAGCTAAATCCACACCATCTACTT|  |  |  |  |  |  |  |  |  |  |  |  |  |  |  |  |  |  |  |  |  |  |  |  |  |  |  |  |  |  |  |  |  |  |  |  |  |  |  |  |  |  |  |  |  |  |  |  |  |  |  |  |  |  |  |  |  |  |  |  |  |  |  |  | | --- | --- | --- | --- | --- | --- | --- | --- | --- | --- | --- | --- | --- | --- | --- | --- | --- | --- | --- | --- | --- | --- | --- | --- | --- | --- | --- | --- | --- | --- | --- | --- | --- | --- | --- | --- | --- | --- | --- | --- | --- | --- | --- | --- | --- | --- | --- | --- | --- | --- | --- | --- | --- | --- | --- | --- | --- | --- | --- | --- | --- | --- | --- | --- | | Orangutan ponAbe2 chr6 90593512 90593623 + **GC** | ATATCCTTGGTGGATGAATTAAAAACCTGGTGCATGTTAAAGATTCAGAATCTGGAGCAGAAGCTTTCTGGAGATTCTAGAGCCTGCAGAGCTAAATCCACACCGTCTACTT|  |  |  |  |  |  |  |  |  |  |  |  |  |  |  |  |  |  |  |  |  |  |  |  |  |  |  |  |  |  |  |  |  |  |  |  |  |  |  |  |  |  |  |  |  |  |  |  |  |  |  |  |  |  |  |  | | --- | --- | --- | --- | --- | --- | --- | --- | --- | --- | --- | --- | --- | --- | --- | --- | --- | --- | --- | --- | --- | --- | --- | --- | --- | --- | --- | --- | --- | --- | --- | --- | --- | --- | --- | --- | --- | --- | --- | --- | --- | --- | --- | --- | --- | --- | --- | --- | --- | --- | --- | --- | --- | --- | --- | --- | | Rhesus rheMac2 chr4 85489977 85490088 + **GC** | ATCTCCTTGGTGGATGAATTAAAAACTTGGTGCATGTTAAAGATTCAGAATCTGGAGCAGAAGCTTTCTGGAGATTCTAGGGCCTGCAGAGCTAAATCCACACCATCTACTT|  |  |  |  |  |  |  |  |  |  |  |  |  |  |  |  |  |  |  |  |  |  |  |  |  |  |  |  |  |  |  |  |  |  |  |  |  |  |  |  |  |  |  |  |  |  |  |  | | --- | --- | --- | --- | --- | --- | --- | --- | --- | --- | --- | --- | --- | --- | --- | --- | --- | --- | --- | --- | --- | --- | --- | --- | --- | --- | --- | --- | --- | --- | --- | --- | --- | --- | --- | --- | --- | --- | --- | --- | --- | --- | --- | --- | --- | --- | --- | --- | | Baboon papHam1 scaffold21683 3049 3160 + **GC** | ATCTCCTTGGTGGATGAATTAAAAACTTGGTGCATGTTAAAGATTCAGAATCTGGAGCAGAAGCTTTCTGGAGATTCTAGGGCCTGCAGAGCTAAATCCACACCATCTACTT|  |  |  |  |  |  |  |  |  |  |  |  |  |  |  |  |  |  |  |  |  |  |  |  |  |  |  |  |  |  |  |  |  |  |  |  |  |  |  |  | | --- | --- | --- | --- | --- | --- | --- | --- | --- | --- | --- | --- | --- | --- | --- | --- | --- | --- | --- | --- | --- | --- | --- | --- | --- | --- | --- | --- | --- | --- | --- | --- | --- | --- | --- | --- | --- | --- | --- | --- | | Marmoset calJac1 Contig44 453429 453540 + **GC** | ATATCCTTGGTGGATGAATTAAAAACCTGGTGCATGTTAAAGATTCAGAATCTGGAGCTGAAGCTTTCTGGAGATTCTAGGGCCTCCAGAGCTAAATCCACACCATCTACTT|  |  |  |  |  |  |  |  |  |  |  |  |  |  |  |  |  |  |  |  |  |  |  |  |  |  |  |  |  |  |  |  | | --- | --- | --- | --- | --- | --- | --- | --- | --- | --- | --- | --- | --- | --- | --- | --- | --- | --- | --- | --- | --- | --- | --- | --- | --- | --- | --- | --- | --- | --- | --- | --- | | Lemur micMur1 scaffold\_2057 140957 141068 + **GC** | ACATCCTTGGTGGATGAATTAAAAACCTGGTGCATGTTAAAGATTCAGAATCTGGAGCTGAAGCTTTCTGGAGATTCTAGAGCCTCCAGGGCTAAATCCACACCATCTACTT|  |  |  |  |  |  |  |  |  |  |  |  |  |  |  |  |  |  |  |  |  |  |  |  | | --- | --- | --- | --- | --- | --- | --- | --- | --- | --- | --- | --- | --- | --- | --- | --- | --- | --- | --- | --- | --- | --- | --- | --- | | Mouse mm9 chr4 32895674 32895785 - **CT** | ATGTCTTTGGTAGATGAACTAAAAGCCTGGTGCATGTTGAAGATCCAGAGTCTGGAGCTAAGACTCTCTGGAGAGTCTCGGACCTTCAGAGCTAAATCCACACCACCTCCGT|  |  |  |  |  |  |  |  |  |  |  |  |  |  |  |  | | --- | --- | --- | --- | --- | --- | --- | --- | --- | --- | --- | --- | --- | --- | --- | --- | | Cow bosTau4 chr9 63226762 63226873 - **GC** | ATGTCCTTGGTGGATGAATTAAAAACCTGGTGCATGTTAAAGATTCAGAATCTGGAGCTGAAGCTTTCTGGAGATTCTAGGGCCTCCAGGACTAAATCCACACCGTCCACTT|  |  |  |  |  |  |  |  | | --- | --- | --- | --- | --- | --- | --- | --- | | Dog canFam2 chr12 51842219 51842330 + **GC** | ATGTCATTAGTGGATGAATTAAAAACCTGGTGCATGTTAAAGATTCAGAATCTGGAGCTGAAGCTTTCTGGAGATTCTAGGGCCTCTAGGACCAAATCCACACCATCTACTT | | | | | | | | | | | | | | | | | | | | | | | | | | | | | | | | | | | | | | | | | | | | | | | | | | | | | | | | | | | | | | | | | | | | | | | | | | | | | | | | | | | | |

**Alignment** (splice site sequences are in lowercase)  

```
Human      agATATCCTTGGTGGATGAATTAAAAACCTGGTGCATGTTAAAGATTCAGAATCTGGAGCAGAAGCTTTCTGGAGATTCT
Chimp      ................................................................................
Gorilla    ................................................................................
Orangutan  ................................................................................
Rhesus     ....C.......................T...................................................
Baboon     ....C.......................T...................................................
Marmoset   ............................................................T...................
Lemur      ...C........................................................T...................
Mouse      ....G..T.....A......C.....G.............G.....C....G........TA.GA..C........G...
Cow        ....G.......................................................T...................
Dog        ....G..A..A.................................................T...................

Human      AGGGCCTGCAGAGCTAAATCCACACCATCTACTTgt
Chimp      ...................................c
Gorilla    ...................................c
Orangutan  ..A.......................G........c
Rhesus     ...................................c
Baboon     ...................................c
Marmoset   .......C...........................c
Lemur      ..A....C...G.......................c
Mouse      C..A...T...................C..C.G.c.
Cow        .......C...GA.............G..C.....c
Dog        .......CT..GA.C....................c
```

---

## 16. uc011kma.1\_7\_8

**Summary**  

|  |  |  |  |  |  |  |  |  |  |  |  |  |  |  |  |  |  |  |  |  |  |  |  |  |  |
| --- | --- | --- | --- | --- | --- | --- | --- | --- | --- | --- | --- | --- | --- | --- | --- | --- | --- | --- | --- | --- | --- | --- | --- | --- | --- |
| No Exon ID Position (hg19) Dir Human donor Chimp donor Category Usage Gene symbol Protein accession mRNA accession Gene title Note|  |  |  |  |  |  |  |  |  |  |  |  |  | | --- | --- | --- | --- | --- | --- | --- | --- | --- | --- | --- | --- | --- | | 16 uc011kma.1\_7\_8 chr7:107254177 + GT AT (D6) exonization; frameshift alternative BCAP29 NP\_001008405.1 NM\_001008405.2 B-cell receptor-associated protein 29  | | | | | | | | | | | | | | | | | | | | | | | | | |

**Orthologs**  

|  |  |  |  |  |  |  |  |  |  |  |  |  |  |  |  |  |  |  |  |  |  |  |  |  |  |  |  |  |  |  |  |  |  |  |  |  |  |  |  |  |  |  |  |  |  |  |  |  |  |  |  |  |  |  |  |  |  |  |  |  |  |  |  |  |  |  |  |  |  |  |  |  |  |  |  |  |  |  |  |
| --- | --- | --- | --- | --- | --- | --- | --- | --- | --- | --- | --- | --- | --- | --- | --- | --- | --- | --- | --- | --- | --- | --- | --- | --- | --- | --- | --- | --- | --- | --- | --- | --- | --- | --- | --- | --- | --- | --- | --- | --- | --- | --- | --- | --- | --- | --- | --- | --- | --- | --- | --- | --- | --- | --- | --- | --- | --- | --- | --- | --- | --- | --- | --- | --- | --- | --- | --- | --- | --- | --- | --- | --- | --- | --- | --- | --- | --- | --- | --- |
| Species Assembly Chromosome Exon start Exon end Dir Donor Exon sequence|  |  |  |  |  |  |  |  |  |  |  |  |  |  |  |  |  |  |  |  |  |  |  |  |  |  |  |  |  |  |  |  |  |  |  |  |  |  |  |  |  |  |  |  |  |  |  |  |  |  |  |  |  |  |  |  |  |  |  |  |  |  |  |  |  |  |  |  |  |  |  |  | | --- | --- | --- | --- | --- | --- | --- | --- | --- | --- | --- | --- | --- | --- | --- | --- | --- | --- | --- | --- | --- | --- | --- | --- | --- | --- | --- | --- | --- | --- | --- | --- | --- | --- | --- | --- | --- | --- | --- | --- | --- | --- | --- | --- | --- | --- | --- | --- | --- | --- | --- | --- | --- | --- | --- | --- | --- | --- | --- | --- | --- | --- | --- | --- | --- | --- | --- | --- | --- | --- | --- | --- | | Human hg19 chr7 107254099 107254177 + **GT** | CATTCCAGTTTTGGTGAATTTTTAAGCAAAAGAAGCCACAAAAATGGCAGCATTGGAAAACAGACTGGTTCTAGAAAAG|  |  |  |  |  |  |  |  |  |  |  |  |  |  |  |  |  |  |  |  |  |  |  |  |  |  |  |  |  |  |  |  |  |  |  |  |  |  |  |  |  |  |  |  |  |  |  |  |  |  |  |  |  |  |  |  |  |  |  |  |  |  |  |  | | --- | --- | --- | --- | --- | --- | --- | --- | --- | --- | --- | --- | --- | --- | --- | --- | --- | --- | --- | --- | --- | --- | --- | --- | --- | --- | --- | --- | --- | --- | --- | --- | --- | --- | --- | --- | --- | --- | --- | --- | --- | --- | --- | --- | --- | --- | --- | --- | --- | --- | --- | --- | --- | --- | --- | --- | --- | --- | --- | --- | --- | --- | --- | --- | | Chimp panTro2 chr7 107565757 107565839 + **AT** | CATTCCAGTTTTGGTGAATTTTTAAGCAAAAGAATTAAGCCACAAAAATGGCAGCATTGGAAAACAGACTGGTTCTAGAAAAG|  |  |  |  |  |  |  |  |  |  |  |  |  |  |  |  |  |  |  |  |  |  |  |  |  |  |  |  |  |  |  |  |  |  |  |  |  |  |  |  |  |  |  |  |  |  |  |  |  |  |  |  |  |  |  |  | | --- | --- | --- | --- | --- | --- | --- | --- | --- | --- | --- | --- | --- | --- | --- | --- | --- | --- | --- | --- | --- | --- | --- | --- | --- | --- | --- | --- | --- | --- | --- | --- | --- | --- | --- | --- | --- | --- | --- | --- | --- | --- | --- | --- | --- | --- | --- | --- | --- | --- | --- | --- | --- | --- | --- | --- | | Orangutan ponAbe2 chr7 103782359 103782441 + **AT** | CATTCCAGTTTTGGTGAATTTTTAAGCAAAAGAATTAAGCCACAAAAATGGCAGCATTGGAAAACAGACTGGTTCTAGAAAAG|  |  |  |  |  |  |  |  |  |  |  |  |  |  |  |  |  |  |  |  |  |  |  |  |  |  |  |  |  |  |  |  |  |  |  |  |  |  |  |  |  |  |  |  |  |  |  |  | | --- | --- | --- | --- | --- | --- | --- | --- | --- | --- | --- | --- | --- | --- | --- | --- | --- | --- | --- | --- | --- | --- | --- | --- | --- | --- | --- | --- | --- | --- | --- | --- | --- | --- | --- | --- | --- | --- | --- | --- | --- | --- | --- | --- | --- | --- | --- | --- | | Rhesus rheMac2 chr3 145037787 145037869 + **AT** | CATTCCAGTTTTGGTGAACTTTTAAGCAAAAGAATTAAGCCACAAAAATGGCAGCACTGGAAAACAGACGGGTTCTAGAAAAG|  |  |  |  |  |  |  |  |  |  |  |  |  |  |  |  |  |  |  |  |  |  |  |  |  |  |  |  |  |  |  |  |  |  |  |  |  |  |  |  | | --- | --- | --- | --- | --- | --- | --- | --- | --- | --- | --- | --- | --- | --- | --- | --- | --- | --- | --- | --- | --- | --- | --- | --- | --- | --- | --- | --- | --- | --- | --- | --- | --- | --- | --- | --- | --- | --- | --- | --- | | Baboon papHam1 scaffold4836 11208 11290 + **AT** | CATTCCAGTTTTGGTGAACTTTTAAGCAAAAGAATTAAGCCACAAAAATGGCAGCACTGGAAAACAGACGGGTTCTAGAAAAG|  |  |  |  |  |  |  |  |  |  |  |  |  |  |  |  |  |  |  |  |  |  |  |  |  |  |  |  |  |  |  |  | | --- | --- | --- | --- | --- | --- | --- | --- | --- | --- | --- | --- | --- | --- | --- | --- | --- | --- | --- | --- | --- | --- | --- | --- | --- | --- | --- | --- | --- | --- | --- | --- | | Marmoset calJac1 Contig2609 211589 211671 - **TT** | CATTCCAGTTTTAGTGAATCTTTAAGCAAAAGAAGTAAGCCACAAAAATGGCAGCATTGGAAAACAGACTGGTTCTAGAAGAG|  |  |  |  |  |  |  |  |  |  |  |  |  |  |  |  |  |  |  |  |  |  |  |  | | --- | --- | --- | --- | --- | --- | --- | --- | --- | --- | --- | --- | --- | --- | --- | --- | --- | --- | --- | --- | --- | --- | --- | --- | | Lemur micMur1 scaffold\_1185 102388 102470 + **TT** | CATTCCAGATTTGGTGAAAGTTTAACTCAAAGAATTGATCCACAAAACTGGAAGCATTGAAAAAGATACTAGTTCTAGAAAAA|  |  |  |  |  |  |  |  |  |  |  |  |  |  |  |  | | --- | --- | --- | --- | --- | --- | --- | --- | --- | --- | --- | --- | --- | --- | --- | --- | | Galago otoGar1 scaffold\_94154.1-94172 87428 87510 - **TT** | CATTCCAGATTTGGTGAAGGCTTAAGCAAAAAAATTGAACCACAAAACCAACAGCATTGAAAAAGAGACTAGTTCTAGAAAAA|  |  |  |  |  |  |  |  | | --- | --- | --- | --- | --- | --- | --- | --- | | Dog canFam2 chr18 15962859 15962941 - **TT** | TGTTCTAGTTCTAGTGAAATTCTAAGCAAAAGAATTGAGCCACAAAAATGGCAGCATTGAAAAATGGGCTGTTTCTGTAAACA | | | | | | | | | | | | | | | | | | | | | | | | | | | | | | | | | | | | | | | | | | | | | | | | | | | | | | | | | | | | | | | | | | | | | | |

**Alignment** (splice site sequences are in lowercase)  

```
Human      agCATTCCAGTTTTGGTGAATTTTTAAGCAAAAGA----AGCCACAAAAATGGCAGCATTGGAAAACAGACTGGTTCTAG
Chimp      ...................................ATTA.........................................
Orangutan  ...................................ATTA.........................................
Rhesus     ....................C..............ATTA...................C............G........
Baboon     ....................C..............ATTA...................C............G........
Marmoset   ..............A......C.............AGTA.........................................
Lemur      ..........A.........AG.....CTC.....ATTG.T........C...A.......A....G.T...A.......
Galago     ..........A.........GGC..........A.ATTG.A........CCAA........A....G.....A.......
Dog        t.TG...T....C.A.....A..C...........ATTG......................A....TG.G...T....GT

Human      AAAAGgt
Chimp      .....a.
Orangutan  .....a.
Rhesus     .....a.
Baboon     .....a.
Marmoset   ..G..t.
Lemur      ....At.
Galago     ....At.
Dog        ...CAt.
```

---

## 17. uc003wii.2\_2\_10

**Summary**  

|  |  |  |  |  |  |  |  |  |  |  |  |  |  |  |  |  |  |  |  |  |  |  |  |  |  |
| --- | --- | --- | --- | --- | --- | --- | --- | --- | --- | --- | --- | --- | --- | --- | --- | --- | --- | --- | --- | --- | --- | --- | --- | --- | --- |
| No Exon ID Position (hg19) Dir Human donor Chimp donor Category Usage Gene symbol Protein accession mRNA accession Gene title Note|  |  |  |  |  |  |  |  |  |  |  |  |  | | --- | --- | --- | --- | --- | --- | --- | --- | --- | --- | --- | --- | --- | | 17 uc003wii.2\_2\_10 chr7:150725998 + GT TT (D5) exonization; inframe alternative ABCB8 Q6ZRM3 AK128129.1 ATP-binding cassette, sub-family B (MDR/TAP), member 8  | | | | | | | | | | | | | | | | | | | | | | | | | |

**Orthologs**  

|  |  |  |  |  |  |  |  |  |  |  |  |  |  |  |  |  |  |  |  |  |  |  |  |  |  |  |  |  |  |  |  |  |  |  |  |  |  |  |  |  |  |  |  |  |  |  |  |  |  |  |  |  |  |  |  |
| --- | --- | --- | --- | --- | --- | --- | --- | --- | --- | --- | --- | --- | --- | --- | --- | --- | --- | --- | --- | --- | --- | --- | --- | --- | --- | --- | --- | --- | --- | --- | --- | --- | --- | --- | --- | --- | --- | --- | --- | --- | --- | --- | --- | --- | --- | --- | --- | --- | --- | --- | --- | --- | --- | --- | --- |
| Species Assembly Chromosome Exon start Exon end Dir Donor Exon sequence|  |  |  |  |  |  |  |  |  |  |  |  |  |  |  |  |  |  |  |  |  |  |  |  |  |  |  |  |  |  |  |  |  |  |  |  |  |  |  |  |  |  |  |  |  |  |  |  | | --- | --- | --- | --- | --- | --- | --- | --- | --- | --- | --- | --- | --- | --- | --- | --- | --- | --- | --- | --- | --- | --- | --- | --- | --- | --- | --- | --- | --- | --- | --- | --- | --- | --- | --- | --- | --- | --- | --- | --- | --- | --- | --- | --- | --- | --- | --- | --- | | Human hg19 chr7 150725888 150725998 + **GT** | GAGGCTCCTGCGTCTGCAGCCGCGTGTCAGCCAGAAGGAGGGGACGCTCGGGGTCAGTGACCACGCCCAGTCCGCACTCCCGACCGGGGGTCCCCTTTCCTGGCCCTGGAG|  |  |  |  |  |  |  |  |  |  |  |  |  |  |  |  |  |  |  |  |  |  |  |  |  |  |  |  |  |  |  |  |  |  |  |  |  |  |  |  | | --- | --- | --- | --- | --- | --- | --- | --- | --- | --- | --- | --- | --- | --- | --- | --- | --- | --- | --- | --- | --- | --- | --- | --- | --- | --- | --- | --- | --- | --- | --- | --- | --- | --- | --- | --- | --- | --- | --- | --- | | Chimp panTro2 chr7 151562169 151562279 + **TT** | GAGGCTCCTGCGTCTGCAGCCGCGTGTCAGCCAAAAGGAGGGGACGCTCGGGGTCAGTGACCACGCCCAGTCCGCACTCCCGACCAGGGGTCCCCTTTCCTGGCCCTGGAG|  |  |  |  |  |  |  |  |  |  |  |  |  |  |  |  |  |  |  |  |  |  |  |  |  |  |  |  |  |  |  |  | | --- | --- | --- | --- | --- | --- | --- | --- | --- | --- | --- | --- | --- | --- | --- | --- | --- | --- | --- | --- | --- | --- | --- | --- | --- | --- | --- | --- | --- | --- | --- | --- | | Gorilla gorGor1 Supercontig\_0007123 33062 33172 - **TT** | GAGGCTCCTGCGTCTGCAGCCGCGTGTCAGCCAGAAGGAGGGGACGCTCGGGGTCAGTGACCACGCCCAGTCCGCACTCCCGACCAGGGGTCCCCTTTCCTGGCCCTGGAG|  |  |  |  |  |  |  |  |  |  |  |  |  |  |  |  |  |  |  |  |  |  |  |  | | --- | --- | --- | --- | --- | --- | --- | --- | --- | --- | --- | --- | --- | --- | --- | --- | --- | --- | --- | --- | --- | --- | --- | --- | | Orangutan ponAbe2 chr7 148712369 148712479 + **TT** | GAGGCTCCTGCGTCTGCAGCCGCGTGTCAGCCAGAAGGAGGGGACGCTCGGGGTCAGTGACCACGCCCAGTCAGCACTCCCGACCAGGGGTCGCCTTTCCTGGCCCTGGAG|  |  |  |  |  |  |  |  |  |  |  |  |  |  |  |  | | --- | --- | --- | --- | --- | --- | --- | --- | --- | --- | --- | --- | --- | --- | --- | --- | | Rhesus rheMac2 chr3 187973971 187974081 + **TT** | GAGGTTCCGGCGTCTGCATCGGCGTGTCAGCCAGAAGGAGGGAACGCTCCGGGTCTGTGACCACGCCCAGTCCGCACTCTCGACCAGGGGTCGCCTTTCCTGGACCCCGAG|  |  |  |  |  |  |  |  | | --- | --- | --- | --- | --- | --- | --- | --- | | Baboon papHam1 scaffold4613 132098 132208 - **TT** | AAGGTGCCGGCGTCTGCATCGGCGTGTCAGCCAGAAGGAGGGAACGCTCCGGGTCTGTGACCACGCCCAGTCCGCACTCTCGACCAGGGGTCGGCTTTCCTGGACCCCGAG | | | | | | | | | | | | | | | | | | | | | | | | | | | | | | | | | | | | | | | | | | | | | | | | | |

**Alignment** (splice site sequences are in lowercase)  

```
Human      agGAGGCTCCTGCGTCTGCAGCCGCGTGTCAGCCAGAAGGAGGGGACGCTCGGGGTCAGTGACCACGCCCAGTCCGCACT
Chimp      ...................................A............................................
Gorilla    ................................................................................
Orangutan  ..........................................................................A.....
Rhesus     ......T...G.........T.G.....................A......C.....T......................
Baboon     ..A...TG..G.........T.G.....................A......C.....T......................

Human      CCCGACCGGGGGTCCCCTTTCCTGGCCCTGGAGgt
Chimp      .......A.........................t.
Gorilla    .......A.........................t.
Orangutan  .......A......G..................t.
Rhesus     .T.....A......G..........A..CC...t.
Baboon     .T.....A......GG.........A..CC...t.
```

---

## 18. uc003wjw.2\_11\_14

**Summary**  

|  |  |  |  |  |  |  |  |  |  |  |  |  |  |  |  |  |  |  |  |  |  |  |  |  |  |
| --- | --- | --- | --- | --- | --- | --- | --- | --- | --- | --- | --- | --- | --- | --- | --- | --- | --- | --- | --- | --- | --- | --- | --- | --- | --- |
| No Exon ID Position (hg19) Dir Human donor Chimp donor Category Usage Gene symbol Protein accession mRNA accession Gene title Note|  |  |  |  |  |  |  |  |  |  |  |  |  | | --- | --- | --- | --- | --- | --- | --- | --- | --- | --- | --- | --- | --- | | 18 uc003wjw.2\_11\_14 chr7:151071287 + GT GC (D3) shift; decrease; inframe alternative NUB1 NP\_057202.3 NM\_016118.4 negative regulator of ubiquitin-like proteins 1  | | | | | | | | | | | | | | | | | | | | | | | | | |

**Orthologs**  

|  |  |  |  |  |  |  |  |  |  |  |  |  |  |  |  |  |  |  |  |  |  |  |  |  |  |  |  |  |  |  |  |  |  |  |  |  |  |  |  |  |  |  |  |  |  |  |  |  |  |  |  |  |  |  |  |  |  |  |  |  |  |  |  |  |  |  |  |  |  |  |  |  |  |  |  |  |  |  |  |  |  |  |  |  |  |  |  |  |  |  |  |  |  |  |  |  |  |  |  |  |  |  |  |
| --- | --- | --- | --- | --- | --- | --- | --- | --- | --- | --- | --- | --- | --- | --- | --- | --- | --- | --- | --- | --- | --- | --- | --- | --- | --- | --- | --- | --- | --- | --- | --- | --- | --- | --- | --- | --- | --- | --- | --- | --- | --- | --- | --- | --- | --- | --- | --- | --- | --- | --- | --- | --- | --- | --- | --- | --- | --- | --- | --- | --- | --- | --- | --- | --- | --- | --- | --- | --- | --- | --- | --- | --- | --- | --- | --- | --- | --- | --- | --- | --- | --- | --- | --- | --- | --- | --- | --- | --- | --- | --- | --- | --- | --- | --- | --- | --- | --- | --- | --- | --- | --- | --- | --- |
| Species Assembly Chromosome Exon start Exon end Dir Donor Exon sequence|  |  |  |  |  |  |  |  |  |  |  |  |  |  |  |  |  |  |  |  |  |  |  |  |  |  |  |  |  |  |  |  |  |  |  |  |  |  |  |  |  |  |  |  |  |  |  |  |  |  |  |  |  |  |  |  |  |  |  |  |  |  |  |  |  |  |  |  |  |  |  |  |  |  |  |  |  |  |  |  |  |  |  |  |  |  |  |  |  |  |  |  |  |  |  |  | | --- | --- | --- | --- | --- | --- | --- | --- | --- | --- | --- | --- | --- | --- | --- | --- | --- | --- | --- | --- | --- | --- | --- | --- | --- | --- | --- | --- | --- | --- | --- | --- | --- | --- | --- | --- | --- | --- | --- | --- | --- | --- | --- | --- | --- | --- | --- | --- | --- | --- | --- | --- | --- | --- | --- | --- | --- | --- | --- | --- | --- | --- | --- | --- | --- | --- | --- | --- | --- | --- | --- | --- | --- | --- | --- | --- | --- | --- | --- | --- | --- | --- | --- | --- | --- | --- | --- | --- | --- | --- | --- | --- | --- | --- | --- | --- | | Human hg19 chr7 151071183 151071287 + **GT** | GAACTGGCCCAAATAAGGAAGGAGGAAAAAGAGAAGAAAAGACGCCGCCTCGAGAACATCAGGTTTCTGAAAGGGATGGGCTACTCCACGCACGCGGCCCAGCAG|  |  |  |  |  |  |  |  |  |  |  |  |  |  |  |  |  |  |  |  |  |  |  |  |  |  |  |  |  |  |  |  |  |  |  |  |  |  |  |  |  |  |  |  |  |  |  |  |  |  |  |  |  |  |  |  |  |  |  |  |  |  |  |  |  |  |  |  |  |  |  |  |  |  |  |  |  |  |  |  |  |  |  |  |  |  |  |  | | --- | --- | --- | --- | --- | --- | --- | --- | --- | --- | --- | --- | --- | --- | --- | --- | --- | --- | --- | --- | --- | --- | --- | --- | --- | --- | --- | --- | --- | --- | --- | --- | --- | --- | --- | --- | --- | --- | --- | --- | --- | --- | --- | --- | --- | --- | --- | --- | --- | --- | --- | --- | --- | --- | --- | --- | --- | --- | --- | --- | --- | --- | --- | --- | --- | --- | --- | --- | --- | --- | --- | --- | --- | --- | --- | --- | --- | --- | --- | --- | --- | --- | --- | --- | --- | --- | --- | --- | | Chimp panTro2 chr7 151916784 151916888 + **GC** | GAACTGGCCCAAATAAGGAAGGAGGAAAAAGAGAAGAAAAGACGCCGCCTGGAGAACATCAGGTTTCTGAAAGGGATGGGTTACTCCACGCACGCGGCCCAGCAG|  |  |  |  |  |  |  |  |  |  |  |  |  |  |  |  |  |  |  |  |  |  |  |  |  |  |  |  |  |  |  |  |  |  |  |  |  |  |  |  |  |  |  |  |  |  |  |  |  |  |  |  |  |  |  |  |  |  |  |  |  |  |  |  |  |  |  |  |  |  |  |  |  |  |  |  |  |  |  |  | | --- | --- | --- | --- | --- | --- | --- | --- | --- | --- | --- | --- | --- | --- | --- | --- | --- | --- | --- | --- | --- | --- | --- | --- | --- | --- | --- | --- | --- | --- | --- | --- | --- | --- | --- | --- | --- | --- | --- | --- | --- | --- | --- | --- | --- | --- | --- | --- | --- | --- | --- | --- | --- | --- | --- | --- | --- | --- | --- | --- | --- | --- | --- | --- | --- | --- | --- | --- | --- | --- | --- | --- | --- | --- | --- | --- | --- | --- | --- | --- | | Gorilla gorGor1 Supercontig\_0005020 7567 7671 + **GC** | GAACTGGCCCAAATAAGGATGGAGGAAAAAGAGAAGAAAAGACGCCGCCTCGAGAACATCAGGTTTCTGGAAGGGATGGGCTACTCCACGCACGCGGCCCAGCAG|  |  |  |  |  |  |  |  |  |  |  |  |  |  |  |  |  |  |  |  |  |  |  |  |  |  |  |  |  |  |  |  |  |  |  |  |  |  |  |  |  |  |  |  |  |  |  |  |  |  |  |  |  |  |  |  |  |  |  |  |  |  |  |  |  |  |  |  |  |  |  |  | | --- | --- | --- | --- | --- | --- | --- | --- | --- | --- | --- | --- | --- | --- | --- | --- | --- | --- | --- | --- | --- | --- | --- | --- | --- | --- | --- | --- | --- | --- | --- | --- | --- | --- | --- | --- | --- | --- | --- | --- | --- | --- | --- | --- | --- | --- | --- | --- | --- | --- | --- | --- | --- | --- | --- | --- | --- | --- | --- | --- | --- | --- | --- | --- | --- | --- | --- | --- | --- | --- | --- | --- | | Orangutan ponAbe2 chr7 149040268 149040372 + **GC** | GAACTGGCCCAAATAAGGAAGGAGGAAAAAGAGAAGAAAAGACGCCGCCTCGAGAACATCAGGTGTCTGAAAGGGATGGGCTACTCCACGCACGCGGCCCAGCAG|  |  |  |  |  |  |  |  |  |  |  |  |  |  |  |  |  |  |  |  |  |  |  |  |  |  |  |  |  |  |  |  |  |  |  |  |  |  |  |  |  |  |  |  |  |  |  |  |  |  |  |  |  |  |  |  |  |  |  |  |  |  |  |  | | --- | --- | --- | --- | --- | --- | --- | --- | --- | --- | --- | --- | --- | --- | --- | --- | --- | --- | --- | --- | --- | --- | --- | --- | --- | --- | --- | --- | --- | --- | --- | --- | --- | --- | --- | --- | --- | --- | --- | --- | --- | --- | --- | --- | --- | --- | --- | --- | --- | --- | --- | --- | --- | --- | --- | --- | --- | --- | --- | --- | --- | --- | --- | --- | | Rhesus rheMac2 chr3 188328069 188328173 + **GC** | GAATTGGCCCAAATAAGGAAGGAGGAAAAAGAGAAGAAAAGACGCCGCCTCGAGAACATCAGGTCTCTGAAAGGGATGGGCTACTCCACGCACGCAGCCCAGCAG|  |  |  |  |  |  |  |  |  |  |  |  |  |  |  |  |  |  |  |  |  |  |  |  |  |  |  |  |  |  |  |  |  |  |  |  |  |  |  |  |  |  |  |  |  |  |  |  |  |  |  |  |  |  |  |  | | --- | --- | --- | --- | --- | --- | --- | --- | --- | --- | --- | --- | --- | --- | --- | --- | --- | --- | --- | --- | --- | --- | --- | --- | --- | --- | --- | --- | --- | --- | --- | --- | --- | --- | --- | --- | --- | --- | --- | --- | --- | --- | --- | --- | --- | --- | --- | --- | --- | --- | --- | --- | --- | --- | --- | --- | | Baboon papHam1 scaffold1041 24977 25081 - **GC** | GAATTGGCCCAAATAAGGAAGGAGGAAAAAGAGAAGAAAAGACGCCGCCTCGAGAACATCAGGTCTCTGAAAGGGATGGGCTACTCCACGCACGCAGCCCAGCAG|  |  |  |  |  |  |  |  |  |  |  |  |  |  |  |  |  |  |  |  |  |  |  |  |  |  |  |  |  |  |  |  |  |  |  |  |  |  |  |  |  |  |  |  |  |  |  |  | | --- | --- | --- | --- | --- | --- | --- | --- | --- | --- | --- | --- | --- | --- | --- | --- | --- | --- | --- | --- | --- | --- | --- | --- | --- | --- | --- | --- | --- | --- | --- | --- | --- | --- | --- | --- | --- | --- | --- | --- | --- | --- | --- | --- | --- | --- | --- | --- | | Marmoset calJac1 Contig3894 216054 216158 + **GC** | GAACTGGCCCAAATAAGGAAGGAGGAAAAAGAGAAGAAAAGACGCCACCTGGAGAAGATCAGGTCCCTGAAAGGGATGGGCTACTCCACGCACGCAGCCCAGCAG|  |  |  |  |  |  |  |  |  |  |  |  |  |  |  |  |  |  |  |  |  |  |  |  |  |  |  |  |  |  |  |  |  |  |  |  |  |  |  |  | | --- | --- | --- | --- | --- | --- | --- | --- | --- | --- | --- | --- | --- | --- | --- | --- | --- | --- | --- | --- | --- | --- | --- | --- | --- | --- | --- | --- | --- | --- | --- | --- | --- | --- | --- | --- | --- | --- | --- | --- | | Tarsier tarSyr1 scaffold\_70333 5770 5874 + **GC** | GAATTGGCCCAAATAAGGAAGGAAGAAAAAGAGAAGAAAAGAAACCGCCTGGAAAACATCAACGCCCTGAAGGTGATGGGCTACTCCACGCAGGCAGCCAAGCAG|  |  |  |  |  |  |  |  |  |  |  |  |  |  |  |  |  |  |  |  |  |  |  |  |  |  |  |  |  |  |  |  | | --- | --- | --- | --- | --- | --- | --- | --- | --- | --- | --- | --- | --- | --- | --- | --- | --- | --- | --- | --- | --- | --- | --- | --- | --- | --- | --- | --- | --- | --- | --- | --- | | Galago otoGar1 scaffold\_73616.1-11485 10469 10572 + **GC** | GAATTGGCCCAAATAAGGAAAGAGGAAAAGAGAAGAAAAGACGCCGCCTGGAGAACATCAACTCTCTGAAGTGCATGGGCTACTCCACACACGCAGCCAAGCAG|  |  |  |  |  |  |  |  |  |  |  |  |  |  |  |  |  |  |  |  |  |  |  |  | | --- | --- | --- | --- | --- | --- | --- | --- | --- | --- | --- | --- | --- | --- | --- | --- | --- | --- | --- | --- | --- | --- | --- | --- | | Mouse mm9 chr5 24212256 24212360 + **GC** | GAACTGGCCCAAATCCGGAAGGAAGAGAAGGAGAAAAGGAGACGCCGTCTGGAAAATGTCAACACTTTGAGAGGAATGGGCTACTCCACACAAGCTGCCAAACAA|  |  |  |  |  |  |  |  |  |  |  |  |  |  |  |  | | --- | --- | --- | --- | --- | --- | --- | --- | --- | --- | --- | --- | --- | --- | --- | --- | | Cow bosTau4 chr4 118220992 118221096 + **GC** | GAACTGGACCAAATAAAGAAGGAGGAAAGAGAGAAGAAAAAACGCCGCCTGGAGAACATTAACCATTTGAAAGGAATGGGCTACTCCATGCGAGCAGCCAGGCAG|  |  |  |  |  |  |  |  | | --- | --- | --- | --- | --- | --- | --- | --- | | Dog canFam2 chr16 18325869 18325972 + **GC** | GAATTGGCCCAATAATGAAAGAGGAAAAAGAGAAGAAAAGACGCCGGCTGGAGAACATAAACTCCTTGAAAGGAATGGGCTACTCTACTCATGCAGCCAAGCAG | | | | | | | | | | | | | | | | | | | | | | | | | | | | | | | | | | | | | | | | | | | | | | | | | | | | | | | | | | | | | | | | | | | | | | | | | | | | | | | | | | | | | | | | | | | |

**Alignment** (splice site sequences are in lowercase)  

```
Human      agGAACTGGCCCAAATAAGGAAGGAGGAAAAAGAGAAGAAAAGACGCCGCCTCGAGAACATCAGGTTTCTGAAAGGGATG
Chimp      ....................................................G...........................
Gorilla    .....................T.................................................G........
Orangutan  ..................................................................G.............
Rhesus     .....T............................................................C.............
Baboon     .....T............................................................C.............
Marmoset   ................................................A...G.....G.......CC............
Tarsier    .....T...................A..................AA......G..A.......ACGCC.....G.T....
Galago     .....T................A.....-.......................G..........AC.C......GT.C...
Mouse      ................CC.......A..G..G.....A.GG........T..G..A..TG...ACAC.T...G...A...
Cow        .........A........A...........G...........A.........G........T.ACCA.T.......A...
Dog        .....T........-...T...A..........................G..G........A.AC.CCT.......A...

Human      GGCTACTCCACGCACGCGGCCCAGCAGgt
Chimp      ..T.........................c
Gorilla    ............................c
Orangutan  ............................c
Rhesus     .................A..........c
Baboon     .................A..........c
Marmoset   .................A..........c
Tarsier    ..............G..A...A......c
Galago     ...........A.....A...A......c
Mouse      ...........A..A..T...A.A..A.c
Cow        ..........T..GA..A...AG.....c
Dog        ........T..T..T..A...A......c
```

---

## 19. uc003ybd.2\_5\_9

**Summary**  

|  |  |  |  |  |  |  |  |  |  |  |  |  |  |  |  |  |  |  |  |  |  |  |  |  |  |
| --- | --- | --- | --- | --- | --- | --- | --- | --- | --- | --- | --- | --- | --- | --- | --- | --- | --- | --- | --- | --- | --- | --- | --- | --- | --- |
| No Exon ID Position (hg19) Dir Human donor Chimp donor Category Usage Gene symbol Protein accession mRNA accession Gene title Note|  |  |  |  |  |  |  |  |  |  |  |  |  | | --- | --- | --- | --- | --- | --- | --- | --- | --- | --- | --- | --- | --- | | 19 uc003ybd.2\_5\_9 chr8:79601608 + GT AT (D1) shift; increase; inframe constitutive FAM164A NP\_057094.2 NM\_016010.2 family with sequence similarity 164, member A GTNGTN | | | | | | | | | | | | | | | | | | | | | | | | | |

**Orthologs**  

|  |  |  |  |  |  |  |  |  |  |  |  |  |  |  |  |  |  |  |  |  |  |  |  |  |  |  |  |  |  |  |  |  |  |  |  |  |  |  |  |  |  |  |  |  |  |  |  |  |  |  |  |  |  |  |  |  |  |  |  |  |  |  |  |  |  |  |  |  |  |  |  |  |  |  |  |  |  |  |  |
| --- | --- | --- | --- | --- | --- | --- | --- | --- | --- | --- | --- | --- | --- | --- | --- | --- | --- | --- | --- | --- | --- | --- | --- | --- | --- | --- | --- | --- | --- | --- | --- | --- | --- | --- | --- | --- | --- | --- | --- | --- | --- | --- | --- | --- | --- | --- | --- | --- | --- | --- | --- | --- | --- | --- | --- | --- | --- | --- | --- | --- | --- | --- | --- | --- | --- | --- | --- | --- | --- | --- | --- | --- | --- | --- | --- | --- | --- | --- | --- |
| Species Assembly Chromosome Exon start Exon end Dir Donor Exon sequence|  |  |  |  |  |  |  |  |  |  |  |  |  |  |  |  |  |  |  |  |  |  |  |  |  |  |  |  |  |  |  |  |  |  |  |  |  |  |  |  |  |  |  |  |  |  |  |  |  |  |  |  |  |  |  |  |  |  |  |  |  |  |  |  |  |  |  |  |  |  |  |  | | --- | --- | --- | --- | --- | --- | --- | --- | --- | --- | --- | --- | --- | --- | --- | --- | --- | --- | --- | --- | --- | --- | --- | --- | --- | --- | --- | --- | --- | --- | --- | --- | --- | --- | --- | --- | --- | --- | --- | --- | --- | --- | --- | --- | --- | --- | --- | --- | --- | --- | --- | --- | --- | --- | --- | --- | --- | --- | --- | --- | --- | --- | --- | --- | --- | --- | --- | --- | --- | --- | --- | --- | | Human hg19 chr8 79601457 79601608 + **GT** | ATTATATTCAATGTCCATATTGTCAGAGGAGATTCAATGAAAATGCAGCTGATAGACATATAAATTTCTGTAAAGAACAGGCAGCACGTATTAGTAATAAAGGGAAATTTTCTACAGATACCAAAGGAAAACCAACTTCTCGGACACAGGTG|  |  |  |  |  |  |  |  |  |  |  |  |  |  |  |  |  |  |  |  |  |  |  |  |  |  |  |  |  |  |  |  |  |  |  |  |  |  |  |  |  |  |  |  |  |  |  |  |  |  |  |  |  |  |  |  |  |  |  |  |  |  |  |  | | --- | --- | --- | --- | --- | --- | --- | --- | --- | --- | --- | --- | --- | --- | --- | --- | --- | --- | --- | --- | --- | --- | --- | --- | --- | --- | --- | --- | --- | --- | --- | --- | --- | --- | --- | --- | --- | --- | --- | --- | --- | --- | --- | --- | --- | --- | --- | --- | --- | --- | --- | --- | --- | --- | --- | --- | --- | --- | --- | --- | --- | --- | --- | --- | | Chimp panTro2 chr8 77244221 77244372 + **AT** | ATTATATTCAATGTCCATATTGTCAGAGGAGATTCAATGAAAATGCAGCTGATAGACATATAAATTTCTGTAAAGAACAGGCAGCACGTATTAGTAATAAAGGGAAATTTTCTACAGATACCAAAGGAAAACCAACTTCTCGGACACAGGTG|  |  |  |  |  |  |  |  |  |  |  |  |  |  |  |  |  |  |  |  |  |  |  |  |  |  |  |  |  |  |  |  |  |  |  |  |  |  |  |  |  |  |  |  |  |  |  |  |  |  |  |  |  |  |  |  | | --- | --- | --- | --- | --- | --- | --- | --- | --- | --- | --- | --- | --- | --- | --- | --- | --- | --- | --- | --- | --- | --- | --- | --- | --- | --- | --- | --- | --- | --- | --- | --- | --- | --- | --- | --- | --- | --- | --- | --- | --- | --- | --- | --- | --- | --- | --- | --- | --- | --- | --- | --- | --- | --- | --- | --- | | Gorilla gorGor1 Supercontig\_0250583 4671 4822 + **AT** | ATTATATTCAATGTCCATATTGTCAGAGGAGATTCAATGAAAATGCAGCTGATAGACATATAAATTTCTGTAAAGAACAGGCAGCACGTATTAGTAATAAAGGGAAATTTTCTACAGATACCAAAGGAAAACCAACTTCTCGGACACAGGTG|  |  |  |  |  |  |  |  |  |  |  |  |  |  |  |  |  |  |  |  |  |  |  |  |  |  |  |  |  |  |  |  |  |  |  |  |  |  |  |  |  |  |  |  |  |  |  |  | | --- | --- | --- | --- | --- | --- | --- | --- | --- | --- | --- | --- | --- | --- | --- | --- | --- | --- | --- | --- | --- | --- | --- | --- | --- | --- | --- | --- | --- | --- | --- | --- | --- | --- | --- | --- | --- | --- | --- | --- | --- | --- | --- | --- | --- | --- | --- | --- | | Orangutan ponAbe2 chr8 81520068 81520219 + **AT** | ATTATATTCAATGTCCATATTGTCAGAGGAGATTCAATGAAAATGCAGCTGATAGACATATAAATTTCTGTAAAGAACAGGCAGCACGTATTAGTAATAAAGGGAAATTTTCTACAGATACCAAAGGAAAACCAACTTCTCGGACACAGGTG|  |  |  |  |  |  |  |  |  |  |  |  |  |  |  |  |  |  |  |  |  |  |  |  |  |  |  |  |  |  |  |  |  |  |  |  |  |  |  |  | | --- | --- | --- | --- | --- | --- | --- | --- | --- | --- | --- | --- | --- | --- | --- | --- | --- | --- | --- | --- | --- | --- | --- | --- | --- | --- | --- | --- | --- | --- | --- | --- | --- | --- | --- | --- | --- | --- | --- | --- | | Rhesus rheMac2 chr8 81143885 81144036 + **AT** | ATTATATTCAGTGTCCATATTGTCAGAGGAGATTCAATGAAAATGCAGCTGATAGACATATAAATTTCTGTAAAGAACAGGCAGCACGTATTAGTAATAAAGGCAAATTTTCTACAGATACCAAAGGAAAACCAACTTCTCGGACACAGGTG|  |  |  |  |  |  |  |  |  |  |  |  |  |  |  |  |  |  |  |  |  |  |  |  |  |  |  |  |  |  |  |  | | --- | --- | --- | --- | --- | --- | --- | --- | --- | --- | --- | --- | --- | --- | --- | --- | --- | --- | --- | --- | --- | --- | --- | --- | --- | --- | --- | --- | --- | --- | --- | --- | | Baboon papHam1 scaffold11214 63688 63839 - **AT** | ATTATATTCAGTGTCCATATTGTCAGAGGAGATTCAATGAAAATGCAGCTGATAGACATATAAATTTCTGTAAAGAACAGGCAGCACGTATTAGTAATAAAGGCAAATTTTCTACAGATACCAAAGGAAAACCAACTTCTCGGACACAGGTG|  |  |  |  |  |  |  |  |  |  |  |  |  |  |  |  |  |  |  |  |  |  |  |  | | --- | --- | --- | --- | --- | --- | --- | --- | --- | --- | --- | --- | --- | --- | --- | --- | --- | --- | --- | --- | --- | --- | --- | --- | | Marmoset calJac1 Contig9735 32637 32788 + **AT** | ATTATATTCAGTGTCCATATTGTCAGAGGAGATTTAATGAAAATGCAGCTGATAGACATATAAATTTCTGTAAAGAACAGGCAGCACGTATTAGTAATAAAGGCAAATATTCTACAGATACCAAAGGAAAACCAACGTCTCGGACACAGGTG|  |  |  |  |  |  |  |  |  |  |  |  |  |  |  |  | | --- | --- | --- | --- | --- | --- | --- | --- | --- | --- | --- | --- | --- | --- | --- | --- | | Cow bosTau4 chr14 38934552 38934703 + **AT** | ACTATATTCAGTGTCCATATTGCCAGAGAAGATTTAATGAAAATGCAGCTGATAGACATATAAATTTCTGTAAAGAACAGGCAGCACGTATTAGTAATAAAGGCAAATTTTCTACTGATACCAAAGGAAAATCAACCTCTCGGACACAGGTA|  |  |  |  |  |  |  |  | | --- | --- | --- | --- | --- | --- | --- | --- | | Dog canFam2 chr29 29340088 29340239 + **AA** | ATTATATTCAGTGTCCATATTGCCAGAGGAGATTTAATGAAAATGCAGCTGATAGACATATTAACTTCTGTAAAGAACAGGCAGCACGTATTAGTAATAAAGGCAAATTTTCTACTGACACCAAAGGAAAGGCATCTTCTCGGACACAGGTG | | | | | | | | | | | | | | | | | | | | | | | | | | | | | | | | | | | | | | | | | | | | | | | | | | | | | | | | | | | | | | | | | | | | | | |

**Alignment** (splice site sequences are in lowercase)  

```
Human      agATTATATTCAATGTCCATATTGTCAGAGGAGATTCAATGAAAATGCAGCTGATAGACATATAAATTTCTGTAAAGAAC
Chimp      ................................................................................
Gorilla    ................................................................................
Orangutan  ................................................................................
Rhesus     ............G...................................................................
Baboon     ............G...................................................................
Marmoset   ............G.......................T...........................................
Cow        ...C........G...........C.....A.....T...........................................
Dog        ............G...........C...........T..........................T..C.............

Human      AGGCAGCACGTATTAGTAATAAAGGGAAATTTTCTACAGATACCAAAGGAAAACCAACTTCTCGGACACAGGTGgt
Chimp      ..........................................................................a.
Gorilla    ..........................................................................a.
Orangutan  ..........................................................................a.
Rhesus     .........................C................................................a.
Baboon     .........................C................................................a.
Marmoset   .........................C....A...........................G...............a.
Cow        .........................C...........T...............T....C..............Aa.
Dog        .........................C...........T..C...........GG..T.................aa
```

---

## 20. uc003yil.1\_11\_12

**Summary**  

|  |  |  |  |  |  |  |  |  |  |  |  |  |  |  |  |  |  |  |  |  |  |  |  |  |  |
| --- | --- | --- | --- | --- | --- | --- | --- | --- | --- | --- | --- | --- | --- | --- | --- | --- | --- | --- | --- | --- | --- | --- | --- | --- | --- |
| No Exon ID Position (hg19) Dir Human donor Chimp donor Category Usage Gene symbol Protein accession mRNA accession Gene title Note|  |  |  |  |  |  |  |  |  |  |  |  |  | | --- | --- | --- | --- | --- | --- | --- | --- | --- | --- | --- | --- | --- | | 20 uc003yil.1\_11\_12 chr8:99205613 - GT AT (D6) exonization; frameshift alternative NIPAL2 NP\_079035.1 NM\_024759.1 NIPA-like domain containing 2 dbSNP:rs3735887 | | | | | | | | | | | | | | | | | | | | | | | | | |

**Orthologs**  

|  |  |  |  |  |  |  |  |  |  |  |  |  |  |  |  |  |  |  |  |  |  |  |  |  |  |  |  |  |  |  |  |  |  |  |  |  |  |  |  |  |  |  |  |  |  |  |  |  |  |  |  |  |  |  |  |  |  |  |  |  |  |  |  |  |  |  |  |  |  |  |  |
| --- | --- | --- | --- | --- | --- | --- | --- | --- | --- | --- | --- | --- | --- | --- | --- | --- | --- | --- | --- | --- | --- | --- | --- | --- | --- | --- | --- | --- | --- | --- | --- | --- | --- | --- | --- | --- | --- | --- | --- | --- | --- | --- | --- | --- | --- | --- | --- | --- | --- | --- | --- | --- | --- | --- | --- | --- | --- | --- | --- | --- | --- | --- | --- | --- | --- | --- | --- | --- | --- | --- | --- |
| Species Assembly Chromosome Exon start Exon end Dir Donor Exon sequence|  |  |  |  |  |  |  |  |  |  |  |  |  |  |  |  |  |  |  |  |  |  |  |  |  |  |  |  |  |  |  |  |  |  |  |  |  |  |  |  |  |  |  |  |  |  |  |  |  |  |  |  |  |  |  |  |  |  |  |  |  |  |  |  | | --- | --- | --- | --- | --- | --- | --- | --- | --- | --- | --- | --- | --- | --- | --- | --- | --- | --- | --- | --- | --- | --- | --- | --- | --- | --- | --- | --- | --- | --- | --- | --- | --- | --- | --- | --- | --- | --- | --- | --- | --- | --- | --- | --- | --- | --- | --- | --- | --- | --- | --- | --- | --- | --- | --- | --- | --- | --- | --- | --- | --- | --- | --- | --- | | Human hg19 chr8 99205613 99205640 - **GT** | ACACAACCCCAGAGAGAAAGGCTTGGAG|  |  |  |  |  |  |  |  |  |  |  |  |  |  |  |  |  |  |  |  |  |  |  |  |  |  |  |  |  |  |  |  |  |  |  |  |  |  |  |  |  |  |  |  |  |  |  |  |  |  |  |  |  |  |  |  | | --- | --- | --- | --- | --- | --- | --- | --- | --- | --- | --- | --- | --- | --- | --- | --- | --- | --- | --- | --- | --- | --- | --- | --- | --- | --- | --- | --- | --- | --- | --- | --- | --- | --- | --- | --- | --- | --- | --- | --- | --- | --- | --- | --- | --- | --- | --- | --- | --- | --- | --- | --- | --- | --- | --- | --- | | Chimp panTro2 chr8 97120943 97120970 - **AT** | ACACAACCCCAGAGAGAAAGACTTGGAG|  |  |  |  |  |  |  |  |  |  |  |  |  |  |  |  |  |  |  |  |  |  |  |  |  |  |  |  |  |  |  |  |  |  |  |  |  |  |  |  |  |  |  |  |  |  |  |  | | --- | --- | --- | --- | --- | --- | --- | --- | --- | --- | --- | --- | --- | --- | --- | --- | --- | --- | --- | --- | --- | --- | --- | --- | --- | --- | --- | --- | --- | --- | --- | --- | --- | --- | --- | --- | --- | --- | --- | --- | --- | --- | --- | --- | --- | --- | --- | --- | | Gorilla gorGor1 Supercontig\_0003142 14453 14480 + **AT** | ACACAACCCCAGAGAGAAAGGCTTGGAG|  |  |  |  |  |  |  |  |  |  |  |  |  |  |  |  |  |  |  |  |  |  |  |  |  |  |  |  |  |  |  |  |  |  |  |  |  |  |  |  | | --- | --- | --- | --- | --- | --- | --- | --- | --- | --- | --- | --- | --- | --- | --- | --- | --- | --- | --- | --- | --- | --- | --- | --- | --- | --- | --- | --- | --- | --- | --- | --- | --- | --- | --- | --- | --- | --- | --- | --- | | Orangutan ponAbe2 chr8 104573089 104573116 - **AT** | ACACAACCCCAGAGAGAAAGGCTTGGAG|  |  |  |  |  |  |  |  |  |  |  |  |  |  |  |  |  |  |  |  |  |  |  |  |  |  |  |  |  |  |  |  | | --- | --- | --- | --- | --- | --- | --- | --- | --- | --- | --- | --- | --- | --- | --- | --- | --- | --- | --- | --- | --- | --- | --- | --- | --- | --- | --- | --- | --- | --- | --- | --- | | Rhesus rheMac2 chr8 100737464 100737491 - **AT** | ACACAACCCTGGGGAGAAAGGCTTGGAG|  |  |  |  |  |  |  |  |  |  |  |  |  |  |  |  |  |  |  |  |  |  |  |  | | --- | --- | --- | --- | --- | --- | --- | --- | --- | --- | --- | --- | --- | --- | --- | --- | --- | --- | --- | --- | --- | --- | --- | --- | | Baboon papHam1 scaffold7311 93298 93325 - **AT** | ACACAACCCTGGGGAGAAAGGCTTGGAG|  |  |  |  |  |  |  |  |  |  |  |  |  |  |  |  | | --- | --- | --- | --- | --- | --- | --- | --- | --- | --- | --- | --- | --- | --- | --- | --- | | Cow bosTau4 chr14 64224490 64224517 + **AT** | ACAGAACAGCGGAGAGAGAGGTCTGGAG|  |  |  |  |  |  |  |  | | --- | --- | --- | --- | --- | --- | --- | --- | | Dog canFam2 chr13 3429240 3429267 - **AT** | AAAGGACCCCAGGAAGGAAGGTGTGGAG | | | | | | | | | | | | | | | | | | | | | | | | | | | | | | | | | | | | | | | | | | | | | | | | | | | | | | | | | | | | | | | |

**Alignment** (splice site sequences are in lowercase)  

```
Human      agACACAACCCCAGAGAGAAAGGCTTGGAGgt
Chimp      ......................A.......a.
Gorilla    ..............................a.
Orangutan  ..............................a.
Rhesus     ...........TG.G...............a.
Baboon     ...........TG.G...............a.
Cow        .....G...AG.G......G...TC.....a.
Dog        ...A.GG.......GA..G....TG.....a.
```

---

## 21. uc001ljt.2\_45\_52

**Summary**  

|  |  |  |  |  |  |  |  |  |  |  |  |  |  |  |  |  |  |  |  |  |  |  |  |  |  |
| --- | --- | --- | --- | --- | --- | --- | --- | --- | --- | --- | --- | --- | --- | --- | --- | --- | --- | --- | --- | --- | --- | --- | --- | --- | --- |
| No Exon ID Position (hg19) Dir Human donor Chimp donor Category Usage Gene symbol Protein accession mRNA accession Gene title Note|  |  |  |  |  |  |  |  |  |  |  |  |  | | --- | --- | --- | --- | --- | --- | --- | --- | --- | --- | --- | --- | --- | | 21 uc001ljt.2\_45\_52 chr10:129216805 + GT GC (D10) type change (GC to GT) constitutive DOCK1 NP\_001371.1 NM\_001380.3 dedicator of cytokinesis 1 GC to GT | | | | | | | | | | | | | | | | | | | | | | | | | |

**Orthologs**  

|  |  |  |  |  |  |  |  |  |  |  |  |  |  |  |  |  |  |  |  |  |  |  |  |  |  |  |  |  |  |  |  |  |  |  |  |  |  |  |  |  |  |  |  |  |  |  |  |  |  |  |  |  |  |  |  |  |  |  |  |  |  |  |  |  |  |  |  |  |  |  |  |  |  |  |  |  |  |  |  |  |  |  |  |  |  |  |  |  |  |  |  |  |  |  |  |
| --- | --- | --- | --- | --- | --- | --- | --- | --- | --- | --- | --- | --- | --- | --- | --- | --- | --- | --- | --- | --- | --- | --- | --- | --- | --- | --- | --- | --- | --- | --- | --- | --- | --- | --- | --- | --- | --- | --- | --- | --- | --- | --- | --- | --- | --- | --- | --- | --- | --- | --- | --- | --- | --- | --- | --- | --- | --- | --- | --- | --- | --- | --- | --- | --- | --- | --- | --- | --- | --- | --- | --- | --- | --- | --- | --- | --- | --- | --- | --- | --- | --- | --- | --- | --- | --- | --- | --- | --- | --- | --- | --- | --- | --- | --- | --- |
| Species Assembly Chromosome Exon start Exon end Dir Donor Exon sequence|  |  |  |  |  |  |  |  |  |  |  |  |  |  |  |  |  |  |  |  |  |  |  |  |  |  |  |  |  |  |  |  |  |  |  |  |  |  |  |  |  |  |  |  |  |  |  |  |  |  |  |  |  |  |  |  |  |  |  |  |  |  |  |  |  |  |  |  |  |  |  |  |  |  |  |  |  |  |  |  |  |  |  |  |  |  |  |  | | --- | --- | --- | --- | --- | --- | --- | --- | --- | --- | --- | --- | --- | --- | --- | --- | --- | --- | --- | --- | --- | --- | --- | --- | --- | --- | --- | --- | --- | --- | --- | --- | --- | --- | --- | --- | --- | --- | --- | --- | --- | --- | --- | --- | --- | --- | --- | --- | --- | --- | --- | --- | --- | --- | --- | --- | --- | --- | --- | --- | --- | --- | --- | --- | --- | --- | --- | --- | --- | --- | --- | --- | --- | --- | --- | --- | --- | --- | --- | --- | --- | --- | --- | --- | --- | --- | --- | --- | | Human hg19 chr10 129216629 129216805 + **GT** | GTGGAAATCAGCCCCCTGGAGAATGCCATTGAGACCATGCAGCTGACGAACGACAAGATCAACAGCATGGTGCAGCAGCACCTGGATGACCCCAGCCTGCCCATCAACCCGCTCTCCATGCTCCTGAACGGCATCGTGGACCCAGCTGTCATGGGGGGCTTCGCAAACTACGAAAAG|  |  |  |  |  |  |  |  |  |  |  |  |  |  |  |  |  |  |  |  |  |  |  |  |  |  |  |  |  |  |  |  |  |  |  |  |  |  |  |  |  |  |  |  |  |  |  |  |  |  |  |  |  |  |  |  |  |  |  |  |  |  |  |  |  |  |  |  |  |  |  |  |  |  |  |  |  |  |  |  | | --- | --- | --- | --- | --- | --- | --- | --- | --- | --- | --- | --- | --- | --- | --- | --- | --- | --- | --- | --- | --- | --- | --- | --- | --- | --- | --- | --- | --- | --- | --- | --- | --- | --- | --- | --- | --- | --- | --- | --- | --- | --- | --- | --- | --- | --- | --- | --- | --- | --- | --- | --- | --- | --- | --- | --- | --- | --- | --- | --- | --- | --- | --- | --- | --- | --- | --- | --- | --- | --- | --- | --- | --- | --- | --- | --- | --- | --- | --- | --- | | Chimp panTro2 chr10 128497849 128498025 + **GC** | GTGGAAATCAGCCCCCTGGAGAATGCCATCGAGACCATGCAGCTGACGAACGACAAGATCAACAGCATGGTGCAGCAGCACCTGGATGACCCCAGCCTGCCCATCAACCCGCTCTCCATGCTCCTGAACGGCATTGTGGACCCAGCTGTCATGGGGGGCTTCACAAACTACGAAAAG|  |  |  |  |  |  |  |  |  |  |  |  |  |  |  |  |  |  |  |  |  |  |  |  |  |  |  |  |  |  |  |  |  |  |  |  |  |  |  |  |  |  |  |  |  |  |  |  |  |  |  |  |  |  |  |  |  |  |  |  |  |  |  |  |  |  |  |  |  |  |  |  | | --- | --- | --- | --- | --- | --- | --- | --- | --- | --- | --- | --- | --- | --- | --- | --- | --- | --- | --- | --- | --- | --- | --- | --- | --- | --- | --- | --- | --- | --- | --- | --- | --- | --- | --- | --- | --- | --- | --- | --- | --- | --- | --- | --- | --- | --- | --- | --- | --- | --- | --- | --- | --- | --- | --- | --- | --- | --- | --- | --- | --- | --- | --- | --- | --- | --- | --- | --- | --- | --- | --- | --- | | Orangutan ponAbe2 chr10 127063741 127063917 + **GC** | GTGGAAATCAGCCCCCTGGAGAATGCTATCGAGACCATGCAGCTGACGAACGACAAGATCAACAGCATGGTACAGCAGCACCTGGATGACCCCAGCCTGCCCATCAACCCGCTCTCCATGCTCCTGAATGGCATCGTGGACCCAGCTGTCATGGGGGGCTTCACAAACTACGAAAAG|  |  |  |  |  |  |  |  |  |  |  |  |  |  |  |  |  |  |  |  |  |  |  |  |  |  |  |  |  |  |  |  |  |  |  |  |  |  |  |  |  |  |  |  |  |  |  |  |  |  |  |  |  |  |  |  |  |  |  |  |  |  |  |  | | --- | --- | --- | --- | --- | --- | --- | --- | --- | --- | --- | --- | --- | --- | --- | --- | --- | --- | --- | --- | --- | --- | --- | --- | --- | --- | --- | --- | --- | --- | --- | --- | --- | --- | --- | --- | --- | --- | --- | --- | --- | --- | --- | --- | --- | --- | --- | --- | --- | --- | --- | --- | --- | --- | --- | --- | --- | --- | --- | --- | --- | --- | --- | --- | | Rhesus rheMac2 chr9 127050562 127050738 + **GC** | GTGGAAATCAGCCCCCTGGAGAACGCCATCGAGACCATGCAGCTGACGAACGACAAGATCAACAGCATGGTGCAGCAGCACCTGGATGACCCCAGCCTGCCCATCAACCCACTCTCCATGCTCCTGAACGGCATCGTGGACCCAGCTGTCATGGGGGGCTTCACAAACTACGAAAAG|  |  |  |  |  |  |  |  |  |  |  |  |  |  |  |  |  |  |  |  |  |  |  |  |  |  |  |  |  |  |  |  |  |  |  |  |  |  |  |  |  |  |  |  |  |  |  |  |  |  |  |  |  |  |  |  | | --- | --- | --- | --- | --- | --- | --- | --- | --- | --- | --- | --- | --- | --- | --- | --- | --- | --- | --- | --- | --- | --- | --- | --- | --- | --- | --- | --- | --- | --- | --- | --- | --- | --- | --- | --- | --- | --- | --- | --- | --- | --- | --- | --- | --- | --- | --- | --- | --- | --- | --- | --- | --- | --- | --- | --- | | Baboon papHam1 scaffold2063 130725 130901 - **GC** | GTGGAAATCAGCCCCCTGGAGAACGCCATCGAGACCATGCAGCTGACGAACGACAAGATCAACAGCATGGTGCAGCAGCACCTGGATGACCCCAGCCTGCCCATCAACCCGCTCTCCATGCTCCTGAACGGCATCGTGGACCCAGCTGTCATGGGGGGCTTCACAAACTACGAAAAG|  |  |  |  |  |  |  |  |  |  |  |  |  |  |  |  |  |  |  |  |  |  |  |  |  |  |  |  |  |  |  |  |  |  |  |  |  |  |  |  |  |  |  |  |  |  |  |  | | --- | --- | --- | --- | --- | --- | --- | --- | --- | --- | --- | --- | --- | --- | --- | --- | --- | --- | --- | --- | --- | --- | --- | --- | --- | --- | --- | --- | --- | --- | --- | --- | --- | --- | --- | --- | --- | --- | --- | --- | --- | --- | --- | --- | --- | --- | --- | --- | | Marmoset calJac1 Contig3078 214365 214541 + **GC** | GTGGAAATTAGCCCCCTGGAGAACGCCATCGAGACCATGCAGCTGACAAATGACAAGATCAACAGCATGGTGCAGCAGCACCTGGACGACCCCAGCCTGCCCATCAACCCGCTCTCCATGCTCCTGAACGGCATCGTGGACCCAGCTGTCATGGGGGGCTTCACAAACTACGAAAAG|  |  |  |  |  |  |  |  |  |  |  |  |  |  |  |  |  |  |  |  |  |  |  |  |  |  |  |  |  |  |  |  |  |  |  |  |  |  |  |  | | --- | --- | --- | --- | --- | --- | --- | --- | --- | --- | --- | --- | --- | --- | --- | --- | --- | --- | --- | --- | --- | --- | --- | --- | --- | --- | --- | --- | --- | --- | --- | --- | --- | --- | --- | --- | --- | --- | --- | --- | | Lemur micMur1 scaffold\_164 727334 727510 + **GC** | GTGGAGATCAGCCCCCTGGAGAACGCCATCGAGACCATGCAGCTGACAAACGACAAGATCGACAGCATGGTGCAGCAGCACCTGGACGACCCCAGCCTCCCTGTCAACCCCCTCTCCATGCTGCTTAACGGAATCGTGGACCCTGCCGTTATGGGGGGCTTTGCAAACTACGAAAAG|  |  |  |  |  |  |  |  |  |  |  |  |  |  |  |  |  |  |  |  |  |  |  |  |  |  |  |  |  |  |  |  | | --- | --- | --- | --- | --- | --- | --- | --- | --- | --- | --- | --- | --- | --- | --- | --- | --- | --- | --- | --- | --- | --- | --- | --- | --- | --- | --- | --- | --- | --- | --- | --- | | Galago otoGar1 scaffold\_103683.1-619930 121077 121253 + **GC** | GTGGAGGTCAGCCCCCTGGAGAACGCCATAGAGACCATGCAACTGACCAATGACAAGATCGACAGCATGGTGCAGCAGCACCTGGATGACCCCAGCCTGCCCATCAACCCCCTGTCCATGCTCCTCAACGGCATCGTGGACCCCGCTGTCATGGGAGGATTCGCAAACTATGAAAAG|  |  |  |  |  |  |  |  |  |  |  |  |  |  |  |  |  |  |  |  |  |  |  |  | | --- | --- | --- | --- | --- | --- | --- | --- | --- | --- | --- | --- | --- | --- | --- | --- | --- | --- | --- | --- | --- | --- | --- | --- | | Mouse mm9 chr7 142337097 142337273 + **GC** | GTAGAGATCAGCCCACTGGAGAATGCCATCGAGACAATGCAGTTGACCAATGACAAGATCAGCAGCATGGTCCAGCAGCACCTGGATGACCCGGGCCTGCCTATCAACCCCCTGTCCATGCTCCTGAATGGCATTGTGGATCCTGCTGTCATGGGTGGTTTTGCCAATTATGAGAAG|  |  |  |  |  |  |  |  |  |  |  |  |  |  |  |  | | --- | --- | --- | --- | --- | --- | --- | --- | --- | --- | --- | --- | --- | --- | --- | --- | | Cow bosTau4 chr26 47680961 47681137 + **GC** | GTGGAGATCAGCCCCCTGGAGAACGCCATCGAGACCATGCAGCTGACCAACGACAAGATCAACAGCATGGTCCAGCAGCACCTAGACGACCCCAGCCTCCCCATCAACCCGCTCTCCATGCTCCTAAACGGCATCGTGGACCCCGCCGTCATGGGAGGTTTTGCCAATTACGAGAAG|  |  |  |  |  |  |  |  | | --- | --- | --- | --- | --- | --- | --- | --- | | Dog canFam2 chr28 39370692 39370868 + **GT** | GTGGAGATCAGCCCCCTGGAGAATGCCATCGAGACCATGCAGCTGACCAACGACAAGATGAACAGCATGGTGCAGCAGCACCTGGACGACCCCAGCCTCCCCATCAACCCCCTCTCCATGCTCCTCAATGGCATCGTGGACCCCGCCGTCATGGGAGGCTTTGCAAATTATGAAAAG | | | | | | | | | | | | | | | | | | | | | | | | | | | | | | | | | | | | | | | | | | | | | | | | | | | | | | | | | | | | | | | | | | | | | | | | | | | | | | | | | | | | |

**Alignment** (splice site sequences are in lowercase)  

```
Human      agGTGGAAATCAGCCCCCTGGAGAATGCCATTGAGACCATGCAGCTGACGAACGACAAGATCAACAGCATGGTGCAGCAG
Chimp      ...............................C................................................
Orangutan  ............................T..C.........................................A......
Rhesus     .........................C.....C................................................
Baboon     .........................C.....C................................................
Marmoset   ..........T..............C.....C.................A..T...........................
Lemur      .......G.................C.....C.................A............G.................
Galago     .......GG................C.....A...........A.....C..T.........G.................
Mouse      ....A..G........A..............C.....A......T....C..T..........G.........C......
Cow        .......G.................C.....C.................C.......................C......
Dog        .......G.......................C.................C...........G..................

Human      CACCTGGATGACCCCAGCCTGCCCATCAACCCGCTCTCCATGCTCCTGAACGGCATCGTGGACCCAGCTGTCATGGGGGG
Chimp      ........................................................T.......................
Orangutan  ..................................................T.............................
Rhesus     ................................A...............................................
Baboon     ................................................................................
Marmoset   ........C.......................................................................
Lemur      ........C...........C..TG.......C...........G..T.....A...........T..C..T........
Galago     ................................C..G...........C.................C...........A..
Mouse      ..............GG.......T........C..G..............T.....T.....T..T...........T..
Cow        .....A..C...........C..........................A.................C..C........A..
Dog        ........C...........C...........C..............C..T..............C..C........A..

Human      CTTCGCAAACTACGAAAAGgt
Chimp      ....A...............c
Orangutan  ....A...............c
Rhesus     ....A...............c
Baboon     ....A...............c
Marmoset   ....A...............c
Lemur      ...T................c
Galago     A...........T.......c
Mouse      T..T..C..T..T..G....c
Cow        T..T..C..T.....G....c
Dog        ...T.....T..T........
```

---

## 22. uc001lvx.1\_6\_7

**Summary**  

|  |  |  |  |  |  |  |  |  |  |  |  |  |  |  |  |  |  |  |  |  |  |  |  |  |  |
| --- | --- | --- | --- | --- | --- | --- | --- | --- | --- | --- | --- | --- | --- | --- | --- | --- | --- | --- | --- | --- | --- | --- | --- | --- | --- |
| No Exon ID Position (hg19) Dir Human donor Chimp donor Category Usage Gene symbol Protein accession mRNA accession Gene title Note|  |  |  |  |  |  |  |  |  |  |  |  |  | | --- | --- | --- | --- | --- | --- | --- | --- | --- | --- | --- | --- | --- | | 22 uc001lvx.1\_6\_7 chr11:2334938 + GT GC (D4) shift; decrease; frameshift alternative TSPAN32 Q96QS1-3 AF176070.1 tetraspanin-32  | | | | | | | | | | | | | | | | | | | | | | | | | |

**Orthologs**  

|  |  |  |  |  |  |  |  |  |  |  |  |  |  |  |  |  |  |  |  |  |  |  |  |  |  |  |  |  |  |  |  |  |  |  |  |  |  |  |  |  |  |  |  |  |  |  |  |  |  |  |  |  |  |  |  |  |  |  |  |  |  |  |  |  |  |  |  |  |  |  |  |  |  |  |  |  |  |  |  |  |  |  |  |  |  |  |  |
| --- | --- | --- | --- | --- | --- | --- | --- | --- | --- | --- | --- | --- | --- | --- | --- | --- | --- | --- | --- | --- | --- | --- | --- | --- | --- | --- | --- | --- | --- | --- | --- | --- | --- | --- | --- | --- | --- | --- | --- | --- | --- | --- | --- | --- | --- | --- | --- | --- | --- | --- | --- | --- | --- | --- | --- | --- | --- | --- | --- | --- | --- | --- | --- | --- | --- | --- | --- | --- | --- | --- | --- | --- | --- | --- | --- | --- | --- | --- | --- | --- | --- | --- | --- | --- | --- | --- | --- |
| Species Assembly Chromosome Exon start Exon end Dir Donor Exon sequence|  |  |  |  |  |  |  |  |  |  |  |  |  |  |  |  |  |  |  |  |  |  |  |  |  |  |  |  |  |  |  |  |  |  |  |  |  |  |  |  |  |  |  |  |  |  |  |  |  |  |  |  |  |  |  |  |  |  |  |  |  |  |  |  |  |  |  |  |  |  |  |  |  |  |  |  |  |  |  |  | | --- | --- | --- | --- | --- | --- | --- | --- | --- | --- | --- | --- | --- | --- | --- | --- | --- | --- | --- | --- | --- | --- | --- | --- | --- | --- | --- | --- | --- | --- | --- | --- | --- | --- | --- | --- | --- | --- | --- | --- | --- | --- | --- | --- | --- | --- | --- | --- | --- | --- | --- | --- | --- | --- | --- | --- | --- | --- | --- | --- | --- | --- | --- | --- | --- | --- | --- | --- | --- | --- | --- | --- | --- | --- | --- | --- | --- | --- | --- | --- | | Human hg19 chr11 2334884 2334938 + **GT** | GTGGAGGACGCCATGCTGGACACCTACGACCTGGTATATGAGCAGGCGATGAAAG|  |  |  |  |  |  |  |  |  |  |  |  |  |  |  |  |  |  |  |  |  |  |  |  |  |  |  |  |  |  |  |  |  |  |  |  |  |  |  |  |  |  |  |  |  |  |  |  |  |  |  |  |  |  |  |  |  |  |  |  |  |  |  |  |  |  |  |  |  |  |  |  | | --- | --- | --- | --- | --- | --- | --- | --- | --- | --- | --- | --- | --- | --- | --- | --- | --- | --- | --- | --- | --- | --- | --- | --- | --- | --- | --- | --- | --- | --- | --- | --- | --- | --- | --- | --- | --- | --- | --- | --- | --- | --- | --- | --- | --- | --- | --- | --- | --- | --- | --- | --- | --- | --- | --- | --- | --- | --- | --- | --- | --- | --- | --- | --- | --- | --- | --- | --- | --- | --- | --- | --- | | Chimp panTro2 chr11 2368438 2368492 + **GC** | GTGGAGGACGCCATGCTGGACACCTACGACCTGGTATATGAGCAGGCGATGAAAG|  |  |  |  |  |  |  |  |  |  |  |  |  |  |  |  |  |  |  |  |  |  |  |  |  |  |  |  |  |  |  |  |  |  |  |  |  |  |  |  |  |  |  |  |  |  |  |  |  |  |  |  |  |  |  |  |  |  |  |  |  |  |  |  | | --- | --- | --- | --- | --- | --- | --- | --- | --- | --- | --- | --- | --- | --- | --- | --- | --- | --- | --- | --- | --- | --- | --- | --- | --- | --- | --- | --- | --- | --- | --- | --- | --- | --- | --- | --- | --- | --- | --- | --- | --- | --- | --- | --- | --- | --- | --- | --- | --- | --- | --- | --- | --- | --- | --- | --- | --- | --- | --- | --- | --- | --- | --- | --- | | Orangutan ponAbe2 chr11 3598089 3598143 + **GC** | GTGGAGGATGCCATGCTGGACACCTACGACCTGGTATATGAGCAGGCGATGAAAG|  |  |  |  |  |  |  |  |  |  |  |  |  |  |  |  |  |  |  |  |  |  |  |  |  |  |  |  |  |  |  |  |  |  |  |  |  |  |  |  |  |  |  |  |  |  |  |  |  |  |  |  |  |  |  |  | | --- | --- | --- | --- | --- | --- | --- | --- | --- | --- | --- | --- | --- | --- | --- | --- | --- | --- | --- | --- | --- | --- | --- | --- | --- | --- | --- | --- | --- | --- | --- | --- | --- | --- | --- | --- | --- | --- | --- | --- | --- | --- | --- | --- | --- | --- | --- | --- | --- | --- | --- | --- | --- | --- | --- | --- | | Rhesus rheMac2 chr14 2321288 2321342 + **GC** | GTGGAGGATGCCATGCTGGACACCTACGACCTGGTATATGAGCAGGCGATGAAAG|  |  |  |  |  |  |  |  |  |  |  |  |  |  |  |  |  |  |  |  |  |  |  |  |  |  |  |  |  |  |  |  |  |  |  |  |  |  |  |  |  |  |  |  |  |  |  |  | | --- | --- | --- | --- | --- | --- | --- | --- | --- | --- | --- | --- | --- | --- | --- | --- | --- | --- | --- | --- | --- | --- | --- | --- | --- | --- | --- | --- | --- | --- | --- | --- | --- | --- | --- | --- | --- | --- | --- | --- | --- | --- | --- | --- | --- | --- | --- | --- | | Baboon papHam1 scaffold5713 527 581 - **GC** | GTGGAGGATGCCATGCTGGACACCTACGACCTGGTATATGAGCAGGCAATGAAAG|  |  |  |  |  |  |  |  |  |  |  |  |  |  |  |  |  |  |  |  |  |  |  |  |  |  |  |  |  |  |  |  |  |  |  |  |  |  |  |  | | --- | --- | --- | --- | --- | --- | --- | --- | --- | --- | --- | --- | --- | --- | --- | --- | --- | --- | --- | --- | --- | --- | --- | --- | --- | --- | --- | --- | --- | --- | --- | --- | --- | --- | --- | --- | --- | --- | --- | --- | | Marmoset calJac1 Contig11729 63051 63105 - **GC** | GTTGAGGATGCCATGCTGGACACCTACGACCTGGTGTATGAGCAGGCAGTGAAAG|  |  |  |  |  |  |  |  |  |  |  |  |  |  |  |  |  |  |  |  |  |  |  |  |  |  |  |  |  |  |  |  | | --- | --- | --- | --- | --- | --- | --- | --- | --- | --- | --- | --- | --- | --- | --- | --- | --- | --- | --- | --- | --- | --- | --- | --- | --- | --- | --- | --- | --- | --- | --- | --- | | Galago otoGar1 scaffold\_94756.1-68377 8045 8099 + **CT** | GTGGAGGATGCTGCTTTGGATACCTACGACCTGGTATATGAACAAGCGGTGAAGA|  |  |  |  |  |  |  |  |  |  |  |  |  |  |  |  |  |  |  |  |  |  |  |  | | --- | --- | --- | --- | --- | --- | --- | --- | --- | --- | --- | --- | --- | --- | --- | --- | --- | --- | --- | --- | --- | --- | --- | --- | | Mouse mm9 chr7 150200864 150200918 + **GC** | GTGGAGGATGCGGTGTTGGACACCTATGACTTCGTGTATGATCAGGCAATGAAGA|  |  |  |  |  |  |  |  |  |  |  |  |  |  |  |  | | --- | --- | --- | --- | --- | --- | --- | --- | --- | --- | --- | --- | --- | --- | --- | --- | | Cow bosTau4 chr29 51123701 51123755 - **GC** | ATGGAGGACGCCGTGCTGGATGCCTATGACCGGGCCTATGAGCGGGCGCTGAGGA|  |  |  |  |  |  |  |  | | --- | --- | --- | --- | --- | --- | --- | --- | | Dog canFam2 chr18 49457421 49457475 + **AC** | GTGGAGGATGCTGTTTTGGACACTTACGACCTGGTGTATGACCAGGCGGTGAAGA | | | | | | | | | | | | | | | | | | | | | | | | | | | | | | | | | | | | | | | | | | | | | | | | | | | | | | | | | | | | | | | | | | | | | | | | | | | | | |

**Alignment** (splice site sequences are in lowercase)  

```
Human      agGTGGAGGACGCCATGCTGGACACCTACGACCTGGTATATGAGCAGGCGATGAAAGgt
Chimp      ..........................................................c
Orangutan  ..........T...............................................c
Rhesus     ..........T...............................................c
Baboon     ..........T......................................A........c
Marmoset   ....T.....T..........................G...........AG.......c
Galago     ..........T..TGCTT....T....................A..A...G....GAc.
Mouse      ..........T..GG..T..........T...T.C..G.....T.....A.....GA.c
Cow        ..A...........G.......TG....T....G..CC.......G....C...GGA.c
Dog        ..........T..TG.TT.......T...........G.....C......G....GAac
```

---

## 23. uc009ywj.2\_1\_19

**Summary**  

|  |  |  |  |  |  |  |  |  |  |  |  |  |  |  |  |  |  |  |  |  |  |  |  |  |  |
| --- | --- | --- | --- | --- | --- | --- | --- | --- | --- | --- | --- | --- | --- | --- | --- | --- | --- | --- | --- | --- | --- | --- | --- | --- | --- |
| No Exon ID Position (hg19) Dir Human donor Chimp donor Category Usage Gene symbol Protein accession mRNA accession Gene title Note|  |  |  |  |  |  |  |  |  |  |  |  |  | | --- | --- | --- | --- | --- | --- | --- | --- | --- | --- | --- | --- | --- | | 23 uc009ywj.2\_1\_19 chr11:94225808 - GT AT (D1) shift; increase; inframe alternative MRE11A B3KTC7 AK095388.1 meiotic recombination 11 homolog A (S. cerevisiae) dbSNP:rs496797 | | | | | | | | | | | | | | | | | | | | | | | | | |

**Orthologs**  

|  |  |  |  |  |  |  |  |  |  |  |  |  |  |  |  |  |  |  |  |  |  |  |  |  |  |  |  |  |  |  |  |  |  |  |  |  |  |  |  |  |  |  |  |  |  |  |  |  |  |  |  |  |  |  |  |  |  |  |  |  |  |  |  |  |  |  |  |  |  |  |  |  |  |  |  |  |  |  |  |
| --- | --- | --- | --- | --- | --- | --- | --- | --- | --- | --- | --- | --- | --- | --- | --- | --- | --- | --- | --- | --- | --- | --- | --- | --- | --- | --- | --- | --- | --- | --- | --- | --- | --- | --- | --- | --- | --- | --- | --- | --- | --- | --- | --- | --- | --- | --- | --- | --- | --- | --- | --- | --- | --- | --- | --- | --- | --- | --- | --- | --- | --- | --- | --- | --- | --- | --- | --- | --- | --- | --- | --- | --- | --- | --- | --- | --- | --- | --- | --- |
| Species Assembly Chromosome Exon start Exon end Dir Donor Exon sequence|  |  |  |  |  |  |  |  |  |  |  |  |  |  |  |  |  |  |  |  |  |  |  |  |  |  |  |  |  |  |  |  |  |  |  |  |  |  |  |  |  |  |  |  |  |  |  |  |  |  |  |  |  |  |  |  |  |  |  |  |  |  |  |  |  |  |  |  |  |  |  |  | | --- | --- | --- | --- | --- | --- | --- | --- | --- | --- | --- | --- | --- | --- | --- | --- | --- | --- | --- | --- | --- | --- | --- | --- | --- | --- | --- | --- | --- | --- | --- | --- | --- | --- | --- | --- | --- | --- | --- | --- | --- | --- | --- | --- | --- | --- | --- | --- | --- | --- | --- | --- | --- | --- | --- | --- | --- | --- | --- | --- | --- | --- | --- | --- | --- | --- | --- | --- | --- | --- | --- | --- | | Human hg19 chr11 94225808 94225836 - **GT** | ATGAACAGAAATATTAGTCATCAAAAAGG|  |  |  |  |  |  |  |  |  |  |  |  |  |  |  |  |  |  |  |  |  |  |  |  |  |  |  |  |  |  |  |  |  |  |  |  |  |  |  |  |  |  |  |  |  |  |  |  |  |  |  |  |  |  |  |  |  |  |  |  |  |  |  |  | | --- | --- | --- | --- | --- | --- | --- | --- | --- | --- | --- | --- | --- | --- | --- | --- | --- | --- | --- | --- | --- | --- | --- | --- | --- | --- | --- | --- | --- | --- | --- | --- | --- | --- | --- | --- | --- | --- | --- | --- | --- | --- | --- | --- | --- | --- | --- | --- | --- | --- | --- | --- | --- | --- | --- | --- | --- | --- | --- | --- | --- | --- | --- | --- | | Chimp panTro2 chr11 93012501 93012529 - **AT** | ATCAACAGAAATATTAGTCATCAAAAAGG|  |  |  |  |  |  |  |  |  |  |  |  |  |  |  |  |  |  |  |  |  |  |  |  |  |  |  |  |  |  |  |  |  |  |  |  |  |  |  |  |  |  |  |  |  |  |  |  |  |  |  |  |  |  |  |  | | --- | --- | --- | --- | --- | --- | --- | --- | --- | --- | --- | --- | --- | --- | --- | --- | --- | --- | --- | --- | --- | --- | --- | --- | --- | --- | --- | --- | --- | --- | --- | --- | --- | --- | --- | --- | --- | --- | --- | --- | --- | --- | --- | --- | --- | --- | --- | --- | --- | --- | --- | --- | --- | --- | --- | --- | | Gorilla gorGor1 Supercontig\_0009178 19023 19051 + **AT** | ATCAACAGAAATATTAGTCATCAAAAAGG|  |  |  |  |  |  |  |  |  |  |  |  |  |  |  |  |  |  |  |  |  |  |  |  |  |  |  |  |  |  |  |  |  |  |  |  |  |  |  |  |  |  |  |  |  |  |  |  | | --- | --- | --- | --- | --- | --- | --- | --- | --- | --- | --- | --- | --- | --- | --- | --- | --- | --- | --- | --- | --- | --- | --- | --- | --- | --- | --- | --- | --- | --- | --- | --- | --- | --- | --- | --- | --- | --- | --- | --- | --- | --- | --- | --- | --- | --- | --- | --- | | Orangutan ponAbe2 chr11 90464221 90464249 - **AT** | ATGAACAGAAATATTAGTCATCAAAAAGG|  |  |  |  |  |  |  |  |  |  |  |  |  |  |  |  |  |  |  |  |  |  |  |  |  |  |  |  |  |  |  |  |  |  |  |  |  |  |  |  | | --- | --- | --- | --- | --- | --- | --- | --- | --- | --- | --- | --- | --- | --- | --- | --- | --- | --- | --- | --- | --- | --- | --- | --- | --- | --- | --- | --- | --- | --- | --- | --- | --- | --- | --- | --- | --- | --- | --- | --- | | Rhesus rheMac2 chr14 93041985 93042013 - **AT** | ATGAACAGAAATACTAGTCATCAAAAAGG|  |  |  |  |  |  |  |  |  |  |  |  |  |  |  |  |  |  |  |  |  |  |  |  |  |  |  |  |  |  |  |  | | --- | --- | --- | --- | --- | --- | --- | --- | --- | --- | --- | --- | --- | --- | --- | --- | --- | --- | --- | --- | --- | --- | --- | --- | --- | --- | --- | --- | --- | --- | --- | --- | | Baboon papHam1 scaffold11054 29745 29773 - **AT** | ATGAACAGAAATACTAGTCATCAAAAAGG|  |  |  |  |  |  |  |  |  |  |  |  |  |  |  |  |  |  |  |  |  |  |  |  | | --- | --- | --- | --- | --- | --- | --- | --- | --- | --- | --- | --- | --- | --- | --- | --- | --- | --- | --- | --- | --- | --- | --- | --- | | Marmoset calJac1 Contig3955 19968 19996 + **AT** | GTGAACAGAAATATTAGTCATCAAAAAGG|  |  |  |  |  |  |  |  |  |  |  |  |  |  |  |  | | --- | --- | --- | --- | --- | --- | --- | --- | --- | --- | --- | --- | --- | --- | --- | --- | | Lemur micMur1 scaffold\_11751 28191 28219 - **AT** | ATGAACAGGAATGTGAGTCATCAAAAAGG|  |  |  |  |  |  |  |  | | --- | --- | --- | --- | --- | --- | --- | --- | | Dog canFam2 chr21 9450035 9450063 + **AT** | ATGAACAGAAACGTTCGTCATCAAAAAGA | | | | | | | | | | | | | | | | | | | | | | | | | | | | | | | | | | | | | | | | | | | | | | | | | | | | | | | | | | | | | | | | | | | | | | |

**Alignment** (splice site sequences are in lowercase)  

```
Human      ATGAACAGAAATATTAGTCATCAAAAAGGgt
Chimp      ..C..........................a.
Gorilla    ..C..........................a.
Orangutan  .............................a.
Rhesus     .............C...............a.
Baboon     .............C...............a.
Marmoset   G............................a.
Lemur      ........G...G.G..............a.
Dog        ...........CG..C............Aa.
```

---

## 24. uc001qip.3\_20\_26

**Summary**  

|  |  |  |  |  |  |  |  |  |  |  |  |  |  |  |  |  |  |  |  |  |  |  |  |  |  |
| --- | --- | --- | --- | --- | --- | --- | --- | --- | --- | --- | --- | --- | --- | --- | --- | --- | --- | --- | --- | --- | --- | --- | --- | --- | --- |
| No Exon ID Position (hg19) Dir Human donor Chimp donor Category Usage Gene symbol Protein accession mRNA accession Gene title Note|  |  |  |  |  |  |  |  |  |  |  |  |  | | --- | --- | --- | --- | --- | --- | --- | --- | --- | --- | --- | --- | --- | | 24 uc001qip.3\_20\_26 chr12:999676 + GT AT (D3) shift; decrease; inframe alternative WNK1 NP\_055638.2 NM\_014823.2 serine/threonine-protein kinase WNK1 GTNGTN | | | | | | | | | | | | | | | | | | | | | | | | | |

**Orthologs**  

|  |  |  |  |  |  |  |  |  |  |  |  |  |  |  |  |  |  |  |  |  |  |  |  |  |  |  |  |  |  |  |  |  |  |  |  |  |  |  |  |  |  |  |  |  |  |  |  |  |  |  |  |  |  |  |  |  |  |  |  |  |  |  |  |  |  |  |  |  |  |  |  |  |  |  |  |  |  |  |  |  |  |  |  |  |  |  |  |  |  |  |  |  |  |  |  |  |  |  |  |  |  |  |  |
| --- | --- | --- | --- | --- | --- | --- | --- | --- | --- | --- | --- | --- | --- | --- | --- | --- | --- | --- | --- | --- | --- | --- | --- | --- | --- | --- | --- | --- | --- | --- | --- | --- | --- | --- | --- | --- | --- | --- | --- | --- | --- | --- | --- | --- | --- | --- | --- | --- | --- | --- | --- | --- | --- | --- | --- | --- | --- | --- | --- | --- | --- | --- | --- | --- | --- | --- | --- | --- | --- | --- | --- | --- | --- | --- | --- | --- | --- | --- | --- | --- | --- | --- | --- | --- | --- | --- | --- | --- | --- | --- | --- | --- | --- | --- | --- | --- | --- | --- | --- | --- | --- | --- | --- |
| Species Assembly Chromosome Exon start Exon end Dir Donor Exon sequence|  |  |  |  |  |  |  |  |  |  |  |  |  |  |  |  |  |  |  |  |  |  |  |  |  |  |  |  |  |  |  |  |  |  |  |  |  |  |  |  |  |  |  |  |  |  |  |  |  |  |  |  |  |  |  |  |  |  |  |  |  |  |  |  |  |  |  |  |  |  |  |  |  |  |  |  |  |  |  |  |  |  |  |  |  |  |  |  |  |  |  |  |  |  |  |  | | --- | --- | --- | --- | --- | --- | --- | --- | --- | --- | --- | --- | --- | --- | --- | --- | --- | --- | --- | --- | --- | --- | --- | --- | --- | --- | --- | --- | --- | --- | --- | --- | --- | --- | --- | --- | --- | --- | --- | --- | --- | --- | --- | --- | --- | --- | --- | --- | --- | --- | --- | --- | --- | --- | --- | --- | --- | --- | --- | --- | --- | --- | --- | --- | --- | --- | --- | --- | --- | --- | --- | --- | --- | --- | --- | --- | --- | --- | --- | --- | --- | --- | --- | --- | --- | --- | --- | --- | --- | --- | --- | --- | --- | --- | --- | --- | | Human hg19 chr12 999619 999676 + **GT** | CCTGTGTCCATGGCGGCTCCAACAGCAATCACAGAAGCAGGAACACAGCCTCAGAAGG|  |  |  |  |  |  |  |  |  |  |  |  |  |  |  |  |  |  |  |  |  |  |  |  |  |  |  |  |  |  |  |  |  |  |  |  |  |  |  |  |  |  |  |  |  |  |  |  |  |  |  |  |  |  |  |  |  |  |  |  |  |  |  |  |  |  |  |  |  |  |  |  |  |  |  |  |  |  |  |  |  |  |  |  |  |  |  |  | | --- | --- | --- | --- | --- | --- | --- | --- | --- | --- | --- | --- | --- | --- | --- | --- | --- | --- | --- | --- | --- | --- | --- | --- | --- | --- | --- | --- | --- | --- | --- | --- | --- | --- | --- | --- | --- | --- | --- | --- | --- | --- | --- | --- | --- | --- | --- | --- | --- | --- | --- | --- | --- | --- | --- | --- | --- | --- | --- | --- | --- | --- | --- | --- | --- | --- | --- | --- | --- | --- | --- | --- | --- | --- | --- | --- | --- | --- | --- | --- | --- | --- | --- | --- | --- | --- | --- | --- | | Chimp panTro2 chr12 913688 913745 + **AT** | CCTGTGTCCGTGGTGGCTCCAGCAGCAATTACAGAAGCAGGAGCACAGCCTCAGAAGG|  |  |  |  |  |  |  |  |  |  |  |  |  |  |  |  |  |  |  |  |  |  |  |  |  |  |  |  |  |  |  |  |  |  |  |  |  |  |  |  |  |  |  |  |  |  |  |  |  |  |  |  |  |  |  |  |  |  |  |  |  |  |  |  |  |  |  |  |  |  |  |  |  |  |  |  |  |  |  |  | | --- | --- | --- | --- | --- | --- | --- | --- | --- | --- | --- | --- | --- | --- | --- | --- | --- | --- | --- | --- | --- | --- | --- | --- | --- | --- | --- | --- | --- | --- | --- | --- | --- | --- | --- | --- | --- | --- | --- | --- | --- | --- | --- | --- | --- | --- | --- | --- | --- | --- | --- | --- | --- | --- | --- | --- | --- | --- | --- | --- | --- | --- | --- | --- | --- | --- | --- | --- | --- | --- | --- | --- | --- | --- | --- | --- | --- | --- | --- | --- | | Orangutan ponAbe2 chr12 899525 899582 + **AT** | CCTGTGCCCGTGGTGGTTCCAACAGCAATTACAGAAGCAGGAGCACAGCCTCAGAAGG|  |  |  |  |  |  |  |  |  |  |  |  |  |  |  |  |  |  |  |  |  |  |  |  |  |  |  |  |  |  |  |  |  |  |  |  |  |  |  |  |  |  |  |  |  |  |  |  |  |  |  |  |  |  |  |  |  |  |  |  |  |  |  |  |  |  |  |  |  |  |  |  | | --- | --- | --- | --- | --- | --- | --- | --- | --- | --- | --- | --- | --- | --- | --- | --- | --- | --- | --- | --- | --- | --- | --- | --- | --- | --- | --- | --- | --- | --- | --- | --- | --- | --- | --- | --- | --- | --- | --- | --- | --- | --- | --- | --- | --- | --- | --- | --- | --- | --- | --- | --- | --- | --- | --- | --- | --- | --- | --- | --- | --- | --- | --- | --- | --- | --- | --- | --- | --- | --- | --- | --- | | Rhesus rheMac2 chr11 881860 881917 + **AT** | CCTGTGTCCGTGGTGGCTCCAGCAGCAGTTACAGAAGCAGGAGCACAGCCTCAGAAGG|  |  |  |  |  |  |  |  |  |  |  |  |  |  |  |  |  |  |  |  |  |  |  |  |  |  |  |  |  |  |  |  |  |  |  |  |  |  |  |  |  |  |  |  |  |  |  |  |  |  |  |  |  |  |  |  |  |  |  |  |  |  |  |  | | --- | --- | --- | --- | --- | --- | --- | --- | --- | --- | --- | --- | --- | --- | --- | --- | --- | --- | --- | --- | --- | --- | --- | --- | --- | --- | --- | --- | --- | --- | --- | --- | --- | --- | --- | --- | --- | --- | --- | --- | --- | --- | --- | --- | --- | --- | --- | --- | --- | --- | --- | --- | --- | --- | --- | --- | --- | --- | --- | --- | --- | --- | --- | --- | | Baboon papHam1 scaffold108 105965 106022 - **AT** | CCTGTGTCCGTGGTGGCTCCAGCAGCAGTTACAGAAGCAGGAGCACAGCCTCAGAAGG|  |  |  |  |  |  |  |  |  |  |  |  |  |  |  |  |  |  |  |  |  |  |  |  |  |  |  |  |  |  |  |  |  |  |  |  |  |  |  |  |  |  |  |  |  |  |  |  |  |  |  |  |  |  |  |  | | --- | --- | --- | --- | --- | --- | --- | --- | --- | --- | --- | --- | --- | --- | --- | --- | --- | --- | --- | --- | --- | --- | --- | --- | --- | --- | --- | --- | --- | --- | --- | --- | --- | --- | --- | --- | --- | --- | --- | --- | --- | --- | --- | --- | --- | --- | --- | --- | --- | --- | --- | --- | --- | --- | --- | --- | | Marmoset calJac1 Contig707 362457 362514 - **AT** | CCTGTGTCCGTGGTGGCTCCAACAGCAGTTACAGAAGCAGGAGCACAGCCTCAGAAGG|  |  |  |  |  |  |  |  |  |  |  |  |  |  |  |  |  |  |  |  |  |  |  |  |  |  |  |  |  |  |  |  |  |  |  |  |  |  |  |  |  |  |  |  |  |  |  |  | | --- | --- | --- | --- | --- | --- | --- | --- | --- | --- | --- | --- | --- | --- | --- | --- | --- | --- | --- | --- | --- | --- | --- | --- | --- | --- | --- | --- | --- | --- | --- | --- | --- | --- | --- | --- | --- | --- | --- | --- | --- | --- | --- | --- | --- | --- | --- | --- | | Tarsier tarSyr1 scaffold\_6429 43022 43079 + **AT** | CCTGTGTCCGTGGTGGCTCCAACAGCGGGTACGGAAGCAGGAGCACCGCTTCAAAAGG|  |  |  |  |  |  |  |  |  |  |  |  |  |  |  |  |  |  |  |  |  |  |  |  |  |  |  |  |  |  |  |  |  |  |  |  |  |  |  |  | | --- | --- | --- | --- | --- | --- | --- | --- | --- | --- | --- | --- | --- | --- | --- | --- | --- | --- | --- | --- | --- | --- | --- | --- | --- | --- | --- | --- | --- | --- | --- | --- | --- | --- | --- | --- | --- | --- | --- | --- | | Lemur micMur1 scaffold\_6516 28599 28656 + **AT** | CTGGTGTCCATGGTGGCTCCAACAGCAGTTACAGAAGCAGGAGCACAGCCTGAGAAGG|  |  |  |  |  |  |  |  |  |  |  |  |  |  |  |  |  |  |  |  |  |  |  |  |  |  |  |  |  |  |  |  | | --- | --- | --- | --- | --- | --- | --- | --- | --- | --- | --- | --- | --- | --- | --- | --- | --- | --- | --- | --- | --- | --- | --- | --- | --- | --- | --- | --- | --- | --- | --- | --- | | Galago otoGar1 scaffold\_103064.1-155269 16633 16690 + **AT** | CCTGTGTCCATGGTGGCTCCAATAGCTGTTACAGAATCAGGAGCACAGCCTCAGAAGG|  |  |  |  |  |  |  |  |  |  |  |  |  |  |  |  |  |  |  |  |  |  |  |  | | --- | --- | --- | --- | --- | --- | --- | --- | --- | --- | --- | --- | --- | --- | --- | --- | --- | --- | --- | --- | --- | --- | --- | --- | | Mouse mm9 chr6 119893827 119893884 - **AT** | CCTTTGTCTACGATGTCTTCAACAACAGTTACAGAAGCAGGGACTCGGCTTCAAAAGG|  |  |  |  |  |  |  |  |  |  |  |  |  |  |  |  | | --- | --- | --- | --- | --- | --- | --- | --- | --- | --- | --- | --- | --- | --- | --- | --- | | Cow bosTau4 chr5 114602325 114602382 + **AT** | CCCGTGTCTGTGGTGGCTCCAACAGCAGTTGTGGAGACCGAAGCCCAACTTCCAAAGG|  |  |  |  |  |  |  |  | | --- | --- | --- | --- | --- | --- | --- | --- | | Dog canFam2 chr27 45929131 45929188 + **AT** | CCTGTGTCCATGGTGGCTCCAACAGCAGTCACAGAAGCAGGAGCACAGCCTCAGAAGG | | | | | | | | | | | | | | | | | | | | | | | | | | | | | | | | | | | | | | | | | | | | | | | | | | | | | | | | | | | | | | | | | | | | | | | | | | | | | | | | | | | | | | | | | | | |

**Alignment** (splice site sequences are in lowercase)  

```
Human      agCCTGTGTCCATGGCGGCTCCAACAGCAATCACAGAAGCAGGAACACAGCCTCAGAAGGgt
Chimp      ...........G...T.......G.......T............G...............a.
Orangutan  ........C..G...T..T............T............G...............a.
Rhesus     ...........G...T.......G.....G.T............G...............a.
Baboon     ...........G...T.......G.....G.T............G...............a.
Marmoset   ...........G...T.............G.T............G...............a.
Tarsier    ...........G...T............GGGT..G.........G...C..T...A....a.
Lemur      ...TG..........T.............G.T............G........G......a.
Galago     ...............T........T...TG.T......T.....G...............a.
Mouse      .....T....T.C.AT.T..T.....A..G.T...........G..T.G..T...A....a.
Cow        ....C.....TG...T.............G.TGTG..GA.C.A.G.C..A.T..CA....a.
Dog        ...............T.............G..............G...............a.
```

---

## 25. uc001szm.1\_1\_16

**Summary**  

|  |  |  |  |  |  |  |  |  |  |  |  |  |  |  |  |  |  |  |  |  |  |  |  |  |  |
| --- | --- | --- | --- | --- | --- | --- | --- | --- | --- | --- | --- | --- | --- | --- | --- | --- | --- | --- | --- | --- | --- | --- | --- | --- | --- |
| No Exon ID Position (hg19) Dir Human donor Chimp donor Category Usage Gene symbol Protein accession mRNA accession Gene title Note|  |  |  |  |  |  |  |  |  |  |  |  |  | | --- | --- | --- | --- | --- | --- | --- | --- | --- | --- | --- | --- | --- | | 25 uc001szm.1\_1\_16 chr12:81472207 + GT TT (D3) shift; decrease; inframe alternative ACSS3 Q9H6R3 DA156034.1 acyl-CoA synthetase short-chain family member 3 GTNGTN | | | | | | | | | | | | | | | | | | | | | | | | | |

**Orthologs**  

|  |  |  |  |  |  |  |  |  |  |  |  |  |  |  |  |  |  |  |  |  |  |  |  |  |  |  |  |  |  |  |  |  |  |  |  |  |  |  |  |  |  |  |  |  |  |  |  |  |  |  |  |  |  |  |  |  |  |  |  |  |  |  |  |  |  |  |  |  |  |  |  |  |  |  |  |  |  |  |  |  |  |  |  |  |  |  |  |  |  |  |  |  |  |  |  |
| --- | --- | --- | --- | --- | --- | --- | --- | --- | --- | --- | --- | --- | --- | --- | --- | --- | --- | --- | --- | --- | --- | --- | --- | --- | --- | --- | --- | --- | --- | --- | --- | --- | --- | --- | --- | --- | --- | --- | --- | --- | --- | --- | --- | --- | --- | --- | --- | --- | --- | --- | --- | --- | --- | --- | --- | --- | --- | --- | --- | --- | --- | --- | --- | --- | --- | --- | --- | --- | --- | --- | --- | --- | --- | --- | --- | --- | --- | --- | --- | --- | --- | --- | --- | --- | --- | --- | --- | --- | --- | --- | --- | --- | --- | --- | --- |
| Species Assembly Chromosome Exon start Exon end Dir Donor Exon sequence|  |  |  |  |  |  |  |  |  |  |  |  |  |  |  |  |  |  |  |  |  |  |  |  |  |  |  |  |  |  |  |  |  |  |  |  |  |  |  |  |  |  |  |  |  |  |  |  |  |  |  |  |  |  |  |  |  |  |  |  |  |  |  |  |  |  |  |  |  |  |  |  |  |  |  |  |  |  |  |  |  |  |  |  |  |  |  |  | | --- | --- | --- | --- | --- | --- | --- | --- | --- | --- | --- | --- | --- | --- | --- | --- | --- | --- | --- | --- | --- | --- | --- | --- | --- | --- | --- | --- | --- | --- | --- | --- | --- | --- | --- | --- | --- | --- | --- | --- | --- | --- | --- | --- | --- | --- | --- | --- | --- | --- | --- | --- | --- | --- | --- | --- | --- | --- | --- | --- | --- | --- | --- | --- | --- | --- | --- | --- | --- | --- | --- | --- | --- | --- | --- | --- | --- | --- | --- | --- | --- | --- | --- | --- | --- | --- | --- | --- | | Human hg19 chr12 81471900 81472207 + **GT** | ATGAAACCGTCTTGGCTGCAGTGTCGTAAAGTCACCAGCGCCGGGGGGCTCGGAGGGCCCTTGCCTGGGTCCTCTCCGGCCCGGGGAGCCGGTGCGGCCCTCAGGGCTTTAGTGGTCCCGGGCCCGCGGGGCGGTCTCGGGGGCCGGGGATGCAGGGCACTGTCCTCCGGCAGTGGCAGCGAGTACAAGACCCACTTCGCAGCCTCGGTGACCGACCCCGAGAGGTTCTGGGGCAAAGCTGCCGAGCAGATCAGCTGGTACAAGCCCTGGACCAAAACGCTGGAGAACAAACACTCGCCCTCTACCAG|  |  |  |  |  |  |  |  |  |  |  |  |  |  |  |  |  |  |  |  |  |  |  |  |  |  |  |  |  |  |  |  |  |  |  |  |  |  |  |  |  |  |  |  |  |  |  |  |  |  |  |  |  |  |  |  |  |  |  |  |  |  |  |  |  |  |  |  |  |  |  |  |  |  |  |  |  |  |  |  | | --- | --- | --- | --- | --- | --- | --- | --- | --- | --- | --- | --- | --- | --- | --- | --- | --- | --- | --- | --- | --- | --- | --- | --- | --- | --- | --- | --- | --- | --- | --- | --- | --- | --- | --- | --- | --- | --- | --- | --- | --- | --- | --- | --- | --- | --- | --- | --- | --- | --- | --- | --- | --- | --- | --- | --- | --- | --- | --- | --- | --- | --- | --- | --- | --- | --- | --- | --- | --- | --- | --- | --- | --- | --- | --- | --- | --- | --- | --- | --- | | Chimp panTro2 chr12 81728625 81728932 + **TT** | ATGAAACCGTCTTGGCTGCAATGTCGTAAAGTCACCAGCGCCGGGGGGCTCGGAGGACCCTTGCCTGGGTCCTCTCCGGCCCGGGGAGCCGGTGCGGCCCTCAGGGCTTTAGTGGTCCCGGGCCCGCGGGGCGGTCTCGGGGGCCGGGGATGCAGGGCACTGTCCTCCGGCAGTGGCAGCGAGTACAAGACCCACTTCGCAGCCTCGGTGACCGACCCCGAGAGGTTCTGGGGCACAGCTGCCGAGCAGATCAGCTGGTACAAGCCCTGGACCAAAACGCTGGAGAACAAACACTCGCCCTCTACCAG|  |  |  |  |  |  |  |  |  |  |  |  |  |  |  |  |  |  |  |  |  |  |  |  |  |  |  |  |  |  |  |  |  |  |  |  |  |  |  |  |  |  |  |  |  |  |  |  |  |  |  |  |  |  |  |  |  |  |  |  |  |  |  |  |  |  |  |  |  |  |  |  | | --- | --- | --- | --- | --- | --- | --- | --- | --- | --- | --- | --- | --- | --- | --- | --- | --- | --- | --- | --- | --- | --- | --- | --- | --- | --- | --- | --- | --- | --- | --- | --- | --- | --- | --- | --- | --- | --- | --- | --- | --- | --- | --- | --- | --- | --- | --- | --- | --- | --- | --- | --- | --- | --- | --- | --- | --- | --- | --- | --- | --- | --- | --- | --- | --- | --- | --- | --- | --- | --- | --- | --- | | Gorilla gorGor1 Supercontig\_0155513 18955 19262 - **TT** | ATGAAACCGTCTTGGCTGCAATGTCGTAAAGTCACCAGCGCCGGGGGGCTCGGAGGGCCCTTGCCTGGGTCCTCTCCGGCCCGGGGAGCCGGTGCGGCCCTCAGGGCTTTAGTGGTCCCGGGCCCGCGGGGCGGTCTCGGGGGCCGGGGATGCAGGGCACTGTCCTCCGGCAGTGGCAGCGAGTACAAGACCCACTTCGCAGCCTCGGTGACCGACCCCGAGAGGTTCTGGGGCAAAGCTGCCGAGCAGATCAGCTGGTACAAGCCCTGGACCAAAACGCTGGAGAACAAACACTCGCCTTCTACCAG|  |  |  |  |  |  |  |  |  |  |  |  |  |  |  |  |  |  |  |  |  |  |  |  |  |  |  |  |  |  |  |  |  |  |  |  |  |  |  |  |  |  |  |  |  |  |  |  |  |  |  |  |  |  |  |  |  |  |  |  |  |  |  |  | | --- | --- | --- | --- | --- | --- | --- | --- | --- | --- | --- | --- | --- | --- | --- | --- | --- | --- | --- | --- | --- | --- | --- | --- | --- | --- | --- | --- | --- | --- | --- | --- | --- | --- | --- | --- | --- | --- | --- | --- | --- | --- | --- | --- | --- | --- | --- | --- | --- | --- | --- | --- | --- | --- | --- | --- | --- | --- | --- | --- | --- | --- | --- | --- | | Orangutan ponAbe2 chr12 81619645 81619952 + **TT** | ATGAAACCGTCTTGGCTGCAATGTCATAAAGTCACCAGCGCCGGGGGGCTCGGAGGGCCCTTGCCTGGGTCCTCTCCGGCCCGGGGAGCCAGTGCGGCCCTCAGGGCTTTAGTGGTCCCGGGCCCGCGGGGCGGTCTCGGGGGCCGGGGATGCAGGGCGCTGTCCTCCGGCAGTGGCAGCGAGTACAAGACCCACTTCGCAGCGTCGGTGACCGACCCGGAGAGGTTCTGGGGCAAAGCTGCCGAGCAGATCAGCTGGTACAAGCCCTGGACCAAAACGCTGGAGAACAAACACTCGCCCTCTACCAG|  |  |  |  |  |  |  |  |  |  |  |  |  |  |  |  |  |  |  |  |  |  |  |  |  |  |  |  |  |  |  |  |  |  |  |  |  |  |  |  |  |  |  |  |  |  |  |  |  |  |  |  |  |  |  |  | | --- | --- | --- | --- | --- | --- | --- | --- | --- | --- | --- | --- | --- | --- | --- | --- | --- | --- | --- | --- | --- | --- | --- | --- | --- | --- | --- | --- | --- | --- | --- | --- | --- | --- | --- | --- | --- | --- | --- | --- | --- | --- | --- | --- | --- | --- | --- | --- | --- | --- | --- | --- | --- | --- | --- | --- | | Rhesus rheMac2 chr11 78037995 78038302 + **TT** | ATGAAACCCTCTTGGCTGCAATGTCGTAAAGTCACCGGCGCCGGGGGGCTCGGAGGGACCTTGCCTGGGTCCTCTCCGGCCCGGGGAGCCGGTGCGGCCCTCAGGGCTTTAGTggccccgggcccgcggggcggTCTTGGAGGTCGGGGCTGCAGGGCGCTGTCCTCCGGCAGTGGCAGCGAGTACAAGACCCACTTCACAGCCTCGGTGACCGACCCCGAGAGGTTCTGGGGCAAAGCTGCAGAGCAGATCAGCTGGTACAAGCCCTGGACCAAAACGCTGGAGAACAAACACTCGCCCTCTACCAG|  |  |  |  |  |  |  |  |  |  |  |  |  |  |  |  |  |  |  |  |  |  |  |  |  |  |  |  |  |  |  |  |  |  |  |  |  |  |  |  |  |  |  |  |  |  |  |  | | --- | --- | --- | --- | --- | --- | --- | --- | --- | --- | --- | --- | --- | --- | --- | --- | --- | --- | --- | --- | --- | --- | --- | --- | --- | --- | --- | --- | --- | --- | --- | --- | --- | --- | --- | --- | --- | --- | --- | --- | --- | --- | --- | --- | --- | --- | --- | --- | | Baboon papHam1 scaffold24807 13102 13409 + **TT** | ATGAAACCCTCTTGGCTGCAATGTCGTAAAGTCACCGGCGCCGGGGGGCTCGGAGGGACCTTGCCTGGGTCCTCTCCGGCCCGGGGAGCCGGTGCGGCCCTCAGGGCTTTAGTGGCCCCAGGCCCGCGGGGCGGTCTCGGAGGTCGGGGCTGCAGGGCGCTGTCCTCCGGCAGTGGCAGCGAGTACAAGACCCACTTCACAGCCTCGGTGACCGACCCCGAGAGGTTCTGGGGCAAAGCTGCTGAGCAGATCAGCTGGTACAAGCCCTGGACCAAAACGCTGGAGAACAAACACTCGCCCTCTACCAG|  |  |  |  |  |  |  |  |  |  |  |  |  |  |  |  |  |  |  |  |  |  |  |  |  |  |  |  |  |  |  |  |  |  |  |  |  |  |  |  | | --- | --- | --- | --- | --- | --- | --- | --- | --- | --- | --- | --- | --- | --- | --- | --- | --- | --- | --- | --- | --- | --- | --- | --- | --- | --- | --- | --- | --- | --- | --- | --- | --- | --- | --- | --- | --- | --- | --- | --- | | Marmoset calJac1 Contig4152 128699 129006 - **TT** | ATGAAACCGTCTTGGCTGCAATACCGTAAAGTCACCGGCGCCGGGGGGCTCGGAGGGCCTTTGCCCGGGTCTTCTGCGGCCCGGGGAGCCGGTGCGGCCCGCAGGGCTTTCGTGGCCCCGGGTCCGCGGGGTGGTCTCGGGACCCTGGGCTGCAGAGCGCTGTCCTCGGGCAGTGGCAGCGAGTACAAGACCCACTTCGCCGCCTCGGTGACGGACCCCGAGAGGTTCTGGGGCAAAGCTGCTGAGCAGATCAGCTGGTACAAGCCCTGGACCAAAACGCTGGAGAACAAACACTCGCCCTCCACCAG|  |  |  |  |  |  |  |  |  |  |  |  |  |  |  |  |  |  |  |  |  |  |  |  |  |  |  |  |  |  |  |  | | --- | --- | --- | --- | --- | --- | --- | --- | --- | --- | --- | --- | --- | --- | --- | --- | --- | --- | --- | --- | --- | --- | --- | --- | --- | --- | --- | --- | --- | --- | --- | --- | | Tarsier tarSyr1 scaffold\_19051 14794 15101 + **TT** | ATGAAACCGTCTTGGCTGCAGTATCGCAAAGTCACCGGCGCCGGGGGCCTCGGGGGTCCCCTGCCCGGGTCCTCTGCGGCCAGGGGCGCCGGAGCGGCCCTCAGGGCGTACGTGGCTCCCGGCCCCCGGGCTGGCCTCGGGGGCCGGGGCTGCAGGGCGCTGTCCTCAGGCAGCGGCGGCGAGTACAGGACCCACTTCGCAGCCTCGGTGGGCGACCCCGAGAGGTTCTGGGGCAAAGCTGCGGAGCAGATCAGCTGGTACAAGCCCTGGACCAAAACGCTGGAGAACAGACACCCGCCTTTCACCAG|  |  |  |  |  |  |  |  |  |  |  |  |  |  |  |  |  |  |  |  |  |  |  |  | | --- | --- | --- | --- | --- | --- | --- | --- | --- | --- | --- | --- | --- | --- | --- | --- | --- | --- | --- | --- | --- | --- | --- | --- | | Lemur micMur1 scaffold\_2130 179455 179762 + **TT** | ATGAAACCGTCTTGGCTACAATGTCGCAAAGTCAccggcgccgggggcctcggggggcccctgcccgggTCCCCTCCAGCCCGGGGAGCCGGTGCGGCCCGCAGGGCTTTGGTGGCCCCGGGCCCGTGGGGCGCTGTCGGGGGCCGGGGCTGCAGGGCGCTGGCCTCGGGCAGCGTGGGCGAGTACAAGACGCACTTCGCGGCCTCGCTGGCCGACCCCGAGAGGTTCTGGGGCAAAGCTGCCGAGCAGATCAGCTGGTACAAGCCCTGGACCAAAACGCTAGAGAACAGACACCCTCCTTCCACCAG|  |  |  |  |  |  |  |  |  |  |  |  |  |  |  |  | | --- | --- | --- | --- | --- | --- | --- | --- | --- | --- | --- | --- | --- | --- | --- | --- | | Mouse mm9 chr10 106560347 106560639 - **TT** | ATGAAGCCATCCTGGTTGCAATGTCGCAAAGTAACAGGCGCCGGGACGCTTGGGGCACCTCTGCCAGGATCCCCTTCAGTAAGAGGAGCCGCTGTGACCCGAAGGGCTTTGGTGGCCGGGTTTGGGGGCAGGGGCTGCAGGGCTCTGACCACCGGCAGTGGTGGTGAGTACAAGACCCACTTCGCAGCCTCTGTGGCTGACCCCGAAAGATTCTGGGGTAAAGCTGCTGAGCAGATCAGTTGGTACAAGCCCTGGACCAAAACTCTAGAGAGCAGATACCCGCCTTCCACAAG|  |  |  |  |  |  |  |  | | --- | --- | --- | --- | --- | --- | --- | --- | | Cow bosTau4 chr5 12742310 12742614 + **TT** | ATGAAGCCGTCTTGGCTGCAGTGTCGCAAAGTCACCGGCGCGGGGGGCCTCGGCGGGTCCCTGCCCGCGTCCTCTcccgcgcgcggagccggcccggcccgccgggcgtacgtggcccccggcccgcggggcgctctcgggggccggggcTGCAGGGCGCTGTCCTCGGGCGGCGGCGAGTACAAGACCCACTTCGCGGCCTCGGTGACCGACCCCGAGAGGTTCTGGGGCAAAGCCGCGGAGCAGATCAGCTGGTACAAGCCCTGGACCAAGACGCTGGAGAACAGACATTCGCCTTCCACCAG | | | | | | | | | | | | | | | | | | | | | | | | | | | | | | | | | | | | | | | | | | | | | | | | | | | | | | | | | | | | | | | | | | | | | | | | | | | | | | | | | | | | |

**Alignment** (splice site sequences are in lowercase)  

```
Human      ATGAAACCGTCTTGGCTGCAGTGTCGTAAAGTCACCAGCGCCGGGGGGCTCGGAGGGCCCTTGCCTGGGTCCTCTCCGGC
Chimp      ....................A...................................A.......................
Gorilla    ....................A...........................................................
Orangutan  ....................A....A......................................................
Rhesus     ........C...........A...............G....................A......................
Baboon     ........C...........A...............G....................A......................
Marmoset   ....................A.AC............G......................T.....C.....T...G....
Tarsier    ......................A...C.........G..........C.....G..T...C....C.........G....
Lemur      .................A..A.....C.........G..........C.....G......C....C......C....A..
Mouse      .....G..A..C...T....A.....C.....A..AG........AC...T..G.CA..TC....A..A...C..T.A.T
Cow        .....G....................C.........G....G.....C.....C...T..C....C.C.........C..

Human      CCGGGGAGCCGGTGCGGCCCTCAGGGCTTTAGTGGTCCCGGGCCCGCGGGGCGGTCTCGGGGGCCGGGGATGCAGGGCAC
Chimp      ................................................................................
Gorilla    ................................................................................
Orangutan  ..........A...................................................................G.
Rhesus     ...................................C.....................T..A..T.....C........G.
Baboon     ...................................C...A....................A..T.....C........G.
Marmoset   ....................G.........C....C......T........T.........AC..T...C.....A..G.
Tarsier    .A....C.....A..............G.AC....CT..C.....C....CT..C..............C........G.
Lemur      ....................G.........G....C..........T......C.G.............C........G.
Mouse      AA.A.......C..T.A...GA........G....C.---------------..GT.T......A....C........T.
Cow        G..C........CC......G.C....G.AC....C...C.............C...............C........G.

Human      TGTCCTCCGGCAGTGGCAGCGAGTACAAGACCCACTTCGCAGCCTCGGTGACCGACCCCGAGAGGTTCTGGGGCAAAGCT
Chimp      ...........................................................................C....
Gorilla    ................................................................................
Orangutan  ...........................................G..............G.....................
Rhesus     ......................................A.........................................
Baboon     ......................................A.........................................
Marmoset   .......G................................C...........G...........................
Tarsier    .......A.....C...G.........G......................GG............................
Lemur      ..G....G.....C.TGG.............G........G......C..G.............................
Mouse      ..A..A..........TG.T..........................T...G.T........A..A........T......
Cow        .......G...---...G......................G......................................C

Human      GCCGAGCAGATCAGCTGGTACAAGCCCTGGACCAAAACGCTGGAGAACAAACACTCGCCCTCTACCAGgt
Chimp      ....................................................................t.
Gorilla    ...........................................................T........t.
Orangutan  ....................................................................t.
Rhesus     ..A.................................................................t.
Baboon     ..T.................................................................t.
Marmoset   ..T...........................................................C.....t.
Tarsier    ..G..............................................G....C....T.TC.....t.
Lemur      .........................................A.......G....C.T..T..C.....t.
Mouse      ..T...........T.......................T..A....G..G.T..C....T..C..A..t.
Cow        ..G................................G.............G...T.....T..C.....t.
```

---

## 26. uc001wxi.1\_1\_3

**Summary**  

|  |  |  |  |  |  |  |  |  |  |  |  |  |  |  |  |  |  |  |  |  |  |  |  |  |  |
| --- | --- | --- | --- | --- | --- | --- | --- | --- | --- | --- | --- | --- | --- | --- | --- | --- | --- | --- | --- | --- | --- | --- | --- | --- | --- |
| No Exon ID Position (hg19) Dir Human donor Chimp donor Category Usage Gene symbol Protein accession mRNA accession Gene title Note|  |  |  |  |  |  |  |  |  |  |  |  |  | | --- | --- | --- | --- | --- | --- | --- | --- | --- | --- | --- | --- | --- | | 26 uc001wxi.1\_1\_3 chr14:50472312 - GT CT (D7) exonization; novel start constitutive C14orf182 NP\_001012724.1 NM\_001012706.1 chromosome 14 open reading frame 182 de novo protein | | | | | | | | | | | | | | | | | | | | | | | | | |

**Orthologs**  

|  |  |  |  |  |  |  |  |  |  |  |  |  |  |  |  |  |  |  |  |  |  |  |  |  |  |  |  |  |  |  |  |  |  |  |  |  |  |  |  |  |  |  |  |  |  |  |  |  |  |  |  |  |  |  |  |  |  |  |  |  |  |  |  |
| --- | --- | --- | --- | --- | --- | --- | --- | --- | --- | --- | --- | --- | --- | --- | --- | --- | --- | --- | --- | --- | --- | --- | --- | --- | --- | --- | --- | --- | --- | --- | --- | --- | --- | --- | --- | --- | --- | --- | --- | --- | --- | --- | --- | --- | --- | --- | --- | --- | --- | --- | --- | --- | --- | --- | --- | --- | --- | --- | --- | --- | --- | --- | --- |
| Species Assembly Chromosome Exon start Exon end Dir Donor Exon sequence|  |  |  |  |  |  |  |  |  |  |  |  |  |  |  |  |  |  |  |  |  |  |  |  |  |  |  |  |  |  |  |  |  |  |  |  |  |  |  |  |  |  |  |  |  |  |  |  |  |  |  |  |  |  |  |  | | --- | --- | --- | --- | --- | --- | --- | --- | --- | --- | --- | --- | --- | --- | --- | --- | --- | --- | --- | --- | --- | --- | --- | --- | --- | --- | --- | --- | --- | --- | --- | --- | --- | --- | --- | --- | --- | --- | --- | --- | --- | --- | --- | --- | --- | --- | --- | --- | --- | --- | --- | --- | --- | --- | --- | --- | | Human hg19 chr14 50472312 50472517 - **GT** | ATGACAGGTCGGATGGCAACCCTGGAAAAGTCACACAGCTCAGCCTGTTGGAGGAAGAGGAGCAGTAGAACCTGTGTGGAGCCTGACAGAACCCAAGATGCCATTCACGAACCGAGAGGTCTTTCAAGATCACACACAGTTCTACGACACAGACATTTCGTCTTCTTGCCACTCAGTAGCGGGGCCCATCCCTCTGTGCCTCCAAG|  |  |  |  |  |  |  |  |  |  |  |  |  |  |  |  |  |  |  |  |  |  |  |  |  |  |  |  |  |  |  |  |  |  |  |  |  |  |  |  |  |  |  |  |  |  |  |  | | --- | --- | --- | --- | --- | --- | --- | --- | --- | --- | --- | --- | --- | --- | --- | --- | --- | --- | --- | --- | --- | --- | --- | --- | --- | --- | --- | --- | --- | --- | --- | --- | --- | --- | --- | --- | --- | --- | --- | --- | --- | --- | --- | --- | --- | --- | --- | --- | | Chimp panTro2 chr14 49196418 49196623 - **CT** | ATAACAGGTCGGATGGCAACCCTGGAAAAGTCACACAGCTCAGCCTGTTGGAGGAAGAGGAGCAGTAGAACCTGTGTGGAGCCTGACAGAACCCAAGATGCCATTCACGAACCGCGAGGTCTTTCAAGATCACACACAGTTCTACGACACAGACATTTCATCTTCTTGCTACTCAGTAGCGGGGCCCATCCCTCTGTGCCTCCAAG|  |  |  |  |  |  |  |  |  |  |  |  |  |  |  |  |  |  |  |  |  |  |  |  |  |  |  |  |  |  |  |  |  |  |  |  |  |  |  |  | | --- | --- | --- | --- | --- | --- | --- | --- | --- | --- | --- | --- | --- | --- | --- | --- | --- | --- | --- | --- | --- | --- | --- | --- | --- | --- | --- | --- | --- | --- | --- | --- | --- | --- | --- | --- | --- | --- | --- | --- | | Orangutan ponAbe2 chr14 50438720 50438925 - **CT** | ATGACAGGTCGGATGGCAACCCTGGAAAAGTCACACAGCTCAGCCTGTTGGAGGAAGAGGAGCAGTAGAACCTGTGTGGAGCCTGACAGAACCCAAGATGCCATTCACGAACCGAGAGGTCTTTCAAGATCACACACAGTTCTATGACACAGACATTTCGTCTTCTTGCCACTCAGTAGCGGGGCCCGTCCCTCTGTGCCTCCAAG|  |  |  |  |  |  |  |  |  |  |  |  |  |  |  |  |  |  |  |  |  |  |  |  |  |  |  |  |  |  |  |  | | --- | --- | --- | --- | --- | --- | --- | --- | --- | --- | --- | --- | --- | --- | --- | --- | --- | --- | --- | --- | --- | --- | --- | --- | --- | --- | --- | --- | --- | --- | --- | --- | | Rhesus rheMac2 chr7 113073642 113073847 - **CT** | GTGACAGGTTGGATGGCAACCCTGGAAAAGCCACACACCTTGGCCTGTTGGAGGAAGAGGAGCAGTAGAACCTGCGTGGAGCCTGACAGAACCCAAGATGCCGTTCAGGAACCGAGAGGCCTTCCAAGATCACACACAGTTCTACAACACAGACATTTCGTCTTCTTGCCACTTAGCAGAGGGGCCCATCCCTCTGTGCCTCCAAG|  |  |  |  |  |  |  |  |  |  |  |  |  |  |  |  |  |  |  |  |  |  |  |  | | --- | --- | --- | --- | --- | --- | --- | --- | --- | --- | --- | --- | --- | --- | --- | --- | --- | --- | --- | --- | --- | --- | --- | --- | | Baboon papHam1 scaffold19136 24672 24877 - **CT** | GTGACAGGTTGGATGGCAACCCTGGAAAAGCCACACACCTTGGCCTGTTGGAGGAAGAGGAGCAGTAGAACCTGCGTGGAGCCTGACAGAACCCAAGATGCCGTTCAGGAACCGAGAGGCCTTTCAAGATCACACACAGTTCTACAACACAGACATTTCGTCTTCTTGCCACTTAGCAGCGGGGCCCATCCCTCTGTGCCTCCAAG|  |  |  |  |  |  |  |  |  |  |  |  |  |  |  |  | | --- | --- | --- | --- | --- | --- | --- | --- | --- | --- | --- | --- | --- | --- | --- | --- | | Lemur micMur1 scaffold\_2039 125594 125769 - **CT** | ATGATGGGTCTGATGGCAACCCAGGAAAAGCCACACAGCTCAGCCTATTGGAGAAAGAGGAGCAACAGAACCCGTGTGGAGCCCGACAGAGCCCAGCACATAGCTCAGGAACCAAGAGGTCCTTCAAGATCAAACCCAGCTCACCAGCAGCAGGGACCATCCATTGCGCCTCCCAG|  |  |  |  |  |  |  |  | | --- | --- | --- | --- | --- | --- | --- | --- | | Galago otoGar1 scaffold\_115424.1-163071 721 892 - **CT** | AAGATGTGTCCGATGGCAACCCAGGAAAAGTCCCGAGGCTCGGCCTGGTGGAGGAAGAGCAGCAGAACCTGGGTGGAGGCCGATAGTATCCAGGACCATGTCTCAGGAACCAAGAGGTCTTTCCAGATTAAACACATCTTACCAGCAGCAGGGATGGGCCCTTGTCCCCATG | | | | | | | | | | | | | | | | | | | | | | | | | | | | | | | | | | | | | | | | | | | | | | | | | | | | | | | | |

**Alignment** (splice site sequences are in lowercase)  

```
Human      ATGACAGGTCGGATGGCAACCCTGGAAAAGTCACACAGCTCAGCCTGTTGGAGGAAGAGGAGCAGTAGAACCTGTGTGGA
Chimp      ..A.............................................................................
Orangutan  ................................................................................
Rhesus     G........T....................C......C..TG................................C.....
Baboon     G........T....................C......C..TG................................C.....
Lemur      ....TG....T...........A.......C...............A......A..........AC......C.......
Galago     .A..TGT...C...........A.........C.GAG....G.....G........---......C........G.....

Human      GCCTGACAGAACCCAAGA-TGCCATTCACGAACCGAGAGGTCTTTCAAGATCACACACAGTTCTACGACACAGACATTTC
Chimp      ..................-................C............................................
Orangutan  ..................-..............................................T..............
Rhesus     ..................-....G....G...........C...C.....................A.............
Baboon     ..................-....G....G...........C.........................A.............
Lemur      ...C......G....GC.-CATAGC...G.....A.......C..........A..C...C..-----------------
Galago     .G.C..T..T.T...G..CCATGTC...G.....A...........C....T.A.....TC.T-----------------

Human      GTCTTCTTGCCACTCAGTAGCGGGGCCCATCCCTCTGTGCCTCCAAGgt
Chimp      A.........T....................................c.
Orangutan  ............................G..................c.
Rhesus     ..............T..C..A..........................c.
Baboon     ..............T..C.............................c.
Lemur      ------------AC...C...A...A......A.-..C......C..c.
Galago     ------------AC...C...A...ATGGG....---..T.C...T.c.
```

---

## 27. uc010tyr.1\_1\_8

**Summary**  

|  |  |  |  |  |  |  |  |  |  |  |  |  |  |  |  |  |  |  |  |  |  |  |  |  |  |
| --- | --- | --- | --- | --- | --- | --- | --- | --- | --- | --- | --- | --- | --- | --- | --- | --- | --- | --- | --- | --- | --- | --- | --- | --- | --- |
| No Exon ID Position (hg19) Dir Human donor Chimp donor Category Usage Gene symbol Protein accession mRNA accession Gene title Note|  |  |  |  |  |  |  |  |  |  |  |  |  | | --- | --- | --- | --- | --- | --- | --- | --- | --- | --- | --- | --- | --- | | 27 uc010tyr.1\_1\_8 chr14:105939849 + GT GC (D7) exonization; novel start alternative CRIP2 B7Z6C0 AK300092.1 cysteine-rich protein 2  | | | | | | | | | | | | | | | | | | | | | | | | | |

**Orthologs**  

|  |  |  |  |  |  |  |  |  |  |  |  |  |  |  |  |  |  |  |  |  |  |  |  |  |  |  |  |  |  |  |  |  |  |  |  |  |  |  |  |  |  |  |  |  |  |  |  |
| --- | --- | --- | --- | --- | --- | --- | --- | --- | --- | --- | --- | --- | --- | --- | --- | --- | --- | --- | --- | --- | --- | --- | --- | --- | --- | --- | --- | --- | --- | --- | --- | --- | --- | --- | --- | --- | --- | --- | --- | --- | --- | --- | --- | --- | --- | --- | --- |
| Species Assembly Chromosome Exon start Exon end Dir Donor Exon sequence|  |  |  |  |  |  |  |  |  |  |  |  |  |  |  |  |  |  |  |  |  |  |  |  |  |  |  |  |  |  |  |  |  |  |  |  |  |  |  |  | | --- | --- | --- | --- | --- | --- | --- | --- | --- | --- | --- | --- | --- | --- | --- | --- | --- | --- | --- | --- | --- | --- | --- | --- | --- | --- | --- | --- | --- | --- | --- | --- | --- | --- | --- | --- | --- | --- | --- | --- | | Human hg19 chr14 105939585 105939849 + **GT** | ATGCCTCCTCACCATCTTCTGCCTTGGCTGGCACAGGTCCCAAGTGCAGAGGGGGAGCTGGTCAGGCTGGTCAGTCGGGCGGGGGGGCGGGGGGCATGTTTCTGGCCTGCTGTCACCATGGAAATGGCAGTGGCTGCAGGGTGTGTGTGCAAGGGAGGAGGGTGCTGCCACAGAGAGCCCAGCCAGGACCACCATGAAAGCCAGGAGCACCGAGGGCCTCTGGTTGGCTCACAAACATGCCTGGTGCACCAGGCAGAGGGAACAG|  |  |  |  |  |  |  |  |  |  |  |  |  |  |  |  |  |  |  |  |  |  |  |  |  |  |  |  |  |  |  |  | | --- | --- | --- | --- | --- | --- | --- | --- | --- | --- | --- | --- | --- | --- | --- | --- | --- | --- | --- | --- | --- | --- | --- | --- | --- | --- | --- | --- | --- | --- | --- | --- | | Chimp panTro2 chr14 106009367 106009631 + **GC** | ATGCCTCCTTGCCATCTTCTGCCTTGGCTGGCACAGGTCCCAAGTGCAGAGGGGGAGCTGGTCAGGCTGGTCAGTTGGGCGGGGGGGCGGGGGGCATGTTTCTGGCCTGCTGTCACCATGGAAATGGCAGTGGCTGCAGGGTGTGTGTGCAAGGGAGGAGGGTGCTGCCACAGAGAGCCCAGCCAGGACCACCATGAAAGCCAGGAGCACCGAGGGCCTCTGGTTGGCTCACAAACATGCCTGGTGCACCAGGCAGAGGGAACAG|  |  |  |  |  |  |  |  |  |  |  |  |  |  |  |  |  |  |  |  |  |  |  |  | | --- | --- | --- | --- | --- | --- | --- | --- | --- | --- | --- | --- | --- | --- | --- | --- | --- | --- | --- | --- | --- | --- | --- | --- | | Orangutan ponAbe2 chr14 107255556 107255820 + **GC** | ATGCCTCCTCGCCATCTTCTGCCTTGGCTGGCACAGGTCCCAAGTGCAGAGGGGGAGCTGGTCAGGCTGGTCAGTCGGGCGTGGGGGCGGGGGGCATGTTTCTGGCCTGCTGTTACCATGGAAATGGCAGTGGCTGCAGGGTGTGTGTGCAAGGGAGGAGGGTGCTGCCATAGAGAGCCCGGCCAGGGCCACCATGAAAGCCAGGAGCACCGAGGGCCACTGGTTGGCTCACAAACATGCCTGGTGCACCAGGCAGAGGAAACAG|  |  |  |  |  |  |  |  |  |  |  |  |  |  |  |  | | --- | --- | --- | --- | --- | --- | --- | --- | --- | --- | --- | --- | --- | --- | --- | --- | | Rhesus rheMac2 chr7 168836280 168836540 + **GC** | ATGCCTCCTCGCCATCTTCTGCCTTGGCTGGCACAGATCCCAAGTGCAGAGGGGGAGCTGGTCAGGCTGGTCAGTCCGGCGTGGGGGCGGGGGGCATGTTTCTGGCCTGCGGTCACCATGGAAATGGCAGTGGCTGCAGGGTGTGTGTGCAAGGGAGGAGGGTGCTGCCACAGGGAGCCCTGCCAGGGCCACCATGAAAGCCAGGAGTGCCTAGGGCCACTGGCTCACAGACATGCCTGGTGCACCAGGCAGAGGAAACAG|  |  |  |  |  |  |  |  | | --- | --- | --- | --- | --- | --- | --- | --- | | Baboon papHam1 scaffold9107 50694 50954 + **GC** | ATGTCTCCTCGCCATCTTCTGCCTTGGCTGGCACAGATCCCAAGTGCAGAGGGGGAGCTGATCAGGCTGGTCAGTCCGGTGTGGGGGCGGGGGGCATGTTTCTGGCCTGCTGTCACCATGGAAATGGCAGTGGCTGCAGGGTGCGTATGCAAGGGAGGAGGGTGCTGCCACAGGGAGCCCTGCCAGGGCCACCATGAAAGCCAGGAGTGCCTAGGGCCACTGGCTCACAGACATGCCTGGTGCACCAGGCAGAGGAAACAG | | | | | | | | | | | | | | | | | | | | | | | | | | | | | | | | | | | | | | | | | | |

**Alignment** (splice site sequences are in lowercase)  

```
Human      ATGCCTCCTCACCATCTTCTGCCTTGGCTGGCACAGGTCCCAAGTGCAGAGGGGGAGCTGGTCAGGCTGGTCAGTCGGGC
Chimp      .........TG................................................................T....
Orangutan  ..........G.....................................................................
Rhesus     ..........G.........................A.......................................C...
Baboon     ...T......G.........................A.......................A...............C..T

Human      GGGGGGGCGGGGGGCATGTTTCTGGCCTGCTGTCACCATGGAAATGGCAGTGGCTGCAGGGTGTGTGTGCAAGGGAGGAG
Chimp      ................................................................................
Orangutan  .T...............................T..............................................
Rhesus     .T............................G.................................................
Baboon     .T.............................................................C..A.............

Human      GGTGCTGCCACAGAGAGCCCAGCCAGGACCACCATGAAAGCCAGGAGCACCGAGGGCCTCTGGTTGGCTCACAAACATGC
Chimp      ................................................................................
Orangutan  ..........T.........G......G..............................A.....................
Rhesus     .............G......T......G...................TG..T......A.----.........G......
Baboon     .............G......T......G...................TG..T......A.----.........G......

Human      CTGGTGCACCAGGCAGAGGGAACAGgt
Chimp      ..........................c
Orangutan  ...................A......c
Rhesus     ...................A......c
Baboon     ...................A......c
```

---

## 28. uc010ujw.1\_1\_8

**Summary**  

|  |  |  |  |  |  |  |  |  |  |  |  |  |  |  |  |  |  |  |  |  |  |  |  |  |  |
| --- | --- | --- | --- | --- | --- | --- | --- | --- | --- | --- | --- | --- | --- | --- | --- | --- | --- | --- | --- | --- | --- | --- | --- | --- | --- |
| No Exon ID Position (hg19) Dir Human donor Chimp donor Category Usage Gene symbol Protein accession mRNA accession Gene title Note|  |  |  |  |  |  |  |  |  |  |  |  |  | | --- | --- | --- | --- | --- | --- | --- | --- | --- | --- | --- | --- | --- | | 28 uc010ujw.1\_1\_8 chr15:67841545 + GT TT (D7) exonization; novel start alternative MAP2K5 NP\_001193733.1 NM\_001206804.1 mitogen-activated protein kinase kinase 5  | | | | | | | | | | | | | | | | | | | | | | | | | |

**Orthologs**  

|  |  |  |  |  |  |  |  |  |  |  |  |  |  |  |  |  |  |  |  |  |  |  |  |  |  |  |  |  |  |  |  |  |  |  |  |  |  |  |  |  |  |  |  |  |  |  |  |  |  |  |  |  |  |  |  |  |  |  |  |  |  |  |  |  |  |  |  |  |  |  |  |  |  |  |  |  |  |  |  |  |  |  |  |  |  |  |  |
| --- | --- | --- | --- | --- | --- | --- | --- | --- | --- | --- | --- | --- | --- | --- | --- | --- | --- | --- | --- | --- | --- | --- | --- | --- | --- | --- | --- | --- | --- | --- | --- | --- | --- | --- | --- | --- | --- | --- | --- | --- | --- | --- | --- | --- | --- | --- | --- | --- | --- | --- | --- | --- | --- | --- | --- | --- | --- | --- | --- | --- | --- | --- | --- | --- | --- | --- | --- | --- | --- | --- | --- | --- | --- | --- | --- | --- | --- | --- | --- | --- | --- | --- | --- | --- | --- | --- | --- |
| Species Assembly Chromosome Exon start Exon end Dir Donor Exon sequence|  |  |  |  |  |  |  |  |  |  |  |  |  |  |  |  |  |  |  |  |  |  |  |  |  |  |  |  |  |  |  |  |  |  |  |  |  |  |  |  |  |  |  |  |  |  |  |  |  |  |  |  |  |  |  |  |  |  |  |  |  |  |  |  |  |  |  |  |  |  |  |  |  |  |  |  |  |  |  |  | | --- | --- | --- | --- | --- | --- | --- | --- | --- | --- | --- | --- | --- | --- | --- | --- | --- | --- | --- | --- | --- | --- | --- | --- | --- | --- | --- | --- | --- | --- | --- | --- | --- | --- | --- | --- | --- | --- | --- | --- | --- | --- | --- | --- | --- | --- | --- | --- | --- | --- | --- | --- | --- | --- | --- | --- | --- | --- | --- | --- | --- | --- | --- | --- | --- | --- | --- | --- | --- | --- | --- | --- | --- | --- | --- | --- | --- | --- | --- | --- | | Human hg19 chr15 67841519 67841545 + **GT** | ATGATGGAGGGTCACTTTCCCCAGAGC|  |  |  |  |  |  |  |  |  |  |  |  |  |  |  |  |  |  |  |  |  |  |  |  |  |  |  |  |  |  |  |  |  |  |  |  |  |  |  |  |  |  |  |  |  |  |  |  |  |  |  |  |  |  |  |  |  |  |  |  |  |  |  |  |  |  |  |  |  |  |  |  | | --- | --- | --- | --- | --- | --- | --- | --- | --- | --- | --- | --- | --- | --- | --- | --- | --- | --- | --- | --- | --- | --- | --- | --- | --- | --- | --- | --- | --- | --- | --- | --- | --- | --- | --- | --- | --- | --- | --- | --- | --- | --- | --- | --- | --- | --- | --- | --- | --- | --- | --- | --- | --- | --- | --- | --- | --- | --- | --- | --- | --- | --- | --- | --- | --- | --- | --- | --- | --- | --- | --- | --- | | Chimp panTro2 chr15 65257274 65257300 + **TT** | ATGATGGAGGGTCACTTTCCCCAGAGC|  |  |  |  |  |  |  |  |  |  |  |  |  |  |  |  |  |  |  |  |  |  |  |  |  |  |  |  |  |  |  |  |  |  |  |  |  |  |  |  |  |  |  |  |  |  |  |  |  |  |  |  |  |  |  |  |  |  |  |  |  |  |  |  | | --- | --- | --- | --- | --- | --- | --- | --- | --- | --- | --- | --- | --- | --- | --- | --- | --- | --- | --- | --- | --- | --- | --- | --- | --- | --- | --- | --- | --- | --- | --- | --- | --- | --- | --- | --- | --- | --- | --- | --- | --- | --- | --- | --- | --- | --- | --- | --- | --- | --- | --- | --- | --- | --- | --- | --- | --- | --- | --- | --- | --- | --- | --- | --- | | Gorilla gorGor1 Supercontig\_0000754 44665 44691 + **TT** | ATGATGGAGGGTCACTTTCCCCAGAGC|  |  |  |  |  |  |  |  |  |  |  |  |  |  |  |  |  |  |  |  |  |  |  |  |  |  |  |  |  |  |  |  |  |  |  |  |  |  |  |  |  |  |  |  |  |  |  |  |  |  |  |  |  |  |  |  | | --- | --- | --- | --- | --- | --- | --- | --- | --- | --- | --- | --- | --- | --- | --- | --- | --- | --- | --- | --- | --- | --- | --- | --- | --- | --- | --- | --- | --- | --- | --- | --- | --- | --- | --- | --- | --- | --- | --- | --- | --- | --- | --- | --- | --- | --- | --- | --- | --- | --- | --- | --- | --- | --- | --- | --- | | Orangutan ponAbe2 chr15 64708352 64708378 + **TT** | ATGATGGAGGGTCACTTTCCCCAGAGC|  |  |  |  |  |  |  |  |  |  |  |  |  |  |  |  |  |  |  |  |  |  |  |  |  |  |  |  |  |  |  |  |  |  |  |  |  |  |  |  |  |  |  |  |  |  |  |  | | --- | --- | --- | --- | --- | --- | --- | --- | --- | --- | --- | --- | --- | --- | --- | --- | --- | --- | --- | --- | --- | --- | --- | --- | --- | --- | --- | --- | --- | --- | --- | --- | --- | --- | --- | --- | --- | --- | --- | --- | --- | --- | --- | --- | --- | --- | --- | --- | | Rhesus rheMac2 chr7 46140046 46140072 + **TT** | GTGATGGAGGGTCACTTTTCCCAGAGT|  |  |  |  |  |  |  |  |  |  |  |  |  |  |  |  |  |  |  |  |  |  |  |  |  |  |  |  |  |  |  |  |  |  |  |  |  |  |  |  | | --- | --- | --- | --- | --- | --- | --- | --- | --- | --- | --- | --- | --- | --- | --- | --- | --- | --- | --- | --- | --- | --- | --- | --- | --- | --- | --- | --- | --- | --- | --- | --- | --- | --- | --- | --- | --- | --- | --- | --- | | Baboon papHam1 scaffold1574 131910 131936 - **TT** | GTGATGGAGGGTCACTTTCCCCAGAGT|  |  |  |  |  |  |  |  |  |  |  |  |  |  |  |  |  |  |  |  |  |  |  |  |  |  |  |  |  |  |  |  | | --- | --- | --- | --- | --- | --- | --- | --- | --- | --- | --- | --- | --- | --- | --- | --- | --- | --- | --- | --- | --- | --- | --- | --- | --- | --- | --- | --- | --- | --- | --- | --- | | Marmoset calJac1 Contig3372 128771 128797 - **TT** | ATGATGGACGGTCACTTTCCCCAGAGC|  |  |  |  |  |  |  |  |  |  |  |  |  |  |  |  |  |  |  |  |  |  |  |  | | --- | --- | --- | --- | --- | --- | --- | --- | --- | --- | --- | --- | --- | --- | --- | --- | --- | --- | --- | --- | --- | --- | --- | --- | | Galago otoGar1 scaffold\_105979.1-28627 10120 10146 + **TG** | ATAGTGGAGGGTCACTTTCCCCAGAAC|  |  |  |  |  |  |  |  |  |  |  |  |  |  |  |  | | --- | --- | --- | --- | --- | --- | --- | --- | --- | --- | --- | --- | --- | --- | --- | --- | | Cow bosTau4 chr10 14258568 14258594 + **TG** | ATCATGGAGGGTCACTTTTCCCAGAGC|  |  |  |  |  |  |  |  | | --- | --- | --- | --- | --- | --- | --- | --- | | Dog canFam2 chr30 34664024 34664050 + **TG** | ATAATGGAGGGTCACTTTTCCCAGAAC | | | | | | | | | | | | | | | | | | | | | | | | | | | | | | | | | | | | | | | | | | | | | | | | | | | | | | | | | | | | | | | | | | | | | | | | | | | | | |

**Alignment** (splice site sequences are in lowercase)  

```
Human      ATGATGGAGGGTCACTTTCCCCAGAGCgt
Chimp      ...........................t.
Gorilla    ...........................t.
Orangutan  ...........................t.
Rhesus     G.................T.......Tt.
Baboon     G.........................Tt.
Marmoset   ........C..................t.
Galago     ..AG.....................A.tg
Cow        ..C...............T........tg
Dog        ..A...............T......A.tg
```

---

## 29. uc002ayt.1\_10\_14

**Summary**  

|  |  |  |  |  |  |  |  |  |  |  |  |  |  |  |  |  |  |  |  |  |  |  |  |  |  |
| --- | --- | --- | --- | --- | --- | --- | --- | --- | --- | --- | --- | --- | --- | --- | --- | --- | --- | --- | --- | --- | --- | --- | --- | --- | --- |
| No Exon ID Position (hg19) Dir Human donor Chimp donor Category Usage Gene symbol Protein accession mRNA accession Gene title Note|  |  |  |  |  |  |  |  |  |  |  |  |  | | --- | --- | --- | --- | --- | --- | --- | --- | --- | --- | --- | --- | --- | | 29 uc002ayt.1\_10\_14 chr15:75114241 + GT GC (D5) exonization; inframe constitutive LMAN1L NP\_068591.2 NM\_021819.2 lectin, mannose-binding, 1 like  | | | | | | | | | | | | | | | | | | | | | | | | | |

**Orthologs**  

|  |  |  |  |  |  |  |  |  |  |  |  |  |  |  |  |  |  |  |  |  |  |  |  |  |  |  |  |  |  |  |  |  |  |  |  |  |  |  |  |  |  |  |  |  |  |  |  |  |  |  |  |  |  |  |  |  |  |  |  |  |  |  |  |  |  |  |  |  |  |  |  |
| --- | --- | --- | --- | --- | --- | --- | --- | --- | --- | --- | --- | --- | --- | --- | --- | --- | --- | --- | --- | --- | --- | --- | --- | --- | --- | --- | --- | --- | --- | --- | --- | --- | --- | --- | --- | --- | --- | --- | --- | --- | --- | --- | --- | --- | --- | --- | --- | --- | --- | --- | --- | --- | --- | --- | --- | --- | --- | --- | --- | --- | --- | --- | --- | --- | --- | --- | --- | --- | --- | --- | --- |
| Species Assembly Chromosome Exon start Exon end Dir Donor Exon sequence|  |  |  |  |  |  |  |  |  |  |  |  |  |  |  |  |  |  |  |  |  |  |  |  |  |  |  |  |  |  |  |  |  |  |  |  |  |  |  |  |  |  |  |  |  |  |  |  |  |  |  |  |  |  |  |  |  |  |  |  |  |  |  |  | | --- | --- | --- | --- | --- | --- | --- | --- | --- | --- | --- | --- | --- | --- | --- | --- | --- | --- | --- | --- | --- | --- | --- | --- | --- | --- | --- | --- | --- | --- | --- | --- | --- | --- | --- | --- | --- | --- | --- | --- | --- | --- | --- | --- | --- | --- | --- | --- | --- | --- | --- | --- | --- | --- | --- | --- | --- | --- | --- | --- | --- | --- | --- | --- | | Human hg19 chr15 75114170 75114241 + **GT** | GCCCTGGATGCTTCCTGCCAGATTCCATCCACCCCAGGGAGGGGTGGCCACCTCTCCATGTCACTCAATAAG|  |  |  |  |  |  |  |  |  |  |  |  |  |  |  |  |  |  |  |  |  |  |  |  |  |  |  |  |  |  |  |  |  |  |  |  |  |  |  |  |  |  |  |  |  |  |  |  |  |  |  |  |  |  |  |  | | --- | --- | --- | --- | --- | --- | --- | --- | --- | --- | --- | --- | --- | --- | --- | --- | --- | --- | --- | --- | --- | --- | --- | --- | --- | --- | --- | --- | --- | --- | --- | --- | --- | --- | --- | --- | --- | --- | --- | --- | --- | --- | --- | --- | --- | --- | --- | --- | --- | --- | --- | --- | --- | --- | --- | --- | | Chimp panTro2 chr15 72654849 72654920 + **GC** | GCCCTGGATGCTTCCTGCCAGATTCCATCCACCCCAGGGAGGGGTGGCCACCTCTCCATGTCACTCAATAAG|  |  |  |  |  |  |  |  |  |  |  |  |  |  |  |  |  |  |  |  |  |  |  |  |  |  |  |  |  |  |  |  |  |  |  |  |  |  |  |  |  |  |  |  |  |  |  |  | | --- | --- | --- | --- | --- | --- | --- | --- | --- | --- | --- | --- | --- | --- | --- | --- | --- | --- | --- | --- | --- | --- | --- | --- | --- | --- | --- | --- | --- | --- | --- | --- | --- | --- | --- | --- | --- | --- | --- | --- | --- | --- | --- | --- | --- | --- | --- | --- | | Gorilla gorGor1 Supercontig\_0040171 29450 29521 - **GC** | GCCCTGGATGCTTCCTGCCAGATTCCATCCACCCCAGGGAGGGGTGGCCACCTCTCCATGTCACTCAATAAG|  |  |  |  |  |  |  |  |  |  |  |  |  |  |  |  |  |  |  |  |  |  |  |  |  |  |  |  |  |  |  |  |  |  |  |  |  |  |  |  | | --- | --- | --- | --- | --- | --- | --- | --- | --- | --- | --- | --- | --- | --- | --- | --- | --- | --- | --- | --- | --- | --- | --- | --- | --- | --- | --- | --- | --- | --- | --- | --- | --- | --- | --- | --- | --- | --- | --- | --- | | Orangutan ponAbe2 chr15 72232961 72233032 + **GC** | GCCTTGGACTCTTCCTGCCAGATTCCATCCACCCCAGGGAGGGGTGGCCACCTCTCCATGTCACTCAATAAG|  |  |  |  |  |  |  |  |  |  |  |  |  |  |  |  |  |  |  |  |  |  |  |  |  |  |  |  |  |  |  |  | | --- | --- | --- | --- | --- | --- | --- | --- | --- | --- | --- | --- | --- | --- | --- | --- | --- | --- | --- | --- | --- | --- | --- | --- | --- | --- | --- | --- | --- | --- | --- | --- | | Lemur micMur1 scaffold\_3915 77454 77525 - **GC** | GCCCTGCACCCCTCCTGCCAGGTTCCATCAGCCCCAGAGAAACGTGGCCACCTCTCCAGGTCACTCAGCGAG|  |  |  |  |  |  |  |  |  |  |  |  |  |  |  |  |  |  |  |  |  |  |  |  | | --- | --- | --- | --- | --- | --- | --- | --- | --- | --- | --- | --- | --- | --- | --- | --- | --- | --- | --- | --- | --- | --- | --- | --- | | Mouse mm9 chr9 57458203 57458274 - **AC** | GCCCTGGACCCCTCTTGCTTGATTGCACTCACCCTCAGAAATGTCAGCTACCTCTCCAGGTCCTTCTGCCAG|  |  |  |  |  |  |  |  |  |  |  |  |  |  |  |  | | --- | --- | --- | --- | --- | --- | --- | --- | --- | --- | --- | --- | --- | --- | --- | --- | | Cow bosTau4 chr21 33930813 33930884 - **AT** | GCCCAGGACTCCCCCTGCCAGGCTCCACCCACCCCTGAGAGAGGTGGCTCCCTCTCCAGGTCACTGGGCAAG|  |  |  |  |  |  |  |  | | --- | --- | --- | --- | --- | --- | --- | --- | | Dog canFam2 chr30 40881753 40881824 + **AC** | GCCCAGAACTCCTCCTGCCAGGTTCTACCCACCCCGGAGAGGGGCGGTCACTTCTCCAGGCCTCTCAGCGAG | | | | | | | | | | | | | | | | | | | | | | | | | | | | | | | | | | | | | | | | | | | | | | | | | | | | | | | | | | | | | | | |

**Alignment** (splice site sequences are in lowercase)  

```
Human      agGCCCTGGATGCTTCCTGCCAGATTCCATCCACCCCAGGGAGGGGTGGCCACCTCTCCATGTCACTCAATAAGgt
Chimp      ...........................................................................c
Gorilla    ...........................................................................c
Orangutan  .....T....CT...............................................................c
Lemur      ........C.CC.C.........G.......AG......A..AAC...............G........GCG...c
Mouse      ..........CC.C..T...TT....G..CT.....TCA.A.AT.TCA..T.........G...CT..TGCC..ac
Cow        ......A...CT.CC........GC....C.......T.A...A......TC........G......GGGC...a.
Dog        ......A.A.CT.C.........G...T.C.......G.A......C..T...T......G.C.T....GCG..ac
```

---

## 30. uc002fck.2\_4\_9

**Summary**  

|  |  |  |  |  |  |  |  |  |  |  |  |  |  |  |  |  |  |  |  |  |  |  |  |  |  |
| --- | --- | --- | --- | --- | --- | --- | --- | --- | --- | --- | --- | --- | --- | --- | --- | --- | --- | --- | --- | --- | --- | --- | --- | --- | --- |
| No Exon ID Position (hg19) Dir Human donor Chimp donor Category Usage Gene symbol Protein accession mRNA accession Gene title Note|  |  |  |  |  |  |  |  |  |  |  |  |  | | --- | --- | --- | --- | --- | --- | --- | --- | --- | --- | --- | --- | --- | | 30 uc002fck.2\_4\_9 chr16:72863678 - GT GC (D3) shift; decrease; inframe constitutive ZFHX3 NP\_001158238.1 NM\_001164766.1 zinc finger homeobox protein 3  | | | | | | | | | | | | | | | | | | | | | | | | | |

**Orthologs**  

|  |  |  |  |  |  |  |  |  |  |  |  |  |  |  |  |  |  |  |  |  |  |  |  |  |  |  |  |  |  |  |  |  |  |  |  |  |  |  |  |  |  |  |  |  |  |  |  |  |  |  |  |  |  |  |  |  |  |  |  |  |  |  |  |  |  |  |  |  |  |  |  |  |  |  |  |  |  |  |  |  |  |  |  |  |  |  |  |  |  |  |  |  |  |  |  |  |  |  |  |  |  |  |  |
| --- | --- | --- | --- | --- | --- | --- | --- | --- | --- | --- | --- | --- | --- | --- | --- | --- | --- | --- | --- | --- | --- | --- | --- | --- | --- | --- | --- | --- | --- | --- | --- | --- | --- | --- | --- | --- | --- | --- | --- | --- | --- | --- | --- | --- | --- | --- | --- | --- | --- | --- | --- | --- | --- | --- | --- | --- | --- | --- | --- | --- | --- | --- | --- | --- | --- | --- | --- | --- | --- | --- | --- | --- | --- | --- | --- | --- | --- | --- | --- | --- | --- | --- | --- | --- | --- | --- | --- | --- | --- | --- | --- | --- | --- | --- | --- | --- | --- | --- | --- | --- | --- | --- | --- |
| Species Assembly Chromosome Exon start Exon end Dir Donor Exon sequence|  |  |  |  |  |  |  |  |  |  |  |  |  |  |  |  |  |  |  |  |  |  |  |  |  |  |  |  |  |  |  |  |  |  |  |  |  |  |  |  |  |  |  |  |  |  |  |  |  |  |  |  |  |  |  |  |  |  |  |  |  |  |  |  |  |  |  |  |  |  |  |  |  |  |  |  |  |  |  |  |  |  |  |  |  |  |  |  |  |  |  |  |  |  |  |  | | --- | --- | --- | --- | --- | --- | --- | --- | --- | --- | --- | --- | --- | --- | --- | --- | --- | --- | --- | --- | --- | --- | --- | --- | --- | --- | --- | --- | --- | --- | --- | --- | --- | --- | --- | --- | --- | --- | --- | --- | --- | --- | --- | --- | --- | --- | --- | --- | --- | --- | --- | --- | --- | --- | --- | --- | --- | --- | --- | --- | --- | --- | --- | --- | --- | --- | --- | --- | --- | --- | --- | --- | --- | --- | --- | --- | --- | --- | --- | --- | --- | --- | --- | --- | --- | --- | --- | --- | --- | --- | --- | --- | --- | --- | --- | --- | | Human hg19 chr16 72863678 72863758 - **GT** | AAGAAGCCATTGAAGATGTTGAAGGACCCAGTGAAACAGCTGCTGATCCAGAGGAGCTTGCTAAGGACCAAGAGGGCGGAG|  |  |  |  |  |  |  |  |  |  |  |  |  |  |  |  |  |  |  |  |  |  |  |  |  |  |  |  |  |  |  |  |  |  |  |  |  |  |  |  |  |  |  |  |  |  |  |  |  |  |  |  |  |  |  |  |  |  |  |  |  |  |  |  |  |  |  |  |  |  |  |  |  |  |  |  |  |  |  |  |  |  |  |  |  |  |  |  | | --- | --- | --- | --- | --- | --- | --- | --- | --- | --- | --- | --- | --- | --- | --- | --- | --- | --- | --- | --- | --- | --- | --- | --- | --- | --- | --- | --- | --- | --- | --- | --- | --- | --- | --- | --- | --- | --- | --- | --- | --- | --- | --- | --- | --- | --- | --- | --- | --- | --- | --- | --- | --- | --- | --- | --- | --- | --- | --- | --- | --- | --- | --- | --- | --- | --- | --- | --- | --- | --- | --- | --- | --- | --- | --- | --- | --- | --- | --- | --- | --- | --- | --- | --- | --- | --- | --- | --- | | Chimp panTro2 chr16 72709211 72709291 - **GC** | AAGAAGCCATTGAAGATGTTGAAGGACCCAGTGAAACAGCTGCTGATCCAGAGGAGCTTGCTAAGGACCAAGAGGGCGGAG|  |  |  |  |  |  |  |  |  |  |  |  |  |  |  |  |  |  |  |  |  |  |  |  |  |  |  |  |  |  |  |  |  |  |  |  |  |  |  |  |  |  |  |  |  |  |  |  |  |  |  |  |  |  |  |  |  |  |  |  |  |  |  |  |  |  |  |  |  |  |  |  |  |  |  |  |  |  |  |  | | --- | --- | --- | --- | --- | --- | --- | --- | --- | --- | --- | --- | --- | --- | --- | --- | --- | --- | --- | --- | --- | --- | --- | --- | --- | --- | --- | --- | --- | --- | --- | --- | --- | --- | --- | --- | --- | --- | --- | --- | --- | --- | --- | --- | --- | --- | --- | --- | --- | --- | --- | --- | --- | --- | --- | --- | --- | --- | --- | --- | --- | --- | --- | --- | --- | --- | --- | --- | --- | --- | --- | --- | --- | --- | --- | --- | --- | --- | --- | --- | | Gorilla gorGor1 Supercontig\_0462669 312 392 + **GC** | AAGAAGCCATTGAAGATGTGGAAGGACCCAGTGAAACAGCTGCTGATCCAGAGGAGCTTGCTAAGGACCAAGAGGGCGGAG|  |  |  |  |  |  |  |  |  |  |  |  |  |  |  |  |  |  |  |  |  |  |  |  |  |  |  |  |  |  |  |  |  |  |  |  |  |  |  |  |  |  |  |  |  |  |  |  |  |  |  |  |  |  |  |  |  |  |  |  |  |  |  |  |  |  |  |  |  |  |  |  | | --- | --- | --- | --- | --- | --- | --- | --- | --- | --- | --- | --- | --- | --- | --- | --- | --- | --- | --- | --- | --- | --- | --- | --- | --- | --- | --- | --- | --- | --- | --- | --- | --- | --- | --- | --- | --- | --- | --- | --- | --- | --- | --- | --- | --- | --- | --- | --- | --- | --- | --- | --- | --- | --- | --- | --- | --- | --- | --- | --- | --- | --- | --- | --- | --- | --- | --- | --- | --- | --- | --- | --- | | Orangutan ponAbe2 chr16 58950074 58950154 + **GC** | AAGAAGCCATTGAAGATGTTGAAGGACCCAGTGAAACAGCTGCTGATCCAGAGGAGCTTGCTAAGGACCAAGAGGGCGGAG|  |  |  |  |  |  |  |  |  |  |  |  |  |  |  |  |  |  |  |  |  |  |  |  |  |  |  |  |  |  |  |  |  |  |  |  |  |  |  |  |  |  |  |  |  |  |  |  |  |  |  |  |  |  |  |  |  |  |  |  |  |  |  |  | | --- | --- | --- | --- | --- | --- | --- | --- | --- | --- | --- | --- | --- | --- | --- | --- | --- | --- | --- | --- | --- | --- | --- | --- | --- | --- | --- | --- | --- | --- | --- | --- | --- | --- | --- | --- | --- | --- | --- | --- | --- | --- | --- | --- | --- | --- | --- | --- | --- | --- | --- | --- | --- | --- | --- | --- | --- | --- | --- | --- | --- | --- | --- | --- | | Rhesus rheMac2 chr20 69825299 69825379 + **GC** | AAGAAGCCATTGAAGATGTTGAAGGACCCAGTGAAACAGCTGCTGATCCAGAGGAGCTTGCTAAGGAGCAAGAGGGTGGAG|  |  |  |  |  |  |  |  |  |  |  |  |  |  |  |  |  |  |  |  |  |  |  |  |  |  |  |  |  |  |  |  |  |  |  |  |  |  |  |  |  |  |  |  |  |  |  |  |  |  |  |  |  |  |  |  | | --- | --- | --- | --- | --- | --- | --- | --- | --- | --- | --- | --- | --- | --- | --- | --- | --- | --- | --- | --- | --- | --- | --- | --- | --- | --- | --- | --- | --- | --- | --- | --- | --- | --- | --- | --- | --- | --- | --- | --- | --- | --- | --- | --- | --- | --- | --- | --- | --- | --- | --- | --- | --- | --- | --- | --- | | Baboon papHam1 scaffold12248 39346 39426 - **GC** | AAGAAGCCATTGAAGATGTTGAAGGACCCAATGAAACAGCTGCTGATCCAGAGGAGCTTGCTAAGGAGCAAGAGGGTGGAG|  |  |  |  |  |  |  |  |  |  |  |  |  |  |  |  |  |  |  |  |  |  |  |  |  |  |  |  |  |  |  |  |  |  |  |  |  |  |  |  |  |  |  |  |  |  |  |  | | --- | --- | --- | --- | --- | --- | --- | --- | --- | --- | --- | --- | --- | --- | --- | --- | --- | --- | --- | --- | --- | --- | --- | --- | --- | --- | --- | --- | --- | --- | --- | --- | --- | --- | --- | --- | --- | --- | --- | --- | --- | --- | --- | --- | --- | --- | --- | --- | | Marmoset calJac1 Contig2164 66443 66523 + **GC** | AAGAAGCCATTGAAGATGTTGAAGGACCCAGTGAAACAGCTGCTGATCCAGAGGAGCTTGCTAAGGACCAAGAGAGTGGAG|  |  |  |  |  |  |  |  |  |  |  |  |  |  |  |  |  |  |  |  |  |  |  |  |  |  |  |  |  |  |  |  |  |  |  |  |  |  |  |  | | --- | --- | --- | --- | --- | --- | --- | --- | --- | --- | --- | --- | --- | --- | --- | --- | --- | --- | --- | --- | --- | --- | --- | --- | --- | --- | --- | --- | --- | --- | --- | --- | --- | --- | --- | --- | --- | --- | --- | --- | | Tarsier tarSyr1 scaffold\_1587 56912 56992 - **GC** | AAGAAGCCATTGAAGATGTTGAAGGACCCAGTGAAACAGCTGCTGATCCAGAGGAGCTCGCCAAGGACCAAGAGAGCGGAG|  |  |  |  |  |  |  |  |  |  |  |  |  |  |  |  |  |  |  |  |  |  |  |  |  |  |  |  |  |  |  |  | | --- | --- | --- | --- | --- | --- | --- | --- | --- | --- | --- | --- | --- | --- | --- | --- | --- | --- | --- | --- | --- | --- | --- | --- | --- | --- | --- | --- | --- | --- | --- | --- | | Galago otoGar1 scaffold\_112367.1-262559 204075 204155 - **GC** | AAGAGGCTGTTGAAGATGTTGAAGGACCCAGTGAAACAGCTGCTGATCCAGAGGAGCTTGCTAAGGACCAAGAGAGTGGAG|  |  |  |  |  |  |  |  |  |  |  |  |  |  |  |  |  |  |  |  |  |  |  |  | | --- | --- | --- | --- | --- | --- | --- | --- | --- | --- | --- | --- | --- | --- | --- | --- | --- | --- | --- | --- | --- | --- | --- | --- | | Mouse mm9 chr8 111438422 111438502 + **GC** | AGGAAGCCGTTGAAGATGCCGAAGGACCCAGCGAAGCATCTGCTGACCCAGAGGAGCTGGCCAAAGACCAGGGGAGCGGCA|  |  |  |  |  |  |  |  |  |  |  |  |  |  |  |  | | --- | --- | --- | --- | --- | --- | --- | --- | --- | --- | --- | --- | --- | --- | --- | --- | | Cow bosTau4 chr18 37377548 37377628 + **GC** | AAGAAGCCATTGAAGATGTTGAAGGGCCCAGTGAAGCGGCTGCTGATCCCGAGGAGCTCGCTAAAGACCAGGAGAGTGGAG|  |  |  |  |  |  |  |  | | --- | --- | --- | --- | --- | --- | --- | --- | | Dog canFam2 chr5 81503471 81503551 - **GC** | AAGAAGCCATTGAAGATGTTGAAGGGCCCAGTGAAGCGGCTGCTGATCCAGAGGAGCTTGCTAAAGACCAGGAGAGTGGAG | | | | | | | | | | | | | | | | | | | | | | | | | | | | | | | | | | | | | | | | | | | | | | | | | | | | | | | | | | | | | | | | | | | | | | | | | | | | | | | | | | | | | | | | | | | |

**Alignment** (splice site sequences are in lowercase)  

```
Human      agAAGAAGCCATTGAAGATGTTGAAGGACCCAGTGAAACAGCTGCTGATCCAGAGGAGCTTGCTAAGGACCAAGAGGGCG
Chimp      ................................................................................
Gorilla    .....................G..........................................................
Orangutan  ................................................................................
Rhesus     .....................................................................G........T.
Baboon     ................................A....................................G........T.
Marmoset   ............................................................................A.T.
Tarsier    ............................................................C..C............A...
Galago     ......G..TG.................................................................A.T.
Mouse      ...G......G.........CC...........C...G..T.......C...........G..C..A.....G.G.A...
Cow        ...........................G.........G.G...........C........C.....A.....G...A.T.
Dog        ...........................G.........G.G..........................A.....G...A.T.

Human      GAGgt
Chimp      ....c
Gorilla    ....c
Orangutan  ....c
Rhesus     ....c
Baboon     ....c
Marmoset   ....c
Tarsier    ....c
Galago     ....c
Mouse      .CA.c
Cow        ....c
Dog        ....c
```

---

## 31. uc010vqa.1\_2\_3

**Summary**  

|  |  |  |  |  |  |  |  |  |  |  |  |  |  |  |  |  |  |  |  |  |  |  |  |  |  |
| --- | --- | --- | --- | --- | --- | --- | --- | --- | --- | --- | --- | --- | --- | --- | --- | --- | --- | --- | --- | --- | --- | --- | --- | --- | --- |
| No Exon ID Position (hg19) Dir Human donor Chimp donor Category Usage Gene symbol Protein accession mRNA accession Gene title Note|  |  |  |  |  |  |  |  |  |  |  |  |  | | --- | --- | --- | --- | --- | --- | --- | --- | --- | --- | --- | --- | --- | | 31 uc010vqa.1\_2\_3 chr17:650785 - GT AT (D9) intronization; frameshift alternative GEMIN4 B4DZ29 AK302722.1 gem (nuclear organelle) associated protein 4  | | | | | | | | | | | | | | | | | | | | | | | | | |

**Orthologs**  

|  |  |  |  |  |  |  |  |  |  |  |  |  |  |  |  |  |  |  |  |  |  |  |  |  |  |  |  |  |  |  |  |  |  |  |  |  |  |  |  |  |  |  |  |  |  |  |  |  |  |  |  |  |  |  |  |  |  |  |  |  |  |  |  |  |  |  |  |  |  |  |  |  |  |  |  |  |  |  |  |  |  |  |  |  |  |  |  |  |  |  |  |  |  |  |  |  |  |  |  |  |  |  |  |  |  |  |  |  |  |  |  |
| --- | --- | --- | --- | --- | --- | --- | --- | --- | --- | --- | --- | --- | --- | --- | --- | --- | --- | --- | --- | --- | --- | --- | --- | --- | --- | --- | --- | --- | --- | --- | --- | --- | --- | --- | --- | --- | --- | --- | --- | --- | --- | --- | --- | --- | --- | --- | --- | --- | --- | --- | --- | --- | --- | --- | --- | --- | --- | --- | --- | --- | --- | --- | --- | --- | --- | --- | --- | --- | --- | --- | --- | --- | --- | --- | --- | --- | --- | --- | --- | --- | --- | --- | --- | --- | --- | --- | --- | --- | --- | --- | --- | --- | --- | --- | --- | --- | --- | --- | --- | --- | --- | --- | --- | --- | --- | --- | --- | --- | --- | --- | --- |
| Species Assembly Chromosome Exon start Exon end Dir Donor Exon sequence|  |  |  |  |  |  |  |  |  |  |  |  |  |  |  |  |  |  |  |  |  |  |  |  |  |  |  |  |  |  |  |  |  |  |  |  |  |  |  |  |  |  |  |  |  |  |  |  |  |  |  |  |  |  |  |  |  |  |  |  |  |  |  |  |  |  |  |  |  |  |  |  |  |  |  |  |  |  |  |  |  |  |  |  |  |  |  |  |  |  |  |  |  |  |  |  |  |  |  |  |  |  |  |  | | --- | --- | --- | --- | --- | --- | --- | --- | --- | --- | --- | --- | --- | --- | --- | --- | --- | --- | --- | --- | --- | --- | --- | --- | --- | --- | --- | --- | --- | --- | --- | --- | --- | --- | --- | --- | --- | --- | --- | --- | --- | --- | --- | --- | --- | --- | --- | --- | --- | --- | --- | --- | --- | --- | --- | --- | --- | --- | --- | --- | --- | --- | --- | --- | --- | --- | --- | --- | --- | --- | --- | --- | --- | --- | --- | --- | --- | --- | --- | --- | --- | --- | --- | --- | --- | --- | --- | --- | --- | --- | --- | --- | --- | --- | --- | --- | --- | --- | --- | --- | --- | --- | --- | --- | | Human hg19 chr17 650785 651272 - **GT** | GACCCTTGAACATCTGTGAAGAAATGACTATTCTGCATGGAGGCTTCTTGCTGGCCGAGCAGCTGTTCCACCCTAAGGCACTGGCAGAATTAACAAAGTCTGACTGGGAACGTGTTGGACGGCCCATCGTGGAGGCCTTAAGGGAGATCTCCTCGGCTGCAGCACACTCCCAGCCCTTTGCCTGGAAGAAGAAAGCCCTGATCATCATCTGGGCCAAGGTTCTGCAGCCGCACCCCGTGACCCCGTCCGACACAGAGACACGGTGGCAGGAAGACCTGTTCTTCTCGGTGGGCAACATGATCCCCACCATCAACCACACCATCCTCTTCGAGCTGCTCAAATCCCTGGAAGCTTCTGGACTCTTTATCCAGCTCCTGATGGCCCTGCCCACCACCATCTGCCATGCAGAACTAGAGCGCTTTCTGGAACATGTGACCGTTGACACTTCTGCCGAAGACGTGGCCTTCTTCCTGGACGTCTGGTGGGAG|  |  |  |  |  |  |  |  |  |  |  |  |  |  |  |  |  |  |  |  |  |  |  |  |  |  |  |  |  |  |  |  |  |  |  |  |  |  |  |  |  |  |  |  |  |  |  |  |  |  |  |  |  |  |  |  |  |  |  |  |  |  |  |  |  |  |  |  |  |  |  |  |  |  |  |  |  |  |  |  |  |  |  |  |  |  |  |  |  |  |  |  |  |  |  |  | | --- | --- | --- | --- | --- | --- | --- | --- | --- | --- | --- | --- | --- | --- | --- | --- | --- | --- | --- | --- | --- | --- | --- | --- | --- | --- | --- | --- | --- | --- | --- | --- | --- | --- | --- | --- | --- | --- | --- | --- | --- | --- | --- | --- | --- | --- | --- | --- | --- | --- | --- | --- | --- | --- | --- | --- | --- | --- | --- | --- | --- | --- | --- | --- | --- | --- | --- | --- | --- | --- | --- | --- | --- | --- | --- | --- | --- | --- | --- | --- | --- | --- | --- | --- | --- | --- | --- | --- | --- | --- | --- | --- | --- | --- | --- | --- | | Chimp panTro2 chr17 669117 669604 - **AT** | GACCCTTGAACATCTGTGAAGAAATGACTATTCTGCATGGAGGCTTCTTGCTGGCTGAGCAGCTGTTCCACCCTAAGGCACTGGCAGAATTAACAAAGTCTGACTGGGAACATGTTGGACGGCCCATCGTGGAGGCCTTAAGGGAGATCTCCTCGGCTGCAGCACACTCCCAGCCCTTTGCCTGGAAGAAGAAAGCCCTGATCATCATCTGGGCCAAGGTTCTGCAGCCGCACCCCGTGACCCCGTCCGACACAGAGACACGGTGGCAGGAAGACCTGTTCTTCTCGGTGGGCAACATGATCCCCACCATCAACCACACCATCCTTTTCGAGCTGCTCAAATCCCTGGAAGCTTCTGGACTCTTTATCCAGCTCCTGATGGCCCTGCCCACCACCATCTGCCATGCAGAACTAGAGCGCTTTCTGGAACACGTGACCGTTGACACTTCTTCCGAAGACGTGGCCTTCTTCCTGGACGTCTGGTGGGAG|  |  |  |  |  |  |  |  |  |  |  |  |  |  |  |  |  |  |  |  |  |  |  |  |  |  |  |  |  |  |  |  |  |  |  |  |  |  |  |  |  |  |  |  |  |  |  |  |  |  |  |  |  |  |  |  |  |  |  |  |  |  |  |  |  |  |  |  |  |  |  |  |  |  |  |  |  |  |  |  |  |  |  |  |  |  |  |  | | --- | --- | --- | --- | --- | --- | --- | --- | --- | --- | --- | --- | --- | --- | --- | --- | --- | --- | --- | --- | --- | --- | --- | --- | --- | --- | --- | --- | --- | --- | --- | --- | --- | --- | --- | --- | --- | --- | --- | --- | --- | --- | --- | --- | --- | --- | --- | --- | --- | --- | --- | --- | --- | --- | --- | --- | --- | --- | --- | --- | --- | --- | --- | --- | --- | --- | --- | --- | --- | --- | --- | --- | --- | --- | --- | --- | --- | --- | --- | --- | --- | --- | --- | --- | --- | --- | --- | --- | | Gorilla gorGor1 Supercontig\_0428564 107 594 - **AT** | GACCCTTGAACATCTGTGAAGAAATGACTATTCTGCATGGAGGCTTCTTGCTGGCTGAGCAGCTGTTCCACCCTAAGGCACTGGCAGAATTAACAAAGTCTGACTGGGAACATGTTGGACGGCCCATCGTGGAGGCCTTAAGGGAGATCTCCTCGGCTGCAGCACACTCCCAGCCCTTTGCCTGGAAGAAGAAAGCCCTGATCATCATCTGGGCCAAGGTTCTGCAGCCGCACCCTGTGACCCCGTCCGACACAGAGACACGGTGGCAGGAAGACCTGTTCTTCTCGGTGGGCAACATGATCCCCACCATCAACCACACTGTCCTCTTCGAGCTGCTCAAATCCCTGGAAGCTTCTGGACTCTTTATCCAGCTCCTGATGGCCCTGCCCACCACCATCTGCCATGCAGAACTGGAGCGCTTTCTGGAACACGTGACCGTTGACACTTCTTCTGAAGACGTGGCCTTCTTCCTGGACGTCTGGTGGGAG|  |  |  |  |  |  |  |  |  |  |  |  |  |  |  |  |  |  |  |  |  |  |  |  |  |  |  |  |  |  |  |  |  |  |  |  |  |  |  |  |  |  |  |  |  |  |  |  |  |  |  |  |  |  |  |  |  |  |  |  |  |  |  |  |  |  |  |  |  |  |  |  |  |  |  |  |  |  |  |  | | --- | --- | --- | --- | --- | --- | --- | --- | --- | --- | --- | --- | --- | --- | --- | --- | --- | --- | --- | --- | --- | --- | --- | --- | --- | --- | --- | --- | --- | --- | --- | --- | --- | --- | --- | --- | --- | --- | --- | --- | --- | --- | --- | --- | --- | --- | --- | --- | --- | --- | --- | --- | --- | --- | --- | --- | --- | --- | --- | --- | --- | --- | --- | --- | --- | --- | --- | --- | --- | --- | --- | --- | --- | --- | --- | --- | --- | --- | --- | --- | | Orangutan ponAbe2 chr17 571451 571938 - **AT** | GACCCTTGAACATCTGTGAAGAAATGACTATTCTGCATGGAGGCTTCTTGCTGGCCGAGCAGCTGTTCCGCCCTAAGGCCCTGGCAGAATTAACAAAGTCTGACTGGGAACATGTTGGACGGCCCATCGTGGAGGCCTTAAGGGAGATCTCCTCGGCTGCAGCACACTCCCAGCCCTTTGCCTGGAAGAAGAAAGCCCTGATCATCATCTGGGCCAAGGTTCTGCAGCCGCACCCCGTGACCCCGTCTGACACGGAGACACGGTGGCAGGAAGACCTGTTCTTCTCGGTGGGCAACATGATCCCCACCATCAACCATACCGTCCTCTTTGAGCTGCTCAAATCCCTGGAAGCTTCTGCACTCTTTATCCAGCTCCTGATGGCCCTGCCCACTACCATCTGCCATGCAGAACTAGAGCGCTTTCTGGAACACGTGACCGTTGACACTTCTTCTGAAGATGTGGCCTTCTTCTTGGACGTCTGGTGGGAG|  |  |  |  |  |  |  |  |  |  |  |  |  |  |  |  |  |  |  |  |  |  |  |  |  |  |  |  |  |  |  |  |  |  |  |  |  |  |  |  |  |  |  |  |  |  |  |  |  |  |  |  |  |  |  |  |  |  |  |  |  |  |  |  |  |  |  |  |  |  |  |  | | --- | --- | --- | --- | --- | --- | --- | --- | --- | --- | --- | --- | --- | --- | --- | --- | --- | --- | --- | --- | --- | --- | --- | --- | --- | --- | --- | --- | --- | --- | --- | --- | --- | --- | --- | --- | --- | --- | --- | --- | --- | --- | --- | --- | --- | --- | --- | --- | --- | --- | --- | --- | --- | --- | --- | --- | --- | --- | --- | --- | --- | --- | --- | --- | --- | --- | --- | --- | --- | --- | --- | --- | | Rhesus rheMac2 chr16 539100 539587 - **AT** | GGCCCTTGAACATCTGTGAAGAAATGACTATTCTGCATGGAGGCTTCTTGCTGGCCGAGCAGCTGTTCCGCCCCAAGGCACTGGCAGAATTGACAAAGTCTGACTGGGAACGTGTTGGACGCCCCATCGTGGAGGCCTTAAGGGAGATCTCCTCGGCCGCAGCACACTCCCAGCCCTTTGCCTGGAAGAAGAAAGCCCTGATTATCATCTGGGCCAAGGTTCTGCAGCCGCACCCCGTGACCCCCTCCGACATGGAGACACGGTGGCAGGAAGACCTGTTCTTCTCGGTGGGCAACATGATCCCCACCATCAACCACACTGTCCTTTTTGAGCTGCTCAAATCCCTGGAAGCTTCTGGACTCTTTATCCAGCTCCTGATGGCCCTGCCCACCACCATCCGCCATGCAGAACTAGAGCGCTTTCTGGAGCACGTGACCGTTGACACTTCTTCTGAAGACGTGGCCTTCTTCCTGGACGTCTGGTGGGAG|  |  |  |  |  |  |  |  |  |  |  |  |  |  |  |  |  |  |  |  |  |  |  |  |  |  |  |  |  |  |  |  |  |  |  |  |  |  |  |  |  |  |  |  |  |  |  |  |  |  |  |  |  |  |  |  |  |  |  |  |  |  |  |  | | --- | --- | --- | --- | --- | --- | --- | --- | --- | --- | --- | --- | --- | --- | --- | --- | --- | --- | --- | --- | --- | --- | --- | --- | --- | --- | --- | --- | --- | --- | --- | --- | --- | --- | --- | --- | --- | --- | --- | --- | --- | --- | --- | --- | --- | --- | --- | --- | --- | --- | --- | --- | --- | --- | --- | --- | --- | --- | --- | --- | --- | --- | --- | --- | | Baboon papHam1 scaffold16075 30178 30665 + **AT** | GGCCCTTGAACATCTGTGAAGAAATGACTATTCTGCATGGAGGCTTCTTGCTGGCCGAGCAGCTGTTCCGCCCCAAGGCACTGGCAGAATTGACAAAGTCTGACTGGGAACATGTTGGACGCCCCATCGTGGAGGCCTTAAGGGAGATCTCCTCGGCCGCGGCACACTCCCAGCCCTTTGCCTGGAAGAAGAAAGCCCTGATTATCATCTGGGCCAAGGTTCTGCAGCCGCACCCCGTGACCCCCTCCGACACGGAGACACGGTGGCAGGAAGACCTGTTCTTCTCGGTGGGCAACATGATCCCCACCATCAACCACACTGTCCTCTTTGAGCTGCTCAAATCCCTGGAAGCTTCTGGACTCTTTATCCAACTCCTGATGGCCCTGCCCACCACCATCCGCCATGCAGAACTAGAGCGCTTTCTGGAGCACGTGACTGTTGACACTTCTTCTGAAGATGTGGCCTTCTTCCTGGACGTCTGGTGGGAG|  |  |  |  |  |  |  |  |  |  |  |  |  |  |  |  |  |  |  |  |  |  |  |  |  |  |  |  |  |  |  |  |  |  |  |  |  |  |  |  |  |  |  |  |  |  |  |  |  |  |  |  |  |  |  |  | | --- | --- | --- | --- | --- | --- | --- | --- | --- | --- | --- | --- | --- | --- | --- | --- | --- | --- | --- | --- | --- | --- | --- | --- | --- | --- | --- | --- | --- | --- | --- | --- | --- | --- | --- | --- | --- | --- | --- | --- | --- | --- | --- | --- | --- | --- | --- | --- | --- | --- | --- | --- | --- | --- | --- | --- | | Marmoset calJac1 Contig1580 411261 411748 + **AT** | GACCCTTGAACATCTGTGAGGAAATGACTATTCTGCACGGAGGCTTCCTGTTGGCTGAGCAGCTGTTCCACCCCAGAGCTCTGACAGAACTGACCAAGTCTGACTGGGAGCACGTTGGGCGGCCCATTGTGGAGGCCTTGCAGGAGATCTCCTCCGCAGCAGCGCATTCCCAGCCCTTTGCCTGGAAGAAGAAAGCTCTGATCATCATCTGGGCCAAGGTTCTGCAGCCTCAGCCTGTGACCTCTTCTGACACGGACACTCGGTGGCAGGAAGATGTGTTCTTCTCAGTGGGCAATATGATCCCCACCCTCAATCACACCGTCCTGTTTGAGCTGCTCAAGTCCCTAGAAGCTTCTGGACTCTTTATCGAGCTCCTGATGGCCCTGCCTGCCATTGTCTGCTGTGCAGAACTCGAGCGCTTTTTGGAGCACGTGAGCATTGACACGTCCTCTGAGGACGTGGCCTTCTTCCTGGATGTCTGGTGGGAA|  |  |  |  |  |  |  |  |  |  |  |  |  |  |  |  |  |  |  |  |  |  |  |  |  |  |  |  |  |  |  |  |  |  |  |  |  |  |  |  |  |  |  |  |  |  |  |  | | --- | --- | --- | --- | --- | --- | --- | --- | --- | --- | --- | --- | --- | --- | --- | --- | --- | --- | --- | --- | --- | --- | --- | --- | --- | --- | --- | --- | --- | --- | --- | --- | --- | --- | --- | --- | --- | --- | --- | --- | --- | --- | --- | --- | --- | --- | --- | --- | | Tarsier tarSyr1 scaffold\_10072 26491 26978 + **AT** | GGGCCTTGAACATCTGTGAAGAAATAACTATCCTGCATGGGGGCTTCTTGCTGGCCGAGCAGCTGTTCCACCCCAAAGTATTGGCAGACTTGGCAAAGTCTGACTGGGAACATGTTGGGAGGCCCATTGTGGAGGCCTTGAGGGAGATCTCCTCAGCCACTGCACATTCCCAGCCCTTTGCCTGGAAGAAGAAAGCTCTGATCATCATCTGGGCCAAGGTTCTTCAGCCCTGCCCTGTTAGCCCTTCTGACACTGAAACTCGGTGGCAGGAAGATGTGTTCTTCTCAGTGGGCAACATGATCCCTACTATCAATCACACAGTTCTTTTTGAGCTGCTCAAATCCCTGGAAGCTTCTGGACTCTTTATCCAGCTCCTGATGGCCCTGCCTACCACGGTCTGCCGCACAGAACTAGAGCGCTTTTTGGAGCACATGACTACTGACACTTCATCCAAAGATGTGGCCTTCTTCCTCGATGTCTGGTGGGAG|  |  |  |  |  |  |  |  |  |  |  |  |  |  |  |  |  |  |  |  |  |  |  |  |  |  |  |  |  |  |  |  |  |  |  |  |  |  |  |  | | --- | --- | --- | --- | --- | --- | --- | --- | --- | --- | --- | --- | --- | --- | --- | --- | --- | --- | --- | --- | --- | --- | --- | --- | --- | --- | --- | --- | --- | --- | --- | --- | --- | --- | --- | --- | --- | --- | --- | --- | | Lemur micMur1 scaffold\_1499 49020 49507 - **AT** | GACCCTTGAACATCTGTGAAGAAATGACTATTCTGCATGGGGGCTTCTTGCTGGCCGAGCAGCTGTTCCGCCCCAAAGCACTGGCAGAATTGACAAAGTCTGACTGGGAGCACGTTGGGCGGCCCATCGTGGAGGCCTTGAGGGAGATCTCCTCGGCCACAGCGCATGCACAGTCCTTTGCCTGGAAGAAGAAAGCTCTGATCATCATCTGGGCCAAGGTTCTTCAACCCTGCCCCGTTACCCCTTCTGACACTGAAGCTCGGTGGCAGGAAGATGTGTTCTTCTCAGTAGGCAACATGATCCCTACAATCAACCACACAGTCCTTTTTGAGCTGCTCAAGTCCCTGGAAGCTTCTGGACTCTTTATTCAGCTCCTGATGGCTCTGCCCACCACCATCTGCCGTGCAGAACTAGAGCGCTTTTTGGAGCACATGACTATTGACACTTCTTCCAAGGACGTGGCCTTCTTCCTGGATGTCTGGTGGGAA|  |  |  |  |  |  |  |  |  |  |  |  |  |  |  |  |  |  |  |  |  |  |  |  |  |  |  |  |  |  |  |  | | --- | --- | --- | --- | --- | --- | --- | --- | --- | --- | --- | --- | --- | --- | --- | --- | --- | --- | --- | --- | --- | --- | --- | --- | --- | --- | --- | --- | --- | --- | --- | --- | | Galago otoGar1 scaffold\_16583.1-122997 45211 45698 + **AT** | GACCCTTGAACATCTGTGAAGAAATGACTATTCTGCATGGGGGCTTCTTGCTGGCTGAGCAGCTGTTCCACCCCAGAGCATTGGCAGAATTGACAAAGTCTGACTGGGAACATGTTGGCCGGCCCATTGTGGAAGCCTTACGGGAGATCTCCTCAGCCACAGCACACTCCCAATCCTTTGCCTGGAAGAAGAAAGCTCTCATCATTATCTGGGCCAAAGTTCTTCAGCCCTACCCTGTTACCCCTTCTGACACTGAAACTCGGTGGCAGGAAGATGTATTTTTCTCGGTAGGTAACATGATCCCTACCATCAATCACACAGTCCTTTTTGAGCTGCTCAAGTCCCTGGAAGCTTCTGGACTTTTTATTCAGCTCCTCATGGCTCTGCCCACCACCATCTGCCGCACAGAACTTGAGCGCTTTTTGGAGCACATGACTATTGACACTTCTTCTAGGGATGTGGCCTTCTTCTTGGATGTCTGGTGGGAA|  |  |  |  |  |  |  |  |  |  |  |  |  |  |  |  |  |  |  |  |  |  |  |  | | --- | --- | --- | --- | --- | --- | --- | --- | --- | --- | --- | --- | --- | --- | --- | --- | --- | --- | --- | --- | --- | --- | --- | --- | | Mouse mm9 chr11 76026938 76027425 - **AT** | GACCCTTGAATATCTGTGAGGAGATGACTATTCTGCATGGGGGCTTCTTGATGGCTGAGCAGCTGTTCCACCCCAAAGCCCTGGTACAGTTGACCAAGTCTGATTGGGAACACGTGGGACAGCCCATTGTGGAGGCCTTGAAGGAGATCTCCTCAGCCACAGCACATTCCCAGCCCTTTGCCTGGAAGAAGAAAGCACTGATCATCATCTGGACCAAGGTTCTTCAGCCCTCCCCTGTCAGCCCTTCTGACACTGATACTCGGTGGCAGGAAGATGTATTCTTCTCCGTGGGCAACATGATCCCTACCATCAACCACACAGTCCTTTTTGAGCTGCTCAAGTCTCTGGAAGCTTCTGGACTCTTCATTCAGCTGCTAATGGCCTTGCCTACCACCATTGGCCGCTCAGAACTACAGAGCTTCCTGGAGCACATGACTGTTGACACATCTTCCAAGGATGTGGCCTTCTTCTTAGATGTCTGGTGGGAA|  |  |  |  |  |  |  |  |  |  |  |  |  |  |  |  | | --- | --- | --- | --- | --- | --- | --- | --- | --- | --- | --- | --- | --- | --- | --- | --- | | Cow bosTau4 chr19 22114444 22114931 + **AT** | GACCTTTGAATATCTGTGAGGAAATGACTATCTTGCACGGGGGCTTCTTGCTGGCTGAGCAGCTGTTCCGCCCCAAAGCGCTGGCCGAACTGACCAAGTCTGACTGGGTGCACGTTGGGCGGCCCATTGTGGAGGCTCTGAGGGAGATCTCCTCCACCACAGCCTGCTCCCAGCCCTTCGCCTGGAAGAAGAAAGCGCTGATCATCATCTGGGCCAAGGTGCTACAGCCCTACCCCATCACCCCTTCCGACATGGAAACCCGCTGGCAGGAAGATGTGTTCTTCTCTGTGGGCAACATGATCCCCACCATCAATCACACAGTCCTCTTTGAGCTGCTCAAGTCGCTGGAGGCCTCTGGACTCTTTATCCAGCTCCTGATGGCCCTGCCCACCACTGTCTGCCGTGCGGAACTAGAGCACTTTTTGGAGCACATGACCATCGACACCTCTTCCAAGGACGTGGCCTTCTTCCTCGACGTCTGGTGGGAA|  |  |  |  |  |  |  |  | | --- | --- | --- | --- | --- | --- | --- | --- | | Dog canFam2 chr9 48367803 48368290 + **AT** | GACCTTTGAATATCTGTGAAGAAATGACTATTCTGCATGGGGGCTTTTTGCTTGCCGAGCAGCTCTTCCACCCCAAAGCACTGACAGAACTGACAAAGTCTGACTGGGAACATGTTGGGCAGCCCATTGTGGAGGCCCTAAGGGAGATCTCCTCCGCCACAACATGCTCCCAGCCCTTTGCCTGGAAGAAGAAAGCTCTGATCATCATCTGGGCCAAGGTTCTTCAGCCCTACCCTGTCACCCCTTCAGACACTGACACTCGGTGGCAGGAAGATGTGTTCTTCTCGGTAGGCAACATGATCCCTACCATCAATCACACGGTCCTTTTTGAGCTGCTCAAGTCTCTGGAAGCTTCTGGACTCTTTATCCAGCTTCTGATGGCCCTGCCCACCACCATCTGCCATGCAGAACTAGAGCGCTTTTTGGAGCACATGTCTGTTGACACTTCTTCAGAGGATGTGGCCTTTTTCCTCAGTGTCTGGTGGGAA | | | | | | | | | | | | | | | | | | | | | | | | | | | | | | | | | | | | | | | | | | | | | | | | | | | | | | | | | | | | | | | | | | | | | | | | | | | | | | | | | | | | | | | | | | | | | | | | | | |

**Alignment** (splice site sequences are in lowercase)  

```
Human      agGACCCTTGAACATCTGTGAAGAAATGACTATTCTGCATGGAGGCTTCTTGCTGGCCGAGCAGCTGTTCCACCCTAAGG
Chimp      .........................................................T......................
Gorilla    .........................................................T......................
Orangutan  .......................................................................G........
Rhesus     ...G...................................................................G...C....
Baboon     ...G...................................................................G...C....
Marmoset   .....................G.................C.........C..T....T.................C.GA.
Tarsier    ...GG......................A.....C........G................................C..A.
Lemur      ..........................................G............................G...C..A.
Galago     ..........................................G..............T.................C.GA.
Mouse      ............T........G..G.................G.........A....T.................C..A.
Cow        ......T.....T........G...........CT....C..G..............T.............G...C..A.
Dog        ......T.....T.............................G.....T.....T...........C........C..A.

Human      CACTGGCAGAATTAACAAAGTCTGACTGGGAACGTGTTGGACGGCCCATCGTGGAGGCCTTAAGGGAGATCTCCTCGGCT
Chimp      .................................A..............................................
Gorilla    .................................A..............................................
Orangutan  .C...............................A..............................................
Rhesus     .............G.............................C...................................C
Baboon     .............G...................A.........C...................................C
Marmoset   .T...A.....C.G..C..............G.AC.....G........T...........GCA............C..A
Tarsier    T.T.......C..GG..................A......GA.......T...........G..............A..C
Lemur      .............G.................G.AC.....G....................G.................C
Galago     ..T..........G...................A......C........T.....A......C.............A..C
Mouse      .C....T.C.G..G..C........T.......AC..G....A......T...........G.A............A..C
Cow        .G.....C...C.G..C.............TG.AC.....G........T........TC.G..............CA.C
Dog        .....A.....C.G...................A......G.A......T.........C................C..C

Human      GCAGCACACTCCCAGCCCTTTGCCTGGAAGAAGAAAGCCCTGATCATCATCTGGGCCAAGGTTCTGCAGCCGCACCCCGT
Chimp      ................................................................................
Gorilla    .............................................................................T..
Orangutan  ................................................................................
Rhesus     ............................................T...................................
Baboon     ..G.........................................T...................................
Marmoset   .....G..T.............................T................................T..G..T..
Tarsier    A.T.....T.............................T..........................T.....CTG...T..
Lemur      A....G..TG.A...T......................T..........................T..A..CTG......
Galago     A.............AT......................T..C.....T...........A.....T.....CT....T..
Mouse      A.......T.............................A...............A..........T.....CTC...T..
Cow        A....CTG............C.................G.......................G..A.....CT.....A.
Dog        A..A..TG..............................T..........................T.....CT....T..

Human      GACCCCGTCCGACACAGAGACACGGTGGCAGGAAGACCTGTTCTTCTCGGTGGGCAACATGATCCCCACCATCAACCACA
Chimp      ................................................................................
Gorilla    ................................................................................
Orangutan  .........T.....G..............................................................T.
Rhesus     ......C.......TG................................................................
Baboon     ......C........G................................................................
Marmoset   ....T.T..T.....G..C..T..............TG..........A........T............C....T....
Tarsier    T.G...T..T.....T..A..T..............TG..........A.................T..T.....T....
Lemur      T.....T..T.....T..AG.T..............TG..........A..A..............T..A..........
Galago     T.....T..T.....T..A..T..............TG.A..T........A..T...........T........T....
Mouse      C.G...T..T.....T..T..T..............TG.A........C.................T.............
Cow        C.....T.......TG..A..C..C...........TG..........T..........................T....
Dog        C.....T..A.....T..C..T..............TG.............A..............T........T....

Human      CCATCCTCTTCGAGCTGCTCAAATCCCTGGAAGCTTCTGGACTCTTTATCCAGCTCCTGATGGCCCTGCCCACCACCATC
Chimp      .......T........................................................................
Gorilla    .TG.............................................................................
Orangutan  ..G.......T............................C.................................T......
Rhesus     .TG....T..T.....................................................................
Baboon     .TG.......T.........................................A...........................
Marmoset   ..G....G..T...........G.....A.....................G...................TG...TTG..
Tarsier    .AG.T..T..T...........................................................T.....GG..
Lemur      .AG....T..T...........G..........................T..............T...............
Galago     .AG....T..T...........G....................T.....T........C.....T...............
Mouse      .AG....T..T...........G..T....................C..T.....G..A......T....T........T
Cow        .AG.......T...........G..G.....G..C.........................................TG..
Dog        .GG....T..T...........G..T.............................T........................

Human      TGCCATGCAGAACTAGAGCGCTTTCTGGAACATGTGACCGTTGACACTTCTGCCGAAGACGTGGCCTTCTTCCTGGACGT
Chimp      ................................C..................T............................
Gorilla    ..............G.................C..................T.T..........................
Orangutan  ................................C..................T.T.....T............T.......
Rhesus     C............................G..C..................T.T..........................
Baboon     C............................G..C.....T............T.T.....T....................
Marmoset   ...TG.........C.........T....G..C....G.A.......G..CT.T..G....................T..
Tarsier    ....GCA.................T....G..CA....TAC.........AT..A....T..............C..T..
Lemur      ....G...................T....G..CA....TA...........T..A.G....................T..
Galago     ....GCA.......T.........T....G..CA....TA...........T.TAGG..T............T....T..
Mouse      G...GCT........C..A....C.....G..CA....T........A...T..A.G..T............T.A..T..
Cow        ....G...G..........A....T....G..CA.....A.C.....C...T..A.G.................C.....
Dog        ........................T....G..CA..T.T............T.A..G..T........T.....CAGT..

Human      CTGGTGGGAGgt
Chimp      ..........a.
Gorilla    ..........a.
Orangutan  ..........a.
Rhesus     ..........a.
Baboon     ..........a.
Marmoset   .........Aa.
Tarsier    ..........a.
Lemur      .........Aa.
Galago     .........Aa.
Mouse      .........Aa.
Cow        .........Aa.
Dog        .........Aa.
```

---

## 32. uc002htp.2\_2\_5

**Summary**  

|  |  |  |  |  |  |  |  |  |  |  |  |  |  |  |  |  |  |  |  |  |  |  |  |  |  |
| --- | --- | --- | --- | --- | --- | --- | --- | --- | --- | --- | --- | --- | --- | --- | --- | --- | --- | --- | --- | --- | --- | --- | --- | --- | --- |
| No Exon ID Position (hg19) Dir Human donor Chimp donor Category Usage Gene symbol Protein accession mRNA accession Gene title Note|  |  |  |  |  |  |  |  |  |  |  |  |  | | --- | --- | --- | --- | --- | --- | --- | --- | --- | --- | --- | --- | --- | | 32 uc002htp.2\_2\_5 chr17:38172107 + GT GC (D1) shift; increase; inframe alternative CSF3 NP\_000750.1 NM\_000759.3 colony stimulating factor 3 (granulocyte)  | | | | | | | | | | | | | | | | | | | | | | | | | |

**Orthologs**  

|  |  |  |  |  |  |  |  |  |  |  |  |  |  |  |  |  |  |  |  |  |  |  |  |  |  |  |  |  |  |  |  |  |  |  |  |  |  |  |  |  |  |  |  |  |  |  |  |  |  |  |  |  |  |  |  |  |  |  |  |  |  |  |  |  |  |  |  |  |  |  |  |  |  |  |  |  |  |  |  |  |  |  |  |  |  |  |  |  |  |  |  |  |  |  |  |  |  |  |  |  |  |  |  |
| --- | --- | --- | --- | --- | --- | --- | --- | --- | --- | --- | --- | --- | --- | --- | --- | --- | --- | --- | --- | --- | --- | --- | --- | --- | --- | --- | --- | --- | --- | --- | --- | --- | --- | --- | --- | --- | --- | --- | --- | --- | --- | --- | --- | --- | --- | --- | --- | --- | --- | --- | --- | --- | --- | --- | --- | --- | --- | --- | --- | --- | --- | --- | --- | --- | --- | --- | --- | --- | --- | --- | --- | --- | --- | --- | --- | --- | --- | --- | --- | --- | --- | --- | --- | --- | --- | --- | --- | --- | --- | --- | --- | --- | --- | --- | --- | --- | --- | --- | --- | --- | --- | --- | --- |
| Species Assembly Chromosome Exon start Exon end Dir Donor Exon sequence|  |  |  |  |  |  |  |  |  |  |  |  |  |  |  |  |  |  |  |  |  |  |  |  |  |  |  |  |  |  |  |  |  |  |  |  |  |  |  |  |  |  |  |  |  |  |  |  |  |  |  |  |  |  |  |  |  |  |  |  |  |  |  |  |  |  |  |  |  |  |  |  |  |  |  |  |  |  |  |  |  |  |  |  |  |  |  |  |  |  |  |  |  |  |  |  | | --- | --- | --- | --- | --- | --- | --- | --- | --- | --- | --- | --- | --- | --- | --- | --- | --- | --- | --- | --- | --- | --- | --- | --- | --- | --- | --- | --- | --- | --- | --- | --- | --- | --- | --- | --- | --- | --- | --- | --- | --- | --- | --- | --- | --- | --- | --- | --- | --- | --- | --- | --- | --- | --- | --- | --- | --- | --- | --- | --- | --- | --- | --- | --- | --- | --- | --- | --- | --- | --- | --- | --- | --- | --- | --- | --- | --- | --- | --- | --- | --- | --- | --- | --- | --- | --- | --- | --- | --- | --- | --- | --- | --- | --- | --- | --- | | Human hg19 chr17 38171944 38172107 + **GT** | CCCTGCAGCTGCTGCTGTGGCACAGTGCACTCTGGACAGTGCAGGAAGCCACCCCCCTGGGCCCTGCCAGCTCCCTGCCCCAGAGCTTCCTGCTCAAGTGCTTAGAGCAAGTGAGGAAGATCCAGGGCGATGGCGCAGCGCTCCAGGAGAAGCTGGTGAGTGAG|  |  |  |  |  |  |  |  |  |  |  |  |  |  |  |  |  |  |  |  |  |  |  |  |  |  |  |  |  |  |  |  |  |  |  |  |  |  |  |  |  |  |  |  |  |  |  |  |  |  |  |  |  |  |  |  |  |  |  |  |  |  |  |  |  |  |  |  |  |  |  |  |  |  |  |  |  |  |  |  |  |  |  |  |  |  |  |  | | --- | --- | --- | --- | --- | --- | --- | --- | --- | --- | --- | --- | --- | --- | --- | --- | --- | --- | --- | --- | --- | --- | --- | --- | --- | --- | --- | --- | --- | --- | --- | --- | --- | --- | --- | --- | --- | --- | --- | --- | --- | --- | --- | --- | --- | --- | --- | --- | --- | --- | --- | --- | --- | --- | --- | --- | --- | --- | --- | --- | --- | --- | --- | --- | --- | --- | --- | --- | --- | --- | --- | --- | --- | --- | --- | --- | --- | --- | --- | --- | --- | --- | --- | --- | --- | --- | --- | --- | | Chimp panTro2 chr17 17523558 17523721 - **GC** | CCCTGCAGCTGCTGCTGTGGCACAGTGCACTCTGGACAGTGCAGGAAGCCACCCCCCTGGGCCCTGCCAGCTCCCTGCCCCAGAGCTTCCTGCTCAAGTGCTTAGAGCAAGTGAGGAAGATCCAGGGCGATGGCGCGGCGCTCCAGGAGAAGCTGGTGAGTGAG|  |  |  |  |  |  |  |  |  |  |  |  |  |  |  |  |  |  |  |  |  |  |  |  |  |  |  |  |  |  |  |  |  |  |  |  |  |  |  |  |  |  |  |  |  |  |  |  |  |  |  |  |  |  |  |  |  |  |  |  |  |  |  |  |  |  |  |  |  |  |  |  |  |  |  |  |  |  |  |  | | --- | --- | --- | --- | --- | --- | --- | --- | --- | --- | --- | --- | --- | --- | --- | --- | --- | --- | --- | --- | --- | --- | --- | --- | --- | --- | --- | --- | --- | --- | --- | --- | --- | --- | --- | --- | --- | --- | --- | --- | --- | --- | --- | --- | --- | --- | --- | --- | --- | --- | --- | --- | --- | --- | --- | --- | --- | --- | --- | --- | --- | --- | --- | --- | --- | --- | --- | --- | --- | --- | --- | --- | --- | --- | --- | --- | --- | --- | --- | --- | | Gorilla gorGor1 Supercontig\_0006833 10703 10866 - **GC** | CCCTGCAGCTGCTGCTGTGGCACAGTGCACTCTGGACAGTGCAGGAAGCCACCCCCCTGGGCCCTGCCAGCTCCCTGCCCCAGAGCTTCCTGCTCAAGTGCTTAGAGCAAGTGAGGAAGATCCAGGGCGATGGCGCGGCACTCCAGGAGAAGCTGGTGAGTGAG|  |  |  |  |  |  |  |  |  |  |  |  |  |  |  |  |  |  |  |  |  |  |  |  |  |  |  |  |  |  |  |  |  |  |  |  |  |  |  |  |  |  |  |  |  |  |  |  |  |  |  |  |  |  |  |  |  |  |  |  |  |  |  |  |  |  |  |  |  |  |  |  | | --- | --- | --- | --- | --- | --- | --- | --- | --- | --- | --- | --- | --- | --- | --- | --- | --- | --- | --- | --- | --- | --- | --- | --- | --- | --- | --- | --- | --- | --- | --- | --- | --- | --- | --- | --- | --- | --- | --- | --- | --- | --- | --- | --- | --- | --- | --- | --- | --- | --- | --- | --- | --- | --- | --- | --- | --- | --- | --- | --- | --- | --- | --- | --- | --- | --- | --- | --- | --- | --- | --- | --- | | Orangutan ponAbe2 chr17 49412086 49412249 - **GC** | CCCTGCAGCTGCTGCTGTGGCACAGCGCACTCTGGACAGTGCAGGAAGCCACCCCCCTGGGCCCTGCCAGCTCCCTGCCCCAGAGCTTCCTGCTCAAGTGCTTAGAGCAAGTGAGGAAGATCCAGGGCGATGGCGCGGCACTCCAGGAGAAGCTGGTGAGTGAG|  |  |  |  |  |  |  |  |  |  |  |  |  |  |  |  |  |  |  |  |  |  |  |  |  |  |  |  |  |  |  |  |  |  |  |  |  |  |  |  |  |  |  |  |  |  |  |  |  |  |  |  |  |  |  |  |  |  |  |  |  |  |  |  | | --- | --- | --- | --- | --- | --- | --- | --- | --- | --- | --- | --- | --- | --- | --- | --- | --- | --- | --- | --- | --- | --- | --- | --- | --- | --- | --- | --- | --- | --- | --- | --- | --- | --- | --- | --- | --- | --- | --- | --- | --- | --- | --- | --- | --- | --- | --- | --- | --- | --- | --- | --- | --- | --- | --- | --- | --- | --- | --- | --- | --- | --- | --- | --- | | Rhesus rheMac2 chr16 50114753 50114916 + **GC** | CCCTGCAGCTGCTGCTGTGGCACAGCGCACTCTGGACAGTGCAGGAAGCCACCCCCCTGGGCCCTGCCAGCTCCCTGCCCCAGAGCTTCCTGCTCAAGTGCTTAGAGCAAGTAAGGAAGATCCAGGGCGACGGTGCCGCGCTGCAGGAGAAGTTGGTGAGTGAG|  |  |  |  |  |  |  |  |  |  |  |  |  |  |  |  |  |  |  |  |  |  |  |  |  |  |  |  |  |  |  |  |  |  |  |  |  |  |  |  |  |  |  |  |  |  |  |  |  |  |  |  |  |  |  |  | | --- | --- | --- | --- | --- | --- | --- | --- | --- | --- | --- | --- | --- | --- | --- | --- | --- | --- | --- | --- | --- | --- | --- | --- | --- | --- | --- | --- | --- | --- | --- | --- | --- | --- | --- | --- | --- | --- | --- | --- | --- | --- | --- | --- | --- | --- | --- | --- | --- | --- | --- | --- | --- | --- | --- | --- | | Baboon papHam1 scaffold1311 36057 36220 - **GC** | CCCTGCAGCTGCTGTTGTGGCACAGCGCACTCTGGACAGTGCAGGAAGCCACCCCCCTGGGCCCTGCCAGCTCCCTGCCCCAGAGCTTCCTGCTCAAGTGCTTAGAGCAAGTAAGGAAGATCCAGGGCGACGGTGCCGCGCTGCAGGAGAAGCTGGTGAGTGAG|  |  |  |  |  |  |  |  |  |  |  |  |  |  |  |  |  |  |  |  |  |  |  |  |  |  |  |  |  |  |  |  |  |  |  |  |  |  |  |  |  |  |  |  |  |  |  |  | | --- | --- | --- | --- | --- | --- | --- | --- | --- | --- | --- | --- | --- | --- | --- | --- | --- | --- | --- | --- | --- | --- | --- | --- | --- | --- | --- | --- | --- | --- | --- | --- | --- | --- | --- | --- | --- | --- | --- | --- | --- | --- | --- | --- | --- | --- | --- | --- | | Marmoset calJac1 Contig9599 52140 52303 - **GC** | CCCTGCAGCTGCTGCTATGGCACAGTGCACTCTGGACGGTGCAGGAAGCCGCCCCCCTGGGCCCTGCCAGCTCCCTGCCCCAGAGTTTCCTGCTCAAGTGCTTAGAGCAAGTGAGGAAGGTCCAGGGCGATGGCACGGAGTTGCAGGAGAAGCTGGTGAGTGAG|  |  |  |  |  |  |  |  |  |  |  |  |  |  |  |  |  |  |  |  |  |  |  |  |  |  |  |  |  |  |  |  |  |  |  |  |  |  |  |  | | --- | --- | --- | --- | --- | --- | --- | --- | --- | --- | --- | --- | --- | --- | --- | --- | --- | --- | --- | --- | --- | --- | --- | --- | --- | --- | --- | --- | --- | --- | --- | --- | --- | --- | --- | --- | --- | --- | --- | --- | | Lemur micMur1 scaffold\_3270 80075 80238 + **AG** | CCCTGCAGCTGCTGCTCTGGCACAGCACACTCTGGCCAGCGCAAGAAGCCAGCCCCCTGGACCCTGCCAGCTCCCTGCCCCAGAGCTTCCTGCTCAAGTGCTTGGAGCAAGTGAGAAAGATCCAGAGCGACGGCGCAGCCCTGCAGGAGAAGCTGGTGAGTGGG|  |  |  |  |  |  |  |  |  |  |  |  |  |  |  |  |  |  |  |  |  |  |  |  |  |  |  |  |  |  |  |  | | --- | --- | --- | --- | --- | --- | --- | --- | --- | --- | --- | --- | --- | --- | --- | --- | --- | --- | --- | --- | --- | --- | --- | --- | --- | --- | --- | --- | --- | --- | --- | --- | | Galago otoGar1 scaffold\_20611.1-1041 376 539 - **GG** | CCCTACAGCTGCTGCTCTGGCACAGTGCACTCTGGACGGTGCAGGAAGCCAGTCCCCTGGGCCCTGCCAGTTCCCTGCCCCAGGGCTTTCTGCTCAAGTGCTTAGAGCAAGTGAGGAAGATCCAGAATGAAGGTGCGGCCCTGCAGGAGAATCTGGTGAGTGAG|  |  |  |  |  |  |  |  |  |  |  |  |  |  |  |  |  |  |  |  |  |  |  |  | | --- | --- | --- | --- | --- | --- | --- | --- | --- | --- | --- | --- | --- | --- | --- | --- | --- | --- | --- | --- | --- | --- | --- | --- | | Mouse mm9 chr11 98562818 98562999 + **GT** | CCCTGCAGCTGCTGCTGTGGCAAAGTGCACTATGGTCAGGACGAGAGGCCGTTCCCCTGGTCACTGTCAGCGCTCTGCCACCATCCCTGCCTCTGCCCCGAAGCTTCCTGCTTAAGTCCCTGGAGCAAGTGAGGAAGATCCAGGCCAGCGGCTCGGTGCTGCTGGAGCAGTTGGTGAGTGGG|  |  |  |  |  |  |  |  |  |  |  |  |  |  |  |  | | --- | --- | --- | --- | --- | --- | --- | --- | --- | --- | --- | --- | --- | --- | --- | --- | | Cow bosTau4 chr19 41596347 41596510 + **GG** | TCCTGCAGCTGCTCCTCTGGCACAGTGCGCTCTGGACGGTGCACGAAGCCACCCCCCTTGGCCCTGCCCGATCCCTGCCCCAGAGCTTCCTGCTCAAGTGCTTAGAGCAAGTGAGGAAAATCCAGGCTGATGGCGCCGAGCTGCAGGAGAGGCTGGTGAGTGAG|  |  |  |  |  |  |  |  | | --- | --- | --- | --- | --- | --- | --- | --- | | Dog canFam2 chr9 25859702 25859865 - **AG** | CCCTGCAGCTGCTGCTGTGGCACAGCGCACTCTGGATGGTGCAAGAAGCCGCCCCCCTGGGCCCTACCGGCCCCCTGCCCCAGAGCTTCCTGCTCAAGTGCCTAGAGCAAATGAGGAAGGTCCAGGCTGATGGCACGGCGCTGCAGGAGACGCTGGTGAGGGAG | | | | | | | | | | | | | | | | | | | | | | | | | | | | | | | | | | | | | | | | | | | | | | | | | | | | | | | | | | | | | | | | | | | | | | | | | | | | | | | | | | | | | | | | | | | |

**Alignment** (splice site sequences are in lowercase)  

```
Human      agCCCTGCAGCTGCTGCTGTGGCACAGTGCACTCTGGACAGTGCAGGAAGCCACCCCCCTGGGCCCTGCCAGC-------
Chimp      .........................................................................-------
Gorilla    .........................................................................-------
Orangutan  ...........................C.............................................-------
Rhesus     ...........................C.............................................-------
Baboon     ................T..........C.............................................-------
Marmoset   ..................A....................G............G....................-------
Lemur      ..................C........CA........C...C...A.......G........A..........-------
Galago     ......A...........C....................G.............GT.................T-------
Mouse      ........................A........A...T...GA.GA..G...GTT.......T.A...T....GCTCTGC
Cow        ..T............C..C...........G........G.....C..............T.........C.A-------
Dog        ...........................C..........TG.....A......G..............A..G..-------

Human      -----------TCCCTGCCCCAGAGCTTCCTGCTCAAGTGCTTAGAGCAAGTGAGGAAGATCCAGGGCGATGGCGCAGCG
Chimp      -----------.................................................................G...
Gorilla    -----------.................................................................G..A
Orangutan  -----------.................................................................G..A
Rhesus     -----------.........................................A.................C..T..C...
Baboon     -----------.........................................A.................C..T..C...
Marmoset   -----------..............T.................................G..............A.G.A.
Lemur      -----------................................G...........A.........A....C........C
Galago     -----------............G....T....................................AAT..A..T..G..C
Mouse      CACCATCCCTGC.T.......GA...........T....C.C.G......................C.AGC...T.G.T.
Cow        -----------...............................................A.......CT........C.A.
Dog        -----------C.............................C........A........G......CT......A.G...

Human      CTCCAGGAGAAGCTGGTGAGTGAGgt
Chimp      .........................c
Gorilla    .........................c
Orangutan  .........................c
Rhesus     ..G.........T............c
Baboon     ..G......................c
Marmoset   T.G......................c
Lemur      ..G...................G.ag
Galago     ..G........T.............g
Mouse      ..G.T....C..T.........G...
Cow        ..G.......G..............g
Dog        ..G.......C.........G...ag
```

---

## 33. uc002kms.1\_1\_6

**Summary**  

|  |  |  |  |  |  |  |  |  |  |  |  |  |  |  |  |  |  |  |  |  |  |  |  |  |  |
| --- | --- | --- | --- | --- | --- | --- | --- | --- | --- | --- | --- | --- | --- | --- | --- | --- | --- | --- | --- | --- | --- | --- | --- | --- | --- |
| No Exon ID Position (hg19) Dir Human donor Chimp donor Category Usage Gene symbol Protein accession mRNA accession Gene title Note|  |  |  |  |  |  |  |  |  |  |  |  |  | | --- | --- | --- | --- | --- | --- | --- | --- | --- | --- | --- | --- | --- | | 33 uc002kms.1\_1\_6 chr18:5406764 - GT GC (D1) shift; increase; inframe alternative EPB41L3 B3KT50 AK094952.1 erythrocyte membrane protein band 4.1-like 3  | | | | | | | | | | | | | | | | | | | | | | | | | |

**Orthologs**  

|  |  |  |  |  |  |  |  |  |  |  |  |  |  |  |  |  |  |  |  |  |  |  |  |  |  |  |  |  |  |  |  |  |  |  |  |  |  |  |  |  |  |  |  |  |  |  |  |  |  |  |  |  |  |  |  |  |  |  |  |  |  |  |  |  |  |  |  |  |  |  |  |  |  |  |  |  |  |  |  |  |  |  |  |  |  |  |  |  |  |  |  |  |  |  |  |  |  |  |  |  |  |  |  |
| --- | --- | --- | --- | --- | --- | --- | --- | --- | --- | --- | --- | --- | --- | --- | --- | --- | --- | --- | --- | --- | --- | --- | --- | --- | --- | --- | --- | --- | --- | --- | --- | --- | --- | --- | --- | --- | --- | --- | --- | --- | --- | --- | --- | --- | --- | --- | --- | --- | --- | --- | --- | --- | --- | --- | --- | --- | --- | --- | --- | --- | --- | --- | --- | --- | --- | --- | --- | --- | --- | --- | --- | --- | --- | --- | --- | --- | --- | --- | --- | --- | --- | --- | --- | --- | --- | --- | --- | --- | --- | --- | --- | --- | --- | --- | --- | --- | --- | --- | --- | --- | --- | --- | --- |
| Species Assembly Chromosome Exon start Exon end Dir Donor Exon sequence|  |  |  |  |  |  |  |  |  |  |  |  |  |  |  |  |  |  |  |  |  |  |  |  |  |  |  |  |  |  |  |  |  |  |  |  |  |  |  |  |  |  |  |  |  |  |  |  |  |  |  |  |  |  |  |  |  |  |  |  |  |  |  |  |  |  |  |  |  |  |  |  |  |  |  |  |  |  |  |  |  |  |  |  |  |  |  |  |  |  |  |  |  |  |  |  | | --- | --- | --- | --- | --- | --- | --- | --- | --- | --- | --- | --- | --- | --- | --- | --- | --- | --- | --- | --- | --- | --- | --- | --- | --- | --- | --- | --- | --- | --- | --- | --- | --- | --- | --- | --- | --- | --- | --- | --- | --- | --- | --- | --- | --- | --- | --- | --- | --- | --- | --- | --- | --- | --- | --- | --- | --- | --- | --- | --- | --- | --- | --- | --- | --- | --- | --- | --- | --- | --- | --- | --- | --- | --- | --- | --- | --- | --- | --- | --- | --- | --- | --- | --- | --- | --- | --- | --- | --- | --- | --- | --- | --- | --- | --- | --- | | Human hg19 chr18 5406764 5406940 - **GT** | ATGAAACATCAAACCAACATTAGCGAGCTGAAAAGAACCTTCTTAGAAACCTCAACAGACACTGCCGTAACGAATGAATGGGAGAAGAGGCTTTCCACCTCCCCCGTGCGACTGGCCGCCAGGCAGGAGGATGCCCCCATGATCGAACCACTTGTCCCTGAAGAGGTCAGTAGTCAG|  |  |  |  |  |  |  |  |  |  |  |  |  |  |  |  |  |  |  |  |  |  |  |  |  |  |  |  |  |  |  |  |  |  |  |  |  |  |  |  |  |  |  |  |  |  |  |  |  |  |  |  |  |  |  |  |  |  |  |  |  |  |  |  |  |  |  |  |  |  |  |  |  |  |  |  |  |  |  |  |  |  |  |  |  |  |  |  | | --- | --- | --- | --- | --- | --- | --- | --- | --- | --- | --- | --- | --- | --- | --- | --- | --- | --- | --- | --- | --- | --- | --- | --- | --- | --- | --- | --- | --- | --- | --- | --- | --- | --- | --- | --- | --- | --- | --- | --- | --- | --- | --- | --- | --- | --- | --- | --- | --- | --- | --- | --- | --- | --- | --- | --- | --- | --- | --- | --- | --- | --- | --- | --- | --- | --- | --- | --- | --- | --- | --- | --- | --- | --- | --- | --- | --- | --- | --- | --- | --- | --- | --- | --- | --- | --- | --- | --- | | Chimp panTro2 chr18 11263147 11263323 + **GC** | ATGAAACATCAAACCAACATTAGCGAGCTGAAAAGAACCTTCTTAGAAACTTCAACAGACACTGCCGTAACGAATGAATGGGAGAAGAGGCTTTCCACCTCCCCCGTGCGACTGGCCGCCAGGCAGGAGGATGCCCCCATGATCGAACCACTTGTCCCTGAAGAGGTCAGTAGTCAG|  |  |  |  |  |  |  |  |  |  |  |  |  |  |  |  |  |  |  |  |  |  |  |  |  |  |  |  |  |  |  |  |  |  |  |  |  |  |  |  |  |  |  |  |  |  |  |  |  |  |  |  |  |  |  |  |  |  |  |  |  |  |  |  |  |  |  |  |  |  |  |  |  |  |  |  |  |  |  |  | | --- | --- | --- | --- | --- | --- | --- | --- | --- | --- | --- | --- | --- | --- | --- | --- | --- | --- | --- | --- | --- | --- | --- | --- | --- | --- | --- | --- | --- | --- | --- | --- | --- | --- | --- | --- | --- | --- | --- | --- | --- | --- | --- | --- | --- | --- | --- | --- | --- | --- | --- | --- | --- | --- | --- | --- | --- | --- | --- | --- | --- | --- | --- | --- | --- | --- | --- | --- | --- | --- | --- | --- | --- | --- | --- | --- | --- | --- | --- | --- | | Gorilla gorGor1 Supercontig\_0010726 17793 17969 - **GC** | ATGAAACATCAAACCAACATTAGCGAACTGAAAAGAACCTTCTTAGAAACCTCAACAGACACTGCCNTAACAAATGAATGGGAGAAGAGGCTTTCCACCTCCCCCGTGCGACTGGCCGCCAGGCAGGAGGATGCCCCCATGATCGAACCACTTGTCCCTGAAGAGGTCAGTAGTTAG|  |  |  |  |  |  |  |  |  |  |  |  |  |  |  |  |  |  |  |  |  |  |  |  |  |  |  |  |  |  |  |  |  |  |  |  |  |  |  |  |  |  |  |  |  |  |  |  |  |  |  |  |  |  |  |  |  |  |  |  |  |  |  |  |  |  |  |  |  |  |  |  | | --- | --- | --- | --- | --- | --- | --- | --- | --- | --- | --- | --- | --- | --- | --- | --- | --- | --- | --- | --- | --- | --- | --- | --- | --- | --- | --- | --- | --- | --- | --- | --- | --- | --- | --- | --- | --- | --- | --- | --- | --- | --- | --- | --- | --- | --- | --- | --- | --- | --- | --- | --- | --- | --- | --- | --- | --- | --- | --- | --- | --- | --- | --- | --- | --- | --- | --- | --- | --- | --- | --- | --- | | Orangutan ponAbe2 chr18 26952849 26953025 + **GC** | ATGAAACATCAAACCAACATTAGCGAGCTGAAAAGAACCTTCTTAGAAACCTCAACAGACACTGCCATAACGAATGAATGGGAGAAGAGGCTTTCCACCTCCCCCGTACGACTGGCCGCCAGGCAGGAGGATGCCCCCATGATCGAACCACTTGTCCCTGAAGAGGTCAGTAGTCAG|  |  |  |  |  |  |  |  |  |  |  |  |  |  |  |  |  |  |  |  |  |  |  |  |  |  |  |  |  |  |  |  |  |  |  |  |  |  |  |  |  |  |  |  |  |  |  |  |  |  |  |  |  |  |  |  |  |  |  |  |  |  |  |  | | --- | --- | --- | --- | --- | --- | --- | --- | --- | --- | --- | --- | --- | --- | --- | --- | --- | --- | --- | --- | --- | --- | --- | --- | --- | --- | --- | --- | --- | --- | --- | --- | --- | --- | --- | --- | --- | --- | --- | --- | --- | --- | --- | --- | --- | --- | --- | --- | --- | --- | --- | --- | --- | --- | --- | --- | --- | --- | --- | --- | --- | --- | --- | --- | | Rhesus rheMac2 chr18 8880328 8880504 + **GC** | ATGAAACATCAAACCAACATTAGCGAGCTAAAAAGAACCTTCTTAGAAACCTCAACAGACACTGCCATAACGAATGAATGGGAGAAGAGGCTTTCCACCTCCCCGGTGCGACTGGCCGCCAGGCAGGAGGATGCCCCCATGATCGAACCACTTGTCCCTGAAGAGGTCAGTAGTCAG|  |  |  |  |  |  |  |  |  |  |  |  |  |  |  |  |  |  |  |  |  |  |  |  |  |  |  |  |  |  |  |  |  |  |  |  |  |  |  |  |  |  |  |  |  |  |  |  |  |  |  |  |  |  |  |  | | --- | --- | --- | --- | --- | --- | --- | --- | --- | --- | --- | --- | --- | --- | --- | --- | --- | --- | --- | --- | --- | --- | --- | --- | --- | --- | --- | --- | --- | --- | --- | --- | --- | --- | --- | --- | --- | --- | --- | --- | --- | --- | --- | --- | --- | --- | --- | --- | --- | --- | --- | --- | --- | --- | --- | --- | | Baboon papHam1 scaffold9513 20770 20946 - **GC** | ATGAAACATCAAACCAACATTAGCGAGCTAAAAAGAACCTTCTTAGAAACCTCAACAGACACTGCCATAACGAATGAATGGGAGAAGAGGCTTTCCACCTCCCCGGTGCGACTGGCCGCCAGGCAGGAGGATGCCCCCATGATCGAACCACTTGTCCCTGAAGAGGTCAGTAGTCAG|  |  |  |  |  |  |  |  |  |  |  |  |  |  |  |  |  |  |  |  |  |  |  |  |  |  |  |  |  |  |  |  |  |  |  |  |  |  |  |  |  |  |  |  |  |  |  |  | | --- | --- | --- | --- | --- | --- | --- | --- | --- | --- | --- | --- | --- | --- | --- | --- | --- | --- | --- | --- | --- | --- | --- | --- | --- | --- | --- | --- | --- | --- | --- | --- | --- | --- | --- | --- | --- | --- | --- | --- | --- | --- | --- | --- | --- | --- | --- | --- | | Marmoset calJac1 Contig2763 173492 173668 + **GC** | ATGAAACATCAAACCAACATTAGTGAGCTGAAAAGAACCTTCTTAGAAACCTCAACAGACACTGCTGTCACGAATGAATGGGAGAAGAGGCTTTCCACGTCCCCCGTGAGACTGGCCGCCAGGCAGGAGGATGCCCCCATGATCGAACCACTTGTTCCTGAAGAGGTCAGTAGTCAG|  |  |  |  |  |  |  |  |  |  |  |  |  |  |  |  |  |  |  |  |  |  |  |  |  |  |  |  |  |  |  |  |  |  |  |  |  |  |  |  | | --- | --- | --- | --- | --- | --- | --- | --- | --- | --- | --- | --- | --- | --- | --- | --- | --- | --- | --- | --- | --- | --- | --- | --- | --- | --- | --- | --- | --- | --- | --- | --- | --- | --- | --- | --- | --- | --- | --- | --- | | Tarsier tarSyr1 scaffold\_192 25712 25888 - **GC** | ATGAAACATCAAACCAACATTAGTGAGCTGAAAAGAACCTTCTTAGAAACCTCAACAGACACTGCCATAACGAATGAATGGGAAAAGAGGCTTTCTACCTCCCCAGTGCGACTAGCTGCCAGGCAGGAGGATGCCCCCATGATCGAGCCACTTGTCCCTGAAGAGGTCAGTAGTCAG|  |  |  |  |  |  |  |  |  |  |  |  |  |  |  |  |  |  |  |  |  |  |  |  |  |  |  |  |  |  |  |  | | --- | --- | --- | --- | --- | --- | --- | --- | --- | --- | --- | --- | --- | --- | --- | --- | --- | --- | --- | --- | --- | --- | --- | --- | --- | --- | --- | --- | --- | --- | --- | --- | | Galago otoGar1 scaffold\_114927.1-283004 223234 223410 - **GC** | ATGAAACACCAGACTAACATCAGTGAGCTGAAAAGAACCTTCTTAGAAACTTCAACAGACACTGCCGTCACCAATGAGTGGGAGAAGAGGCTGTCCACCTCCCCGGTGCGGCTGGCAGCCAGGCAGGAGGACGCGCCCATGATCGAGCCGCTTGTGCCTGAGGAGGTCAGTGCACGG|  |  |  |  |  |  |  |  |  |  |  |  |  |  |  |  |  |  |  |  |  |  |  |  | | --- | --- | --- | --- | --- | --- | --- | --- | --- | --- | --- | --- | --- | --- | --- | --- | --- | --- | --- | --- | --- | --- | --- | --- | | Mouse mm9 chr17 69624071 69624247 + **GC** | ATGAAGCACCAAACCAATATTAGTGAGCTGAAAAGAACCTTCTTAGAAACCTCTACAGAAACTGCCTTAACAAACGAGTGGGAAAAGAGGCTCTCTACATCTCCCGTGCGGCTGGCGGCCAGGCAGGAGGATGCACCCATGATTGAGCCGCTGGTACCCGAGGAGGTCAGTATTCTG|  |  |  |  |  |  |  |  |  |  |  |  |  |  |  |  | | --- | --- | --- | --- | --- | --- | --- | --- | --- | --- | --- | --- | --- | --- | --- | --- | | Cow bosTau4 chr24 40385828 40386004 - **GC** | ATGAAACATCAGACCAATATTAGTGAGCTGAAAAGAACCTTTTTAGAAACCTCCACAGACACTGCCATCACGAACGAGTGGGAAAAGAGGCTTTCCACCTCCCCCGTGCGACTAGCCGCCCGGCAGGAGGATGCACCCATGATCGAGCCGCTGGTACCCGAAGAGGTTGGTATTCAG|  |  |  |  |  |  |  |  | | --- | --- | --- | --- | --- | --- | --- | --- | | Dog canFam2 chr7 74830542 74830718 - **GC** | ATGAAACACCAAACCAATATTAGTGAGCTGAAAAGAACCTTTTTAGAAACCTCCACAGACACTGCCATCACGAATGAATGGGAAAAGAGGCTTTCTACTTCCCCAGTGCGACTAGCCGCCAGGCAGGAGGACGCGCCCATGATCGAACCACTTGTACCTGAAGAGGTCGGTATTCGG | | | | | | | | | | | | | | | | | | | | | | | | | | | | | | | | | | | | | | | | | | | | | | | | | | | | | | | | | | | | | | | | | | | | | | | | | | | | | | | | | | | | | | | | | | | |

**Alignment** (splice site sequences are in lowercase)  

```
Human      ATGAAACATCAAACCAACATTAGCGAGCTGAAAAGAACCTTCTTAGAAACCTCAACAGACACTGCCGTAACGAATGAATG
Chimp      ..................................................T.............................
Gorilla    ..........................A.......................................N....A........
Orangutan  ..................................................................A.............
Rhesus     .............................A....................................A.............
Baboon     .............................A....................................A.............
Marmoset   .......................T.........................................T..C...........
Tarsier    .......................T..........................................A.............
Galago     ........C..G..T.....C..T..........................T.................C..C.....G..
Mouse      .....G..C........T.....T.............................T.....A......T....A..C..G..
Cow        ...........G.....T.....T.................T...........C............A.C.....C..G..
Dog        ........C........T.....T.................T...........C............A.C...........

Human      GGAGAAGAGGCTTTCCACCTCCCCCGTGCGACTGGCCGCCAGGCAGGAGGATGCCCCCATGATCGAACCACTTGTCCCTG
Chimp      ................................................................................
Gorilla    ................................................................................
Orangutan  ...........................A....................................................
Rhesus     ........................G.......................................................
Baboon     ........................G.......................................................
Marmoset   ..................G.........A..............................................T....
Tarsier    ...A...........T........A........A..T.............................G.............
Galago     ............G...........G.....G.....A..............C..G...........G..G.....G....
Mouse      ...A........C..T..A..T........G.....G.................A........T..G..G..G..A..C.
Cow        ...A.............................A......C.............A...........G..G..G..A..C.
Dog        ...A...........T..T.....A........A.................C..G....................A....

Human      AAGAGGTCAGTAGTCAGgt
Chimp      ..................c
Gorilla    ..............T...c
Orangutan  ..................c
Rhesus     ..................c
Baboon     ..................c
Marmoset   ..................c
Tarsier    ..................c
Galago     .G.........GCA.G..c
Mouse      .G..........T..T..c
Cow        .......TG...T.....c
Dog        ........G...T..G..c
```

---

## 34. uc010dpa.2\_1\_7

**Summary**  

|  |  |  |  |  |  |  |  |  |  |  |  |  |  |  |  |  |  |  |  |  |  |  |  |  |  |
| --- | --- | --- | --- | --- | --- | --- | --- | --- | --- | --- | --- | --- | --- | --- | --- | --- | --- | --- | --- | --- | --- | --- | --- | --- | --- |
| No Exon ID Position (hg19) Dir Human donor Chimp donor Category Usage Gene symbol Protein accession mRNA accession Gene title Note|  |  |  |  |  |  |  |  |  |  |  |  |  | | --- | --- | --- | --- | --- | --- | --- | --- | --- | --- | --- | --- | --- | | 34 uc010dpa.2\_1\_7 chr18:48346267 - GT GG (D7) exonization; novel start alternative MRO NP\_001120648.1 NM\_001127176.1 maestro  | | | | | | | | | | | | | | | | | | | | | | | | | |

**Orthologs**  

|  |  |  |  |  |  |  |  |  |  |  |  |  |  |  |  |  |  |  |  |  |  |  |  |  |  |  |  |  |  |  |  |  |  |  |  |  |  |  |  |  |  |  |  |  |  |  |  |  |  |  |  |  |  |  |  |  |  |  |  |  |  |  |  |  |  |  |  |  |  |  |  |  |  |  |  |  |  |  |  |
| --- | --- | --- | --- | --- | --- | --- | --- | --- | --- | --- | --- | --- | --- | --- | --- | --- | --- | --- | --- | --- | --- | --- | --- | --- | --- | --- | --- | --- | --- | --- | --- | --- | --- | --- | --- | --- | --- | --- | --- | --- | --- | --- | --- | --- | --- | --- | --- | --- | --- | --- | --- | --- | --- | --- | --- | --- | --- | --- | --- | --- | --- | --- | --- | --- | --- | --- | --- | --- | --- | --- | --- | --- | --- | --- | --- | --- | --- | --- | --- |
| Species Assembly Chromosome Exon start Exon end Dir Donor Exon sequence|  |  |  |  |  |  |  |  |  |  |  |  |  |  |  |  |  |  |  |  |  |  |  |  |  |  |  |  |  |  |  |  |  |  |  |  |  |  |  |  |  |  |  |  |  |  |  |  |  |  |  |  |  |  |  |  |  |  |  |  |  |  |  |  |  |  |  |  |  |  |  |  | | --- | --- | --- | --- | --- | --- | --- | --- | --- | --- | --- | --- | --- | --- | --- | --- | --- | --- | --- | --- | --- | --- | --- | --- | --- | --- | --- | --- | --- | --- | --- | --- | --- | --- | --- | --- | --- | --- | --- | --- | --- | --- | --- | --- | --- | --- | --- | --- | --- | --- | --- | --- | --- | --- | --- | --- | --- | --- | --- | --- | --- | --- | --- | --- | --- | --- | --- | --- | --- | --- | --- | --- | | Human hg19 chr18 48346267 48346285 - **GT** | ATGGCGTGGGGAAGCAAAG|  |  |  |  |  |  |  |  |  |  |  |  |  |  |  |  |  |  |  |  |  |  |  |  |  |  |  |  |  |  |  |  |  |  |  |  |  |  |  |  |  |  |  |  |  |  |  |  |  |  |  |  |  |  |  |  |  |  |  |  |  |  |  |  | | --- | --- | --- | --- | --- | --- | --- | --- | --- | --- | --- | --- | --- | --- | --- | --- | --- | --- | --- | --- | --- | --- | --- | --- | --- | --- | --- | --- | --- | --- | --- | --- | --- | --- | --- | --- | --- | --- | --- | --- | --- | --- | --- | --- | --- | --- | --- | --- | --- | --- | --- | --- | --- | --- | --- | --- | --- | --- | --- | --- | --- | --- | --- | --- | | Chimp panTro2 chr18 47083331 47083349 - **GG** | ATGGCGTGGGGAAGCAAAG|  |  |  |  |  |  |  |  |  |  |  |  |  |  |  |  |  |  |  |  |  |  |  |  |  |  |  |  |  |  |  |  |  |  |  |  |  |  |  |  |  |  |  |  |  |  |  |  |  |  |  |  |  |  |  |  | | --- | --- | --- | --- | --- | --- | --- | --- | --- | --- | --- | --- | --- | --- | --- | --- | --- | --- | --- | --- | --- | --- | --- | --- | --- | --- | --- | --- | --- | --- | --- | --- | --- | --- | --- | --- | --- | --- | --- | --- | --- | --- | --- | --- | --- | --- | --- | --- | --- | --- | --- | --- | --- | --- | --- | --- | | Gorilla gorGor1 Supercontig\_0007196 18978 18996 - **GG** | ATGGCGTAGGGAAGCAAAG|  |  |  |  |  |  |  |  |  |  |  |  |  |  |  |  |  |  |  |  |  |  |  |  |  |  |  |  |  |  |  |  |  |  |  |  |  |  |  |  |  |  |  |  |  |  |  |  | | --- | --- | --- | --- | --- | --- | --- | --- | --- | --- | --- | --- | --- | --- | --- | --- | --- | --- | --- | --- | --- | --- | --- | --- | --- | --- | --- | --- | --- | --- | --- | --- | --- | --- | --- | --- | --- | --- | --- | --- | --- | --- | --- | --- | --- | --- | --- | --- | | Orangutan ponAbe2 chr18 63241274 63241292 - **GG** | ATGTCGTGGGAAAGCAAAG|  |  |  |  |  |  |  |  |  |  |  |  |  |  |  |  |  |  |  |  |  |  |  |  |  |  |  |  |  |  |  |  |  |  |  |  |  |  |  |  | | --- | --- | --- | --- | --- | --- | --- | --- | --- | --- | --- | --- | --- | --- | --- | --- | --- | --- | --- | --- | --- | --- | --- | --- | --- | --- | --- | --- | --- | --- | --- | --- | --- | --- | --- | --- | --- | --- | --- | --- | | Rhesus rheMac2 chr18 43915689 43915707 - **GG** | ATGGCGTGGGGAAGCAAAG|  |  |  |  |  |  |  |  |  |  |  |  |  |  |  |  |  |  |  |  |  |  |  |  |  |  |  |  |  |  |  |  | | --- | --- | --- | --- | --- | --- | --- | --- | --- | --- | --- | --- | --- | --- | --- | --- | --- | --- | --- | --- | --- | --- | --- | --- | --- | --- | --- | --- | --- | --- | --- | --- | | Baboon papHam1 scaffold5166 31203 31221 + **GG** | ATGGCGTGGGGAAGCAAAG|  |  |  |  |  |  |  |  |  |  |  |  |  |  |  |  |  |  |  |  |  |  |  |  | | --- | --- | --- | --- | --- | --- | --- | --- | --- | --- | --- | --- | --- | --- | --- | --- | --- | --- | --- | --- | --- | --- | --- | --- | | Marmoset calJac1 Contig56 634633 634651 + **GG** | AAGGCGTGGGGAAGCCAAG|  |  |  |  |  |  |  |  |  |  |  |  |  |  |  |  | | --- | --- | --- | --- | --- | --- | --- | --- | --- | --- | --- | --- | --- | --- | --- | --- | | Lemur micMur1 scaffold\_9903 21889 21907 - **GG** | ACAGCGAGGGCAAGCAAAG|  |  |  |  |  |  |  |  | | --- | --- | --- | --- | --- | --- | --- | --- | | Galago otoGar1 scaffold\_60966.1-8252 4401 4419 - **GG** | ACAGTGAAGGGAAGCAAAG | | | | | | | | | | | | | | | | | | | | | | | | | | | | | | | | | | | | | | | | | | | | | | | | | | | | | | | | | | | | | | | | | | | | | | |

**Alignment** (splice site sequences are in lowercase)  

```
Human      ATGGCGTGGGGAAGCAAAGgt
Chimp      ....................g
Gorilla    .......A............g
Orangutan  ...T......A.........g
Rhesus     ....................g
Baboon     ....................g
Marmoset   .A.............C....g
Lemur      .CA...A...C.........g
Galago     .CA.T.AA............g
```

---

## 35. uc010zuc.1\_1\_15

**Summary**  

|  |  |  |  |  |  |  |  |  |  |  |  |  |  |  |  |  |  |  |  |  |  |  |  |  |  |
| --- | --- | --- | --- | --- | --- | --- | --- | --- | --- | --- | --- | --- | --- | --- | --- | --- | --- | --- | --- | --- | --- | --- | --- | --- | --- |
| No Exon ID Position (hg19) Dir Human donor Chimp donor Category Usage Gene symbol Protein accession mRNA accession Gene title Note|  |  |  |  |  |  |  |  |  |  |  |  |  | | --- | --- | --- | --- | --- | --- | --- | --- | --- | --- | --- | --- | --- | | 35 uc010zuc.1\_1\_15 chr20:31619550 + GT GC (D1) shift; increase; inframe alternative BPIFB6 NP\_777557.1 NM\_174897.2 BPI fold containing family B, member 6 GTNGTN | | | | | | | | | | | | | | | | | | | | | | | | | |

**Orthologs**  

|  |  |  |  |  |  |  |  |  |  |  |  |  |  |  |  |  |  |  |  |  |  |  |  |  |  |  |  |  |  |  |  |  |  |  |  |  |  |  |  |  |  |  |  |  |  |  |  |  |  |  |  |  |  |  |  |  |  |  |  |  |  |  |  |  |  |  |  |  |  |  |  |  |  |  |  |  |  |  |  |  |  |  |  |  |  |  |  |  |  |  |  |  |  |  |  |  |  |  |  |  |  |  |  |
| --- | --- | --- | --- | --- | --- | --- | --- | --- | --- | --- | --- | --- | --- | --- | --- | --- | --- | --- | --- | --- | --- | --- | --- | --- | --- | --- | --- | --- | --- | --- | --- | --- | --- | --- | --- | --- | --- | --- | --- | --- | --- | --- | --- | --- | --- | --- | --- | --- | --- | --- | --- | --- | --- | --- | --- | --- | --- | --- | --- | --- | --- | --- | --- | --- | --- | --- | --- | --- | --- | --- | --- | --- | --- | --- | --- | --- | --- | --- | --- | --- | --- | --- | --- | --- | --- | --- | --- | --- | --- | --- | --- | --- | --- | --- | --- | --- | --- | --- | --- | --- | --- | --- | --- |
| Species Assembly Chromosome Exon start Exon end Dir Donor Exon sequence|  |  |  |  |  |  |  |  |  |  |  |  |  |  |  |  |  |  |  |  |  |  |  |  |  |  |  |  |  |  |  |  |  |  |  |  |  |  |  |  |  |  |  |  |  |  |  |  |  |  |  |  |  |  |  |  |  |  |  |  |  |  |  |  |  |  |  |  |  |  |  |  |  |  |  |  |  |  |  |  |  |  |  |  |  |  |  |  |  |  |  |  |  |  |  |  | | --- | --- | --- | --- | --- | --- | --- | --- | --- | --- | --- | --- | --- | --- | --- | --- | --- | --- | --- | --- | --- | --- | --- | --- | --- | --- | --- | --- | --- | --- | --- | --- | --- | --- | --- | --- | --- | --- | --- | --- | --- | --- | --- | --- | --- | --- | --- | --- | --- | --- | --- | --- | --- | --- | --- | --- | --- | --- | --- | --- | --- | --- | --- | --- | --- | --- | --- | --- | --- | --- | --- | --- | --- | --- | --- | --- | --- | --- | --- | --- | --- | --- | --- | --- | --- | --- | --- | --- | --- | --- | --- | --- | --- | --- | --- | --- | | Human hg19 chr20 31619454 31619550 + **GT** | ATGCTGCGGATCCTGTGCCTGGCACTCTGCAGCCTGCTGACTGGCACGCGAGCTGACCCTGGGGCACTGCTGCGGTTGGGCATGGACATCATGAACC|  |  |  |  |  |  |  |  |  |  |  |  |  |  |  |  |  |  |  |  |  |  |  |  |  |  |  |  |  |  |  |  |  |  |  |  |  |  |  |  |  |  |  |  |  |  |  |  |  |  |  |  |  |  |  |  |  |  |  |  |  |  |  |  |  |  |  |  |  |  |  |  |  |  |  |  |  |  |  |  |  |  |  |  |  |  |  |  | | --- | --- | --- | --- | --- | --- | --- | --- | --- | --- | --- | --- | --- | --- | --- | --- | --- | --- | --- | --- | --- | --- | --- | --- | --- | --- | --- | --- | --- | --- | --- | --- | --- | --- | --- | --- | --- | --- | --- | --- | --- | --- | --- | --- | --- | --- | --- | --- | --- | --- | --- | --- | --- | --- | --- | --- | --- | --- | --- | --- | --- | --- | --- | --- | --- | --- | --- | --- | --- | --- | --- | --- | --- | --- | --- | --- | --- | --- | --- | --- | --- | --- | --- | --- | --- | --- | --- | --- | | Chimp panTro2 chr20 30013963 30014059 + **GC** | ATGCTGCGGATCCTGTGCCTGGCACTCTGCAGCCTGCTGACTGGCATGCGAGCTGACCCTGGGGCACTGCTGCGGTTGGGCATGGACATCATGAACC|  |  |  |  |  |  |  |  |  |  |  |  |  |  |  |  |  |  |  |  |  |  |  |  |  |  |  |  |  |  |  |  |  |  |  |  |  |  |  |  |  |  |  |  |  |  |  |  |  |  |  |  |  |  |  |  |  |  |  |  |  |  |  |  |  |  |  |  |  |  |  |  |  |  |  |  |  |  |  |  | | --- | --- | --- | --- | --- | --- | --- | --- | --- | --- | --- | --- | --- | --- | --- | --- | --- | --- | --- | --- | --- | --- | --- | --- | --- | --- | --- | --- | --- | --- | --- | --- | --- | --- | --- | --- | --- | --- | --- | --- | --- | --- | --- | --- | --- | --- | --- | --- | --- | --- | --- | --- | --- | --- | --- | --- | --- | --- | --- | --- | --- | --- | --- | --- | --- | --- | --- | --- | --- | --- | --- | --- | --- | --- | --- | --- | --- | --- | --- | --- | | Gorilla gorGor1 Supercontig\_0004547 33444 33540 - **GC** | ATGCTGCGGATCCTGTGCCTGGCACTCTGCAGCCTGCTGACTGGCACGCGAGCTGACCCTGGGGCACTGCTGCGGTTGGGCATGGACATCATGAACC|  |  |  |  |  |  |  |  |  |  |  |  |  |  |  |  |  |  |  |  |  |  |  |  |  |  |  |  |  |  |  |  |  |  |  |  |  |  |  |  |  |  |  |  |  |  |  |  |  |  |  |  |  |  |  |  |  |  |  |  |  |  |  |  |  |  |  |  |  |  |  |  | | --- | --- | --- | --- | --- | --- | --- | --- | --- | --- | --- | --- | --- | --- | --- | --- | --- | --- | --- | --- | --- | --- | --- | --- | --- | --- | --- | --- | --- | --- | --- | --- | --- | --- | --- | --- | --- | --- | --- | --- | --- | --- | --- | --- | --- | --- | --- | --- | --- | --- | --- | --- | --- | --- | --- | --- | --- | --- | --- | --- | --- | --- | --- | --- | --- | --- | --- | --- | --- | --- | --- | --- | | Orangutan ponAbe2 chr20\_random 5105759 5105855 + **GC** | ATGCTGAGGATCCTGTGCCTGGCCCTCTGCAGCCTGCTGACTGGCACGCGAGCTGACCCTGGGGCACTGCTGAGGTTGGGCATGGACATCATGAACC|  |  |  |  |  |  |  |  |  |  |  |  |  |  |  |  |  |  |  |  |  |  |  |  |  |  |  |  |  |  |  |  |  |  |  |  |  |  |  |  |  |  |  |  |  |  |  |  |  |  |  |  |  |  |  |  |  |  |  |  |  |  |  |  | | --- | --- | --- | --- | --- | --- | --- | --- | --- | --- | --- | --- | --- | --- | --- | --- | --- | --- | --- | --- | --- | --- | --- | --- | --- | --- | --- | --- | --- | --- | --- | --- | --- | --- | --- | --- | --- | --- | --- | --- | --- | --- | --- | --- | --- | --- | --- | --- | --- | --- | --- | --- | --- | --- | --- | --- | --- | --- | --- | --- | --- | --- | --- | --- | | Rhesus rheMac2 chr10 31516584 31516680 - **GC** | ATGCTGCGGATCCTGTGCCTGGCACTCTGCAGCCTGCTGACCCACGCGCGAGCTGACCCTGGGGCACTGCTGCGGTTGGGCATGGACATCATGAACC|  |  |  |  |  |  |  |  |  |  |  |  |  |  |  |  |  |  |  |  |  |  |  |  |  |  |  |  |  |  |  |  |  |  |  |  |  |  |  |  |  |  |  |  |  |  |  |  |  |  |  |  |  |  |  |  | | --- | --- | --- | --- | --- | --- | --- | --- | --- | --- | --- | --- | --- | --- | --- | --- | --- | --- | --- | --- | --- | --- | --- | --- | --- | --- | --- | --- | --- | --- | --- | --- | --- | --- | --- | --- | --- | --- | --- | --- | --- | --- | --- | --- | --- | --- | --- | --- | --- | --- | --- | --- | --- | --- | --- | --- | | Baboon papHam1 scaffold5329 64718 64814 + **GC** | ATGCTGCGGATCCTGTGCCTGGCACTCTGCAGCCTGCTGACCCACGTGCGAGCTGACCCTGGGGCACTGCTGCGGTTGGGCATGGACATCATGAATC|  |  |  |  |  |  |  |  |  |  |  |  |  |  |  |  |  |  |  |  |  |  |  |  |  |  |  |  |  |  |  |  |  |  |  |  |  |  |  |  |  |  |  |  |  |  |  |  | | --- | --- | --- | --- | --- | --- | --- | --- | --- | --- | --- | --- | --- | --- | --- | --- | --- | --- | --- | --- | --- | --- | --- | --- | --- | --- | --- | --- | --- | --- | --- | --- | --- | --- | --- | --- | --- | --- | --- | --- | --- | --- | --- | --- | --- | --- | --- | --- | | Marmoset calJac1 Contig2380 4972 5068 + **AC** | ATGCTGCGGATCCTGGGCCTGGCACTCTGCAGCCTGCTGACCTGCACGCAAGCCGAGCCTGGGGCCCTGCTGCGGCTGGGCATGGACATCATGAACC|  |  |  |  |  |  |  |  |  |  |  |  |  |  |  |  |  |  |  |  |  |  |  |  |  |  |  |  |  |  |  |  |  |  |  |  |  |  |  |  | | --- | --- | --- | --- | --- | --- | --- | --- | --- | --- | --- | --- | --- | --- | --- | --- | --- | --- | --- | --- | --- | --- | --- | --- | --- | --- | --- | --- | --- | --- | --- | --- | --- | --- | --- | --- | --- | --- | --- | --- | | Lemur micMur1 scaffold\_1836 158643 158739 + **GA** | ATGCTTCGGATCCTGTGTCTGGCACTCTGCAGCCTGCTGAGCGGCGCTCGAGCTGACCCGGGGGCGCTGCTGCGCTTGGGCATGGACGTCATGAACC|  |  |  |  |  |  |  |  |  |  |  |  |  |  |  |  |  |  |  |  |  |  |  |  |  |  |  |  |  |  |  |  | | --- | --- | --- | --- | --- | --- | --- | --- | --- | --- | --- | --- | --- | --- | --- | --- | --- | --- | --- | --- | --- | --- | --- | --- | --- | --- | --- | --- | --- | --- | --- | --- | | Galago otoGar1 scaffold\_100622.1-7485 2933 3029 + **GC** | ATGCTGCGGATCCTATGTTTGGCACTCTGTGGCCTACTGACCGGCACACGAGCAGACCCTGGGGCGCTGCTGCGCTTGGGCATGGACATCATGAACC|  |  |  |  |  |  |  |  |  |  |  |  |  |  |  |  |  |  |  |  |  |  |  |  | | --- | --- | --- | --- | --- | --- | --- | --- | --- | --- | --- | --- | --- | --- | --- | --- | --- | --- | --- | --- | --- | --- | --- | --- | | Mouse mm9 chr2 153728377 153728473 + **AT** | ATGCTGTGTAGCCTGAGCCTGGTCCTCTGCGGCCTGCTGGCTGGCACTCGAGCTGACCCTGGGGGTCTTCTGAGGTTGGGCATGGACATTATGAACC|  |  |  |  |  |  |  |  |  |  |  |  |  |  |  |  | | --- | --- | --- | --- | --- | --- | --- | --- | --- | --- | --- | --- | --- | --- | --- | --- | | Cow bosTau4 chr13 63203323 63203419 + **GC** | ATGCTGTGGATCCTGTGTCTGGCGCTCTGCAGCCTGCTGACTCCCACGCGAGCAGACCCTGGGGCACTGCTGCGGCTGGGCATGGACGTCCTGAATC|  |  |  |  |  |  |  |  | | --- | --- | --- | --- | --- | --- | --- | --- | | Dog canFam2 chr24 25272426 25272522 + **AC** | ATGCTGTGGATCCTGTTTCTGGCACTCTGTGGCCTGCTGACCCACACCCGAGCTGATCCCGGGGCATTGCTGAGGCTGGGCATGGACGTCATGAACC | | | | | | | | | | | | | | | | | | | | | | | | | | | | | | | | | | | | | | | | | | | | | | | | | | | | | | | | | | | | | | | | | | | | | | | | | | | | | | | | | | | | | | | | | | | |

**Alignment** (splice site sequences are in lowercase)  

```
Human      ATGCTGCGGATCCTGTGCCTGGCACTCTGCAGCCTGCTGACTGGCACGCGAGCTGACCCTGGGGCACTGCTGCGGTTGGG
Chimp      ..............................................T.................................
Gorilla    ................................................................................
Orangutan  ......A................C................................................A.......
Rhesus     .........................................CCA.G..................................
Baboon     .........................................CCA.GT.................................
Marmoset   ...............G.........................CT......A...C..G........C.........C....
Lemur      .....T...........T......................GC...G.T...........G.....G........C.....
Galago     ..............A..TT..........TG....A.....C.....A.....A...........G........C.....
Mouse      ......T.T.G....A......TC......G........G.......T................GT..T...A.......
Cow        ......T..........T.....G..................CC.........A.....................C....
Dog        ......T.........TT...........TG..........CCA...C........T..C......T.....A..C....

Human      CATGGACATCATGAACCgt
Chimp      ..................c
Gorilla    ..................c
Orangutan  ..................c
Rhesus     ..................c
Baboon     ...............T..c
Marmoset   .................ac
Lemur      .......G..........a
Galago     ..................c
Mouse      .........T.......a.
Cow        .......G..C....T..c
Dog        .......G.........ac
```

---
